# Supplementary material for: Ectopic Expression of AeNAC83, a NAC Transcription Factor from Abelmoschus esculentus, Inhibits Growth and Confers Tolerance to Salt Stress in Arabidopsis
Source: Int J Mol Sci. 2022 Sep 5;23(17):10182. doi: 10.3390/ijms231710182 (PMC9456028; doi:10.3390/ijms231710182)
Supplement: Supplementary file 1 [file ijms-23-10182-s001.zip › Figure S2.pdf]

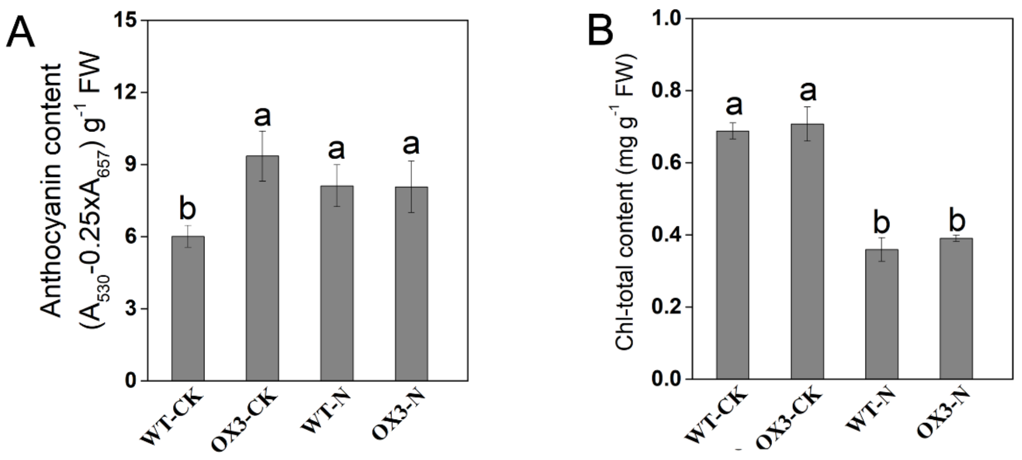

Figure S2. Effect of NaCl treatment on anthocyanin content (**A**) and total chlorophyll content (**B**) of WT (Col-0) and AeNAC83-overexpression transgenic (OX3) seedlings.
