# Supplementary material for: Ectopic Expression of AeNAC83, a NAC Transcription Factor from Abelmoschus esculentus, Inhibits Growth and Confers Tolerance to Salt Stress in Arabidopsis
Source: Int J Mol Sci. 2022 Sep 5;23(17):10182. doi: 10.3390/ijms231710182 (PMC9456028; doi:10.3390/ijms231710182)
Supplement: Supplementary file 1 [file ijms-23-10182-s001.zip › Table S1.pdf]

Table S1. Illumina-seq output statistics of 12 samples.

Sample	Clean Reads	Clean Bases	Q20	Q30	GC content
WT-CK 1	22,831,614	6,823,195,164	97.88	94.37	46.05
WT-CK 2	20,879,412	6,237,486,148	97.72	94.00	45.83
WT-CK 3	19,297,979	5,771,005,098	97.93	94.49	45.72
OX3-CK 1	19,190,301	5,740,086,572	97.74	94.11	45.60
OX3-CK 2	21,141,667	6,322,250,946	97.72	94.04	45.59
OX3-CK 3	20,570,137	6,153,537,524	97.86	94.40	45.61
WT-N 1	19,468,147	5,799,741,630	97.77	94.19	45.39
WT-N 2	21,085,699	6,284,851,384	97.62	93.96	46.12
WT-N 3	19,239,340	5,737,199,530	97.61	93.91	45.36
OX3-N 1	20,933,549	6,256,654,336	97.85	94.36	45.56
OX3-N 2	19,311,053	5,773,478,276	97.77	94.19	45.53
OX3-N 3	21,592,092	6,453,601,894	97.82	94.28	45.57
