# Supplementary material for: Ectopic Expression of AeNAC83, a NAC Transcription Factor from Abelmoschus esculentus, Inhibits Growth and Confers Tolerance to Salt Stress in Arabidopsis
Source: Int J Mol Sci. 2022 Sep 5;23(17):10182. doi: 10.3390/ijms231710182 (PMC9456028; doi:10.3390/ijms231710182)
Supplement: Supplementary file 1 [file ijms-23-10182-s001.zip › Table S2.pdf]

Table S2. All DEGs in four comparison groups were listed.

geneID	N83-CK1	N83-CK2	N83-CK3	N83-N1	N83-N2	N83-N3	WT-CK1	WT-CK2
gene-AT2G	4.577599	4.975858	3.933429	5.560058	6.019353	5.927066	1.290866	1.488556
gene-AT5G	10.56374	10.71535	11.02476	10.68624	9.438	10.28437	7.412613	7.815142
gene-AT1G	3.479214	2.875879	2.738198	0.66187	0.683057	0.491823	1.294455	1.42393
gene-AT5G	11.30281	10.96007	11.0497	32.02547	28.31489	31.04313	9.359926	10.38881
gene-AT1G	2.401339	2.197073	3.245059	0.633904	0.796236	0.902389	4.225217	4.313984
gene-AT1G	1.637659	2.883839	3.844619	3.432486	2.668509	3.893561	7.5683	6.512756
gene-AT2G	3.467044	4.353713	5.073214	1.125255	1.600141	1.936968	9.371178	7.199521
gene-AT5G	11.32688	10.15011	11.1659	11.25886	9.959128	10.47751	3.582478	3.211659
gene-AT5G	8.179133	9.851901	9.577751	10.07643	8.350603	8.732809	12.15099	11.67314
gene-AT2G	16.17506	15.42004	16.24733	8.688294	8.642314	9.135255	24.31232	27.00973
gene-AT2G	63.1437	60.59316	65.58495	58.76346	53.20762	54.26329	92.46497	91.40608
gene-AT2G	1.890157	2.321922	2.244958	3.30962	3.750779	3.58306	3.315152	3.170119
gene-AT1G	0.808046	0.759825	0.622289	2.132633	2.614071	2.35977	1.685832	1.841305
gene-AT2G	754.1722	785.3731	756.7799	472.4468	450.5837	468.8393	1236.634	1174.321
gene-AT3G	6.752603	6.427538	7.749687	7.607152	8.528473	10.64255	6.106005	8.494687
gene-AT3G	28.84082	28.29679	32.48663	18.07122	15.34129	14.66354	10.24029	10.54621
gene-AT3G	15.00016	16.00021	15.55584	10.24454	10.03954	9.419615	22.05425	21.57357
gene-AT2G	93.68265	99.51715	101.0284	213.818	219.5144	218.1868	92.32333	99.78637
gene-AT1G	46.59234	43.53997	44.50977	21.87727	20.40475	22.407	47.73658	47.84626
gene-AT2G	33.60972	34.73193	36.12122	23.76682	25.5125	25.04511	13.54617	13.65342
gene-AT1G	22.7546	23.12154	20.19481	17.30965	15.35308	15.92768	22.65891	21.03701
gene-AT5G	23.1459	23.88031	24.08121	25.62619	26.01608	26.53387	27.0256	26.49906
gene-AT5G	0.753574	0.914216	0.904077	4.218724	3.714456	4.27199	2.462121	2.71205
gene-AT2G	65.22364	65.66919	66.85492	18.08013	18.1155	19.64747	30.68832	34.79509
gene-AT1G	2.535472	2.294016	3.299194	6.063204	5.778117	5.885484	2.586507	2.859922
Arabidopsi	0.583116	0.830652	0.813849	0.368474	0.401124	0.483441	0.190062	0.383602
gene-AT3G	0.531041	0.375052	0.346741	0.326538	0.325052	0.768357	0.215632	0.427839
gene-AT1G	0.881683	0.713609	0.559316	2.509634	2.004399	1.334926	1.149717	0.775279
gene-AT4G	6.656528	5.818449	6.130471	0.824746	1.203278	1.413861	0	0.042229
gene-AT2G	95.24638	84.17063	87.1061	53.97879	66.19451	64.40684	18.0251	20.22157
gene-AT3G	18.81039	18.54001	19.38654	13.62078	15.91566	14.21684	29.20958	30.48956
gene-AT5G	3.341695	3.006459	3.622417	7.107901	7.856015	5.860406	2.844847	2.319732
gene-AT4G	25.16353	23.46742	24.23093	45.17174	48.67527	47.2971	14.85211	14.8916
gene-AT4G	0.526217	0.186966	0.29516	0.985131	1.108659	1.339762	0.205317	0.133357
gene-AT3G	6.425933	5.013836	6.446245	5.256896	4.835364	4.765589	11.06124	11.45725
gene-AT4G	7.844226	6.668251	7.423862	8.839018	9.615707	7.939143	24.64285	28.62488
gene-AT2G	4.451075	5.381802	6.062297	2.803252	2.769503	3.757449	5.717975	6.224804
gene-AT5G	2.219392	2.899726	2.832852	1.58876	0.897783	1.714736	0.437304	0.64576
gene-AT1G	20.52831	20.01624	20.24788	15.15404	12.38661	13.77305	26.40242	28.76178
gene-AT2G	1.143134	1.105704	1.648869	1.158815	0.851843	1.346197	0.765653	0.97699
gene-AT1G	2.59471	1.715062	2.670758	2.473204	1.225871	1.643076	4.240771	3.916312
gene-AT1G	6.903265	6.058368	5.352623	2.98377	2.829616	3.320211	6.764503	6.335777
gene-AT5G	5.457744	5.438921	4.754151	4.243247	5.255085	4.413672	6.241005	5.955097
gene-AT5G	1.464023	1.634955	1.825236	2.442728	1.939277	2.78348	0.690503	0.661341
gene-AT3G	2.510457	2.245968	2.057409	2.871495	2.699549	2.835646	0.701741	0.884647
gene-AT4G	0.264043	0.706924	0.641712	1.402344	1.209563	1.980322	0.479191	1.054478
gene-AT5G	11.14597	11.50617	13.00832	10.39022	10.10041	11.67732	23.84996	22.15534
gene-AT4G	0.545339	0.705439	0.5977	0.409678	0.939272	0.730006	0.938032	0.572052
gene-AT1G	5.927229	6.471956	5.407688	7.597126	7.496008	5.817646	10.34914	11.28758

gene-AT5G	8.994969	8.328506	9.692565	5.171298	6.596332	7.154352	10.42772	10.67618
gene-AT4G	4.32456	3.498191	3.649502	5.775964	5.562452	5.832437	11.33321	11.92855
gene-AT3G	0.404097	0.683371	0.276735	1.43482	1.861068	1.88259	0.759083	0.513523
gene-AT3G	19.00628	20.46723	19.74325	16.15379	16.77297	14.50747	27.21676	26.6578
gene-AT4G	14.69844	15.21873	13.86288	13.21566	12.64072	12.51186	8.438023	8.304382
gene-AT2G	0.400612	0.307389	0.289532	0.528795	0.162238	0.418916	0.277093	0.528539
gene-AT3G	9.624415	9.728236	7.443127	9.007657	9.777651	7.323987	4.967819	3.742217
gene-AT5G	45.40014	42.82619	44.16167	74.90027	75.37183	72.93548	40.16959	45.19968
gene-AT4G	7.815086	7.451686	8.037931	9.845583	8.615165	9.760102	5.602573	6.395146
gene-AT1G	0.935753	0.709016	0.584638	1.549558	2.10789	1.816394	0.984775	0.810788
gene-AT2G	0.613222	0.550952	0.536714	1.100635	2.001061	1.173864	0.113161	0.199234
gene-AT5G	11.32907	9.203467	10.73888	7.140362	5.219883	9.208457	0.863873	1.167159
gene-AT4G	1.85466	1.803224	1.735937	0.32094	0.421936	0.52469	1.290509	2.115643
gene-AT1G	45.13566	46.56791	44.58051	40.95488	42.39653	42.75976	60.8641	62.45107
gene-AT5G	0.838387	2.02095	1.332929	1.828593	2.079529	1.829176	1.532208	0.628839
gene-AT1G	4.512066	6.640485	6.173859	4.22727	3.30599	4.024535	6.86031	4.442848
gene-AT4G	18.49392	20.01385	20.51766	13.69317	12.6939	14.22481	27.3242	26.73573
gene-AT1G	4.568051	5.838507	6.486374	15.35184	15.73878	15.82232	5.451475	5.085068
gene-AT1G	2.390301	3.122245	3.005499	4.537783	3.866972	5.085478	1.367564	1.447567
gene-AT2G	0	0.14302	0.329351	0.256833	0.124736	0.268889	0.278888	0.203198
gene-AT4G	243.1942	238.3583	244.6469	193.3188	191.2032	191.5679	336.7663	361.8965
gene-AT3G	182.9579	183.5947	181.9917	80.08539	79.58005	81.52477	67.74532	68.53971
gene-AT5G	16.61643	14.06542	14.60005	15.76829	15.38866	15.41629	23.74495	23.41752
gene-AT1G	2.337005	2.072643	1.882199	1.791123	1.436324	1.387023	0.495287	0.665431
gene-AT2G	12.57409	14.63106	14.86873	11.31329	10.85123	11.11255	17.82575	21.18446
gene-AT4G	5.346705	6.760986	6.371969	9.4312	9.26893	9.096456	3.82019	3.151201
gene-AT2G	16.61623	18.88748	16.66707	14.73489	16.4123	14.80098	24.99584	25.09867
gene-AT1G	0.721202	0.957679	0.761275	0.473606	1.534783	1.100828	0.756138	0.731629
gene-AT3G	3.579171	4.387857	4.399158	5.134341	4.923782	5.594696	0.936081	1.026717
gene-AT1G	0.247465	0.139201	0.128719	0.731743	1.005106	0.811443	0.160155	0.119364
gene-AT2G	8.284741	7.624221	9.193724	12.44541	11.85654	12.6248	6.359453	6.040504
gene-AT5G	23.74254	24.35166	24.11487	17.10679	18.75994	20.12478	34.22984	34.95719
gene-AT3G	74.67752	73.3909	78.75615	116.912	113.7935	120.1285	24.18978	22.10328
gene-AT2G	26.2021	25.02062	23.89344	24.24631	23.44024	23.09913	38.51901	39.96852
gene-AT1G	11.46654	11.66122	12.78552	23.10534	21.9652	25.08623	2.652047	2.265055
gene-AT5G	2.326301	1.727184	1.893361	1.450542	1.640977	1.505655	0.177039	0.311118
gene-AT4G	221.2522	216.0887	218.8891	130.8942	136.6023	134.0135	135.8389	134.6656
gene-AT2G	12.41105	13.06683	12.51778	24.62813	23.7837	23.6893	11.35845	11.55447
gene-AT5G	19.59365	18.36971	18.47621	13.59968	13.50673	13.85741	16.7292	17.98837
gene-AT4G	30.705	28.7297	29.07495	25.28783	23.7817	25.04257	24.53369	26.49471
gene-AT3G	26.65622	24.63456	27.74791	55.05987	56.00791	56.08974	11.56674	10.74298
gene-AT5G	16.46008	17.85539	16.77839	12.31356	13.6737	14.32315	10.99678	11.85646
gene-AT1G	6.560092	6.195933	6.772435	11.10023	10.51725	10.40479	15.39263	13.26349
gene-AT3G	5.836115	5.718344	5.741957	4.374107	4.708249	4.407032	2.483481	2.249168
gene-AT2G	6.867115	7.558598	5.743071	7.413185	9.133923	9.476798	8.406385	7.748215
gene-AT4G	2.638095	2.75264	3.172452	2.011609	2.688482	2.562567	3.12576	2.647313
gene-AT5G	2.667013	2.099708	2.738354	2.606997	2.110853	2.926364	2.909558	4.357356
gene-AT4G	25.63468	27.05225	19.5289	56.21655	77.60038	62.3054	11.31887	17.45619
gene-AT3G	6.595204	8.395597	7.359986	6.597771	8.454794	7.352163	12.39976	9.89828
gene-AT3G	18.57533	18.0504	16.89547	32.2787	32.54498	31.77315	23.01937	23.30806
gene-AT5G	7.925704	8.964309	7.949548	4.7805	4.258037	5.370856	12.3489	13.217

gene-AT4G	4.012262	4.430698	4.056123	6.692467	6.96276	6.146708	10.8714	11.20058
gene-AT2G	3.041799	2.980253	3.295085	2.145522	2.287809	2.364868	3.590047	4.184984
gene-AT3G	1.947586	2.109067	1.680276	1.156079	0.610801	0.639165	3.061154	3.502561
gene-AT3G	6.722224	6.981831	7.785026	31.35958	29.91884	31.68434	5.388951	5.330131
gene-AT5G	0.649184	0.711218	0.493109	0.460664	0.590555	0.404254	0.133237	0.093361
gene-AT4G	3.113123	2.423222	2.653005	2.276775	2.029663	2.720502	1.121773	1.238313
gene-AT1G	2.871751	3.504766	2.824358	1.636958	0.812432	0.859603	4.021294	4.082539
gene-AT2G	3.640651	3.512039	2.676924	3.842647	3.74898	3.838986	4.183378	3.758557
gene-AT4G	14.78947	13.67596	12.73626	5.991581	5.744775	5.762094	13.02612	13.42457
gene-AT1G	3.236338	3.702208	1.761252	1.021349	0.533161	1.097799	4.381565	5.12771
gene-AT1G	0.622175	1.019069	0.604303	0.290863	0.446795	0.516459	0.532189	0.839637
gene-AT1G	2.831797	2.292412	2.311508	1.813192	2.08148	2.570293	5.858571	5.527677
gene-AT5G	8.256047	8.293741	8.752466	5.497819	5.425395	5.321885	3.4629	2.964008
gene-AT1G	0.070359	0.570317	0.365056	0.048738	0.726353	0.91056	0.315268	0.483627
gene-AT1G	42.9895	45.19538	44.8196	26.44745	24.89351	27.26296	16.05998	16.30603
gene-AT5G	5.391464	4.937868	5.594079	7.155199	6.628714	6.270865	7.720567	6.70309
gene-AT4G	13.91473	13.78216	14.597	12.56654	11.15532	12.44829	19.57876	21.47069
gene-AT1G	1.471761	1.961995	1.912714	1.032868	1.01123	0.793572	0.296687	0.296264
gene-AT3G	42.81619	41.39799	42.28822	18.12153	16.61329	17.6072	39.19022	43.61132
gene-AT3G	60.93535	57.29219	59.4437	101.6543	96.38494	102.0611	16.34474	13.43485
gene-AT5G	25.26056	27.17899	29.44955	23.53606	23.84276	23.96958	10.85457	12.06237
gene-AT2G	1.418267	1.687486	2.566696	2.086755	2.131199	2.48757	2.748686	3.265865
gene-AT4G	23.3521	21.4602	22.82279	11.47617	8.696126	9.020021	23.30146	24.18568
gene-AT1G	2.771355	2.979089	3.285222	2.072704	1.848914	1.719091	4.94943	5.54668
gene-AT3G	3.504466	4.393812	5.116239	7.357883	6.358043	6.730171	2.643136	2.53052
gene-AT1G	0.518278	0.573035	0.435754	0.914399	1.320019	0.932943	1.015727	1.079267
gene-AT1G	7.127395	8.128267	7.632195	3.939154	3.723793	3.205761	12.13915	12.73617
gene-AT3G	0.909935	0.880748	0.644433	1.540163	1.558274	1.236593	0.408009	0.286474
gene-AT3G	33.85135	34.79917	37.73844	32.35659	32.88299	31.06123	25.21009	23.56825
gene-AT4G	7.303217	7.351005	7.537692	4.339826	3.924152	4.85751	2.535758	2.729052
gene-AT5G	4.252276	4.849641	5.669125	3.831354	3.136911	3.596424	3.880947	2.365412
gene-AT3G	4.549305	4.354509	3.734854	1.396174	1.157398	1.162541	4.674288	5.591
gene-AT5G	19.96906	20.18189	20.09908	19.27903	20.18572	20.52326	9.973923	10.52904
gene-AT5G	5.601378	7.206297	6.322175	13.73985	12.25592	12.36676	2.118395	2.791633
gene-AT3G	9.790036	11.80232	12.91803	8.449344	6.259309	7.616596	13.05994	15.74874
gene-AT5G	16.24313	16.44743	15.72627	20.99674	20.25102	20.29407	5.886994	5.98511
gene-AT5G	0.050859	0.022807	0.141997	0.152212	0.095225	0.068716	0.38525	0.509851
gene-AT4G	3.146294	3.36545	2.834075	3.379762	3.627007	3.255895	4.003933	4.819269
gene-AT1G	1.212253	1.706564	1.540113	1.680378	2.101526	1.712176	0.935042	0.51575
Arabidopsi	1.399703	1.506416	1.303088	0.172192	0.564189	0.717613	0.097693	0
gene-AT3G	18.37869	17.44356	16.9616	7.307126	9.247971	8.288943	21.50351	24.29322
gene-AT1G	21.57805	25.15951	47.70029	4.870177	6.892437	17.89191	83.7686	69.09708
gene-AT1G	893.8192	896.3174	922.7979	484.3582	477.7105	476.3204	1538.525	1550.597
gene-AT2G	6.342207	5.784056	5.591908	5.373302	3.442854	5.087108	1.267663	1.140617
gene-AT1G	0.326475	0.058246	0.227588	0.704449	1.537008	1.049276	0	0
gene-AT1G	1.092983	1.110745	1.732142	1.140216	1.082004	1.102812	3.493957	1.879529
gene-AT1G	4.702106	4.147306	5.050467	5.610297	6.01901	6.428871	1.770349	1.68959
gene-AT4G	18.70646	17.37942	20.45099	14.34322	12.28649	14.9993	23.68307	24.39306
gene-AT5G	2.170279	1.865769	1.853338	2.844803	3.510381	2.213081	3.495803	4.502989
gene-AT3G	1.713068	2.082199	1.775232	6.253724	6.128244	6.579699	2.222338	1.779004
gene-AT5G	67.67246	68.66984	69.98847	62.41076	65.39211	65.16674	48.72588	51.87011

gene-AT2G	0.303429	0	0.127285	1.865152	1.187392	1.898168	0.772296	0.653706
gene-AT4G	9.113698	10.3071	9.10587	6.134542	5.288344	6.4258	13.87821	12.6697
gene-AT2G	9.939531	9.459467	10.03501	7.220983	8.332801	7.790099	2.535666	2.204386
gene-AT5G	10.92195	9.770694	10.92729	8.677075	7.964658	9.056503	0.779677	0.814523
gene-AT1G	12.8685	8.599607	11.75412	6.619292	5.562778	6.343806	27.03176	29.1718
gene-AT3G	140.5869	136.194	129.0255	71.70898	72.06921	73.22749	179.1606	177.9403
gene-AT4G	4.94534	4.257928	4.580117	6.044114	7.69207	6.424069	4.030457	4.794202
gene-AT1G	41.53953	41.66038	42.04736	43.62458	41.07248	42.02159	35.12863	33.51628
gene-AT2G	44.47553	44.32349	48.22326	16.98175	16.42626	17.15256	36.56682	43.0402
gene-AT5G	0.450045	0.787997	0.883898	0.526229	0.448932	0.361153	0.920517	1.106179
gene-AT5G	1.950814	1.668696	1.833606	2.708393	2.380835	2.40426	0.656165	0.729268
gene-AT3G	6.637464	6.695431	7.729053	2.537957	3.242778	3.927635	3.462713	3.089769
gene-AT1G	0.302238	0.070456	0.414429	0.747616	1.038035	0.931573	0.033729	0.162708
gene-AT2G	5.583073	5.937237	5.600354	6.547697	6.070765	6.219846	2.314257	1.67189
gene-AT2G	9.143665	10.28937	8.598149	11.2526	9.70186	11.00315	8.405851	8.516188
gene-AT5G	230.0742	224.7412	226.8965	185.9691	183.8101	182.9411	211.5751	221.3732
gene-AT5G	9.586466	7.9913	8.575846	4.677283	4.784483	3.856203	9.214659	8.451165
gene-AT3G	5.219512	5.48822	6.397471	5.368934	7.187004	5.196653	2.252284	2.736101
gene-AT3G	7.632679	8.400259	10.06542	8.929121	10.33621	8.382887	16.51431	13.68736
gene-AT3G	57.53808	56.67075	55.90145	82.25615	80.67047	82.52252	25.09654	25.47619
gene-AT1G	5.955709	6.804707	7.859583	4.888164	4.665934	4.524444	3.954683	2.49831
gene-AT3G	1.814895	0.879306	0.856728	0.315415	0.609885	0.447909	1.858314	2.03165
gene-AT3G	18.78048	16.94368	19.03443	15.27804	12.28449	15.59802	26.42549	25.67496
gene-AT4G	4.925263	3.98635	4.86216	9.006109	9.648806	9.91332	3.927683	4.047478
gene-AT2G	4.849886	3.821468	5.014565	1.057648	1.241456	0.928513	0.085742	0.032889
gene-AT2G	13.03662	13.3997	13.98146	19.6202	19.41596	20.97757	8.505821	9.298319
gene-AT1G	1.205852	1.243367	0.933792	2.056353	1.928264	1.660176	1.128479	1.273588
gene-AT5G	27.41324	25.43615	27.70207	26.78927	27.59826	25.52385	26.12124	30.40721
gene-AT1G	0.010866	0	0	0.030066	0	0.028868	0.030656	0.015385
gene-AT4G	7.080225	8.906923	8.307815	6.664815	7.290513	6.892311	2.979824	2.783322
gene-AT5G	5.072066	4.888407	5.622946	11.09383	12.31007	10.21538	4.245451	4.501221
gene-AT2G	0.211115	0.334685	0.167856	0.05522	0.08997	0.028486	0.515059	0.611344
gene-AT3G	8.680419	8.351017	9.877261	6.797962	8.572848	6.969118	10.50928	9.926744
gene-AT1G	0.323689	0.592751	0.382091	2.464275	2.294873	2.291527	0.18208	0
gene-AT2G	19.84285	23.676	22.29475	27.51951	26.14338	26.67186	7.885571	7.351705
gene-AT5G	19.32797	18.99007	17.9383	5.758705	5.763538	5.739374	10.57415	10.35106
gene-AT4G	4.630406	5.350882	4.515065	3.576928	4.752261	4.309231	2.034776	2.213609
gene-AT3G	20.46312	21.52765	19.41562	19.04456	20.24766	20.39955	35.17656	39.27157
gene-AT3G	8.419194	7.852824	7.787086	6.533311	5.672878	5.214597	13.50387	14.2652
gene-AT3G	6.253475	7.286184	6.527555	4.546512	5.920445	4.761533	4.978808	5.335847
gene-AT4G	1.329447	1.007966	1.312591	0.405247	0.39795	0.209707	1.711193	1.306167
gene-AT4G	8.470805	8.521626	9.722278	4.03235	3.15024	3.197979	14.28913	14.13979
gene-AT3G	230.7535	221.1081	230.5011	110.8976	115.952	111.8777	113.7149	119.2429
gene-AT1G	0.497857	0.617976	0.482123	0.985541	0.69414	0.84387	1.063374	1.271303
gene-AT5G	0.669354	0.339107	0.558692	0.908281	0.710693	0.822784	1.114778	0.960876
gene-AT5G	2.870073	3.175964	2.864361	2.650717	2.145882	2.864805	0.953383	1.281079
gene-AT4G	1.154828	0.931593	0.798081	0.67092	0.667039	0.825793	0.244465	0.586659
gene-AT2G	7.988239	7.069348	6.464797	3.094729	3.621246	3.363495	2.161339	2.371766
gene-AT2G	0.985179	0.806813	0.911038	0.942996	0.649226	0.639698	1.990299	2.080786
gene-AT4G	11.28935	11.4108	9.87924	6.848691	5.455971	7.51003	17.56873	21.39022
gene-AT3G	9.730729	10.07861	9.762004	5.268681	5.341974	6.168488	3.715804	4.031691

gene-AT5G	60.06019	58.09776	57.52201	44.96443	45.49198	46.47325	87.00642	93.07435
gene-AT5G	0.855293	1.244985	1.495358	2.024465	1.214463	2.235657	1.703346	0.901728
gene-AT5G	10.25886	10.54976	9.248989	14.44212	15.60063	16.86535	9.260661	11.14916
gene-AT3G	2.060248	1.74024	1.967043	2.584322	2.394249	1.677545	1.198189	1.358663
gene-AT3G	2.557723	2.388291	2.228244	1.112866	1.734864	1.789369	3.030688	3.066414
gene-AT5G	5.905695	6.017686	5.844929	3.800451	4.120525	3.449687	9.297481	9.3719
gene-AT1G	3.963753	2.664405	3.297856	3.182508	3.68503	4.14886	3.867498	4.384546
gene-AT1G	0.829397	0.984492	0.928107	1.368252	2.152083	1.564926	0.494763	0.627431
gene-AT5G	23.38243	22.12743	23.75368	4.311756	4.728782	4.158962	38.66786	37.99085
gene-AT3G	3.112158	2.757987	2.259018	2.717404	2.970247	2.569467	1.069211	1.082477
Arabidopsi	1.62215	1.61114	1.691446	1.778499	2.458635	2.9039	0.749093	0.834693
Arabidopsi	5.806135	5.250912	6.149989	10.26506	9.691575	9.882351	5.292066	5.65033
gene-AT3G	0.572144	0.301727	0.79699	0.515285	1.167284	0.70611	0.471468	0.12016
gene-AT2G	0.133225	0.217084	0.214923	1.179151	1.27709	0.986417	0	0.241514
gene-AT1G	0.587593	0.643337	0.518159	0.868816	0.999655	0.794094	0.068037	0.270203
gene-AT1G	9.976843	11.58041	7.879023	4.563464	2.664367	5.036596	4.579472	3.238271
gene-AT5G	7.106634	6.923625	8.349385	3.319982	3.010106	3.381015	0.948786	1.570218
gene-AT3G	26.45076	28.62235	28.35837	22.66602	27.76326	26.82704	48.22272	49.72963
gene-AT5G	0.25	0.307081	0.351499	2.017289	2.17176	2.365742	0.316231	0.520082
gene-AT2G	2.659438	2.886214	2.280998	2.706669	1.86234	1.912559	1.413036	1.003125
gene-AT1G	1.653161	1.513439	1.180075	0.678325	0.998115	0.973173	2.066136	2.357007
gene-AT1G	19.66847	19.2548	20.83016	19.49779	22.58505	20.72454	38.75035	40.2629
gene-AT4G	0.99326	1.508549	0.898361	1.235233	1.256256	1.340473	0.332432	0.373013
Arabidopsi	1.198482	1.400608	1.632403	1.614327	1.959133	1.900223	0.013059	0.035754
gene-AT1G	4.276789	4.723098	5.351816	4.936907	4.204443	2.70282	7.335573	5.735768
gene-AT3G	3.65549	3.999074	4.432622	7.300351	8.358423	9.87918	6.091086	6.561021
gene-AT5G	5.203357	4.942996	5.678959	5.623516	5.763519	6.098945	2.068081	2.139299
gene-AT5G	0.624439	0.550661	0.781207	1.044125	1.120338	1.135834	0.224298	0.295194
gene-AT1G	3.69292	4.025308	3.611593	4.336097	4.342098	3.563707	5.912095	5.433056
gene-AT3G	3.416367	2.963558	3.619591	0.821792	1.191853	1.523821	6.619192	4.173298
gene-AT3G	47.18235	45.62425	45.08644	35.44228	36.19165	35.95499	70.96131	75.78523
gene-AT2G	143.4775	142.1434	152.1492	123.9489	120.3965	117.2844	244.0357	253.954
gene-AT3G	0.778847	0.60311	0.772349	0.740274	1.267215	0.586606	0.377693	0.594955
gene-AT5G	40.33961	40.21775	38.84617	26.44183	25.29532	23.69427	68.25656	73.67589
gene-AT5G	6.682024	5.262902	7.073419	11.31409	10.20339	9.051729	10.51017	9.378696
gene-AT1G	12.41709	13.95573	12.80022	14.51682	17.13451	15.60017	5.786841	6.422443
gene-AT5G	59.3938	57.61424	61.01345	41.95767	41.41425	39.50841	53.14864	49.7505
gene-AT1G	52.92508	55.62071	51.36324	57.09379	56.3908	59.605	59.0964	63.71401
gene-AT5G	0.95543	1.460051	1.388655	1.214739	0.438836	0.607176	1.147793	1.05721
gene-AT2G	319.0331	311.0764	321.2571	166.691	167.8423	161.5917	526.803	543.6436
gene-AT1G	26.02025	21.33278	24.49449	21.33903	19.32844	20.04371	13.53881	12.73397
gene-AT5G	23.94606	19.72234	23.02268	20.331	20.35865	22.96201	24.92753	24.33239
gene-AT4G	1.151168	0.716663	0.649453	0.447241	0.909784	0.38284	0.801046	0.826892
gene-AT3G	61.19015	64.99757	62.53321	61.7732	62.6005	60.68671	119.8839	111.3456
gene-AT1G	7.851128	8.195485	8.527533	41.51085	38.07591	41.53941	13.74334	12.46594
gene-AT1G	1.756923	1.779963	2.027727	1.891708	1.421983	1.858068	2.5576	2.264551
gene-AT3G	52.34697	53.92691	60.72158	43.5443	43.1227	41.84941	69.74837	71.75157
gene-AT3G	11.4681	10.42958	10.17176	6.973497	4.912467	6.03742	1.993058	1.772051
gene-AT4G	0.021085	0	0	1.617924	1.395588	2.260786	0	0.065741
gene-AT5G	2.508628	2.037711	3.000651	2.902657	1.728626	2.53334	4.219256	5.197149
gene-AT5G	9.517963	13.68697	11.41664	8.914211	9.203641	10.05857	14.03407	14.96661

gene-AT3G	0.098601	1.388878	0.966823	2.942206	2.030511	2.530453	1.311714	0.981578
gene-AT1G	0.128925	0.364331	0.4486	0.768633	1.136826	0.833765	0.379764	0.535143
gene-AT3G	4.053532	5.057834	4.736813	5.005955	4.531641	3.795743	1.973152	2.427448
gene-AT2G	97.94502	98.11959	100.4816	45.2642	45.35111	45.03466	62.64237	63.87751
gene-AT4G	25.28829	22.30873	25.93366	35.16254	34.22636	32.43021	12.14056	10.40647
gene-AT1G	39.45424	35.78476	36.61059	33.93428	32.66178	37.85157	63.18794	67.18588
gene-AT4G	6.925163	7.062219	7.966986	13.91893	14.40155	15.75907	4.368158	5.033632
gene-AT5G	67.29022	66.05528	67.08197	31.0641	29.74914	30.47422	239.1126	243.5584
gene-AT1G	2.22206	2.367773	2.112155	6.52242	5.330904	6.428613	1.6001	1.483722
gene-AT2G	13.56191	11.11704	12.93984	37.63677	34.41996	33.80497	16.39395	18.68091
gene-AT1G	6.350683	6.747904	6.285038	3.885818	4.21836	3.284676	11.67274	11.7387
gene-AT5G	4.300229	4.712241	5.119202	6.720973	6.184369	7.034829	5.691667	5.984081
gene-AT2G	156.6314	159.4465	160.12	122.4219	120.9955	126.9445	234.9061	247.971
gene-AT5G	144.1845	159.7053	166.3216	40.01225	38.54159	35.09351	135.9728	134.6331
gene-AT2G	7.531531	7.941289	7.655073	5.04344	5.217806	5.665682	5.164385	5.292733
gene-AT5G	12.99004	11.88274	12.79221	25.73406	26.69995	31.40638	30.71516	26.95271
gene-AT5G	0.217005	0.397198	0.174563	0.111156	0.239597	0.425486	0.456302	0.509341
gene-AT3G	47.77417	49.24943	48.30693	45.39344	45.3879	44.06385	55.05504	54.75806
gene-AT2G	20.22018	22.61917	19.38536	18.4869	19.45296	18.18217	7.690712	5.612789
gene-AT3G	41.86491	39.25936	42.15315	31.7021	33.37536	34.37157	60.53402	58.72155
gene-AT1G	0.706087	0.501186	0.697643	0.884969	0.690804	0.898046	0.536255	0.610289
gene-AT3G	7.854569	7.588481	7.589561	9.848154	9.432496	10.04472	10.44421	11.68818
gene-AT5G	15.68004	16.93498	15.17653	6.931963	7.48521	6.745403	13.31607	12.91398
gene-AT2G	7.068809	7.48362	7.688075	6.681754	6.740676	6.146976	12.94052	13.26404
gene-AT1G	169.7189	164.6758	169.4463	108.6306	108.3251	106.9069	160.6389	167.3982
gene-AT4G	1.07752	0.60015	1.106914	1.230693	1.200674	1.002728	0.612205	0.211584
gene-AT3G	2.977932	3.049402	2.446283	1.715352	2.25269	1.513439	1.840454	1.733498
gene-AT2G	2.131695	1.745799	2.40139	3.791787	3.314717	3.539398	2.071978	2.178088
gene-AT1G	7.293173	6.570457	7.226798	5.510419	5.933708	5.761294	2.061456	1.741454
gene-AT3G	3.183902	3.598808	3.497998	2.173	2.055448	2.429934	1.445807	1.414397
gene-AT3G	2.376815	2.314258	3.03181	2.257667	2.042785	1.785409	3.184186	2.730257
gene-AT2G	11.22719	11.02434	11.08503	32.22172	30.63985	30.94446	8.065171	8.918841
gene-AT2G	6.549009	5.253371	6.69464	10.00093	10.26718	10.14939	7.237518	7.411976
gene-AT4G	3.864012	2.946651	2.600913	4.629947	4.929338	4.55492	2.611849	3.013548
gene-AT1G	11.58608	12.79708	14.02089	14.99459	15.01038	14.8558	11.31183	10.27112
gene-AT5G	8.973419	8.072039	7.721951	5.707786	6.08441	4.595372	14.17439	14.43098
gene-AT5G	1.376301	1.547528	1.132825	3.501363	3.415689	2.496056	2.015944	1.825323
gene-AT3G	9.119854	10.02545	10.93113	13.40541	14.41877	13.36042	17.7147	15.91592
gene-AT1G	0.169523	0.28447	0	0.212205	0.381774	0.376158	0.287064	0.263166
gene-AT5G	0.386337	0.365691	0.274141	1.06082	1.132801	1.04103	0.867834	1.057715
gene-AT3G	160.8224	157.3116	162.0657	97.48376	95.23343	93.47067	226.0476	245.1247
Arabidopsi	0.281409	0.244053	0.328512	0.661457	0.38235	0.464411	0.351883	0.32531
gene-AT1G	14.12556	15.12701	15.2614	4.104669	5.066076	3.608071	9.323449	9.898174
gene-AT1G	16.16591	16.93388	16.95724	11.7354	10.74738	11.59014	14.94308	15.78872
gene-AT1G	41.6534	37.76576	40.21949	37.93519	35.25703	37.3173	32.44922	32.31636
gene-AT2G	119.5909	110.0371	116.5215	85.86156	89.73071	88.77117	196.7062	202.9198
gene-AT1G	5.353589	7.928957	6.53304	17.10146	16.2164	17.47394	4.997972	5.108033
gene-AT5G	0.839657	1.117655	1.007924	4.618219	4.359303	4.900955	0.534324	0.449886
gene-AT2G	3.159404	3.047224	3.345213	3.336099	3.011663	3.921283	1.363522	1.342138
gene-AT5G	2.06845	1.635715	3.09932	2.420126	2.728945	2.808704	4.7072	3.041495
gene-AT3G	9.13623	8.250082	8.419168	9.624036	9.121963	9.484062	12.39808	12.46406

gene-AT2G	12.55102	12.7309	11.7343	14.18868	14.74669	14.24373	3.532669	5.125851
gene-AT2G	20.87149	19.38175	22.24624	18.18437	18.67758	18.20776	27.19003	28.58022
gene-AT4G	0.766307	0.827475	0.793711	0.511499	0.435529	0.30039	1.027249	1.065459
gene-AT3G	9.835813	9.819056	9.330873	10.50421	11.22977	10.28485	3.52665	3.685696
gene-AT3G	4.719335	3.86613	4.54709	5.503216	5.353696	5.605842	2.912492	3.252778
gene-AT1G	0.791388	0.96771	0.976239	1.613822	1.388176	1.488841	0.563839	0.557476
gene-AT3G	262.5054	245.3798	241.3856	225.8493	229.724	213.9187	457.3905	485.6896
gene-AT5G	7.658209	8.033532	8.015385	15.60385	16.86783	14.18126	6.100278	5.245433
gene-AT3G	113.5153	97.95041	113.5237	53.92996	45.38266	42.63819	16.17608	14.36399
gene-AT3G	0.06155	0	0.05719	0.716735	0.639325	0.62116	0	0.081861
gene-AT4G	1.110339	1.174713	1.513551	1.760828	1.76301	1.385082	0.998986	1.192532
gene-AT3G	3.811161	4.076052	4.264723	5.223208	5.581198	5.202318	5.814192	6.223212
gene-AT5G	6.320192	5.788444	6.382693	8.957422	8.535647	8.551252	2.518892	3.882741
gene-AT1G	43.85691	41.10362	43.95076	16.84539	18.39704	15.66192	105.8527	109.1415
gene-AT5G	4.382199	3.38885	3.711192	6.018508	6.876523	5.061971	2.762747	3.802184
gene-AT5G	14.27853	15.75117	16.04442	10.51948	9.052373	9.442638	16.04295	16.98345
gene-AT5G	29.99629	28.43113	32.90672	42.82228	41.86101	43.01484	15.96846	15.82487
gene-AT4G	0.70917	0.659392	1.080599	2.446986	2.88527	2.866765	0.090707	0.187217
gene-AT4G	61.81436	61.13346	62.20989	45.18918	49.21345	46.64084	79.72952	80.77287
gene-AT5G	69.38446	66.55853	64.95529	57.50999	56.96378	59.15954	84.97816	90.37273
gene-AT5G	8.335743	7.750383	8.189694	4.924342	5.205787	5.087581	8.252791	8.095343
gene-AT3G	0.737984	0.429835	0.811525	1.443329	1.000932	1.039663	0.144054	0.359836
gene-AT5G	6.421296	6.391999	4.990338	7.151925	8.829621	8.136177	11.75639	14.43446
gene-AT5G	153.5692	155.1602	159.8389	292.8104	296.5269	287.2906	142.0722	151.9441
gene-AT4G	4.490443	4.022786	4.629922	5.208052	4.542405	5.260238	6.907361	7.169721
gene-AT5G	4.18746	4.409419	3.886872	71.64204	69.87016	66.78571	0.194977	0.234755
gene-AT4G	2.381579	3.299538	1.957358	2.482326	2.852151	2.792981	3.184249	3.639323
gene-AT1G	2.690403	2.712208	3.333799	2.596224	2.891794	2.348409	1.31378	1.141618
gene-AT5G	204.0476	198.3675	188.6646	125.1307	128.2683	123.5866	369.605	369.8045
gene-AT4G	2.18034	2.253022	1.892673	1.344516	2.003984	1.444032	2.475424	2.012559
gene-AT3G	23.24611	22.27305	20.81432	16.88502	16.04792	17.39448	32.55468	35.06678
gene-AT3G	0.546264	0.359285	0.504005	1.251248	0.44431	0.439783	0.4241	0.333656
gene-AT4G	10.03377	9.837114	11.00651	7.316778	5.73848	6.656811	3.306915	4.618854
gene-AT5G	77.57243	77.22727	79.08797	69.03257	61.95903	64.8368	134.5374	134.1134
gene-AT5G	4.266439	4.911592	5.346128	5.435239	5.288237	5.337095	6.513647	7.615877
gene-AT5G	4.684598	4.571018	4.806029	5.570158	6.885728	7.828035	4.890009	5.21123
gene-AT5G	9.381759	7.261325	8.343823	3.534416	4.101245	3.490289	19.48232	20.45121
gene-AT5G	41.21079	41.39355	39.46369	27.00895	31.02127	28.47395	46.49762	49.58206
gene-AT2G	11.43575	10.92736	11.08952	12.42237	13.38822	11.93069	17.39964	17.08761
gene-AT5G	13.9047	13.64145	13.86665	20.95069	18.72322	20.08641	8.870783	9.609345
gene-AT1G	0.465984	0.141433	0.897504	0.61401	0.720352	0.52469	1.437733	1.179398
gene-AT2G	1.184026	1.242494	1.064856	0.472736	1.137365	0.707424	1.543588	1.529177
gene-AT5G	9.98949	9.968845	9.414311	5.303988	4.925616	5.579002	2.868681	3.320321
gene-AT2G	0.188277	0.1744	0	0.219771	0.281715	0.248379	0.603333	0.228059
gene-AT3G	88.38028	86.54524	87.94827	100.2808	101.0624	99.84742	135.1782	139.0189
Arabidopsi	0.235629	0.303215	0.371548	0.728055	0.346349	0.6399	0.04784	0
gene-AT2G	129.1263	129.4462	128.703	441.0915	456.4331	454.8149	84.6523	89.84227
gene-AT2G	3.760336	4.132829	4.241612	3.545157	3.113547	2.852206	7.349882	8.545597
gene-AT2G	0.752604	0.427379	0.376064	0.355295	0.155267	0.522055	0	0
gene-AT2G	2.248287	1.846158	1.867386	2.95043	2.187392	2.701526	0	0.279138
gene-AT2G	12.12104	14.67096	14.41714	20.15681	20.87016	21.18428	9.421553	11.66538

gene-AT5G	1.074865	1.051102	0.991306	13.15971	14.83483	14.63646	0.710237	0.573922
gene-AT5G	38.04565	33.86436	39.24726	31.77602	31.81985	29.14881	49.75282	46.99477
gene-AT1G	0.767792	0.801286	0.499932	0.04712	0	0.067116	0.622794	0.721394
gene-AT1G	0.661982	0.672644	0.675998	0.036207	0.075768	0.040009	0.56438	0.780817
gene-AT5G	27.28478	29.36695	24.0788	15.69881	14.98123	14.4404	27.2312	29.70486
gene-AT1G	0.853504	0.799448	1.174776	0.870847	0.696279	0.728925	0.089153	0.107737
gene-AT1G	4.249868	4.640097	5.45224	7.625299	7.556797	7.589987	1.398251	1.424724
gene-AT3G	2.230108	2.875651	2.670353	1.057593	1.099003	0.5907	11.24091	12.02135
gene-AT4G	12.93511	11.60585	13.79747	11.37512	11.33953	10.74861	14.24008	13.70662
gene-AT1G	21.8287	19.42899	19.13488	13.4683	13.84716	13.27477	3.66353	4.205105
gene-AT5G	3.409072	4.368397	4.826219	1.742662	1.770684	2.091152	0.67312	0.723813
gene-AT1G	32.44882	31.12124	34.23993	27.21669	28.34401	28.28532	48.04476	51.07219
gene-AT1G	30.53727	29.22732	29.39166	19.76846	17.88006	20.06736	52.19638	56.44174
gene-AT2G	26.74099	27.57254	27.36917	40.47828	39.07771	38.29482	16.21131	16.72898
gene-AT5G	3.126329	2.262816	2.607061	4.32407	3.343042	3.601609	5.220044	4.354308
gene-AT1G	0.644489	0.686684	0.504315	0.635087	0.275961	0.64262	0.606712	0.278447
gene-AT1G	29.89934	30.27196	31.38894	21.0346	24.73133	24.11525	29.707	30.8742
gene-AT1G	1.763548	1.723803	1.000791	2.436404	1.686307	1.606557	1.774017	1.578003
gene-AT5G	18.46566	18.12848	18.32559	15.8274	15.72715	15.57393	29.96755	27.25369
gene-AT1G	6.009482	6.084607	6.303783	5.620996	4.255386	6.172173	1.494732	1.806581
gene-AT3G	9.553016	10.69603	11.51728	7.156961	6.487495	5.519311	10.44843	11.82449
gene-AT2G	5.04725	4.358522	4.500123	4.771985	4.752744	4.566573	3.976473	5.03618
gene-AT4G	92.47494	96.53723	96.06323	43.40598	42.88434	42.78987	49.97166	55.73548
gene-AT3G	0.559809	0.328637	0.532309	1.375627	1.527736	1.715908	0.344607	0.544746
gene-AT1G	63.02263	62.99824	63.56812	30.62925	35.01854	30.31486	149.7979	158.4793
gene-AT3G	0.190961	0.378954	0.140816	0.110706	0.519771	0.140488	1.042524	1.089711
gene-AT1G	78.87586	83.84956	77.4774	191.0663	197.4148	218.3276	86.87151	63.37895
gene-AT1G	74.13497	75.06185	74.18957	132.5238	133.0958	130.1434	43.73903	45.40359
gene-AT3G	15.50868	17.29421	16.78267	13.59097	14.56961	16.71247	9.201571	10.04713
gene-AT1G	2.375289	2.372774	2.629878	2.108102	1.524201	1.828609	1.177898	1.178532
gene-AT3G	12.9734	12.14837	12.74363	7.149828	6.387373	8.034219	3.992613	4.377031
gene-AT2G	1.178287	1.039694	1.236252	1.486177	1.453503	1.774238	0.509641	0.50856
gene-AT5G	14.12852	13.93272	14.1643	6.006891	6.138624	6.980396	7.644854	7.668496
gene-AT1G	1.45162	1.955313	1.749376	3.044583	3.884682	4.449139	1.884798	1.885517
gene-AT3G	2.447119	3.178936	3.529109	4.695131	4.494581	3.866882	5.497588	6.587827
gene-AT1G	13.60559	11.56881	13.15951	12.39452	13.63998	11.85328	1.737461	1.823138
gene-AT2G	63.43192	59.82412	64.3612	69.29596	70.9466	66.81332	87.15258	91.62279
gene-AT2G	15.47977	13.62425	14.11952	14.41084	12.61238	12.47307	6.878797	6.485353
gene-AT1G	3.65964	3.860323	3.8585	1.572162	1.170694	1.610271	0.509279	0.38176
gene-AT5G	3.827864	2.590414	3.052576	8.545911	9.883094	9.218322	0.592108	1.225627
gene-AT4G	81.19072	78.07729	77.511	163.6141	159.2847	158.1367	62.9036	62.6026
gene-AT5G	0.774965	0.841771	0.528867	0.571466	0.413796	0.366257	1.470152	1.493775
gene-AT2G	0.571113	0.638943	0.591332	0.69217	0.826029	0.69406	1.318948	1.545445
gene-AT5G	0.391646	0.368703	0.745998	1.780607	1.189423	2.243845	0.304279	0.876285
gene-AT1G	1.297557	0.564284	0.2683	1.116189	0.932288	1.008448	0.538646	0.548625
gene-AT1G	44.19887	41.72876	45.59218	60.72335	60.8494	61.08231	20.40183	19.9589
gene-AT5G	14.40528	13.75632	14.67951	38.18117	42.03864	38.44667	15.58403	15.65024
gene-AT2G	3.958604	4.830575	3.012871	4.365487	5.141513	6.762936	1.766978	2.438364
gene-AT2G	36.82448	37.47938	34.88747	27.90944	29.53928	27.78879	65.07132	73.73331
gene-AT1G	1.377427	1.169377	1.307054	1.326434	1.462201	1.423356	0.631601	0.466509
gene-AT5G	8.753743	8.793842	8.922344	5.432995	5.390934	5.848027	3.046282	2.494885

gene-AT1G	84.03326	80.40747	78.11348	70.70731	72.3965	69.78092	109.4131	116.9373
gene-AT2G	6.621714	6.789519	6.739376	16.95192	17.17127	16.5803	1.100549	1.057253
gene-AT3G	30.73159	29.68278	33.00167	119.6537	117.3691	118.0813	50.7987	51.19976
Arabidopsi	0.014717	0.033625	0.139813	1.966271	1.619704	1.666079	0.501632	0.192591
Arabidopsi	21.72406	30.39248	29.66898	39.04225	40.43876	48.59887	1044.689	436.4725
gene-AT2G	1.830897	1.450603	2.156163	1.477193	1.213993	1.139392	1.485618	2.478257
gene-AT3G	29.03625	26.53078	30.96589	48.48583	46.81666	48.71695	16.24529	14.89669
gene-AT5G	14.24796	15.29261	13.97014	19.86114	19.99233	21.27129	3.79527	4.173225
gene-AT5G	1.358978	1.543035	1.211071	1.209573	1.070711	1.264672	0.319838	0.49539
gene-AT4G	39.36105	36.6181	37.96895	23.90913	29.43472	27.57466	56.39216	58.97361
gene-AT5G	0.964481	0.76679	1.110059	0.371486	0.412958	0.295627	0.83275	0.723774
gene-AT1G	21.54051	19.23891	20.58825	9.585784	10.1145	9.928943	31.15204	32.33913
gene-AT2G	15.11295	15.7727	14.73717	5.107964	5.203915	5.747651	5.351045	5.143027
gene-AT1G	0.359938	0.334832	0.268556	0.351815	0.699053	0.429705	0.997303	0.6596
gene-AT3G	16.27821	15.98928	17.73605	8.673481	9.949047	8.946936	29.51062	28.84789
gene-AT5G	4.303823	3.949009	3.243094	4.320517	4.979145	5.189458	2.411536	2.451809
gene-AT2G	0.15106	0.141697	0.145129	0.339211	0.207362	0.206158	0.143446	0.119242
gene-AT3G	4.783698	5.257186	5.263124	0.373195	0.533101	0.722477	0.343391	0.667765
gene-AT5G	1.035661	1.52948	2.203664	1.90665	1.211059	1.370032	2.345445	2.321915
gene-AT4G	160.5751	164.8539	168.0627	133.519	137.5646	133.7507	106.2992	104.9897
gene-AT1G	9.438608	8.910025	10.75732	9.945207	9.010909	7.573483	3.279365	3.034858
gene-AT2G	21.66027	18.14256	20.3902	19.97049	18.90859	21.57754	25.61126	27.80578
gene-AT5G	0.27096	0.345395	0.111667	0.929686	1.200152	1.033974	0.192551	0.420143
gene-AT2G	30.71867	29.23586	28.53267	22.65705	25.22872	25.11	41.48698	45.48203
gene-AT3G	0.126742	0.213895	0.053236	0.609757	0.656033	0.496379	0.084283	0.159776
gene-AT1G	1.856925	1.345838	1.730805	1.434102	1.074026	1.498887	0.744669	0.594782
gene-AT3G	9.457758	8.744254	9.145138	5.313258	5.152111	4.897681	12.78118	13.7528
gene-AT1G	29.29104	30.49027	30.00791	18.03854	18.18326	16.66032	26.18235	28.28988
gene-AT4G	2.179316	1.745958	2.267141	1.868492	1.284638	1.991409	1.054479	0.674974
gene-AT1G	17.42953	15.53145	16.74065	16.31112	17.2331	16.66406	6.218987	6.390543
gene-AT3G	15.10899	14.33686	14.61522	9.792005	8.014173	6.997421	0.816282	1.762871
gene-AT4G	22.09775	23.81889	22.45842	4.363193	5.091276	5.269419	16.38261	16.53961
gene-AT1G	18.9974	14.73469	15.66944	17.73865	16.85508	17.29343	6.776943	6.287691
gene-AT1G	20.55353	19.609	21.64048	15.49909	15.37778	14.84821	30.73452	29.18773
gene-AT1G	2.739391	2.67116	2.962914	2.154074	2.636316	2.185301	3.454593	4.331189
gene-AT2G	24.21149	26.22818	27.51241	17.76886	17.2716	18.23528	31.09748	30.35062
gene-AT5G	21.94087	23.91708	23.46167	10.62534	8.986389	9.520556	47.10343	48.43615
gene-AT1G	12.42381	13.14019	13.95218	9.522991	8.905421	8.260535	18.20812	19.50005
gene-AT1G	18.68695	19.39532	18.22151	41.98974	42.36362	38.72419	10.07449	12.27647
gene-AT2G	2.49303	3.261821	2.248505	0.635146	0.524292	0.582876	13.4495	9.648785
gene-AT5G	1.908723	2.037381	1.659311	0.966716	1.138281	1.181386	0.687857	0.518416
gene-AT4G	0.498218	0.740616	0.455407	0.576237	1.074451	0.743604	1.929859	2.119912
gene-AT5G	3.764087	2.219819	3.128408	1.279223	1.295318	1.192596	7.560661	6.70905
gene-AT3G	8.275178	11.78614	8.520212	8.822973	10.60771	11.20369	3.464533	3.220567
gene-AT5G	28.43474	29.202	31.40847	18.68127	18.3379	17.90138	29.9658	31.02258
gene-AT4G	6.485075	7.873141	6.83079	6.016547	5.758019	6.651112	2.564211	2.789398
gene-AT4G	14.6957	14.05391	14.66396	16.66201	17.3499	17.44425	13.52292	13.11353
gene-AT3G	81.70292	78.50166	80.16824	48.26725	49.78952	46.8712	153.2573	137.6442
gene-AT4G	4.681745	4.665134	4.158191	4.388668	3.787863	3.791296	2.615768	2.652667
gene-AT1G	23.89683	25.04731	26.91568	22.87124	21.30165	20.26188	24.83131	25.21243
gene-AT5G	134.6605	128.2259	135.526	124.6294	129.4037	127.4437	58.06727	63.74469

gene-AT3G	2.265489	2.944311	2.352115	26.88159	24.44259	25.38209	2.076131	2.263015
gene-AT5G	6.589466	5.23985	5.415485	6.016622	5.378944	6.575787	10.80825	11.12833
gene-AT5G	0.670432	0.751136	0.297966	0.083369	0.270025	0.318687	1.580091	1.081461
gene-AT4G	11.37013	10.48432	10.65667	8.284024	9.083714	9.686168	1.875716	2.299416
gene-AT2G	19.28021	16.09551	17.53611	28.24549	25.20362	25.46472	14.43638	15.06596
gene-AT2G	4.657257	3.761731	4.213537	1.399975	2.252357	1.515952	4.489209	3.788018
gene-AT4G	14.146	10.81927	9.670003	9.167398	8.587161	8.510455	13.50956	13.26976
gene-AT4G	16.06678	15.88058	17.45563	17.48654	17.46321	19.05255	6.024008	5.946055
gene-AT2G	35.03596	35.24049	39.07093	50.93728	46.3834	46.47455	16.5354	18.15392
gene-AT4G	48.98902	47.46475	48.18953	45.15601	44.587	44.94818	17.06331	18.11108
gene-AT5G	19.27961	21.03482	18.4709	12.81534	13.44517	11.39585	64.27028	59.28513
gene-AT5G	2.049658	2.296863	1.959744	1.851233	2.311754	2.193763	2.307229	2.447314
gene-AT2G	10.92662	11.59834	11.22947	1.889125	3.291902	1.659841	18.31508	17.83719
gene-AT3G	22.52125	21.45332	20.82155	39.53606	39.82944	40.02963	14.47966	14.68683
gene-AT2G	44.90479	49.43408	44.62519	20.69098	19.40984	19.28333	14.61664	15.90469
gene-AT2G	12.4107	14.03269	11.8181	16.02718	18.11714	16.59059	2.412384	1.918605
gene-AT1G	1.048465	0.642543	0.575591	0.885744	1.411525	0.68074	0.624147	0.4069
gene-AT1G	24.8247	26.54401	25.97748	42.37083	39.82062	43.39953	15.068	14.61156
gene-AT1G	2.310642	1.932392	1.397399	0.762977	0.660627	1.013467	0.724069	1.487541
gene-AT2G	3.23552	3.197039	2.628947	4.929912	5.079859	3.992283	0.88147	1.529194
gene-AT3G	22.31614	20.15868	19.90655	21.42918	21.75175	19.57452	8.649098	7.805974
gene-AT3G	6.565254	6.313624	5.388688	5.262277	4.616473	5.565239	9.151206	11.46999
gene-AT1G	97.96365	102.4884	102.6139	63.94379	64.65856	66.32794	44.76534	45.50189
gene-AT1G	4.726694	4.514722	4.996632	4.89495	5.206197	4.65319	6.875194	7.759729
gene-AT4G	6.984163	7.696763	7.755931	4.253234	4.453235	3.965854	0.639395	0.936348
gene-AT1G	7.008492	7.915453	7.37254	4.053385	5.303416	3.776737	1.291579	1.494327
gene-AT1G	7.997677	7.901701	8.652796	1.873413	3.043241	1.863453	7.395092	8.243022
gene-AT2G	3.411715	3.764273	3.256643	6.034633	6.479447	7.51828	2.393779	3.042415
gene-AT5G	227.8426	227.9101	228.6399	88.69582	89.12647	88.5128	32.9299	34.71314
gene-AT1G	4.510909	5.215812	6.074357	6.720931	5.240088	7.457435	5.30674	6.806537
gene-AT4G	8.347305	8.302976	8.128995	7.015098	6.05302	6.660069	26.08658	24.29072
gene-AT1G	28.90997	24.38377	26.26149	29.15663	28.52193	26.9085	26.36227	28.92193
gene-AT1G	8.622958	7.43314	8.29093	23.36593	21.6029	22.12561	9.731037	8.043273
gene-AT1G	0.199013	0.282174	0.582337	0.165264	0.402327	0.289349	0.223573	0.186351
gene-AT3G	5.477544	6.085099	5.302805	5.346809	5.334524	4.750704	2.475335	2.056251
gene-AT3G	2.583545	3.029348	2.90467	21.39364	20.06891	20.94611	5.527944	5.207139
gene-AT1G	1.658109	1.312958	1.803774	0.669653	0.949399	0.670006	0.378123	0.382996
gene-AT4G	6.165088	6.416362	6.476067	18.3543	18.35307	16.17045	10.21002	12.26988
gene-AT5G	1.75213	1.898629	1.673381	1.360889	0.571934	0.586468	0.550833	0.485999
gene-AT2G	46.81733	46.85794	43.18894	13.8697	12.56873	15.02655	27.60121	30.54776
gene-AT3G	0	0	0	1.256937	1.75003	1.36532	0.777811	0.47069
gene-AT3G	3.329137	2.784313	2.594548	1.307579	1.276847	0.829818	1.943709	2.249405
gene-AT1G	20.24325	19.80528	21.85569	19.4943	19.80693	19.71596	18.38784	19.75732
gene-AT5G	7.043722	7.350916	7.712902	14.4043	15.80999	14.1888	3.099063	2.278659
gene-AT2G	0.384253	0	0	0.492084	0.440222	0.212479	0	0.048685
gene-AT5G	68.85509	65.92323	67.74635	15.02027	15.98363	16.22482	12.94426	16.28218
gene-AT3G	31.61212	31.15428	33.35951	62.23104	63.95883	63.82496	32.20838	36.80209
gene-AT1G	0.409448	0.476294	0.31588	1.689481	0.851687	1.291068	0.570851	0.528295
gene-AT4G	1.236035	1.294509	0.987651	4.187447	3.310302	3.52087	2.003567	2.483101
gene-AT3G	2.344692	3.295375	2.613726	4.277285	3.61498	4.248058	0.66782	1.079161
gene-AT5G	3.379596	2.927358	3.528681	9.606265	9.018319	8.652333	3.194377	2.867097

gene-AT5G	23.15685	19.50804	22.27757	23.55981	23.64	23.7844	20.66057	18.72795
gene-AT4G	19.40846	18.53305	19.03734	8.0055	7.894943	8.254216	6.419947	7.869248
gene-AT3G	27.66721	27.22244	30.68784	27.68757	24.85904	22.47697	14.55796	19.29599
gene-AT1G	6.219999	6.619647	5.969872	8.995549	9.675104	9.297701	4.728	4.455769
gene-AT5G	0.979578	0.652209	0.831647	0.399729	0.95454	0.677577	1.554869	1.810334
gene-AT1G	11.44162	10.5163	10.4725	12.06811	12.63849	12.85715	7.28792	8.067949
gene-AT5G	13.53005	12.36445	12.34857	10.4031	10.70414	10.4781	1.72355	1.550705
gene-AT5G	6.945761	5.83286	5.98875	4.575908	4.823384	4.617195	2.033537	2.320152
gene-AT1G	0.367072	0.376266	0.524913	0.916596	0.416939	0.979086	1.216625	1.564024
gene-AT5G	26.96726	23.21445	28.54408	17.71179	18.48883	17.14378	40.89827	39.08991
gene-AT1G	52.75147	46.6281	50.05298	8.785285	10.25007	9.888741	5.463468	6.513276
gene-AT2G	5.65734	4.776315	4.92478	1.742713	2.124193	1.876268	1.72141	1.553295
gene-AT2G	9.216772	6.04836	9.21596	10.47524	11.43796	12.11384	4.761885	5.446683
gene-AT5G	27.18258	26.11678	27.48185	21.76499	21.30577	22.54574	38.57632	41.37971
gene-AT3G	1.331416	1.226198	1.166828	0.144425	0.271566	0.729511	2.050817	2.670533
gene-AT2G	1.228446	2.41304	1.432853	0.907185	0.610662	0.531852	3.961995	3.746333
gene-AT3G	16.15488	17.94823	16.66928	14.64804	14.76794	15.76464	8.985302	9.565912
gene-AT1G	2.674421	1.695342	2.192513	1.746973	2.252254	1.684246	3.144212	2.394578
gene-AT5G	3.080349	2.419528	2.83825	4.681654	4.663746	4.993911	2.070479	1.693813
gene-AT4G	22.64973	20.02205	21.51751	38.19815	36.38015	40.35057	15.21787	15.1278
gene-AT4G	1.29621	1.476783	0.920734	1.008906	0.70814	0.788807	2.694924	3.182244
gene-AT1G	116.9754	113.6957	113.7306	69.13826	72.5744	68.60656	214.6828	219.9505
gene-AT2G	1.12786	1.178251	1.165832	2.245951	2.094708	2.0051	1.043725	1.23984
gene-AT3G	2.227827	1.493897	1.462972	0.225426	0.173951	0.455365	0.732693	1.33542
gene-AT4G	1320.727	1348.286	1343.161	883.5767	864.8965	874.0438	523.6861	499.3153
gene-AT1G	1.806481	1.708268	1.372773	0.854424	0.737978	1.206947	1.489747	1.938452
gene-AT5G	5.220347	4.788924	6.053922	13.66758	12.85693	11.97	8.43502	9.157169
gene-AT1G	1.871335	1.763023	1.70972	2.228814	2.804753	3.181986	0.621665	1.267604
gene-AT4G	31.85672	30.91309	32.27905	29.19765	26.8192	28.67854	12.95937	13.05703
gene-AT3G	29.07757	30.20368	29.14564	21.74399	23.86956	20.99748	39.90969	41.50913
gene-AT5G	6.517049	7.66016	7.76287	10.45828	10.9367	11.32551	4.07376	4.085114
gene-AT2G	25.46017	24.68102	22.98395	24.54603	26.27124	23.8964	20.7977	22.48644
gene-AT5G	0.779859	0.606929	0.711772	1.944682	2.076788	1.547608	0.383946	0.390535
gene-AT1G	0.903786	0.753796	0.500878	2.3905	2.231973	2.105066	1.882934	0.89209
gene-AT5G	2.704441	2.097461	2.496715	3.394289	3.326798	2.544321	5.430758	5.022889
gene-AT4G	1.101336	1.339359	1.147374	1.108236	1.659715	1.141754	1.052103	1.585376
gene-AT1G	32.84257	30.64391	100.7118	27.67006	21.5833	36.06304	59.76522	38.7511
gene-AT5G	5.490094	5.336962	4.517494	3.539957	4.447984	3.988953	7.681769	8.433113
gene-AT3G	5.53817	6.542572	6.580337	7.180375	8.754794	6.648787	6.874162	7.602476
gene-AT5G	2.029471	2.518468	3.17358	0.375026	0.451201	0.144412	2.078589	2.849089
gene-AT1G	1.166607	1.105667	0.730363	0.864438	1.302336	0.436272	2.357904	2.475289
gene-AT1G	73.51283	69.78223	71.60851	36.68304	34.36496	37.28668	8.543853	7.499221
gene-AT4G	28.20155	28.54026	26.03911	14.52167	15.42965	15.17518	26.90598	24.56735
gene-AT2G	13.49508	13.04466	13.52457	32.63261	30.63009	30.52683	14.55861	15.81524
gene-AT2G	18.81481	18.97624	17.6213	15.32308	13.35909	15.19136	30.39005	31.85023
gene-AT5G	0.711577	0.987128	1.345763	1.837309	2.610209	2.695604	1.203902	0.920121
gene-AT5G	9.673854	8.05154	8.663379	5.315919	5.196069	5.372663	13.69849	13.51658
gene-AT2G	5.695871	6.2538	5.613875	2.790693	3.39267	2.6785	4.098647	4.209193
gene-AT4G	2.095129	1.974319	1.83695	1.969778	2.19362	1.342764	2.739603	3.168653
gene-AT1G	12.67774	11.76016	10.9989	6.758378	7.678	6.180687	10.5738	10.79639
gene-AT3G	1.454925	1.315521	1.30542	1.326648	1.312238	1.437619	2.305371	2.994938

gene-AT2G	157.2133	148.7789	147.5636	93.90923	97.65123	93.5719	279.3561	293.0796
gene-AT5G	130.2018	131.1711	129.406	250.5801	255.6185	248.7395	75.69669	76.30424
gene-AT1G	27.16101	24.09566	27.36025	28.91734	27.21777	29.01509	8.482755	8.945295
gene-AT3G	5.305909	4.174124	4.661034	6.009282	6.560764	6.510957	1.935346	2.389486
gene-AT4G	11.67222	10.35705	11.26087	11.59011	9.787208	11.76903	17.18026	17.00545
gene-AT5G	203.1943	200.8644	200.2847	57.37441	56.33858	55.35729	399.3639	392.9538
gene-AT5G	9.0749	8.660462	9.172421	10.3041	10.55849	9.728361	12.29978	13.25348
gene-AT2G	4.409939	6.121681	8.620055	10.83773	10.57458	8.739742	0	1.327305
gene-AT1G	53.96445	58.40583	52.24819	39.32241	43.38703	38.56045	67.5574	69.2247
gene-AT3G	542.7202	542.7064	547.5013	471.0257	468.2191	469.3896	194.3317	209.8414
gene-AT4G	4.758426	4.179798	3.72459	0.541623	0.66996	0.725395	0.26363	0.576657
gene-AT1G	30.64911	30.17978	30.50625	39.33887	39.68195	39.52031	13.49413	13.88871
gene-AT3G	438.1726	455.2553	447.9	275.796	264.9125	214.7768	910.9262	687.2453
gene-AT4G	4.243495	3.443746	4.403225	4.912798	3.898943	4.630031	2.184804	2.713655
gene-AT3G	43.18624	41.7458	42.7122	28.80477	28.60319	29.46062	13.28189	12.04161
gene-AT3G	1.763968	2.588914	2.337658	6.808689	6.342554	6.90406	1.383787	1.264983
gene-AT3G	1.875565	2.634893	1.934623	1.2816	1.646097	1.573901	2.87903	2.94411
gene-AT3G	4.52865	4.543634	4.175131	9.331415	9.464767	9.316366	2.754941	2.650869
gene-AT1G	37.07464	37.34796	37.41253	21.39303	22.63131	21.65832	14.77017	14.99752
gene-AT5G	8.558405	9.448853	7.571453	7.213728	7.27769	7.03354	3.50246	3.873407
gene-AT5G	1086.561	1069.582	1094.991	622.373	617.9731	623.0388	1669.905	1653.34
gene-AT3G	17.88497	18.86289	19.47966	11.02166	11.28017	10.88313	27.53635	26.84106
gene-AT4G	59.96769	56.73229	59.54502	9.07115	7.907019	7.023816	10.07392	9.147984
gene-AT1G	1.957991	1.970249	2.389467	2.3799	2.252867	1.963246	2.384479	3.023722
gene-AT1G	10.54356	9.367988	7.92924	4.918773	6.261713	5.739174	2.818834	2.098839
gene-AT1G	5.791687	4.662302	5.049546	6.362273	5.925702	5.887983	11.32109	9.566645
gene-AT1G	19.57367	21.60052	20.18581	15.46875	14.97093	15.54498	24.87512	25.80955
gene-AT5G	4.915575	4.471628	4.269873	2.425049	2.559865	2.831733	5.060803	5.165038
gene-AT5G	3.070591	2.662539	2.66108	4.199877	4.185733	4.136442	2.004795	2.309951
gene-AT3G	0.085687	0.112923	0.036427	0.206353	0.179121	0.054122	0.11904	0
gene-AT1G	14.18802	13.31746	14.44493	19.22685	20.97569	20.46535	11.58365	12.09439
gene-AT3G	3.377855	4.05801	3.78479	4.382645	5.092883	5.336068	6.893447	8.506915
gene-AT3G	5.0913	6.097374	5.966153	5.713484	5.721749	6.21091	17.28732	18.45865
gene-AT4G	2.645319	2.771226	2.280028	18.25153	18.45503	16.6338	1.117031	1.412508
gene-AT2G	7.028141	5.967798	6.897369	4.444478	4.522001	3.968834	7.900598	8.780717
gene-AT5G	6.808142	7.582566	7.526385	5.364176	5.52056	4.987416	3.403669	3.437874
gene-AT4G	5.826038	6.140105	8.996495	3.579744	2.681348	2.991424	1.746952	1.4633
gene-AT4G	1.237248	1.506246	1.373123	1.872026	1.276836	1.362121	0.468925	0.312203
gene-AT3G	0.961196	0.986391	0.871422	1.25408	0.857424	1.074531	0.424794	0.474218
gene-AT4G	0.550007	0.522497	0.694752	0.864838	0.986156	0.830523	2.546316	3.308249
gene-AT1G	6.126313	6.06558	6.208147	3.616395	3.32641	3.741025	6.151591	5.740012
gene-AT3G	6.161203	4.94336	6.159862	6.122993	5.937699	6.057327	6.341884	6.346823
gene-AT1G	48.23455	45.87579	42.09423	46.29518	44.91098	47.6222	20.69673	20.03013
gene-AT1G	0.265288	0	0.4055	2.493335	1.696247	1.595415	2.6387	2.909652
gene-AT2G	3.226396	2.666185	3.553709	5.785142	5.425257	6.006624	1.584334	1.683414
gene-AT3G	23.75414	24.39572	23.68798	19.03564	17.95201	18.4127	7.014448	7.300025
gene-AT1G	0.313304	0.501741	0.71596	1.546192	2.118519	0.96483	1.153973	1.307512
gene-AT1G	0.507215	0.777283	0.879851	0.361557	0.178013	0.496054	1.70665	1.892795
gene-AT5G	87.09399	82.92255	81.90299	71.07468	70.27119	69.26645	49.19588	52.04203
gene-AT3G	2.708621	3.485792	2.406877	4.228577	4.305235	3.761482	2.49358	2.727709
gene-AT5G	4.080454	2.761684	3.190687	7.187894	8.152166	6.646646	1.923639	2.678929

gene-AT2G	24.1204	22.58264	22.39472	53.86554	51.48333	55.08862	17.76522	15.28386
gene-AT1G	2.393501	2.766554	1.937592	4.916764	4.709265	4.905707	2.665841	3.328613
gene-AT2G	7.086812	7.565368	6.781573	14.12391	13.49578	13.57961	4.44742	3.642011
gene-AT1G	36.96914	39.39341	38.65323	28.8748	28.30631	27.69055	54.36743	56.83028
gene-AT5G	8.511628	8.768439	9.434788	9.479974	9.406713	8.437562	5.024626	4.946486
gene-AT5G	6.210257	6.180522	5.511147	4.056509	4.866839	4.594153	0.965935	1.503452
gene-AT1G	17.55156	16.09178	18.64077	14.82856	14.39432	15.21564	22.83342	21.86578
gene-AT2G	29.78074	29.11088	25.36394	26.40319	24.71479	25.20549	49.41032	49.6496
gene-AT2G	15.92632	17.29891	18.20413	13.53099	14.05325	13.21012	22.81298	23.16545
gene-AT2G	33.3457	29.83934	29.89291	31.3821	30.48208	28.72457	48.37564	53.28932
gene-AT1G	22.05949	19.257	20.87072	15.54675	16.28783	16.79876	22.9708	23.32714
gene-AT2G	28.88069	25.8146	26.29824	27.31273	25.37916	25.47294	42.22393	39.22262
gene-AT2G	9.124791	8.844543	6.402175	5.010739	5.619251	6.683166	2.643401	2.740195
gene-AT5G	2.230461	1.912167	2.382741	4.034417	4.439853	3.153503	1.521304	1.334848
gene-AT5G	26.49145	23.41029	28.21469	11.47786	11.42124	11.90786	11.8688	11.69877
gene-AT5G	10.51937	10.66403	11.19442	8.523599	9.309064	9.565464	4.823149	3.924192
gene-AT4G	49.5837	51.20356	50.2453	105.9919	103.6052	107.2649	53.27896	54.61234
gene-AT4G	1.387634	1.16516	1.402622	0.771815	0.850149	0.924115	0.35293	0.493867
gene-AT1G	1.433749	1.592263	1.549101	1.126548	1.258627	1.623878	1.504067	1.224331
gene-AT3G	13.4475	13.56139	14.84839	4.23207	4.649777	3.810417	7.243084	7.515595
gene-AT5G	169.9446	161.3116	164.6431	91.39605	84.17496	88.09288	19.6398	22.42145
gene-AT3G	0.17375	0.128012	0.11468	0.314711	0.307799	0.418104	0.176756	0.02289
gene-AT5G	4.149031	4.248735	4.72701	4.918934	4.751567	5.376129	7.003943	5.525869
gene-AT4G	4.150384	4.224971	3.847608	2.21806	1.676702	2.225841	1.179168	1.243963
gene-AT1G	1.315998	1.263414	0.999094	0.805403	1.246104	0.487732	1.019973	2.216414
gene-AT1G	2.583013	2.683973	2.462007	4.295278	5.698705	5.905783	1.575941	0.93124
gene-AT5G	4.513699	6.511581	6.179865	2.24854	2.293868	4.574005	5.578989	6.405691
gene-AT5G	0.156786	0	0.058272	0	0.113589	0.155834	2.08485	1.94383
gene-AT5G	2.291005	2.953017	3.128835	3.335116	3.053761	4.134049	1.913453	1.623519
gene-AT1G	3.941319	3.847465	3.6768	3.140358	2.922245	2.86481	4.520731	4.399299
gene-AT4G	7.222284	6.403829	6.238893	4.868568	5.13693	5.411776	7.8318	8.115953
gene-AT1G	2.082347	1.98476	2.171383	0.484154	0.433547	0.518492	3.95069	4.892329
gene-AT5G	19.36127	19.03538	18.22134	18.24918	17.60244	18.54071	28.64608	30.46461
gene-AT1G	2.218188	2.993061	2.306053	3.149839	3.188518	2.695443	3.036636	4.398446
gene-AT1G	2.54965	2.046782	2.016051	0.887105	0.866197	1.046639	4.590028	5.452463
gene-AT4G	40.40857	41.21033	36.84863	37.21348	37.91802	33.69361	51.23523	53.25377
gene-AT3G	28.1647	27.52771	28.32501	21.78598	22.62919	23.83329	53.07207	52.52927
gene-AT5G	27.86046	26.87585	25.73661	5.775248	7.406322	6.258501	6.409438	7.801148
gene-AT5G	3.48216	2.431982	2.949748	2.23402	1.856103	2.226745	0.472002	0.524643
gene-AT2G	81.51353	75.29644	75.63023	89.21133	95.86597	93.82776	33.72453	35.76885
gene-AT4G	3.866886	4.082703	3.616763	10.55441	9.351872	8.511484	4.454517	4.070743
gene-AT1G	1.405901	1.239844	2.323709	6.552431	7.079302	5.978359	2.457344	2.200292
gene-AT5G	0.583331	0.450978	0.842513	1.384496	1.216077	1.221029	0.769911	0.830437
gene-AT2G	1.165202	1.349067	1.272839	1.35936	1.32495	1.273868	0.427107	0.552133
gene-AT3G	16.05569	11.65982	12.55487	6.150136	7.209036	6.606644	22.51497	23.08231
gene-AT5G	0.398972	0.392014	0.353992	0.103273	0.208704	0.174093	1.290239	1.236209
gene-AT3G	271.4904	273.3346	279.7803	266.0896	259.8307	263.0777	435.7926	471.2359
gene-AT5G	0.596103	0.877251	0.855304	0.922774	1.309027	1.07337	0.128645	0.185205
gene-AT4G	11.03239	12.76489	11.62477	5.683392	6.401732	5.628796	9.990671	10.36155
gene-AT4G	0.953781	0.858146	1.060931	0.696295	0.953104	0.958558	0.191806	0.14921
gene-AT3G	5.425367	4.489201	5.312419	4.87552	6.118714	5.08686	8.097909	8.424091

gene-AT1G	25.91271	25.52527	23.56003	23.81366	20.5157	23.52535	30.3297	29.49696
gene-AT3G	22.06091	20.18474	21.59928	16.38727	18.82385	18.64064	28.54076	31.09729
gene-AT5G	1.100439	0.530776	0.631582	1.005776	1.352406	1.604609	0.855661	0.871575
gene-AT5G	2.019997	2.184471	2.256846	2.340775	1.994681	2.129018	2.482742	2.000228
gene-AT1G	10.04019	10.36119	11.24676	6.741922	6.514601	7.382143	15.28499	15.7266
gene-AT5G	4.566337	4.172319	3.877241	2.8624	2.263632	3.170236	4.648602	3.68876
gene-AT4G	37.34029	34.34498	33.6028	18.5799	17.52028	22.92353	49.94499	43.92624
gene-AT3G	75.68749	69.50127	74.60075	52.96663	49.60605	49.70152	115.9472	123.3152
gene-AT3G	2.887845	2.033713	3.277254	1.984344	1.741551	1.677614	1.842274	2.148508
gene-AT1G	1.517419	1.46749	1.479033	1.985736	2.222411	2.764483	1.073715	0.565457
gene-AT4G	39.69376	41.77177	43.0161	30.48803	30.02007	30.04533	40.06543	43.59921
Arabidopsi	1.336154	1.447594	1.289892	2.005321	1.202841	1.512122	6.332586	3.784845
gene-AT5G	3.333099	2.825549	3.504381	3.037242	2.478397	2.907307	1.359203	1.108885
gene-AT4G	1.669841	1.250504	1.247149	2.133656	2.148379	2.512908	0.478497	1.196068
gene-AT1G	4.06713	3.562284	4.037601	5.141282	5.276002	5.414968	4.445852	5.741622
gene-AT4G	10.66974	9.52044	9.242363	7.6325	7.471191	7.827903	3.995113	4.595504
gene-AT3G	3.359386	3.084254	2.673212	6.681102	6.35568	5.867712	5.771575	6.157788
gene-AT5G	0.198967	0.347255	0.43274	0.254849	0.74548	0.398686	1.128828	1.355553
gene-AT2G	11.44641	11.35926	10.03339	14.75367	12.38951	12.98935	3.650398	2.837652
gene-AT1G	1.14147	1.554378	1.281495	0.815777	0.66995	0.444974	0.525277	0.277922
gene-AT1G	2.55722	2.210587	1.973541	0.486711	0.696651	0.570801	0.293101	0.040617
gene-AT1G	5.232623	5.179479	5.024225	2.409132	3.096233	4.302057	5.255273	4.10178
gene-AT2G	4.086774	3.821837	3.469736	10.74042	9.84362	10.34146	1.858256	1.911601
gene-AT2G	6.892762	8.969715	7.611596	11.99842	11.64573	12.84266	5.43482	6.540838
gene-AT4G	1.345447	1.463076	1.359884	0.919659	0.452361	0.640292	0.296557	0.174461
gene-AT1G	18.29465	18.67695	19.39535	20.27075	22.25654	21.53485	0.514711	0.667392
gene-AT2G	10.2415	9.115915	10.88231	23.09836	23.61826	23.64839	4.355078	4.472019
gene-AT1G	1.206769	1.413816	1.500956	0.20758	0.389136	0.179379	4.409412	3.84898
gene-AT1G	6.021605	6.478492	7.18443	10.49806	10.84127	9.92267	2.600668	3.875547
gene-AT4G	0	0.138572	0.177352	0.529012	0.530154	0.747935	0.099367	0.106176
gene-AT2G	0.842853	0.985326	1.164549	1.361791	1.260513	1.683127	0.993926	0.942365
gene-AT1G	1.756146	1.404288	1.795217	2.264506	1.99451	2.042995	0.612833	0.661841
gene-AT5G	19.3384	15.88629	15.94264	18.60498	18.96743	17.27967	33.50475	37.37009
gene-AT4G	46.24427	48.13897	49.39717	34.42383	34.55866	34.57582	64.90112	69.59827
gene-AT5G	3.233993	2.927433	3.276603	4.468651	4.855476	5.440723	7.597625	6.021692
gene-AT3G	27.3198	27.22273	28.15583	24.46733	23.38399	24.82275	24.7686	24.30129
gene-AT1G	17.43726	15.94553	18.36487	16.30075	14.61775	17.06833	29.66633	28.68163
gene-AT1G	21.15129	22.53157	21.44133	12.55769	11.65069	12.54881	18.41406	17.4479
gene-AT5G	0.412993	0	0.163016	0.102129	0.026262	0.286144	0.144692	0
gene-AT3G	9.205	10.06401	8.825632	5.064619	4.658048	5.337062	1.170949	1.638481
gene-AT3G	6.778191	7.113842	5.45608	39.83323	40.93428	42.41672	9.380001	10.32725
gene-AT1G	48.70704	50.75434	49.60644	49.51066	47.15796	45.14362	17.72714	16.97782
gene-AT5G	2.604991	2.516694	2.110253	1.682069	1.89039	1.701912	1.764887	2.959172
gene-AT5G	57.85786	56.01858	58.1954	61.76056	54.94694	56.57072	73.90255	72.18618
gene-AT1G	1.080408	0.633682	1.341668	0.512564	0.913664	1.618859	0.145572	0.160254
gene-AT3G	12.79488	12.39933	13.33339	8.819252	9.345063	7.710614	23.09346	22.48779
gene-AT3G	16.76591	15.5599	15.48502	9.824234	10.06027	9.999525	21.69763	18.99725
gene-AT1G	20.34632	18.25267	18.45665	17.42912	16.15461	18.07128	26.55796	29.00119
gene-AT4G	0.735808	0.888702	0.746412	1.060111	1.043509	0.885537	0.533417	0.274773
gene-AT4G	2.970293	2.731784	3.164231	3.085926	1.846861	1.831736	0.443443	0.897623
gene-AT5G	7.156285	7.367422	7.832814	10.7994	8.962497	9.418697	10.9043	11.58393

gene-AT1G	4.680823	2.961271	4.434871	3.800267	4.119288	3.42338	4.976441	5.155723
gene-AT5G	4.197209	3.294608	3.392164	3.650327	4.089731	3.407584	1.284379	1.625512
gene-AT1G	0.123521	0.189817	0.160104	0.697036	1.357195	0.496195	0.902116	1.275106
gene-AT1G	51.03552	49.67351	49.71484	25.10559	25.70619	23.07517	10.52642	13.14926
gene-AT3G	17.96672	18.95049	19.49958	19.45017	19.02824	20.0606	30.11301	33.14315
gene-AT1G	5.570287	5.590888	6.621668	4.846969	4.331295	3.814347	1.94099	2.242408
gene-AT1G	85.21647	79.81583	88.40627	67.19964	70.33103	66.51135	107.9986	111.1223
Arabidopsi	0.181318	0.199361	0.204751	0.178819	0.258748	0.256816	1.419127	0.765453
gene-AT1G	0.414459	0.340896	0.273509	1.223204	1.148332	0.70787	0.629867	0.130276
Arabidopsi	0.827132	1.334131	0.854931	0.572807	0.941672	0.954844	0.78933	0.20883
gene-AT3G	0.109401	0.067961	0.076366	1.959517	2.024906	1.982306	0	0
gene-AT4G	3.016937	3.625824	3.196599	1.665336	2.286311	1.467795	0.830263	0.667902
gene-AT4G	2.582839	2.588085	2.929152	5.629884	4.902173	5.069369	0.683277	0.533301
gene-AT4G	5.129014	5.549938	5.184055	4.952067	5.758509	6.369787	4.09381	2.679262
gene-AT1G	21.65651	22.45822	24.05712	38.85159	36.07867	32.86509	18.13922	17.49922
gene-AT1G	2.192971	3.219487	2.706096	2.269847	2.175197	2.686457	3.635979	3.553046
gene-AT5G	12.98323	14.53662	14.44586	12.89165	12.23232	11.21889	5.391271	5.511518
gene-AT5G	23.23799	23.2416	22.43972	11.36021	11.13989	9.237058	8.546831	8.960294
Arabidopsi	2.854266	3.981596	2.621654	6.259573	6.182005	8.356073	1.743795	1.221145
gene-AT2G	4.465538	5.108957	5.313717	3.014213	3.649306	4.159817	8.186253	7.567163
gene-AT1G	10.16419	9.826893	9.560579	11.19217	11.08695	10.33533	4.442898	4.914909
gene-AT1G	4.213809	3.491048	3.759642	5.493714	6.407128	4.953192	4.715104	5.731264
gene-AT2G	3.851291	3.906459	3.702063	3.470774	3.095511	3.139069	5.167031	5.137413
gene-AT5G	0.028146	0.090981	0.025644	1.200923	0.816903	0.707172	0.134283	0.124928
gene-AT1G	44.91736	44.23477	45.83793	58.91683	58.65982	61.04644	96.39858	99.78223
gene-AT3G	42.66742	41.51263	42.56463	29.28369	29.901	28.77164	55.1009	54.53325
gene-AT2G	2.134859	2.138236	2.270267	2.753455	2.847755	3.820868	0.893377	0.889506
gene-AT2G	0.71713	0.929333	0.811916	0.93274	1.473237	1.291243	0.157752	0.233143
gene-AT2G	44.23748	38.72387	42.08086	59.48354	61.78542	61.09405	10.67066	14.40173
gene-AT5G	6.629696	6.914438	6.950676	5.678081	4.531953	4.359415	8.343771	10.01621
gene-AT5G	7.565108	6.972277	5.01915	8.772228	8.215755	8.739632	9.168535	9.27203
gene-AT1G	22.59402	21.43129	21.16356	10.48497	10.3486	10.4777	24.02141	25.5826
gene-AT5G	8.189131	8.93291	9.697162	14.71705	13.83196	12.72702	7.988257	7.933345
gene-AT3G	0.653698	0.724609	1.071398	2.283922	1.771304	2.770743	1.487454	1.452005
gene-AT3G	0.172376	0.278853	0.349351	0.904878	0.608046	0.594251	0.11479	0.096865
gene-AT3G	4.398478	4.36463	3.684336	282.5592	283.7614	286.9476	3.682685	3.506024
gene-AT4G	1.374998	1.349373	1.401069	0.541524	0.079942	0.58505	0.524619	0.222566
gene-AT4G	14.901	18.07932	20.96755	22.95941	22.63615	22.6247	13.92706	10.86354
gene-AT3G	6.576401	5.161585	5.408312	3.890258	3.636643	3.963192	5.085866	5.366876
gene-AT5G	0.317156	0.387417	0.12532	0.428483	0.436068	0.575552	2.556145	1.404628
gene-AT3G	5.523995	5.486002	5.690386	11.09668	9.242067	11.32991	3.468532	3.09101
gene-AT5G	134.078	126.7838	134.724	111.7128	112.8394	117.3217	193.1435	202.7838
gene-AT3G	443.6089	454.0056	457.9992	299.2849	310.2204	299.2711	625.7729	634.1228
gene-AT1G	5.312242	5.633339	5.431873	1.239679	1.619673	1.862347	8.422584	8.896955
gene-AT1G	6.469131	4.676964	5.409488	21.19733	20.64186	22.43614	1.298037	1.313805
gene-AT5G	0.099027	0.204038	0.06555	1.035104	1.423146	1.843916	0.204336	0.212674
gene-AT1G	2.171245	1.900796	2.73644	0.456561	0.512076	0.478242	0.851316	0.942944
gene-AT3G	12.59926	10.40684	11.12199	5.048183	4.032664	5.110531	3.751042	4.38365
gene-AT5G	1.78213	2.321433	1.337464	2.704939	3.975003	2.867357	1.100228	1.263961
gene-AT5G	10.415	12.09748	12.10487	6.479128	8.366267	6.402312	12.89253	13.06373
gene-AT5G	2.953221	3.597247	2.675481	3.588872	3.505884	3.327863	1.505124	1.175786

gene-AT1G	2.613932	3.002783	3.431893	2.412732	1.776655	2.224544	6.500004	5.295354
gene-AT1G	7.799531	6.901764	6.406761	8.072964	9.354141	8.909878	5.014141	4.591661
gene-AT3G	6.75132	5.417962	5.193036	1.690708	2.20646	0.978681	1.087891	1.059699
gene-AT1G	8.930071	9.893047	8.83788	7.585588	8.483412	8.853723	65.37421	71.80823
gene-AT1G	63.29822	61.96741	61.63934	98.79554	102.0966	98.09049	37.37749	38.76949
gene-AT5G	1.772755	1.787413	1.595681	2.513131	2.357744	2.902769	1.661866	2.05999
gene-AT5G	37.96471	39.98847	38.75683	28.45647	24.39716	27.10734	55.31312	58.32842
gene-AT5G	6.237542	6.155833	6.411668	6.354073	6.958652	7.017476	3.059129	3.53626
gene-AT1G	21.97128	21.94801	22.28323	27.94171	28.85894	28.64554	32.29111	33.82884
gene-AT3G	2.238678	2.58105	2.678299	1.96948	2.225573	1.966306	1.890329	2.486854
gene-AT1G	64.00357	67.82271	63.6044	34.38785	34.61313	37.29329	78.3318	82.81049
gene-AT4G	16.40853	16.59661	15.5575	4.812597	4.397191	3.572925	34.79018	36.56405
gene-AT1G	2.961345	3.954224	3.203173	2.835156	2.624984	1.605563	0.684482	0.528199
gene-AT1G	6.31972	5.241659	5.167385	5.710478	5.886012	6.783327	7.311976	7.682166
gene-AT1G	0.521219	0.977928	0.751842	2.080303	1.14371	2.036989	0.580408	0.968045
gene-AT4G	3.364184	2.905375	3.358331	4.146014	3.356021	3.170979	1.339739	1.717063
gene-AT5G	0.660315	0.644299	0.831548	1.902757	1.95876	1.444028	0.117414	0.439709
gene-AT4G	3.464696	3.070456	2.829774	3.533967	3.160498	3.221859	4.843068	5.718997
gene-AT1G	10.14449	9.748026	9.595155	11.5501	11.23623	12.47361	20.03946	19.60382
gene-AT4G	8.34179	5.625314	17.53522	13.30089	25.49247	11.50935	3.130656	0.168676
gene-AT1G	31.82039	31.01737	29.63567	21.74401	23.01667	22.16334	31.2198	31.34627
gene-AT1G	13.88029	14.19998	14.03497	7.327504	5.877719	6.610567	41.21345	42.06109
gene-AT4G	2.956367	3.137287	2.155969	1.505982	1.233691	0.862901	7.725038	6.899268
gene-AT4G	5.140338	4.68037	4.017108	10.72353	11.07046	11.83857	2.956215	2.252701
gene-AT4G	5.008969	7.363751	4.539736	8.787377	6.900801	8.207551	7.461737	7.654542
gene-AT4G	0.378604	0.523827	0.897688	0.712986	1.194273	0.728261	0.085905	0.149953
gene-AT4G	1.358481	1.503717	1.680307	5.41649	5.293665	6.075661	3.453015	3.699485
gene-AT1G	5.016004	3.899848	3.601499	4.106756	5.649568	3.997609	1.696313	2.108068
gene-AT3G	0.428942	0.577094	0.447603	0.361942	0.260377	0.431862	1.508695	2.170761
gene-AT1G	5.39058	5.82023	5.304005	7.703768	6.800113	7.83211	3.68478	3.546171
gene-AT3G	2.957047	3.206739	2.981878	4.146315	3.879745	4.571346	2.597167	3.498921
gene-AT3G	21.74709	22.03348	21.00692	62.23822	59.80146	59.95789	13.48642	13.48599
gene-AT1G	0.457454	0.183415	0.316702	2.594118	2.856522	1.995162	0.060285	0.050101
gene-AT2G	59.10808	60.82065	62.15743	51.06809	52.07995	49.84968	96.90347	100.007
gene-AT4G	2.890263	3.172696	2.535248	4.079063	4.709982	3.729046	1.005448	1.659258
gene-AT2G	2.861816	2.511106	2.828729	6.816679	5.746563	4.760598	1.763898	1.665525
gene-AT2G	140.4851	209.9373	195.4534	245.5982	257.5591	322.7142	1702	581.6684
gene-AT4G	8.034156	8.055696	7.412602	5.995674	6.083696	5.952487	8.291924	8.510295
gene-AT1G	3.859236	3.136058	3.383547	0.848926	1.430986	1.104778	4.506174	4.365598
gene-AT3G	10.32824	10.18574	10.26789	0.381599	0.540714	0.54205	0.643769	0.534964
gene-AT4G	41.92314	35.27596	37.04176	40.22999	41.82922	45.93263	50.61584	46.38931
gene-AT3G	18.18637	18.31999	17.73305	10.72662	9.494364	11.13531	22.85112	24.81902
gene-AT5G	0.756386	0.354758	0.663012	0.815924	0.521186	0.602303	0.731616	0.922394
gene-AT4G	12.95663	13.32071	13.23887	29.24092	31.93715	29.628	8.176281	6.564569
gene-AT3G	2.038388	2.428275	1.443531	5.256279	5.895673	6.624939	2.523096	2.803528
gene-AT1G	0.944372	0.897312	1.078264	0.327769	0.181476	0.310105	0.35506	0.241302
gene-AT2G	9.636937	8.955409	11.29148	12.26015	11.80701	11.63806	5.631353	6.244436
gene-AT2G	2.655103	2.760485	3.351934	1.113498	1.059092	1.19334	1.864428	2.514267
gene-AT5G	12.88307	12.1476	12.31564	3.564889	3.031487	2.717107	4.79782	5.852024
gene-AT5G	22.9965	22.13694	20.48587	9.495621	8.56628	9.28598	12.86813	14.41222
gene-AT5G	1.655599	1.636198	1.851169	2.026345	2.222402	1.706645	0.528421	0.199317

gene-AT2G	8.616838	7.33242	8.661926	5.414948	4.521732	4.219653	10.8481	10.68548
gene-AT3G	0.790858	0.882827	0.932155	1.082831	0.897833	1.235608	0.379183	0.131017
gene-AT5G	4.039246	3.166467	4.199752	2.803397	2.28855	2.279239	0.406579	0.32045
gene-AT1G	88.31628	87.37129	87.48054	49.90164	48.61062	47.42978	168.4947	168.6681
gene-AT1G	4.303191	4.71801	4.777522	3.413801	4.206764	3.876626	1.804976	1.864531
gene-AT1G	2.122552	2.090261	2.255896	1.339942	1.146139	0.864616	3.466654	3.711233
gene-AT5G	1.780401	1.090097	1.929575	1.772476	2.884185	2.220659	0.456755	1.246387
gene-AT2G	4.849983	4.665572	5.495044	10.82162	11.76765	11.89654	3.703292	4.065691
gene-AT5G	14.69581	13.94969	13.85371	5.803541	5.519551	6.499606	10.3768	12.50262
gene-AT5G	12.74563	11.7529	10.54813	11.76847	11.73792	14.74811	11.5787	12.40223
gene-AT4G	1.75438	1.670131	1.600121	1.135969	0.639586	0.727102	0.454882	0.4025
gene-AT3G	8.766975	9.393203	8.478644	4.072988	5.2256	5.435846	6.079224	6.475036
gene-AT1G	90.53565	92.73584	89.19969	86.26984	86.57849	86.91115	25.40551	26.41342
gene-AT2G	1.328816	1.206384	1.162791	0.485332	0.684215	1.083304	0.37763	0.595047
gene-AT1G	18.29832	20.28477	21.18149	4.40828	5.136789	4.30345	56.32655	62.6555
gene-AT4G	40.79745	43.48453	41.33399	66.2173	69.00811	64.20509	22.14337	25.24731
gene-AT4G	13.75869	13.13123	13.93537	10.83516	11.49563	11.0684	3.480708	3.047283
gene-AT5G	9.392709	8.510347	8.394443	6.668654	7.156751	6.258022	9.291754	9.711421
gene-AT4G	0.079191	0.148046	0.220044	0.89774	0.819161	0.778889	0.228383	0.221433
gene-AT5G	75.56589	74.43578	75.18745	278.6668	283.5122	279.4815	32.53914	32.26946
gene-AT3G	15.55442	18.18221	17.37446	12.31681	13.1584	12.48667	22.38171	18.96395
gene-AT3G	5.929034	6.17401	5.706296	2.514969	3.357207	2.930549	4.339271	6.058345
gene-AT1G	0.161842	0.133134	0.232786	1.073491	0.642261	0.731437	0.159471	0.263719
gene-AT5G	1.501366	1.300703	1.146998	1.833282	1.650542	0.862132	0.043262	0.128819
gene-AT3G	9.693014	11.21997	11.1205	15.53596	15.2582	15.8867	2.45795	2.368322
gene-AT5G	3.35844	2.857206	2.55156	1.094984	1.863902	1.963944	4.268979	4.773665
gene-AT4G	0.626442	1.032342	0.681232	0.96106	1.143246	0.804239	0.975216	0.637281
gene-AT1G	6.506689	7.752277	8.077129	3.479501	4.464051	4.152198	8.286556	7.412046
gene-AT5G	10.08244	11.1751	9.674989	9.185321	8.224173	7.813133	13.36796	14.1137
gene-AT5G	39.82701	40.59816	34.93358	30.82604	33.70997	29.9223	29.7328	32.82229
gene-AT4G	46.78877	45.74542	42.8247	86.3697	82.17602	72.17245	30.0016	31.74017
gene-AT5G	2.268513	1.290225	2.035821	0.324238	0.182581	0.628416	3.769127	4.016915
gene-AT1G	12.53336	12.68736	13.16921	20.74414	20.98093	19.8275	3.7962	4.108015
gene-AT4G	28.85175	26.85455	30.18157	34.23668	29.01011	30.87707	13.56508	13.13996
gene-AT5G	1.697211	1.17424	1.242938	2.809994	3.501506	4.085578	0.836507	0.396466
gene-AT1G	2.915063	3.122899	3.782501	2.644939	2.35687	2.744521	0.530169	1.176842
gene-AT1G	8.546916	8.332499	8.759093	8.629219	10.14709	9.814667	9.037724	9.164255
gene-AT3G	2.133974	1.906947	1.446768	1.723413	2.794278	3.059812	2.953339	4.059557
gene-AT2G	36.84664	33.01197	37.10502	12.83883	13.90505	12.69772	72.16296	74.88381
gene-AT1G	5.959823	5.98062	6.058359	3.343252	2.888567	2.358812	3.323332	2.908835
gene-AT5G	13.48719	13.74448	15.395	13.29273	14.85295	11.90441	18.08578	15.07114
gene-AT1G	15.88811	15.19798	14.50709	5.490713	5.875165	5.245726	14.48183	16.58804
gene-AT5G	4.311741	3.409892	2.953907	1.308226	1.095316	1.580627	2.577852	3.756827
gene-AT4G	4.61136	4.464961	5.107347	7.520148	7.822923	8.27028	1.754666	1.151178
gene-AT1G	154.6569	140.9722	148.2841	110.6859	108.2571	102.9124	228.6694	234.6968
gene-AT1G	11.6722	12.15125	11.77834	10.82677	10.80034	12.38914	13.28595	12.75901
gene-AT1G	39.04559	40.0592	39.37163	41.05164	41.98075	40.46311	50.40165	46.57642
gene-AT5G	29.02895	28.13998	31.08638	20.18454	20.8161	19.24874	27.52848	31.41542
gene-AT4G	2.714035	2.138783	3.211206	2.896368	3.683199	2.782965	0.614208	1.216287
gene-AT2G	1.871125	2.133268	1.574065	0.281254	0.166719	0.605742	1.028592	2.323619
gene-AT1G	35.06014	37.79441	36.40744	23.96197	21.76928	22.99098	11.63734	10.52743

gene-AT5G	7.182314	6.662823	7.144704	13.43212	12.36528	12.8708	1.576658	1.83955
gene-AT5G	5.808073	5.278215	6.055742	6.925293	7.010741	6.861358	10.1876	10.44018
gene-AT4G	5.27946	6.418096	6.468404	5.233874	2.995052	6.151886	4.654397	4.314973
gene-AT5G	10.52808	9.590934	11.07755	8.01418	7.974242	8.433363	41.0042	43.79557
gene-AT2G	1.142839	1.711099	1.490557	2.904664	3.610709	3.845135	3.264566	3.812775
Arabidopsi	6.343429	6.354011	5.352225	1.594731	1.498546	0.765345	3.007985	2.772621
gene-AT4G	29.88398	36.72236	26.16362	40.5544	42.25468	42.11304	40.81452	40.49204
gene-AT5G	12.00279	12.07797	12.28244	27.58663	30.52185	27.89221	6.239638	8.194307
gene-AT2G	69.52252	70.17135	66.39348	34.3924	35.99908	35.36215	69.05195	72.3604
gene-AT5G	1294.629	1250.12	1287.985	1307.577	1289.392	1281.719	2112.336	2047.294
gene-AT5G	4.425183	3.267262	2.805981	4.75122	5.538013	3.759452	1.323313	1.305728
gene-AT4G	5.567469	4.438493	3.854459	5.272307	5.807377	5.446289	5.67269	5.779962
gene-AT3G	33.29112	32.1666	36.09572	56.19689	52.14186	49.46724	21.74896	21.22399
gene-AT3G	0.893106	0.475869	0.582495	0.608206	0.270742	0.586216	0.219553	0.305302
gene-AT4G	11.20611	11.1429	11.98702	9.42977	9.256769	9.883751	12.7743	13.84548
gene-AT1G	20.44925	19.24204	20.38578	7.613514	7.520114	8.295345	40.98708	41.76225
gene-AT3G	15.5311	20.86247	16.61141	6.428823	8.176037	7.243299	9.642377	11.79409
gene-AT2G	10.28398	10.32994	12.79103	31.12963	33.49655	30.91485	0.503106	0.314223
gene-AT3G	1150.567	1162.737	1147.344	415.734	410.5983	415.1689	2031.317	2016.117
gene-AT5G	7.29096	7.37727	7.092817	6.328834	4.80393	4.729178	2.011495	1.669509
gene-AT2G	2.156114	1.7252	1.386671	1.079992	1.062317	0.934757	2.745598	2.440038
gene-AT5G	1.382245	1.390893	1.032128	1.034708	1.036165	1.044879	0.683233	0.534105
gene-AT5G	0.999614	0.673119	1.010164	2.994661	2.918991	2.055058	1.200737	1.369764
gene-AT1G	2.275562	2.508489	2.4102	2.297938	3.359727	3.835535	0.609303	0.571813
gene-AT5G	17.66867	18.48712	21.32573	28.10125	28.52548	28.87287	46.40193	48.76049
gene-AT1G	0.846738	0.972248	0.90311	1.065378	0.9385	1.142076	1.165858	1.612201
gene-AT4G	19.63082	18.49351	19.90503	19.57665	18.36757	18.52178	25.89539	27.86266
gene-AT5G	0.030978	0.045883	0.0345	1.142273	0.281969	1.202131	0.434277	0.147591
gene-AT2G	4.567425	4.293185	4.652897	19.47959	18.80892	20.08832	4.733935	5.616438
gene-AT3G	9.036845	10.35252	8.946649	4.459821	3.8805	4.306199	19.15865	17.99348
gene-AT1G	7.719941	5.596595	7.526406	6.040167	4.831701	4.433098	2.027977	2.332711
gene-AT3G	19.07188	23.64207	16.58654	42.67251	45.72434	46.10126	15.03138	12.39921
gene-AT5G	7.67592	8.398678	7.953959	13.14742	13.35761	12.38413	7.825258	8.78495
gene-AT5G	18.70331	19.17017	19.14237	12.98439	13.33886	12.66301	35.22673	36.227
gene-AT2G	4.067119	3.438746	2.829231	1.171693	1.568064	1.365819	4.486009	4.117834
gene-AT3G	163.6552	168.3804	163.4062	119.1532	116.7725	116.7721	229.4613	244.2255
gene-AT3G	17.12999	16.7686	16.03193	7.69592	8.110353	9.057423	6.33206	5.09314
gene-AT5G	1.665264	1.544839	1.722209	1.856081	1.891078	2.405282	2.238116	2.655276
gene-AT1G	3.778408	2.255068	2.533824	2.24339	2.765357	2.379032	3.081057	2.676948
gene-AT5G	3.834008	4.588997	3.606303	2.446271	3.097319	3.383694	1.599616	1.649485
gene-AT1G	6.750017	6.319193	6.980876	14.82642	11.87735	14.17527	9.912275	8.451511
gene-AT1G	3.17471	4.04099	3.753875	3.87272	4.281541	3.645814	4.382267	3.629884
gene-AT4G	35.46502	34.43972	36.48745	19.62342	19.35894	17.54177	124.9385	131.9426
gene-AT5G	27.15764	27.86837	26.32077	30.61518	31.24541	27.7044	9.187973	9.767468
gene-AT5G	9.623982	8.088353	7.29334	10.98793	10.98515	12.09039	10.583	14.25615
gene-AT2G	998.2686	969.3002	1003.979	462.0198	472.1992	458.1474	1471.368	1528.239
gene-AT5G	6.560096	6.854269	6.21283	10.37121	9.336932	11.20155	4.204331	3.397558
gene-AT5G	428.4521	414.3733	431.4495	61.30397	64.05647	61.63017	84.02628	82.94901
gene-AT2G	8.632119	9.666623	9.286824	4.793433	5.320014	4.992291	17.35355	16.85291
gene-AT5G	8.277591	7.580709	8.36305	14.32634	13.88559	12.92263	6.939114	7.316356
gene-AT5G	1077.433	1074.446	1054.157	503.9712	549.7659	526.41	157.9715	157.0753

gene-AT4G	6.574463	6.645756	5.898975	11.76785	10.96702	11.10498	2.561678	2.403165
gene-AT3G	32.29854	34.05642	31.7744	14.25162	16.21297	14.32836	80.72509	73.75487
gene-AT5G	12.23372	12.9094	11.92518	9.501419	8.805842	9.19704	12.03472	13.35154
gene-AT3G	2.856462	3.492425	3.142685	14.93017	13.99596	13.33661	0.254346	0.217283
gene-AT5G	2.5321	2.977059	2.988912	3.577699	3.805583	4.396513	1.152034	1.734441
gene-AT5G	27.54632	25.37421	26.96705	16.67308	18.67985	17.86736	33.98589	33.44775
gene-AT2G	6.77832	7.106328	6.425062	3.503932	3.993577	4.133042	15.38006	15.23731
gene-AT3G	29.70919	27.84406	30.1597	24.02647	24.29417	24.68356	26.35047	29.6899
gene-AT1G	45.68764	44.73183	47.03855	36.50653	33.76342	34.64306	73.2996	80.29975
gene-AT1G	11.50547	11.25295	11.08234	14.62496	14.43159	15.9956	3.176695	3.067251
gene-AT1G	77.38738	77.09097	79.62234	59.56215	58.94598	58.55447	129.5952	132.0224
gene-AT5G	7.2978	8.039624	7.354421	8.028546	7.553002	7.268328	2.783601	2.7758
gene-AT1G	26.3494	29.02283	26.61835	31.66512	28.72879	30.2593	11.37116	11.42061
gene-AT2G	4.427981	4.131083	4.576204	3.871196	4.625982	4.336869	2.549464	1.754453
gene-AT4G	26.28544	25.51451	26.20369	22.64305	23.05011	22.92985	38.8511	43.09202
gene-AT1G	55.44038	53.79012	50.80784	58.3425	55.33668	51.75666	88.14823	94.83384
gene-AT1G	4.964122	5.562299	5.413448	5.889476	6.530473	5.11542	1.977194	2.44106
gene-AT4G	13.13759	13.14976	12.50791	13.74831	14.57475	13.18902	17.82823	19.93114
gene-AT1G	10.48323	8.995498	10.24019	13.41201	15.26177	14.3576	1.763861	2.66458
gene-AT2G	1.236055	1.112113	2.060592	5.462754	5.345235	4.954358	0.692689	0.840524
gene-AT2G	21.77093	22.45117	22.29142	26.94102	28.71341	28.84824	17.4461	17.42151
gene-AT4G	9.435349	9.928733	10.81089	16.95597	19.01068	17.67701	7.159004	6.983667
gene-AT4G	0.693718	0.994743	0.798591	2.419082	2.113292	1.951131	0.404191	0.572326
gene-AT3G	3.796199	3.309457	2.801682	3.520541	3.575938	4.151844	1.233305	0.514692
gene-AT2G	0.100263	0	0	0.095782	0.491889	0.380612	0	0
gene-AT2G	25.31823	25.15097	27.06115	13.04954	12.07308	14.70476	40.61045	42.25441
gene-AT2G	0.634173	0.341787	0.514702	1.00701	0.494498	0.556952	0.13297	0.457621
gene-AT3G	3.033535	2.736197	2.595776	2.851603	3.999405	3.419984	0.318268	0.432858
gene-AT5G	9.718136	8.974833	8.222258	19.59417	17.33156	18.38084	1.518488	1.861541
gene-AT2G	1.302481	1.278938	2.057869	2.459559	2.077584	1.889102	1.035981	1.528515
gene-AT3G	0.958522	0.673106	0.830517	1.16076	1.249981	1.315611	1.77088	1.478863
gene-AT5G	7.943921	5.483329	7.706334	7.604157	7.756448	8.077418	4.141374	3.821561
gene-AT5G	2.594165	3.067593	3.095755	3.245748	2.751425	2.655116	2.031039	2.104406
gene-AT3G	3.321738	3.582877	3.192414	5.223549	4.162372	4.606421	1.256794	1.099672
gene-AT3G	0.99647	0.878995	0.80702	2.109634	1.847687	1.136878	2.948218	2.447245
gene-AT5G	2.642663	2.730886	2.784838	1.189078	1.824986	0.750253	0.550715	0.423343
gene-AT1G	2.307555	1.689378	2.994417	1.79776	1.430912	2.661873	4.794165	3.901882
gene-AT1G	2.835046	3.781213	2.945643	0.17274	0.533076	0.633381	5.529034	3.941758
gene-AT1G	3.561298	3.541343	3.155475	5.709797	5.096319	5.287361	2.033374	2.296116
gene-AT5G	24.75153	23.60052	23.08008	22.40373	20.43823	19.83306	21.23318	21.26008
gene-AT3G	4.973583	4.449895	5.52576	0.641524	0.804919	0.827471	7.358316	8.69054
gene-AT5G	3.619693	3.868266	4.122634	9.529368	7.855566	9.395716	0.304201	0.448926
gene-AT5G	1.281452	1.031452	1.914418	2.387806	2.508289	2.240157	2.840637	1.729367
gene-AT2G	2.176854	1.743194	1.398525	5.478888	4.768062	5.456438	0.40815	0.952698
gene-AT1G	0.602697	0.409243	0.697025	1.798445	1.214906	1.412661	0.346859	0.742163
gene-AT5G	1.213804	0.85093	1.031439	1.729923	2.167128	1.831983	0.965499	1.215621
gene-AT1G	1.024797	1.056192	1.515731	1.758674	1.702412	1.707462	0.712129	1.415614
gene-AT1G	1.245943	1.770145	1.109378	0.248323	0.081324	0.416975	3.195374	4.086457
gene-AT5G	9.260654	9.311557	9.997835	9.742653	9.54057	9.68316	8.654912	8.91502
gene-AT4G	0.444705	0.677925	0.925627	0.756706	0.851276	0.602517	0.713867	0.682342
gene-AT2G	10.67128	8.908705	11.93428	5.948015	6.888046	4.711785	9.702897	9.96455

gene-AT1G	13.41355	12.3315	12.88996	9.767647	11.66413	10.25106	16.08341	17.52027
gene-AT1G	34.89853	32.17175	33.79865	27.68894	28.01821	26.32584	12.22466	14.13432
gene-AT3G	8.586674	8.704355	7.311247	5.201452	5.537665	4.782026	14.03922	15.21746
gene-AT3G	26.218	24.18513	27.82752	20.72012	18.86104	21.60267	51.36368	46.46358
gene-AT5G	0.327332	0.653282	0.773958	5.343101	5.353586	6.350197	1.077395	1.76184
gene-AT4G	4.052149	3.560047	4.115698	3.310724	3.405395	3.047485	8.751881	10.13701
gene-AT4G	0.251002	0.108301	0.086601	0.090689	0.135404	0.275731	0.240641	0.387418
gene-AT4G	2.256866	1.201136	1.830629	4.870774	5.805203	4.975083	0.816779	0.604877
gene-AT1G	1.131972	1.188513	1.00315	0.417502	0.728088	0.417961	1.65641	1.418692
gene-AT1G	7.090067	7.621322	8.383284	5.32146	5.028581	6.340791	10.36654	10.34054
gene-AT1G	1.184923	0.811651	1.159505	1.962648	2.092642	1.627887	0.336414	0.525001
gene-AT3G	50.94414	49.6697	49.85878	43.33487	41.37145	39.3601	54.25002	57.61759
gene-AT4G	4.36782	4.8491	4.871893	2.741095	2.599679	3.657939	5.145491	5.682555
gene-AT3G	15.08486	13.79865	15.41199	19.7122	17.91672	20.13163	0.622204	0.804871
gene-AT3G	7.427963	10.3787	7.635456	8.349859	7.703297	7.976279	17.49293	18.26603
gene-AT4G	0.398989	0.593062	0.32592	0.939804	0.733633	0.83478	0	0
gene-AT3G	9.596597	8.364972	10.79462	4.917663	5.833594	5.548271	4.490135	3.612613
gene-AT1G	186.4226	182.0594	196.1346	241.585	240.6183	237.2682	56.26239	60.89753
gene-AT4G	2.211094	2.37605	2.288327	2.331868	1.842068	2.052118	1.071543	0.996816
gene-AT5G	17.55731	18.32138	18.47739	24.26983	24.99879	23.99861	21.41865	19.34081
gene-AT1G	0.938738	0.792471	0.482039	0.654964	0.521327	0.584789	0.224565	0.287073
gene-AT1G	4.330992	3.791674	5.080533	7.446202	5.939978	6.934178	2.241357	2.668801
gene-AT5G	0.530253	0.351058	0.292902	9.086822	7.909531	9.62802	0.294218	0.409323
gene-AT5G	17.50995	21.98974	19.31588	42.65319	43.88667	42.28657	22.3766	21.46948
gene-AT5G	17.62347	17.63103	17.54854	14.86474	13.46532	14.34264	18.07653	17.20062
gene-AT2G	2.175957	1.893254	1.715599	1.930888	1.912129	1.995187	0.6508	0.919099
gene-AT3G	11.63858	9.871181	11.27349	5.30346	5.166701	4.363619	24.40376	28.38667
gene-AT3G	12.82519	10.13954	12.69198	11.84788	13.67019	11.73032	4.157288	3.755106
gene-AT3G	8.037099	7.199688	7.829003	6.044436	5.483323	5.859132	8.317228	8.796145
gene-AT5G	27.36042	31.8091	29.55597	36.46979	38.84855	39.09077	20.59872	22.13405
gene-AT4G	4.331869	4.454101	6.777253	4.990536	4.258801	4.763703	5.989975	5.737805
gene-AT3G	1.523387	1.019175	1.117861	2.190049	2.079567	1.836913	0.777642	0.827703
gene-AT5G	81.06863	75.11679	78.32228	39.51976	37.8364	43.07914	152.8755	154.2469
gene-AT2G	4.481937	5.460845	4.826718	5.988187	5.661382	5.533855	2.815174	2.859749
gene-AT1G	0.709	0.726181	0.623073	1.223693	0.971393	1.321213	0.57846	0.468994
gene-AT2G	21.63752	20.67529	20.37886	7.821831	9.823702	7.627594	6.642854	6.339103
gene-AT5G	14.50644	13.88808	16.12912	6.682795	5.093115	5.183081	2.985055	2.951298
gene-AT3G	62.98929	64.62867	63.4475	62.1761	63.85658	57.17881	80.28106	70.02375
gene-AT3G	1.988108	2.270454	3.016939	4.253997	4.504516	4.158396	1.38884	1.084317
gene-AT1G	8.991961	8.81318	8.24697	8.032077	6.74965	7.503497	1.634471	1.740713
gene-AT4G	0.792483	0.345248	0.602989	2.719203	2.09472	3.472587	0.162524	0
gene-AT5G	5.099845	5.512328	6.066841	13.20334	14.08802	13.47759	5.009038	4.985481
gene-AT4G	8.325592	9.480002	8.475476	12.75929	14.43261	12.18938	2.219509	2.430477
gene-AT1G	3.739837	3.946938	2.827052	2.771599	2.013077	2.922075	9.907969	9.938235
gene-AT4G	0.941951	0.747831	1.450259	2.511065	1.712208	1.636339	3.547244	4.76403
gene-AT1G	26.13908	25.93339	24.26483	17.69435	17.33222	17.52834	35.14314	36.20462
gene-AT4G	12.9839	12.33914	12.6456	26.49134	27.69455	25.6088	18.38525	20.35968
gene-AT2G	7.108179	8.194627	6.059446	25.49311	25.48186	23.86795	2.154642	1.189906
gene-AT1G	4.609944	3.494871	3.533506	4.961094	4.421404	4.789659	2.409111	1.560593
gene-AT1G	9.829042	9.43658	8.913274	4.831247	4.734592	4.299196	4.437574	4.888423
gene-AT2G	169.3932	168.5978	174.7975	138.2558	142.1609	134.6765	148.4351	155.7436

gene-AT5G	5.295987	4.309025	5.029049	4.17275	4.786492	5.150418	5.872935	6.350876
gene-AT4G	2.14845	2.132356	1.618732	1.592558	1.263035	1.087011	0.416536	0.332046
gene-AT2G	6.271444	5.661541	5.209089	1.847338	1.757194	1.88642	2.779224	3.038212
gene-AT4G	0.817329	0.666966	0.905261	1.589707	1.83394	1.65624	2.152188	2.083993
gene-AT2G	27.57361	30.99139	25.75925	40.4215	38.88936	37.57775	89.75762	76.81698
gene-AT1G	10.19257	11.92868	11.07781	8.678719	9.524264	10.18671	35.56672	35.84219
gene-AT4G	187.4057	180.8028	192.7609	207.0614	200.4715	203.6682	80.6898	85.34295
gene-AT2G	12.81114	15.93731	14.47394	29.66236	30.4641	28.92925	29.4766	31.92742
Arabidopsi	1.948361	2.057949	2.167517	2.340751	2.347494	2.881015	5.218045	4.413305
gene-AT4G	508.1794	480.7704	499.0911	211.9368	203.6038	210.9056	759.6898	801.0813
gene-AT3G	0.130753	0.248227	0.04034	0.594851	0.47739	0.438475	0.029825	0.024187
gene-AT5G	10.19899	11.59721	10.82565	16.08978	15.55773	15.1047	3.922432	3.714984
gene-AT3G	318.6598	308.8989	320.0756	243.229	244.7591	241.7981	216.5689	225.9436
gene-AT5G	1.345306	1.389637	1.204037	1.556579	1.751875	1.80569	3.078768	3.236443
gene-AT3G	2.740582	2.963161	3.592917	3.340269	3.778392	3.100708	6.038803	7.056911
gene-AT3G	10.08167	8.434118	10.90493	4.328107	5.420827	6.945772	14.33039	16.06071
gene-AT2G	6.105499	5.966798	6.979834	5.112864	5.055392	4.41941	4.202489	5.193044
gene-AT3G	181.4177	174.6769	176.9916	132.4105	132.803	139.0679	319.8366	328.6649
gene-AT1G	17.84626	16.2472	16.39993	1.750119	1.910684	0.857995	0.233834	0.579707
gene-AT1G	13.63605	12.91078	13.28798	15.23692	15.52278	15.86399	4.281456	4.70004
gene-AT3G	6.689657	6.526972	7.733145	6.218513	5.948871	6.591133	9.457308	9.526205
gene-AT1G	6.024764	4.749005	6.053393	5.860938	7.105517	4.37834	3.064717	2.006913
gene-AT2G	22.40427	24.1624	21.06262	106.6448	103.7326	102.7153	14.28221	13.04305
gene-AT5G	16.87601	15.15676	16.01258	29.85947	29.33679	30.86462	5.222345	6.556566
gene-AT1G	3.256579	2.507333	3.055873	3.362797	3.853958	2.876227	3.225648	3.797223
gene-AT2G	7.753203	10.05296	8.709024	9.693734	8.962763	7.65408	3.407331	4.640431
gene-AT1G	16.29281	16.30314	17.97881	10.51725	9.506148	10.20186	17.63134	18.514
gene-AT5G	0.446459	0.205207	0.275611	0.342022	0.213654	0.197877	0.709906	0.582391
gene-AT5G	2.264699	2.67337	2.984546	1.046593	0.787637	1.297715	1.18344	1.542496
gene-AT4G	48.16391	49.43957	51.78505	31.42866	31.4359	33.33482	68.55973	72.23887
gene-AT3G	22.01325	21.28268	21.91294	19.85228	17.70442	18.27852	34.54274	32.88873
gene-AT1G	5.695692	4.582603	6.392154	6.596847	3.836151	5.755236	7.359668	7.768287
gene-AT2G	141.5234	135.1384	142.0698	99.61468	99.54479	105.5032	187.9338	199.6408
gene-AT1G	1.172061	1.401624	1.238334	2.063177	1.715934	2.073081	0.74484	1.029508
gene-AT1G	0.284653	0.339499	0.37232	1.289293	0.94507	1.510801	0.384207	0.308593
gene-AT4G	46.76201	44.90565	46.27625	32.67039	31.76422	36.06615	61.18765	52.72031
gene-AT3G	11.55372	10.63229	10.32824	7.467881	6.738388	6.918339	5.06033	4.172719
gene-AT5G	9.153141	8.535266	9.011758	6.748568	6.245941	6.410628	8.278468	8.70269
gene-AT1G	3.210255	3.481923	2.425479	6.064552	6.2754	6.406875	2.259062	2.392596
gene-AT1G	37.78991	41.1564	39.64695	23.34681	25.20221	22.69216	69.60812	70.33521
gene-AT5G	5.244882	4.630074	4.665036	4.505937	4.127125	4.232418	1.921528	2.303387
gene-AT5G	2.238685	2.568038	2.437629	1.860349	2.282595	2.035593	1.151907	1.123548
gene-AT4G	1.212125	0.918739	0.806801	0.654721	0.269789	0.583336	0.506312	0.761302
gene-AT2G	7.371099	7.545072	6.913481	12.63092	14.81032	13.62113	3.18151	4.634772
gene-AT4G	1.23096	1.182153	1.257751	1.494458	1.71583	1.198623	2.276095	2.078683
gene-AT3G	5.770435	6.026358	6.186535	5.399171	4.917001	5.277404	9.484384	12.29713
gene-AT1G	9.225786	8.721218	8.044583	12.75635	11.21241	12.55755	2.75558	2.524791
gene-AT3G	5.542539	5.366063	6.116373	3.623819	3.441409	3.701317	0.68628	0.748705
gene-AT3G	27.07296	27.76686	29.83862	54.03109	46.52544	45.4913	42.39847	41.81849
gene-AT2G	16.83211	16.76784	16.13195	39.61467	38.88817	37.81675	19.85547	20.87237
gene-AT5G	56.97101	56.40542	56.40506	84.52098	85.37962	81.29714	51.28072	50.78845

gene-AT5G	0.52668	0.501821	0.760119	0.142381	0.223521	0.15895	2.060627	1.887929
gene-AT2G	593.1707	633.9581	569.991	697.3806	720.4486	715.4745	191.2337	178.2577
gene-AT1G	2.514315	2.69615	3.436979	0.233487	0.468759	0.350066	1.356213	1.229521
gene-AT1G	55.50875	55.6849	56.85916	24.03271	24.57342	25.3601	49.82859	52.67369
gene-AT2G	1.263314	1.120528	0.859284	0.938375	0.914911	1.175162	1.817876	1.668548
gene-AT5G	43.25575	43.02736	43.25293	18.38249	17.01139	17.11017	53.7064	58.30309
gene-AT1G	0.405074	0.388523	0.755586	1.697517	1.061352	1.182087	0.28391	0.540126
gene-AT5G	11.4899	11.08102	12.82616	8.010594	6.282203	8.457033	3.924702	3.113362
gene-AT4G	1.088505	1.469924	1.049365	3.111762	2.59574	2.933925	1.802272	1.942972
gene-AT4G	0.61088	0.666123	0.629686	0.336886	0.357481	1.023122	2.705879	3.102018
gene-AT5G	7.594293	7.518038	8.402135	9.582229	9.134199	7.240177	10.26909	10.17084
gene-AT2G	8.711866	11.88801	8.434311	5.039734	7.105601	6.631591	14.15033	12.19286
gene-AT1G	2.969496	3.110042	2.837127	3.729739	4.380796	3.528371	1.337708	1.244878
gene-AT1G	108.8958	104.6433	111.0746	71.98711	76.74001	80.79179	212.3427	205.4674
gene-AT1G	11.37768	8.172289	12.26656	15.53929	16.47154	16.74627	9.2412	11.55427
gene-AT4G	1.280161	1.26594	1.808827	0.863745	1.141583	0.796538	0.291757	0.57351
gene-AT5G	31.81969	29.54359	33.30639	36.46947	38.0232	36.3247	18.90433	18.35264
gene-AT2G	12.16573	12.63727	12.46006	9.932223	8.52875	9.174429	14.73257	13.98317
gene-AT2G	10.2044	9.26071	9.864287	7.807173	6.452712	6.743624	3.978659	3.283109
gene-AT5G	5.42164	6.008494	5.138493	1.809393	1.146935	1.854925	2.531307	1.946759
gene-AT4G	393.5683	387.2057	391.162	247.975	254.3546	254.5026	693.086	713.9662
gene-AT3G	2.895935	2.608656	3.284327	2.54085	3.353282	2.752561	2.098101	2.287455
gene-AT4G	2.798956	4.359305	4.188739	2.620938	2.009619	1.757097	3.045473	3.494601
gene-AT5G	1.242594	1.363185	1.596183	1.166283	0.880133	1.211437	0.666196	0.870307
gene-AT1G	0.815116	0.734664	0.55323	0.434073	0.600535	0.434771	0.265513	0.15242
gene-AT5G	10.5081	13.6643	12.04971	14.10417	13.36817	15.25673	3.632532	4.053451
gene-AT4G	6.202518	6.352314	7.473183	14.84227	15.34488	15.55504	6.894294	7.963682
gene-AT3G	0.977662	0.586489	0.819701	1.451768	1.415659	0.967817	0.488935	0.062055
gene-AT1G	4.889967	5.335342	5.251278	2.150175	1.947808	1.420046	2.184265	2.727854
gene-AT1G	26.55097	23.16639	23.75996	31.38915	29.5095	29.29322	13.61288	17.38656
gene-AT2G	0.938479	0.791028	0.727783	1.418353	1.082287	1.536123	0.414147	0.594187
gene-AT4G	4.973736	4.345222	4.568712	4.013014	5.898452	5.405182	2.274246	1.924307
gene-AT3G	4.718986	3.389894	5.451934	4.063922	4.146087	4.027137	6.030396	6.707941
gene-AT1G	13.43488	13.05409	12.65932	13.94484	14.81466	14.39465	23.06315	24.31491
gene-AT5G	97.75443	99.25418	96.85736	246.9372	263.3703	254.4807	94.82393	92.86291
gene-AT5G	60.82854	55.55257	62.87834	767.1424	768.4717	758.7737	27.04843	28.16661
gene-AT3G	201.4068	197.9741	195.124	143.8034	146.3523	141.4484	112.85	121.7113
gene-AT1G	3.695405	3.519627	4.135292	1.583249	3.008829	1.644918	4.594628	4.684337
gene-AT4G	26.32539	25.8366	26.0863	17.86724	17.65292	15.34171	27.5944	29.53663
gene-AT1G	11.7265	12.61209	11.08126	9.675028	8.039586	7.673226	8.919709	9.697661
gene-AT4G	7.198942	8.507484	7.202786	7.016198	6.792284	7.229691	10.37805	8.898339
gene-AT3G	9.075264	8.128616	8.683569	3.942866	3.761397	4.525714	8.179941	8.22438
gene-AT1G	34.83986	34.82306	34.8222	17.06959	16.74902	17.4869	49.34002	51.28876
gene-AT1G	85.78138	85.40963	87.5232	55.27663	57.26173	54.037	30.098	28.54018
gene-AT3G	50.86181	49.98787	44.90474	38.98116	42.38981	38.80267	111.7578	116.8525
gene-AT5G	3.413908	2.782738	2.678908	2.353122	2.045417	1.736754	3.24028	3.069195
gene-AT5G	0.394134	0.256899	0.618369	0.575596	0.673391	0.541088	0.363698	0.564854
gene-AT4G	7.151664	7.54699	6.934922	5.09821	4.345427	4.849399	5.644802	5.572786
gene-AT5G	553.5175	585.8576	556.3459	337.2715	328.8175	345.8366	232.3648	212.6206
gene-AT1G	43.42872	42.96955	43.27811	26.74761	27.42431	27.17169	47.45707	48.81217
gene-AT3G	152.9901	156.7897	153.366	87.15675	84.37656	79.98611	324.8445	348.4238

gene-AT5G	32.42505	29.15024	31.83452	27.95645	26.48523	29.61713	76.20808	73.90981
gene-AT5G	6.850192	7.451398	6.950515	7.309177	8.360091	6.676193	2.372855	2.576612
gene-AT4G	0.676508	0.397423	0.444084	0.846399	0.595531	0.604447	0.401018	0.550415
gene-AT5G	93.2764	91.42622	90.10965	45.83875	51.92352	45.98566	106.1537	107.2797
gene-AT2G	0.945713	0.950067	1.083561	1.615866	1.04328	1.07751	1.261476	1.904348
gene-AT3G	2.127718	2.674243	2.300144	2.003018	0.933514	1.753827	0.253421	0.37635
gene-AT1G	8.195279	8.308754	9.157183	7.213673	6.765354	7.470963	7.713804	8.647503
gene-AT1G	21.41732	21.1792	22.46929	17.63465	18.98838	20.30652	37.47137	38.56188
gene-AT1G	5.406598	4.092231	4.873382	2.994494	3.728994	3.083959	0.810816	1.226256
gene-AT4G	0.306309	0.366971	0.122738	0.487686	0.252061	0.403578	1.695565	2.108458
gene-AT2G	2.82765	2.811519	2.165684	3.675861	3.716034	3.44378	7.388427	7.746814
gene-AT5G	0.98098	0.459862	0.268321	1.83145	1.828476	1.790565	1.64563	1.819534
gene-AT4G	78.95309	79.45546	85.73266	85.26914	84.95794	83.34441	118.2639	121.4897
gene-AT5G	0.260975	0.386418	0.230699	0.170723	0.050128	0.190582	0.272729	0.230716
gene-AT5G	7.301877	8.674779	6.82682	15.14983	16.65733	15.33621	1.885	2.692443
Arabidopsi	6.549325	8.932432	8.445055	3.767677	4.346759	2.928849	5.601007	5.551952
gene-AT3G	3.715677	3.355579	4.013483	2.262222	2.369653	2.72693	3.567094	2.850428
gene-AT5G	22.00755	21.25757	20.67558	10.91826	12.45807	10.61178	8.575503	8.436154
gene-AT5G	7.420532	7.787469	8.262166	3.337592	4.010686	3.348548	8.575844	8.700825
gene-AT5G	2.934578	3.151285	2.835388	3.051439	3.124153	3.048383	2.288052	2.473595
gene-AT5G	11.76466	9.626058	10.7136	11.28013	6.952291	7.65922	4.166624	4.92363
gene-AT4G	37.70825	36.15517	37.71054	25.98199	22.13169	25.02562	28.75622	29.48722
gene-AT1G	3.148078	4.579891	3.95512	4.612106	2.410866	3.8171	5.694708	5.497552
gene-AT2G	285.9867	278.3918	282.1974	276.339	285.3572	267.9591	314.5426	314.756
gene-AT5G	2.77036	3.145426	3.182387	13.4499	13.57654	14.23993	1.468565	1.807763
gene-AT5G	11.33772	12.59455	9.6697	3.325442	3.334115	3.854355	5.238689	6.220051
gene-AT1G	1.692516	1.176784	1.839802	0.389511	0.490189	0.433711	0.67276	0.354256
gene-AT2G	27.9447	26.20449	24.93851	19.96142	19.83827	20.80038	15.02702	12.52352
gene-AT1G	4.3285	4.572267	3.977465	10.3906	11.17489	9.325851	2.577595	3.054438
gene-AT3G	17.62406	13.32684	13.4319	11.4199	11.59949	12.17593	24.08363	23.26474
gene-AT1G	22.26676	22.26989	24.14543	15.52576	15.54653	14.4564	36.8308	38.8331
gene-AT4G	26.26661	32.91092	27.36293	18.77298	20.74356	19.17703	40.36778	39.49392
gene-AT1G	12.05821	12.32859	10.64338	11.08052	12.96001	11.44764	19.23224	18.37454
gene-AT4G	15.071	14.28449	14.84143	9.927823	12.26048	9.041052	11.35128	12.8956
gene-AT5G	0.393486	0.4848	1.244844	2.351776	3.57369	2.057029	0.64633	0
gene-AT2G	0.660223	0.590374	0.7554	2.174987	2.527791	1.441926	1.69676	1.458554
gene-AT5G	9.585134	9.84724	10.84217	8.622908	8.866881	8.086544	11.38062	12.49973
gene-AT1G	0	0	0.363867	0.677454	0.439902	0.451135	0.193574	0.168868
gene-AT3G	8.471147	9.208846	9.685924	74.97086	74.79652	73.82526	14.61669	15.22067
gene-AT3G	205.1099	197.9423	206.9712	127.9745	131.5053	126.856	252.1492	266.0447
gene-AT4G	4.646946	3.806311	3.896257	5.074598	4.46619	4.324202	2.614251	2.122417
gene-AT1G	0	0	0	0.100519	0	0	0	0.057294
gene-AT4G	0.68815	0.93776	1.471956	0.639358	0.436439	0.547958	1.813714	1.630264
gene-AT1G	702.5119	669.5898	696.0471	419.9948	409.3784	412.2793	1116.578	1193.341
gene-AT1G	22.24231	20.56628	21.29367	13.55405	14.07362	13.34963	8.170809	8.0123
gene-AT1G	5.354123	4.005631	5.602963	6.964231	6.410426	7.429355	3.721756	3.993341
gene-AT5G	44.07752	42.11455	47.33475	16.82802	18.65579	18.39808	56.88286	59.44391
gene-AT3G	21.4378	19.13836	23.62885	10.01194	9.576089	9.885698	20.81804	22.41593
gene-AT1G	0.591052	0.599569	0.421107	3.744879	1.919686	2.322817	0.264394	0.259128
gene-AT4G	6.94099	8.510017	9.636808	5.691669	5.249088	5.503389	3.377783	2.96243
gene-AT4G	29.58719	29.94894	31.59942	32.18868	30.54062	30.10552	15.58076	16.79139

gene-AT5G	2.775805	2.590717	2.750904	3.462041	3.32709	3.642375	1.00911	0.870175
gene-AT3G	2.044946	2.572987	1.995924	1.873085	2.217056	1.588396	5.792045	4.807067
gene-AT2G	44.54956	42.47392	46.42344	60.0515	52.11472	55.34595	26.43428	32.45168
gene-AT3G	75.94782	71.62656	76.23425	84.16465	83.94986	82.6443	35.89103	34.49867
gene-AT3G	1.901257	2.308016	2.153343	1.936101	3.593757	3.635733	6.116009	6.813862
gene-AT1G	2.403061	1.621968	1.400006	2.182055	2.426293	2.316043	0.351239	0.477505
gene-AT5G	1.891654	2.147606	2.842091	0.685509	0.588166	0.561174	0.990194	0.751111
gene-AT4G	6.592542	6.890909	7.459365	4.830308	4.797413	4.742849	1.36147	1.277704
gene-AT1G	38.89358	39.4026	40.84648	18.80418	19.40599	19.52504	40.07818	42.88645
gene-AT1G	43.81896	39.54353	40.72725	50.60836	50.21881	51.85263	21.76508	25.23433
gene-AT5G	287.0075	291.1013	282.5248	263.6865	275.0886	273.4799	505.8001	500.118
gene-AT1G	10.91736	9.315152	10.4493	10.48458	10.95471	8.803709	4.464069	4.503743
gene-AT1G	1.15385	1.091687	1.243155	2.158752	2.692933	1.819594	2.112234	2.843907
gene-AT3G	0.649214	1.304564	0.576043	0.954714	1.274979	1.04042	1.47139	1.477776
gene-AT5G	1.185731	1.508875	1.201343	1.135601	1.365949	1.089151	1.078865	1.32808
gene-AT4G	0.822648	1.318411	1.231003	2.420523	2.535914	2.411015	1.168203	2.020406
gene-AT1G	7.039706	6.601604	7.020572	7.289895	7.143253	6.748313	14.86615	15.7281
gene-AT3G	27.14263	26.94148	27.10724	18.87721	18.67084	17.67198	37.44297	38.18094
gene-AT4G	2.805953	2.570834	3.132932	1.24977	1.158404	1.773527	2.798941	2.470438
gene-AT3G	0.231141	0.066519	0.110197	0.414202	0.713073	0.65722	1.111132	2.115029
gene-AT1G	1.373698	0.855731	1.32995	2.438622	1.927484	2.740035	9.963064	9.529788
gene-AT4G	18.61602	17.09095	17.24654	25.54284	28.03068	26.79567	18.11034	17.98532
gene-AT5G	25.42143	22.90762	24.46335	20.03344	19.97338	19.34766	9.016477	10.29185
gene-AT2G	35.40497	40.5067	36.82348	14.41412	13.18722	14.2689	6.739546	6.34395
gene-AT1G	42.07431	36.4207	37.97441	49.52363	50.08045	49.76554	19.90533	20.16569
gene-AT2G	15.07881	16.26038	16.99027	21.56046	20.48111	20.38893	10.85401	9.952187
gene-AT4G	26.05326	25.51964	22.10257	22.91014	21.06546	26.44686	22.44034	24.79095
gene-AT3G	3.038422	3.001468	3.262692	2.618065	2.206713	2.575653	0.544071	0.433303
gene-AT1G	4.141635	3.893619	3.996923	3.257385	3.965169	2.773108	7.351997	7.852694
gene-AT1G	12.72817	15.25203	12.63796	19.07492	17.1842	18.28462	3.473119	3.717093
gene-AT2G	43.82428	42.00203	42.97673	22.28442	23.1024	22.59443	10.16237	9.71119
gene-AT3G	36.00752	36.31273	36.70157	20.77612	22.93535	21.56479	66.32127	65.98768
gene-AT2G	3.650363	2.802998	2.574606	2.336508	2.184665	1.973225	3.253731	3.691869
gene-AT2G	13.45735	13.50501	11.64831	13.3334	12.05345	13.46512	14.48562	12.8844
gene-AT2G	69.70615	71.84325	74.88941	83.42799	86.1564	85.40101	90.85455	90.58957
gene-AT2G	2.484889	2.844172	3.564903	3.777511	2.723182	3.469652	15.57215	13.46873
gene-AT5G	40.71595	42.13099	42.89902	71.81104	75.61068	72.58458	14.31455	15.28251
gene-AT5G	50.03247	51.16383	53.02052	45.13344	44.78381	43.25963	85.73254	92.1663
gene-AT5G	1.837651	1.990518	1.809835	1.784733	1.957938	1.21964	3.273491	4.29259
gene-AT2G	1.857274	1.359126	1.588462	0.929344	1.176849	0.924349	1.810642	2.301286
gene-AT4G	0.216502	0.135608	0.321021	1.725323	1.264702	2.050453	0.179717	0.076681
gene-AT2G	78.47482	78.68155	75.78902	55.3648	57.46593	57.91621	25.97594	26.08898
gene-AT1G	15.90332	15.79026	15.81682	15.74106	15.45947	14.99417	1.783018	2.156626
gene-AT1G	7.839858	6.347659	7.858739	2.465858	1.804405	1.85331	16.24665	15.00343
gene-AT2G	0.816958	0.841986	0.654292	1.728886	3.055193	1.712289	1.404475	2.790321
gene-AT2G	2.339399	2.493611	2.405401	1.441868	1.57128	1.443532	2.444648	2.561462
gene-AT5G	0.594651	0.658094	0.384872	1.100014	1.556989	1.502308	0.346652	0.354324
gene-AT4G	4.194774	4.429803	4.490737	6.029919	5.975991	5.936264	3.589571	2.859088
gene-AT5G	7.320452	7.039977	7.915662	10.65502	7.959759	8.775398	1.821689	1.840093
gene-AT3G	10.85851	10.37636	11.90208	30.85675	26.70193	25.21599	5.904886	6.880564
gene-AT2G	51.65539	48.67643	51.56215	41.10651	41.42882	42.94098	87.39896	97.05608

gene-AT2G	27.46589	26.38326	28.39838	26.68499	27.4117	27.36696	17.5543	18.73903
gene-AT5G	0.59661	1.139905	1.136372	0.640803	0.361123	0.467431	1.044257	1.182223
gene-AT5G	14.04328	15.82971	14.38839	13.1346	14.12352	13.86801	6.359438	6.300122
gene-AT5G	14.2555	12.7557	14.33071	10.14696	8.934272	9.757252	3.067029	3.768848
gene-AT4G	13.11891	13.08174	12.29834	5.541761	4.724149	4.69892	30.67723	30.09414
gene-AT1G	3.784163	3.116817	3.179082	1.946263	2.55086	2.659262	2.889841	4.113361
gene-AT4G	6.268249	6.047273	6.441272	3.799823	3.811483	3.76284	6.57995	7.29791
gene-AT5G	110.993	104.7279	111.7711	49.97875	47.09604	47.41253	102.7406	108.4516
gene-AT2G	131.4709	127.6645	135.0152	80.07467	75.21523	76.31004	54.76667	51.47558
gene-AT1G	2.089912	2.04002	2.208261	2.163993	2.049163	2.16159	2.636556	2.624023
gene-AT1G	1.073492	0.967186	0.946576	0.426024	0.552061	0.405815	0.510676	0.627074
gene-AT1G	1.577204	1.320193	1.204977	7.65611	8.353778	7.868818	1.845102	1.709052
gene-AT5G	2.015291	1.508038	1.438194	1.436442	1.859919	1.127288	1.306987	1.800101
gene-AT4G	126.6708	124.5849	120.8972	23.26182	25.54227	24.85074	8.467166	9.525462
gene-AT3G	66.66533	68.75584	66.76238	44.18115	45.02168	44.98699	27.93512	30.13988
gene-AT5G	1.898801	2.190206	1.710658	1.578019	1.386813	1.677775	1.096221	0.798523
gene-AT5G	0.576662	0.72722	0.613453	0.1176	0.123185	0.264489	0.302929	0.238472
gene-AT5G	6.828063	10.50009	8.965386	4.64025	4.681625	3.850703	7.536398	8.069187
gene-AT1G	6.810717	6.099645	7.167338	82.02398	79.71541	77.82015	4.905326	4.357438
gene-AT5G	11.88596	11.70259	12.25602	3.992474	4.587646	3.445567	5.087958	3.953654
gene-AT1G	2.39512	1.758501	1.87811	1.516564	1.588193	1.529069	0.730178	1.528528
gene-AT5G	1.788008	1.002161	1.097166	1.307247	1.861324	1.590813	0.356486	0.305078
gene-AT3G	26.18615	20.41015	25.61372	21.74207	20.7789	24.66555	20.79812	19.91653
gene-AT3G	21.04881	21.24113	20.78129	27.88769	27.1952	27.83572	13.84913	15.66309
gene-AT1G	12.11291	9.533608	9.536562	9.071539	7.2514	8.309142	14.54292	14.78237
gene-AT3G	14.94003	15.72406	15.24458	11.90416	12.74308	11.2102	12.41312	11.27394
gene-AT2G	0.751131	1.396207	0.772317	0.340524	0.567617	0.571659	0.454927	0.270902
gene-AT5G	15.41781	14.37446	12.81969	13.12026	11.86801	12.35081	19.74972	21.66017
gene-AT1G	3.539012	1.610489	2.296702	2.04505	1.606847	1.530446	1.186789	1.123723
gene-AT5G	4.579748	5.033725	5.183677	7.417937	7.236747	7.328125	7.447631	7.204317
gene-AT4G	4.812006	3.675076	3.444419	5.669042	6.475092	6.001861	1.888989	1.474871
gene-AT1G	8.631898	9.73058	7.92841	4.429794	3.928114	3.835085	8.018563	9.191567
gene-AT3G	5.580659	5.481649	5.063406	4.67489	3.882127	4.168849	6.067574	6.746562
gene-AT5G	2.255194	2.241221	3.085647	4.126739	5.204116	4.878732	3.181054	2.632391
gene-AT4G	5.498642	5.494679	5.25541	2.599532	3.707037	3.373272	2.079512	2.265899
gene-AT3G	23.9082	22.53543	21.86493	10.57902	12.34491	10.39712	18.87903	17.92286
gene-AT1G	250.601	252.0268	255.8392	568.679	557.0402	558.9466	195.153	192.8742
gene-AT3G	0.649011	0.603449	0.846299	3.319053	3.364859	3.424229	0.982011	1.596317
gene-AT1G	23.97068	24.31262	21.94123	41.45123	39.43733	41.77049	61.09903	63.77213
gene-AT2G	22.17785	22.59572	20.68123	6.827806	6.289672	7.451245	4.687202	6.320155
gene-AT2G	2.226025	2.095789	2.389565	1.787942	1.657048	2.332787	3.509339	2.865558
gene-AT5G	59.66742	53.00141	53.98917	46.28412	44.79161	47.29177	83.19965	73.77406
gene-AT4G	25.65331	25.30735	24.83127	12.47867	12.50455	12.75534	10.39848	11.37339
gene-AT2G	13.95917	13.89095	15.68873	2.454437	2.742348	2.60989	24.5511	23.32362
gene-AT1G	20.339	19.44846	19.81234	11.57374	11.69979	12.30662	18.46591	19.12333
gene-AT2G	9.547165	8.933185	9.460121	9.919818	7.574339	8.498017	10.20226	11.92205
gene-AT4G	5.50305	5.725559	6.130658	5.548976	4.412728	4.978645	7.325858	7.223853
gene-AT3G	0.806125	0.519108	0.923647	2.018693	2.136863	2.108314	0.706724	0.477895
gene-AT3G	8.723561	12.43294	9.863963	13.92007	14.30869	12.58791	7.421433	8.138559
gene-AT4G	18.54497	21.05917	20.84067	15.39509	16.38818	14.01163	28.16858	28.4897
gene-AT3G	202.4806	195.6892	192.5938	156.3709	149.7051	155.1268	345.6573	345.3178

gene-AT2G	486.8487	504.6586	490.3378	287.7987	297.0273	297.1335	511.4841	514.1794
gene-AT1G	0	0	0	2.779858	3.41004	3.059069	0	0
gene-AT1G	323.4728	329.2311	331.2281	166.9647	165.1837	161.5467	325.8629	368.2851
gene-AT4G	7.860702	7.653942	9.113067	11.84677	11.98028	11.26963	10.40066	12.75314
gene-AT1G	104.8718	104.3227	98.89188	108.8386	111.5935	107.8398	150.0131	152.5313
gene-AT4G	66.44613	67.08328	69.52984	22.95031	23.85862	24.47234	5.504752	6.730219
gene-AT4G	11.14844	9.504125	9.677442	6.083667	5.841701	5.922493	19.54494	18.65951
gene-AT1G	1.095838	1.13113	0.878748	1.064181	1.414044	1.280918	0.237341	0.231011
gene-AT2G	31.96037	32.48126	30.58181	84.6154	89.60526	89.56617	15.38915	13.64764
gene-AT3G	14.03653	14.71108	13.21432	31.26434	29.97729	27.83241	18.74452	19.27795
gene-AT2G	0.37633	0.441068	0.320717	0.397559	0.337427	0.256697	0.681725	0.440362
gene-AT4G	2.816988	3.032235	2.018577	1.739936	1.635129	1.937502	0.79019	0.672613
gene-AT3G	2.077738	2.908697	2.184832	1.55103	1.363437	1.585356	2.496941	3.209187
gene-AT5G	5.156306	4.446056	5.078126	2.047542	1.560113	1.747308	4.909899	5.407246
gene-AT5G	1.901412	1.52163	1.596158	1.703554	3.046577	3.109832	0.473147	1.037138
gene-AT2G	18.86072	19.1349	17.88793	14.20849	13.3552	14.42402	4.072669	3.714054
gene-AT4G	45.90744	45.54287	43.48405	22.95865	23.3102	25.76639	71.0974	75.89195
gene-AT3G	2.233777	1.746484	1.796879	3.087573	2.808631	2.520594	0.931802	0.833424
gene-AT2G	35.16359	33.71491	36.54714	27.5271	25.45741	24.62757	26.46233	25.85038
gene-AT4G	34.73252	33.59222	39.51216	20.62197	18.21528	22.08308	62.07331	61.45787
gene-AT5G	6.140587	4.484601	3.752541	1.463104	1.400074	2.402383	1.360168	2.317295
gene-AT5G	188.5316	184.2844	187.0196	59.5284	56.04855	55.15339	299.2838	306.8987
Arabidopsi	1.109902	1.183867	1.751703	0.334905	0.64215	0.698869	1.509207	1.179819
gene-AT2G	16.65673	16.34722	16.28663	14.49116	12.85363	14.38931	18.78315	20.20447
gene-AT1G	15.12978	12.03234	12.75681	13.14002	11.90522	12.08739	16.93171	18.90215
gene-AT1G	3.144183	3.030616	3.452054	5.44839	7.179812	5.718787	5.073962	5.371936
gene-AT5G	2.853623	2.504781	2.841692	3.282957	3.52467	4.034392	4.61371	4.787603
gene-AT4G	118.1853	114.2015	110.6722	72.97477	73.6488	71.46028	124.6477	120.3446
gene-AT5G	1.592526	2.231442	2.259319	1.070192	1.831073	1.319331	0.535639	0.289427
gene-AT3G	22.42987	22.82538	25.26558	38.15005	40.34722	37.46334	2.497069	2.98994
gene-AT4G	25.85012	26.21646	24.60307	39.20918	42.45155	39.10497	13.09657	15.03105
gene-AT1G	52.11104	53.31603	49.74387	26.55872	31.70707	26.17533	110.0435	117.2271
gene-AT3G	1.573283	1.816147	1.361315	0.698768	0.835374	0.370216	0.831115	0.450401
gene-AT1G	7.472223	7.156719	7.626026	6.880243	7.947882	7.370499	11.7186	13.1439
gene-AT5G	3.841164	4.157549	5.015267	25.62303	23.43106	25.15102	24.95635	23.64042
gene-AT1G	2.363476	2.129388	2.446878	8.076636	7.527936	9.300092	0.90249	0.956192
gene-AT4G	4.111474	4.867227	5.553669	7.420231	8.676316	8.227556	5.232336	6.106188
gene-AT3G	11.09865	13.48423	11.2045	11.42572	8.60717	10.73322	19.97076	15.21797
gene-AT5G	5.36272	6.002793	5.83298	4.01867	4.866117	4.831274	9.699729	9.441188
gene-AT1G	4.248587	4.345943	5.12326	1.59953	1.958245	2.029796	16.78206	17.55146
gene-AT2G	6.260577	4.46831	5.731482	24.67665	26.25706	28.92304	1.525311	1.215853
gene-AT4G	74.71053	79.74633	79.81768	25.66153	25.0395	25.06873	19.98676	19.73558
gene-AT4G	1.01472	1.470593	0.785309	11.89205	11.31501	13.28879	0.138512	0.075742
gene-AT4G	2.388817	1.683847	2.253797	9.013153	10.02772	9.037465	2.456983	1.45882
gene-AT5G	250.7728	252.5476	266.0401	210.7546	214.065	210.6309	388.2112	401.0577
gene-AT4G	11.34173	10.05886	11.16167	11.82127	10.52479	10.69912	16.14544	17.42094
gene-AT4G	6.46474	6.58036	6.027769	12.27816	12.80117	12.53228	4.840546	5.26098
gene-AT1G	2.211968	1.69571	2.616861	2.548739	2.759499	2.737919	2.225499	2.298097
gene-AT3G	6.316603	6.20075	5.480138	6.86385	6.918749	5.658648	0.620155	0.250534
gene-AT2G	0.294932	0.595961	1.033978	0.526993	0.26973	0.1587	0.135517	0
gene-AT1G	68.89299	66.65496	69.70839	18.17261	17.38535	18.39943	4.247421	4.879215

gene-AT5G	3.530168	3.051427	3.697926	3.140755	3.456782	3.476028	4.13751	4.063075
gene-AT4G	5.177982	6.166877	5.650045	8.290138	8.293538	7.549715	7.369969	7.62914
gene-AT4G	0.262593	0.263887	0.133615	0.602759	0.843595	0.644257	0.433472	0.620065
gene-AT3G	47.72594	46.79038	48.33335	42.26926	42.87826	42.75757	69.60742	68.79552
gene-AT2G	1.21977	1.526939	1.860962	0.197758	0.229595	0.154506	0	0.078256
gene-AT5G	6.035018	6.632192	6.320299	7.893302	7.705124	8.863069	9.529737	8.709586
gene-AT1G	21.43486	18.21127	21.313	12.57411	11.70278	10.5801	37.77057	38.28431
Arabidopsi	1.871784	2.125191	1.850755	2.155624	2.28613	2.126138	7.0822	6.747365
gene-AT1G	5.094067	5.128421	5.44401	12.47541	13.01986	13.72329	4.602223	4.872693
gene-AT5G	33.38125	32.32384	32.90313	21.19168	20.46159	22.14157	47.03738	53.83567
gene-AT2G	3.793751	3.801603	3.803607	4.186147	4.361149	4.052878	0.78699	0.691346
gene-AT5G	7.914548	8.913617	9.476441	10.8863	12.51939	11.28902	8.470841	8.214351
gene-AT5G	31.67942	31.00763	33.70773	32.75648	32.50646	31.65677	17.31539	17.44126
gene-AT3G	8.144468	9.061061	9.284727	8.1269	9.493605	8.159341	3.795571	4.238265
gene-AT5G	1.091008	0.984022	1.477363	1.011814	1.121698	1.621005	0.451841	0.462514
gene-AT1G	11.84822	11.091	11.08697	7.904325	7.49862	7.174595	2.321298	2.362949
gene-AT4G	5.283018	5.106333	5.167479	98.87278	96.858	98.28633	12.26152	9.903623
Arabidopsi	5.90163	6.215499	6.42776	8.071852	7.783888	10.42237	4.972008	4.077753
gene-AT2G	4.979028	5.003747	4.67207	8.189206	9.532787	8.806257	2.746587	3.842818
gene-AT1G	0.93537	0.499918	0.442232	1.375917	1.917065	1.842692	0.220301	0.311353
gene-AT3G	121.1484	127.65	133.5195	93.16637	93.48879	93.93994	196.4723	212.123
gene-AT1G	111.0375	106.9968	112.1615	70.41119	69.20375	67.07491	201.7659	206.3218
gene-AT4G	5.372088	5.625555	6.594211	5.084756	4.165809	5.318105	2.718334	2.113004
gene-AT4G	282.8201	279.5877	288.033	263.9877	272.8908	269.1577	426.481	432.4069
gene-AT1G	7.274424	6.735817	7.426498	8.431539	8.388984	8.208851	6.239621	6.03882
gene-AT5G	1.798363	1.828354	1.981298	0.717583	0.473018	0.385986	4.027767	3.395159
gene-AT3G	2.350157	3.164001	2.546333	1.766844	1.279435	2.007435	1.017824	1.966958
gene-AT5G	5.696599	5.3642	5.71769	9.075365	9.363498	10.13811	3.184225	2.61762
gene-AT2G	13.50635	11.46153	12.34175	8.843963	8.984481	9.846888	16.85495	18.9335
gene-AT2G	1.811164	1.479636	1.517963	0.852743	0.565766	0.748955	1.066804	0.790767
gene-AT4G	18.88967	17.28778	18.24324	40.40739	39.23617	39.05961	5.368738	3.580604
gene-AT4G	9.615475	9.718998	10.20377	7.525537	6.419964	7.157238	14.68066	17.3647
gene-AT5G	2.386662	3.194095	2.807903	3.416495	3.311403	4.121852	9.963635	8.523088
gene-AT4G	0.221524	0.128954	0.074734	0.631296	0.800158	0.479236	0.305854	0.166529
gene-AT5G	7.470809	9.383261	8.439589	8.531563	7.106174	6.082716	3.153316	3.181622
gene-AT1G	3.453971	2.75629	2.107042	3.024388	3.700559	4.086438	5.418639	6.121842
gene-AT4G	4.353661	3.212215	4.035781	3.959737	4.779283	4.471865	2.195464	2.711937
gene-AT1G	10.65901	10.25694	11.017	5.576982	4.097818	5.025438	28.3559	28.36324
gene-AT1G	19.01243	20.70877	19.39999	25.43043	24.06218	24.1073	11.1943	8.402905
gene-AT3G	15.36785	15.47554	14.92953	13.68494	12.55057	13.80184	20.21039	21.08367
gene-AT3G	7.823161	9.201354	7.662362	1.653041	1.361246	1.502401	2.97028	2.377705
gene-AT3G	64.33144	63.47558	63.118	72.5682	74.02844	77.57704	41.78951	44.17701
gene-AT3G	0.637916	1.408761	0.945217	2.208311	2.651297	2.845339	0.456559	0.847798
gene-AT3G	23.1909	22.61431	22.70368	9.794649	10.0526	10.66234	14.77297	13.6779
gene-AT1G	1.698799	1.705902	1.592952	1.898552	1.125858	1.47705	3.293831	3.285643
gene-AT4G	4.424666	3.662348	4.010057	3.76767	2.634575	2.414815	4.239787	4.843029
gene-AT3G	2.318458	2.158312	2.317537	4.181225	4.079242	2.876874	1.702704	1.512512
gene-AT1G	0.704047	1.125358	1.559511	1.477762	0.656668	1.109992	0.415987	0.288504
gene-AT1G	26.04661	26.13106	24.71585	27.98838	26.15507	28.81964	27.8343	23.58163
gene-AT2G	28.83634	30.56474	27.67822	36.37134	38.59996	35.90685	48.64288	49.90602
gene-AT5G	1.594621	1.144442	2.066407	1.862806	1.357675	1.311196	0.870635	0.894395

gene-AT2G	8.190804	9.647985	8.489029	2.35605	2.221891	2.424138	27.82587	28.15549
gene-AT1G	458.2351	446.7782	458.2618	751.6603	751.0892	747.7997	149.2495	156.496
gene-AT1G	28.31691	23.37748	28.75877	23.5617	21.43227	22.52824	15.66027	17.46577
gene-AT3G	136.2718	148.3766	145.2451	101.7706	99.61176	99.67873	42.90195	38.94584
gene-AT5G	0.203044	0.167917	0.247538	0.024097	0.096849	0.143311	0.223882	0.364798
gene-AT4G	29.02525	23.26177	26.4001	26.46166	29.00161	28.66705	2.53734	2.944127
gene-AT3G	4.415587	6.367399	6.575173	4.924529	5.875652	3.212322	4.20341	4.359051
gene-AT1G	4.646779	2.713994	3.905925	11.89519	10.46886	9.047571	3.420067	4.38388
gene-AT4G	1.135175	1.367592	1.256646	1.349443	1.717446	1.249415	0.326977	0.262998
gene-AT1G	14.26827	13.10549	13.67926	6.621777	6.740955	6.970368	4.097834	5.063425
gene-AT4G	40.89679	40.63752	40.89056	27.63712	28.26045	30.15242	68.44115	67.2369
gene-AT5G	0.574849	0.851083	1.016951	0.519798	0.617621	0.742122	5.406491	7.31106
gene-AT1G	1.684069	2.504798	3.756913	4.588347	4.455461	4.753729	1.476165	1.471193
gene-AT4G	8.271505	7.592609	9.892562	9.417098	9.361233	9.402946	5.809024	6.62715
gene-AT5G	16.28957	13.39392	14.9078	11.40212	11.91159	12.06798	18.89052	18.53599
gene-AT1G	7.245252	7.157885	7.069324	3.95889	4.688119	3.893968	5.676225	4.754974
gene-AT2G	26.3036	27.73931	25.79502	163.8876	155.4524	144.1614	15.02345	16.33337
gene-AT2G	2.771703	3.092646	3.391381	3.557552	4.203555	3.238626	1.060976	1.54373
gene-AT1G	4.135508	3.991421	3.115787	3.169571	3.147265	3.721628	3.696731	3.320852
gene-AT5G	2.837723	1.909971	2.484248	5.467309	5.671479	6.45442	2.771129	3.84445
gene-AT5G	1.978858	1.835231	1.479791	7.474375	7.699846	7.896007	0.790085	0.707115
gene-AT3G	13.4384	13.59511	14.31847	7.101212	7.160425	7.984362	16.93767	18.34048
gene-AT2G	12.28885	10.09591	10.0598	7.830961	9.16591	8.744765	4.749249	3.465477
gene-AT4G	6.928689	5.823987	4.651663	1.50797	1.711803	0.495744	3.240859	8.062498
gene-AT4G	622.8212	640.2164	631.8339	355.8669	389.7267	383.4058	1063.435	1082.409
gene-AT4G	3.283411	4.105579	4.860919	7.899558	9.98944	10.44513	6.763726	6.048541
gene-AT4G	1.712861	1.721695	1.902982	1.500658	1.938383	1.802911	1.398787	1.177606
gene-AT1G	4.52833	4.366778	3.870414	4.124713	4.133862	4.486451	2.07083	1.573106
gene-AT3G	33.22624	33.97479	37.52228	59.97509	57.75672	59.48813	15.329	16.32093
gene-AT1G	7.682538	8.653915	8.20437	20.00227	18.26451	17.10053	11.60791	12.31467
gene-AT2G	8.844877	10.03686	9.694326	5.438433	5.601943	4.501744	16.74282	16.9432
gene-AT5G	6.872358	6.640266	6.100419	7.719547	6.064911	6.327814	9.963552	10.298
Arabidopsi	0.541319	0.680144	0.98937	1.314156	1.466085	1.185846	0.545274	0.949532
gene-AT1G	358.4529	358.2474	366.8236	268.0254	269.0735	254.4359	534.9987	554.9094
gene-AT4G	31.11247	29.24152	32.5561	25.43303	20.93867	20.96751	41.7952	39.79049
gene-AT5G	2.761978	3.330864	2.694345	1.413	1.804524	1.060781	2.634359	3.576472
gene-AT5G	0.594281	0.623733	0.582368	1.120864	0.836755	1.165763	0.360617	0.33695
gene-AT4G	1.786637	1.959597	1.725802	4.334448	5.144269	3.510516	2.395372	1.732349
gene-AT4G	2.118342	4.953482	3.825081	1.798444	2.17057	1.665336	7.096878	7.450876
gene-AT1G	213.7794	218.0533	224.1155	141.1928	135.9641	141.4732	247.7027	266.7157
gene-AT2G	1.398285	0.825422	0.71582	2.70235	1.629642	1.764915	0.361412	0.95874
gene-AT4G	12.01938	11.23928	11.80524	6.496417	6.277958	6.539624	4.928836	5.107484
gene-AT1G	64.92559	61.10666	66.56719	58.30662	61.51929	56.7272	16.83311	19.69535
gene-AT5G	7.067444	5.513059	5.774679	8.912262	10.04796	10.20382	7.239983	6.228504
gene-AT4G	6.377855	6.65665	5.900602	7.552157	8.595538	7.360635	5.275912	5.039872
gene-AT3G	0.571107	0.585881	0.504961	0.553821	0.490279	0.667889	0.303274	0.235848
gene-AT1G	19.59906	17.84725	19.657	20.3158	18.90583	20.89888	9.884137	8.041697
gene-AT4G	13.74159	13.6439	13.56319	8.847557	9.807501	9.473601	19.68947	20.85018
gene-AT1G	7.434617	6.244826	6.878265	4.506966	2.750069	3.425373	7.248491	8.28121
gene-AT3G	8.514397	10.41556	9.729172	10.28508	9.806995	9.84369	14.60633	14.02055
gene-AT3G	28.23727	27.54331	29.75906	21.68763	22.8814	21.74353	27.69194	28.20733

gene-AT5G	7.144545	7.800162	7.190987	9.707823	10.77173	10.29338	4.444338	5.958603
gene-AT4G	1.009344	1.222013	1.043185	0.553746	0.27174	0.525391	0.182471	0
gene-AT3G	18.41722	15.1965	17.49356	22.44789	20.31593	20.54687	7.84218	8.275328
gene-AT3G	10.85882	10.78653	12.38416	7.036973	7.224443	8.226695	5.865643	6.215029
gene-AT2G	8.540883	8.298031	8.748816	10.42882	10.1229	10.94224	2.09832	2.733562
gene-AT5G	1.032507	1.395178	0.309954	2.810185	1.862206	2.033597	0.035749	0
gene-AT4G	7.817762	9.705667	6.567931	2.662873	3.171519	2.852937	13.17057	8.448701
gene-AT4G	3.037124	2.727307	3.398577	3.198283	4.679832	3.698714	10.02266	11.47773
gene-AT4G	41.22911	38.38759	36.47279	27.65377	32.18364	26.83942	11.80174	15.12921
gene-AT4G	1.217107	1.117023	0.82162	2.03765	1.629027	1.383946	1.240088	1.057335
gene-AT4G	2.377515	4.516411	2.111904	7.393082	5.031875	6.82281	0.477671	0.458399
gene-AT4G	168.7485	163.8059	165.9871	214.4858	205.2324	221.4674	43.83718	41.44075
gene-AT3G	1.502475	1.434011	1.06335	2.422778	2.061403	2.991937	0.933742	0.531088
gene-AT4G	0.818493	0.803301	0.310282	1.490832	2.393031	2.228404	0.309185	0.624907
gene-AT3G	12.95628	14.37203	15.04886	13.53951	10.2427	13.56452	20.65131	16.38725
gene-AT3G	1.785912	1.639294	1.134243	1.69992	1.202317	1.629954	1.600411	1.699932
gene-AT5G	6.008147	6.230202	5.17454	4.70816	5.294181	4.026188	11.82012	9.968648
gene-AT1G	0.935838	0.799966	0.693203	0.099272	0.198025	0.206044	0.078537	0.098664
gene-AT2G	1.848524	2.390628	2.293083	4.182813	3.765469	4.206744	1.322099	1.765679
gene-AT5G	14.85819	15.85046	16.56338	11.83118	11.05242	11.3799	23.15506	25.43801
gene-AT3G	10.4076	9.269794	9.001162	1.513065	1.001947	0.990539	10.13166	11.17474
gene-AT4G	2.28661	1.449126	2.355041	2.033661	2.535663	1.73703	2.692827	2.663613
gene-AT1G	8.865882	7.474808	7.623313	5.277213	4.611564	5.134411	2.175502	3.043152
gene-AT3G	2.236772	2.263507	2.120321	4.522082	3.929349	5.412301	4.332966	4.003025
gene-AT1G	6.218513	4.261085	4.501073	11.59019	11.13552	10.1765	6.225038	6.041478
gene-AT1G	4.23518	4.259611	4.687488	6.173196	5.27355	5.992128	2.969811	3.627186
gene-AT3G	22.51613	23.53504	25.68885	32.36472	32.62726	31.15336	44.32797	45.4012
gene-AT2G	82.10704	78.5423	80.683	26.03534	27.38654	26.27254	50.73264	62.0279
gene-AT5G	2.560848	3.028028	2.527242	2.901547	3.12053	2.969573	0.907826	0.931361
gene-AT3G	48.25798	47.11425	50.51275	24.18513	22.42719	22.69835	11.83081	12.03351
gene-AT2G	60.07057	58.98782	57.48765	44.63387	43.21164	43.85214	93.21395	88.89791
gene-AT4G	12.25487	11.36329	11.03994	10.20919	12.18657	10.38724	15.44116	18.69039
gene-AT5G	4.620623	4.223044	4.574829	3.26832	2.957156	3.932587	6.968391	7.311234
gene-AT1G	51.2737	48.97441	51.2757	46.39005	46.13219	46.78725	21.24295	21.71854
gene-AT2G	3.33732	3.71598	3.772607	4.95424	5.906963	5.486635	2.686635	2.641336
gene-AT1G	5.367619	3.997136	3.517849	7.573589	7.819316	7.244094	2.330022	2.332876
gene-AT1G	5.845741	6.145431	5.518935	4.192914	3.698782	4.896982	10.72643	10.77638
gene-AT2G	233.0298	223.9744	236.1328	180.9932	185.002	182.1247	326.5905	325.8627
gene-AT1G	5.79281	5.954589	5.692273	2.255334	2.018328	2.268905	0.628048	0.585453
gene-AT2G	31.84466	33.27559	32.70589	23.24156	21.47128	23.57278	43.80258	45.50962
gene-AT1G	21.85192	21.41581	16.34007	81.05843	76.50501	81.52277	13.07654	10.85276
gene-AT1G	37.70637	34.46536	34.69556	36.60394	35.73259	37.31815	9.387744	10.21951
gene-AT2G	0.891641	0.648996	0.944172	0.372598	0.415343	0.444186	1.736371	1.713498
gene-AT2G	0.103785	0.498246	0.107654	0.567541	0.430562	0.279055	0.209331	0.190534
gene-AT1G	188.477	175.3668	184.5479	200.3138	194.2678	197.8404	281.9019	277.6225
gene-AT1G	3.098948	2.939024	2.917403	6.315766	6.957201	7.110266	0.658341	0.832181
gene-AT2G	0.759377	0.865844	0.602865	0.792997	0.885797	0.850393	0.161757	0.037101
gene-AT5G	28.71629	28.99854	31.0737	16.89117	15.8814	15.62796	2.786984	3.484008
gene-AT2G	0	0.067692	0	0	0.053698	0	0.590882	0.67136
gene-AT2G	29.95431	34.39716	30.39257	60.98535	65.39481	63.85997	10.41157	9.806696
gene-AT2G	93.82856	94.83711	94.36205	29.5961	26.26535	28.05196	35.33756	34.58159

gene-AT1G	3.467162	3.765955	3.825422	3.293971	3.80882	3.042755	11.59607	11.26175
gene-AT2G	19.85843	18.46491	20.89441	35.57116	35.45206	39.66097	13.67815	11.92175
gene-AT2G	16.2307	15.02367	16.47828	8.17504	8.739504	8.991155	26.43274	25.4351
gene-AT1G	2.064727	1.478572	1.108802	3.555649	3.72449	3.647604	1.223688	2.140593
gene-AT5G	3.283951	2.539234	2.92765	6.809247	7.903676	6.254153	1.9476	1.514666
Arabidopsi	0.794311	1.230976	1.402257	0.027984	0	0	0.723799	0.541113
gene-AT1G	0.822226	0.885602	1.120145	2.419093	1.996612	1.743268	2.58903	2.82174
gene-AT4G	7.517337	6.053611	7.478913	6.738375	7.790586	6.63936	10.46118	10.68596
gene-AT5G	176.3972	170.268	177.0697	196.5484	196.7449	199.7496	64.50772	67.28746
gene-AT4G	13.34348	13.57804	13.22261	10.25494	10.68588	11.76521	2.998411	3.290214
gene-AT4G	5.594442	5.433386	5.815259	12.79385	12.36586	12.12249	8.316771	7.525833
gene-AT1G	3.696143	4.245108	3.483598	4.975021	5.227212	4.490936	1.564935	2.031933
gene-AT5G	10.24955	10.11593	10.33876	7.247365	6.428988	5.456531	7.769338	8.0166
gene-AT5G	1.43634	1.326054	1.608323	0.411478	1.18512	1.294444	1.813381	1.895185
gene-AT2G	3.772552	3.741246	2.61944	2.956802	3.506484	3.749248	3.880361	4.468349
gene-AT2G	2.500396	2.934966	2.575531	5.203524	4.779137	4.643587	5.05229	5.749812
gene-AT5G	15.20116	14.09186	13.47652	10.71105	11.03328	11.09562	15.61633	17.07439
gene-AT5G	2.719487	2.280352	3.625631	2.561533	1.665707	2.249043	3.169166	3.393054
gene-AT5G	159.8438	152.8228	156.8804	123.7255	128.652	123.4028	173.1072	178.9309
gene-AT1G	2.946711	2.880455	2.392413	0.889475	0.189143	0.369555	3.551116	2.349592
gene-AT3G	3.542274	4.01152	3.55734	5.572433	5.417047	5.036011	2.825199	3.032893
gene-AT3G	9.7182	9.005819	9.521161	8.526007	8.56137	7.736502	11.231	10.62319
gene-AT5G	17.16448	19.26835	17.3616	16.82478	17.61545	15.30698	7.743368	8.776134
gene-AT1G	0.175851	0.984503	0.185696	0.085526	0.068673	0.381975	0.474227	0.584786
gene-AT3G	19.13612	22.15379	19.93057	13.99595	16.16375	15.49022	14.36276	15.62545
gene-AT2G	1.321228	0.946643	1.137582	0.603848	0.416002	0.309332	0.376141	0.511048
gene-AT2G	40.53957	39.62243	40.46793	36.06447	32.1896	31.97506	44.15329	46.70551
gene-AT3G	1.965655	2.434731	2.257322	2.340589	2.449963	2.323566	4.94953	4.823991
gene-AT5G	0.847422	0.741954	0.599637	1.125351	0.845499	0.997079	0.307513	0.314233
gene-AT5G	0.037397	0.074112	0.237701	0.029656	0.074498	0.022512	1.234914	0.977211
gene-AT1G	5.587208	5.61987	5.382484	3.362241	3.23908	4.099429	5.322916	4.906001
gene-AT1G	87.0774	88.85642	92.71472	34.05225	31.9095	32.36671	29.20861	33.53239
gene-AT1G	4.134287	5.041272	4.270941	2.972536	2.59313	2.946204	3.057524	2.698323
gene-AT1G	445.1768	430.9413	440.489	985.631	957.6326	971.6454	316.5109	317.3391
gene-AT1G	3.104632	4.056136	3.113933	1.786749	0.915237	0.923008	4.441849	4.702384
gene-AT1G	2.084898	2.993603	2.048927	1.233045	1.170891	0.797787	0.873017	0.934573
gene-AT5G	1.516002	0.847185	2.055956	1.289863	1.047657	2.141018	4.666669	3.895711
gene-AT1G	0.899764	0.83301	0.776393	0.942372	0.504947	0.605005	0.113515	0.176405
gene-AT4G	8.181252	6.419017	6.745295	7.969815	8.397079	7.935984	3.661638	2.98665
gene-AT3G	0.873014	0.7204	1.700516	0.724991	0.592205	0.846114	1.478304	1.093402
gene-AT1G	1.070262	1.260118	1.173501	0.140568	0.265311	0.182477	0.605716	0.647737
gene-AT3G	4.070027	4.81729	3.314959	3.399761	2.688957	2.138295	4.271276	5.040558
gene-AT4G	66.15832	65.7333	63.50065	33.92686	34.52608	34.29285	29.51906	32.24602
gene-AT3G	36.20098	39.42069	38.50736	25.71189	26.87993	26.30725	52.94597	51.88723
gene-AT3G	18.24974	16.4353	16.30975	21.1422	19.94641	22.24992	46.39919	46.34517
gene-AT3G	42.8539	43.64272	42.16119	50.79619	52.28928	51.17275	77.54417	81.38963
gene-AT5G	6.020126	3.647046	5.163077	38.40849	36.49774	36.88893	0.595126	0.9696
gene-AT5G	2.691452	2.572191	2.527314	1.359242	0.761433	0.826706	3.061132	3.864331
gene-AT3G	4.563168	4.846808	5.329261	3.753743	4.476218	4.177675	5.071589	5.561901
gene-AT5G	4.406582	4.343911	5.342494	2.79468	2.886079	3.059107	1.721622	1.517247
gene-AT3G	1.679879	1.459354	0.980978	3.22363	2.94606	3.861945	0.93094	0.952179

gene-AT2G	6.466908	6.481484	6.019662	5.799829	7.534997	6.479714	6.969512	6.935371
gene-AT1G	15.74634	18.69975	16.63131	16.8079	16.3995	16.06034	9.015794	8.193801
gene-AT3G	415.1548	399.7083	408.2326	398.1898	388.1877	385.7686	554.2612	566.3421
gene-AT5G	1.648528	1.533919	2.168767	0.34167	0.191411	0.400874	16.57035	16.17674
gene-AT3G	0.592445	0.752111	0.899076	0.524112	0.461182	0.468513	1.306312	1.957704
gene-AT5G	11.80263	11.35663	10.95625	8.423361	8.695651	9.421293	13.24205	12.53453
gene-AT4G	8.344152	9.516745	8.496444	4.474306	4.738537	4.538152	3.99278	3.785737
gene-AT3G	1.474129	0.80844	1.005166	1.012651	0.785544	1.181764	1.237056	1.920138
gene-AT4G	0	0	0	0.484338	1.540108	1.064538	0	0
gene-AT2G	13.85323	13.23913	13.81859	23.55486	22.50885	22.57579	7.830224	8.248515
gene-AT4G	1.985089	1.753041	1.96476	0.679089	0.478059	0.550968	0.80188	1.035105
gene-AT2G	3.59493	4.225387	3.512539	4.637097	4.397889	3.38782	3.770084	4.238087
gene-AT2G	42.51808	43.62499	43.45909	71.21681	70.73487	74.13841	26.72636	26.45227
gene-AT3G	4.993789	6.126592	5.575706	5.44438	5.358849	4.989724	3.307645	2.719214
gene-AT5G	13.69659	12.83621	13.6159	6.548474	5.634732	6.239543	37.17566	39.28549
gene-AT5G	6.3127	7.471562	7.435747	10.2221	13.83366	10.3444	3.884989	3.29928
gene-AT5G	17.76481	17.44099	17.21755	14.84417	12.14578	12.37559	36.4752	40.54607
gene-AT4G	27.80333	33.39496	32.22015	77.51981	78.14097	66.60058	32.58349	44.09573
gene-AT1G	6.247694	7.622486	7.091761	7.580971	8.583973	8.842931	11.9603	11.94594
gene-AT3G	9.467068	10.01468	10.49516	4.463962	5.568263	4.502343	17.89072	18.74282
gene-AT1G	6.490771	7.637652	7.79484	0.598636	0.799685	1.197094	9.799952	9.918791
Arabidopsi	17.00545	17.90305	15.98075	11.19385	9.891441	11.28584	20.77778	16.49773
gene-AT1G	3.244368	3.625345	3.736564	5.003217	4.930113	5.728285	5.826069	4.624413
gene-AT1G	10.69533	10.78966	12.0775	11.74733	11.96021	12.24751	13.40325	13.47826
gene-AT5G	138.9887	141.8365	142.5919	112.5436	116.1411	117.7922	263.285	262.4432
gene-AT3G	3.670003	4.086669	3.416784	9.830582	11.23806	9.699633	1.99423	1.80259
gene-AT5G	19.41152	18.06722	21.19226	18.85332	20.0491	18.35636	6.729456	5.08121
Arabidopsi	3.6544	3.934282	3.248858	1.883516	1.384641	1.612503	3.931153	4.224306
gene-AT1G	31.51727	30.92916	32.71136	31.49149	32.65828	31.92098	49.31855	48.56574
Arabidopsi	9.132799	10.21668	11.8102	3.755518	4.704279	5.122048	2.576879	2.183942
gene-AT1G	1.274637	0.839136	1.895095	5.239217	7.34553	6.298374	0.326906	0.359239
gene-AT3G	1.025216	1.21396	0.924633	0.11374	0.144863	0.112259	0.927899	1.147798
gene-AT2G	15.00334	10.63082	13.33991	5.012596	5.448524	3.499204	3.694066	4.655969
gene-AT3G	134.4379	144.1755	136.3647	60.53059	60.85282	61.80856	125.3907	110.4782
gene-AT1G	2.023342	1.962936	1.711605	2.254271	1.575685	1.664884	1.520743	2.138164
gene-AT5G	12.92821	13.17375	12.95503	19.60714	19.84201	17.74859	10.21942	12.64529
gene-AT5G	69.45402	69.45349	74.26929	85.48003	83.33553	86.81078	28.78204	30.38077
gene-AT5G	3.157399	2.930531	4.485899	2.204773	2.444838	2.281378	0.993006	1.317082
gene-AT2G	186.2325	179.8904	187.712	171.3915	173.8786	182.6655	131.9831	130.3811
gene-AT1G	20.26767	22.68385	21.44719	34.43126	34.13303	32.30307	15.77288	17.46208
gene-AT2G	0.552546	0.477065	0.3736	1.473505	1.197052	1.137781	0.873712	0.854674
gene-AT3G	17.99097	17.47254	17.44281	23.59051	22.40424	23.93572	9.549763	9.349081
gene-AT2G	1.597143	1.361451	2.03724	1.764817	0.981673	1.203246	0.33707	0.459143
gene-AT5G	0.4653	0.59542	0.338504	2.419714	3.208174	2.893252	0.347542	0.369513
gene-AT5G	0.544857	0.523166	0.50192	0.157165	0.258346	0.241193	0.335669	0.609739
gene-AT1G	1.681928	1.497646	2.181928	1.29136	0.423917	0.881448	1.737825	1.981874
gene-AT2G	0.473761	0.284742	0.700331	1.341598	1.131727	1.435575	0.490521	0.422708
gene-AT3G	1.6995	1.792072	0.875781	3.056815	2.379232	4.288148	1.406179	1.98627
gene-AT5G	4.251308	4.009665	4.079594	1.734321	1.928374	1.685301	2.33257	2.466098
gene-AT1G	0.167177	0.167832	0.10558	0.181571	0.205018	0.149419	0.282193	0.043496
gene-AT5G	10.88754	11.13424	12.25677	17.10153	18.50793	17.9228	8.271492	9.656827

gene-AT5G	1.349871	1.095096	1.684003	1.095016	1.596593	1.834002	3.656831	3.381493
gene-AT1G	169.6076	171.2474	176.6479	102.1494	96.54483	89.21995	312.0752	323.5804
gene-AT2G	1.195278	0.619231	0.523723	2.659328	2.906656	2.491639	0.215302	0.475594
gene-AT1G	33.65359	30.10875	29.29209	35.51968	36.86293	36.79644	36.21834	38.21366
gene-AT1G	66.00046	63.84617	67.10381	41.52073	44.45665	45.37865	112.1078	108.9826
gene-AT1G	4.11021	3.308956	4.137216	5.020109	5.175699	6.720569	8.78698	8.51306
gene-AT1G	4.396026	4.259483	4.357162	0.23367	0.37734	0.263631	0.033412	0.069014
gene-AT1G	4.76627	5.488914	5.377612	6.612664	6.632215	7.401452	1.676593	2.423652
gene-AT2G	0.199345	0.097479	0.229341	0.628896	0.561385	0.57158	0.245251	0.27975
gene-AT1G	2.722265	2.445904	2.742803	2.603837	2.259308	2.708188	0.245297	0.231414
gene-AT1G	4.164057	3.286568	3.626179	4.244204	4.214323	4.219079	3.443858	3.554738
gene-AT3G	3.348191	2.802512	3.409667	1.50985	1.878171	2.236683	5.451135	5.64468
gene-AT4G	80.95054	76.85048	79.61454	51.81284	50.38429	51.57879	145.2952	150.5057
gene-AT3G	468.7184	467.9968	468.4949	390.3054	390.3909	383.3127	162.4161	173.7691
gene-AT2G	3.13483	2.742426	3.230357	6.329285	7.466006	6.325021	2.738765	2.503571
gene-AT1G	0.400481	0.516801	1.038674	2.30449	2.557774	2.418475	1.11571	0.813697
gene-AT3G	24.80135	32.61914	22.93741	63.90727	55.8848	63.10365	25.36676	26.09406
gene-AT4G	39.64875	37.94927	37.02619	27.05563	26.62369	26.6914	3.317944	2.289966
gene-AT3G	1.375181	1.683467	1.494893	1.849609	1.329243	1.57558	1.238718	1.102436
gene-AT1G	973.6938	948.7407	979.0559	508.6818	504.722	514.3302	1465.607	1479.082
gene-AT5G	5.812327	5.081198	5.893431	7.482066	6.850754	6.683289	8.083688	7.331299
gene-AT3G	1.592548	1.209808	0.735451	1.059217	1.373808	0.409799	0.989118	1.111328
gene-AT5G	1.633324	2.032389	1.863665	3.580167	4.382683	3.434522	1.126343	0.71554
gene-AT1G	1.596245	1.49431	1.748169	1.238734	0.896507	1.116482	2.171328	1.887593
gene-AT3G	6.378996	7.470324	5.575267	4.059258	3.871104	3.845459	1.561304	1.453697
gene-AT5G	0.209874	0.534605	0.274654	1.025532	0.885774	0.869701	0.234727	0.453782
gene-AT3G	0.479314	0.486381	0.633331	1.032137	0.851015	0.775413	1.318316	1.152202
gene-AT1G	10.59983	10.98787	11.14146	13.32951	13.2351	14.53183	4.860627	4.413666
gene-AT1G	29.65927	30.67188	28.02357	11.59556	16.40334	12.1539	13.61321	11.77608
gene-AT4G	3.239744	3.463988	3.116633	1.024651	0.945691	1.35552	2.189706	3.046739
gene-AT1G	11.69585	11.11782	10.44215	4.861361	4.083698	4.551991	20.55169	21.30781
gene-AT1G	1.426533	1.167494	1.170687	0.835714	1.015147	0.845675	0.491109	0.428079
gene-AT5G	28.2216	22.71119	19.41279	28.88015	29.23617	33.09408	11.24204	10.86551
gene-AT5G	161.9352	166.7113	163.4677	185.8718	189.7034	186.5527	39.20775	41.84509
gene-AT4G	31.85168	29.20672	25.252	30.52133	28.31475	33.15112	11.63563	11.43017
gene-AT5G	15.0111	18.34782	17.5735	13.5299	16.09294	14.15991	31.10638	34.01067
gene-AT5G	0.618307	0.998653	0.593781	1.383877	1.79428	2.30592	1.056105	0.825892
gene-AT4G	1.343238	1.233773	0.847442	0.996892	2.316529	1.521558	0.207713	0.526828
gene-AT4G	41.81568	37.74522	39.26759	32.56889	35.03808	33.24144	53.00228	53.1861
gene-AT3G	8.046	10.13762	10.19431	7.280523	6.118982	6.019732	15.54287	14.80927
gene-AT4G	13.42614	11.11673	10.24386	11.42927	12.55383	12.16473	4.963765	5.381361
gene-AT2G	4.255393	3.958128	3.569564	1.730289	2.365376	2.20638	1.386025	1.634469
gene-AT5G	9.044201	10.59189	10.53527	22.62028	24.16929	24.57293	8.623355	7.749855
gene-AT2G	1.552454	2.271206	1.582073	1.379858	1.623394	1.243108	1.239417	1.462656
gene-AT3G	1.142627	1.122676	1.24807	0.85113	0.444656	0.328811	3.028653	3.473997
gene-AT2G	5.466859	6.109047	6.873452	6.174033	5.381189	5.665988	11.28268	13.20236
gene-AT3G	3.589666	4.115014	3.278818	3.60503	3.989901	4.037154	4.25837	4.326303
gene-AT4G	3.784111	2.865013	4.558621	7.09796	7.101878	7.637899	8.44151	6.78455
gene-AT5G	100.3769	99.44634	100.2322	102.4296	98.72772	104.353	71.26267	73.11909
gene-AT2G	4.176521	3.672318	5.135166	5.569871	6.484998	6.736072	1.646874	1.94897
gene-AT4G	3.838726	3.687141	3.791954	2.022988	2.695654	1.933252	7.355618	9.443129

gene-AT1G	9.295728	9.751688	9.056006	7.672778	5.852081	7.036728	21.10569	22.17709
gene-AT4G	15.54456	15.23146	17.95355	6.077801	5.877729	5.240867	15.56548	17.93598
gene-AT1G	8.907212	8.990154	10.48623	6.866292	7.432262	6.634063	10.41738	10.8913
gene-AT5G	73.61517	78.45817	74.31714	28.1073	25.1009	27.81859	97.32585	89.6715
gene-AT3G	1.827929	1.343923	1.370439	0.73416	0.566862	0.873544	0.820319	0.249374
gene-AT2G	0	0.156777	0.091961	0.630833	0.703963	0.499566	0.898096	0.962036
gene-AT2G	7.284722	7.907005	7.165274	2.879448	3.869164	3.744683	6.182796	5.126879
gene-AT1G	22.08273	22.65411	22.44421	20.31753	21.67966	19.50244	26.62118	28.25651
gene-AT4G	106.6807	105.1658	108.0467	61.53245	61.47111	61.17031	103.5413	108.1939
gene-AT4G	35.05796	33.19468	33.92032	29.79588	29.19277	28.71248	46.1381	50.79302
gene-AT2G	26.67289	28.87682	27.29981	23.10757	20.90717	20.27137	44.95419	41.89666
gene-AT1G	22.77224	23.56894	24.28329	23.70714	23.82628	23.22617	13.75457	14.03005
gene-AT1G	1.574673	1.621181	1.657829	0.458142	0.839566	0.662235	3.281584	5.186144
gene-AT2G	4.953863	4.892131	5.26696	6.794347	6.92259	8.437451	2.397355	2.337153
gene-AT1G	12.14224	11.62384	12.09861	10.73399	11.17342	10.78958	17.24693	17.72057
gene-AT5G	7.576384	7.176273	6.419488	1.881435	2.13407	3.255246	1.265874	1.729499
gene-AT1G	14.63358	13.04558	12.45001	9.478144	10.19636	9.924548	26.25995	28.6209
gene-AT2G	0.588173	0.847984	0.54797	0.845383	0.541775	0.613049	1.28234	1.220663
gene-AT2G	55.84122	55.70587	58.6783	34.53313	34.99532	31.90754	101.3418	103.8199
gene-AT1G	2.205245	1.476327	1.604967	1.38818	0.89496	0.975095	1.996509	2.499302
gene-AT1G	6.985989	6.557075	6.102451	9.50468	8.433048	8.386424	3.968003	4.151097
gene-AT2G	3.269575	2.999894	3.399536	2.633598	3.045116	2.862975	0.445987	0.402683
gene-AT3G	29.22726	31.95752	37.54245	33.30496	31.73097	27.73582	41.2488	42.36538
gene-AT3G	7.082401	6.140295	6.087863	5.047737	5.401714	4.218052	11.0773	12.30483
gene-AT2G	0.21231	0.262736	0.311793	1.579637	1.215281	1.117871	0.360751	0.333679
gene-AT1G	14.26261	13.53979	14.35379	8.813721	8.124685	8.018848	14.87077	15.89961
Arabidopsi	2.160445	1.676519	1.861401	1.036703	1.113001	0.731126	0.310994	0.478515
gene-AT1G	65.73245	64.06804	67.32558	41.98807	39.88076	39.00084	25.41209	27.90428
gene-AT5G	20.43975	19.14817	20.13056	48.02253	51.88062	50.43451	27.41377	27.33922
gene-AT2G	0.946304	1.735847	1.059406	1.790062	2.431171	2.014364	0	0
gene-AT3G	4.696347	3.43123	3.86308	9.092832	10.22822	7.578119	4.9284	4.299495
gene-AT1G	0.93552	0.910248	0.678174	0.699517	0.896415	0.825697	0.977534	1.350571
gene-AT4G	63.8778	60.3555	62.21007	22.58452	21.12621	23.1704	16.50087	14.8057
gene-AT3G	3.355223	2.642417	3.224581	1.025271	1.122936	1.024804	2.053303	2.531908
gene-AT5G	7.366349	5.715704	6.2906	6.543464	6.366673	5.651372	9.134675	8.594094
gene-AT4G	40.67433	41.64439	42.43184	46.95839	46.01492	50.01342	98.2326	103.2748
gene-AT4G	11.23916	13.56185	12.99588	16.8936	15.89541	16.10091	5.696542	4.877762
gene-AT5G	5.803234	6.508172	5.328962	7.762392	9.845052	10.21922	3.015484	2.791052
gene-AT1G	4.138082	3.469853	4.708622	4.942127	3.980022	3.774576	7.595312	8.50948
gene-AT1G	2.450819	2.461527	2.591967	0.701838	0.740988	1.157894	0.293795	0.910792
gene-AT1G	0.306715	0.121229	0.317764	0.944283	0.717363	0.854852	0.52634	0.444817
gene-AT3G	0.376425	0.37665	0.368088	0.702689	0.504737	0.710776	0.413213	0.529719
gene-AT5G	0.956781	1.345503	1.277071	2.474834	2.026566	1.725203	0.676672	0.82754
gene-AT3G	8.0604	10.37264	10.29625	4.271742	3.519929	4.527398	9.733117	9.662695
gene-AT4G	0.208507	0.156272	0.112962	0.15747	0.1209	0.036602	0.036739	0
gene-AT5G	73.81626	73.88427	73.70566	60.30534	65.57762	67.24789	108.7216	116.9812
gene-AT2G	6.567817	6.159718	6.261453	15.67102	17.2045	15.21711	3.518069	4.370477
gene-AT2G	3.630679	3.066425	3.030863	0.38439	0.157234	0.6291	0	0
gene-AT1G	14.34782	16.57827	16.95148	9.244859	10.24172	10.01163	3.098222	3.592967
gene-AT4G	0.035076	0	0.091386	0.138924	0.140913	0.047438	0	0
gene-AT1G	2.161618	2.242585	2.727078	1.887692	2.1688	2.033591	0.976342	0.828389

gene-AT3G	0.168388	0	0.128745	0	0	0	0	0
gene-AT3G	8.325328	8.651353	8.51565	9.258397	10.34006	9.173303	4.599448	3.801337
gene-AT1G	9.124084	9.697216	8.431509	6.502371	6.70849	6.331296	18.19741	18.50649
gene-AT1G	0.529447	0.577417	0.761354	0.509123	0.849777	0.674914	0.192188	0.259529
gene-AT5G	54.1929	61.07449	54.54613	125.591	122.5132	117.5459	69.54142	74.8915
gene-AT1G	10.30135	9.734852	10.84301	11.8996	14.06133	15.09755	3.8696	4.099944
gene-AT4G	8.062537	9.328301	9.601725	8.7773	7.992456	8.549972	14.36898	10.73687
gene-AT3G	2.312554	3.156779	2.745649	2.888553	2.592559	2.526292	3.483412	3.180173
gene-AT5G	2.371468	2.095852	2.508027	2.029615	2.321362	2.690998	3.960138	3.914892
gene-AT5G	12.00353	8.629646	11.9984	5.131481	5.981744	3.835235	1.482446	1.6332
gene-AT3G	4.389567	4.532449	4.616272	2.418128	2.08538	2.852991	6.394683	6.371552
gene-AT3G	13.54758	15.06737	12.99233	29.16532	27.88542	32.32774	6.817238	5.703826
gene-AT5G	40.28925	39.04491	41.88732	22.47794	22.46636	22.92537	49.18652	51.46213
gene-AT4G	6.588291	7.42971	7.492383	7.404715	8.932764	7.170204	2.484597	3.621866
gene-AT2G	7.630882	8.04656	9.269403	5.233153	6.178574	5.788265	13.33054	12.54732
gene-AT2G	5.739409	9.92079	6.428198	0.357423	0.4072	0	8.354247	7.928118
gene-AT2G	17.26229	14.89205	14.70872	19.14858	19.52038	19.82497	27.30383	29.867
gene-AT5G	469.0678	459.8968	465.4637	186.9607	186.2102	183.3269	650.1901	669.9632
gene-AT4G	49.20565	47.17239	51.76963	26.75304	26.4982	26.83414	61.45644	64.71626
gene-AT5G	0.294866	0.297296	0.49454	0.312486	0.401984	0.357779	0.819502	0.849216
gene-AT5G	2.705253	2.661205	3.565303	2.678549	2.405455	2.655061	1.941731	1.838669
Arabidopsi	10.79013	10.15234	10.56006	7.311	7.870237	6.947112	3.77688	4.679077
gene-AT2G	0.173255	0.840185	0.946184	105.339	104.7607	102.2673	0.198788	0.330785
gene-AT5G	19.76018	18.06416	18.94168	14.66277	15.21102	15.06215	27.20515	27.52966
gene-AT1G	9.372616	10.27466	10.38683	8.049803	8.690491	9.328758	4.122781	4.96067
gene-AT4G	5.975884	5.144598	6.476767	3.636563	2.952861	3.194681	1.974369	1.359242
gene-AT3G	3.376956	4.789772	3.48312	1.236422	1.822505	1.274549	1.572126	1.40392
gene-AT1G	0.578651	1.06473	0.578164	0.878516	0.901044	0.961462	0.255868	0.753993
gene-AT2G	0.941729	0.731656	0.72491	0.786549	0.808196	0.541893	1.375556	1.120038
gene-AT2G	14.74251	14.74067	11.50713	20.06311	18.75178	18.18492	7.998188	6.909104
Arabidopsi	0.459819	0.300489	0.417732	5.075244	4.198666	2.924176	0.588354	0.518779
gene-AT2G	130.4056	128.806	127.7696	116.7907	117.2067	116.4469	206.2138	220.6971
Arabidopsi	4.089203	3.333515	3.050555	2.802382	2.842929	2.454889	1.231058	1.077665
gene-AT5G	19.1923	19.03291	17.86864	17.99424	18.49532	18.78194	31.42526	30.87422
gene-AT3G	19.06581	20.88811	18.92008	14.95978	14.65127	14.96723	24.82828	26.49039
gene-AT2G	601.1054	578.9587	613.7623	426.3969	415.2297	426.1786	258.6151	239.4717
gene-AT1G	101.2952	100.3982	105.8478	256.3561	260.8629	246.9682	92.86343	85.8755
gene-AT5G	0.391836	0.925206	0.773022	0.754033	0.740214	0.702269	0.421323	0.369952
gene-AT1G	12.70177	11.8498	13.10417	12.19528	13.21104	13.04607	21.24919	18.59709
gene-AT1G	5.656371	6.468714	5.862207	6.635437	5.707286	6.150062	8.67204	8.716032
gene-AT4G	306.7366	298.8039	306.2052	419.549	418.4951	416.5397	150.2351	151.309
gene-AT5G	14.50455	17.19361	14.88898	11.70371	12.71555	11.59339	29.09164	31.27197
gene-AT1G	11.34467	10.71779	12.38784	1.729311	1.402892	1.73247	3.779884	3.491829
gene-AT3G	156.7669	155.5567	157.8884	160.3564	158.0397	153.5857	170.9836	175.2574
gene-AT1G	2.794192	2.511182	2.947434	2.545096	4.126942	4.004342	0.863829	1.207555
gene-AT5G	2.474273	2.288098	2.213306	3.319853	2.971937	3.525083	0.868986	0.805879
gene-AT1G	1.583053	0.927663	1.176121	5.026885	4.62485	4.334598	1.4897	1.476402
gene-AT5G	15.1823	14.76803	15.01135	19.60211	19.16577	21.22588	6.33695	6.103051
gene-AT2G	3.69805	3.393021	3.822155	2.94938	4.294044	3.379301	0.500841	1.421044
gene-AT3G	0.101638	0	0.06813	0.606527	0.592035	0.650966	0	0
gene-AT5G	0.225692	0.208293	0.20634	0.116384	0.132321	0.128945	0.248721	0.089017

gene-AT1G	7.988709	7.785176	8.266562	7.288224	6.188333	8.203955	12.46542	13.26812
gene-AT1G	1.104366	0.582467	0.888173	0.704118	0.512718	0.290718	0.93793	0.896737
gene-AT3G	40.26133	40.45592	42.01203	31.6995	31.96285	33.09058	47.19496	48.62637
gene-AT3G	35.08763	39.96923	38.63161	32.13162	29.27548	27.8644	13.32187	15.04658
gene-AT5G	21.78343	21.8525	23.00192	104.5493	101.0715	101.5777	16.88813	18.66478
gene-AT5G	1.765994	3.123143	1.228599	2.338453	1.983401	2.19607	1.276438	2.529705
gene-AT5G	6.457038	5.18398	5.938427	5.015832	3.901938	4.703843	12.32094	11.57632
gene-AT2G	11.68026	12.42807	13.57022	14.24455	14.02601	14.39582	5.152297	5.892669
gene-AT4G	14.09919	14.78146	15.06854	20.55555	20.3646	19.60936	10.06497	12.44038
gene-AT1G	61.79219	60.39136	64.72079	1316.385	1310.107	1313.341	97.85001	98.61541
gene-AT4G	54.22981	50.05949	56.39978	30.87285	30.10665	30.99452	67.76373	74.03331
gene-AT1G	6.956105	6.254317	6.0387	3.369241	3.444897	3.637884	7.591862	8.152285
gene-AT2G	1.211894	1.135606	1.036362	1.159935	1.618255	1.791642	0.304915	0.366112
gene-AT3G	35.50306	36.97986	38.56178	47.35018	48.40657	48.6036	27.57773	27.87769
gene-AT4G	40.05334	37.49983	39.64656	24.50632	23.8345	22.37358	55.28224	53.96098
gene-AT5G	6.800532	7.149379	7.218681	6.94396	5.134748	7.307671	6.382232	5.592889
gene-AT5G	36.56966	37.63332	34.68863	27.9441	24.92761	23.48356	61.20845	63.89668
gene-AT1G	13.67068	13.80597	13.65485	10.44845	10.10226	9.602995	23.59641	24.56973
gene-AT2G	2.99023	3.115322	3.399442	2.122602	1.943282	2.027614	1.046003	0.758332
gene-AT5G	129.3056	133.5504	123.6612	146.4652	143.9279	133.5397	155.5129	147.2456
gene-AT2G	90.16292	84.64276	89.05476	46.68849	45.57562	44.60673	87.27307	100.9819
gene-AT2G	12.82478	12.26698	11.98256	14.52057	13.41357	14.93335	8.425116	7.001321
gene-AT3G	1.391798	2.937065	2.187301	3.163237	4.262242	3.429581	1.233711	0.936687
gene-AT5G	0.97047	1.319509	1.446421	0.717265	0.262372	0.524701	0.366846	0.311569
Arabidopsi	0.614984	0.454339	1.055903	0.511764	0.690517	0.199866	1.304599	0.929005
Arabidopsi	2.944397	2.244099	2.530619	3.624032	2.030069	2.479991	0.75143	1.733206
gene-AT1G	7.739997	8.042237	8.022533	3.186332	2.690521	3.018249	15.26485	16.33525
gene-AT2G	177.1332	166.5157	173.39	307.1275	324.3179	325.4962	34.47848	30.91214
gene-AT2G	40.32325	39.81504	42.4782	23.80797	22.18094	24.76649	38.24204	34.00172
gene-AT4G	419.644	420.2444	421.9486	318.3368	324.2126	316.3432	639.9514	645.7068
gene-AT1G	36.39361	32.17434	32.38036	32.87025	33.58202	32.27681	35.25374	38.93512
gene-AT1G	15.96136	15.64878	12.90309	11.90069	12.39568	13.31253	21.92836	21.24387
gene-AT5G	22.04731	19.74404	20.37034	18.43938	19.63355	19.38101	7.585935	6.486104
gene-AT2G	33.32423	36.34609	37.29606	8.334513	8.27529	7.732079	18.85835	20.66462
gene-AT4G	4.843512	5.766901	5.378047	4.322874	5.279711	3.954144	2.555702	1.684064
gene-AT2G	58.38213	62.20562	62.27939	30.69095	29.02254	30.33357	28.61936	28.66485
gene-AT5G	5.964507	6.267678	6.038903	5.805095	6.3409	6.264496	1.062126	0.683596
gene-AT1G	2.911086	3.675222	3.58855	4.941781	5.84195	4.302084	3.404855	3.168911
gene-AT3G	16.54558	15.57031	16.56454	14.67249	15.81265	15.69025	22.32391	22.30308
gene-AT1G	3.630899	2.132049	2.694453	2.48975	2.311689	3.074735	1.64417	2.187874
gene-AT1G	19.56179	19.48899	18.48155	17.68456	17.62709	17.86171	22.26792	22.12682
gene-AT2G	1.306691	1.74242	1.180173	1.743732	2.406319	2.997173	1.25636	1.233844
gene-AT1G	12.40211	12.52125	14.2795	11.66821	11.7425	11.00936	4.129474	5.845396
gene-AT2G	2.378002	2.665362	3.780859	1.382022	1.73446	1.520873	0.94806	0.686632
gene-AT2G	3.386019	3.644106	3.501227	2.289003	1.78574	2.067642	4.030699	4.201217
gene-AT4G	210.7267	217.3701	212.0986	178.5089	183.3206	173.3574	371.4569	330.1382
gene-AT4G	3.209344	3.350145	2.594962	0.910992	1.216666	0.854799	0.837636	0.517478
gene-AT1G	0.614876	0.421214	0.349959	0.944964	0.458036	0.745646	0.509513	0.411263
gene-AT1G	3.852374	4.727904	4.393692	0.509278	0.947282	0.487255	4.538387	4.134004
gene-AT5G	1.105119	0.536284	0.853309	2.879933	4.038033	3.568522	0.260546	0.716859
gene-AT3G	45.70835	39.42492	42.38468	33.20204	33.35327	33.43562	16.90201	15.51437

gene-AT1G	11.83804	12.06168	12.8532	9.112272	8.32415	8.538671	11.95013	12.19564
gene-AT3G	0.777591	1.307885	0.716091	0.728593	0.227357	0.072685	1.89764	2.624743
gene-AT4G	10.22867	9.185081	9.688547	62.31699	65.39527	64.81522	5.908732	5.854416
gene-AT3G	6.511324	6.120465	6.195657	3.823798	3.606227	4.925064	12.28649	13.06525
gene-AT5G	3.553038	2.736017	2.900154	2.677739	1.937266	2.557041	3.838486	4.570286
gene-AT1G	2.217796	1.490603	1.702069	2.858471	2.577127	2.754674	3.264354	1.731349
gene-AT1G	7.710446	9.956376	6.200254	4.193615	3.438894	4.457083	6.952192	6.573557
gene-AT1G	3.372302	4.042632	3.177142	1.620827	2.021434	1.215717	2.827105	2.224847
gene-AT1G	14.10485	17.33205	13.82825	7.750066	9.361261	8.224738	9.314592	8.896194
gene-AT1G	46.63044	42.30563	46.63045	23.34971	21.85478	22.45251	36.19651	39.17837
gene-AT3G	87.96642	89.01854	88.12882	52.05963	54.04281	49.68089	119.6383	119.3829
gene-AT3G	2.666429	2.349231	2.855839	3.775967	3.595387	3.333353	1.214469	1.321894
gene-AT5G	30.74014	28.53342	31.03667	53.41169	56.00826	55.24763	19.09017	22.31332
gene-AT3G	1.834638	2.645352	1.258608	0.674891	0.847211	1.063478	2.405186	1.915435
gene-AT4G	3.129917	3.282515	2.884516	4.731115	4.85947	5.444417	2.923451	2.68973
gene-AT5G	107.9637	107.4018	105.7603	155.7591	154.741	150.6313	68.72908	71.51082
gene-AT3G	27.80963	27.00145	28.84671	43.30952	42.73538	44.1836	8.768845	9.194834
gene-AT4G	2.567916	3.252693	1.86522	1.395304	1.771759	1.215822	1.941941	2.252
gene-AT4G	4.77996	5.322544	6.185428	6.631272	6.732611	5.29143	0.616427	0.717591
gene-AT1G	1.872736	1.834138	1.599361	1.643118	1.672851	1.287292	1.478253	2.049648
gene-AT5G	0.436955	1.051884	0.342524	1.829468	1.733998	2.022188	0.398134	0.547632
gene-AT1G	1.354489	1.216745	1.379569	1.230201	1.270527	0.903118	3.36158	2.617809
gene-AT2G	14.50108	12.86956	13.34669	37.44013	35.99389	38.59362	5.878492	5.768209
gene-AT3G	40.43721	38.37647	40.54827	19.41217	18.30298	19.86433	31.45203	34.73218
gene-AT4G	1.717555	2.068401	1.701504	0.381771	0.503371	0.156253	0.266489	0.112708
gene-AT1G	27.11011	25.41293	25.96578	21.06716	19.36754	20.83189	35.05818	35.94107
gene-AT1G	21.17484	21.46306	19.11147	12.26511	12.87587	11.60355	25.87045	26.63771
gene-AT4G	1.671291	1.426584	1.730416	1.760076	1.917796	1.312814	2.621025	2.817318
gene-AT3G	15.60704	15.42171	15.13022	7.474136	6.318308	7.653345	17.28967	17.34233
gene-AT5G	5.424157	5.563027	4.491855	7.024483	6.164915	7.177291	7.738487	7.951537
gene-AT5G	4.41868	4.100238	4.072129	4.952743	5.091348	5.04859	5.984115	5.384066
gene-AT3G	2.461286	3.133833	2.402277	2.425144	2.966986	2.524603	5.92853	5.104103
gene-AT1G	4.564271	4.654368	4.828139	4.317884	3.429452	3.400682	7.434372	6.814844
gene-AT3G	2.097637	2.997851	3.634129	2.248996	1.640012	1.7484	5.558397	4.730745
gene-AT4G	0.89457	1.354613	1.480042	0.829145	1.38031	1.256582	0.259437	0.517483
gene-AT4G	51.70407	54.72516	53.01692	55.71795	56.03629	54.81008	62.68903	67.42309
gene-AT5G	0.319118	0.388959	0.460826	0.891657	0.679725	0.872191	0.125607	0.273403
gene-AT4G	42.22968	41.54586	44.20094	57.41443	58.27229	56.90598	18.85543	21.05386
gene-AT5G	2.885327	2.351552	2.277199	3.662871	3.227891	3.262405	1.35482	1.295031
gene-AT3G	2.720798	4.043802	3.558602	5.581777	6.47298	5.430183	1.554421	2.130792
gene-AT5G	2.515377	2.74401	2.820863	1.106915	0.757426	0.603136	2.09513	1.748465
gene-AT1G	6.559453	6.431404	6.513449	6.306121	6.7335	6.004083	2.052767	2.40936
gene-AT2G	0.02779	0.210799	0.04974	5.15353	3.635904	4.529415	0.170643	0.193531
gene-AT1G	16.86067	16.20003	16.18257	11.41411	10.06308	10.50769	6.058046	5.690125
gene-AT4G	17.12067	17.80285	18.8446	28.23255	26.82639	25.43339	2.142809	1.572956
gene-AT1G	3.158205	3.041738	3.251215	4.883201	4.716132	4.935039	8.833878	9.763505
gene-AT3G	84.41473	82.34261	80.09831	56.31569	57.25847	57.29821	140.4913	148.0422
gene-AT2G	2.563282	1.844022	2.160852	3.958197	3.484026	3.442597	1.654073	1.720957
gene-AT5G	1.959969	1.816487	3.424623	0.662456	1.406325	0.750921	2.40968	2.622704
gene-AT1G	36.57604	40.07465	41.32319	65.89241	65.83209	64.42822	22.20351	21.38685
gene-AT5G	2.546338	1.81957	2.493776	1.126634	0.849967	0.837206	0.581832	0.869723

gene-AT3G	17.81461	19.43535	20.28333	32.71319	28.87839	26.98959	14.17103	14.07667
gene-AT4G	1.298384	1.183065	1.129807	4.88	5.209826	5.390392	1.380787	1.899491
gene-AT1G	10.24357	10.88579	9.475862	11.2825	10.83159	12.67755	30.11632	26.18966
gene-AT2G	0.140211	0.07417	0.099361	0.020993	0.161331	0.102013	0.400711	0.461664
gene-AT4G	9.818214	8.094343	5.62833	8.075745	7.218903	7.214082	9.972176	8.679908
gene-AT4G	33.22496	33.71609	35.09114	20.77546	20.73851	20.82118	49.11079	48.549
gene-AT2G	4.73748	4.127048	4.522927	21.15879	21.70413	18.67921	3.210028	3.355875
gene-AT5G	1.186185	0.835596	1.510831	0.671019	1.340331	1.36275	2.955743	2.011043
gene-AT4G	0.235463	0.541921	0.567092	0.798049	1.145951	1.194775	0.289362	0.178383
gene-AT4G	5.206016	4.346445	4.209144	2.397395	1.809733	1.771657	7.575359	8.878899
gene-AT4G	1.831027	3.115585	2.033163	2.70733	3.134334	2.595726	3.117755	3.041354
gene-AT3G	38.88566	42.24688	40.84345	35.69604	38.2146	34.43058	56.71271	56.4656
gene-AT1G	27.61982	27.64459	28.76391	41.33669	41.8291	39.79933	13.99731	16.68553
gene-AT1G	28.18965	29.90709	28.39713	34.32933	33.97593	32.83505	24.68472	25.1599
gene-AT4G	21.77956	21.17734	20.67255	20.45373	20.1393	17.92811	21.87193	23.34702
gene-AT5G	41.86609	42.2278	41.00099	21.43572	20.44791	18.79683	32.1405	32.8772
gene-AT4G	5.160963	5.769204	4.929611	1.445924	1.049943	1.517992	1.556441	1.294295
gene-AT3G	12.38379	10.47417	10.20668	3.642604	4.824218	4.215841	7.177544	8.845962
gene-AT1G	2.413484	2.317051	2.292555	2.990495	3.0201	2.928056	0.971133	1.021358
gene-AT5G	33.72654	38.45154	34.1809	24.52982	26.20474	23.66168	14.002	10.75127
gene-AT5G	43.1821	44.64912	42.68359	25.45172	25.88873	25.34489	65.66811	67.00245
gene-AT3G	20.43064	19.37608	19.08908	7.31058	7.107614	5.788727	39.14386	41.02299
gene-AT5G	3.033821	3.128056	3.641369	4.750126	4.007082	3.775553	0.911388	1.618419
gene-AT3G	38.31818	35.0104	38.55043	28.27317	26.94263	27.19548	63.63184	67.39031
gene-AT3G	0.33741	0.602024	0.808737	1.084776	1.029884	1.281114	1.607094	1.187438
gene-AT1G	16.86726	15.3321	16.41196	29.33084	27.99187	29.20775	5.203912	4.888597
gene-AT3G	359.6173	348.0082	354.7539	601.5516	603.2603	590.5808	242.1916	250.9646
gene-AT4G	101.4676	104.8461	105.2055	90.20657	86.97601	86.75842	164.4482	160.4852
gene-AT1G	1.332291	0.94873	1.377373	11.78573	11.38954	11.2926	1.924773	1.302806
gene-AT3G	8.996071	10.47777	9.062995	18.34602	19.75079	17.49721	8.670303	7.970958
gene-AT1G	10.96121	9.893139	8.890679	7.193643	8.136474	6.07884	2.156524	2.061984
gene-AT1G	1.527914	2.300692	2.066959	4.336666	4.274972	3.927988	0.896147	1.144038
gene-AT5G	168.2347	161.1196	165.3553	50.55479	48.5338	49.94671	120.7259	125.9651
gene-AT5G	55.5076	52.54702	55.81798	25.75713	26.67843	25.89466	128.7082	115.6946
gene-AT3G	1.712258	1.865176	1.782739	0.91095	0.743843	0.745279	1.171188	0.890543
gene-AT1G	3.462171	2.822637	1.909659	2.54652	3.484222	3.737764	3.952361	3.664564
gene-AT3G	13.09965	15.93273	15.96111	41.60613	39.30151	42.81254	14.7324	14.55041
gene-AT3G	0.503459	0.628676	0.520449	0.018745	0.033372	0.018123	0.082987	0.07396
gene-AT2G	4.437275	3.670796	4.228109	6.33396	6.333843	5.835519	5.988697	6.241271
gene-AT5G	43.48816	43.35955	40.2078	31.48773	31.81502	32.2485	12.44242	13.50758
gene-AT4G	11.47856	12.64428	10.46538	8.804088	8.389302	8.425222	1.93051	2.596267
gene-AT3G	0.591767	0.452855	0.704703	0.808211	0.652205	0.774375	0.077493	0.137643
gene-AT4G	1.437175	1.320087	1.437035	0	0	0	0	0
gene-AT1G	17.60488	20.2623	20.26261	12.17419	12.63851	10.97262	28.4997	30.87282
gene-AT3G	7.152619	7.352808	8.751429	4.708988	4.583986	6.139727	5.002819	6.606113
gene-AT1G	0.485959	0.685309	0.380423	0.393894	0.703319	0.497887	0.230609	0.216323
gene-AT3G	5.318156	4.036316	3.603602	2.781852	2.786924	3.251193	9.823985	11.31109
gene-AT2G	3.467678	3.813732	4.76559	3.505539	3.542634	2.959737	3.746876	3.257201
gene-AT1G	1.486761	1.081298	0.977554	2.734158	3.392827	2.918418	2.342361	1.337252
gene-AT3G	3.995728	3.576833	3.638852	5.145354	4.854672	4.814445	1.203845	1.33822
gene-AT3G	0.619075	0.568804	0.605797	0.282405	0.322868	0.099878	0.112794	0.223648

gene-AT5G	0.608497	1.486398	1.367248	2.767043	1.778465	1.738526	0.403152	0.69541
gene-AT4G	20.45576	22.3499	21.69268	16.82733	15.99578	16.29928	35.29953	39.7668
gene-AT4G	8.708371	8.396156	8.173796	8.247481	6.592893	7.568892	3.268776	4.05694
gene-AT3G	1.857055	1.77873	1.873976	3.01599	3.540766	2.093157	2.294008	1.758138
gene-AT1G	105.9301	97.36757	94.97812	72.64315	80.89321	72.85819	173.3639	183.2476
gene-AT5G	15.64459	15.35226	14.02618	15.67524	13.27298	16.37314	6.484185	7.002244
gene-AT4G	129.3525	119.6159	128.0981	66.16361	65.86619	64.0173	41.26885	42.93956
gene-AT5G	0.569407	0.428756	0.37262	0.676651	0.294359	0.436168	0.980637	1.579569
gene-AT3G	60.56513	60.79386	60.44706	46.74752	45.62768	45.41106	95.80064	93.43029
gene-AT3G	4.18587	4.300224	4.164514	1.792165	2.481332	2.386821	6.520592	5.793938
gene-AT5G	4.439531	3.71855	3.665803	7.021649	8.288627	8.084923	2.998915	1.806504
gene-AT5G	15.87255	16.60063	16.41647	13.28684	14.86133	12.58417	27.53507	31.20362
gene-AT2G	146.1097	136.8764	145.7522	85.54237	82.26366	82.38274	259.2732	256.0082
gene-AT1G	28.56884	31.04101	29.91328	28.9127	31.13612	30.7235	7.936633	7.171441
gene-AT2G	7.891013	7.799372	7.757276	12.45457	11.79388	12.61715	4.808798	5.894599
gene-AT2G	0.702814	0.635813	0.743497	0.481918	0.59222	0.219543	1.754622	1.843105
gene-AT1G	2.847924	3.729774	3.730363	1.968132	1.954491	2.238676	10.67899	10.31514
gene-AT1G	7.124542	5.480699	7.751837	10.53426	11.86363	10.45942	5.240045	4.123216
gene-AT4G	0.954721	1.074075	1.111847	1.785292	2.249063	1.760705	0.326896	0.34038
gene-AT5G	1.067009	1.19347	1.049829	1.112565	0.887167	1.61866	0.640001	0.808881
gene-AT5G	16.5866	16.81455	16.72156	6.416471	7.202203	7.815075	2.624404	3.035098
gene-AT1G	15.76866	13.98392	12.98574	6.564427	7.240405	6.582722	2.949439	3.744375
gene-AT2G	8.084064	6.927764	7.738722	6.501904	6.200548	5.748722	15.23092	14.09794
gene-AT2G	6.350164	7.120257	6.51453	5.505402	5.74101	5.635736	2.76628	2.74167
gene-AT1G	3.898106	3.742677	3.467487	7.327276	8.150499	9.995188	2.677011	3.653963
gene-AT3G	1.966792	2.694092	2.391401	2.978149	3.66185	2.622696	0.665673	0.810807
gene-AT2G	9.701049	9.707541	10.53026	10.37596	10.80203	11.0194	4.031497	3.351699
gene-AT5G	12.2614	10.97823	12.67754	13.11403	16.51902	15.50035	5.562286	5.077964
gene-AT5G	1.031254	1.085484	1.251374	0.316452	0.311188	0.731674	2.000641	2.196477
gene-AT3G	193.5758	203.3178	196.4464	96.46853	92.46094	97.65315	349.7902	353.3736
gene-AT5G	12.44	12.18605	13.15498	19.67673	22.81007	21.36684	11.30566	13.87245
gene-AT2G	18.04649	18.17046	18.83319	16.07344	16.08777	15.44568	28.90358	27.648
gene-AT2G	0.988181	1.646339	1.319894	0	0	0	0	0
gene-AT1G	17.34563	14.9114	17.64074	15.32471	14.18143	14.78788	7.631686	8.713058
gene-AT3G	4.02177	5.003233	4.003822	8.162891	7.026835	10.22554	2.818779	2.270678
gene-AT4G	308.0632	302.7171	311.7435	367.113	357.7943	354.0929	196.1658	210.8298
gene-AT3G	0	0	0	0.659954	0.577648	0.382165	0.166111	0
gene-AT3G	2.48076	1.774334	1.908626	4.183636	4.819418	4.482203	5.224761	5.304448
gene-AT1G	123.7486	125.084	121.0994	41.6049	40.59385	43.37417	186.6413	195.0572
gene-AT1G	50.52443	53.49363	53.60183	25.9225	25.514	23.41931	141.7061	144.161
gene-AT3G	5.310022	5.651468	4.57407	6.732847	6.829452	7.772683	2.188059	2.01612
gene-AT5G	27.04088	19.82392	23.12861	30.13137	30.11252	28.55267	15.71529	12.60239
gene-AT3G	4.22536	4.163389	3.480692	4.900767	4.569265	4.594807	11.3178	10.24355
gene-AT1G	372.4491	363.315	367.0176	188.0342	178.0289	182.1331	81.68665	92.08933
gene-AT1G	4.738499	4.981795	4.479751	3.722126	4.109873	3.814882	4.447147	4.869341
gene-AT5G	9.843488	9.121028	10.21692	10.02834	9.401706	10.04345	6.808939	6.893502
gene-AT4G	0.243677	0.183442	0.202677	0.801393	1.043633	0.74515	0.291607	0.17173
gene-AT4G	27.7223	30.75848	28.46911	9.288747	9.286406	9.824519	20.89551	21.79178
gene-AT2G	5.208755	6.395279	5.82084	2.95238	3.010663	2.137446	2.437283	3.094144
gene-AT5G	1.486675	1.723232	1.507999	1.009055	1.018467	1.118532	2.820332	2.522516
gene-AT3G	11.13016	9.539778	10.47989	15.55569	14.79056	16.31363	5.603767	6.188662

gene-AT1G	13.80346	13.91316	12.03879	13.35735	13.62025	13.39534	17.22174	16.45914
gene-AT4G	6.465986	6.372663	7.305735	6.504534	5.333933	6.111733	11.70197	10.46619
gene-AT2G	420.9178	418.7064	426.6009	355.347	359.8911	346.1129	612.7378	644.8268
gene-AT1G	5.921582	6.351111	6.136713	2.895695	2.113471	2.96655	0.85439	0.91092
gene-AT1G	0.470542	0.648187	0.883267	1.165924	1.204136	0.966437	1.522976	1.75036
gene-AT2G	13.2504	13.01421	13.50392	11.15697	11.11931	10.87538	22.20009	20.00486
gene-AT3G	9.215043	8.731795	11.49001	7.494732	8.73456	6.914392	2.723216	2.836111
gene-AT2G	13.67765	13.73441	12.86217	9.85373	10.74291	10.60632	3.434437	2.651099
gene-AT4G	3.608548	3.424384	3.772406	8.907683	8.823439	7.234044	2.172484	1.804971
gene-AT5G	85.07533	95.15321	86.78499	58.40793	53.78562	59.55345	157.0191	142.6855
gene-AT4G	892.1707	915.2256	907.8893	1528.049	1547.146	1504.759	424.6983	444.3815
gene-AT5G	3.259496	2.711171	2.379574	2.005332	2.073651	1.829078	2.992743	3.922449
gene-AT3G	0.448688	0.386011	0.561199	0.547113	0.69779	0.695366	1.492973	1.915793
gene-AT2G	2.60587	2.079336	2.120816	3.82545	3.998605	3.164708	1.050269	0.854939
gene-AT2G	0.196961	0.204556	0.098912	0.181833	0.323552	0.22741	0.260238	0.139875
gene-AT1G	4.213969	3.630187	4.625201	6.241777	5.420519	6.798327	2.99424	3.113386
gene-AT1G	0.928377	0.505536	1.177158	0.925475	0.844209	0.875411	1.892887	2.396845
gene-AT4G	113.5314	113.2485	114.2609	53.65006	51.08232	49.82357	28.59586	29.29089
gene-AT5G	12.83	11.23965	13.50693	62.50257	57.42953	63.48743	8.712996	8.546893
gene-AT1G	16.84107	19.36076	18.94286	9.491036	7.418356	9.799975	19.02697	19.13886
gene-AT3G	210.6405	202.3138	206.7706	136.1335	137.6431	135.7812	299.8099	321.4302
gene-AT1G	0	0	0.126287	0.623797	1.039346	0.688767	0.460029	1.205955
Arabidopsi	0.726569	0.87742	0.907098	0.194213	0.432319	0.388314	5.076103	5.625683
gene-AT5G	0.586983	0.573351	0.649212	1.13698	1.109393	1.034111	1.932363	2.159603
gene-AT5G	2.046937	2.741762	2.177856	4.534806	4.036513	5.076386	1.602958	1.812875
gene-AT2G	3.839374	3.2208	4.013475	5.08431	4.890466	3.602879	4.200082	5.561151
gene-AT5G	6.104192	4.955905	5.681712	3.628771	5.007133	5.915616	3.330473	3.441225
gene-AT5G	26.99234	27.71373	25.35617	32.33399	32.63049	31.58618	15.25766	15.88997
gene-AT5G	0.303373	0.283663	0.147831	0.26318	0.182684	0.173616	3.506206	3.90374
gene-AT4G	6.999725	7.440665	6.371718	5.15481	4.38998	4.311398	2.371135	2.504705
gene-AT4G	0.22565	0.160885	0.371007	0.700047	0.436881	0.595029	0.263771	0.017944
gene-AT2G	2.427994	1.63102	1.44046	2.070414	1.817128	2.206636	1.372137	1.133933
gene-AT1G	22.18912	21.82627	20.59739	17.48222	15.40084	17.33059	30.6034	32.24825
gene-AT3G	0	0	0	0	0.121319	0	0.17602	0.111165
gene-AT5G	0.920102	1.134415	2.060131	1.928228	1.300331	1.44168	3.584945	2.726956
gene-AT5G	2.70315	2.961413	2.581764	7.592455	7.500482	6.596179	4.778545	5.070435
gene-AT5G	4.221015	4.81551	4.557651	6.061041	5.75499	6.654688	1.837775	2.233062
gene-AT4G	0.399548	0.449037	0.316936	0.089307	0.07326	0.171533	0.163062	0.029546
gene-AT1G	12.85785	12.48573	11.95863	13.34268	13.87548	13.95598	13.72911	13.68353
gene-AT1G	8.321942	7.779731	7.365825	6.772036	6.446512	6.775943	10.76783	11.40288
gene-AT5G	1.273605	1.203867	2.157835	2.148858	1.960122	2.035359	2.502456	1.753235
gene-AT4G	14.57008	13.99756	14.47431	10.56229	9.770521	11.60964	23.15157	20.99749
gene-AT4G	6.882397	7.082555	8.04149	13.36566	11.4907	12.03863	3.416859	2.362419
gene-AT5G	40.5975	35.59702	35.65148	56.92307	61.10547	59.24803	18.68179	18.29265
gene-AT3G	13.7827	13.07653	14.34313	12.11757	13.42124	11.39024	17.09293	18.75643
gene-AT4G	553.7575	550.321	562.6738	451.9831	448.5809	445.3326	741.0837	786.5399
gene-AT4G	9.568487	9.974877	10.92847	5.288098	5.520844	4.647089	15.88582	16.56874
gene-AT3G	17.93662	19.37652	20.86269	21.71542	19.85689	20.15905	7.82711	7.247982
gene-AT3G	13.18252	12.35122	12.69132	8.544265	8.077564	8.42468	10.47716	11.33572
gene-AT3G	0.996801	1.208808	1.186345	6.507615	7.421376	7.672991	0.186008	0.2322
gene-AT3G	102.5277	108.5264	106.2874	72.63976	69.0147	70.28653	29.21642	25.67351

gene-AT5G	2.499237	2.257693	2.207284	1.078327	1.306705	2.081297	0.754522	0.900692
gene-AT1G	0.244547	0.221021	0.278852	0.934825	0.718905	1.268391	0.357499	0.360168
gene-AT2G	0.955404	1.854933	0.842411	2.113723	1.794312	2.305904	1.838459	2.92745
gene-AT5G	21.5854	21.36048	22.38162	20.55801	19.30302	18.63212	10.00194	11.01712
gene-AT5G	13.84217	12.99274	12.26521	38.47585	37.6607	37.54898	21.36337	21.32145
gene-AT5G	16.9168	16.43565	13.99418	20.29008	16.78203	22.04808	13.72553	16.14259
gene-AT3G	15.46943	15.91357	16.594	12.86478	12.92448	12.02847	7.57805	5.808136
gene-AT5G	2.566464	2.826526	2.550966	7.370791	6.837476	6.745699	0	0
gene-AT4G	1.398597	0.595942	1.00526	1.489474	0.980364	1.192851	0.694046	0.940826
gene-AT5G	11.9979	13.23274	13.24546	30.66163	33.24582	33.84064	18.3006	17.67274
gene-AT1G	1.667768	1.465951	1.812215	2.158111	2.001361	1.620962	2.834765	2.974296
gene-AT1G	125.9295	139.1619	125.3718	65.01038	72.22115	67.21227	227.7927	225.281
gene-AT2G	24.6744	24.65851	24.15844	24.9619	21.88851	22.03231	26.18651	27.78388
gene-AT5G	4.689948	7.707778	7.198543	8.240914	8.606982	7.792868	1.414972	1.520478
gene-AT3G	4.94007	5.028276	5.703888	1.990464	3.057446	3.229307	6.445233	6.45393
gene-AT4G	11.29836	12.08374	10.27882	9.004208	7.952781	9.61249	4.487433	4.104653
gene-AT3G	1.209735	1.272138	0.810799	0.313339	0.360842	0.176557	0.592869	0.504914
gene-AT4G	3.934082	3.943629	3.591596	2.212613	2.152966	2.253015	5.240883	4.324318
gene-AT3G	22.04355	21.7171	21.22445	14.89717	16.43981	16.20272	17.4757	18.48425
gene-AT3G	16.12315	20.17137	14.45304	15.90601	16.12931	17.01372	33.63488	37.47157
gene-AT1G	9.383865	9.56415	11.54715	20.13675	22.53876	20.38796	11.69647	14.02184
gene-AT1G	177.118	175.1856	172.777	111.3601	105.8972	104.8135	120.5561	133.4525
gene-AT4G	0.995849	1.342486	0.703027	0.333356	0.092653	0.101791	0.027664	0.111193
gene-AT3G	0.602209	0.820303	0.486132	0.383508	0.915422	0.691681	0.375521	0.58197
gene-AT5G	1.060597	1.080477	0.734076	1.965705	1.865247	1.638371	0.296766	0.158958
gene-AT1G	0.561795	0.937642	0.488699	0.130416	0.293154	0.302778	0.928863	0.842
gene-AT3G	1.457027	1.106381	1.21549	2.899897	3.02128	3.174983	1.084119	1.06072
gene-AT5G	0.782523	0.601557	0.993146	0.425932	0.141317	0.249048	0.155926	0.227837
gene-AT3G	6.357608	6.828379	5.244111	14.76124	13.39525	14.22234	6.48281	5.373055
gene-AT2G	3.094687	3.39142	3.829115	3.975312	3.332723	4.551284	5.552916	5.264576
gene-AT5G	2.598107	3.208656	2.892866	10.32306	10.45916	9.284126	1.354423	2.006366
gene-AT3G	11.18859	13.06515	10.86906	8.039547	6.880628	9.876844	2.487075	3.252851
gene-AT3G	1.550368	1.208948	1.623942	1.317589	1.248534	1.580203	2.737578	3.271467
gene-AT3G	2.468559	1.921016	2.160882	0.330562	0.389544	0.716205	0.063665	0.167716
gene-AT1G	1.391057	1.348868	1.233278	9.959415	10.49182	9.473489	0.158148	0.263271
gene-AT1G	0.835023	1.056491	0.61138	1.75269	2.056278	1.772057	1.133993	0.889729
gene-AT1G	0.133786	0.352638	0.173593	1.415129	0.630821	0.820923	0.69909	0.373616
gene-AT3G	3.926652	3.00297	2.022847	1.955187	2.368216	1.326589	3.559417	5.959585
gene-AT3G	8.898171	8.385497	8.535298	1.914509	1.105394	2.080947	4.537674	4.697886
gene-AT1G	11.29408	11.54153	11.20208	2.185393	1.792978	2.111797	6.631694	6.159183
gene-AT3G	15.75846	14.75162	15.30088	24.38202	21.72776	24.11348	9.112621	8.7981
gene-AT1G	1.418475	1.154797	1.085726	1.67307	1.160127	1.555575	1.491131	1.132859
gene-AT1G	0	0.117113	0	35.92117	32.27152	38.02084	0.199566	0.367827
gene-AT3G	4.102019	3.894957	3.322441	10.99	10.10679	10.60209	2.322596	2.481594
gene-AT2G	9.379697	9.510751	9.616342	12.08281	13.74292	12.95622	9.413474	7.607497
gene-AT4G	123.5648	118.2054	119.597	85.45728	88.01932	88.39791	186.0184	185.3934
gene-AT5G	4.488742	4.936148	4.043766	2.045309	1.556672	1.370871	0.867871	0.664045
gene-AT1G	7.571536	7.571167	7.319086	8.979251	9.139906	8.367059	6.086654	6.19986
gene-AT4G	2.907509	2.598225	3.473191	0.826547	0.867705	0.540049	4.759968	4.407948
gene-AT2G	17.77085	15.77183	15.98578	9.797353	10.34675	9.466467	9.665232	9.401172
gene-AT3G	8.456442	9.12306	10.28226	6.924527	6.922732	7.766985	8.926286	8.536548

gene-AT2G	17.51337	15.88831	17.48916	11.44856	12.30462	12.17876	12.66762	13.0288
gene-AT4G	55.14987	55.39098	55.05407	43.87233	42.90151	44.27857	112.7046	116.3686
gene-AT1G	537.2159	568.6733	542.1718	381.9486	381.9943	379.2188	230.4551	218.371
gene-AT4G	0.671782	0.800951	1.07228	0.481318	0.862755	0.82203	0.47864	0.593258
gene-AT5G	0.67702	0.447094	0.530117	1.169046	1.157151	1.11153	0.951784	1.235932
gene-AT2G	4.461024	4.417551	4.928227	0.860973	0.521069	1.072767	10.29235	10.34632
gene-AT2G	5.13204	6.043487	5.324806	6.223239	4.721416	4.927636	2.34548	2.133086
gene-AT2G	4.523165	4.063172	4.608689	6.804307	6.242576	6.735437	8.789603	7.151226
gene-AT2G	1.024301	1.456578	1.145507	1.738258	1.145694	1.200698	0.317392	0.718743
gene-AT2G	152.2312	153.2316	150.1989	141.6195	137.5645	141.4501	245.0692	251.3292
gene-AT1G	11.47147	11.77774	11.27517	6.402511	5.543926	6.057065	15.21361	18.12247
gene-AT4G	416.3971	396.7563	409.7494	434.8535	435.4029	429.673	808.7984	794.7934
gene-AT2G	6.131416	6.259094	6.626496	7.586107	6.696316	8.098748	3.66983	4.709317
gene-AT4G	1.378419	1.023662	1.459245	0.823638	1.044598	1.096715	2.568912	4.367607
gene-AT1G	4.49111	4.616091	3.850031	14.80106	15.50402	14.84932	4.412369	4.389649
gene-AT1G	2.392013	3.311913	2.708278	2.398276	1.690792	2.815307	6.181252	4.711713
gene-AT3G	6.835927	7.915412	5.845438	5.08817	5.356828	6.180277	8.793805	9.967205
gene-AT4G	396.2083	386.6392	406.5569	262.5531	269.7531	261.142	578.0612	592.4223
gene-AT3G	22.18345	13.28867	16.58914	4.065372	5.448948	4.885871	7.280633	6.804192
gene-AT5G	0.693469	0.92799	0.566852	0.452642	0.8172	0.402162	0.881161	0.519687
gene-AT2G	10.44895	9.175113	11.4278	3.456123	3.75614	4.694523	2.441341	2.053224
gene-AT4G	19.61635	18.26852	17.85512	22.68274	19.77458	21.45285	11.16617	9.379271
gene-AT1G	5.490916	5.517745	5.015886	2.733232	3.087035	2.758796	2.575398	2.744627
gene-AT5G	1.870519	2.23932	2.128788	1.233188	1.355428	0.853193	0.732791	0.448318
Arabidopsi	0.700272	0.621944	0.826173	1.211466	1.599987	1.444307	0.249054	0.055155
gene-AT5G	14.03884	12.15471	14.16475	14.20115	13.30639	14.17399	24.36341	22.8251
gene-AT5G	4.036821	3.937459	3.890544	2.330865	2.584548	2.687216	1.575956	1.396462
gene-AT3G	18.45762	19.69478	20.88239	30.94524	30.0309	31.5299	11.37339	11.28582
gene-AT3G	117.5079	123.1923	123.9459	24.86052	24.67261	24.14521	122.8663	128.3839
gene-AT2G	1.026496	1.001045	1.198346	1.852634	1.448467	2.172428	2.059797	1.649807
gene-AT4G	30.55834	33.102	30.49542	22.74351	23.63365	22.78575	63.60184	65.91149
gene-AT4G	11.80558	11.6925	11.66847	6.517724	6.743153	6.564288	18.23133	18.24253
gene-AT1G	1.205166	1.036992	0.67758	3.818159	2.929932	2.858522	1.652047	2.102096
gene-AT2G	2.17053	2.517645	3.46753	2.702575	4.251738	4.058024	3.875403	4.323164
gene-AT1G	701.4609	721.9219	688.3069	349.7032	340.45	354.8841	1193.728	1098.193
gene-AT3G	1.408308	1.720192	1.364008	2.908077	3.343205	2.466696	0.484422	0.712835
gene-AT2G	80.43273	82.79746	78.43189	61.99706	64.16756	60.72962	32.2113	30.5942
gene-AT1G	1.799375	2.696061	2.301907	0.816905	0.820257	1.171462	3.826809	4.086267
gene-AT3G	15.01763	15.13265	16.47446	20.97651	20.42033	19.426	4.567247	5.411315
gene-AT1G	3.702962	3.641672	4.973669	2.874084	3.250886	3.957595	7.994181	6.230493
gene-AT5G	2.273171	1.952884	2.354702	6.089221	4.766151	5.319102	1.201052	1.565272
gene-AT4G	29.69199	28.68432	27.7717	23.62373	24.78	22.16011	9.393859	12.92394
gene-AT3G	2.361445	2.649125	2.664088	1.827313	1.813318	2.000171	4.044618	4.284119
gene-AT4G	6.400666	6.51952	5.030545	5.637391	6.795268	5.929463	1.768231	2.308987
gene-AT5G	10.65338	10.42657	11.58532	21.86802	23.30934	22.50743	3.156394	2.289712
gene-AT3G	39.55809	41.56162	39.20791	30.54185	29.55047	31.74308	61.39726	62.772
gene-AT3G	0.837985	0.708286	0.661656	1.110355	1.73248	1.112473	0.351628	0.33659
gene-AT5G	2.818756	2.159901	2.290793	2.339775	2.404322	2.471767	5.373227	5.419357
gene-AT4G	0.090081	0.129499	0.306017	0.722624	0.762791	0.694689	0.352078	0.237258
gene-AT3G	17.8156	15.69126	17.2008	22.76017	23.13603	23.68945	9.421322	10.19328
gene-AT5G	12.0767	12.52963	12.28364	22.53613	21.83641	21.8723	9.883612	12.30536

gene-AT5G	23.41314	23.66249	23.85634	17.9171	16.11589	15.32553	29.04159	30.96269
gene-AT5G	0.97237	1.925623	1.723577	2.995618	1.885661	1.931267	0.662484	1.032484
gene-AT4G	3.117235	2.247694	3.088455	1.744436	2.090875	2.265351	1.667823	1.603185
gene-AT1G	6.267839	6.787787	5.604278	3.513296	4.41855	3.035654	9.624901	8.265309
gene-AT3G	0.388773	0.272219	0.331682	2.349538	1.970857	1.196778	0	0.341603
gene-AT5G	1.885922	2.022597	2.008637	1.135519	1.013805	1.245229	2.604623	2.763121
gene-AT5G	0.95985	0.733216	0.773068	0.650683	0.870083	0.646287	2.058341	2.078472
gene-AT2G	17.08058	13.90353	13.14269	19.88515	15.3468	19.01654	24.54408	29.40697
gene-AT1G	2.936852	2.877512	2.829986	5.563251	3.789574	4.55574	0.73118	0.877799
gene-AT5G	45.47095	45.64626	43.34871	31.08719	29.81072	32.58532	14.24524	14.80228
gene-AT5G	1.141013	2.346445	2.095099	1.872652	1.461603	2.796101	4.251349	2.907229
gene-AT1G	19.16346	18.34872	20.11586	20.48892	20.80533	19.11259	8.683322	8.984544
gene-AT5G	0.982607	1.4687	1.401837	0.78427	0.594118	0.688978	2.24911	2.650074
gene-AT2G	67.45992	67.81014	68.33207	61.94505	54.64901	61.53212	12.72593	15.46993
gene-AT1G	166.9745	165.486	164.8388	136.3647	141.3816	136.8046	290.8048	293.2681
gene-AT4G	46.60503	46.39792	49.03932	29.28272	30.15428	30.25836	38.22801	39.24059
gene-AT3G	1.794956	1.867628	1.228484	2.925152	2.486446	2.613382	1.3185	0.996655
gene-AT2G	9.356	8.062889	7.115009	20.12398	20.5662	22.19478	8.009178	6.997319
gene-AT4G	6.499077	5.487915	6.393802	8.011118	7.157001	7.056001	7.926638	7.23478
gene-AT3G	0.642506	1.774063	0.287767	3.052711	2.323984	3.858774	0.442819	0.729837
gene-AT4G	16.94955	16.12445	15.07495	16.51023	19.56125	16.13953	20.26278	19.44791
gene-AT1G	1.248101	1.071684	1.455325	1.198056	1.121413	0.642302	1.317121	1.253805
gene-AT2G	620.0544	710.787	540.2836	1110.209	1142.422	1222.181	406.6383	339.7651
gene-AT1G	9.031447	8.403473	9.34225	4.685935	4.455356	4.525943	1.629598	1.42927
gene-AT4G	12.88199	11.42147	13.47482	8.771293	7.39682	8.669809	20.51116	19.01487
gene-AT1G	6.386906	5.640092	5.905949	8.560854	8.830389	9.145739	5.293862	6.164371
gene-AT3G	0.814021	1.046653	0.94678	2.034633	1.653045	2.466877	0.361445	0.053825
gene-AT3G	0.483249	0.206405	0.210947	0.503384	0.553987	0.344115	0.107272	0.327824
gene-AT5G	0.646134	1.299779	0.483038	0.260802	0.072571	0.064141	1.666348	2.269779
gene-AT1G	13.09607	12.04113	12.78414	5.749463	6.107334	6.268144	8.073825	7.181286
gene-AT4G	5.782016	5.039573	4.931261	8.274399	8.000656	8.299206	11.97827	10.23387
gene-AT2G	2.938659	2.364412	2.887213	3.140017	2.982226	2.92249	1.590482	1.814748
gene-AT1G	16.20615	16.33092	16.12709	11.8609	11.18512	11.4514	16.1623	15.81949
gene-AT2G	10.25169	9.680555	9.973953	14.87624	14.64511	13.91865	9.223661	14.02333
gene-AT3G	6.479177	7.196778	6.471393	9.70776	9.26205	8.544481	2.773379	2.339648
gene-AT1G	5.570916	4.599724	6.576946	7.066562	6.323493	7.039413	3.471968	2.485702
gene-AT2G	3.957045	3.45887	3.863225	2.130144	3.059254	2.96419	1.334146	1.855422
gene-AT4G	4.272417	4.746243	4.264882	12.68937	14.74482	14.29626	3.60694	2.108745
gene-AT4G	14.56108	16.30905	16.63862	30.68188	27.83306	29.42079	8.33629	9.48033
gene-AT2G	3.334001	4.956893	5.324556	13.0999	15.15072	12.65278	1.9619	2.685014
gene-AT5G	1.340914	1.63245	1.325712	1.672811	1.722212	1.352375	0.603803	0.454521
gene-AT5G	27.22579	25.94363	27.26171	20.26536	22.11102	22.18728	21.0774	21.91494
gene-AT2G	0.948778	1.279296	1.370593	1.047464	0.645865	1.19258	2.812539	3.456555
gene-AT2G	0.5073	1.00862	0.623836	1.782227	2.023572	2.347634	0.778063	0.606242
gene-AT2G	45.00979	42.15418	43.20371	34.01793	31.77536	34.96638	61.9605	60.60478
gene-AT5G	1.509876	1.250726	1.451671	1.234802	1.294743	1.033268	2.486545	2.152742
gene-AT1G	3.247479	3.844792	4.120944	3.034509	3.40796	3.576281	6.833296	6.989644
gene-AT2G	14.70715	14.84643	14.36869	9.710325	10.26599	11.92993	4.019005	4.659944
gene-AT1G	2.165148	1.801837	1.640508	2.931564	2.339859	2.425841	0.928542	0.980294
gene-AT1G	9.003441	9.035793	8.973931	8.058654	9.067487	8.921029	3.636699	4.244158
gene-AT5G	16.05483	14.26297	16.61507	13.16502	12.06508	12.14951	5.89375	6.223263

gene-AT5G	6.055319	6.594903	5.412841	3.711195	3.972771	3.147644	2.478438	2.286888
Arabidopsi	2.49226	2.433459	2.541064	1.891761	1.405546	1.580117	1.16099	1.188271
gene-AT5G	126.8465	123.427	127.5245	86.96305	87.84764	87.53741	35.8797	37.05698
gene-AT3G	3.912624	4.11727	4.626282	6.08637	5.855258	6.232728	3.72564	3.754433
gene-AT2G	21.9522	23.44697	25.4913	23.53028	19.67305	20.51448	35.65052	37.92588
gene-AT4G	3.451135	3.206448	3.106462	2.384062	1.952452	3.117503	5.532018	5.855425
gene-AT2G	0.524266	1.058905	1.104479	0.858976	0.764814	0.76339	2.047675	2.393052
gene-AT1G	8.278636	8.158648	8.17901	4.261986	4.670125	4.429717	1.364854	1.217023
gene-AT4G	1.810817	2.259207	2.540149	2.186858	2.08235	1.959949	0.273852	0.122018
gene-AT1G	362.4831	347.8598	365.7802	468.9038	458.4762	457.108	193.8155	198.8094
gene-AT5G	3.049614	3.704468	3.400795	1.08508	0.864679	0.821817	0.7371	0.406498
gene-AT3G	2.77002	2.571223	2.244144	4.4488	3.903186	3.733803	0.842207	0.944402
gene-AT1G	5.483699	4.22093	5.513515	5.414992	4.851832	6.366099	0.86978	0.700383
gene-AT5G	15.16698	15.71951	16.51884	11.53306	12.32044	12.13787	6.290338	6.627319
gene-AT4G	4.471108	3.525779	4.680868	5.534182	4.635056	4.850489	5.098528	5.861804
gene-AT4G	0.656241	0.533375	0.790494	1.585628	1.73622	1.877497	1.078641	0.9326
gene-AT3G	0.938263	0.664148	0.613781	0.291717	0.299848	0.181576	1.758982	2.081726
gene-AT1G	6.979335	7.244949	6.6772	6.655957	5.882953	6.667232	1.22174	1.542005
gene-AT3G	14.24276	18.26369	15.68477	16.91628	17.52075	18.82665	24.66441	23.33181
gene-AT5G	73.19463	70.19332	72.17073	46.35924	47.23534	46.36962	93.76847	95.45866
gene-AT1G	0.96464	0.513189	0.628636	0.870144	0.868319	0.859717	0.727592	1.060566
gene-AT5G	26.73026	31.85535	28.98877	16.9034	16.57141	15.28437	11.13571	12.38255
gene-AT5G	29.99823	30.46063	29.56933	28.99021	25.2298	29.49557	31.57167	32.11901
gene-AT1G	22.46097	21.74092	22.51117	16.75774	15.56758	15.05297	29.93394	31.22266
gene-AT5G	103.5845	103.6613	104.221	63.36479	65.50069	60.9777	121.6187	121.9539
gene-AT5G	7.387441	8.300227	8.252752	6.625086	6.922981	6.148942	2.672403	2.947444
gene-AT2G	0.58441	0.532032	0.668235	0.233272	0.20205	0.162783	0.156616	0.031637
gene-AT1G	5.500931	5.282145	4.647773	3.949638	3.003764	3.730913	7.147053	6.93197
gene-AT4G	39.71992	42.84397	42.12211	40.37769	45.1017	40.26462	52.25851	51.64216
gene-AT4G	14.0354	11.91718	12.68154	9.821765	9.224141	9.212437	12.13921	13.5911
gene-AT5G	6.036746	4.574651	6.111093	7.033966	8.37449	7.67473	1.692341	2.117533
gene-AT5G	21.52	20.8529	22.1413	99.7759	101.3334	101.1389	26.96412	29.03788
gene-AT5G	38.05953	34.9438	33.16283	31.42076	32.9914	33.9565	66.76744	65.08999
gene-AT2G	191.2358	187.1997	184.8127	118.2491	105.7955	116.6837	241.2145	250.7975
gene-AT5G	4.094303	3.157459	2.626217	4.331933	3.487091	2.955818	0.694612	0.944188
gene-AT2G	1.836826	2.021541	1.911993	1.445805	0.948125	1.209107	5.01968	4.330436
Arabidopsi	4.003049	5.713205	4.065447	1.704813	3.154921	2.699631	7.648062	8.177597
gene-AT5G	12.87354	12.23313	12.60332	57.97161	61.99947	61.11355	4.095795	4.798449
gene-AT2G	20.19271	19.32827	20.22447	40.48498	37.35929	39.16505	9.170269	9.979162
gene-AT1G	6.790776	7.058396	7.278133	5.07791	4.955391	6.567922	3.378515	3.852909
gene-AT1G	149.3866	143.1754	151.723	55.84519	60.15731	55.63241	116.0429	118.1224
gene-AT3G	18.67224	16.35619	15.96022	22.43831	19.38218	20.70635	5.373175	5.512424
gene-AT1G	6.475562	5.933938	7.796278	4.793191	5.650301	4.807348	8.917167	10.04403
gene-AT3G	12.33922	12.69221	13.01084	8.727282	8.321954	7.858004	9.837724	11.22981
gene-AT1G	79.31657	78.87666	79.53079	42.11478	40.83281	40.9286	122.5387	127.5398
gene-AT2G	37.75945	32.78443	37.19616	33.74333	34.0492	35.0734	43.88356	43.67713
gene-AT2G	0.195081	0.107029	0.12803	0.418722	0.596599	0.286838	0.375355	0.154156
gene-AT1G	9.491896	8.537283	9.144043	21.30937	21.4206	20.32972	8.913767	7.964513
gene-AT2G	10.09367	9.298612	9.625683	5.552536	6.03907	5.673613	11.34637	12.90883
gene-AT1G	1.662028	3.336815	2.163418	0.333592	0.640662	0.560644	2.717487	3.116883
gene-AT1G	95.57101	94.29593	97.48817	79.16782	68.58653	71.46886	89.73715	94.11552

gene-AT4G	41.21684	46.22836	44.56712	17.6183	18.00615	16.18784	46.75805	40.19877
gene-AT4G	82.11666	81.93173	76.09937	62.5847	62.49814	60.79684	138.4508	133.1558
gene-AT1G	0.304006	0.396691	0.329949	0.812441	0.698394	0.727372	0.483179	0.404296
gene-AT4G	7.476727	5.707682	6.962622	7.781412	7.888029	5.352293	8.236459	8.087434
gene-AT5G	4.013877	3.385263	4.450714	2.582009	3.429031	3.836277	0.701933	0.560488
gene-AT3G	20.27665	20.09338	20.24728	18.10847	18.12216	18.44093	28.39822	27.95945
gene-AT3G	919.3613	895.446	932.6257	456.1518	453.5345	443.3786	1424.776	1504.628
gene-AT1G	2.119191	2.170099	2.368359	4.765282	5.889124	4.431959	1.147206	1.779596
gene-AT5G	0.308739	0.476913	0.538781	0.886031	0.662629	1.137032	0.370986	0.25402
gene-AT5G	20.61907	20.74934	20.87571	16.9054	16.6363	16.0227	9.411962	10.55292
gene-AT1G	1.011113	1.645265	0.409103	2.138194	1.564403	1.433012	1.167864	0.937045
gene-AT1G	0.505624	0.370596	0.495609	0.245498	0.155472	0.159365	0.283436	0.370788
gene-AT5G	33.84829	31.07452	31.03364	33.16593	30.8871	27.09784	30.14329	32.91393
gene-AT3G	785.8591	801.9742	797.2651	286.9418	294.002	294.0622	1236.265	1208.287
gene-AT3G	3.527405	3.093432	1.910418	6.063477	5.241688	4.396131	0.309887	0.613181
gene-AT2G	0.639211	0.390197	0.577806	1.660787	1.568318	1.471874	1.386992	1.173979
gene-AT5G	92.29862	89.20653	96.10018	71.44595	64.10595	65.08054	116.4003	119.5932
gene-AT5G	12.35965	12.15794	10.59082	14.02422	15.45036	15.83157	7.433754	9.861662
gene-AT3G	1.347274	1.437456	1.264694	0.927823	1.796404	1.386528	0.360143	0.434719
gene-AT3G	37.58226	38.75623	38.01141	21.13623	21.7976	20.19397	33.85145	36.117
gene-AT1G	2.45992	2.38338	2.342508	7.972903	7.802358	7.755536	2.633072	2.815939
gene-AT1G	7.339802	6.603623	7.980924	3.586009	3.595478	4.321703	2.37922	2.838957
gene-AT1G	6.203971	6.002887	6.594432	15.65156	14.84274	15.90321	6.231972	6.427445
gene-AT4G	9.991045	11.78062	9.781506	18.28582	17.61581	15.4756	7.195181	5.623842
gene-AT3G	1.888546	1.403188	2.476456	1.407449	2.023278	1.743218	1.340742	1.453273
gene-AT1G	1.149654	1.283931	0.99095	2.784169	1.658806	1.732446	0.483094	0.399117
gene-AT2G	38.52628	38.55155	43.57325	39.85772	40.78741	41.48882	52.76039	55.66916
gene-AT1G	158.3359	145.9943	153.6483	91.48004	87.58303	81.64083	286.722	313.8248
gene-AT4G	38.27696	33.857	37.52383	51.91762	52.90564	48.82886	15.22026	15.78497
gene-AT1G	6.534707	7.162827	6.174543	9.019706	10.0855	12.36106	2.79211	2.681832
gene-AT1G	1.876165	2.767123	2.045043	2.416916	3.365022	1.936427	0.806646	0.821388
gene-AT4G	3.634602	3.318393	4.433058	4.555635	3.552143	4.258049	0.9903	0.648157
gene-AT1G	20.91286	20.24057	22.4413	11.33966	12.02264	10.58298	41.01375	46.31325
gene-AT3G	60.97947	60.12943	62.28574	47.58864	46.66208	45.44084	85.06966	89.08556
gene-AT4G	8.047161	7.413278	7.66364	3.430144	3.513281	3.463618	3.918968	3.491681
gene-AT3G	16.41583	16.92559	17.68802	15.50021	15.3047	15.89521	20.97582	21.71924
gene-AT3G	5.461236	6.190803	5.75913	3.700279	4.181189	3.600697	14.72854	15.77354
gene-AT2G	66.43205	64.10426	71.95508	62.38378	61.2682	62.25169	85.89738	89.29203
gene-AT4G	18.27861	18.57295	18.45004	13.16319	12.78204	14.25154	25.99448	25.56734
gene-AT3G	28.47002	31.56014	26.09761	83.67667	74.68392	75.77112	25.58962	24.87991
gene-AT2G	70.01426	67.98453	71.31205	95.70275	94.61889	97.10127	105.0337	103.9315
gene-AT5G	50.14328	48.70952	51.04548	39.91939	38.7827	39.46318	60.70429	65.8725
gene-AT5G	7.216521	9.37993	8.654721	17.70356	21.17466	19.08166	11.3965	11.19784
gene-AT4G	0.973013	0.720081	0.527276	1.335332	1.500602	1.676229	0.274333	0.399207
gene-AT3G	15.74478	17.14239	17.64326	17.77945	16.97977	17.83885	6.499101	7.205234
gene-AT1G	10.77697	10.31643	11.62988	13.62568	15.35334	14.99025	7.345212	7.324544
gene-AT3G	56.37514	54.18464	53.55273	42.80648	46.83576	48.60165	80.76148	86.52853
gene-AT2G	0.216538	0.132556	0.067178	0.119382	0.505294	0.060672	0	0.247852
gene-AT2G	3.461219	2.594958	3.833951	2.808543	2.141948	2.152918	3.989158	4.923474
gene-AT2G	6.425911	5.752645	5.303263	4.255326	5.64178	3.627085	7.553782	6.972583
gene-AT4G	4.351242	3.496267	5.000601	7.228852	7.192391	7.924044	8.420081	5.694906

gene-AT3G	17.09251	17.36399	18.20445	22.91959	22.72769	23.38579	23.83687	24.92921
gene-AT4G	11.56876	10.00099	10.8545	9.120092	9.832742	9.145195	22.91505	21.90739
gene-AT2G	0.425813	0.224597	0.231965	0.466257	0.540697	0.611156	0.9875	0.233695
gene-AT5G	5.386757	4.753147	5.433768	3.113527	2.950438	3.609547	11.26956	9.946786
gene-AT5G	14.18578	11.95428	14.58602	12.54789	12.03277	13.15478	22.92659	25.12108
gene-AT1G	5.835006	4.734197	5.024465	6.737191	6.636683	6.303292	3.204548	3.553498
gene-AT3G	7.374196	5.850433	5.590198	11.39654	9.461832	10.9518	15.42643	16.05443
gene-AT3G	0.990314	1.051455	1.196071	1.382846	1.156937	1.374981	2.105927	1.531843
gene-AT1G	1.855473	1.622769	1.490801	1.054462	2.064736	1.496171	5.564734	4.715713
gene-AT2G	2.819409	2.398252	3.706004	1.491229	2.582088	2.461064	6.665349	6.294349
gene-AT5G	47.03881	45.57107	47.7165	19.77171	19.39184	19.93304	12.03341	14.02865
gene-AT5G	0.893208	0.984244	0.783224	1.046294	0.870201	0.903347	0	0.322542
gene-AT1G	0.307632	0.38595	0.132905	0.477335	0.743768	1.220869	0.538722	0.479495
gene-AT2G	0.272802	0.197417	0.097243	0	0	0.077836	0.019558	0
gene-AT1G	2.944931	4.027491	2.917736	2.669385	3.924633	4.617502	6.662337	5.54037
gene-AT1G	783.6026	786.6204	799.6424	382.6951	389.5115	387.8348	1724.682	1758.144
gene-AT4G	3.317979	2.548559	2.773538	2.270852	4.605694	2.881025	5.916074	5.199506
gene-AT3G	8.387747	7.901363	6.819718	3.334312	3.555003	3.443428	4.130612	3.261199
gene-AT5G	11.2935	12.70035	14.33481	12.44527	10.26198	12.53383	3.447585	4.345225
gene-AT2G	8.089089	8.524127	8.014676	21.00589	21.95511	18.56245	4.25915	3.979581
gene-AT5G	13.78899	14.67539	14.94609	25.60293	23.62778	25.27492	4.880031	6.48875
gene-AT5G	5.433857	5.409002	5.838397	2.68562	1.948594	1.722923	6.23803	6.455358
gene-AT5G	3.741264	5.364058	4.916783	3.018642	1.272665	3.105386	7.593239	6.722847
gene-AT5G	0.844985	0.66626	0.91274	0.410684	0.505676	0.283006	1.681464	1.489783
gene-AT1G	1.813368	1.614961	1.676532	2.483348	1.728761	3.254529	1.157195	1.506801
gene-AT4G	4.013711	3.363564	3.556629	2.285098	1.243475	1.69229	6.728111	6.345376
gene-AT4G	12.75143	11.84142	11.90552	6.153789	6.101156	6.512779	9.89388	9.71098
gene-AT3G	11.47704	12.27402	10.7783	19.0128	12.5077	15.74667	20.8132	22.56587
gene-AT1G	25.36885	23.51117	24.42431	11.50041	12.6978	12.37105	46.50383	49.53341
gene-AT4G	13.73539	14.06727	15.19007	11.19269	10.34378	9.465318	18.07671	19.2085
gene-AT3G	25.92598	23.30182	26.6896	23.32075	24.26653	23.86222	26.13956	26.25382
gene-AT1G	14.42484	13.2811	13.99879	7.716048	7.505243	7.558117	15.70507	16.71261
gene-AT2G	24.58953	24.60576	25.0306	18.24635	18.81127	18.73118	30.81026	29.7407
gene-AT5G	13.36886	15.13061	15.31191	11.89588	11.9492	10.25278	41.81622	34.58239
gene-AT5G	0.434913	0.1595	0	0.63839	0.417977	0.696443	0.319774	0.206584
gene-AT2G	6.027102	5.812105	6.586292	5.740233	6.462245	7.073871	9.704574	8.008025
gene-AT4G	12.30013	13.66638	11.41078	29.26914	26.67578	28.15204	26.76106	30.56996
gene-AT3G	7.93918	10.20983	8.059674	14.81502	13.61261	15.34074	6.199609	8.221647
gene-AT3G	0.39449	0.943912	0.51393	0.291608	0.247669	0.4465	0.945997	0.813917
gene-AT1G	0.652395	0.571197	0.4428	0.36593	0.481483	0.309835	0.039244	0
gene-AT1G	1.65034	1.651298	1.896771	0.806534	0.717114	1.021632	0.691939	0.771222
gene-AT1G	19.54376	18.47177	20.10699	13.50992	12.62417	11.07687	7.603783	7.385745
gene-AT4G	3.344014	2.831634	3.648299	1.919295	2.393267	2.841915	5.926574	4.577749
gene-AT4G	7.97701	8.164492	7.675161	10.8215	11.40616	10.97604	5.735708	6.655904
gene-AT1G	6.586256	8.274394	7.822121	12.59618	11.69808	12.38248	3.540994	3.027524
gene-AT5G	3.200341	3.744911	3.545495	4.708302	5.734882	4.790856	4.402586	4.028007
gene-AT5G	8.259137	7.527847	7.643946	12.46423	10.06062	13.12676	7.522826	6.374233
gene-AT4G	1.228143	1.11886	0.693031	0.615396	0.321054	0.603996	0.442087	0.227383
gene-AT3G	48.45576	49.5059	51.32826	37.38973	34.22551	32.77938	76.55595	79.66728
gene-AT1G	3.386232	3.059859	2.991579	2.280582	1.407077	1.596828	0.501959	0.976341
gene-AT5G	19.00883	19.35573	18.1087	11.32487	10.11697	11.99807	29.38086	31.44404

gene-AT3G	20.93445	19.25357	19.80751	33.965	34.38283	35.3713	22.7926	22.38913
gene-AT3G	19.70181	19.38356	21.23718	26.5189	24.66548	24.31117	16.06685	17.15266
gene-AT5G	1.320125	0.941742	1.562105	0.164546	0.439874	0.224371	0	0
gene-AT3G	0.648528	0.878685	0.918865	1.087976	1.345451	1.042371	2.07735	2.075615
gene-AT1G	0.772454	1.332855	1.293001	3.123489	2.571264	2.796883	1.421332	1.143153
gene-AT1G	0.726721	0.888947	0.657356	3.012524	2.336848	2.90643	0.329441	0.445854
gene-AT3G	4.009736	3.645608	3.436778	2.641669	3.298706	2.678143	1.641711	1.618957
gene-AT1G	0.276649	0.372783	0.721594	0.60882	0.562332	0.608899	0.378214	0.396597
gene-AT2G	6.8606	7.079365	8.038382	6.66208	6.623395	6.011317	14.93196	14.27745
gene-AT2G	7.154418	5.499792	5.993898	6.902262	5.718114	6.475571	4.724942	5.459857
gene-AT1G	1.323068	0.846778	0.608627	0.37691	0.458587	0.452115	0.09003	0.018986
gene-AT1G	0.400759	0.420084	0.510491	0.809883	0.385988	0.896108	0.03304	0
gene-AT4G	1.2228	0.685027	1.474209	1.522977	0.965461	1.481216	2.630157	1.977976
gene-AT5G	9.095278	8.766537	8.395645	1.952848	2.699326	2.298584	29.0961	30.94651
gene-AT4G	5.343623	4.59442	5.22202	2.655878	2.898958	3.157698	12.60597	13.66842
gene-AT2G	1.089376	1.216727	0.459822	22.99452	21.91316	23.37021	0.210565	0.77193
gene-AT3G	19.02144	19.07188	18.73447	4.521134	3.746759	4.63429	29.69363	33.08228
gene-AT5G	0.672904	1.563241	0.981959	2.656488	4.076122	4.415142	0.386461	0.341358
gene-AT5G	1.745916	1.445528	2.004885	1.797044	1.200198	2.065833	1.940849	2.453798
gene-AT1G	249.2565	252.2006	218.4039	132.5439	130.2989	132.9316	254.0535	258.8968
gene-AT2G	22.23297	22.55062	19.68716	16.06572	15.6254	17.55358	35.37572	33.61676
gene-AT1G	2.020298	1.935323	2.287903	4.393415	5.627401	4.846103	1.871832	1.536361
gene-AT5G	6.282913	5.856803	6.858243	11.90762	11.91325	12.34955	7.928309	8.974093
gene-AT4G	3.163637	3.041096	2.394043	2.360614	2.64775	3.438791	0.790392	0.911399
gene-AT1G	155.2652	154.429	160.0399	99.36787	96.14813	90.59564	279.9815	279.9544
gene-AT5G	1.937553	2.291506	2.516738	1.95202	1.087491	2.352688	9.279765	10.78444
gene-AT5G	3.177078	2.814096	2.630021	0.299438	0.482853	0.44569	0.695451	0.627823
gene-AT2G	21.45308	19.64903	20.0876	23.77144	24.48071	25.20775	27.49974	27.43177
gene-AT4G	2.02405	2.604148	2.430614	2.025789	2.459916	1.918598	0.397311	0.704103
gene-AT1G	73.90603	72.82118	74.5814	49.53416	48.97937	51.35732	86.48037	86.66497
gene-AT1G	0.355659	0.432581	0.407406	0.194093	0.470275	0.234875	0.641948	0.686728
gene-AT3G	54.22675	54.79964	55.78467	37.49628	36.26238	35.73748	20.51421	18.79655
gene-AT5G	1.847671	1.696899	1.871208	0.693125	0.652976	0.27556	0.781102	1.113791
gene-AT2G	1.393348	1.018762	1.476974	3.418525	4.720217	3.490749	2.865029	3.084724
gene-AT3G	6.494452	7.771139	8.123006	8.592382	11.10369	11.44603	17.58635	14.64929
gene-AT3G	8.935867	9.583955	11.27003	9.391514	8.784558	9.719297	13.11485	11.35604
gene-AT1G	19.11244	17.49679	20.4001	29.66455	28.20015	27.91521	10.39035	10.97137
gene-AT5G	341.9061	300.5623	353.4658	210.0841	209.5744	213.9524	544.9723	561.9111
gene-AT4G	1.045785	0.709925	0.679107	1.199155	1.314213	1.137409	1.203914	0.794614
gene-AT4G	168.3738	172.3561	178.6072	129.2197	129.6657	131.1133	303.84	305.6659
gene-AT1G	5.136323	5.131844	4.94858	11.79241	10.95663	12.32825	3.281965	3.436498
gene-AT5G	1.090277	1.830624	1.724563	0.343213	0.610942	0.697776	1.123404	1.349978
gene-AT5G	1.30597	1.186484	1.109447	1.607742	1.103869	1.342211	1.696533	1.5103
gene-AT1G	128.2993	126.924	127.1563	61.89521	63.49762	62.46694	12.88677	13.96148
gene-AT1G	9.305943	10.96952	11.79888	20.25405	19.66188	20.48234	9.669951	11.14814
gene-AT3G	43.34118	41.1477	41.64436	19.01737	17.12843	17.40338	17.20746	18.63357
gene-AT2G	4.163101	5.624928	4.596141	66.07826	67.55114	70.68294	0.479897	0.39623
gene-AT1G	0.824712	0.760919	1.186561	1.337899	1.023219	1.23789	1.536514	1.092566
gene-AT4G	6.380065	5.802821	5.891254	6.283573	7.409707	6.950397	8.143809	8.467553
gene-AT5G	12.12713	11.0369	10.50607	11.24254	9.705225	9.498029	2.462171	2.369378
gene-AT5G	4.682342	4.127816	5.390112	2.518256	2.606354	2.159622	12.31618	10.68661

gene-AT5G	17.61079	18.69211	15.80866	15.05749	15.20072	15.11176	7.424667	6.675105
gene-AT1G	32.79361	31.43343	36.11113	5.184363	6.242381	5.382589	0.206366	0.499381
gene-AT5G	55.67645	52.15791	52.7678	49.42472	49.47805	51.3985	87.38965	90.91779
gene-AT1G	4.295147	4.727343	3.944392	3.817099	3.811996	4.397534	4.468772	4.298848
gene-AT1G	1.43996	1.441793	1.400154	0.333031	0.525871	0.36346	0.705177	0.170284
gene-AT3G	5.90283	4.11993	3.212974	1.893663	1.426548	1.098208	2.583292	2.956834
gene-AT2G	30.29912	29.57102	31.11165	29.65628	32.27944	32.22488	40.24599	44.9123
gene-AT3G	20.49235	21.14112	21.44368	14.26458	13.31645	11.84624	4.679005	4.774491
gene-AT5G	66.55277	66.228	68.13735	69.68698	69.97781	68.59093	28.81406	29.97574
gene-AT1G	0.362434	0.488068	0.42813	1.274487	0.950446	0.734788	0.362792	0.333937
gene-AT2G	1.962573	2.249319	2.272285	0.848946	1.149736	0.864222	2.120203	1.994846
gene-AT4G	20.35449	17.92474	18.37353	16.46878	19.06148	16.32964	20.19151	20.56138
gene-AT3G	6.711324	6.759814	7.088984	4.867844	6.591175	5.991185	11.34102	10.86619
gene-AT1G	240.7986	234.4814	235.667	307.3885	307.6559	309.0146	46.52941	46.43369
gene-AT4G	12.28943	12.56122	13.29838	10.27537	10.79918	10.16677	15.25655	16.38502
gene-AT3G	38.75188	38.17469	38.56078	65.56352	62.47208	65.63836	24.3633	23.6593
gene-AT4G	4.686067	3.540443	4.13435	3.335431	2.987249	3.188471	1.443738	1.097228
gene-AT3G	25.55147	22.75869	23.69425	20.7859	22.07288	22.15434	45.24974	52.38889
gene-AT3G	0.721057	0.940499	1.380149	1.968184	2.154512	2.020995	0.850598	0.803873
gene-AT1G	5.281516	3.356191	5.506732	3.430154	2.983466	4.116392	4.715057	5.158159
gene-AT5G	22.71726	22.36455	25.70618	19.03171	16.61273	18.42418	35.19059	33.48377
gene-AT5G	27.39704	26.39566	28.12829	9.4928	10.75792	9.912785	7.915714	8.841857
gene-AT3G	3.559388	4.90009	3.896249	5.401688	5.381039	4.319144	11.56322	12.88504
Arabidopsi	5.895567	5.954571	4.363783	8.771159	8.45599	9.224608	3.959149	3.322808
Arabidopsi	0.613595	1.0024	0.446464	0.291617	0.341008	0.890073	0.84574	0.33246
Arabidopsi	1.173908	1.057159	1.209935	0.660818	1.197729	0.890042	1.826818	1.595901
gene-AT1G	2.981282	3.36664	2.231793	2.216501	3.185389	2.345855	5.782481	4.832191
gene-AT3G	158.1067	152.7254	162.9474	180.7911	176.0757	168.8718	264.8934	274.0389
gene-AT5G	4.58848	4.593529	5.402552	5.236625	5.645905	4.969408	5.736841	6.725476
gene-AT3G	0.382057	0.732391	1.502082	1.507915	1.223957	1.219039	1.786662	2.049224
gene-AT2G	13.30751	12.70564	13.85319	10.00621	11.55763	11.19907	4.070424	3.754878
gene-AT1G	73.81493	72.76679	74.25842	20.92616	22.31509	23.98557	43.82787	45.92025
gene-AT4G	0.131333	0	0.12634	1.754734	1.190512	1.53389	0	0.733832
gene-AT1G	7.101736	5.824734	6.458468	5.369292	5.780032	5.385154	6.199876	6.765924
gene-AT4G	3.860273	2.906802	4.431452	3.718719	4.20642	3.458229	7.453485	8.581224
gene-AT2G	4.784545	2.618623	4.752818	6.738464	4.70431	4.431918	5.751335	6.469736
gene-AT2G	0.526436	0.351461	0.536162	0.125071	0.13398	0	0.052911	0.051128
gene-AT3G	2.021106	1.519971	1.682251	3.909479	3.483311	3.892427	0.205773	0.231274
gene-AT4G	0.897948	0.818541	0.880767	0.214942	0.355272	0.237274	0.136259	0.230698
gene-AT4G	18.42729	18.45089	20.79254	14.92423	14.98363	14.64032	27.47741	27.62665
gene-AT4G	4.477521	2.397067	3.186432	2.095321	1.509522	2.036944	3.868637	4.219197
gene-AT2G	3.111109	2.521904	2.550683	0.50615	0.201016	0.915616	0	0.035757
gene-AT2G	0.331079	0.197242	0.165106	0.116017	0.09701	0.113779	1.472672	1.556836
gene-AT1G	41.63554	40.55565	44.64119	82.26437	90.24649	83.70033	50.52187	52.81387
gene-AT2G	59.57925	49.90694	54.31737	46.2626	44.14332	44.40812	72.33157	67.33319
gene-AT1G	4.690286	4.824571	4.126006	3.466855	2.473285	2.443947	6.041437	5.443739
gene-AT4G	5.092273	4.20243	4.185325	1.698695	2.065386	1.634685	4.178535	4.996105
gene-AT4G	523.9338	525.0102	537.6152	282.8349	288.9315	283.5018	833.2572	837.7217
gene-AT5G	20.565	23.63504	24.5816	24.24292	25.30605	25.08413	25.48793	27.67895
gene-AT1G	22.4182	22.99508	23.09491	13.07588	11.8695	13.84184	31.30314	35.48586
gene-AT2G	3.121422	3.592559	3.047867	0.83118	0.753571	0.724398	6.439071	7.010168

gene-AT1G	162.2538	163.5881	170.731	187.9568	194.5277	192.2919	72.19527	86.57066
gene-AT5G	3.954685	3.596742	3.583913	2.395095	3.09855	2.65748	2.121862	2.504538
gene-AT5G	9.36162	8.201777	9.082075	10.64491	9.229806	9.489852	2.397554	2.394192
gene-AT5G	3.325092	2.019518	2.680133	4.231546	3.610428	3.526438	1.133617	1.08347
gene-AT2G	0.257714	0.489318	0.562629	7.235216	7.489619	7.198207	2.325524	1.754723
gene-AT5G	4.629109	3.58651	4.899204	1.552037	1.812099	1.628646	0.914753	1.007267
gene-AT3G	76.09411	74.20976	82.55273	71.57719	77.07027	73.93198	12.32046	14.41376
gene-AT2G	19.09051	20.00215	18.12833	67.22643	72.64817	64.82271	2.751622	2.923027
gene-AT1G	9.349182	10.68982	10.57747	15.75268	17.16937	15.2929	8.169862	9.961121
gene-AT2G	3.477	4.765403	3.253876	2.814125	3.766664	2.515077	5.707141	4.850367
gene-AT3G	9.301092	11.63627	9.396682	12.02527	13.6021	14.7533	7.187842	8.078134
gene-AT2G	7.573358	5.912681	7.303022	2.890685	2.682306	3.008738	1.432699	1.556231
gene-AT2G	40.48331	40.01228	39.6015	56.44843	56.60624	56.31497	22.89127	24.42504
gene-AT1G	19.46151	20.8045	21.14775	26.80239	24.61985	24.49383	8.812978	8.847969
gene-AT3G	6.6594	5.3593	5.51653	5.72789	4.879524	5.078911	7.272465	8.263706
gene-AT5G	2.86328	3.258856	2.326071	5.357778	6.173171	5.578325	0.709008	1.040012
gene-AT1G	1.53441	1.282958	2.066361	2.979895	3.098738	2.38504	0.598131	0.648345
gene-AT2G	9.303421	7.79881	8.110349	7.578086	7.194734	7.55881	12.18344	12.78801
gene-AT1G	15.71627	15.88249	14.79506	21.69533	23.37821	21.60953	22.03908	24.1265
gene-AT5G	15.75015	15.21471	17.05129	11.05893	10.30283	10.08855	24.30959	24.80465
gene-AT5G	2.223207	2.825501	2.115693	2.973065	2.337415	2.668225	5.755026	5.82597
gene-AT3G	161.4332	157.3938	157.0422	118.1115	114.3456	110.9193	197.755	201.9013
gene-AT3G	0.212952	0.764683	0.73845	0.71955	0.625561	0.833606	1.214172	0.96034
gene-AT1G	4.228609	3.069589	4.018181	3.615551	4.422126	4.099173	0.532108	0.834241
gene-AT4G	0.796576	0.875324	1.17566	2.430403	1.712283	1.531444	1.867808	1.856753
gene-AT5G	0.985096	1.382188	1.325422	3.937469	3.818542	4.185797	1.75392	2.943155
gene-AT5G	25.58417	19.30978	23.69493	26.50136	24.25702	22.21648	6.798895	8.208258
gene-AT1G	31.45781	31.62703	34.58397	20.59887	20.26514	21.13721	38.42061	40.96931
gene-AT5G	31.34379	31.13808	30.92725	28.88009	30.92278	29.389	46.78248	50.51231
gene-AT4G	30.46455	29.25541	29.10444	30.3384	27.32797	28.56043	18.00443	20.32452
gene-AT2G	11.34382	10.57367	11.05801	9.672748	10.28413	10.15825	14.22031	14.64564
gene-AT1G	39.52008	39.30513	40.22337	24.86442	25.16525	25.89667	61.03981	65.75681
gene-AT2G	21.40647	20.50945	22.07606	5.895873	6.452739	6.061145	2.340116	2.984109
gene-AT1G	20.05703	19.8751	17.35865	15.24465	17.92717	18.79971	26.06325	27.00591
gene-AT1G	154.6185	146.7301	154.0212	18.62216	17.26685	17.81958	1.436544	1.75131
gene-AT5G	3.370832	3.659502	3.732718	4.587662	4.449924	5.017896	1.011879	1.515126
gene-AT5G	76.77007	74.54956	76.28929	40.4404	39.89544	41.67775	53.3219	54.94601
gene-AT1G	1.98244	1.877697	2.660138	1.268805	1.021123	0.940332	4.255325	3.114231
gene-AT3G	6.3901	6.10927	6.297418	3.876988	4.295014	5.101292	7.575742	6.849092
gene-AT4G	1.153699	0.804215	1.152853	2.158265	1.617946	1.45136	0.41394	0.680546
gene-AT4G	33.88406	34.10978	36.10205	31.18133	31.2905	30.09886	49.7012	51.23384
gene-AT3G	0.601229	0.57254	1.154486	1.028168	1.245533	1.170724	1.066754	2.061293
gene-AT1G	11.66148	12.2315	11.48077	10.17591	11.22005	11.00157	15.38326	14.59696
gene-AT3G	5.034379	4.853492	3.998505	4.09279	3.200485	3.130001	3.153928	3.925826
gene-AT2G	35.81742	34.28284	32.90986	26.95278	27.76882	25.6748	43.37683	43.68145
gene-AT1G	6.329335	5.378474	5.524985	5.25061	4.867718	4.556071	8.161811	9.588254
gene-AT1G	0.307613	0	0.14443	14.00228	13.20028	11.5635	0.28005	0.418708
gene-AT3G	0.083137	0.098286	0.194495	0.250298	0.27957	0.184375	0.31824	0.28132
gene-AT4G	2.278666	2.193246	2.355611	3.705489	2.622813	3.233831	1.367171	1.609691
gene-AT1G	69.63335	70.53375	68.2253	89.48246	87.63079	85.22047	23.69092	24.80802
gene-AT4G	4.539042	4.087771	4.586656	5.055958	3.832807	4.721661	5.10219	5.446168

gene-AT1G	1.225328	1.24131	1.20334	1.856016	2.319634	3.50429	2.664326	2.551933
gene-AT5G	9.114548	9.231175	9.435987	6.254893	7.588164	6.639464	14.76795	12.7563
gene-AT4G	402.096	398.6662	386.5043	226.2164	222.554	228.6756	781.5494	798.8557
gene-AT5G	12.94079	12.49534	12.72751	6.108362	6.14505	7.820487	19.48381	20.18593
gene-AT4G	15.87301	15.50763	16.33688	17.91336	17.25388	18.99362	24.12014	22.23554
gene-AT1G	6.176813	5.765864	6.644722	7.27432	6.748689	6.481819	4.175132	3.901726
gene-AT4G	405.9173	398.7709	410.945	360.9178	358.298	352.5428	74.35896	80.55163
gene-AT5G	2.741142	2.739842	3.475175	2.635047	1.320153	3.118911	6.974287	7.466816
gene-AT5G	4.612885	4.447587	4.216193	1.104569	1.23178	1.180049	0.843062	1.089685
gene-AT1G	5.356431	4.000003	5.547916	2.459907	3.558153	3.030041	1.73148	1.24674
gene-AT3G	12.14687	10.4391	10.71591	29.08642	30.07545	29.19256	7.550745	8.313967
gene-AT3G	0.42945	0.421876	0.463746	0.451258	0.336179	0.545286	0.108615	0.074957
gene-AT4G	2.517633	2.352751	1.916035	0.500016	0.637319	0.621369	3.023208	3.92011
gene-AT2G	11.51248	12.40904	11.88428	7.762336	7.412898	7.961935	3.959635	4.636321
gene-AT2G	7.408848	8.10713	9.047213	15.03451	14.78426	13.39385	3.710323	4.078157
gene-AT5G	7.907558	7.417334	6.307829	6.94043	6.995158	6.978879	4.886176	6.019484
gene-AT1G	143.1371	138.2856	149.7854	93.05593	90.48529	92.16529	253.5273	256.5565
gene-AT1G	2.948173	3.826932	3.738926	3.392555	3.671136	3.430789	0.906484	0.910998
gene-AT1G	1.32462	0.870615	1.600721	2.187231	2.452732	2.327527	0.73811	0.77484
gene-AT1G	46.80227	47.76479	48.22127	12.01724	12.7286	12.3632	95.36604	93.62963
gene-AT2G	0.574855	0.901823	0.813797	1.471825	1.593393	1.214765	0.507186	0.431885
gene-AT1G	2.935387	4.429754	3.969375	3.4238	2.79986	2.733334	0.856817	0.981786
gene-AT1G	1.431325	1.172948	0.839379	0.105794	0.499846	0.275432	9.597548	9.779526
gene-AT5G	170.5966	160.7164	169.9863	130.6171	131.85	135.0538	264.9301	277.1505
gene-AT3G	6.543463	5.847549	6.716856	6.884376	6.677404	6.224662	10.57109	11.69648
gene-AT2G	11.46452	10.90352	12.15122	24.95481	24.19118	23.39581	10.24659	10.60508
gene-AT2G	1.274073	0.963625	0.792395	1.042328	0.898628	0.846277	0.567087	0.681993
gene-AT4G	4.889145	6.096083	5.699979	7.146692	5.159885	4.509264	8.147695	5.13659
gene-AT4G	6.904286	5.769495	7.170028	7.238068	7.02063	6.139807	5.116561	5.766758
gene-AT2G	44.64093	50.22667	50.06389	279.4806	297.1633	281.4853	43.22291	36.83158
gene-AT2G	182.5063	198.0503	175.7417	170.1934	184.1602	194.5553	75.0035	71.42329
gene-AT4G	0.247878	0.606581	0.405518	1.562094	1.937453	1.913971	0.900093	0.527156
gene-AT1G	20.99654	20.08626	20.66196	16.84051	16.44142	15.87308	21.76429	20.12874
gene-AT2G	10.1112	9.5585	10.65917	6.364357	8.336267	5.265142	10.88226	11.77112
gene-AT3G	42.03858	41.07583	40.51625	29.57118	29.90843	30.20334	41.38344	44.62485
gene-AT2G	1.322618	2.334841	1.329184	1.187412	1.007217	0.87282	3.573066	1.623273
gene-AT2G	36.04059	37.0642	39.7616	18.38194	18.64893	18.15791	51.45122	53.16213
Arabidopsi	1.070431	1.277958	1.661802	0.610203	0.255711	0.150122	0.466911	0.766566
gene-AT1G	24.78445	23.85922	25.92085	14.22916	13.26063	13.02491	22.73284	23.31398
gene-AT2G	79.99188	76.28422	79.51254	42.34337	40.8957	39.27586	101.5396	104.4481
gene-AT5G	0.374202	0.598677	0.308012	0.755052	1.094658	0.549964	0.285026	0.070531
gene-AT5G	3.913035	4.018239	4.070534	6.504688	5.643731	5.042755	2.547336	1.222277
gene-AT3G	0.255817	0.109566	0.39714	0.122946	0.308294	0.325758	0.41923	0.440202
gene-AT1G	61.68913	64.89989	62.10848	35.42751	34.43147	32.40767	93.70798	100.5211
gene-AT5G	1.319945	0.739411	1.05814	0.673028	0.841845	1.092407	0.660247	0.923318
gene-AT4G	6.877181	6.742775	6.458452	5.914513	6.019094	6.941195	5.586118	7.1086
gene-AT2G	145.5519	150.1443	145.2616	135.8114	132.3156	127.9698	208.0019	194.3944
gene-AT3G	34.21545	37.21427	30.34284	16.88271	22.69832	17.33266	9.786469	11.80004
gene-AT3G	20.35149	20.28865	18.78491	31.12717	28.95281	27.68957	15.90297	16.93904
gene-AT1G	2.498081	2.180462	2.189393	0.677577	0.290382	0.851126	2.249225	2.719604
gene-AT1G	1.928455	2.864185	2.553166	5.26391	4.605326	5.954379	1.987195	2.721215

gene-AT3G	3.649182	3.348654	3.342525	3.916443	3.657743	3.078248	0.64507	0.611759
gene-AT2G	4.089846	5.028845	3.807205	9.571067	11.57579	8.041861	6.146898	5.774523
gene-AT4G	46.65781	45.13899	49.11979	32.75289	32.93572	33.91128	16.11455	15.20453
gene-AT4G	4.89408	4.001565	4.210514	5.25122	5.936046	5.844452	4.94489	5.975199
gene-AT5G	3.861368	4.414196	3.598775	5.250677	6.556399	6.094926	1.127239	1.509248
gene-AT1G	0.621703	0.69606	0.63239	0.703775	0.553758	1.089562	2.504808	1.677688
gene-AT5G	60.9847	57.43341	60.7899	41.24209	40.25198	40.71568	43.74252	44.1395
gene-AT4G	22.28758	21.02334	21.88934	8.13296	9.451638	9.456813	6.892808	7.249307
gene-AT3G	8.814539	9.51883	8.33136	18.64365	19.11474	19.5502	18.37589	18.81335
gene-AT5G	0.65775	1.263935	1.548947	0.799028	0.904923	1.176325	0.930755	1.615317
gene-AT2G	1741.031	1796.746	1735.514	1543.882	1552.341	1592.086	1746.024	1717.192
gene-AT3G	92.05427	91.24666	94.37505	79.51708	80.77403	76.49854	132.6256	138.17
gene-AT1G	14.35905	15.33577	14.60839	24.67889	25.08232	23.66139	45.36236	44.24977
gene-AT1G	15.24516	14.97453	13.38451	8.233206	10.31861	8.581154	3.740746	2.245258
gene-AT3G	22.54331	19.91634	21.18958	11.86801	12.37291	11.97894	2.487776	4.179832
gene-AT1G	10.49833	9.594095	11.52464	18.76767	19.32639	18.92594	4.386862	4.385719
gene-AT5G	35.48057	34.04009	38.57901	28.03842	28.1785	27.13434	47.77531	43.74342
gene-AT4G	28.99287	28.43476	29.71072	33.77167	32.71348	34.37574	38.79654	41.16423
gene-AT2G	0.886837	1.146955	1.163622	2.307081	2.134072	2.080808	2.60088	4.405701
gene-AT1G	30.5752	29.36276	30.31884	17.02301	17.58893	16.93056	17.11283	18.74924
gene-AT1G	1708.375	1655.44	1790.959	2355.161	2249.961	2344.035	522.2671	480.2573
gene-AT3G	72.55261	77.13661	68.56088	49.04953	55.96697	49.80664	142.8441	149.1759
gene-AT5G	3.096078	2.673353	2.379101	1.646503	1.214839	1.503905	3.078706	5.391664
gene-AT2G	6.378591	5.511118	4.594407	3.61965	3.767102	3.087821	6.478689	8.96139
gene-AT4G	2.372578	2.340459	2.194099	4.035202	4.047372	3.273524	3.12546	3.24123
gene-AT4G	9.013687	6.502201	7.320722	9.466257	7.499127	8.509099	15.48451	17.38621
gene-AT5G	3.56996	3.008397	3.397278	7.608951	6.495772	6.318844	5.263871	4.221781
gene-AT3G	3.689445	3.962191	4.4084	5.109695	7.720435	5.799913	3.039963	1.753917
gene-AT3G	0.9852	0.853181	0.846219	0.961978	1.224413	1.1015	0.681765	0.863231
gene-AT3G	1.795056	1.413746	0.936266	1.062427	1.386207	1.030677	0.745369	0.519411
gene-AT2G	13.98667	14.15475	13.90511	9.820485	10.70583	10.40016	19.2203	17.91665
gene-AT3G	17.68953	17.8896	16.83371	23.1896	21.85098	20.8041	12.7172	15.10357
gene-AT5G	8.264928	7.684693	9.216098	9.921406	9.487988	9.469333	16.30737	17.72456
gene-AT2G	23.675	21.86321	21.44396	19.93759	20.78706	22.91297	9.725981	11.82539
gene-AT5G	112.3391	107.1336	115.6786	88.68465	83.57822	76.7733	197.4976	198.2001
gene-AT4G	2.874291	2.889634	2.723912	0.850228	1.20379	1.088289	2.403234	2.083329
gene-AT1G	8.587084	8.313268	7.492877	4.028694	3.606972	3.583715	13.23838	11.83658
gene-AT4G	0.691307	0.675869	0.664887	0.306961	0.493495	0.656803	0.067046	0.058606
gene-AT1G	0.351481	0.266508	0.162438	1.175498	1.053031	1.017723	0.339078	0.199877
gene-AT5G	283.8364	271.6129	289.6059	216.6493	212.4137	214.2353	263.3639	277.0673
gene-AT1G	35.46906	34.83202	35.69149	63.42662	64.3041	62.476	16.14629	16.85479
gene-AT5G	3.727717	3.988154	3.125889	3.437063	2.771865	4.849306	8.961987	7.621915
gene-AT3G	1.610973	1.869933	1.76021	8.48892	7.030068	8.237112	0.703443	0.758198
gene-AT2G	1.626149	2.708024	1.552208	4.497018	4.350089	3.967494	1.933057	1.877773
gene-AT1G	2.914891	3.07789	3.31394	1.80104	1.728139	2.084551	3.141219	2.917693
gene-AT4G	0.583852	1.37085	0.80315	0.755716	0.620797	1.245946	1.312067	1.771692
gene-AT1G	2.157492	1.709128	1.798931	3.38618	3.962874	3.501994	1.697078	1.467033
gene-AT3G	25.45279	25.08251	24.40722	16.11947	15.40331	15.73562	32.2273	33.68823
gene-AT2G	268.2066	266.145	263.684	195.1216	191.5056	190.8275	438.7049	442.5765
gene-AT1G	1.819621	2.222148	2.457126	6.025114	6.06117	6.088255	2.294275	1.547761
gene-AT2G	3.569342	4.154599	3.676522	4.963309	5.036194	4.822345	7.714413	7.743948

gene-AT2G	0.54509	0.453747	0.644168	1.367977	1.056125	0.830824	1.55084	2.134975
gene-AT2G	0.911954	1.670229	1.018672	1.85916	1.811507	1.925213	0.136677	0
gene-AT4G	13.97781	13.10245	12.08009	36.14842	38.45967	41.65687	14.03225	16.7962
gene-AT4G	119.4046	118.1538	119.4019	272.5267	271.3534	271.6855	92.78348	94.49541
gene-AT5G	4.210082	4.154506	4.242968	7.638313	7.000106	7.241684	3.539982	3.415562
gene-AT3G	29.03271	28.06245	27.86396	8.842176	9.028242	9.624906	94.18308	99.12151
gene-AT4G	30.92615	36.53993	32.47519	11.41853	11.99925	13.92491	3.710928	3.23839
gene-AT4G	2.650553	3.265106	3.534184	2.663963	3.665701	2.671453	1.410643	1.58732
gene-AT3G	22.21579	23.20416	25.46732	50.29205	48.65198	49.31979	18.22464	18.68553
gene-AT4G	193.6175	186.3951	201.9	18.6655	19.0109	18.89674	195.6312	207.2435
gene-AT5G	7.157834	8.012653	7.991581	5.191501	5.419963	5.089493	2.442763	1.979676
gene-AT3G	0.678421	0.569005	0.781861	1.585047	1.361426	1.805012	0.696879	0.692252
gene-AT3G	637.3097	627.9159	656.2803	598.493	594.9288	582.3721	250.2828	265.2458
gene-AT2G	9.787267	9.400795	10.23294	2.633323	2.118471	2.373851	7.771665	7.195758
gene-AT4G	20.09082	20.88412	23.24155	10.406	9.586701	9.341741	11.40154	12.08768
gene-AT1G	6.515284	5.63714	5.134904	8.61105	9.171643	6.587646	5.870623	5.470866
gene-AT5G	0.640476	0.479049	0.580732	1.821735	1.963356	1.644472	0.270747	0.407045
gene-AT1G	6.114353	6.676519	6.50622	3.96086	4.093614	3.969949	11.4947	11.8799
gene-AT1G	7.884886	6.794307	8.904135	10.62682	12.29664	12.55032	3.810763	4.785414
gene-AT1G	0.334421	0.438061	0.528425	0.109885	0.164013	0.126266	0.687256	0.827766
gene-AT1G	0.650511	0.671019	0.771119	1.524627	1.892935	1.494429	0.632666	0.655854
gene-AT5G	10.04711	9.759333	9.461645	6.513842	6.174986	6.515477	5.977742	5.847777
gene-AT1G	4.593847	3.783793	4.074689	3.612274	3.247559	3.839408	0.862875	1.131543
gene-AT3G	3.199516	2.247939	2.568675	1.938331	2.079102	1.993812	4.188002	5.213491
gene-AT3G	19.42501	17.70563	18.45645	13.58587	13.30386	13.99408	35.45931	34.44395
gene-AT4G	45.31795	47.40079	44.52862	22.09441	25.86262	20.48458	19.77104	18.86249
gene-AT3G	10.37373	9.238313	9.415321	4.494848	5.918605	4.791267	3.574218	3.919325
gene-AT1G	1.317007	2.033238	1.678816	2.107111	2.277781	1.413389	2.321926	1.730464
gene-AT5G	34.00441	32.31175	34.44088	28.31793	28.73325	27.29377	46.66901	49.95984
gene-AT3G	3.763928	3.00764	3.320958	3.211258	3.133775	3.929125	5.101926	3.890702
gene-AT5G	24.84233	23.27691	25.16038	23.73017	21.63043	21.73614	32.13144	35.13202
gene-AT5G	3.376474	3.269589	2.514378	4.799905	5.916443	3.741822	2.18659	1.583361
gene-AT5G	4.617119	3.866936	4.704453	6.946596	8.400062	7.955146	3.182784	2.428298
gene-AT1G	5.806185	6.629871	7.33426	6.736361	7.409685	7.369203	7.164382	8.003242
gene-AT2G	2.478451	3.524376	3.606319	1.600284	1.966575	2.147736	2.953648	3.388746
gene-AT4G	3.759294	3.933153	4.55502	4.941621	4.996478	5.498816	2.405811	2.249692
gene-AT1G	98.82684	101.5835	99.96313	122.6988	118.6759	118.071	33.92987	30.29972
gene-AT1G	39.54057	40.58477	37.98969	32.07856	35.80438	32.88466	13.66016	15.96977
gene-AT1G	13.7045	11.51373	12.70534	2.425919	2.416152	2.300276	31.79607	31.73654
gene-AT1G	23.90352	26.57629	21.39598	17.65468	17.32463	15.50927	36.54987	39.26064
gene-AT5G	23.13068	21.87591	22.74678	18.10597	16.08336	17.19528	36.72909	37.42539
gene-AT1G	15.30606	15.95768	16.30457	11.80619	13.49376	11.34081	23.4037	25.10581
gene-AT1G	2.347412	2.509834	2.93019	1.076343	0.913103	0.805351	0.320428	0.338241
gene-AT1G	13.40665	13.29937	13.9398	12.34078	10.42078	10.72081	5.149363	4.686966
gene-AT5G	11.63431	10.80229	10.03021	15.04409	15.13772	15.17973	7.031756	6.154869
gene-AT1G	89.47784	92.99606	90.51981	56.20054	51.26219	52.86053	163.7877	165.8892
gene-AT3G	0.6849	0.729672	0.35181	0.788073	1.15218	0.757193	0.138662	0
gene-AT2G	0.781956	0.884815	0.975925	2.206762	1.359205	1.916506	0.652435	0.349453
gene-AT3G	0.869222	0.688455	0.761275	0.386164	0.341768	0.264656	1.214881	0.930816
gene-AT5G	2.165814	1.323913	1.576628	2.393583	2.560191	3.411346	1.082512	1.026722
gene-AT1G	0.74809	0.839308	1.052343	2.046391	2.093479	1.715435	1.224055	1.765335

gene-AT5G	79.93438	82.21027	82.53578	69.09438	75.71825	66.50558	29.59507	28.47724
gene-AT2G	22.85569	25.19213	24.4846	36.75166	37.60081	35.81408	18.03259	19.29669
gene-AT5G	0.200231	0.174038	0.176352	0.297481	0.24646	0.210024	0.25775	0.408843
gene-AT1G	45.52158	46.46908	40.6795	20.10629	19.10537	19.14261	36.02018	40.70607
gene-AT4G	0.898582	0.361621	1.341133	1.522663	1.0232	1.991347	0.489631	0.762191
gene-AT2G	4.473716	4.954163	4.778809	4.55626	5.546885	4.559198	1.965993	2.665791
gene-AT4G	8.001978	9.127451	8.815021	54.4853	50.24108	50.53116	8.723145	7.793527
gene-AT3G	4.065135	4.381385	5.091048	0.285595	0.276668	0.874083	0.368562	0.395298
gene-AT2G	5.350131	6.047873	5.498106	4.628581	4.848467	5.910627	1.004584	1.222828
gene-AT1G	358.3568	393.625	357.6869	540.2958	556.443	572.5262	299.7225	240.9793
gene-AT2G	10.12605	9.818976	11.02255	4.318964	5.199381	4.454817	11.4191	13.93733
gene-AT2G	35.43996	37.4252	36.86793	44.383	44.88526	42.83851	46.09662	44.17869
gene-AT1G	12.67989	11.39087	13.25922	15.96974	15.97894	14.84744	29.5088	29.97622
gene-AT1G	5.643484	5.690813	5.88047	6.447768	6.589481	9.337725	13.31269	10.66365
gene-AT5G	2.001337	2.30083	2.445322	1.549743	1.665825	1.373506	2.764971	3.393355
gene-AT5G	15.31388	15.09924	13.96594	23.04691	23.81711	21.51065	15.50478	18.31037
gene-AT2G	1.045272	1.486062	1.239062	2.304502	2.892967	2.319486	2.576301	3.125543
gene-AT1G	1.43888	1.026089	1.250365	2.375507	1.604601	2.888041	0.623238	0.757596
gene-AT2G	15.32283	15.59763	16.32885	12.94678	12.52839	10.82287	11.94841	14.19278
gene-AT5G	7.903063	6.923857	6.929942	4.377137	4.677403	5.162036	9.90172	12.66838
gene-AT5G	42.88155	47.65804	45.53902	34.00669	36.11101	34.95968	19.42988	20.57886
gene-AT1G	31.1517	29.32497	34.87523	29.63276	31.36124	29.11337	34.69093	34.67368
gene-AT4G	5.88882	5.946236	5.648625	3.130442	2.772291	3.380738	11.48868	12.1008
gene-AT1G	15.92297	13.69858	15.51718	13.99956	12.99071	13.54924	24.751	26.46116
gene-AT2G	5.973049	5.715745	5.048841	7.97973	7.347934	7.138821	3.897765	4.310173
gene-AT2G	1.455665	1.148099	1.302925	1.38426	0.985653	0.496289	0.700114	0.655641
Arabidopsi	1.124017	1.194123	1.230899	1.777291	1.604918	1.455693	0.064784	0
gene-AT2G	0.470701	0.314117	0.353777	3.749483	3.315152	3.576946	1.005994	1.460446
gene-AT2G	0.097498	0.218691	0.211292	0.474736	0.280594	0.487343	0.020856	0.055184
gene-AT2G	72.3613	69.7381	67.59205	39.08568	37.32062	41.53932	61.66758	64.96773
gene-AT3G	0.051403	0.06954	0	0	0.103042	0.09519	0.042731	0
gene-AT4G	6.86641	6.481107	6.528075	6.815926	6.768204	7.143856	7.29612	8.924787
gene-AT2G	1.471407	0.873779	1.031253	1.447795	1.714741	1.380118	0.916695	1.341593
gene-AT1G	12.4069	8.537546	9.203225	5.181814	5.836407	6.38173	10.75622	12.91623
gene-AT3G	31.77732	31.24996	32.91935	102.251	102.1806	104.2088	18.90556	19.58462
gene-AT5G	1.580999	0.95237	1.646422	2.518579	2.757373	2.232296	0.995643	1.534687
gene-AT3G	29.10607	29.11406	31.40337	36.91542	36.09074	35.23373	12.9151	17.77748
gene-AT2G	11.72319	10.10072	11.13087	4.514462	5.564192	5.088493	3.335153	2.577309
gene-AT4G	0.65422	0.536804	0.556625	0.523534	0.476778	0.663208	0.623603	0.882917
gene-AT1G	5.301738	6.987804	6.266261	7.792047	7.733212	6.993854	7.190238	8.174794
gene-AT2G	222.6386	221.6628	231.0597	101.3909	99.70078	99.83223	201.0777	203.1216
gene-AT4G	404.55	392.4699	401.3465	298.2866	297.6946	290.1714	571.6555	595.8674
gene-AT1G	6.259086	6.379461	6.179473	5.444473	5.561082	6.049303	9.462082	10.79224
gene-AT4G	18.20007	18.25619	19.78566	18.26974	21.15867	20.92214	30.83387	32.37716
gene-AT2G	33.95747	33.90485	32.64805	37.10693	37.26697	36.59562	13.28286	11.70982
gene-AT3G	47.05849	48.34228	47.48591	35.21124	36.05451	33.57137	52.73182	57.64545
gene-AT1G	46.12256	45.9443	46.18972	32.76297	32.84935	28.66254	75.44201	78.01987
gene-AT3G	0.86247	0.814129	0.628197	0.490079	1.448199	0.841326	0.183549	0.465986
gene-AT5G	1.289389	0.800672	0.783946	1.506689	2.353246	2.246964	1.839774	1.614028
gene-AT3G	6.489695	6.919473	8.143437	6.140798	5.963202	5.954718	1.546609	1.133162
gene-AT2G	19.99862	18.98091	17.64765	16.03541	16.89421	17.01494	22.47284	24.76511

gene-AT1G	0.426041	1.270745	0.622629	2.250838	1.694522	3.001847	0.631176	0.474719
gene-AT3G	201.9651	195.6313	201.2819	344.8495	347.8834	335.8287	123.1213	126.8932
gene-AT3G	0.854141	0.830446	0.995773	0.742254	0.969019	1.10734	3.051908	2.921661
gene-AT5G	1.582432	1.772549	1.863327	0.313595	0.277095	0.510515	1.452257	1.180713
gene-AT2G	2.061787	1.766535	2.021657	0.539795	0.69991	1.26462	0.17051	0.392043
gene-AT1G	5.27165	5.830579	6.014286	10.24386	8.464307	9.27562	5.230844	4.357168
gene-AT5G	13.86429	13.11654	13.7017	14.12284	14.75093	13.30392	18.7818	19.36794
gene-AT3G	4.287414	4.332629	4.135466	3.118459	3.067975	2.922755	4.404561	3.957878
gene-AT3G	1.325586	1.491921	1.814775	1.28712	1.941869	1.599897	1.813796	1.801524
gene-AT3G	3.679853	3.819153	3.550554	2.412091	2.296478	2.781919	2.310002	2.02984
gene-AT5G	1.254776	1.201037	0.99246	0.912708	1.000861	0.740977	1.727863	1.331286
gene-AT3G	46.32717	46.70897	46.28024	72.00295	68.36603	65.79466	8.478877	9.024091
gene-AT5G	7.631907	6.865178	8.522324	7.940087	7.040585	7.273963	3.016764	2.391573
gene-AT5G	2.400302	2.8455	2.640706	1.990314	1.410658	1.863833	3.325979	3.588456
gene-AT5G	234.4505	239.3262	237.2217	109.1286	110.927	114.9158	726.2631	764.1639
gene-AT3G	27.92114	27.00107	26.04657	29.98824	31.48519	31.96534	51.44808	52.59854
gene-AT1G	62.62757	59.606	61.31781	64.31784	62.35981	67.1773	104.6149	104.1313
gene-AT5G	3.221359	3.714839	3.808484	5.211878	4.382087	3.956239	6.428168	6.81403
gene-AT5G	0.600025	0.210883	0.172389	2.243826	2.399824	2.164521	0.652788	0.774279
gene-AT3G	9.258503	9.131083	9.908436	23.83489	26.85391	24.46496	3.002926	2.389232
gene-AT1G	1.695179	2.233809	2.608629	1.0437	0.622792	1.223499	1.315623	0.614363
gene-AT3G	3.627628	3.124822	3.957974	3.615231	4.101062	3.835334	2.026004	1.711519
gene-AT1G	0.870508	0.538769	0.487146	0.880223	1.440136	1.208072	0.417421	1.082543
gene-AT1G	5.588198	6.85815	5.732237	6.416937	7.376655	7.311456	3.716506	3.845801
gene-AT3G	0.09021	0.378397	0.472051	1.182063	1.445457	1.326043	0.397954	0.073896
gene-AT4G	0.493984	0.37214	0.534908	0.607329	0.693371	0.931438	0.238644	0.085291
gene-AT3G	4.317702	4.192747	5.97134	5.712918	7.517611	5.380073	1.365904	1.368543
gene-AT1G	43.05984	40.53726	39.42009	36.29372	33.91545	34.78299	14.93639	18.08884
gene-AT4G	1.553604	1.572459	1.162308	2.419349	2.771709	2.166091	0.599625	0.522394
gene-AT4G	0.158733	0.143601	0.272924	1.198617	0.908913	1.13318	0	0
gene-AT1G	2.320565	3.14065	2.904574	2.761219	2.33698	1.857193	1.154082	0.979721
gene-AT1G	28.23513	30.20307	26.96918	14.76242	13.63579	13.80177	19.34456	19.80716
gene-AT5G	10.55168	9.751666	9.722032	10.58929	9.992885	9.796217	8.803802	9.089597
gene-AT5G	1.336874	1.911237	2.157183	2.822476	1.847304	2.371328	0.922983	0.857368
gene-AT2G	1.484355	0.788247	1.617266	0.217048	0.255556	0.363846	2.169082	2.157118
gene-AT5G	2.289767	2.222684	1.503351	3.238518	2.858121	2.538211	8.55895	10.87333
gene-AT2G	68.27828	66.9272	61.10878	94.22798	98.14699	94.64295	35.06257	37.66857
gene-AT1G	7.107034	5.846701	6.858602	20.13873	18.75681	21.43789	6.469567	5.899537
gene-AT2G	2.463029	2.039929	1.942987	2.261301	1.274192	2.022261	1.341237	1.189286
gene-AT5G	4.445245	4.289564	4.25417	8.779241	9.667919	9.663893	9.402726	8.616967
gene-AT5G	132.2791	124.9728	130.8419	169.2772	173.1539	173.558	48.3734	51.28244
gene-AT5G	8.624762	6.372782	6.515352	3.151253	2.478562	2.283002	1.258106	1.287522
gene-AT2G	40.62466	37.67168	38.412	14.30123	15.37541	14.4118	20.44955	21.50138
gene-AT4G	4.509716	5.133186	4.603925	1.84653	1.603219	1.508962	6.920926	7.602797
gene-AT2G	0.719839	0.925924	0.823959	0.801575	0.789936	1.161519	1.264402	1.050214
gene-AT5G	0.135021	0.274287	0.180626	0.528235	0.504831	0.693011	0.027915	0
gene-AT5G	0.448453	0.470313	0.789572	0.722213	0.567698	0.716217	1.071439	1.035569
gene-AT5G	11.30667	9.973621	11.46556	10.37546	10.93449	9.984862	6.2323	5.817809
gene-AT5G	3.830587	3.956772	4.108245	1.666948	1.497057	1.02074	5.683948	7.200684
gene-AT5G	0.227981	0.166168	0.156597	0.603063	0.740901	0.715285	0.284544	0.421734
gene-AT2G	5.807129	6.377737	6.534336	5.907073	4.5315	6.476176	15.70052	15.34137

gene-AT5G	3.507347	2.977941	2.910856	5.676075	5.162776	6.778988	3.601216	2.127451
gene-AT1G	26.81657	25.16427	26.81714	63.20156	59.17347	62.21187	23.97167	27.0107
gene-AT3G	0.942679	1.419639	1.006215	1.912996	1.190534	1.844976	0.402201	0.329348
gene-AT4G	11.0196	11.40311	12.34126	7.088415	8.170978	7.04844	10.27679	12.09549
gene-AT4G	8.803465	8.170245	7.081177	6.646562	7.607826	6.708683	3.752255	3.788685
gene-AT3G	0.906664	1.215348	1.187831	0	0	0.048911	7.903612	8.667854
gene-AT5G	25.11678	26.94433	24.95747	15.28859	14.97063	13.65897	34.8302	34.70265
gene-AT5G	11.71538	12.28604	12.48768	11.03788	10.05963	10.81882	23.69893	24.17681
gene-AT4G	14.9687	13.81595	14.98314	26.9274	28.665	27.77749	10.50351	11.24102
gene-AT1G	26.99084	26.51756	26.97532	30.89706	30.51643	30.059	18.28328	18.8165
gene-AT1G	2.375384	1.61555	2.54079	0.826258	0.820512	1.476948	0.42749	0.703317
gene-AT4G	11.82172	12.6221	12.28939	7.157144	7.547859	8.243401	19.96162	22.40785
gene-AT3G	667.6144	648.6557	669.5172	274.408	280.4353	271.2797	714.7221	707.4754
gene-AT2G	87.196	105.9142	88.6949	92.17208	96.89494	95.98518	49.05967	41.66852
gene-AT4G	1.417907	1.525928	2.152729	2.486014	1.714944	2.505139	1.576774	1.046494
gene-AT2G	8.25043	7.83077	7.986623	7.670558	7.265377	5.064899	14.13754	14.6354
gene-AT4G	38.55715	37.30263	38.89075	33.51881	31.86142	31.72418	55.12276	53.19532
gene-AT1G	2.300479	1.684652	1.367574	2.319765	2.300278	2.666466	0.81705	0.597818
gene-AT1G	2.255656	1.815636	1.818243	0.841555	1.251393	1.00483	0.311101	0.278105
gene-AT1G	9.532071	12.18384	10.06118	7.648759	5.733465	5.960455	15.70131	17.34105
gene-AT5G	0.688194	0.370476	0.593298	0.596266	0.855734	0.366577	0.230753	0.183532
gene-AT5G	0.934632	1.042467	0.887905	1.517444	1.987975	2.133772	0.488883	0.283209
gene-AT5G	203.6749	197.947	208.0278	106.8233	110.448	102.5757	141.4677	143.7124
gene-AT1G	21.61422	22.22232	22.69809	34.55689	32.58539	32.00524	8.39352	7.38578
gene-AT1G	4.368687	3.729261	4.551953	10.81189	9.951633	11.16154	2.655665	2.280647
gene-AT5G	1.479449	1.41542	1.615932	1.399754	1.636384	1.5159	2.134007	1.787679
gene-AT5G	4.094422	3.687213	3.684291	7.36639	7.693239	8.201406	2.567314	3.017395
gene-AT1G	2.191603	2.383955	0.926064	3.87012	4.231483	4.049829	1.580557	0.734242
gene-AT3G	8.521039	8.579429	7.595389	0.502046	0.363249	0.624731	14.00329	15.05758
gene-AT5G	0.739974	1.063206	0.618231	1.642214	1.846475	1.676946	0.271774	0.277469
gene-AT4G	3.158181	3.732766	3.405237	8.68099	8.249566	9.41369	1.764668	2.030291
gene-AT2G	9.237926	9.063621	11.23965	12.21703	11.60626	13.1993	4.056947	3.880131
gene-AT5G	5.006506	3.322541	3.682322	3.245556	3.483686	4.012105	2.084648	2.870065
gene-AT1G	2.103576	2.868163	2.507576	4.916951	2.986812	4.484005	6.234538	6.870727
gene-AT1G	2.810494	2.75217	2.312975	1.880761	2.355602	2.307717	5.94107	6.170197
gene-AT3G	0.574561	0.70183	0.652327	0.540506	0.371673	0.538858	0.02021	0.024419
gene-AT3G	3.742846	3.348953	4.11746	3.988274	4.284987	3.790668	4.257076	5.285288
gene-AT4G	31.73961	30.62847	29.99998	28.5372	27.88749	26.33824	13.47263	11.46071
gene-AT1G	89.35497	85.83954	85.16424	79.96592	78.37377	77.27839	54.93329	60.09681
gene-AT1G	197.8405	197.9084	204.9868	130.4198	129.945	134.2378	357.8385	367.7871
gene-AT1G	12.08847	11.23415	9.824581	5.08147	5.14565	6.846237	2.947427	5.153702
gene-AT5G	1.038805	0.842232	1.173714	3.677204	4.170582	3.936139	2.010604	1.002168
gene-AT4G	7.522727	7.192959	6.32632	2.189775	1.884781	2.843991	10.90635	15.94752
gene-AT5G	5.18267	5.153769	5.392628	3.002234	3.257563	2.545056	5.914288	5.075873
gene-AT5G	8.244013	9.339776	8.861425	7.837987	7.853069	8.78054	2.895037	2.985583
gene-AT1G	1.09317	0.849627	1.327656	1.518781	1.346588	1.743212	1.033282	1.038325
gene-AT2G	15.10503	15.55872	17.78689	12.79943	12.65688	12.85346	1.283389	1.591625
gene-AT5G	1.024486	0.88163	1.147978	3.190016	3.925797	2.833621	1.450189	2.030961
gene-AT5G	1.815625	1.869088	2.002417	7.448969	7.038636	8.536148	2.590712	3.228855
gene-AT4G	6.303944	7.061337	5.917376	7.157837	5.4205	6.091153	2.601558	2.482839
gene-AT1G	12.88792	12.89662	12.41235	9.658308	10.34502	8.045682	21.85254	25.91085

gene-AT4G	1.027723	1.105177	1.27883	1.132994	1.117006	1.233313	3.652568	3.73761
Arabidopsi	0.214223	0.351981	0.641568	1.984907	1.798426	1.062245	0.452226	0.11333
gene-AT4G	4.11264	4.852933	3.093706	4.081336	4.233576	2.956215	2.783923	3.513102
gene-AT1G	39.2084	43.34197	41.96008	37.2363	40.02182	38.1887	67.94115	67.34038
gene-AT2G	50.85654	50.97516	50.16773	37.49506	37.77039	39.7685	82.78653	85.48973
gene-AT4G	10.91601	11.94445	11.95386	8.353739	7.166702	6.905767	20.45687	19.27758
gene-AT3G	27.04683	25.83291	30.95347	36.34932	36.10183	36.29736	18.07121	18.70761
gene-AT3G	1.569322	1.409667	1.410849	3.473728	3.496471	3.03845	0.883384	1.007289
gene-AT1G	3.554487	2.696741	3.793137	6.598201	8.442278	10.01934	5.053579	3.148426
gene-AT1G	551.2853	533.0566	554.5506	421.2132	413.1115	416.8024	346.132	349.5502
gene-AT2G	1.444203	1.375417	1.834818	1.888656	2.463394	2.633183	1.310528	1.570424
gene-AT1G	30.78392	30.89296	29.43646	24.90785	21.23708	24.14723	35.4106	37.55214
gene-AT1G	12.65629	9.654722	11.85923	9.176894	9.070683	8.491185	9.659956	9.555192
gene-AT1G	3.047587	4.348609	4.698251	0.696593	0.60316	1.003461	5.358408	5.101121
gene-AT1G	30.36777	31.41539	32.52037	42.31532	42.66254	41.30013	17.35725	18.5045
gene-AT5G	4.174587	4.539742	4.329988	1.294222	1.298575	1.466299	1.519933	2.167114
gene-AT3G	3.452858	2.825216	2.363451	1.989148	2.161687	2.833468	1.129885	0.910742
gene-AT3G	63.37644	65.38014	63.23305	25.23095	27.83179	25.75623	106.1223	110.5947
gene-AT3G	3.872095	4.380612	3.241069	3.506967	3.823009	3.168941	4.017323	3.065696
gene-AT5G	16.74284	15.75912	16.52511	1.626554	1.690169	1.826715	37.18466	38.27913
gene-AT2G	145.1421	151.6624	142.1576	80.25543	88.76328	88.43021	367.8567	341.5859
gene-AT4G	5.592193	4.965809	4.36325	7.414539	7.128505	7.038863	0.291937	0.26946
gene-AT4G	4.808292	4.869007	4.522014	3.812222	3.96318	3.94049	6.962562	4.471797
gene-AT4G	1.932496	2.002149	1.341969	1.956822	2.156637	2.086456	0.924096	0.6863
gene-AT1G	1.442158	1.105842	0.812232	0.941363	1.033919	1.387179	2.538161	1.722663
gene-AT2G	11.52587	11.60176	12.15656	13.13737	11.81049	13.20996	10.8906	10.30617
gene-AT3G	6.262242	6.365073	7.30835	9.103258	7.849539	8.011718	10.42594	11.50263
gene-AT4G	16.11622	14.60155	16.79899	12.28572	13.1165	13.50155	22.70191	24.43419
gene-AT5G	70.00301	77.77023	75.98217	33.275	31.44643	31.69932	38.40633	39.1
gene-AT1G	0.654081	0.529271	0.355092	1.813506	1.859761	1.126374	0.726315	0.719552
gene-AT4G	11.78288	13.24451	13.99278	23.36749	24.17045	20.78267	9.400161	7.304352
gene-AT5G	10.44068	8.785198	8.3673	6.947934	7.339036	6.296454	9.535782	12.18499
gene-AT1G	2.06159	2.076035	1.614265	3.16834	4.001266	4.302439	1.090823	0.760082
gene-AT1G	2.215046	3.720983	2.425848	0.643599	1.484067	0.932478	0.471801	0.95771
gene-AT5G	4.399681	4.952795	5.298071	28.3852	31.08076	30.09719	8.346936	7.395011
gene-AT2G	1.751154	1.338962	1.266399	1.115797	1.211496	1.213012	1.040146	0.93884
gene-AT3G	14.04453	12.96764	14.28092	13.32364	14.03682	10.84802	18.90694	21.65744
gene-AT5G	7.24804	5.608005	5.488191	3.614465	2.125874	3.147532	7.196797	7.738216
gene-AT1G	5.947828	6.434305	6.431987	5.574612	5.119973	4.808073	8.741716	8.640074
gene-AT1G	92.20845	100.8577	101.8718	74.46585	75.77261	82.84208	90.83627	94.27502
gene-AT1G	195.643	194.5306	197.4959	105.6985	106.2802	110.162	172.351	170.8494
gene-AT3G	10.57154	9.39076	10.85982	5.517198	4.773947	5.381925	14.23061	13.87258
gene-AT5G	0.263587	0.29144	0.505684	0.325856	0.511907	0.10891	1.333508	1.716875
gene-AT2G	86.18575	92.42513	84.14397	130.3254	131.8102	137.3826	119.2225	117.0038
gene-AT5G	3.23935	3.389392	2.408256	1.384156	0.925634	0.72613	3.762494	3.065452
gene-AT5G	6.643105	6.675843	8.084755	13.07493	10.37774	16.4644	2.589822	3.031691
gene-AT1G	0.36904	0.106538	0.287343	0	0	0	2.178039	2.435431
gene-AT1G	2.193104	1.988735	2.235567	0.738616	1.427663	1.350188	0.731611	0.560191
gene-AT3G	24.17422	23.70991	27.20318	31.68391	30.24186	31.70456	15.26537	16.40713
gene-AT2G	62.91137	62.67944	60.54465	15.29392	13.59817	13.99472	202.3004	216.3544
gene-AT5G	9.166102	11.0588	7.907429	4.306262	5.441252	5.129257	13.90535	14.18421

gene-AT4G	1.064682	1.290236	1.34586	1.467305	1.81398	1.618361	0.411402	0.595407
gene-AT1G	1.8502	2.144197	1.54068	0.883158	0.67986	1.17155	2.809719	1.874121
gene-AT1G	403.3897	406.2131	401.7038	247.0755	252.6879	266.7402	643.4849	659.1227
gene-AT5G	1.18497	1.267689	1.52332	1.548711	1.979064	1.402968	1.488253	1.718931
gene-AT1G	14.65669	13.4362	14.35501	19.28062	19.01579	18.60088	18.65564	18.90939
gene-AT4G	27.54598	27.55785	32.4868	37.12976	33.52729	36.51606	12.77351	14.1378
gene-AT1G	7.525806	5.996249	7.005256	5.563332	5.117898	6.040608	8.720898	11.87418
gene-AT5G	6.137914	5.324781	5.218699	10.3315	9.589892	9.187915	3.209425	2.157257
gene-AT3G	59.52692	56.8906	53.76238	44.46911	46.75777	44.25914	94.51602	93.04437
gene-AT5G	17.66406	17.74908	18.98653	5.431465	5.650819	5.966119	70.73364	73.12258
gene-AT4G	0.48892	0.415229	0.643961	0.701496	0.554394	0.453162	1.296938	0.950747
gene-AT5G	12.07661	11.53611	11.65994	10.20579	9.878927	9.888391	17.54805	18.13509
gene-AT5G	21.97263	20.93882	21.45369	14.99213	16.44578	16.50393	28.20917	27.09057
gene-AT2G	25.71003	23.42798	24.26455	24.0916	24.42988	24.27862	22.12585	24.13137
gene-AT1G	7.417278	8.746698	9.344999	4.929958	4.400925	4.059385	10.58806	9.690903
gene-AT4G	1.564157	2.403856	2.795936	0.647804	0.29492	0.606648	0.148121	0.463532
gene-AT2G	52.57177	53.96233	55.06798	41.74858	38.87401	39.78079	56.57955	58.76818
gene-AT1G	0.382525	0.354246	0.708831	16.85497	16.04624	16.02362	0.221738	0
gene-AT2G	1.643957	1.534809	1.783168	5.756842	4.402965	5.165937	1.772359	1.646647
gene-AT5G	7.033125	7.32897	5.558532	2.550757	2.676542	2.600535	1.861482	1.919445
gene-AT5G	0.373895	0.32288	0.447608	0.693592	0.819087	1.299787	1.072637	2.067087
gene-AT1G	5.515095	4.857348	4.797529	6.519906	5.911575	7.064032	6.217173	6.762175
gene-AT1G	37.91762	36.97571	33.79016	14.86659	15.21378	17.14607	53.07957	50.65495
gene-AT1G	15.0487	14.99873	11.67293	25.60025	22.45531	24.48109	4.339964	6.601353
gene-AT2G	6.83696	6.273324	6.756888	7.702168	7.184194	7.782592	7.33541	7.941062
gene-AT3G	14.61837	15.76216	14.52358	43.51403	46.40142	44.79496	10.53183	10.55582
gene-AT5G	23.79002	23.05968	23.82016	18.33936	16.1072	16.93088	35.60104	34.40298
gene-AT3G	0.464739	0.479973	0.523708	1.235476	1.416846	1.283963	0.687568	0.872892
gene-AT2G	236.29	224.703	231.4922	449.4297	448.3879	461.799	120.1443	122.2025
gene-AT5G	0.161901	0.600604	0.098614	1.172584	0.9674	0.711001	0.827208	0.966527
gene-AT4G	12.26524	10.99356	11.46893	14.82576	14.31092	16.59115	13.04321	11.30781
gene-AT2G	21.49569	22.07453	20.95945	12.13634	11.48867	11.69567	18.73792	20.43044
gene-AT2G	29.11404	28.49206	30.12193	15.60758	16.40505	14.97524	52.68579	53.30885
gene-AT1G	1113.735	1103.921	1121.044	541.1619	535.1069	533.6975	1713.265	1775.3
gene-AT3G	12.15282	13.39161	15.07052	10.69051	11.01507	10.18225	16.2952	16.5148
gene-AT4G	126.363	121.8325	120.1206	226.5188	233.1275	229.175	74.17796	72.77994
gene-AT3G	115.7201	115.3157	105.9161	101.7067	100.602	100.6108	49.53048	47.45075
gene-AT5G	1.375325	2.494238	1.992053	2.35396	2.891191	2.330671	0.978473	1.163987
gene-AT2G	0.222033	0.518874	0.816851	1.988173	2.034669	1.682901	0.311927	0.388537
gene-AT4G	1.662443	1.687416	1.523634	3.021253	2.687494	3.159385	0.691461	0.805934
gene-AT1G	8.426631	8.937656	7.92981	8.882201	8.504271	7.545871	5.775165	7.479156
gene-AT5G	5.056895	3.763064	6.356875	11.16648	10.51296	10.8372	7.543882	7.042029
gene-AT5G	3.342009	3.058407	3.240925	0.502013	0.248027	0.543894	0.021848	0.14387
gene-AT4G	21.24709	20.47733	20.00311	140.7026	129.3537	136.9898	18.20138	20.16132
gene-AT1G	6431.695	6262.263	6461.638	4534.314	4518.575	4464.882	8678.793	8845.758
gene-AT1G	12.37511	11.82638	12.43795	14.88166	13.92864	15.19077	3.102556	1.935862
gene-AT2G	12.74706	9.846916	12.26852	6.541147	6.578805	6.436511	2.253828	2.449879
gene-AT1G	1.489501	1.28756	0.984572	0.894424	0.875619	1.161478	1.963916	2.767637
gene-AT3G	9.887204	7.852511	8.557095	12.98899	11.30327	10.46975	4.426388	4.879006
gene-AT1G	3.672546	2.983227	3.595234	6.512659	7.418661	6.976266	2.214908	2.999943
gene-AT2G	7.045264	7.117465	5.785254	13.72747	11.8746	13.76098	5.866663	5.935055

gene-AT5G	3.294535	2.283804	3.328211	0.662006	1.07207	0.966425	0.343731	0.407397
gene-AT5G	5.548762	5.483528	8.226497	18.03449	17.27865	17.91951	9.895368	9.871889
gene-AT1G	65.86425	67.42442	69.31973	41.70893	46.065	44.5446	111.6919	110.7953
gene-AT1G	106.0679	104.0424	109.2351	87.71364	87.0615	85.87219	156.9586	163.9489
gene-AT1G	18.60965	16.64138	19.18401	14.63458	12.12992	12.71882	28.07638	27.41539
gene-AT5G	14.34151	16.64422	15.85879	11.553	10.91683	9.666983	26.28716	26.52855
gene-AT2G	12.0485	11.0071	11.05175	1.960969	2.557083	2.014168	0.496192	0.398245
gene-AT2G	4.226556	3.635386	4.563491	4.11562	4.937814	4.419471	5.319533	5.094414
gene-AT1G	13.18149	12.48151	13.69807	10.64086	11.38648	11.00391	4.728102	4.879177
gene-AT3G	0	0	0.046036	0.698256	0.147558	0.79188	0.021132	0
gene-AT1G	0.214734	0.368058	0.257432	0.164904	0.401946	0.374878	0.379788	0.35832
gene-AT5G	1.774782	2.421758	1.66764	1.675712	1.659624	2.222717	1.243209	1.381243
gene-AT1G	0.042009	0.15891	0.26221	0.374301	0.453027	0.784834	0.025362	0
gene-AT3G	19.11738	23.27252	19.96799	19.54757	20.86644	17.67343	10.31742	11.09362
gene-AT3G	23.69411	29.61647	32.0184	9.328033	8.931961	7.240404	43.80975	37.90288
gene-AT2G	33.05071	33.78912	35.24133	32.12132	36.70536	34.21668	2.93318	4.406528
gene-AT1G	5.381325	4.824691	5.068032	16.89539	14.97447	14.28487	0.514878	0.660766
gene-AT2G	0.306994	0.520511	0.337049	0.685546	0.654018	0.603727	1.948198	0.876468
gene-AT5G	12.21766	11.45482	11.47706	18.47063	16.93353	16.36633	2.858009	3.148917
gene-AT4G	9.424958	9.572518	11.0523	6.635842	8.412209	8.274994	3.817707	4.610248
gene-AT1G	30.93176	28.82556	30.46282	31.88511	33.02422	33.15812	43.16862	46.67126
gene-AT1G	0.662184	0.881202	0.961639	0.295564	0.241825	0.330967	1.454532	1.625027
gene-AT4G	26.00398	26.31028	28.09656	15.83575	17.50891	19.44255	43.26384	44.78116
gene-AT1G	0.224218	0.233548	0.403757	1.278794	0.775608	0.806977	0.503849	0.741244
gene-AT3G	0.832892	0.928423	0.497492	1.046226	1.558172	1.333796	0.183856	0.164026
gene-AT2G	0.91786	1.097541	1.452067	1.028384	1.032406	0.812501	0.135495	0
gene-AT4G	6.843453	6.993826	6.447054	9.212829	9.126888	9.485748	15.30845	15.62309
gene-AT2G	0.816329	0.64513	0.907995	1.12966	0.861616	0.765724	0.244815	0.407994
gene-AT5G	33.00628	37.1113	34.77082	30.14424	30.26658	32.11219	58.73209	57.78624
gene-AT3G	36.08185	32.93165	34.45101	29.97094	29.14332	31.92799	40.34138	45.78537
gene-AT2G	7.541144	9.13739	7.663832	23.39737	23.65637	25.19246	4.791353	5.202447
gene-AT5G	3.46031	4.026605	2.783751	7.058522	6.887155	7.175368	0.285254	0.211423
gene-AT2G	102.8043	109.9021	113.7296	84.52416	81.57195	85.71062	198.844	185.5506
gene-AT4G	38.4288	37.84214	37.3668	24.0472	23.07363	23.61266	29.97906	30.12215
gene-AT3G	0.90662	1.025551	1.055386	0.336676	0.199967	0.233333	1.573151	1.593299
gene-AT2G	1.101709	1.133219	1.425988	0.713924	0.91281	0.680275	1.255717	1.442414
gene-AT1G	12.05611	12.30312	13.37862	6.667487	6.070779	5.429005	17.14987	16.57831
gene-AT5G	3.292167	3.247513	3.416107	6.100148	6.008922	6.004856	1.977406	2.69218
gene-AT5G	1.2706	0.703603	1.317952	0.90155	1.434525	0.604254	2.606708	2.483148
gene-AT5G	12.27575	11.66251	13.46588	4.764368	4.791031	5.37701	5.263571	5.242393
gene-AT2G	14.23855	13.60073	14.99677	12.21798	12.67986	12.08548	4.655621	5.639945
gene-AT2G	2.930392	2.310573	2.994977	4.526748	4.476734	4.919134	2.280733	1.590653
gene-AT3G	277.3417	271.1907	273.8529	868.9837	869.6764	878.001	248.3508	252.4684
gene-AT1G	70.01396	68.06142	73.01469	72.00188	71.92261	71.55784	72.94729	72.99019
gene-AT2G	13.9253	12.74952	12.51696	9.057844	8.705939	10.12706	16.69969	18.19319
gene-AT1G	8.449444	8.899993	9.422598	12.23787	12.62213	13.34208	6.617082	7.141482
gene-AT5G	2.542369	4.063419	3.170449	1.567204	0.972854	1.241569	0.576345	0.4334
gene-AT4G	10.85608	9.984537	10.23053	16.36213	17.06555	16.13506	5.026546	5.700813
gene-AT3G	3.750568	2.460519	3.659395	0.828635	0.849164	0.772492	0.166166	0.077179
gene-AT2G	14.56025	13.78362	15.92601	11.13462	13.66331	12.0829	19.49857	19.4567
gene-AT2G	2.542974	2.706882	2.579036	3.703516	3.204503	3.897529	1.504873	1.971718

gene-AT4G	3.083427	2.065774	2.298802	2.490145	2.826804	2.471843	0.920893	1.081322
gene-AT5G	55.55455	52.22542	54.88989	135.8488	130.7605	132.5278	36.48119	37.62354
gene-AT5G	35.48849	37.02218	35.37479	18.39335	16.71763	16.21125	63.25314	63.82943
gene-AT1G	14.95263	13.97434	15.42407	29.15494	31.52165	31.70264	18.88268	20.16233
gene-AT5G	0.9736	0.762362	0.436951	2.454129	1.12437	2.575879	0.904412	0.866925
gene-AT1G	6.146244	5.38863	5.089062	3.700287	3.587917	3.288491	4.158715	5.523885
gene-AT2G	3.691906	2.847896	4.155576	2.979946	4.176292	2.433034	3.586184	4.573372
Arabidopsi	1.353564	1.58978	1.733114	0.88382	1.093934	1.16441	0.97675	1.588986
gene-AT5G	8.881326	6.989854	8.679149	6.448624	6.470154	6.489046	5.784387	6.277331
gene-AT5G	3.424329	3.444969	3.284123	3.202709	3.004813	2.694265	2.966981	2.195739
gene-AT1G	9.746736	10.09733	9.291472	2.534295	2.898615	2.377257	17.00721	16.71022
gene-AT5G	3.475353	5.074769	2.783858	7.672755	7.576417	6.323074	2.18026	2.43489
gene-AT4G	0.394056	0.401579	0.221907	0.984742	0.842736	0.693915	0.652671	0.841493
gene-AT1G	1.449995	0.90779	1.128255	1.247591	0.816751	1.019941	0.557584	0.61963
gene-AT1G	0.741074	0.451153	0.402522	1.820427	1.964054	2.036869	2.734149	2.2851
gene-AT1G	2.869025	2.270982	2.341506	5.85877	4.735034	5.361832	2.327008	2.537139
gene-AT5G	74.84885	74.89225	73.89721	57.10044	57.88215	57.15719	113.4693	114.7863
gene-AT2G	12.53366	12.41452	12.33978	12.92036	11.18007	10.2945	27.01046	25.01578
gene-AT1G	7.703795	9.503481	8.498665	5.732112	5.444608	5.208055	6.846849	6.225519
gene-AT4G	1.264856	2.006177	1.786927	0.448429	0.539626	0.356664	1.150286	1.285722
gene-AT3G	12.99347	13.69011	11.93438	33.16725	29.89371	31.07152	12.98349	12.74615
gene-AT2G	30.41521	28.73429	29.37675	52.64584	49.86426	49.65151	28.10098	26.6338
gene-AT2G	15.0245	14.02216	15.23654	26.5378	26.90908	24.40975	2.288348	3.669595
gene-AT1G	28.23065	28.3861	28.59204	7.499111	9.960732	7.640063	6.423521	5.927053
gene-AT5G	2.67796	2.577608	2.283189	3.552326	4.002117	4.192513	0.656892	0.639858
gene-AT4G	1.588587	1.666984	1.695732	1.937459	1.780466	1.447438	0.713438	0.738465
gene-AT3G	1.281908	1.245901	0.887696	1.071207	1.412794	1.483872	0.840801	0.862673
gene-AT2G	15.55991	13.34538	15.86874	15.37764	16.34786	17.18335	8.334095	7.528917
gene-AT4G	2.473937	2.28435	2.062886	1.552588	1.770287	2.111446	5.603796	5.163519
gene-AT4G	16.06138	17.87043	14.40423	37.53394	35.39193	37.90195	35.15308	33.65564
gene-AT3G	37.54335	37.08525	36.6194	23.44658	21.32885	22.81709	51.83796	57.94286
gene-AT3G	4.087508	4.727456	4.877777	4.725746	5.881644	5.612628	5.803156	6.96695
gene-AT4G	0.949966	1.81877	1.178749	1.447842	0.955619	0.827662	1.756581	2.176933
gene-AT5G	15.51563	15.50331	13.76334	38.59635	37.32076	38.4697	7.235412	7.567657
gene-AT4G	1.945042	1.372023	1.565054	0.343841	1.352246	0.687936	6.775659	6.552525
gene-AT5G	8.26854	9.359549	9.856206	8.856411	9.709277	10.13979	13.64405	12.67476
gene-AT3G	2.841496	2.892936	3.448733	8.742038	9.565012	8.266017	2.882762	2.52652
gene-AT2G	31.08164	29.83483	32.71653	23.308	23.52408	23.95696	29.24733	31.19397
gene-AT3G	1.739654	1.346125	1.542711	1.776285	2.170685	2.110356	0.856988	0.491567
gene-AT3G	84.0494	80.46906	82.19638	45.5297	42.04967	43.3761	68.80776	71.01265
gene-AT4G	12.32037	12.54248	11.78586	4.917576	5.05753	4.829783	5.110319	4.337922
gene-AT3G	110.6532	110.7929	111.3267	82.18573	79.12807	81.19588	161.3849	169.0544
gene-AT4G	20.8518	19.69291	20.25576	14.56555	14.75362	14.36805	6.783919	6.806501
gene-AT3G	3.807787	3.497064	4.404902	5.36907	5.007049	4.53072	2.695599	2.24328
gene-AT2G	3.934905	5.222246	5.037814	9.739996	9.595057	9.685487	7.978975	7.614288
gene-AT1G	0.636824	0.950991	0.42023	0.251371	0.679918	0.746345	1.501315	1.580891
gene-AT2G	2.223591	2.058801	1.976318	1.209732	1.17885	1.011905	2.851024	2.851015
gene-AT3G	15.08941	18.18047	16.91305	25.88762	19.39442	20.19462	14.16935	12.91626
gene-AT3G	1.722299	2.678537	2.651115	5.255958	5.369499	5.656728	2.910512	3.650836
gene-AT4G	40.9662	38.87135	42.12866	28.11397	28.70602	29.12834	57.77158	58.00848
gene-AT5G	1.314978	1.736339	1.347285	1.177809	1.229224	1.288836	0.567611	0.49002

gene-AT1G	0.108569	0	0	1.47407	1.296419	1.319923	0	0.349332
gene-AT1G	1.643495	1.425384	0.925941	1.900699	1.917231	1.139657	0.528801	0.646557
gene-AT5G	9.997561	9.893018	9.6672	12.93601	13.2292	13.46771	5.221676	5.286228
gene-AT2G	33.63846	34.12173	33.42912	11.77414	10.77855	11.0973	19.57494	20.76417
gene-AT2G	318.2418	340.3711	329.6171	268.785	267.0274	276.5692	259.9096	249.2247
gene-AT2G	10.49852	10.64645	12.5045	8.583422	8.517578	6.60447	17.28549	14.89469
gene-AT5G	3.671736	3.575131	3.842085	3.048314	2.94103	2.418273	1.099746	0.939459
gene-AT1G	105.2459	104.8707	105.4064	61.10035	68.8093	64.95127	198.9212	212.718
gene-AT5G	3.193296	4.920185	5.459694	4.898326	5.646652	3.773471	1.975135	1.923013
gene-AT2G	0.747545	1.582754	1.474787	1.935982	2.115681	2.413942	4.666975	2.969149
Arabidopsi	2.88904	3.518276	3.113549	3.746677	3.535575	4.47525	2.212036	1.854151
Arabidopsi	0.515823	0.524368	0.656291	0.517824	0.239458	0.219087	0.71054	0.891936
gene-AT1G	0.28219	0.581998	0.24644	0.488263	0.478054	0.703102	0.384342	0.078134
gene-AT4G	115.9742	112.51	117.0474	88.66671	90.85085	87.36091	197.8285	198.0263
gene-AT3G	34.11421	33.03605	34.15507	22.33752	21.21639	20.25076	43.90132	46.71557
gene-AT1G	8.915332	9.483534	9.653286	29.84904	29.31971	29.54074	7.291985	6.332104
gene-AT1G	7.074565	8.65525	6.994498	5.76725	6.641548	5.245885	11.45437	12.03998
gene-AT5G	6.803443	6.561124	6.228639	15.97021	14.81616	15.52686	5.06173	5.535668
gene-AT1G	3.429158	3.301911	2.78041	2.678463	3.125688	2.442119	2.274411	3.114216
gene-AT1G	2.656526	2.855857	3.265437	6.588299	6.127799	5.503686	2.89648	2.742055
gene-AT4G	75.20549	72.69058	74.57707	78.54643	74.43071	80.90992	143.0935	144.2106
gene-AT1G	4.247471	4.071672	5.331318	3.902365	4.22116	4.224552	7.06631	6.693134
gene-AT3G	26.49481	28.97392	27.97183	16.75692	16.3544	15.62291	2.709772	3.26905
gene-AT1G	12.95986	11.88311	11.41818	7.512922	6.428817	6.14187	5.616638	7.084459
gene-AT5G	1.657374	1.076454	1.281883	2.464089	2.326072	2.35607	1.01908	0.893563
gene-AT3G	1.212456	0.761793	1.674676	2.271281	2.251611	2.037212	0.994855	1.069787
gene-AT3G	1.818012	1.582217	2.286361	1.387936	1.059467	1.306928	0.566534	0.641565
gene-AT3G	11.81905	12.46073	13.95842	10.29756	9.034276	10.92743	4.790491	5.350289
gene-AT4G	0.696099	1.365625	1.172422	1.387349	1.033919	1.151492	3.733372	3.047935
gene-AT3G	0	0	0	8.433656	8.228928	9.27215	0.256942	0.04355
gene-AT5G	2.125358	2.292224	2.299273	5.401011	4.833704	5.375606	2.368513	3.125924
gene-AT1G	2.798196	2.887139	3.482086	9.775565	10.32439	8.960264	3.884875	2.406793
gene-AT4G	5.286355	6.053688	5.392531	4.476742	4.606322	5.104714	7.088215	8.74995
gene-AT4G	1.07403	0.87197	0.930731	1.5122	1.84543	2.000896	0.665102	0.400821
gene-AT1G	140.9256	141.052	155.5505	312.2749	294.9124	302.9021	61.84706	62.02758
gene-AT2G	4.008318	3.932967	4.501981	6.437745	8.335608	8.692254	12.48908	7.014438
gene-AT4G	11.46456	11.50032	12.26708	7.032151	7.64089	7.555219	11.15262	11.43423
gene-AT1G	43.18202	44.11746	45.94572	37.07781	37.15691	41.07795	64.98634	68.35298
gene-AT3G	1.672189	1.627987	1.385383	1.276206	1.016817	1.894752	1.674507	2.187399
gene-AT5G	0.800483	0.786343	0.614377	1.930238	1.564477	2.135876	0.775801	1.265584
gene-AT5G	81.2577	82.97624	80.19505	40.85836	42.66676	40.97207	103.617	106.3967
gene-AT1G	150.7452	162.4605	150.5652	115.2628	112.7587	109.8826	302.3016	308.1769
gene-AT3G	113.5674	112.187	116.3527	37.78993	37.37767	41.3237	25.86571	27.16477
gene-AT5G	30.41822	26.03896	27.96444	22.84937	21.51933	22.76872	43.05636	44.45821
gene-AT1G	12.62703	12.44204	11.42555	49.75441	45.69052	47.9435	0.506388	0.490345
gene-AT3G	4.148483	4.577436	5.225492	5.4334	4.393315	4.959518	6.530996	6.05124
gene-AT3G	1.415571	0.871467	1.423106	1.631856	0.951276	1.594564	0.619287	0.532436
gene-AT1G	32.43084	28.60374	32.98322	34.51669	41.11875	39.25011	1.555758	1.312672
gene-AT3G	3.209739	4.488559	3.55134	2.406544	3.065568	2.173038	6.608423	7.106295
gene-AT3G	1.313994	2.004414	2.000205	3.662819	4.970858	3.005344	1.745104	2.405965
gene-AT5G	9.53412	9.027601	8.290939	1.467882	2.372408	2.628786	9.46069	9.538734

gene-AT1G	29.8488	29.30475	27.82617	18.20162	19.3844	16.27599	43.8847	43.16113
gene-AT1G	8.6575	7.944255	10.03814	10.86816	10.43126	10.86227	4.807944	4.532636
gene-AT3G	1.254979	1.503298	1.218398	1.788709	1.788787	1.948664	1.011295	1.183118
gene-AT1G	2.77443	3.656134	2.858768	2.91686	2.774093	2.723349	3.868523	3.764731
gene-AT5G	8.05797	7.708161	9.183611	8.405302	8.384871	8.109088	10.24003	11.60733
gene-AT3G	1.881455	1.576965	1.789235	2.542392	1.611045	1.5491	0.619448	0.642285
Arabidopsi	1.89814	1.197344	2.216624	1.062451	0.199081	0.727009	0.73386	0.812307
gene-AT4G	65.1035	60.05837	65.16402	73.28511	81.30655	72.47446	16.82037	17.8339
gene-AT3G	11.94731	14.49016	9.993394	12.83374	12.02523	15.43564	6.46289	10.21315
gene-AT5G	5.937866	5.481662	6.74693	7.75287	7.385385	6.770418	8.043634	8.26494
gene-AT4G	12.85561	13.13092	12.86927	20.77848	20.13567	19.46209	8.863172	9.350799
gene-AT5G	0.618276	0.491483	0.533601	0.259667	0.09368	0.051365	0.058755	0.105347
gene-AT3G	1.538805	1.69364	1.682714	2.392984	2.132928	2.326608	0.771702	0.665459
gene-AT1G	0.526643	0.339836	0.587498	1.670929	1.812041	1.805384	0.614406	0.420797
gene-AT1G	1.116452	1.956896	1.179531	3.096201	3.651768	3.133782	1.251432	1.552222
gene-AT3G	1.235105	0.775078	1.114951	1.084452	1.025073	0.98677	0.428488	0.521688
gene-AT5G	0.077152	0.21726	0.153551	0.639312	0.884296	1.003221	0.316445	0.407831
gene-AT4G	9.625768	9.680099	10.19847	9.86162	10.4998	9.231231	10.05244	11.90001
gene-AT5G	6.506409	5.049131	5.616289	5.880243	6.216355	5.77306	1.547812	1.236801
gene-AT5G	2.359531	3.45661	2.249662	7.488496	4.879961	8.814219	2.755375	1.762758
gene-AT1G	16.93775	13.62834	14.03698	30.31434	32.04628	30.99682	10.39832	10.49111
gene-AT2G	1.482292	2.499316	1.731824	1.887325	2.681162	2.504019	1.037843	0.95012
gene-AT2G	79.71845	80.8762	79.41187	162.7951	171.0533	166.6639	51.47938	51.71636
gene-AT5G	7.411176	8.147703	7.918704	13.48923	12.32993	10.98111	4.526571	4.202335
gene-AT4G	12.33252	11.00233	11.70165	7.857113	7.219201	7.933895	13.68191	14.77886
gene-AT3G	153.3398	151.7863	149.2169	106.0962	106.32	106.0743	285.1908	295.6655
gene-AT3G	5.335	4.378032	5.627454	0.923212	1.053409	0.925677	0.14415	0.231931
gene-AT1G	1.098623	0.48653	1.251632	2.338771	1.601144	1.265383	0.232682	0.149273
gene-AT5G	0.945615	1.104413	0.791479	1.452363	1.082018	0.69137	1.760728	1.868356
gene-AT1G	98.60944	98.60785	94.55443	150.8159	146.1096	147.5677	240.9922	252.0985
gene-AT5G	6.752083	7.46692	5.704369	2.895616	3.988073	4.837659	7.246176	5.906683
gene-AT2G	9.972577	8.959218	10.08504	5.223684	5.696229	5.219125	10.36468	11.36864
gene-AT5G	2.001515	2.752909	2.382103	1.638163	1.670816	1.634556	3.169106	3.906447
gene-AT1G	0.849243	0.299429	0.114281	0.671423	0.696465	0.615942	2.617957	1.703792
gene-AT1G	2.430984	2.155	2.395058	6.619963	4.684587	5.445919	1.886158	1.658369
gene-AT1G	696.9684	678.3795	682.9358	1607.262	1594.923	1585.574	297.2516	298.7573
gene-AT5G	2.090211	1.863854	1.815758	9.028728	9.972136	9.446955	2.138569	2.222008
gene-AT1G	1.476144	3.021355	1.235078	0.330237	0.301546	0.111698	2.74418	3.357693
gene-AT4G	8.967431	8.551414	8.409697	9.906182	9.117174	10.64579	10.14454	11.2163
gene-AT1G	45.06234	46.84804	50.70124	46.89386	44.11497	44.3086	64.20225	67.21156
gene-AT3G	276.2483	276.5656	279.3764	123.5467	131.9478	122.9588	506.6451	509.756
gene-AT4G	139.9052	134.3602	133.3096	77.50214	72.24905	68.29585	241.0836	245.6932
gene-AT4G	32.43491	33.85535	32.30268	12.72682	13.60224	12.75366	38.6761	35.59321
gene-AT2G	28.09987	25.39734	27.29931	22.70055	21.99877	19.45464	43.77353	45.24611
gene-AT1G	0.508213	0.528703	0.307295	0.8091	0.691703	0.673358	0.226472	0.229127
gene-AT5G	3.614964	3.946965	5.390561	4.451794	5.835933	4.602036	5.876965	5.905971
gene-AT3G	37.599	34.85159	39.02493	35.38984	36.7165	36.31365	11.64543	11.57284
gene-AT4G	11.35794	10.59295	10.93814	6.88057	6.347588	6.918264	12.34375	12.81984
gene-AT2G	3.598836	3.419342	4.285113	11.98981	12.01608	11.67201	1.097243	1.707831
gene-AT5G	1.921102	1.226543	1.571666	2.038615	2.435296	1.664956	1.725078	0.828388
gene-AT4G	0.2568	0.51108	0.445877	1.337025	1.661448	1.191381	0.17238	0.059855

gene-AT4G	1.191051	1.322229	1.325858	2.124734	1.648921	2.48926	3.261216	2.790122
gene-AT1G	1.897353	3.188457	3.122309	3.045294	3.169861	2.303032	2.769943	3.53401
gene-AT3G	174.6878	167.9361	179.8517	123.1226	119.3552	116.9809	291.9222	293.0027
gene-AT5G	55.13709	54.95936	57.16	108.9524	104.3779	111.0023	19.13358	19.91111
gene-AT3G	4.114578	3.662711	4.132322	3.390944	3.136289	3.1704	4.326391	4.723715
gene-AT3G	2.89345	3.26603	2.899964	1.339775	0.992346	1.139576	2.017091	1.629544
gene-AT4G	2.189402	2.507259	2.049868	4.378774	4.720031	4.841558	1.931401	1.957505
gene-AT3G	1.074349	0.465591	0.695275	0.473819	0.418334	0.47951	0.96304	0.984277
gene-AT2G	77.02967	78.22878	75.70632	61.43849	63.96554	63.30301	131.1599	137.0185
gene-AT4G	5.216413	3.615348	3.65801	3.586802	3.898681	5.155	6.039288	7.590411
gene-AT4G	12.31953	10.12316	9.976662	11.6841	13.38485	13.55694	24.63102	25.12672
gene-AT5G	22.73282	25.34808	25.6788	23.11046	21.54909	23.29634	21.35305	19.98285
gene-AT5G	13.75402	12.44454	15.93102	24.64952	25.40826	23.55946	8.999223	9.073301
gene-AT5G	14.50867	14.88693	14.94908	10.10962	9.164517	10.24801	18.68955	20.79971
gene-AT2G	4.837661	4.557983	5.508003	12.07309	11.24192	11.23123	6.010673	5.47384
gene-AT2G	4.111349	3.875726	3.432061	5.04686	5.118379	5.688428	1.154679	1.060285
gene-AT3G	2.042933	2.050103	1.661946	1.747376	2.375859	1.527354	0.40423	0.506712
gene-AT5G	138.661	144.312	149.8236	56.30568	53.67587	55.28763	28.96476	26.78649
gene-AT5G	7.147504	7.750426	7.802756	0.917055	1.205864	1.412679	1.089684	1.012582
gene-AT1G	23.19131	25.06201	26.19595	16.65109	16.10848	17.15524	37.65914	41.74913
gene-AT2G	94.48375	124.6479	87.06458	92.24963	97.22811	102.8199	101.2253	101.7531
gene-AT1G	6.987202	7.437706	7.292103	6.143528	7.067044	7.791668	12.00113	13.03899
gene-AT2G	46.58619	43.57323	45.46428	30.02108	29.54955	31.88652	61.15416	61.20419
gene-AT5G	44.95731	42.83564	47.83394	8.449388	6.907037	6.568929	3.958322	4.186562
gene-AT1G	2.915952	3.98783	2.486751	2.743675	2.205901	2.234533	7.063829	6.285383
gene-AT1G	12.01353	12.82356	12.345	9.713017	10.08317	11.60658	24.46793	20.20756
gene-AT5G	10.67571	9.87276	12.71259	5.971402	6.851669	6.762011	10.39427	8.956106
gene-AT5G	2.058783	1.615985	3.071211	1.702757	0.986023	0.768442	4.549916	6.879199
gene-AT2G	2.190733	1.815916	1.725017	2.874428	3.337172	3.534699	0.856097	0.96542
gene-AT2G	1.52622	1.335385	1.615014	3.705823	4.627779	4.317557	1.170408	0.660978
gene-AT3G	7.668114	7.735316	7.342511	7.552894	8.568252	8.012205	7.270152	8.726795
gene-AT1G	7.505597	7.420679	6.751761	15.26582	14.72092	12.97455	5.901984	4.173655
gene-AT1G	12.06986	13.71863	13.63307	15.70174	16.12423	17.39449	3.52681	3.580805
gene-AT2G	8.515854	9.11835	8.310435	5.665496	5.677793	5.597783	19.21016	17.59665
gene-AT4G	2.077381	2.095463	1.367151	4.363511	4.126173	3.725042	1.161392	1.018685
gene-AT2G	0.875747	1.086278	1.319161	0.865222	1.33997	0.859598	0.024844	0.124682
gene-AT3G	40.4584	43.0912	42.88383	40.72652	37.2985	39.08542	66.24384	65.9078
gene-AT1G	1.649208	1.544771	0.961772	0.634537	0.639481	1.299435	3.69095	4.406342
Arabidopsi	17.24554	16.46942	17.48868	8.112123	5.77887	5.345823	1.679972	2.053205
Arabidopsi	1.04317	1.110432	0.827912	1.370049	2.121724	1.597653	0.824328	0.905736
gene-AT5G	8.698817	9.958076	10.0849	7.896764	6.401077	6.476243	10.28657	10.38995
gene-AT5G	22.29873	23.03408	21.29618	94.12387	95.48714	94.77661	33.45905	33.41677
gene-AT5G	1.58061	2.594204	1.800985	2.26022	1.334455	1.46149	5.427491	5.089541
gene-AT5G	19.07686	17.57172	17.32255	11.87683	10.2609	11.18932	23.18962	21.05417
gene-AT4G	9.292048	9.877873	10.38579	10.4844	10.53821	9.504709	17.42019	18.91173
gene-AT4G	5.915445	5.512424	5.976671	8.167348	6.723988	7.629892	2.555657	4.260873
gene-AT1G	5.72641	5.873633	6.661624	6.06686	6.597674	6.880289	13.25376	13.27376
gene-AT3G	9.699609	8.648563	10.74571	7.819396	7.373204	7.935382	14.85039	16.0756
gene-AT4G	50.64656	49.83236	48.65452	93.93752	93.22335	96.57608	34.24318	38.15611
gene-AT2G	27.82372	27.3233	27.29337	8.531984	8.355165	8.539281	9.548257	11.22531
gene-AT5G	0.869799	0.402764	0.409699	0	0.686776	0.094021	2.948431	1.333342

gene-AT1G	5.970642	8.33915	6.428133	14.88136	16.8631	16.20073	3.181614	3.938327
gene-AT5G	0.265766	0.611021	0.374388	0.360883	0.442601	0.353133	0.648809	0.671592
gene-AT1G	2.487457	1.994304	2.429976	2.646907	2.732035	2.426577	0.848945	0.860989
gene-AT4G	31.48101	28.46883	31.48008	21.39533	21.58458	21.4658	26.0323	25.07395
gene-AT1G	0.753458	0.513698	1.162584	0.517981	0.620318	0.445329	0.04215	0.138957
gene-AT3G	9.927203	9.842209	9.941844	12.19605	11.9858	11.19016	13.29012	12.13423
gene-AT5G	196.8156	194.4619	198.3469	129.0633	136.1298	129.9863	170.8179	177.7472
gene-AT1G	2.281681	2.628056	1.965417	6.286345	6.738247	6.970734	5.324949	4.970157
gene-AT2G	0.943866	1.143285	1.462009	1.953166	0.909165	1.542346	0.708086	0.723044
gene-AT2G	2.825169	2.088844	1.875417	3.3007	2.973533	3.274255	0.694849	0.387179
gene-AT1G	77.13511	74.73267	77.82831	105.2224	108.6032	112.8918	54.63276	57.65365
gene-AT1G	5.811949	5.383951	4.778397	9.731098	7.188861	7.286946	7.194148	6.055879
gene-AT5G	64.01515	65.54528	62.77026	34.77458	33.31275	34.28282	65.90955	69.97843
gene-AT5G	1.428715	0.984532	1.048276	3.531047	2.821414	2.817177	6.415377	7.814683
gene-AT1G	31.9869	33.91223	34.06287	31.19422	30.15876	27.67034	51.01457	54.2889
gene-AT4G	38.33857	41.2853	42.70159	37.4698	38.77193	33.49293	67.99299	69.01185
gene-AT1G	0.56534	0.578362	0.390171	0.173505	0.21429	0.344001	0.384262	0.306635
gene-AT2G	0.275304	0.369779	0.223033	1.396645	0.993443	1.277891	0.098492	0.136594
gene-AT1G	7.618435	8.732951	8.388657	5.231153	6.324961	5.056572	8.86654	10.33243
gene-AT5G	451.0073	451.2991	458.6986	285.5111	277.2871	281.9393	613.2103	647.7404
gene-AT3G	10.20213	9.562501	9.659385	10.58286	10.03922	12.23693	7.467791	8.770999
gene-AT1G	1.275069	1.929769	1.45125	0.858296	0.866199	0.529899	1.728543	1.902681
gene-AT1G	36.57892	35.24646	37.39951	34.78267	33.92116	34.74567	51.62833	48.72593
gene-AT2G	2.864817	3.038371	3.31858	5.237278	4.880079	5.221967	2.459817	2.434708
gene-AT5G	5.223569	3.900252	3.950523	2.33601	1.993281	2.360161	4.283279	3.501172
gene-AT1G	11.11148	13.08436	13.23987	17.01037	14.82959	16.7557	4.743824	4.576411
gene-AT4G	41.43515	38.68109	39.63226	28.10638	26.50032	26.84332	59.26531	57.7798
gene-AT3G	99.86804	105.1643	101.3688	281.538	232.9878	274.6467	63.76961	57.14497
gene-AT2G	10.34126	9.530232	10.97876	9.575905	7.186735	8.152546	9.984254	10.84292
gene-AT5G	17.18928	18.32583	18.97772	13.80332	14.27116	15.17149	34.9812	38.15162
gene-AT3G	14.24382	16.06049	16.04471	17.07196	16.39348	13.01406	22.72333	18.73224
gene-AT3G	114.2924	118.0365	113.0493	87.81852	81.8695	84.32192	125.0224	117.7889
gene-AT2G	0.022904	0.041234	0.063383	0.299986	0.226468	0.29185	0	0.221963
gene-AT4G	8.830993	8.296761	8.633078	6.790629	3.710932	4.595032	14.10516	15.13119
gene-AT2G	4.695482	3.910567	4.476471	13.71187	12.53359	12.12585	1.260545	1.587683
gene-AT5G	0.544134	0.707674	0.884806	0.70649	0.582839	0.867876	0.542558	0.421438
gene-AT4G	5.765325	6.852989	6.700244	8.801196	8.388064	9.109639	4.87727	5.064554
gene-AT5G	6.760343	6.395603	6.187629	0.9611	1.367668	1.313212	18.07063	19.32884
gene-AT5G	5.149451	4.297764	4.331788	3.075887	4.824012	5.149477	4.625639	4.879774
gene-AT1G	30.60046	29.67527	29.99824	29.48485	30.995	31.43038	18.28162	17.20983
gene-AT5G	1.516733	0.756233	0.53128	2.048631	1.364434	2.667887	1.521318	1.722537
gene-AT2G	80.29645	79.96716	86.56223	74.63227	76.64893	75.96123	125.3343	134.0232
gene-AT2G	6.137022	6.380472	5.560254	5.132921	4.815961	5.794515	2.495121	2.508185
gene-AT4G	1.640343	1.510405	1.158122	2.776316	1.906009	2.390849	1.17831	0.713332
gene-AT1G	10.52441	10.05949	9.007548	11.27247	10.0287	11.54828	5.67474	7.211028
gene-AT1G	4.256911	4.427154	4.125682	3.054024	3.051046	2.970277	5.160795	5.559144
gene-AT3G	5.995958	4.743521	6.228545	5.15544	5.651539	4.735348	1.193943	1.083483
gene-AT5G	3.097562	4.382395	3.016436	3.806798	4.062201	2.953017	4.565041	7.376395
gene-AT5G	8.314915	9.532602	8.432015	5.66048	6.876508	5.759002	11.04549	12.87825
gene-AT3G	26.32537	31.77578	24.75272	5.676834	8.021722	7.288911	42.83503	42.8588
gene-AT5G	14.04541	12.54288	12.85841	7.197797	6.904353	6.265214	4.492947	5.454981

gene-AT1G	1.986207	1.003786	1.302115	3.17148	2.695388	2.888534	1.316369	1.257332
gene-AT4G	6.390562	6.353763	6.009759	14.41443	13.60001	13.92022	6.114338	5.326625
gene-AT5G	20.6123	16.45738	16.6985	15.9636	20.43881	17.43692	21.36589	21.15862
gene-AT1G	83.4789	84.21875	88.04277	96.64459	100.1846	92.69368	38.4143	39.23362
gene-AT4G	685.7204	828.3209	848.6484	1167.986	1132.918	1109.469	212.8087	230.9399
gene-AT3G	4.727683	4.565224	4.630372	2.873881	2.800003	2.898063	3.838522	4.816231
gene-AT2G	83.02883	79.02368	86.29758	47.00493	46.79763	42.03883	103.0196	104.8013
gene-AT4G	0.665527	0.592108	0.587253	3.980204	4.020837	5.022708	1.971561	2.707599
gene-AT2G	27.78399	26.66562	28.23023	27.7646	27.34316	27.71073	11.27765	11.7362
gene-AT5G	56.85969	55.37903	55.55984	96.31766	96.46556	93.19553	25.0982	28.542
gene-AT1G	2.379433	2.217482	2.550779	8.208716	8.578666	6.744947	2.493633	2.503647
gene-AT4G	150.2917	149.809	150.6683	102.2598	106.7221	100.5645	121.4567	127.1402
gene-AT4G	1.287942	0.417512	0.447148	0.298798	0.470626	0.197858	0.375832	0.14868
gene-AT1G	118.9095	123.2367	114.8082	55.63442	53.36263	55.23463	54.31784	53.71267
gene-AT4G	4.585987	4.664796	5.187714	5.407037	4.200613	5.127885	0.629527	0.639999
gene-AT3G	6.079716	5.423214	5.240958	8.231764	8.669246	7.656477	7.20874	7.336917
gene-AT1G	78.89407	75.40992	81.45259	58.72549	56.93644	55.24713	108.3494	107.1116
gene-AT3G	7.870968	8.547948	8.942986	69.1558	67.84252	67.65563	13.63599	13.83702
gene-AT3G	1.911241	1.116098	1.764014	0.934156	0.657952	0.804393	1.442751	1.472847
gene-AT2G	0.617976	0.973758	1.046004	0.295146	0.18205	0.377831	0.147595	0.567368
gene-AT2G	1.113064	0.797885	1.132051	1.123147	1.041512	0.923465	1.362952	1.769747
gene-AT1G	11.21443	11.83751	10.78316	22.5733	20.91351	24.05141	12.87472	12.94121
gene-AT3G	0.940403	0.557283	0.850362	1.208991	1.930106	0.863773	1.069505	0.429313
gene-AT5G	87.58336	88.05029	84.46169	67.08877	66.50412	66.6371	129.7883	132.3804
gene-AT5G	8.686852	8.275704	7.826109	11.60699	12.577	11.25787	4.225445	3.252002
gene-AT1G	16.30213	15.86315	15.7212	7.476542	7.11709	6.336966	7.978549	8.495809
gene-AT4G	0.875578	1.891117	1.681792	1.51364	1.327306	1.032161	1.354758	1.819331
gene-AT3G	44.97355	44.9643	44.13492	51.15878	52.79062	49.95669	12.75084	12.7284
gene-AT1G	4.184945	4.160312	4.55048	3.883629	2.434466	3.442037	1.231589	1.422582
gene-AT3G	5.467465	5.670117	5.812169	7.562107	5.285794	4.834544	6.234491	7.923234
gene-AT5G	13.01707	11.66264	13.36544	5.078324	3.424859	5.534971	11.45257	11.03196
gene-AT4G	7.915805	9.973156	8.698552	1.044897	2.435972	2.683901	3.338237	2.934289
gene-AT3G	11.50201	10.84061	11.93733	7.310994	6.984053	7.23642	16.84678	17.7957
gene-AT4G	13.51958	14.69049	9.909803	10.6693	10.22271	9.713915	16.2069	17.48206
gene-AT1G	21.33312	20.21263	20.16043	20.75066	19.78425	18.4866	6.296334	6.712722
gene-AT4G	7.070983	6.603382	5.693796	2.037422	2.62619	1.907404	17.45156	14.59112
gene-AT4G	0.151271	0.215618	0.241806	0.704793	0.493409	0.548897	0.163084	0.076595
gene-AT1G	5.41187	6.289166	5.755556	23.40027	22.31404	23.15114	2.683705	2.539006
gene-AT1G	7.043679	6.822245	8.378518	17.32839	16.60626	15.1039	6.37733	6.629183
gene-AT3G	2.062351	1.784169	1.452352	2.84932	2.39871	2.1505	0.76333	0.918998
gene-AT5G	1.111504	2.299275	2.515895	1.313665	0.777952	1.208068	4.179986	4.564748
gene-AT4G	0.89781	1.097577	0.786105	4.016378	3.75729	4.257503	0.133396	0.046817
gene-AT3G	2.996623	2.308069	2.093615	3.325975	2.029403	2.530168	3.718609	4.268057
gene-AT2G	6.1967	5.606705	5.2456	8.11764	6.320878	5.806992	5.608886	5.90767
gene-AT3G	1.296159	0.894758	1.502266	1.645687	1.833235	2.39706	0.785013	0.959656
gene-AT3G	1.926415	2.158597	1.881341	3.465208	4.345293	4.12036	1.182947	1.319116
gene-AT2G	28.84888	34.39649	29.177	8.731274	8.925735	9.48994	1.458855	1.136528
gene-AT1G	27.21524	24.7592	25.80825	29.80435	31.10265	32.8282	19.85185	21.85673
gene-AT5G	8.681129	6.704908	7.673577	9.458743	12.22496	10.05617	11.53613	13.21646
gene-AT1G	11.13626	12.67565	11.32509	12.18655	12.25977	12.59659	22.27103	21.36789
gene-AT4G	4.82385	4.352551	4.458405	2.631	1.814051	1.763348	15.57732	16.52769

gene-AT1G	1.047271	0.644343	1.005816	1.616965	1.650898	1.660785	1.662042	1.535506
gene-AT4G	1.273199	1.138333	1.200957	3.999157	4.466753	3.794039	0.579186	0.388225
gene-AT1G	4.094305	3.42017	2.784681	2.715283	3.390206	2.706867	1.496868	1.008656
gene-AT1G	0.642067	0.641473	0.522785	0.936538	0.802027	0.813172	0.545098	0.61934
gene-AT4G	2.537985	2.783786	2.393747	5.004649	5.466257	6.775708	2.027735	4.52304
gene-AT1G	47.05896	48.30904	44.5452	39.86893	38.43887	38.988	88.47121	86.79497
gene-AT2G	0.315313	0.488523	0	0.154476	0.260663	0.177609	1.099122	1.111462
gene-AT2G	31.36671	32.16028	30.32848	16.93429	16.47948	16.00057	27.84828	29.88009
gene-AT3G	4.99724	7.381954	6.466906	17.56762	16.39071	17.68341	5.96928	5.164434
gene-AT1G	9.582853	8.748818	10.7523	3.110251	4.061276	3.537549	3.160281	3.421611
gene-AT5G	54.87648	56.68634	54.22635	161.3423	155.1803	160.0051	44.72705	48.28165
gene-AT4G	0	0	0	0.569572	0.143751	0.218303	0.328556	0.168388
gene-AT5G	2.048185	2.432009	2.77494	2.221235	2.236392	2.798826	1.090266	1.14233
gene-AT2G	1.746852	1.694022	1.979682	2.824111	2.911877	3.253115	1.830071	1.363693
gene-AT1G	13.65051	13.09245	13.90158	7.999105	7.46485	8.409956	5.401294	5.583907
gene-AT3G	24.83309	23.18369	24.19968	42.45927	43.74346	42.73673	3.670563	2.528572
gene-AT2G	28.88804	28.4649	35.7903	27.73108	24.77494	24.3561	40.04164	42.3811
gene-AT2G	606.3157	624.4196	623.4678	414.6562	400.0491	416.9147	705.0584	707.2297
gene-AT1G	10.41332	10.41098	10.43853	9.17343	7.85617	7.331335	6.781236	5.864751
gene-AT1G	5.388795	6.550874	5.633239	6.746622	6.741362	7.014853	0.195384	0.29352
gene-AT2G	4.504639	4.279534	4.962198	12.61572	11.45542	13.718	2.990765	3.325043
gene-AT2G	19.09578	17.73263	17.97481	3.951711	4.813037	4.962785	1.232712	2.000117
gene-AT1G	2.067672	2.228882	2.696003	2.461142	2.286518	2.190391	1.95496	1.685851
gene-AT3G	211.4514	207.3948	218.74	117.9744	123.0638	118.9231	308.3683	305.9426
gene-AT5G	5.47516	5.929694	4.486929	9.306024	11.00984	8.6896	1.778313	2.11782
gene-AT5G	2.634395	3.19553	2.041675	9.564481	8.412908	11.79364	0.92536	1.081646
gene-AT3G	9.143353	7.905287	9.79412	8.427928	7.548904	6.290943	9.566323	12.27962
gene-AT5G	3.458726	3.689056	3.379986	3.961268	3.135869	4.174383	1.164036	1.188317
gene-AT5G	5.565634	5.456297	5.391998	6.965331	7.443648	7.386109	1.563181	1.811639
gene-AT5G	25.4569	25.78411	24.83529	13.20384	13.08952	12.55314	24.09843	25.41754
gene-AT5G	5.298836	4.122728	5.864056	2.17676	1.859219	1.860354	1.602685	1.215745
gene-AT2G	3.689724	3.936814	4.3215	1.840817	2.230853	1.861171	9.545164	8.589792
gene-AT1G	19.19241	19.53024	18.98024	15.15498	15.94248	15.26163	8.34158	8.547825
gene-AT3G	58.35242	58.19831	59.14277	41.10254	44.2858	43.90068	92.81669	94.49561
gene-AT5G	2.089431	1.88193	2.44231	0.545601	0.472481	0.707259	0.47882	0.242308
gene-AT5G	0.452385	0.517389	0.590446	0.364139	0.403762	0.259946	0.157739	0.066414
gene-AT1G	3.980483	4.303843	4.795775	2.855692	3.065114	2.253589	5.414568	4.984304
gene-AT5G	2.537724	1.640375	1.628632	1.590229	1.846329	1.478614	2.583741	2.443338
gene-AT4G	7.887334	10.8783	7.166998	5.67816	4.055757	3.706104	10.68234	13.08948
gene-AT4G	4.916228	5.135337	3.998281	6.19943	6.865691	6.868447	3.110872	2.306367
gene-AT4G	9.246504	9.491374	8.261786	13.61538	14.22619	12.3798	6.945885	6.595868
gene-AT1G	41.93664	43.64871	43.2852	34.32613	34.16756	33.09825	23.83197	21.23885
gene-AT2G	14.50398	17.0385	15.6361	5.00613	4.515672	5.54407	7.913252	8.159175
gene-AT1G	5.5654	5.317113	5.891223	4.440795	4.478766	3.992476	2.448938	2.727639
gene-AT1G	9.924378	9.56974	9.717444	20.79418	26.58384	21.11657	3.277142	2.936569
gene-AT3G	14.5678	14.83361	14.52325	20.70235	19.91884	18.93872	10.11207	9.731744
gene-AT1G	0.693136	0.9648	0.822096	0.485756	0.608259	0.804102	0.242036	0.23181
gene-AT4G	24.08028	21.75827	22.92919	1.456785	0.735308	1.164917	7.530301	7.870212
gene-AT5G	11.46463	10.99959	11.16128	13.46133	14.18293	13.77964	3.018959	2.941255
gene-AT3G	2.101914	1.143379	1.204409	0.123816	0.302945	0.198333	0.260373	0.268728
gene-AT1G	0.403811	0.423321	0.406138	1.083853	1.134795	0.765421	0.254397	0.206917

gene-AT3G	22.41077	22.12371	21.47395	19.00658	18.28767	18.606	45.57571	48.84157
gene-AT1G	0.997031	1.053504	1.089256	0.367604	0.352676	0.604043	2.391669	2.548141
gene-AT4G	4.471145	6.067185	5.856206	75.64537	77.97397	75.80382	0.499228	0.776036
gene-AT5G	2.540148	2.867023	2.813338	6.137823	5.421689	5.500127	2.535728	3.073421
gene-AT3G	15.14432	16.6395	15.94923	13.45898	14.32513	12.25088	1.970025	1.379066
gene-AT3G	6.43795	7.440542	4.667003	6.143862	7.102166	7.459996	2.8756	2.77919
gene-AT4G	3.279742	3.278146	3.258607	2.018742	1.496503	1.525608	9.483578	10.88351
gene-AT3G	1.891577	2.117975	1.927312	1.736201	1.894287	2.010674	0.739675	0.408771
gene-AT2G	0.870101	0.592115	0.889552	3.576362	4.458937	3.425652	1.583874	1.122432
gene-AT3G	0.693039	0.947939	1.169016	0.194522	0.238924	0.589249	0.416951	0.322916
gene-AT3G	3.045535	3.507913	3.667198	5.141983	5.187526	5.68671	0.288846	0.247977
gene-AT1G	1.945904	1.708723	1.305314	4.126671	3.92182	3.683833	1.704052	1.51723
gene-AT3G	4.768314	4.064407	4.830315	4.161464	3.032012	3.825344	5.765775	5.110196
gene-AT5G	1.536916	1.489585	1.67134	2.057784	2.098704	2.752154	0.838198	0.709421
gene-AT4G	2.302272	3.488596	3.958975	1.97753	1.51223	2.278442	2.553607	1.986633
gene-AT5G	11.79523	12.55266	12.5827	17.76849	19.87269	19.47951	4.85381	4.364226
gene-AT4G	19.86213	19.51591	19.89717	18.34013	18.07857	18.00775	10.40186	10.26523
gene-AT1G	20.22412	18.11403	20.15676	38.93092	39.26868	40.67577	10.95175	10.6351
gene-AT1G	10.44531	8.959176	9.724263	6.000513	5.651363	6.05514	3.070931	3.444926
gene-AT4G	73.58617	75.06811	81.9025	47.43388	46.32304	46.05757	103.985	100.5352
gene-AT5G	11.97502	12.3541	10.73593	9.237912	8.250115	8.64625	2.768502	2.326168
gene-AT3G	1.538103	1.776522	1.852152	1.358967	1.276582	1.331225	1.551321	2.43151
gene-AT1G	307.7671	301.3765	303.5551	186.0346	192.5416	195.1127	304.5864	321.7896
gene-AT1G	25.29468	24.26692	24.89401	42.76046	43.17371	42.74357	20.00435	23.2993
gene-AT2G	18.17192	16.47364	16.98552	17.86559	15.99366	17.5084	7.18874	6.717691
gene-AT5G	1.610361	0.833826	1.389481	3.208079	2.786225	2.654105	2.758735	2.489919
gene-AT5G	9.542905	8.903517	9.350346	10.6134	9.605449	10.09568	5.854884	6.556576
gene-AT1G	2.631526	3.206234	2.186066	7.634333	6.926731	6.318291	4.214902	5.025376
gene-AT1G	3.051038	2.782934	3.201448	10.57065	10.37255	10.80775	0.651557	0.329776
gene-AT2G	22.30599	26.37911	26.62682	19.93675	18.81558	22.01522	2.131234	1.675709
gene-AT1G	8.979109	9.991382	9.473454	7.410184	7.038472	8.160261	4.035533	4.885821
gene-AT5G	41.36695	42.88378	44.52168	37.67144	37.29126	36.94763	47.21835	48.71236
gene-AT4G	0.229386	0.23819	0.132242	1.925082	1.338629	2.109156	0.199085	0.094018
gene-AT4G	34.52093	32.63948	32.37368	85.93707	87.67185	81.81699	29.81205	30.50719
gene-AT2G	88.35038	87.16287	86.15348	46.88477	48.74141	48.99008	141.7765	147.5929
gene-AT1G	19.53296	21.85892	18.33627	17.1804	16.6208	16.32996	22.66706	25.8341
gene-AT3G	17.10659	16.07901	16.60613	8.377954	9.385268	8.285219	26.03922	26.2727
gene-AT1G	12.52083	12.47369	12.20823	11.76417	10.98457	12.82239	7.068053	7.086267
gene-AT3G	1.338036	2.176766	1.605444	3.157027	2.230976	2.061283	0.256094	0.337898
gene-AT2G	2.394168	2.158183	2.579421	0.916328	1.352813	1.198166	2.226896	2.852898
gene-AT1G	0.282688	0.269864	0.19132	0.505716	0.340329	0.609506	0.168161	0.165113
gene-AT2G	0.300126	0.193596	0.139904	0.784411	0.376088	0.250893	0.371959	0.540194
gene-AT5G	115.3395	110.0907	117.4509	128.9634	128.168	124.6088	59.42902	62.2581
gene-AT1G	25.91527	23.80421	27.02389	37.89845	40.25607	41.90172	8.185375	6.23809
gene-AT4G	2.168662	1.36982	2.250612	0.087056	0.292796	0.140675	3.214536	4.404974
gene-AT1G	9.815753	9.568322	10.28834	51.91375	54.38612	50.16687	7.986082	9.155184
gene-AT5G	2.576433	4.103028	3.099971	2.78012	3.194594	3.498747	1.321832	0.568592
gene-AT1G	53.14431	51.34826	55.94223	80.06463	78.55464	81.07629	16.88782	14.89375
gene-AT1G	32.67955	33.05302	32.51493	22.82365	22.18367	21.15573	13.16937	13.60507
gene-AT5G	0.502004	0.259377	0.450603	2.137267	2.410576	2.309908	0.18149	0.140454
gene-AT3G	1.549309	1.579536	2.717658	0.354713	0.405299	0.336774	1.906185	1.939014

gene-AT3G	88.27309	87.8097	94.64634	182.0564	184.1241	176.8323	125.0323	134.2802
gene-AT4G	2.214092	1.785897	2.052672	2.626269	2.655918	2.591779	2.815459	2.741357
gene-AT1G	21.10712	22.07855	22.08746	12.98125	14.14075	15.7864	4.186173	4.130345
gene-AT4G	1.410797	1.896716	2.103931	1.736214	2.19111	1.756057	3.765057	3.997691
gene-AT4G	3.354602	2.386545	3.126318	1.887745	1.795607	1.738632	0.542273	0.683013
gene-AT4G	3.512589	3.139295	3.807026	2.434434	2.056872	2.34379	11.34762	8.070878
gene-AT2G	4.486285	4.193516	4.825664	2.99126	2.727397	2.848559	7.481285	8.1647
gene-AT1G	0.370312	0.375146	0.28253	2.732824	2.75939	2.907631	0.112438	0.077912
gene-AT3G	59.18083	62.49655	60.91598	160.7409	155.0621	153.596	19.55553	20.62196
gene-AT1G	3.119463	2.31839	3.060281	1.936818	3.151166	3.827552	0.63428	0.565429
gene-AT3G	2.538335	2.246317	2.314303	1.793554	2.728482	1.832113	3.737349	3.931722
gene-AT1G	1.751259	1.745509	1.346569	31.12954	33.16046	33.77759	2.632797	2.418012
gene-AT2G	3.198945	3.144655	3.557896	4.532994	5.197729	5.388395	5.108987	4.904132
gene-AT4G	4.090202	4.257478	3.92391	3.803481	4.849055	4.879878	12.68409	14.1806
gene-AT1G	1.882643	3.15893	2.177191	3.787316	2.564493	2.894004	1.290694	1.202896
gene-AT4G	8.349307	8.085223	7.979705	4.160504	4.258081	4.831117	9.157098	9.840053
gene-AT4G	18.45645	20.90808	22.68794	40.32805	37.19291	41.81291	37.68416	38.44519
gene-AT5G	48.78189	53.04922	52.37593	40.34816	39.20494	39.80388	73.34827	79.2228
gene-AT5G	57.04583	59.45328	56.64206	9.511394	9.873662	9.589201	52.44039	53.40701
gene-AT5G	5.762981	6.621428	6.514755	9.433785	9.633716	9.462988	5.61304	5.729229
gene-AT1G	172.958	177.949	179.1854	225.7886	227.7384	211.9069	77.91079	78.73621
gene-AT3G	15.452	14.07415	13.74178	10.18153	11.19692	10.86394	8.623512	8.204519
gene-AT3G	70.87007	66.13186	67.74613	32.12917	29.55731	29.50722	67.11335	67.03948
gene-AT1G	34.16192	29.89641	31.42928	5.108904	6.268194	5.490467	13.11397	13.50496
gene-AT2G	1.376249	1.556695	0.759536	2.448583	2.813958	2.204492	1.094357	0.734688
gene-AT4G	3.425348	3.440747	4.064689	5.228843	6.739666	5.625186	1.416156	1.101678
gene-AT4G	180.2184	174.0484	177.9958	316.4685	316.8231	308.8623	71.48381	72.27723
gene-AT4G	52.85554	52.63859	48.59414	102.0555	104.4726	102.5404	8.748495	7.550005
gene-AT4G	19.81462	21.44668	21.43373	13.66677	12.40301	12.44491	33.7838	29.96688
gene-AT4G	19.04598	16.03108	18.38324	70.46896	67.0708	70.36822	4.641106	5.006023
gene-AT1G	148.9786	144.2223	146.981	71.83268	71.5284	71.29619	71.11787	78.91796
gene-AT1G	3.75393	4.180713	4.057785	1.217924	1.032082	1.622108	12.08733	10.92862
gene-AT5G	43.64361	44.20835	40.94102	33.14653	34.31546	33.34547	13.55501	14.69458
gene-AT3G	14.97371	15.46061	15.21164	6.373954	7.416121	6.035204	5.049505	4.850725
gene-AT4G	31.66247	29.41517	31.3159	38.24654	38.46088	36.44822	54.85716	58.05124
gene-AT5G	13.54975	16.30685	16.3064	70.27564	72.80904	69.56139	5.319293	5.424184
gene-AT3G	0.291196	0.334478	0.236616	1.603295	1.431372	1.620846	0.094409	0.049524
gene-AT3G	3.104207	2.347606	3.033759	1.408332	1.423154	1.424016	5.113554	4.004742
gene-AT5G	53.14988	53.81491	53.00829	76.7955	78.34844	79.55985	26.66785	26.11474
gene-AT5G	0.871346	0.705004	1.706052	1.198314	0.756928	0.905431	1.96855	1.920569
gene-AT4G	15.71542	14.07796	16.00926	4.117281	4.954665	5.241156	30.68857	32.18568
gene-AT2G	87.7051	91.60822	83.86542	55.64203	60.43066	55.66222	163.5229	155.1709
gene-AT2G	2.725338	1.345981	2.382014	1.003724	1.679446	1.123989	0.369468	0.66421
gene-AT3G	0.178265	0.175774	0.145996	0.236001	0.177717	0.30195	0.067467	0.148404
gene-AT1G	0.584466	0.680387	0.793342	0.255707	0.275495	0.402657	0.150438	0.09041
gene-AT2G	3.269484	3.724765	3.539801	4.89081	4.870015	4.48645	6.076613	6.874629
gene-AT1G	12.88825	13.03423	13.08224	15.92076	15.57572	15.78643	4.353055	3.843018
gene-AT1G	5.559321	5.997516	4.89963	4.485538	4.52736	4.709357	5.528561	6.181873
gene-AT2G	3.677445	3.800246	3.898998	7.835356	7.275101	9.608665	3.04323	4.380373
gene-AT5G	11.16078	11.68771	10.74278	7.975955	6.03602	7.961037	5.275034	4.848233
gene-AT5G	1.341709	2.27918	2.251411	1.037535	0.893236	1.397524	0.573428	0.426378

gene-AT5G	0.253943	0.265768	0.152327	0.778933	0.949538	0.883647	0.334764	0.417591
gene-AT3G	5.532495	4.732358	5.29665	5.471867	5.876683	5.125747	1.659891	1.707389
gene-AT1G	23.34655	24.15889	25.41111	19.18938	18.4118	19.9397	39.23251	40.8832
gene-AT2G	18.15825	17.70086	16.09864	10.97981	11.6532	12.24255	17.15545	17.10716
gene-AT2G	5.072026	5.173465	4.569276	4.315503	4.491175	3.02079	6.787857	7.351562
gene-AT2G	2.314391	2.369233	1.91317	2.207469	2.74204	2.492508	4.035818	3.286286
gene-AT4G	19.69508	19.97472	18.55296	23.49479	23.87814	22.17581	20.98275	20.89792
gene-AT4G	50.68051	51.966	50.60551	51.39696	53.24507	50.97035	32.32149	35.70048
gene-AT1G	1.257476	1.03754	0.921035	0.792103	1.38241	1.939721	0.637963	0.793238
gene-AT4G	1.009476	1.277981	1.01459	1.834727	1.646946	2.259343	1.438571	1.152809
gene-AT3G	17.72667	18.94062	17.38812	8.999178	8.050477	8.497509	10.19363	11.19536
gene-AT4G	72.00916	81.72801	74.71846	47.09882	42.19861	43.21503	124.3835	112.1814
gene-AT4G	5.482555	5.196098	6.159425	5.351021	5.016238	6.393333	1.86965	1.999117
gene-AT4G	6.37633	6.870478	7.974056	5.161831	4.502767	5.887541	10.34877	10.27681
gene-AT5G	1.632169	2.092194	1.875438	2.333015	3.05154	1.833074	2.438973	2.758876
gene-AT1G	14.13824	13.17938	13.0282	22.03026	21.627	24.16172	1.018825	1.093104
gene-AT4G	1.007331	1.429546	1.473303	3.316469	3.241593	2.78173	1.212755	0.991989
gene-AT4G	2.158979	1.153797	1.19636	2.753419	1.050012	2.233993	0.784242	0.858979
gene-AT4G	2.359569	2.01614	2.238061	3.13105	2.401435	2.452542	3.214197	3.44018
gene-AT1G	19.54001	18.04431	17.96284	14.03838	13.08881	13.24542	20.52208	22.33477
gene-AT2G	5.708704	4.932903	5.027042	3.558394	3.086579	3.27494	8.178931	8.10756
gene-AT1G	27.21036	28.27611	28.92663	22.43733	20.73959	21.7052	38.81025	36.23926
gene-AT2G	11.78498	11.32767	10.66158	9.371956	10.65449	9.87444	2.826353	2.705743
gene-AT5G	10.59212	8.125473	9.190034	14.94029	14.27729	15.72382	5.722138	4.44439
gene-AT1G	0.577515	0.573586	0.494788	1.251814	1.035003	1.541385	0.456898	0.288486
gene-AT2G	0.458427	0.559863	1.349986	1.973145	1.583034	2.165203	0.332286	1.163447
gene-AT1G	29.30533	31.39261	30.26842	22.03792	21.68749	20.03409	42.99614	47.9235
gene-AT4G	6.782059	6.666891	6.774395	13.32095	12.39676	14.32032	5.773073	5.812922
gene-AT3G	14.74063	14.01702	14.66789	6.101903	6.414115	5.943805	5.083961	5.142322
gene-AT1G	25.42255	24.8772	24.57689	17.9366	18.72546	17.30938	7.857248	8.222155
gene-AT5G	4.587234	3.823997	4.104329	8.225317	8.173305	8.518418	2.254966	2.685896
gene-AT3G	8.500097	9.107533	8.503284	4.682011	5.711457	4.986466	16.5088	15.93495
gene-AT3G	0.424212	0.767279	0.512321	1.874697	1.414748	1.407479	0.318107	0.125704
gene-AT5G	3.596234	3.523565	3.108067	3.122273	2.777588	3.077951	1.18055	0.745175
gene-AT2G	6.603308	7.404022	7.206143	3.540949	2.21174	3.382478	15.29411	15.72954
gene-AT5G	3.703082	4.250196	4.420107	6.474813	7.924607	8.002087	5.015672	4.019212
gene-AT4G	10.26465	11.24971	11.93476	9.709323	10.08075	9.958501	12.01455	12.10949
gene-AT4G	5.571002	4.700742	5.827211	6.518567	8.423331	7.413148	2.918302	2.900579
gene-AT4G	4.774577	4.750597	4.146263	2.927862	2.547201	1.974998	12.79046	14.0167
gene-AT3G	50.20294	46.89544	48.34224	77.77727	72.32652	75.49835	38.56211	39.89164
gene-AT4G	2.340248	1.800956	2.263033	2.264148	1.953461	1.398152	2.196636	2.167464
gene-AT5G	13.5262	14.55065	13.25804	4.841312	5.033219	5.100065	4.916567	5.855183
gene-AT1G	0.509954	0.290399	0.205507	1.117487	1.031294	0.799177	0.357148	0.181457
gene-AT4G	2.00894	1.357928	1.820904	2.590075	2.305269	2.304187	2.114633	2.491222
gene-AT1G	1.920038	1.996348	1.855724	2.823375	1.990606	1.97801	4.089123	5.242454
gene-AT1G	41.25409	38.87572	40.24316	46.02699	39.38012	39.3934	70.42818	77.74759
gene-AT2G	22.33949	21.03169	19.31088	18.29982	16.83678	16.68962	21.9691	22.78097
gene-AT5G	11.09399	10.12489	9.996592	3.746678	3.000212	3.388884	0.739344	1.192144
gene-AT4G	5.357222	5.338165	5.498268	2.830002	2.307288	1.867784	8.472492	9.130776
gene-AT2G	7.830873	7.990644	6.86322	6.47997	6.535813	6.685733	2.396012	2.546844
gene-AT3G	0.864593	0.584432	0.554964	0.686816	0.994665	0.840952	1.023526	1.008728

gene-AT1G	113.3692	113.2834	118.948	74.3802	72.68908	71.43181	210.2322	202.4324
gene-AT1G	8.780987	8.945162	7.738534	1.832027	1.478595	1.986099	16.12842	17.06142
gene-AT1G	228.7943	225.071	231.3378	76.77666	74.74411	75.59014	211.4576	216.4983
gene-AT1G	7.007991	6.276791	6.618219	8.400372	9.36274	8.663034	1.814195	2.268813
gene-AT5G	37.69272	40.6033	40.49655	30.69027	29.82949	27.53706	70.55961	69.86164
gene-AT1G	5.132736	4.495451	4.291402	4.384682	3.687958	4.421037	1.374909	1.619321
gene-AT1G	6.472301	4.921821	5.387555	4.140695	5.376596	5.071204	6.322386	6.715075
gene-AT5G	0.505128	0.223897	0.324454	0.497004	0.487577	0.67928	0.488117	0.750439
gene-AT4G	1.352944	1.285926	1.456866	2.478723	2.260092	2.275751	3.354397	4.652006
gene-AT1G	140.5515	142.8959	134.0645	65.98017	63.02538	63.19896	80.03307	80.02294
gene-AT1G	260.848	265.7116	276.3699	106.1998	112.6665	103.9591	425.1589	456.3026
gene-AT3G	17.02376	17.10127	15.19601	13.61084	11.49314	12.64682	5.395967	4.334558
gene-AT3G	4.365957	4.454659	4.269442	4.33608	2.752473	2.957194	10.37383	11.12597
gene-AT1G	2.526042	5.670803	6.61381	6.06629	6.073918	3.481327	4.535364	5.93056
gene-AT5G	1.66459	1.557863	1.260023	40.74191	39.83371	38.89686	4.396162	4.180395
gene-AT4G	3.020741	2.683676	2.675936	1.084471	1.155278	0.70266	0.992137	1.423102
gene-AT3G	91.31735	94.15797	92.31922	51.68965	50.22654	48.59432	129.4209	130.5454
gene-AT3G	1.54177	2.343645	2.87356	5.37175	3.838384	4.197517	2.172076	2.128185
gene-AT2G	1.939618	1.949446	1.976125	3.776412	2.980585	3.152065	0.762423	1.12208
gene-AT2G	1.554664	1.422991	1.804522	1.346619	1.76294	0.918575	0.773686	0.831484
gene-AT3G	34.90525	35.22483	35.59601	23.04101	22.02845	22.98758	29.47317	32.30251
gene-AT5G	2.381258	1.36052	1.540245	0.890279	1.080044	0.588392	5.349545	6.833795
gene-AT5G	4.308704	3.865377	4.850061	2.589824	3.770738	2.739349	6.715037	6.851892
gene-AT4G	12.52967	10.92447	13.10147	7.891303	6.648906	6.885284	0.712745	0.623205
Arabidopsi	0.150405	0	0.190295	2.222042	1.607054	1.361195	0.85145	0.310381
gene-AT5G	37.07842	34.4007	36.33821	34.36533	34.26476	33.47515	133.9872	133.9765
Arabidopsi	2.311328	1.962825	2.511036	5.337193	5.853403	6.813074	0.850656	0.581942
gene-AT2G	4.697524	6.70847	6.577118	9.274863	10.10968	9.983872	3.627855	3.42259
gene-AT2G	10.68841	9.110562	8.518053	9.93263	7.947296	8.776692	4.010368	3.29677
gene-AT2G	1.853855	2.226355	1.615904	3.42969	3.714836	2.701703	1.420633	1.362185
gene-AT2G	2.116633	2.479313	3.199029	3.679879	3.177859	2.44227	1.795474	1.342919
gene-AT5G	17.02451	17.35982	19.22587	29.5795	30.82233	27.80075	14.42476	13.32748
gene-AT4G	0.784874	0.472984	1.007609	1.712752	1.812625	1.80547	0.621638	0.79225
gene-AT3G	3.646758	2.735861	2.855185	23.40388	22.96519	21.49087	2.986997	1.948402
gene-AT1G	10.47882	13.82947	10.37439	5.719496	5.535072	6.993582	16.81249	17.54655
gene-AT1G	17.42235	16.49076	18.11896	10.65109	10.74355	11.73312	33.19881	32.78871
gene-AT1G	0.393705	0.13767	0.112614	0.172365	0.203216	0.096241	0.123429	0.073414
gene-AT1G	15.31869	13.82721	13.92289	7.193378	7.804772	7.275958	20.80771	19.68221
gene-AT4G	1.183458	1.439458	1.036476	0.908386	1.382888	1.184358	0	0
gene-AT2G	4.523116	4.003688	4.272412	4.4184	2.768098	3.967254	8.04608	5.398529
gene-AT4G	1.550518	1.241796	1.290061	0.992043	0.902556	0.93273	2.188912	2.157552
gene-AT1G	7.831491	7.749019	8.64141	6.785401	6.62469	7.198233	3.075004	2.609977
gene-AT3G	0.387762	0.270185	0.341277	0.201308	0.260754	0.249927	0.013301	0.015361
gene-AT4G	12.42097	12.07929	10.7445	6.535381	5.249127	5.527394	2.454353	1.981535
gene-AT2G	14.28811	13.72705	13.66187	7.835722	6.729169	7.686302	31.25071	31.84857
gene-AT4G	17.28322	16.65728	17.0771	12.8229	12.07101	12.3891	4.338346	4.939822
gene-AT5G	1.3331	0.930656	1.182252	1.445385	1.969508	1.482547	1.885417	1.928431
gene-AT2G	13.94274	16.05257	17.48015	51.07243	49.27056	49.77425	14.48225	13.73599
gene-AT5G	0.751977	0.537293	0.509144	1.214521	0.753476	1.063107	1.424578	1.063555
gene-AT4G	0.512459	0.431908	0.356486	0.978741	0.92846	1.328108	0.345137	0.391411
gene-AT1G	11.00143	9.100661	11.66638	14.49565	14.87973	13.52331	0.285199	0.430523

gene-AT2G	17.83556	16.18701	15.64795	30.36047	28.93901	32.77891	18.68904	17.13706
gene-AT5G	7.492956	9.097179	8.418453	14.61703	12.7781	12.62284	15.24161	14.80919
gene-AT1G	222.8791	212.262	231.213	152.356	143.8038	151.7167	223.7921	235.0898
gene-AT4G	19.98461	19.32733	19.72902	24.00074	24.18233	24.11729	11.92815	11.86252
gene-AT1G	7.237309	6.335143	6.615592	4.120995	3.211979	4.054626	2.274423	2.778097
gene-AT5G	9.446058	10.35341	10.82159	9.061413	9.492091	10.10316	9.825266	10.13194
gene-AT5G	8.25295	7.088247	9.613421	7.450224	5.993733	6.541034	5.570065	6.052704
gene-AT3G	9.318891	10.98044	11.42515	12.13094	12.45413	13.1065	4.654674	4.207104
gene-AT2G	3.25049	3.139377	2.366425	0.849084	1.207552	1.421435	4.052607	3.75231
gene-AT3G	34.86041	32.75583	34.43776	31.67175	28.92252	30.14005	38.03082	37.50739
gene-AT1G	11.75803	11.4762	13.23787	11.15568	11.72143	9.438227	26.05913	23.18747
gene-AT5G	28.56631	29.13995	31.54523	18.97286	20.73253	21.14549	49.95794	51.77917
gene-AT5G	4.789648	4.206481	3.96461	12.49405	12.15388	11.93341	3.986907	4.212493
gene-AT3G	0.586282	0.7	0.680531	0.737993	1.349385	1.520629	0.321907	0.702107
gene-AT3G	273.1191	274.8017	272.6751	285.7015	281.8326	280.2742	264.4927	276.5637
gene-AT1G	15.49865	13.93212	13.22019	8.280274	8.965605	7.359097	16.29528	20.80957
gene-AT1G	35.20118	36.39677	36.77285	28.89262	29.67328	28.04812	50.51569	50.95366
gene-AT3G	0.653831	0.685079	0.383577	0.50922	0.681759	0.523565	1.352357	0.962394
gene-AT1G	4.770529	4.116698	4.002638	0.296371	0.248641	0.587584	0.547525	0.653561
gene-AT3G	4.915466	4.307069	4.10941	3.647804	3.856386	4.01234	4.900413	5.439342
gene-AT3G	13.31452	15.46603	13.41247	9.319168	11.21662	9.981709	17.31441	18.71632
gene-AT1G	9.517507	9.979764	8.047155	21.10303	19.93828	20.56514	2.36644	1.649052
gene-AT1G	0.337554	0.299673	0.271537	0.149932	0.10215	0.302729	1.986689	2.608903
gene-AT3G	6.926817	6.265079	6.624234	10.84976	12.38527	11.99946	4.783012	6.026771
gene-AT4G	4.020441	4.014357	4.228012	5.56885	5.797782	4.559875	5.608263	5.982892
gene-AT4G	27.10456	25.09055	28.68146	20.63386	20.27435	22.79436	51.87371	51.0054
gene-AT3G	5.595556	4.507942	3.967527	4.921677	6.028779	5.589238	8.323299	6.834863
gene-AT3G	2.864123	2.511248	2.930003	5.630153	4.846035	5.104369	4.925905	4.54603
gene-AT4G	5.210903	4.35286	4.586645	13.30526	14.77242	12.47291	2.807161	3.19738
gene-AT1G	4.596862	4.143087	4.333953	3.782322	3.300802	3.141993	1.647219	2.264134
gene-AT3G	15.71738	13.80734	14.8854	21.03998	21.88064	19.73365	10.07802	11.24808
gene-AT5G	19.26463	16.29847	19.4246	21.19877	19.87637	23.27646	6.595676	5.706343
gene-AT3G	21.08915	20.07064	20.57795	6.51569	7.66879	6.203375	4.08281	4.2837
gene-AT5G	75.38601	76.70632	78.14122	231.3869	234.1075	233.9746	64.519	73.11174
gene-AT2G	15.04492	16.54237	17.04488	9.016011	7.921386	8.321826	20.09163	20.58121
gene-AT4G	7.24081	7.377237	7.890279	11.6733	11.72329	12.58177	7.970978	7.634131
gene-AT5G	21.60736	21.04418	24.27433	16.57359	16.70545	13.96341	9.02623	10.55754
gene-AT1G	2.721379	3.747205	4.331434	8.428893	8.926852	7.946909	2.941084	3.042644
gene-AT1G	274.3412	262.341	279.0445	348.6705	361.8966	358.1325	97.79538	105.2756
gene-AT4G	26.32643	22.90838	24.43943	13.80061	12.89947	15.53565	30.04301	30.56797
gene-AT2G	6.449972	6.218313	5.612043	8.703123	7.267942	8.352168	9.102679	6.690857
gene-AT4G	1.775771	1.444602	1.800544	1.169145	1.125165	0.983174	0.571893	0.730684
gene-AT1G	6.623477	5.562368	7.595249	8.015397	8.146865	7.720569	3.544382	3.434438
gene-AT3G	0.958206	0.879346	1.077303	1.522527	1.290669	1.562547	1.704811	2.612218
gene-AT1G	1.747234	2.425488	2.679266	1.639787	1.688586	1.709466	1.149377	0.634307
gene-AT5G	8.205219	9.752454	9.43368	9.254275	11.29072	8.471325	14.48853	12.1704
gene-AT2G	10.02642	9.851243	10.75401	3.34903	3.913428	4.012136	1.160857	1.416742
gene-AT5G	6.664008	6.781299	5.24742	8.56917	8.022616	7.517769	2.39999	2.130314
gene-AT2G	26.43447	26.83256	26.00836	16.98816	17.58565	15.86693	40.24382	40.49831
gene-AT2G	1.530908	1.812589	2.890183	2.040469	1.39373	2.077277	3.633312	3.573221
gene-AT2G	7.272293	5.997288	5.326545	43.29138	37.55204	40.4778	16.64959	16.33351

gene-AT5G	4.111255	2.954504	3.783531	9.751932	10.90473	9.192365	4.18788	4.707413
gene-AT2G	3.821957	3.816006	2.831068	4.405966	4.504775	4.731587	1.827979	1.790839
gene-AT1G	0.656571	0.313277	0.098132	1.176787	1.533384	1.179104	0.145379	0.112149
gene-AT1G	7.947239	7.291123	7.382376	5.995153	5.990466	5.441252	10.69018	10.87596
gene-AT5G	2.506271	2.623915	3.243051	4.240876	3.637712	3.137125	0.863226	1.243159
gene-AT5G	4.985634	4.527823	4.105548	2.772972	3.567978	3.949983	3.272009	4.83902
gene-AT4G	1.486268	1.531436	1.103418	2.871728	2.678555	3.246352	1.380785	1.090381
gene-AT2G	11.67852	10.80588	12.0368	7.924641	7.60794	8.445054	19.76477	19.62246
gene-AT5G	131.2909	122.7459	125.6241	104.3761	105.1453	108.2703	36.68753	41.22278
gene-AT2G	1.688521	1.852054	1.703046	5.135764	5.80692	4.204393	2.550642	2.937158
gene-AT1G	0.90178	0.784519	1.015948	0.247576	0.489624	0.283079	1.093642	0.71062
gene-AT4G	4.711556	4.823781	4.157296	8.327534	8.91501	8.688823	1.706394	1.628655
gene-AT1G	80.41596	82.46906	76.9763	90.03254	90.88495	90.96706	43.48676	47.34308
gene-AT3G	30.09082	31.29839	27.75622	24.08958	21.46692	21.13016	42.12007	44.94173
gene-AT2G	129.9014	124.2558	126.0899	95.5052	90.34658	90.60621	159.0052	172.2647
gene-AT5G	8.100354	8.168277	7.784813	2.678719	2.123026	3.107584	6.21434	6.652053
gene-AT1G	5.050383	6.635559	6.152368	11.81537	9.239009	9.054734	7.572083	6.302807
Arabidopsi	1.706266	1.220929	1.726837	0.308994	0.758135	0.493573	0.975932	1.278683
gene-AT4G	2.038952	1.839429	1.675234	8.29652	7.091501	8.08987	6.280447	5.337606
gene-AT4G	29.72001	28.74197	31.73477	46.4967	41.99351	41.53849	15.16341	16.01644
gene-AT1G	301.9096	294.0156	300.8643	117.2599	116.7031	113.9359	188.3261	204.4175
gene-AT1G	1.428689	1.567695	1.486293	1.307497	0.895017	1.491185	0.036946	0.373985
gene-AT3G	243.1246	245.9293	256.894	366.3516	353.278	341.747	148.1315	141.4438
gene-AT2G	7.12552	5.748488	6.028914	2.500514	2.85849	2.567902	5.041936	3.715193
gene-AT5G	20.58459	21.08986	19.23966	6.554556	7.584336	6.182626	2.583311	3.464784
gene-AT4G	0.830533	0.7732	0.332428	0.961347	1.136044	0.890067	0.562913	0.417128
gene-AT4G	1.09889	1.861636	1.536528	1.693476	1.725433	0.982968	0.188815	0.598862
gene-AT5G	1.323879	1.425464	1.214857	0.944767	1.245347	0.557951	0.361265	0.323512
gene-AT1G	14.27836	13.74831	14.15445	6.25155	5.157412	6.247734	7.073838	7.087204
gene-AT4G	20.67778	19.92632	18.065	28.48	30.21361	28.15609	27.79665	29.54721
gene-AT1G	7.347095	5.410743	6.405526	7.032716	7.736111	7.402546	1.390938	2.432945
gene-AT1G	19.81127	16.28637	19.3653	9.526355	9.686417	8.185922	9.958796	10.92514
gene-AT3G	6.994638	7.543727	7.582426	6.405735	6.039765	6.539491	9.898885	8.654721
gene-AT3G	12.48233	12.39508	13.67766	13.62081	14.44334	13.24848	8.234157	7.846021
gene-AT5G	0.735371	0.619973	0.764381	5.547388	6.07832	6.163053	4.913582	5.784618
gene-AT5G	1.334571	1.335087	1.861303	0.989307	1.330585	1.3596	1.585552	1.59773
gene-AT3G	1.766431	1.268895	1.638605	1.029281	0.896416	1.090925	1.209835	0.909934
gene-AT3G	5.788648	3.404533	4.093509	4.743989	4.275439	6.178803	1.435317	0.898145
gene-AT5G	4.012208	4.639828	4.89362	32.01414	30.13278	31.72413	1.262784	0.966257
gene-AT1G	9.163077	10.94012	9.718935	11.60414	11.14582	10.71734	3.606797	3.301518
gene-AT1G	64.11925	60.98764	64.7351	35.60458	36.02146	38.14923	98.84837	104.8362
gene-AT4G	3.790238	3.799263	5.244225	10.00629	8.755434	9.348111	2.406475	3.797387
gene-AT1G	3.322431	3.568881	3.433933	7.357184	7.922168	6.722911	2.630208	2.549452
gene-AT1G	605.0459	593.8958	585.003	467.5054	468.765	467.4864	790.7387	782.5436
gene-AT2G	7.501184	6.745888	6.722149	8.482588	8.448001	8.002735	8.805877	8.736328
gene-AT3G	14.58542	14.24307	14.97774	6.895802	6.56835	6.904914	24.81817	24.59486
gene-AT5G	35.36418	37.03854	34.9169	23.75084	21.65693	23.52866	11.20703	11.15603
gene-AT4G	14.23089	13.9741	13.4763	9.666449	8.892467	9.381936	25.02606	22.60061
gene-AT2G	10.37291	9.655176	10.22705	10.58569	9.126311	10.57737	9.147827	10.39302
gene-AT1G	1.92328	1.282411	1.662466	2.096732	1.742279	1.553492	1.206152	1.007633
gene-AT1G	0.41467	0.254321	0.404165	0.744033	0.438088	0.42118	0.929988	1.082022

gene-AT2G	25.55936	28.50635	25.3521	3.854455	4.721541	5.333068	66.82915	71.43588
gene-AT4G	5.165546	4.609551	5.282278	4.945888	5.316443	4.599113	6.289697	7.939422
gene-AT4G	5.57329	5.767196	5.572928	0.872777	0.956531	0.943872	1.235031	1.277295
gene-AT5G	50.18494	50.40039	49.52951	28.48837	30.39868	30.1962	84.39086	84.92211
gene-AT3G	10.78094	11.50838	11.68178	5.959896	5.712022	3.768532	19.47327	19.83115
gene-AT5G	2.136646	1.921752	2.805819	1.479902	1.504895	1.662201	0.909582	0.87221
gene-AT1G	12.1928	11.81715	11.6266	7.718709	5.883666	5.780581	6.456059	6.68073
gene-AT2G	2.361503	2.45123	2.266767	1.334725	2.247863	1.537068	1.029207	1.196878
gene-AT1G	0.76784	0.558829	0.733712	0.885061	0.555638	0.468618	0.798381	0.986709
gene-AT5G	2.441871	2.176072	2.711841	1.569384	2.591623	2.138037	2.139198	2.682911
gene-AT5G	3.204219	3.489168	3.266349	4.659276	4.918082	4.524133	2.407853	2.796176
gene-AT5G	67.14408	64.71587	66.85826	94.87091	93.79716	88.367	106.0204	110.7761
gene-AT1G	6.488598	7.524375	7.293116	7.032503	5.576191	6.404087	9.798583	9.16015
gene-AT3G	6.684103	5.815374	6.215429	2.830623	1.868595	2.53651	3.435129	2.860064
gene-AT4G	21.6684	18.86393	17.75418	17.04297	15.38786	17.76675	34.37049	32.4734
gene-AT2G	3.019795	3.240558	2.987281	5.881766	4.663051	6.352194	0.173552	0.206013
gene-AT2G	19.78824	20.11735	22.52092	32.49936	30.49247	31.38606	9.613867	9.504721
gene-AT5G	2.668124	3.07152	3.734564	1.368077	1.604594	1.748641	0.915117	1.055172
gene-AT3G	16.10826	17.09067	16.07815	17.96616	18.1736	18.11626	4.603824	4.914694
gene-AT2G	0.07491	0.148156	0	2.207417	1.674347	1.913817	0.087124	0.046692
gene-AT2G	0.586382	0.354684	0.31608	0.181304	0.158016	0.272677	0.595818	0.519717
gene-AT5G	6.044426	6.938061	7.026344	1.374613	1.261574	1.64551	0.222078	0.207719
gene-AT5G	52.26065	50.02505	50.54364	35.66734	40.06548	37.42967	56.93838	60.84201
gene-AT3G	9.81063	9.511198	10.81143	8.473187	9.102201	9.532265	5.272983	5.480029
gene-AT5G	10.28853	10.32027	10.56096	9.291528	9.449765	10.10611	16.6436	19.32667
gene-AT4G	1.880087	1.549092	1.345731	1.087204	1.493373	1.159737	2.117082	1.444859
gene-AT1G	11.35194	10.813	11.22003	11.36556	10.6973	11.75351	2.78263	3.330454
gene-AT1G	5.377975	6.091792	6.147588	9.623849	9.554639	9.272693	2.550246	2.759124
gene-AT3G	4.001961	4.950081	4.105379	3.669424	4.657731	4.495395	3.210518	3.173262
gene-AT1G	41.50618	43.30487	45.79515	38.88884	40.00202	40.48153	29.65086	27.00978
gene-AT3G	10.37898	11.76792	10.93151	6.725681	7.954228	7.462401	9.197845	10.42518
gene-AT1G	95.90776	94.74728	95.44864	35.22723	36.0216	33.26452	48.43425	51.73087
gene-AT3G	7.346268	6.721188	5.53308	5.86324	5.974234	5.798491	3.949057	3.662708
gene-AT1G	1.063675	1.301752	1.412395	1.319858	1.189852	1.22852	2.432824	2.377781
gene-AT3G	8.044837	8.449944	8.539262	7.82507	6.793185	7.69716	11.51668	11.97302
gene-AT5G	218.4319	217.9313	212.1474	123.0305	118.7977	121.4468	314.6064	342.6888
gene-AT1G	0.113145	0	0.202032	1.288919	1.408683	1.510225	0.07546	0.027743
gene-AT4G	5.618589	4.955875	4.020901	2.26561	1.968321	3.171324	4.062067	7.214604
gene-AT2G	0.950283	0.313151	0.781578	0.222508	0.203593	0.142997	0.99716	1.196276
gene-AT4G	59.36935	55.732	61.90674	112.2681	121.0852	112.9186	29.10873	26.22697
gene-AT3G	0.871436	0.729898	0.717932	0.370746	0.081951	0.35102	0	0
gene-AT1G	3.907773	3.762375	2.985666	2.970388	3.482204	4.095673	8.582125	6.567982
gene-AT5G	3.96529	3.838723	2.571498	1.798925	1.715525	1.263847	2.501099	2.651833
gene-AT4G	2.004098	1.516965	1.218508	0.560273	1.14062	0.648454	0.252567	0.127955
gene-AT5G	18.76901	18.18978	17.21772	1.688827	1.547441	2.148758	10.3682	13.69002
gene-AT1G	2.261553	2.593369	1.920074	3.360221	3.937122	3.517411	3.444103	6.217551
gene-AT1G	1.447914	0.854126	1.50941	0.957229	1.446517	1.618927	1.951779	1.235015
gene-AT1G	17.12755	15.20639	16.27968	8.963504	9.488642	9.555285	1.902614	2.526211
gene-AT4G	6.530999	5.889693	6.131502	4.12012	4.223591	3.911705	11.87753	11.81177
gene-AT5G	4.331381	4.753031	4.541332	7.424725	6.518823	7.674784	3.170878	2.656594
gene-AT5G	22.76077	23.22463	23.2333	75.52633	79.55051	77.05307	7.76053	6.883484

gene-AT5G	20.01206	18.97217	18.89318	18.60146	18.90214	17.93673	26.1818	29.47608
gene-AT1G	2.127576	1.521193	1.323157	1.259097	1.400652	1.309998	4.912102	3.41799
gene-AT1G	2.554589	2.606004	1.741196	3.043696	1.818012	2.55484	4.027357	3.80158
gene-AT5G	69.47559	67.71605	67.1595	69.40156	68.29732	71.58147	61.29451	65.21183
Arabidopsi	1.030827	1.195799	1.114817	0.662328	0.673847	0.720061	1.094909	1.220557
gene-AT5G	1163.953	1162.062	1202.827	561.1234	565.9576	566.4548	1231.058	1285.019
gene-AT4G	11.39824	9.547654	9.993647	13.44526	14.6185	13.3771	3.644479	4.183709
gene-AT3G	86.77506	82.63319	88.35426	109.0597	105.5686	110.9991	25.49233	21.35698
gene-AT3G	4.686247	5.201365	4.748114	3.735326	3.215501	3.960587	3.108408	4.212912
gene-AT4G	62.50215	55.12222	62.25705	50.2964	49.10861	52.46128	91.07995	94.01419
gene-AT1G	33.4495	32.91554	31.60381	24.25512	23.81697	24.20488	71.12315	79.34251
gene-AT5G	19.21956	18.32968	17.4297	20.21877	19.25793	17.4818	1.401217	0.888961
gene-AT3G	1.466233	1.295149	1.22735	1.998966	0.881915	1.524763	0.250879	0.694841
gene-AT4G	7.013253	6.139287	6.423818	3.620858	3.329895	3.076807	1.521274	1.978443
gene-AT2G	12.09943	16.28198	13.76088	12.3512	11.28632	11.68998	5.916035	7.048659
gene-AT2G	0.407952	0.501269	0.960422	0.082189	0.138259	0.099851	0.839707	0.635557
gene-AT4G	64.75839	59.58211	59.2671	61.29443	64.43322	57.84795	110.7134	115.5053
gene-AT5G	21.94479	23.53145	22.53363	17.25795	17.71239	18.22448	37.30581	37.68414
gene-AT2G	621.4229	618.9009	643.8524	397.0599	383.9041	397.2247	1075.676	966.7455
gene-AT4G	6.629167	5.789776	5.862176	3.768385	4.643816	4.323793	6.587114	6.481816
gene-AT1G	63.36472	60.88556	55.96655	17.60609	20.37495	18.90089	8.541958	9.478915
gene-AT5G	7.633977	8.241323	8.769559	5.178312	5.752218	5.328723	16.26518	16.5333
gene-AT1G	2.468005	1.997567	1.553969	5.737867	4.574163	4.032383	6.351421	5.56857
gene-AT1G	4.0797	5.035069	4.188529	2.841051	2.856652	2.199514	19.61158	20.31628
gene-AT1G	28.29881	27.59934	30.36821	19.85085	21.63157	20.10485	40.71583	45.44848
Arabidopsi	1.501742	1.636594	1.503204	0.665826	0.496013	0.412486	1.19173	1.594319
Arabidopsi	1.364597	1.659501	1.605564	1.850877	1.916555	2.424074	2.564176	3.294019
gene-AT5G	25.0737	22.58895	24.2638	22.58698	22.74544	22.54778	22.04848	21.84441
gene-AT3G	2.254711	2.945115	2.336796	1.485749	1.270504	3.228838	0.221627	0.308558
gene-AT1G	1.537197	1.409858	1.809067	6.413404	7.291197	7.225782	0.108741	0
gene-AT1G	2.395046	1.676466	1.924781	3.149814	2.220869	3.175431	1.487034	1.54177
gene-AT1G	1.9032	1.677638	1.86918	0.618887	0.555833	0.706243	0.970134	0.885632
gene-AT2G	1.258154	0.700799	0.898839	3.193783	1.956499	2.476136	0.418676	0.125523
gene-AT1G	0.402369	0.765371	0.396951	3.425526	3.220368	3.000861	0.488216	0.469833
gene-AT4G	11.44169	8.908474	9.105315	5.183862	4.393407	5.039575	3.199659	2.437203
gene-AT3G	10.58534	12.11809	11.4351	10.91391	11.78192	12.427	5.366872	5.322903
gene-AT1G	2.290327	2.378547	2.336077	5.16792	4.487143	4.662173	2.403544	1.956754
gene-AT5G	1.858526	2.363273	2.367259	1.998786	2.468987	2.008299	0.320242	0.301005
gene-AT3G	2.387348	2.291399	2.536151	2.463154	2.710901	2.67306	0.371827	0.431531
gene-AT5G	353.6623	350.6522	352.7937	220.5183	223.1874	218.8523	332.473	363.7896
gene-AT2G	9.396681	10.97186	9.615334	9.998064	10.57081	9.43701	2.481164	2.758097
gene-AT5G	0.684533	0.660539	0.89872	0.484798	0.383334	0.666097	0.740652	0.725608
gene-AT1G	2.26121	2.836435	1.498173	2.72384	3.087669	3.047217	1.174709	2.846978
gene-AT5G	4.558045	2.463149	4.638668	3.18938	3.928123	4.187844	3.219168	4.984661
gene-AT2G	9.412969	8.098927	9.192339	14.02341	17.04517	15.11719	1.166214	1.425284
gene-AT4G	1.214597	1.181853	1.59782	4.004954	2.508683	2.960335	2.901564	2.368314
gene-AT1G	1.158474	1.649696	1.009917	0.722252	0.736862	0.532841	0.207122	0.404022
gene-AT1G	6.815312	7.165118	7.484942	6.074602	5.574846	5.638216	6.212286	6.787471
gene-AT3G	0.184784	0.216421	0.190809	0.296134	0.446113	0.130366	0	0.024687
gene-AT3G	0.650568	0.72831	0.698067	0.920673	2.3775	0.831085	0.414395	0.634968
gene-AT1G	22.70112	22.83389	24.28488	72.74952	69.58263	72.36253	4.456956	4.496103

gene-AT5G	13.33818	11.86379	13.17878	10.17445	10.07994	9.16007	3.631623	3.493627
gene-AT5G	36.70735	41.01547	35.90822	37.97755	37.63625	38.59717	64.65186	67.52924
gene-AT2G	0.515788	0.784288	0.558919	1.36309	1.050698	0.768547	1.573091	1.436647
gene-AT4G	2.530464	2.059849	2.619434	0.615018	1.343653	1.395241	5.891229	5.696611
gene-AT1G	1.598482	1.269125	1.883018	2.378009	1.481539	1.475847	2.697992	2.722465
gene-AT4G	7.73762	8.690938	9.428525	6.741107	6.07367	7.995612	14.62955	14.28193
gene-AT5G	0.310303	0.087517	0.147929	0.893325	0.910228	0.932822	0.420597	0.201894
gene-AT3G	35.58183	34.08186	37.01761	36.94634	33.94426	37.30507	16.65246	16.51877
gene-AT1G	4.462266	4.804673	4.590656	5.626218	5.40828	5.049474	0.97767	1.112942
gene-AT3G	4.152326	2.711799	2.364042	3.563767	2.198412	1.74295	0.352374	0.530724
gene-AT5G	7.730741	9.123585	8.250896	4.567884	4.929078	4.80016	10.73574	13.11414
gene-AT5G	22.85506	23.80917	23.02925	23.03689	23.29662	24.62459	23.09182	23.29232
gene-AT1G	1.429505	1.707726	0.977022	0.987218	0.703595	1.249037	2.740264	2.045937
gene-AT5G	45.40526	42.60596	44.7416	68.76018	71.80491	69.42062	27.31749	29.93917
gene-AT1G	5.068453	4.96931	5.409905	3.488967	3.842716	3.303484	7.625842	7.894517
gene-AT4G	27.27164	24.52185	24.83159	27.69869	30.61114	28.46744	10.66663	10.6424
gene-AT1G	3.34916	2.460222	2.265329	5.667024	4.937519	4.85551	2.680962	3.05725
gene-AT4G	1.56531	1.234566	1.496277	1.128847	1.440385	1.408413	0.253546	0.404471
gene-AT4G	0.494719	0.820401	0.631048	3.441388	3.338863	3.737228	0.279546	0.63503
gene-AT3G	103.6858	100.5944	100.2271	93.33094	91.21403	91.30379	144.4471	153.6277
gene-AT1G	1.646288	1.947967	1.262114	2.183105	2.236779	2.107664	2.903332	3.181303
gene-AT1G	2.490131	2.895288	2.52169	1.747034	2.03069	2.266082	0.68236	0.908219
gene-AT1G	12.58368	10.86996	11.29796	8.01493	8.313637	9.13833	16.11706	17.97624
gene-AT1G	29.73578	25.91915	25.60113	30.28742	27.04861	27.92692	12.52349	12.96095
gene-AT3G	3.958909	3.743754	4.039761	4.965884	5.531852	3.98941	5.060392	4.60534
gene-AT2G	2.344464	2.722852	2.193501	2.273239	3.049083	2.65217	2.93353	3.084424
gene-AT1G	17.59534	18.33662	18.21168	7.070632	3.601022	7.335569	8.212379	7.087432
gene-AT5G	3.591032	3.309451	3.512167	7.972134	8.195691	6.88589	2.95807	3.435607
gene-AT1G	5.219332	5.660947	5.100101	6.487004	7.14676	6.948537	3.237555	2.973324
gene-AT5G	1.584498	1.588531	0.824494	0.713144	0.915995	1.207675	0.108154	0
gene-AT3G	23.40797	24.64532	25.6363	14.20031	13.69139	14.21358	4.108802	5.360156
gene-AT5G	38.13736	38.71587	39.78698	29.40902	27.16051	28.86605	42.6093	39.79129
gene-AT1G	4.959501	3.715648	3.13785	17.21206	17.50353	17.45232	6.946973	5.622589
gene-AT4G	15.17348	15.28335	18.49991	15.0306	13.53088	14.40114	7.694753	7.887726
gene-AT4G	0.714504	0.840114	0.757944	0.660636	0.734971	0.526969	1.813084	1.389203
gene-AT5G	0.311677	0.147616	0.091035	0.112453	0.226063	0.295068	0.634846	0.675515
gene-AT3G	24.64877	21.92976	21.76418	23.61882	22.51549	23.84474	21.02047	20.33383
gene-AT3G	13.98253	17.7053	15.61898	11.42684	12.94556	12.09237	32.72998	33.75938
gene-AT1G	0.511626	0.871351	0.653447	2.271533	2.968959	2.750062	1.122512	0.747953
gene-AT2G	2.604141	2.859273	3.16129	4.825645	4.638783	4.699291	2.221688	1.623674
gene-AT5G	2.305082	2.427708	2.741755	2.367956	2.590913	2.534798	0.823626	0.615019
gene-AT2G	3.540121	2.483101	2.778237	3.320178	3.781551	4.042528	1.364348	1.474778
gene-AT3G	5.410194	5.837912	6.949772	6.917527	6.815481	7.543026	2.689261	1.961939
gene-AT3G	3.983327	3.174019	4.167457	4.072729	4.650411	4.333889	4.36313	4.779661
gene-AT2G	1.716519	2.451644	1.506193	3.234297	3.135135	2.813665	4.456086	4.460416
gene-AT4G	10.43391	9.754181	10.73763	18.77638	19.28036	19.46427	10.03693	8.840803
gene-AT1G	59.11336	57.88469	56.12385	37.01817	47.29779	41.45476	93.49088	96.20957
gene-AT1G	0.443747	0.591759	0.424911	0.283262	0.357485	0.334735	0.624223	0.948056
gene-AT5G	0	0.040963	0	0.082947	0.090657	0.235534	0.180403	0
gene-AT5G	0.054699	0.134495	0.258772	1.052753	0.695633	0.919614	0.183098	0
gene-AT5G	90.06739	92.62566	80.6228	63.35384	64.92569	57.98974	171.2875	168.6432

gene-AT1G	16.54563	15.42675	16.55798	25.36755	25.00553	25.26466	12.15318	13.28928
gene-AT1G	25.43999	24.50114	25.62865	14.02409	15.07641	15.65738	36.47084	36.03372
gene-AT5G	0.651972	0.741258	0.616149	67.03418	66.07036	63.25911	3.143955	3.645301
gene-AT5G	4.018582	4.102349	4.06958	5.163405	5.453514	4.837211	9.085129	9.122882
gene-AT1G	36.55196	38.98907	37.04829	37.48334	36.35051	37.25815	55.31985	61.26419
gene-AT3G	7.710315	7.86645	9.509842	7.20431	7.635025	7.789791	15.70705	16.08795
gene-AT2G	4.154448	3.725248	3.784241	5.193475	4.83017	5.458763	4.68625	4.200831
gene-AT3G	5.894521	4.969616	6.793755	34.75082	35.70648	35.80043	3.06486	2.52522
gene-AT4G	26.44204	22.60182	24.62829	10.40979	8.392623	8.218688	76.55994	75.31747
gene-AT2G	34.64952	34.49178	33.78038	67.6711	73.65375	66.22546	25.13216	28.23537
gene-AT1G	0.523892	0.744011	0.861869	1.306065	0.759642	0.713133	1.228746	1.806892
gene-AT4G	1.509854	1.142892	1.525535	4.465893	3.478227	4.78459	0.326685	1.081432
gene-AT5G	3.519499	3.117501	2.900235	8.911445	8.800391	9.00791	1.002595	1.765335
gene-AT1G	18.21306	15.26393	17.93237	12.68951	12.53564	10.62094	6.462017	7.233518
gene-AT1G	0.792517	1.319635	0.907806	5.359025	4.27472	2.99679	0.680801	0.307555
gene-AT5G	14.57215	14.18356	14.83499	12.67317	13.18866	13.32473	6.594294	6.254545
gene-AT4G	14.03162	14.72234	15.24256	5.048199	4.293974	5.066946	8.277933	9.624402
gene-AT2G	6.44054	6.143281	6.347292	7.094582	6.963237	7.05323	0.225726	0.111686
gene-AT1G	19.1987	18.72606	18.69935	11.78678	11.00735	11.47873	28.15591	31.83444
gene-AT1G	1.54131	2.106288	2.173517	3.566365	4.261358	4.008389	1.646183	2.390749
gene-AT3G	1.815428	2.347846	2.435896	2.512777	1.876714	2.128273	0.968739	1.070349
Arabidopsi	0.675661	0.786491	0.663044	0.052022	0.211853	0.284573	1.209919	1.203453
gene-AT1G	2.06583	1.755904	0.97477	3.587215	5.181289	4.339341	3.493257	2.619552
gene-AT5G	15.22234	15.09456	12.64981	11.55763	11.06322	11.15177	20.21433	22.12439
gene-AT2G	0.974232	1.110379	0.662503	1.825919	1.241739	1.740674	0.783506	0.589196
gene-AT1G	18.79576	16.82046	19.50139	23.38242	24.04437	23.81234	6.504828	6.513826
gene-AT3G	1.980828	2.687551	2.757725	4.359648	3.578666	4.673093	2.163079	1.850774
gene-AT1G	1.804146	1.875472	1.131085	2.192057	1.795794	1.335485	0.419796	0.764174
gene-AT1G	34.90793	30.52449	32.17979	22.5732	19.63553	21.48644	43.78519	38.7465
gene-AT5G	25.14733	25.9548	26.43109	25.98117	24.15847	24.23606	36.84039	36.72599
gene-AT3G	3.369528	3.980519	4.264958	11.57598	11.87079	10.77324	6.758032	4.932212
gene-AT4G	6.679874	5.079475	6.095673	13.99347	14.49929	14.84856	9.722355	11.22673
gene-AT5G	0.44045	0.264353	0.226565	0.513312	0.302746	0.431803	1.329168	1.979242
gene-AT5G	3.40866	2.310276	2.966049	4.808589	3.869245	4.118597	1.150637	0.816077
gene-AT4G	2.050195	2.281013	1.854654	2.81432	2.894026	2.871002	0.738807	0.518754
gene-AT1G	37.13804	34.63618	40.16379	24.99211	26.67402	25.28943	15.10031	17.50262
gene-AT4G	16.73429	17.52583	18.56192	13.92866	14.3297	13.92083	14.93594	15.23439
gene-AT1G	9.326792	6.596582	9.007887	3.032823	2.997901	3.280299	15.49001	15.76721
gene-AT4G	19.62691	17.67757	14.95776	9.882751	12.62193	9.752242	16.63939	16.51954
gene-AT1G	9.254533	10.66258	9.002719	32.56339	33.51222	29.67636	13.79619	13.81793
gene-AT1G	4.404942	2.927802	4.270479	4.488125	5.767765	4.433958	2.444776	2.027563
gene-AT1G	3.307486	3.308342	4.333848	6.926091	6.217176	6.933436	1.71386	2.29666
gene-AT1G	3.015715	1.557847	3.062376	5.069119	6.227686	4.931542	1.282175	1.341983
gene-AT1G	0.710961	1.219535	1.225661	1.022908	1.166341	1.311741	0.435284	0.41414
gene-AT4G	1.449559	0.736664	0.88616	1.258763	1.61778	1.016668	1.236071	0.777469
gene-AT4G	3.780037	3.32426	2.94902	2.387367	2.301244	1.889704	1.210254	1.257577
gene-AT5G	28.18464	29.17548	29.6857	17.87243	18.2565	16.38446	24.56468	24.59333
gene-AT1G	0.60387	0.903517	0.63761	1.560593	1.699059	0.926088	0.177488	0.504737
gene-AT1G	32.12135	32.95488	34.4633	21.2495	20.25912	18.22037	56.31147	54.43455
gene-AT5G	22.2442	22.92327	24.27907	23.11548	22.8282	21.21664	23.98352	23.56121
gene-AT5G	50.05848	46.48725	48.72457	96.90701	98.22019	98.76274	19.29884	20.52506

gene-AT4G	3.038849	1.966167	1.716203	2.139859	2.701438	1.437668	6.02917	5.367059
gene-AT4G	5.633575	7.305243	7.184762	6.645056	6.077711	6.250508	12.16685	13.12898
gene-AT3G	2.774791	2.500089	3.18602	3.526434	4.065427	4.048907	0.327234	1.156849
gene-AT2G	10.47053	10.89095	10.49529	6.363974	6.285833	6.236262	13.93946	13.87054
gene-AT5G	4.038264	3.518604	3.699908	4.321572	4.05163	4.17996	1.090215	0.976709
gene-AT5G	10.08224	9.40675	6.262818	7.847449	7.03651	8.714299	3.360036	3.478489
gene-AT2G	2.930333	2.684811	2.628784	3.36871	2.527542	2.417098	8.305124	8.851368
gene-AT3G	7.405363	7.060421	8.115055	4.082589	5.352893	4.563995	10.45445	11.06809
gene-AT5G	9.589468	11.11013	13.4212	24.94934	23.4677	23.50912	16.08468	16.92324
Arabidopsi	2.999563	3.430081	3.186606	6.785455	8.19215	8.190762	3.789236	3.726923
gene-AT5G	11.79382	14.70394	14.98335	0.832724	0.830741	1.19147	8.92301	7.14485
gene-AT2G	150.8551	148.9552	147.1667	104.6734	98.2478	101.372	178.9627	193.1914
gene-AT5G	1.278334	2.440737	1.689527	1.444959	1.694112	1.6491	2.115822	2.294848
gene-AT1G	0.545466	0.599061	0.32067	0.599973	0.568327	0.501729	1.352239	0.805613
gene-AT4G	0.854986	0.325085	0.316876	1.098329	0.691853	0.850888	0.308592	0.688165
gene-AT1G	14.8833	15.96602	14.93574	7.329456	9.056257	8.547452	6.511905	5.928787
gene-AT3G	10.88836	10.88296	11.4229	10.04662	11.01852	9.909107	21.66088	22.29156
gene-AT5G	8.131302	9.91886	7.713802	4.936393	5.725257	5.133086	7.888131	8.661834
gene-AT2G	1.80915	1.459709	1.548685	0.632295	0.979627	1.04397	1.799304	1.581585
gene-AT4G	27.72756	29.30251	28.25953	22.77786	27.4801	25.9248	43.87765	45.21333
gene-AT4G	2.33689	1.433354	2.959951	2.508267	2.190513	2.040824	3.558406	3.186497
gene-AT1G	3.780637	2.879474	5.007926	1.933463	2.034324	2.650503	2.209729	1.621217
gene-AT3G	15.55015	17.42326	16.41351	11.36206	11.68479	11.14561	20.65566	20.2229
gene-AT3G	1.607711	0.82756	0.592182	1.674545	0.74964	1.016682	0.206903	0.443398
gene-AT3G	7.56927	6.630561	6.487104	14.71695	15.57643	16.46317	6.688034	6.550643
gene-AT5G	8.204946	8.746295	7.269802	3.899673	3.731232	3.382994	3.134888	4.028121
gene-AT5G	12.511	12.57382	12.29915	15.07937	13.53766	15.23986	5.642736	6.157376
gene-AT2G	76.85371	70.15437	78.51617	53.225	52.90864	49.02225	115.7595	119.4426
gene-AT5G	3.983398	4.442917	3.908224	3.450218	3.236539	3.571699	2.368596	2.061363
gene-AT1G	6.571808	7.198092	6.433334	5.781288	6.138156	6.013798	8.141623	8.90723
gene-AT2G	27.48779	26.31678	25.51085	7.819787	4.366379	5.391115	6.629811	7.907021
gene-AT4G	2.307672	2.474619	2.460909	5.075249	5.344066	4.703425	0.823166	0.896945
gene-AT5G	22.5403	20.19437	22.9506	51.17802	51.86022	51.79376	24.61784	23.75776
gene-AT1G	14.13145	13.43094	13.48868	7.779441	7.121662	7.348228	3.05882	3.045726
gene-AT4G	343.7141	348.0747	358.6909	270.2152	268.9839	277.5519	583.5247	513.0843
gene-AT1G	6.018112	3.753626	4.464434	4.377035	3.867197	4.327252	8.461082	9.473213
gene-AT1G	19.44806	20.74608	20.34373	18.71753	19.78198	19.25004	32.25306	35.38247
gene-AT2G	6.430668	4.333295	4.510701	2.555332	2.357644	1.762944	13.97704	14.41177
gene-AT5G	25.39393	29.81028	28.10225	22.18886	19.71639	20.11909	47.8901	46.92825
gene-AT1G	0.906938	0.884962	0.675472	1.767072	1.588785	1.015218	0.442364	0.495333
gene-AT3G	76.30372	75.52908	79.2787	90.30414	88.93838	89.83826	46.7913	50.39552
gene-AT3G	192.2008	177.6488	186.7727	84.47335	81.65047	88.17753	95.25136	93.85519
gene-AT3G	3.158882	3.552734	4.591614	3.339246	3.710159	3.014601	10.18529	10.00217
gene-AT5G	102.5617	100.2938	99.15415	82.90814	84.31349	84.60121	107.2328	108.2796
gene-AT3G	173.7365	179.2123	179.6739	132.3912	138.6277	140.8535	283.4871	284.8414
gene-AT2G	5.525581	4.184304	4.473307	6.709659	6.539281	5.441014	2.612689	2.447686
gene-AT4G	152.7514	153.1068	155.351	141.6104	143.9977	141.2965	255.3977	270.8145
gene-AT4G	2.581496	2.352485	2.111217	2.403608	2.472875	2.450153	0.983281	1.068849
gene-AT3G	2.822135	2.252529	2.840591	2.594618	2.651286	2.647982	4.101447	5.013094
gene-AT3G	5.382801	4.669571	6.705769	6.713261	8.035786	7.970553	1.823111	1.914242
gene-AT1G	14.12083	14.05186	14.36459	17.73019	16.11409	16.55065	4.27288	3.757478

gene-AT3G	6.116354	5.618865	5.697416	28.15909	26.4045	27.37724	2.769813	2.071102
gene-AT3G	273.995	274.3981	267.9045	159.6888	160.7635	164.4251	362.8459	371.6964
gene-AT1G	5.137359	4.747795	3.968493	3.052778	2.569008	2.589406	5.371553	6.912064
gene-AT1G	0.564731	1.068581	0.450956	1.283608	1.324624	0.919694	0.512571	0.568071
gene-AT5G	3.614238	3.95054	3.601183	5.933803	6.318559	6.045172	6.663532	8.19971
gene-AT5G	0.468689	0.397578	0.503933	1.120033	1.458759	1.237616	0.213082	0.300598
gene-AT2G	2.948884	2.153418	2.437165	3.107285	2.812451	4.011415	1.059686	1.072869
gene-AT5G	4.831614	5.322615	5.183956	2.920261	3.837323	3.094483	5.309615	5.938217
gene-AT5G	0.972159	1.224806	0.926102	1.751055	1.602946	1.30617	3.055157	2.722204
gene-AT1G	32.93467	33.63375	33.29899	23.34377	22.66085	19.75055	36.47952	35.38781
gene-AT4G	19.86428	19.23003	18.26096	21.27956	22.76835	23.29837	10.18938	8.985817
gene-AT3G	12.86015	12.87632	12.40141	7.446572	5.633433	6.589492	11.50821	10.48041
gene-AT4G	7.657629	7.458949	7.427099	8.8837	8.315801	9.660897	9.4061	8.380422
gene-AT1G	21.8708	16.62091	18.7828	23.15536	26.42225	26.1778	15.87561	15.77296
gene-AT1G	3.389096	3.841383	4.860272	8.514971	7.430668	8.299952	1.405279	0.555678
gene-AT5G	6.104799	5.097882	4.476111	225.8033	205.6107	210.2788	2.487082	3.405067
gene-AT1G	4.339644	3.934083	3.700522	4.389095	3.489152	4.261447	1.333355	1.707646
gene-AT5G	5.103274	4.705923	4.925973	4.111621	4.283192	4.390983	1.329889	1.967873
gene-AT4G	24.0488	21.48975	23.63387	48.31282	44.75909	43.34223	46.13937	50.84623
gene-AT1G	35.39253	37.46368	33.72772	7.576748	7.091236	7.793203	2.168289	2.072896
gene-AT3G	0.418823	0.231289	0.374462	0.671013	0.248561	0.285073	0.196454	0.034279
gene-AT2G	16.47664	18.59436	16.39376	19.09531	18.4221	19.67067	32.8366	35.027
gene-AT4G	7.091559	6.853682	6.899475	8.076228	8.462915	8.751297	9.579674	10.4092
gene-AT5G	2.730766	2.701412	2.112434	2.110183	1.239482	2.137351	5.421884	3.612225
gene-AT4G	0.395504	0.599308	0.783924	1.046705	1.813679	0.608203	0.117406	0.264622
gene-AT5G	2.962251	2.206277	1.460066	1.478813	1.215442	1.636317	1.85201	2.029114
gene-AT1G	0.389708	0.668372	0.407722	0.932507	1.162112	0.930517	0.258062	0.400864
gene-AT3G	48.20872	49.72952	48.82724	21.69983	23.04969	23.12373	56.13493	53.94428
gene-AT2G	35.88563	34.68527	35.13746	21.69941	20.01798	22.01081	53.17279	56.13117
gene-AT5G	13.27553	12.83475	13.4178	13.06423	11.53945	11.07266	10.12915	11.24621
gene-AT1G	5.587983	5.506718	5.130784	5.025033	5.866055	6.407341	6.691659	7.057548
gene-AT1G	0.97831	1.516604	0.595993	1.529662	0.655214	1.192867	0.560357	0.522499
gene-AT5G	37.4416	30.81376	34.362	27.51384	27.80607	29.31636	51.40601	50.05302
gene-AT1G	11.75763	11.06608	12.86084	15.94406	15.13261	14.7894	13.85198	14.54992
gene-AT2G	6.304522	5.583092	5.982865	10.55472	12.23426	10.85504	2.602639	2.297237
gene-AT2G	54.08975	48.94846	51.77567	35.3094	32.39361	34.37656	48.92799	50.16288
gene-AT5G	52.54084	47.53769	50.10066	59.36149	55.46966	56.74147	51.83739	53.25067
gene-AT1G	50.09651	51.17424	53.78696	39.08524	36.51314	37.91769	91.67395	87.57509
gene-AT2G	2.819736	2.255996	2.247114	3.391708	3.360034	3.043461	3.210236	3.490748
gene-AT4G	0.653996	0.482278	0.689899	0.292023	0.516916	0.386646	1.38785	1.411124
gene-AT3G	39.33253	40.52334	40.5639	78.72457	78.25671	79.21507	0.295708	0.234163
gene-AT3G	15.56303	13.46456	13.78943	16.74177	16.68876	15.44257	5.402298	5.816314
gene-AT1G	8.092034	7.225046	8.544801	10.46657	9.762786	9.457709	7.749815	7.890742
gene-AT1G	16.0616	16.19397	15.85902	12.46334	13.54553	12.54453	24.22304	22.97505
gene-AT2G	0.758612	0.835386	0.381293	0.332567	0.39914	0.579747	0.59579	0.648995
gene-AT2G	540.7496	520.2451	523.7601	1837.228	1860.377	1848.808	478.9083	500.5042
gene-AT2G	5.742226	4.73064	4.180922	9.138329	10.32365	11.37707	0.764567	1.081014
gene-AT2G	5.142929	4.658967	4.483745	2.370078	2.419076	2.2821	9.599761	9.561287
gene-AT5G	38.57349	38.73822	39.7038	29.85008	27.51799	28.5359	51.97404	50.71626
gene-AT3G	4.046395	2.895167	3.041434	4.461341	5.894313	5.115613	1.28011	0.923558
gene-AT5G	14.12938	13.19591	12.19947	9.514975	9.493112	8.263349	16.95658	18.46791

gene-AT5G	24.61501	23.83044	23.74726	24.30111	24.30899	26.22816	11.89744	12.42118
gene-AT1G	1.789396	2.284195	3.314918	3.085965	2.43106	3.082327	4.312364	6.066958
gene-AT2G	9.413508	8.715655	7.331084	12.54436	13.97235	14.72047	6.507156	5.915105
gene-AT5G	17.21945	14.52624	17.0483	5.573305	6.066484	5.804348	24.32772	25.2071
gene-AT2G	5.851741	6.063365	4.560472	0.587674	1.316689	0.557961	3.337001	3.570949
gene-AT1G	76.98638	72.77905	74.97807	47.22375	47.46808	47.71734	50.87804	55.11612
gene-AT5G	0.590204	0.791457	0.759845	24.91613	25.68029	23.0451	1.761398	1.817199
gene-AT1G	12.63013	11.99165	12.83219	18.73754	18.24759	17.20676	7.778231	6.845926
gene-AT1G	7.218763	6.042942	6.980628	3.530452	2.092372	1.8044	6.706276	4.570718
gene-AT2G	62.48018	69.98781	59.09763	18.29711	19.09579	21.94473	100.1964	83.79015
gene-AT1G	5.778756	5.456013	6.182854	5.365646	6.514152	5.386945	10.44632	8.585087
gene-AT2G	4.118238	3.646522	3.648991	5.621328	5.653856	5.576636	4.755559	4.231424
gene-AT1G	172.6211	170.5293	177.3707	97.35276	90.93465	94.24431	259.0989	265.3024
gene-AT3G	10.85724	8.70546	8.377107	5.147171	4.593797	5.017707	13.96317	13.66938
gene-AT3G	2.416707	2.789327	2.868518	3.801629	3.087393	2.337342	6.170818	5.423892
gene-AT1G	29.85764	27.57585	28.38846	16.59467	14.72156	17.13098	28.94934	28.67346
gene-AT1G	17.07007	15.06391	14.6157	17.06839	16.78793	16.8212	23.24618	19.32885
gene-AT1G	1.588158	1.576805	1.617792	2.53335	2.621682	2.341886	0.751119	0.934828
gene-AT5G	110.7286	117.8907	115.6359	81.38155	81.59362	79.1184	97.28408	103.3653
gene-AT3G	0	0.413941	0.138517	1.693803	1.760104	1.630373	0.446557	0.444879
gene-AT3G	0.638239	0.864913	0.585917	1.136825	0.95551	1.14229	1.847643	2.044949
gene-AT1G	0.936398	1.519512	1.268797	1.271922	1.530016	1.357812	0.431301	0.898562
gene-AT5G	10.07351	9.816747	9.23987	6.570178	5.39046	6.570919	6.011526	5.571505
gene-AT4G	1.14711	1.617383	1.317836	0.957339	0.727964	0.921667	0.851695	0.947017
gene-AT4G	16.67592	16.8166	15.84079	14.33394	13.15892	14.13047	16.35695	15.60161
gene-AT3G	86.64548	87.70574	84.73358	35.17701	35.66552	37.76491	135.1724	135.5549
gene-AT4G	432.8728	429.5403	442.1157	341.3665	331.364	333.4576	532.9772	556.198
gene-AT3G	8.04223	9.330749	8.041466	10.17221	10.00054	9.059309	3.65846	3.436479
gene-AT4G	5.178188	5.294584	5.110366	12.12334	14.27158	14.06479	1.762358	1.508102
gene-AT1G	87.22976	80.30582	80.25916	94.38145	95.78249	94.33397	32.21732	32.78361
gene-AT3G	6.222224	5.711492	6.145053	6.636337	7.092062	7.525059	3.662398	2.833273
gene-AT5G	48.17347	48.4202	50.85477	46.09187	42.86729	43.36338	49.37943	50.84306
gene-AT1G	1.118649	1.283911	1.533758	10.94211	10.35178	9.941382	2.677827	2.74604
gene-AT4G	7.333884	7.161173	7.99603	3.731401	4.070414	4.987241	5.273221	5.157355
gene-AT5G	2.593871	4.037657	2.949275	2.776875	2.743143	2.482143	2.750628	2.61181
gene-AT3G	60.52755	55.3017	58.93523	69.19234	71.51544	73.71063	40.29197	39.03708
gene-AT4G	8.994864	9.975842	10.98893	15.90412	16.5607	17.10426	0.716448	0.757648
gene-AT1G	28.2897	26.41203	23.26717	57.17606	54.95461	54.57035	14.82844	16.90574
gene-AT1G	32.68658	31.38331	31.89297	31.38042	31.28641	33.51244	18.88823	19.01851
gene-AT3G	70.94412	71.67337	71.73602	42.57922	41.79208	42.55054	128.5823	133.2029
gene-AT3G	17.57398	17.88128	17.92363	31.32087	29.31579	29.03651	18.13115	19.59437
gene-AT5G	1.165017	1.552052	2.079342	1.060268	1.398189	0.933411	2.65254	2.725897
gene-AT3G	90.845	92.16863	87.72186	122.8906	125.5435	124.8704	31.86075	31.16686
gene-AT5G	0.075998	0.113517	0.023561	0.28529	0.233677	0.299713	0.021346	0.038108
gene-AT5G	2.932755	2.397385	2.275867	4.948343	5.764915	7.041213	0.68366	1.632603
gene-AT5G	110.9671	110.5273	114.6054	121.0797	125.6797	121.0563	137.1177	147.8489
gene-AT3G	10.88214	10.24845	10.00187	7.878719	7.498675	7.167951	15.89082	15.78838
gene-AT2G	4.562035	5.718887	5.322876	5.040121	4.995495	4.659893	3.033369	3.225867
gene-AT2G	14.34649	11.25855	11.80051	9.884494	9.646208	9.412506	4.0709	4.604991
gene-AT2G	3.869186	4.418356	4.303672	7.356362	7.932579	6.47105	12.31593	11.74535
gene-AT4G	7.880688	8.492674	8.506419	9.918882	12.54428	11.38667	5.693016	5.362778

gene-AT4G	37.08149	36.43549	38.56911	12.33221	9.987429	12.13077	8.742036	7.643434
gene-AT3G	4.232481	3.284901	4.355373	5.38586	5.018965	6.758725	1.576172	1.016303
gene-AT5G	9.711538	9.9322	10.04865	4.706918	4.17499	4.381528	10.38592	10.45207
gene-AT1G	1.700518	1.734275	1.87373	0.929649	1.373484	1.365053	0.64388	0.637327
gene-AT3G	1.396752	1.694151	1.656736	1.708913	2.669645	2.789619	3.871808	4.526163
gene-AT1G	1.159463	0.173681	0.599378	0.275594	0.743449	1.171618	0.477476	0.195266
gene-AT1G	60.14956	67.54204	66.12261	80.67902	79.46023	81.78998	57.69848	59.29731
gene-AT4G	0.919774	0.746601	1.126075	0.438791	0.403742	0.465872	1.675922	1.096003
gene-AT1G	7.204955	6.38933	6.099541	7.54678	8.828301	7.304441	8.019377	8.217775
gene-AT2G	335.0742	324.6067	340.0654	234.1955	235.6252	236.7364	554.377	548.0767
gene-AT5G	10.26138	9.580059	10.14946	13.51017	13.05762	14.61759	4.126729	4.344448
gene-AT2G	8.126135	9.477847	7.915123	13.01392	14.24132	14.12645	5.15999	6.70176
gene-AT2G	11.24272	9.945325	11.86933	6.405513	6.176501	6.608747	14.90822	16.52559
gene-AT2G	2.18301	1.901609	2.282602	1.218589	1.075555	1.228889	2.148875	2.843717
gene-AT3G	4.074038	4.128875	4.341053	5.686003	4.071612	5.668325	7.578644	6.640643
gene-AT4G	46.02949	46.77243	45.95106	28.14387	26.70042	24.61672	53.12265	57.81403
gene-AT2G	5.250886	4.68352	4.386734	11.72922	13.20697	11.33764	1.9706	1.539824
gene-AT3G	1.723495	1.984938	2.067156	3.557974	3.256577	4.021686	2.307427	2.099696
gene-AT5G	44.40908	42.32566	46.69602	27.60313	25.94398	25.83627	73.57613	68.65404
gene-AT2G	49.38266	52.31468	47.83672	13.41975	14.70902	13.61826	14.43565	16.54684
gene-AT2G	39.33028	42.0351	40.81856	56.65343	56.4808	56.35863	25.6971	29.75831
gene-AT1G	0.24491	0.108597	0.233666	1.408585	1.276098	1.268441	0.608689	0.649088
gene-AT4G	12.14713	11.71892	12.80307	8.864438	9.905982	10.48192	25.91985	23.07919
gene-AT4G	0.88714	0.704161	1.014131	0.562916	0.621755	0.398009	0.190947	0.062632
gene-AT5G	2.422798	2.550357	2.653411	1.539518	2.34079	2.327877	3.748316	3.301948
gene-AT1G	15.55018	15.87608	14.55836	26.42906	27.89079	26.0298	1.955214	0.958873
gene-AT5G	1.697719	1.36883	1.047817	1.065726	1.149603	0.811972	2.148522	1.950226
gene-AT5G	0.743981	1.449225	0.79414	0	0.197413	0.395774	2.43878	2.444387
gene-AT5G	110.8556	103.0406	111.3487	75.48848	73.5066	69.22107	162.7214	165.4974
gene-AT5G	164.3595	138.5073	169.5593	77.03434	66.72713	75.65728	32.51473	29.48156
gene-AT1G	146.4804	144.5919	144.8121	99.58386	102.4755	92.999	209.2251	211.7097
gene-AT3G	38.66112	38.83909	37.71574	29.65405	28.36563	30.28844	52.13638	58.37132
gene-AT1G	14.299	12.07744	10.39323	16.75405	17.12174	17.14486	11.00771	12.52459
gene-AT2G	4.379135	4.505173	4.484759	5.211201	4.813443	4.054366	5.282828	5.500336
gene-AT3G	11.94788	10.68798	11.17086	10.08896	11.26902	10.45702	2.59141	2.154857
gene-AT1G	0.145334	0.150388	0.086967	0.370227	0.255096	0.369494	0.298321	0.172915
gene-AT1G	3.406114	3.795607	3.924645	7.867821	7.946496	7.590425	4.48992	4.04354
gene-AT5G	37.19858	37.38646	37.46523	23.68207	24.05128	24.77973	3.478185	4.252077
gene-AT5G	2.33863	2.402009	2.808136	3.243453	3.562028	3.005309	1.107899	1.193912
gene-AT5G	0.980776	0.817054	0.868236	0.869948	1.103313	1.172756	1.635735	1.558646
gene-AT5G	2.62762	3.316889	2.736973	1.683893	1.852082	1.19979	0.396658	0.586978
gene-AT3G	18.75311	18.62688	19.21367	12.6787	9.184108	14.4899	30.06047	28.67419
gene-AT4G	1473.061	1441.763	1490.195	845.9037	845.4506	852.7562	2303.361	2307.054
gene-AT1G	0.256326	0.27676	0.428789	1.675467	1.258755	1.838711	0.280775	0.512015
gene-AT4G	0.822737	0.649045	1.023802	1.340342	1.181091	1.635374	0.743882	0.659024
gene-AT4G	5.979844	5.212455	5.152175	5.376135	4.093085	5.309312	2.507355	2.39668
gene-AT5G	33.09409	36.40402	34.60079	39.63457	39.01652	39.32964	20.24216	19.89331
gene-AT1G	3.553368	3.803378	4.069476	9.879252	9.619508	9.281664	4.356232	4.745156
gene-AT3G	7.822404	8.194617	7.611683	8.157019	8.247246	8.152778	4.811784	4.709818
gene-AT5G	0.635308	0.312554	0.54203	0.691805	0.593739	0.491311	0.317644	0.32255
gene-AT3G	28.61085	28.53285	29.42882	39.51632	40.93559	35.2541	33.53794	36.32254

gene-AT1G	3.671215	3.096339	3.419044	91.51302	86.42569	90.4509	2.570618	1.516526
gene-AT2G	7.247746	6.866742	5.856706	10.79717	9.518473	11.58453	2.31416	2.287134
gene-AT4G	43.4053	41.27001	43.07694	27.91172	30.66977	28.17011	65.97608	68.77068
gene-AT5G	7.617251	7.584439	7.092816	4.878916	4.473884	4.837654	9.146529	9.119151
gene-AT5G	7.460419	7.989598	6.857305	3.051785	3.576288	4.1946	9.339314	7.867779
gene-AT1G	26.88011	26.36835	24.60032	12.21293	12.25159	12.17119	13.91305	15.52765
gene-AT3G	13.00972	11.8774	13.90197	11.27273	9.766742	9.834623	18.74231	17.62083
gene-AT3G	1.311703	1.008329	1.356859	0.491929	0.568819	0.263437	1.450687	1.562059
gene-AT5G	5.8387	4.558564	5.577985	6.091373	6.393869	6.55602	1.377983	1.703098
gene-AT3G	21.40896	19.0486	17.55234	17.38318	15.56293	18.41447	28.27816	22.94066
gene-AT5G	0.590936	0.81056	0.507994	1.003478	0.603553	0.703057	0.883109	0.841977
gene-AT4G	2.785856	4.020332	2.951567	50.8051	52.22372	50.62764	1.13071	1.042154
gene-AT4G	1.756925	1.439163	1.690247	1.419004	1.89724	1.213503	0.662315	0.780282
gene-AT4G	8.728523	9.299655	8.684131	7.351557	9.007684	8.118426	12.85194	13.24031
gene-AT1G	0.058546	0.107607	0.10898	0.365358	0.480978	0.465778	0.378318	0.075566
gene-AT5G	2.650027	2.607796	2.836162	1.553091	1.938603	2.316464	3.812273	3.229788
gene-AT5G	1.848974	2.260949	2.582497	2.36624	2.872303	2.241705	1.476185	1.871726
gene-AT3G	24.91783	26.4391	27.03427	34.37183	35.85622	36.64335	4.532182	4.503256
gene-AT1G	3.496632	3.515638	3.212579	1.811816	2.075125	2.301595	1.215096	1.760291
gene-AT2G	2.920734	3.222437	2.800419	5.442019	5.977491	5.760336	4.212032	6.003734
gene-AT4G	117.1877	117.8123	119.1399	84.04128	83.90075	83.84686	178.5916	182.4249
gene-AT2G	3.93365	5.535564	4.593008	6.53871	4.686863	5.66875	2.66891	3.518262
gene-AT4G	4.200386	4.247204	4.286483	3.750678	4.895775	4.741495	3.653232	4.730227
gene-AT2G	20.18644	20.3479	19.91649	21.06665	19.22609	19.44504	15.39676	18.33795
gene-AT2G	449.1534	369.6495	437.0094	203.5422	214.5855	223.4552	755.2605	779.7211
gene-AT1G	34.24028	35.91314	29.45053	16.22703	16.1745	15.54144	67.31577	69.89416
gene-AT1G	2.972807	2.214461	3.076337	1.70917	2.118698	1.747181	2.457861	2.435747
gene-AT2G	2.019179	2.229569	2.376617	2.014118	1.894266	2.801515	3.188401	2.137743
gene-AT2G	51.02413	47.21761	46.38381	22.18861	19.82217	18.45556	18.6482	21.75549
gene-AT3G	151.9541	151.0956	150.832	70.97718	70.98338	71.22266	113.3902	117.9667
gene-AT2G	2.611744	2.407773	2.470401	16.62435	18.06962	14.55988	0.324147	0.370779
gene-AT5G	27.91965	25.98959	26.2399	10.30979	10.82124	10.5111	8.532991	8.061932
Arabidopsi	1.43629	1.252444	0.678019	1.3577	1.740157	0.976232	1.247667	1.436629
gene-AT1G	4.36725	3.007257	4.225743	24.27625	22.75977	24.41357	1.83565	1.2747
gene-AT1G	93.00451	88.9335	90.09875	125.9346	121.6657	122.1348	47.66638	49.33799
gene-AT3G	7.902481	7.139674	6.530824	13.92416	13.11986	13.12018	2.182896	1.675709
Arabidopsi	2.51925	2.080593	2.447649	8.840501	8.541549	7.788786	1.254457	1.180796
gene-AT2G	6.347411	5.774651	5.238722	3.17631	3.821045	5.116631	10.10232	10.27318
gene-AT1G	19.27517	20.28133	21.26362	9.564356	9.625766	9.586755	5.614365	6.132736
gene-AT2G	20.08137	19.02743	18.54143	15.2878	13.40706	14.30194	29.22698	34.22446
gene-AT2G	20.4296	18.64953	19.1536	20.94071	20.77634	20.03065	8.306191	9.346003
gene-AT5G	3.6467	3.109404	3.430774	2.294109	2.755239	2.394212	0.867699	0.997036
gene-AT3G	6.751678	7.70109	6.96093	9.379373	8.215501	8.480926	6.093121	6.355467
gene-AT4G	5.776035	5.945552	6.669876	2.105209	1.771401	1.409089	2.237066	1.482122
gene-AT5G	48.72091	49.36136	50.39601	50.54293	48.52247	48.12788	77.61063	80.59298
gene-AT1G	16.38692	16.00769	16.23476	10.89558	11.41123	11.92069	26.12023	25.81735
gene-AT1G	103.5989	102.3886	99.30325	144.4611	149.2479	146.9957	101.9803	105.8923
gene-AT1G	90.35156	90.29964	86.81072	91.25829	91.4854	91.03469	38.65631	34.64168
gene-AT5G	12.11528	10.66859	9.464739	12.85422	16.59622	14.83862	3.09049	3.696485
gene-AT1G	34.7959	32.47428	34.64495	22.78352	22.97777	25.36289	48.11538	49.5293
gene-AT4G	41.19848	38.41291	38.27093	82.50201	79.69202	81.69756	26.34072	28.56849

gene-AT3G	1.823299	2.823772	2.841871	0.788149	0.943815	0.923467	1.148812	1.312747
gene-AT5G	10.52831	9.916653	9.154857	10.33443	10.09105	10.18621	4.281095	3.634942
gene-AT2G	0.872574	0.875401	0.588759	0.728959	0.632568	0.745701	0.670697	0.569108
gene-AT2G	1.910885	1.667492	1.820127	3.31322	3.240234	2.175819	0.716801	0.463341
gene-AT1G	0.711509	0.895718	0.569903	1.695166	1.929517	2.552011	0.332707	0.557261
gene-AT1G	1.085658	0.0666	0.096803	1.492011	2.039151	1.743101	0.977747	0.194126
gene-AT1G	2.777626	3.288937	3.586388	5.9143	5.593734	5.899963	1.357622	1.622247
gene-AT4G	5.341291	4.216991	5.042554	6.179929	6.063367	7.177033	5.36998	6.407395
gene-AT4G	8.466447	8.975234	9.364416	19.33448	20.16003	19.8918	7.914626	7.521563
gene-AT3G	7.881147	6.812297	8.382082	5.757996	6.911126	5.969381	9.603541	10.04347
gene-AT1G	3.142011	1.561829	3.146294	4.078909	4.77564	4.617702	5.12966	6.415974
gene-AT5G	68.96527	73.02581	71.69356	49.12427	50.5903	44.37101	136.4919	134.1194
gene-AT2G	748.9479	789.1719	743.6162	319.8854	337.3125	336.4664	920.2755	873.2124
gene-AT2G	11.62399	11.99622	12.26755	3.671756	3.930058	4.014998	20.61001	21.73345
gene-AT5G	0.63579	0.326365	0.358066	0.447101	0.440485	0.498531	1.001029	1.648812
gene-AT1G	7.227758	9.706418	11.01094	3.566796	4.44629	3.918859	27.7855	27.68087
gene-AT1G	1.828308	1.740768	1.837541	0.512917	0.528735	0.282449	16.87305	16.61123
gene-AT5G	33.48577	30.1576	33.33241	8.693776	8.576487	8.50594	78.38165	82.15895
gene-AT1G	5.877043	5.566317	6.293604	10.0672	9.33812	9.657077	2.330647	0.959546
gene-AT3G	283.7573	291.8572	286.9895	183.899	184.5144	193.4731	204.2544	200.3297
gene-AT1G	22.03472	22.55588	21.90485	15.21051	13.90365	13.37664	9.295696	8.202692
gene-AT3G	23.50395	23.39871	25.10933	16.29044	14.51102	15.69495	28.77341	34.14073
gene-AT3G	52.89861	45.03893	44.78646	25.88562	28.56938	29.58765	83.94404	82.39637
gene-AT3G	2.070481	4.584563	4.407009	1.482442	0.815748	1.164706	4.961522	5.138395
gene-AT4G	4.410627	5.130415	5.48631	6.025266	4.898696	4.129778	5.77935	7.595096
gene-AT1G	6.862423	9.111574	9.218977	2.783643	3.737853	3.506532	5.048353	6.347056
gene-AT1G	84.52808	87.50919	83.31147	38.26032	39.6818	40.92158	72.7515	78.7056
gene-AT2G	6.758166	6.668149	6.361045	6.469113	5.587456	5.575137	6.077905	6.474233
gene-AT1G	3.666057	3.759139	3.977476	1.934178	1.409962	1.739045	3.43305	4.118986
gene-AT4G	1.126748	1.134762	1.282441	0.602357	0.936557	0.705447	1.526698	1.469151
gene-AT5G	102.3991	100.1893	101.9169	49.31004	47.11504	46.40324	60.65055	65.10684
gene-AT1G	6.111213	6.924893	6.814812	2.594229	3.223684	3.476553	7.641635	8.796835
gene-AT1G	4.027223	4.449605	4.131046	4.340942	3.936694	3.551633	1.491109	1.517804
gene-AT1G	2.79885	3.671894	2.575528	4.762815	3.627604	4.062438	2.051892	1.637559
gene-AT2G	12.55176	11.94416	12.36203	6.688447	6.645594	6.779208	2.008428	2.375867
gene-AT3G	1.602874	1.313362	1.927686	2.049587	2.265509	1.928153	1.442879	1.100404
gene-AT1G	25.63474	28.34733	27.62853	5.862687	5.805209	5.670891	4.514343	4.180206
gene-AT3G	10.28488	8.971873	9.888609	26.49582	29.41715	26.89309	11.09832	9.793142
gene-AT1G	10.3852	11.91841	10.25302	7.393666	7.359277	8.771439	26.6616	25.21215
gene-AT3G	11.96669	13.59181	12.46193	42.66559	39.0983	36.80384	2.839213	2.469763
gene-AT1G	36.78358	36.05968	34.01175	25.13298	26.9018	24.27205	26.02411	30.7065
gene-AT1G	1.623094	2.14388	1.789137	0.808384	0.929647	0.64951	0.60861	0.380353
gene-AT5G	6.259629	7.846443	5.7886	8.245306	7.43208	6.001351	4.368001	4.309129
gene-AT5G	21.47451	22.37534	20.31033	25.99921	24.34171	27.80194	20.58355	17.4101
gene-AT2G	0.381742	0.355885	0.379536	1.16619	0.615118	0.475828	0.166741	0.28132
gene-AT1G	28.57865	30.94324	27.80577	56.73856	50.84006	54.7021	8.363327	9.291881
gene-AT3G	1.911179	1.598101	1.31555	3.407897	2.932781	2.377849	0	0
gene-AT2G	8.523818	8.870452	9.176194	9.85633	10.47925	8.549212	4.344477	4.11803
gene-AT5G	3.559669	3.18807	3.84241	5.957684	5.740926	4.939265	0.894233	0.953147
gene-AT2G	2.55523	2.78347	2.147616	0.695488	0.434445	0.664644	0.315136	0.467937
gene-AT1G	59.71394	60.50394	57.64301	167.5748	165.7964	155.7282	33.83486	36.78751

gene-AT4G	5.28612	4.697417	5.584062	6.288289	6.073937	5.992136	5.433012	6.956676
gene-AT3G	1.87634	1.722893	1.755257	1.081888	1.081034	0.512979	2.307648	2.231662
gene-AT4G	311.2997	311.3343	302.5682	133.3826	139.0152	131.5624	202.8558	212.7884
gene-AT2G	294.8413	293.1518	302.8581	381.8838	376.1531	377.9749	442.3425	465.3873
gene-AT2G	3.53124	2.443501	1.354088	0.473897	0.153578	0.176967	6.824801	5.200764
gene-AT1G	43.55051	44.81735	40.6819	34.00259	35.45323	29.54019	59.05368	67.47111
gene-AT5G	1.711315	2.200258	2.246503	1.378584	1.151945	1.276345	1.907766	2.37476
gene-AT5G	2.342683	2.388115	3.264832	0.781151	1.201363	1.135152	0.281784	0.285447
gene-AT5G	5.847774	5.631344	6.83237	11.11147	10.4486	9.506138	3.3209	3.666082
gene-AT1G	15.33019	15.42743	14.89433	6.598728	7.614531	6.193453	23.40783	27.18168
gene-AT5G	1.786034	1.542264	1.650858	1.161843	1.212049	1.467578	0.707207	0.846699
gene-AT2G	56.06106	56.04541	58.87841	95.88146	95.99042	94.46883	68.60083	76.80669
gene-AT1G	639.1522	640.0832	516.6779	412.1162	412.3155	373.2769	846.6095	917.5134
gene-AT5G	1.139197	0.99729	0.765069	1.327019	0.922849	0.986319	1.006709	0.879832
gene-AT5G	1.85605	1.704155	1.537406	4.521344	3.851718	4.551405	3.904045	4.205093
gene-AT1G	2.285427	2.217406	1.802627	2.662734	1.857086	1.954168	0.934313	0.76422
gene-AT4G	10.27954	10.51395	8.555169	16.36207	15.524	15.29641	5.935503	7.512372
gene-AT1G	38.09151	35.4071	37.70816	38.77919	35.08106	37.24964	24.40725	23.29677
gene-AT1G	0.415284	0.436754	0.578484	0.430141	0.588658	0.574806	1.626994	0.95419
gene-AT3G	18.07458	16.58589	16.70407	4.870269	5.288646	4.383297	8.529991	8.802005
gene-AT1G	5.245049	5.402235	5.417694	5.237877	4.6732	4.444879	5.972565	6.26232
gene-AT2G	45.40805	44.45025	45.50087	13.58728	14.7512	13.66902	12.99176	18.28324
gene-AT2G	37.0033	40.29873	41.60366	28.85745	28.43016	28.22464	62.69681	62.1762
gene-AT1G	1.821813	1.583971	1.410937	1.458977	1.325119	1.628792	0.537266	0.491856
gene-AT3G	6.27454	6.059785	6.490784	27.99093	29.51184	28.93861	2.807116	2.990779
gene-AT3G	2.450315	2.087736	2.165228	1.144763	1.086603	0.816229	4.488682	3.318353
gene-AT4G	6.837707	7.706655	7.251828	7.191195	5.34896	6.820733	9.536785	12.76339
gene-AT4G	0.260557	0.268818	0.900102	0.532332	0.752075	0.554927	2.599079	1.714673
gene-AT4G	7.710888	7.537161	7.554344	13.00538	12.45702	12.20674	2.76994	2.633168
gene-AT2G	31.21595	29.6107	32.2442	60.22694	60.40964	55.57826	18.80752	18.5455
gene-AT4G	10.93833	11.57143	10.32862	6.509289	5.936896	5.931619	5.592236	4.105421
gene-AT5G	1.024491	1.637233	1.435354	3.415855	3.319261	2.465164	1.678829	1.39049
gene-AT1G	12.43831	10.79122	12.09465	12.36572	12.32068	12.20977	18.5511	18.14321
gene-AT5G	5.584156	5.068281	3.479264	2.369507	2.274366	1.938444	0.591209	1.225932
gene-AT4G	11.61067	12.0415	12.0085	10.58438	9.885115	9.537927	2.875854	3.104654
gene-AT1G	14.74266	13.51615	15.95221	9.735414	9.86099	9.216472	26.0759	26.69444
gene-AT2G	11.66712	10.75862	12.37279	7.109169	7.176394	6.711536	4.382492	4.375806
gene-AT5G	17.83659	16.10548	15.19303	101.8322	101.679	110.2361	13.96602	12.08512
gene-AT1G	13.45713	11.51325	12.45595	17.71651	23.36859	22.88022	9.010368	10.80119
gene-AT5G	1.10752	1.528754	1.715971	4.42926	3.516779	3.313864	1.599584	0.766323
gene-AT2G	0.497149	0.694588	0.397966	0.506437	0.533225	0.53069	0.388612	0.381997
gene-AT5G	58.22789	58.20085	62.25273	47.24639	48.82029	45.31363	87.61233	90.33519
gene-AT1G	2.176276	2.510248	2.812539	1.296366	2.296787	1.492382	0.761129	0.599432
gene-AT1G	4.292549	4.600583	4.178973	5.833074	7.316705	6.277039	3.187822	2.87933
gene-AT5G	23.18445	23.64934	21.7847	21.73197	21.48018	21.91111	41.14618	41.86713
gene-AT4G	16.52802	17.36614	17.38545	11.46922	12.09355	11.78634	34.54239	38.18431
gene-AT5G	48.71576	50.30013	49.76096	99.47742	94.14027	94.994	47.50942	50.81306
gene-AT1G	3.635537	4.348656	5.413747	1.991946	2.498326	1.887408	0	0
gene-AT3G	7.574266	7.3707	7.376811	7.241128	7.20338	7.139084	8.795971	9.552037
gene-AT3G	27.43543	27.78044	30.7608	9.309065	8.804165	9.00096	5.865942	6.417559
gene-AT3G	1.833347	1.612645	0.825001	0.851311	0.815134	1.878989	0.387726	0.591411

gene-AT3G	68.14966	75.85054	70.46373	41.95019	37.46024	39.10438	61.43298	63.0481
gene-AT3G	25.86298	26.12017	24.44909	48.72189	50.79389	49.76352	4.083497	4.747933
gene-AT1G	1.492905	1.564222	0.77364	1.144141	1.177114	1.025928	0.09121	0.205715
gene-AT5G	186.5183	182.7292	197.401	9.167559	8.426332	9.443512	12.11224	11.63444
gene-AT1G	18.2503	18.68465	20.66986	15.33896	16.29655	15.88489	30.06808	28.35336
gene-AT3G	10.37267	11.83681	9.834504	8.339876	9.858769	8.70339	16.86185	17.10262
gene-AT5G	11.23868	10.54451	10.25473	26.27734	26.76217	25.38369	14.52836	15.02301
gene-AT4G	1.398782	2.442538	1.902483	2.746922	2.484112	1.841349	1.679647	1.913489
gene-AT1G	358.0379	357.6225	354.3483	171.0396	178.3549	172.2012	634.6805	673.9341
gene-AT3G	4.405273	4.458411	4.519042	7.031066	6.735689	5.996344	13.9436	12.9882
gene-AT3G	8.56201	8.255166	9.361482	8.422042	8.881678	6.371851	6.078247	6.006697
gene-AT5G	2.196023	1.043499	1.50667	4.949346	4.97865	4.554075	3.12399	2.697999
gene-AT5G	44.64296	47.43026	43.23395	132.6319	137.0131	133.5647	48.86114	45.50033
gene-AT3G	20.41523	21.28158	20.36702	19.09185	17.87249	16.32458	4.205632	4.944498
gene-AT5G	3.808161	3.290676	3.66231	3.605958	3.776002	3.175317	2.983495	2.230595
gene-AT1G	19.04147	18.22454	19.91717	27.22144	25.54884	25.9057	22.96017	22.39321
gene-AT1G	26.74955	23.01733	27.00295	11.5208	11.38207	11.07967	47.58737	43.98481
gene-AT3G	4.368051	4.144736	3.76535	1.619147	1.70099	1.305516	5.063262	4.980822
gene-AT3G	3.658657	3.586034	4.291424	4.696552	4.506836	4.986609	5.016468	4.682358
gene-AT1G	0.931522	1.2068	0.957163	1.439545	1.621434	1.421446	0.447578	0.327443
gene-AT4G	6.636118	8.988969	12.46918	7.45188	7.385855	8.277349	11.94617	10.30198
gene-AT5G	32.78967	36.77447	38.49194	28.03007	25.81422	26.20617	44.66319	46.98399
gene-AT1G	16.53791	16.52725	17.14466	13.97792	15.19523	13.21009	22.22122	23.14453
gene-AT1G	7.386563	6.623266	8.535028	8.946138	9.62711	7.32429	20.0572	22.26605
gene-AT2G	0.77536	0.779293	1.42436	1.956824	2.340735	2.272979	0.174462	0.506351
gene-AT1G	3.665493	3.534414	4.241831	3.942456	3.162163	3.068293	11.12501	12.58963
gene-AT2G	9.181028	9.689194	10.4415	5.764683	5.599011	4.793924	12.28291	12.71052
gene-AT5G	4.329535	3.991674	3.804992	3.73388	3.771996	4.330698	4.962147	4.538573
gene-AT2G	2.675734	2.875705	2.828121	1.51083	1.896304	1.749855	5.576096	6.293189
gene-AT2G	1.398942	1.412039	1.513874	1.304653	1.513371	1.180467	1.864317	1.395869
gene-AT3G	8.493886	10.73088	9.387797	12.3597	13.02307	12.68156	7.05375	9.150835
gene-AT1G	0.832651	0.903026	1.181528	2.554464	2.365531	2.575455	0.379486	0.303745
gene-AT3G	5.499873	4.715594	4.612929	4.681764	4.351344	4.902932	0.908668	0.906951
gene-AT2G	4.199458	4.197754	4.759009	4.97891	3.771664	4.415396	7.088697	8.610038
gene-AT5G	1.377174	2.033104	1.663768	0.680032	0.638136	0.732931	3.107985	3.725165
gene-AT1G	1.510629	1.10473	1.490645	0	0	0	0	0
gene-AT3G	5.811942	5.83925	6.308224	5.936252	6.362216	5.73834	5.313434	5.80558
gene-AT2G	1.583415	1.602862	1.337914	43.52912	44.77031	45.83401	2.964041	2.847974
gene-AT4G	8.79161	11.05776	9.217631	1.063076	0.904835	1.445435	17.56758	17.15084
gene-AT2G	251.22	269.4844	267.5873	177.4404	182.0273	181.0625	402.1385	362.7326
gene-AT3G	6.550962	7.038551	6.996589	14.8422	13.84761	13.28226	18.90446	18.69879
gene-AT1G	0.845558	0.990283	0.867463	1.247594	1.266925	1.114889	0.401304	0.256947
gene-AT1G	19.96115	19.21648	21.22847	6.137495	6.662554	5.884792	27.09215	29.25372
gene-AT1G	9.000126	9.216136	10.84304	6.440502	6.891738	7.059126	17.02378	17.80883
gene-AT3G	7.379214	7.443941	8.789073	2.538168	4.22545	3.491072	11.34679	9.865147
gene-AT2G	22.10838	20.50218	21.82089	14.21573	15.02428	14.73168	5.35562	5.795266
gene-AT1G	102.5301	94.819	96.7741	70.28268	73.48302	83.25026	172.5629	181.8608
gene-AT1G	1.818756	1.773383	1.888879	0.608683	0.605519	0.573305	3.606604	3.262181
gene-AT1G	11.81533	13.19318	11.78954	8.864122	9.002224	9.866361	2.323877	3.109251
gene-AT5G	0.608857	0.534631	1.08587	1.433477	2.049385	2.1647	0.147348	0.894629
gene-AT2G	22.08626	22.05313	22.30271	47.19755	50.65514	45.00473	16.19682	20.99136

gene-AT5G	2.804765	2.165554	2.127121	3.696836	4.196469	4.681336	1.285322	0.883549
gene-AT4G	4.757339	6.323234	7.303816	13.89586	13.99257	10.32587	2.55131	1.919593
gene-AT4G	526.6654	516.6989	531.6837	395.7303	397.2003	393.6087	512.4797	528.6299
gene-AT5G	23.35378	21.36883	24.94694	14.34159	13.57014	12.64268	40.88895	41.91526
gene-AT1G	0.990506	1.144848	0.854358	1.321883	1.447783	0.886837	2.136246	1.976384
gene-AT1G	3.497416	3.813449	3.29149	3.025945	3.625087	3.639223	3.55316	3.050435
gene-AT2G	61.03033	60.28709	62.53678	40.35952	40.73318	38.33177	117.2666	117.3232
gene-AT5G	32.17996	34.38849	33.32435	22.6391	21.58064	20.29467	57.41566	57.17086
gene-AT4G	53.32532	51.02046	52.35081	61.90299	62.67204	61.23732	17.96011	20.49872
gene-AT3G	2.466502	2.444237	3.996775	2.545698	2.643334	3.446846	0.271334	0.043853
gene-AT3G	5.420576	5.411121	5.63994	4.234285	3.408756	4.368723	1.896415	2.382305
gene-AT2G	4.661329	4.948903	4.756511	2.220077	2.553368	2.254098	0.588444	1.082495
gene-AT5G	48.45814	48.82793	52.21091	35.16205	37.82253	36.48488	73.93955	72.06281
gene-AT3G	21.30472	20.43145	23.55159	14.49132	16.1922	14.97439	28.89758	25.86648
gene-AT3G	3.278624	2.484735	2.608043	3.519668	3.387933	3.712251	0.763176	0.595023
gene-AT2G	2.437242	2.155127	2.02984	2.493629	2.069164	1.998435	2.902156	2.831746
gene-AT5G	1.11338	1.187554	1.915662	0.806065	0.807479	0.576463	3.85437	4.11144
gene-AT4G	2.934154	3.662677	3.629818	1.371042	1.277755	1.099381	0.227868	0.252193
gene-AT5G	278.0728	282.5749	271.4088	103.9655	104.2897	101.7571	499.293	521.8724
gene-AT4G	8.420425	8.323981	8.460389	3.235426	4.180536	3.564025	3.160235	2.893539
gene-AT1G	9.151087	10.74238	9.723598	3.629231	4.97344	3.987712	20.0186	21.87642
gene-AT3G	0.246936	0.227341	0.288487	0.343188	0.223204	0.145596	1.392817	1.638045
gene-AT3G	2.666182	2.576135	2.763692	5.437967	4.347874	5.09379	0.990452	2.334561
gene-AT3G	0.584145	0.573213	0.526648	1.021568	1.185866	0.663276	0.227351	0.349624
gene-AT1G	1.678403	2.411858	2.709449	4.016362	4.410092	5.119004	0.984302	1.29663
gene-AT5G	57.02982	56.36451	55.65579	75.88007	76.49065	75.90416	34.85076	32.99091
gene-AT4G	32.96475	29.01997	33.22599	41.37263	38.97676	39.94685	26.58747	24.31027
gene-AT4G	2.354797	3.343431	2.550238	1.682041	2.009088	1.592815	4.547019	4.315704
gene-AT4G	0.31014	0.565826	0.396088	0.319647	0.502522	0.192099	0.888982	1.194249
gene-AT4G	27.04082	24.95306	25.04717	42.60723	40.84441	39.76504	17.90615	19.43859
gene-AT5G	26.99591	22.97908	25.60507	19.55311	23.65098	21.93727	8.583423	7.788497
gene-AT2G	1.948134	1.905257	2.030967	2.935259	3.553805	2.318926	4.094617	3.922135
gene-AT2G	2.638625	3.515766	2.531365	8.790914	7.390575	8.93737	6.101709	4.86517
gene-AT1G	48.21795	39.87938	44.89912	33.9691	36.02047	33.41313	67.89537	70.12698
gene-AT3G	12.77052	12.90337	11.55359	6.129288	6.35035	6.881912	20.24301	21.38116
gene-AT3G	11.4855	11.29991	12.09896	15.90703	17.15268	15.33346	8.808711	9.239597
gene-AT3G	20.30964	18.99784	20.86751	15.21354	15.82552	15.04758	22.48961	23.44102
gene-AT4G	23.91797	23.64772	21.88331	37.72932	38.87726	39.47153	14.62911	15.08609
gene-AT2G	0.304746	0.319442	0.150667	0.540331	0.336443	0.535237	0.450238	0.406097
gene-AT2G	7.502744	7.279133	8.408679	6.47711	7.799544	7.998944	9.965373	11.18968
gene-AT2G	30.06125	28.95158	30.31885	21.23789	23.82308	20.79314	21.64701	22.62976
gene-AT2G	17.15216	16.61466	16.61731	14.4619	14.42341	15.86662	6.758177	6.241152
gene-AT1G	0.979931	1.183615	0.931783	2.65823	2.765441	2.971308	0.507309	1.093108
gene-AT4G	73.4989	68.33717	73.61916	77.96138	78.60368	79.16264	67.69508	72.22167
gene-AT3G	5.924448	7.443211	6.882755	11.13139	10.41174	10.7434	2.301548	1.72409
gene-AT5G	8.908882	9.275226	9.363666	10.75573	8.728976	8.384564	3.674371	4.380796
gene-AT3G	46.20348	44.69182	47.48819	28.46176	30.08843	29.81735	12.23557	12.30226
gene-AT4G	6.406165	4.679281	6.294309	13.75717	14.2502	13.10354	7.885185	9.630856
gene-AT1G	154.0805	155.1572	158.4078	87.32161	80.82427	79.50721	246.315	247.9957
gene-AT2G	404.2138	446.8809	379.3514	361.9718	402.359	413.3219	41.21927	38.62354
gene-AT1G	2.442393	1.645539	2.69478	0.899676	0.361084	0.437017	0	0

gene-AT2G	0.543183	0.25305	0.26135	0.820288	0.905547	0.76557	0.585045	1.100706
gene-AT1G	5.224696	4.749148	5.480914	4.202078	3.312663	3.425744	13.95344	13.4725
gene-AT5G	1.602831	1.246233	2.338979	1.079425	0.719525	0.456138	5.746787	4.468904
gene-AT1G	1.219615	1.394775	0.910908	2.255349	1.405588	2.223102	0.107489	0.085853
gene-AT3G	14.2181	15.41754	15.22508	15.26153	15.46468	13.76443	8.386776	8.45095
gene-AT1G	0.428455	0.188982	0.530606	1.085877	1.473288	1.971544	0.213171	0.510979
gene-AT5G	33.65336	32.66113	33.83159	55.25148	57.29451	57.57312	15.79598	17.85373
gene-AT4G	2.403449	2.099958	2.171578	3.127341	2.844503	3.839983	0.619102	1.054165
gene-AT1G	1.107586	0.249776	0.615539	2.000727	1.376878	2.095502	0.562624	1.392139
gene-AT3G	1.129375	0.89356	1.449	0.348254	0.399916	0.3584	0.451595	0.318454
gene-AT5G	7.850382	6.774969	8.347492	6.635056	5.755054	5.387856	13.57822	14.08329
gene-AT5G	7.646905	7.8408	5.915417	9.413004	9.698237	7.512867	2.472825	3.430953
gene-AT3G	10.748	10.2375	9.841023	6.675679	6.01732	7.485038	16.1176	15.07142
gene-AT1G	661.7947	641.7875	658.9004	262.3568	268.5538	253.9838	385.269	385.2376
gene-AT3G	4.128647	3.344791	3.505463	1.444931	1.002127	1.088011	1.433602	1.768209
gene-AT5G	1.732809	1.568514	2.105134	3.029241	2.884604	2.273902	2.644802	2.999882
gene-AT1G	31.83599	31.9648	35.46288	74.0483	72.65218	72.05174	22.77187	22.41291
gene-AT2G	2.602067	2.170954	3.792672	3.889325	3.531959	3.000423	4.553032	4.74349
gene-AT1G	16.38172	15.6389	15.65051	10.28925	10.96716	10.35232	19.77106	20.0266
gene-AT4G	5.222324	5.961464	7.577459	3.600537	4.692773	4.157237	8.988554	8.534035
gene-AT3G	1.313418	1.626974	1.382347	0.116182	0.086409	0.187618	0.124069	0.148875
gene-AT5G	0.55882	0.461649	1.014312	0.815454	1.350641	1.215183	0.906231	1.000153
gene-AT5G	26.14854	26.00571	24.18485	6.800402	7.492425	8.085823	18.60329	18.40302
gene-AT4G	0.526692	0.380407	0.668014	0.216368	0.079033	0.314851	0.35092	0.480032
gene-AT1G	12.52601	12.34616	12.9551	16.0555	17.12465	15.71422	6.633887	8.607464
gene-AT3G	2.928588	2.138057	2.296014	5.225294	5.495875	6.015585	3.073739	2.942025
gene-AT5G	67.14325	70.27027	69.17071	47.55559	52.27136	45.58272	121.395	128.4416
gene-AT1G	0.321695	0.353412	0.340641	0.406433	0.188865	0.41437	1.107459	0.861613
gene-AT1G	105.8006	108.4829	99.61454	9.805028	11.59796	8.853711	9.629856	9.008038
gene-AT1G	13.90139	15.87019	14.38347	4.364069	4.87649	5.495215	12.46967	10.77831
gene-AT1G	52.19521	51.04104	51.93688	32.19694	34.72267	34.76981	83.62023	86.84032
gene-AT1G	3.179655	3.292306	2.865026	4.12674	3.914536	3.493776	5.266167	4.552264
gene-AT4G	11.74365	11.19357	10.21487	9.152448	11.60201	8.289097	17.61016	21.74215
gene-AT2G	14.97235	13.56607	14.08451	11.18271	12.11699	12.03055	16.13711	15.2651
gene-AT4G	6.890335	6.94665	7.093148	11.62754	11.55736	10.35658	3.624023	3.535123
gene-AT3G	2.135801	1.589304	2.529892	5.293594	5.207041	5.298219	0.377279	0.437058
gene-AT1G	7.089517	7.183355	6.546219	9.5851	8.473476	8.253502	8.768511	9.924241
gene-AT5G	23.75211	23.22087	22.37224	15.99192	17.91456	14.74517	31.32667	31.54507
gene-AT5G	3.081552	4.031871	3.887309	7.497291	7.029857	6.853548	2.912384	3.070738
gene-AT3G	1.621424	0.680215	1.425789	2.767244	1.829306	2.133944	0.266863	0.340029
gene-AT1G	34.83053	35.22177	39.5932	22.69058	26.88612	23.05612	55.95341	55.42776
gene-AT1G	98.58372	98.72524	102.3543	30.85647	27.85635	31.71623	24.16729	25.28205
gene-AT4G	0.659199	0.622756	0.156672	0.3642	0.295329	0.643212	0.114084	0.06118
gene-AT2G	8.212467	9.047133	6.957045	5.853193	5.778899	5.58614	16.58456	14.30013
gene-AT1G	7.655481	6.759586	7.423789	5.239404	5.494184	4.018901	7.95501	7.53964
gene-AT3G	3.926889	5.493345	4.249172	6.747074	5.125121	6.235084	4.366008	3.580903
gene-AT2G	15.75941	17.52238	14.70361	15.52471	16.1541	16.41875	6.284536	5.57856
gene-AT1G	1.148723	0.766002	0.779191	2.101138	1.17036	1.786005	0.532882	0.602793
gene-AT2G	5.962801	6.657228	6.269449	15.0702	16.76016	15.02565	21.32323	19.39737
gene-AT5G	0.171686	0.452708	0.270453	0.57014	0.781586	1.099103	0.365998	0.236872
gene-AT1G	9.075577	9.026442	9.293373	4.93013	6.082669	6.097624	12.06104	14.47881

gene-AT4G	4.210229	4.826251	4.031673	2.407251	2.59178	2.301039	0.400078	0.141702
gene-AT2G	0	0	0.184136	0.136868	0.196179	0.523489	0.162455	0
gene-AT3G	0.241705	0	0.223223	3.741944	3.407152	3.018495	0.074501	0.244347
gene-AT5G	12.80887	11.40667	14.01965	7.434579	7.410961	8.111667	15.87895	18.04559
gene-AT5G	20.71835	21.77839	22.44278	10.5587	10.76497	10.12623	5.457354	4.655426
gene-AT5G	11.25571	11.39255	10.74219	91.53476	90.72797	92.01432	20.19431	20.34841
Arabidopsi	1.314482	2.69309	3.000035	4.282679	3.48669	4.595697	4.898051	4.00938
gene-AT3G	64.94859	63.83674	64.30451	50.41229	48.80563	50.26176	84.67944	86.55882
gene-AT5G	10.22335	9.483276	9.553494	9.684972	11.40358	9.82637	33.55222	34.72222
gene-AT2G	3.623586	4.454109	4.331852	5.437692	4.492318	5.050018	1.4869	2.674481
gene-AT1G	2.246108	1.892598	1.657943	2.126132	1.587424	1.775025	0.563702	0.273255
gene-AT3G	4.305857	4.107155	4.494436	2.279944	2.95973	2.95428	1.536883	1.760591
gene-AT1G	3.772952	6.888309	7.983974	7.406098	10.1457	8.961042	4.232057	2.437436
gene-AT3G	15.39383	12.94162	14.42511	6.110353	4.613158	6.821711	0.058597	0.25053
gene-AT1G	7.006307	6.005392	6.071786	6.939175	7.626969	7.239943	19.10641	19.61533
gene-AT5G	65.45274	65.72988	64.75376	55.28971	53.83422	51.37455	85.80014	88.75863
gene-AT1G	16.80919	16.44518	17.49404	9.782833	11.84349	11.3511	8.226574	8.11133
gene-AT4G	84.87755	77.12485	74.15465	100.954	105.1135	101.319	99.1124	93.46469
gene-AT1G	0.189648	0.136239	0.329974	0.414822	0.762588	0.73882	0.076881	0
gene-AT4G	42.75542	44.26913	43.91029	80.12829	82.01771	74.47789	56.02269	49.4836
gene-AT1G	15.41318	13.08022	14.49076	10.11309	9.529664	10.34482	16.49583	16.558
gene-AT3G	1.409612	0.796644	0.806049	1.288768	0.977098	0.945214	1.093037	1.125394
gene-AT4G	11.96444	11.76215	14.68694	6.564947	6.033505	5.870676	16.56826	16.84871
gene-AT2G	6.389214	5.516158	7.004684	6.431097	7.370113	7.421081	15.0573	15.89809
gene-AT4G	1.484689	1.813955	2.030684	4.112245	4.005206	3.785529	1.150679	1.423923
gene-AT2G	388.231	378.1927	379.6572	193.6742	187.3743	193.3968	259.8649	277.8883
gene-AT4G	31.62221	31.23053	33.36355	45.19619	44.23151	48.41743	31.66162	37.51616
gene-AT4G	130.7166	125.3761	129.3875	200.9277	196.8027	191.7621	72.92687	70.98177
gene-AT4G	17.85945	18.58949	18.33181	6.339962	7.962464	6.964946	17.94756	16.97498
gene-AT2G	0.710932	0.981557	0.812275	1.994449	1.259655	0.920693	0.236648	0.856724
gene-AT4G	22.3378	23.42741	23.50331	22.87983	18.9473	21.64351	33.44499	34.1388
gene-AT4G	4.426823	4.970457	5.236162	4.98187	5.50052	4.867953	5.448677	5.20841
gene-AT3G	5.361635	5.174444	5.295082	8.69921	8.358238	8.659013	8.840093	9.662726
gene-AT2G	24.66722	22.89199	25.52281	22.03229	20.40228	21.7797	10.27656	11.55703
gene-AT5G	17.2414	17.08099	16.77336	19.09607	18.53325	18.50636	2.873588	3.424922
gene-AT1G	49.11414	49.92512	52.85558	27.10882	27.54183	27.35212	60.46254	62.02024
gene-AT1G	9.832072	8.204192	10.25529	6.786528	7.066644	5.583496	25.86585	30.56281
gene-AT1G	11.24557	11.3676	9.887348	7.687235	8.521314	8.21138	16.10942	18.20041
gene-AT1G	1.259179	0.819485	0.826371	3.356479	4.779885	4.696501	0.356359	0.89696
gene-AT5G	23.61168	23.635	29.87956	57.94386	62.90305	59.05075	47.77898	49.46436
gene-AT1G	8.473422	9.231824	8.664632	6.205768	6.649969	6.638799	15.68101	16.22334
gene-AT3G	20.09434	19.05718	18.65342	7.289818	9.163773	5.991558	10.60633	9.082512
gene-AT4G	21.22101	20.65616	22.87027	58.3452	57.9319	58.85544	23.70872	26.9763
gene-AT3G	2.130939	2.041499	0.396742	2.690022	3.18398	1.326869	0.244346	1.257176
gene-AT1G	1464.049	1412.953	1467.506	797.5025	794.2423	813.8904	1819.514	1805.278
gene-AT5G	139.1238	141.8401	141.9733	109.0867	112.4036	110.5628	250.7181	263.0358
gene-AT1G	1.10851	0.485194	0.579955	0.310334	0.367068	0.127925	1.157225	1.657503
gene-AT3G	4.759279	4.605157	5.188	6.168981	6.765274	5.524314	6.425234	6.213938
gene-AT1G	9.229288	8.93546	8.412365	9.069455	10.91804	10.30765	6.555935	5.738771
gene-AT1G	1.885569	2.306763	2.438117	3.751537	2.806894	2.729406	2.858471	2.486085
gene-AT3G	2.506088	2.799813	2.948866	4.748524	5.25037	4.790135	1.378013	1.292217

gene-AT5G	1.174362	1.395092	1.138454	2.513488	2.313745	2.921998	0.225378	0.456394
gene-AT5G	171.7282	182.2267	179.9048	136.4264	138.8967	133.146	243.0582	248.6303
gene-AT1G	1.111296	1.011836	1.126376	0.661088	0.858432	0.488155	0.525065	0.477119
gene-AT3G	24.26671	27.55096	28.37043	27.39377	25.27565	23.8507	50.44477	49.24672
gene-AT1G	75.50501	74.03938	79.64596	41.71446	41.61432	42.32798	31.76331	34.06428
gene-AT1G	23.09954	22.2654	24.40862	22.41571	19.80138	20.86596	28.89679	31.60958
gene-AT3G	9.052774	8.354371	9.24929	8.385142	7.585299	10.31325	7.871532	8.621247
gene-AT2G	1.972408	2.084037	1.772255	1.682158	1.84621	1.744908	1.419516	0.889487
gene-AT1G	37.70292	35.95477	36.47334	41.71879	41.97829	43.06658	26.61678	30.70399
gene-AT5G	1.545188	1.510409	1.775509	3.285117	3.494576	3.753176	1.342743	1.543906
gene-AT3G	33.68926	30.99176	29.89353	34.82795	32.10759	33.00188	49.35921	52.62469
gene-AT1G	50.67538	47.96112	49.21667	92.68439	89.70461	87.64367	19.67101	18.36258
gene-AT1G	37.09849	34.4557	38.23757	33.19455	29.93106	31.57974	60.42533	57.37901
gene-AT1G	1.233762	1.124582	1.049463	1.549169	0.35154	0.667836	0.460232	0.156702
gene-AT1G	31.50509	28.73371	29.28123	20.20684	15.72926	16.45919	9.294597	10.74075
gene-AT3G	2.635834	2.481159	2.186941	4.238276	2.443031	2.566791	1.145246	0.785283
gene-AT3G	11.56828	11.93842	11.76834	15.02974	14.35578	15.14216	10.49994	11.95197
gene-AT3G	19.26243	19.97291	19.97037	3.108497	3.082692	3.051919	2.004915	1.751603
gene-AT3G	1.097979	0.720366	0.770673	0.850111	0.728134	0.918476	0.309121	0.352373
gene-AT2G	66.99103	67.86749	63.74554	118.2319	120.754	121.7867	136.536	137.7857
gene-AT4G	5.087145	4.623821	5.771467	1.906339	3.620006	2.873108	1.476184	1.072743
gene-AT2G	8.920609	9.73587	7.957366	3.664893	3.623056	3.102546	1.776057	1.394977
gene-AT4G	43.10159	45.20299	41.12694	18.3955	18.17574	19.31346	39.43227	40.3088
gene-AT5G	0.600069	0.602246	0.63091	1.476539	1.063402	1.264377	0.678299	0.708008
gene-AT4G	3.386275	2.550752	2.748229	6.950869	6.5278	6.18763	1.477431	2.098659
gene-AT2G	11.63677	12.60307	12.70387	12.28322	10.81692	12.08318	20.65484	18.38289
gene-AT4G	235.6253	233.1573	248.1317	164.8574	158.7058	156.2715	419.5033	439.3019
gene-AT5G	18.81429	14.25312	13.50035	14.52133	14.43475	17.03101	7.785466	5.809697
gene-AT4G	2.656654	2.56269	3.133682	1.563642	1.76246	1.682255	0.849037	1.063241
gene-AT3G	3.047408	4.191164	3.784182	2.795969	2.843756	2.266022	0.295274	0.276089
gene-AT5G	6.536541	6.872966	7.258568	26.42095	24.73037	25.62532	4.118562	2.475748
gene-AT2G	3.472351	3.259451	4.346388	2.710208	3.085116	2.06826	0.503668	0.465194
gene-AT5G	2.289747	2.628844	3.525927	4.267153	5.244803	4.460277	3.809765	3.135667
gene-AT4G	16.23599	13.92147	15.87944	10.47313	10.72656	10.39783	6.987549	5.86681
gene-AT2G	7.27453	5.516433	6.800551	5.374267	5.921757	4.971497	8.161253	7.640587
gene-AT1G	0.267438	0.175115	0.274169	0.18039	0.249854	0.325149	0.894149	1.012201
gene-AT4G	21.70848	20.11302	22.42095	17.61832	18.36608	17.21745	26.91441	26.7385
gene-AT2G	0.524379	0.562259	0.451791	0.380806	0.169603	0.294264	0.411996	0.656975
gene-AT2G	7.025154	6.446084	8.512263	2.162682	3.192087	2.854922	3.063088	2.812761
gene-AT2G	3.99623	3.421685	3.748765	1.952178	1.435219	1.64767	2.140566	2.174416
gene-AT3G	1.779684	2.116005	2.117808	0.827816	1.202295	0.787376	1.345515	1.450079
gene-AT2G	4.528292	3.987091	4.495123	3.104147	3.233886	2.825671	5.829451	5.602635
gene-AT5G	112.0813	138.1439	118.5918	50.03483	53.93146	52.80876	15.84145	15.42374
gene-AT4G	22.9565	25.37375	22.42739	7.089804	7.609809	8.519065	40.65614	43.03878
gene-AT5G	23.83661	24.62464	24.84815	13.55833	11.7734	12.07648	14.98471	15.72295
gene-AT3G	0.48001	0.544828	0.680539	0.817314	0.60347	0.859503	0.743126	0.249069
gene-AT1G	12.91516	14.05117	15.96658	19.60055	20.5289	22.91452	11.1029	10.00833
gene-AT4G	0.525774	0.181275	0.350603	0.294929	0.312988	0.37586	0.337226	0.505684
gene-AT3G	11.31026	10.2584	10.07392	11.6075	13.38043	11.61124	4.71751	3.701381
gene-AT3G	1.429564	1.316864	1.140283	1.439236	1.422637	1.123718	3.094	3.5929
gene-AT5G	8.678771	7.809476	8.44854	7.527395	9.498519	8.632792	3.406573	3.827445

gene-AT5G	6.343196	7.707119	6.576272	6.911654	6.437567	7.941891	1.751235	1.889319
gene-AT3G	17.66233	17.34572	17.41585	21.06963	20.30639	22.02911	10.90873	10.09501
gene-AT4G	6.959079	6.748114	6.295321	8.40629	8.262812	5.863599	12.10375	10.87867
gene-AT4G	176.3251	186.1445	182.4748	242.2743	247.3081	227.428	104.4593	102.914
gene-AT3G	6.362754	6.462138	5.935727	9.05179	6.612022	6.928791	4.313629	3.495976
gene-AT3G	3.281093	3.698612	4.34692	2.772925	1.928301	2.560143	2.133813	2.005079
gene-AT5G	15.1749	15.51938	13.76583	17.99219	17.34582	16.88466	6.200063	5.314574
gene-AT5G	9.040408	8.65969	8.167786	8.229515	7.39966	8.564601	13.40418	13.13205
gene-AT1G	82.93373	81.85076	88.76561	101.2944	104.4161	100.4437	57.7336	59.32358
gene-AT5G	18.78542	18.8799	20.86628	25.77517	28.13571	26.35304	31.41692	28.23285
gene-AT2G	1.508075	0.656226	1.789839	0.647519	2.341306	1.575324	1.36649	1.265111
gene-AT4G	23.31958	23.14319	23.22936	16.26739	19.43449	17.39802	34.76924	37.35018
gene-AT4G	69.87795	73.05527	61.99207	44.11843	48.75241	47.85052	123.66	126.1436
gene-AT1G	57.53043	57.48891	62.1058	48.58119	49.4066	49.87771	65.84624	63.1014
gene-AT1G	15.10532	16.07606	14.25067	11.97329	10.52628	12.30813	17.12793	19.81345
gene-AT1G	4.621981	5.254683	4.899183	6.92573	7.866206	7.739098	4.917423	5.464067
gene-AT1G	39.95583	38.33529	40.19012	21.34784	23.70902	22.60134	142.1293	147.3124
gene-AT5G	32.63704	31.54058	32.58305	20.70753	20.39888	20.7975	36.26061	36.64008
gene-AT3G	2.128799	2.665354	3.044505	3.346212	2.412615	2.609162	9.67845	8.946802
gene-AT1G	103.9856	101.6619	107.9263	26.43903	29.74291	24.86148	83.70006	89.47405
gene-AT5G	3.919341	3.464044	3.321181	4.771459	4.639446	5.19344	3.010507	3.175841
gene-AT1G	16.4207	21.36409	18.23704	16.14512	18.71568	16.14204	24.68127	22.79975
gene-AT4G	5.374627	4.949672	5.699913	3.160493	2.560251	3.050498	5.818462	6.642443
gene-AT2G	31.80893	29.77753	30.97812	20.78477	19.98001	19.49589	45.64674	51.14437
gene-AT1G	1.202076	0.824394	0.948287	1.948613	3.127765	2.127925	0.675207	1.101203
gene-AT2G	45.15459	43.42825	46.98039	34.34272	33.82682	32.56287	18.19996	19.37597
gene-AT1G	1.399155	1.217153	1.909197	1.282592	1.05994	0.74	0.856852	0.316821
gene-AT5G	6.644616	6.939951	6.2708	79.75685	80.39918	75.72122	1.97216	1.617014
gene-AT1G	4.982982	4.717654	4.061766	6.519746	7.556816	7.137465	5.102301	4.79298
gene-AT4G	5.488857	5.562998	6.808776	4.525342	3.692491	5.393686	6.541295	7.669796
gene-AT5G	5.382149	6.214604	5.248309	3.334027	2.982716	3.776737	8.624666	9.944002
gene-AT3G	71.35201	69.79636	71.13557	51.25804	49.37397	50.847	57.95729	61.91625
gene-AT5G	1.642317	1.637072	2.080041	0.932814	1.094437	1.400474	1.250731	1.24765
gene-AT1G	49.64181	48.34362	48.67013	36.59051	37.14569	37.16328	34.04631	35.31165
gene-AT3G	1.339634	0.528307	0.463866	0.585279	0.259927	0.264472	0.640067	1.27842
gene-AT1G	2.054242	2.928505	3.642284	4.300261	4.128778	4.06991	0.954408	1.797671
gene-AT5G	1.528606	1.335339	1.759741	0.714207	1.264091	0.58353	4.849868	4.171638
gene-AT2G	0.073631	0	0.027335	1.563971	1.496811	1.888438	0	0.108474
gene-AT2G	206.8535	209.3878	200.6425	118.3542	124.0192	122.0441	49.38293	50.32481
gene-AT2G	6.165312	5.997623	6.061796	4.705463	4.490793	4.505405	2.393352	2.445577
gene-AT5G	0.922419	0.738943	0.89518	0.632946	0.437527	0.995817	2.349686	2.16209
gene-AT1G	17.43342	16.28407	16.78432	10.25872	8.705971	10.46483	5.102239	6.275574
gene-AT5G	17.17682	19.68647	21.64148	13.98284	14.50467	12.17037	5.396036	6.272469
gene-AT2G	0.902517	1.642317	1.430804	0.752191	0.966879	1.030916	0.304492	0.478349
gene-AT2G	9.227135	8.709137	7.8313	7.883818	6.763412	7.079807	23.78478	23.92712
gene-AT1G	2.58881	2.421934	2.674284	3.549418	3.730739	3.458366	1.298321	1.132005
gene-AT4G	69.77093	70.05797	61.95719	109.2416	112.6598	112.1769	56.18591	52.57803
gene-AT1G	1.538552	0.835258	1.210029	1.036522	0.859215	0.710026	1.289498	1.41479
Arabidopsi	0.283398	0.424012	0.495189	0.476684	0.452615	0.437835	0.392618	0.363236
gene-AT1G	7.945013	7.691591	6.827131	3.985855	4.337148	3.505769	1.323096	1.206882
gene-AT1G	0.5109	0.503447	0.406447	0.329261	0.261074	0.482593	0.989481	0.952262

gene-AT5G	5.361182	5.716747	5.423491	11.22103	12.12128	11.16629	6.820633	7.947846
gene-AT3G	63.78158	69.7129	64.09969	81.4935	84.02841	87.83629	102.9781	88.32845
gene-AT2G	0.50749	0.972994	0.863301	0.425018	0.664638	0.466302	1.129989	1.216399
gene-AT5G	1.297265	1.329042	1.310615	0.969874	0.467515	1.200788	0.713523	1.600772
gene-AT1G	2.340967	1.912524	2.031809	4.501497	3.599022	3.648772	3.640296	3.946614
gene-AT3G	1.233676	0.565254	0.868581	1.266112	0.358578	0.788969	0.474932	0.861569
gene-AT5G	6.081847	5.21952	5.066386	3.435082	3.660498	3.214824	12.72829	12.96264
gene-AT5G	30.89585	26.70555	28.63031	31.15662	28.59416	28.97996	39.93868	45.50446
gene-AT2G	47.19033	45.42022	45.23632	54.42544	50.46662	51.8304	46.06893	48.2798
gene-AT3G	203.5146	222.8146	214.7136	219.9047	221.7896	229.7782	416.8749	410.6049
gene-AT4G	3.083109	2.4214	3.358003	6.475235	6.410736	6.378601	2.731288	3.652585
gene-AT5G	10.46837	10.71555	8.157527	8.592603	10.50024	10.79641	1.831697	2.029727
gene-AT5G	14.42026	17.62844	17.04122	40.01312	38.59936	40.84238	23.79658	17.99359
gene-AT4G	2.3591	1.334843	1.653513	0.29243	1.307341	0.139257	4.666872	1.983229
gene-AT2G	1.094894	1.60457	1.418926	2.638155	1.95133	3.123725	0.470066	0.607212
gene-AT4G	18.61854	16.32242	19.13634	14.13929	14.01722	12.71358	21.72904	22.31223
gene-AT2G	57.6106	55.78703	59.19037	82.00565	81.45721	80.77264	33.60089	34.22933
gene-AT5G	1.377144	1.809687	1.45773	1.444383	1.345247	1.918689	0.53366	0.42249
gene-AT3G	7.341498	8.639254	7.483429	8.416333	9.016729	9.156052	8.885264	10.31171
gene-AT5G	8.996868	8.292235	8.597154	9.48207	9.534879	9.443701	2.934134	3.144285
gene-AT3G	0.424035	0.513456	0.325091	1.193687	1.160257	1.744791	0.810414	1.176226
gene-AT1G	3.173752	2.601836	2.857418	16.03766	15.06945	14.73928	5.760173	5.955921
gene-AT4G	61.17696	57.5722	57.91917	22.51258	22.67145	24.45768	59.18736	65.02161
gene-AT4G	0.063705	0.041993	0.073849	0.10002	0.101703	0.087723	0.019898	0.052963
gene-AT3G	197.7891	191.7317	193.3658	140.2254	143.0927	141.4738	329.3537	315.3945
gene-AT3G	0.518892	0.598297	0.656638	0.32024	0.357194	0.509729	2.150803	2.05595
gene-AT5G	2.44836	3.608065	2.853871	1.993602	2.810117	2.33541	1.144797	1.534292
gene-AT5G	5.575745	6.185223	5.750007	6.348297	5.229571	5.355707	5.189581	7.858932
gene-AT4G	2.116734	1.766189	1.735524	3.923954	3.727066	3.678704	2.234855	2.100632
gene-AT1G	6.315715	4.085789	4.593004	1.173074	0.72855	0.856915	1.702161	0.922247
gene-AT1G	21.11593	18.36657	18.17967	4.28487	4.754186	5.211363	6.69107	7.165469
gene-AT1G	326.7193	324.0903	333.0842	263.2815	258.4091	255.2373	612.8233	566.9518
gene-AT3G	1.597286	1.758373	1.872531	0.433733	0.490808	0.418965	0.521045	0.393557
gene-AT3G	1.830364	1.745123	2.210873	0.590457	0.331295	0.256105	0.127298	0.088576
gene-AT5G	2.968488	3.207963	2.918192	24.19061	23.62918	25.66293	2.172056	2.947401
Arabidopsi	3.249048	2.068151	2.652486	2.201179	2.141031	2.215211	1.216137	1.344575
gene-AT3G	3.917757	4.525506	5.263037	6.173471	6.812246	7.657918	2.648073	2.224797
gene-AT2G	1.109519	1.442678	1.339813	1.547566	1.554401	1.897117	2.269017	1.411753
gene-AT1G	132.2655	125.1104	128.3488	13.34014	13.19996	14.27387	15.40621	15.03127
gene-AT4G	8.350337	7.691081	7.949906	3.055598	2.194917	2.99526	5.877043	4.851779
gene-AT3G	5.654002	4.414432	4.580647	5.158473	4.323948	4.436141	1.403846	1.781501
gene-AT1G	84.52508	82.17969	80.95526	40.75949	43.27218	42.50332	41.17453	42.77623
gene-AT5G	4.084478	4.910052	4.913991	14.60433	13.91395	15.31686	3.260056	3.590101
gene-AT1G	10.09019	11.02914	10.16665	14.61427	13.87575	12.09613	3.560774	3.454864
gene-AT2G	3824.064	3757.125	3898.527	1448.851	1423.023	1426.65	4665.622	4641.924
gene-AT2G	8.653539	9.170561	9.87213	2.343859	1.320927	2.074077	0.272264	0.650865
gene-AT3G	20.3051	21.35562	19.68834	10.89816	11.63275	10.10179	28.18513	27.89664
gene-AT3G	1.249735	0.583452	0.815084	0.874362	1.018209	0.707157	0.090325	0.161898
gene-AT3G	1.901752	3.213857	2.033947	0.639374	1.731165	1.388784	0.556332	0.304444
gene-AT1G	2.75604	2.722033	3.269285	6.982258	6.747303	6.117065	2.385303	2.245904
gene-AT1G	1.864804	1.76205	2.018462	3.382	1.947152	2.528491	2.793277	1.937442

gene-AT5G	12.93586	12.37464	12.4199	5.296329	7.090204	6.242686	1.150157	0.935781
gene-AT4G	35.05547	35.72848	30.64499	3.751164	5.706576	5.592944	32.87477	34.63048
gene-AT3G	1.767294	2.303837	2.325437	3.960875	3.313736	4.668985	0.769672	0.988353
gene-AT2G	2.214881	2.813919	2.588933	3.325433	2.619977	3.125176	1.584126	1.824612
gene-AT1G	4.06184	2.898262	3.085816	10.38405	11.0197	7.900876	4.165012	4.320132
gene-AT4G	21.27889	22.76899	21.68553	21.64826	22.14602	18.16173	7.838128	9.084485
gene-AT1G	22.34592	20.76597	22.53298	6.026556	6.172211	6.851433	42.49743	45.82402
gene-AT4G	125.9082	121.2475	126.8753	85.16176	85.97321	84.86675	20.647	20.00699
gene-AT2G	2.528536	2.128861	2.443003	1.904169	1.657547	1.607658	2.930931	3.581109
gene-AT1G	6.29289	6.495407	5.704311	12.78536	12.65446	13.25636	4.816463	4.628692
gene-AT3G	1.422752	1.659684	1.565663	1.493871	1.648737	1.602681	0.396508	0.367317
gene-AT1G	90.81753	90.37064	93.31243	55.69697	55.35908	58.26404	175.9843	171.2203
gene-AT4G	2.189245	2.154081	1.153428	0.740202	1.326233	2.040949	1.894276	1.661295
gene-AT5G	5.88672	6.100045	5.853458	4.001715	3.169507	2.785016	0.799115	1.160775
gene-AT5G	10.38971	11.78802	10.33154	12.07307	12.20516	10.9624	9.082639	8.850638
gene-AT1G	47.97583	48.25196	47.1634	44.43712	42.83315	42.77771	20.63645	22.77077
gene-AT2G	20.87096	20.25892	20.50784	12.82278	13.00363	13.25141	36.24746	33.10894
gene-AT2G	3.2378	2.022716	2.561632	2.89282	3.187984	3.288943	1.38852	0.99164
gene-AT2G	20.34881	19.69966	19.62159	36.18688	30.57793	33.75912	30.79741	34.19961
gene-AT1G	5.827102	5.204701	5.305981	5.945251	5.457197	6.058224	3.492186	4.145249
gene-AT2G	37.37054	36.3235	36.68521	34.42821	36.05072	38.29747	24.84833	21.19593
gene-AT1G	2.188319	2.04215	1.741084	3.670032	4.509313	4.49396	2.531769	2.341159
gene-AT5G	24.22074	28.27115	25.58549	12.69887	11.04654	10.60582	40.63488	46.58043
gene-AT5G	7.070891	7.076404	7.103601	3.186173	2.955264	3.607929	6.944498	5.636017
gene-AT2G	0.359244	0.150528	0.3214	0.249401	0.556204	0.424936	0.90978	1.218544
gene-AT4G	65.16277	67.94257	65.60059	58.17915	58.57771	62.43813	23.96391	22.69217
gene-AT5G	16.98869	17.81423	17.61317	7.960019	7.925862	7.738669	2.597555	2.699223
gene-AT4G	2.750548	2.824273	2.772449	8.708065	6.170543	6.533654	3.780412	3.097721
gene-AT5G	1.525283	1.42136	1.213929	1.060647	1.647455	1.024037	0.396839	0.183102
gene-AT2G	14.13944	15.52947	17.2652	15.051	13.29134	14.71951	21.99117	23.51069
gene-AT2G	0.613044	1.192065	0.934286	3.829409	3.33839	2.972844	1.06934	0
gene-AT1G	4392.322	4400.068	4581.196	4086.26	4121.011	4006.283	7112.841	7337.433
gene-AT5G	16.4007	17.27472	18.39616	18.40238	17.66031	17.43215	7.256159	8.238047
gene-AT3G	0.857727	1.016206	0.845924	0.436998	0.371704	0.408683	1.01829	1.185451
gene-AT5G	9.149914	7.855098	8.226638	4.980619	5.087671	4.514278	6.555158	6.645499
gene-AT2G	12.73571	15.68908	15.37427	14.2502	13.86833	13.26346	30.98842	29.29576
gene-AT4G	20.69199	20.54733	20.59796	14.9136	14.03399	14.37025	37.39354	37.86318
gene-AT4G	8.465102	9.53967	9.43579	11.57744	9.364819	10.15228	11.74043	13.02213
gene-AT5G	6.239642	7.727223	5.972279	5.283778	4.038988	4.717996	18.3157	19.02853
gene-AT3G	2.38361	3.468792	3.063799	2.642712	2.57053	3.447345	7.838613	9.108454
gene-AT4G	14.42108	13.98902	15.69324	35.8896	35.36843	34.13189	2.44953	3.376857
gene-AT1G	0.846095	0.947352	1.179909	1.917705	1.440754	1.708134	0.608228	0.469127
gene-AT4G	14.91642	14.37771	16.91093	4.773851	5.285687	6.504953	8.288793	7.54006
gene-AT4G	0.767344	0.861037	0.670905	6.008244	7.343291	7.385798	0.539057	0.592599
gene-AT1G	4.952844	5.390841	6.233179	7.006627	6.617286	6.135097	7.920299	8.011134
gene-AT4G	3.977614	3.993138	3.334853	1.815478	1.789638	3.017294	3.689682	4.792496
gene-AT4G	24.31392	23.63983	23.21521	13.59435	13.13521	12.97871	37.78171	38.72564
gene-AT4G	29.18687	29.64248	28.22154	23.02161	20.14135	22.29266	12.13756	10.77583
gene-AT4G	17.30065	17.421	16.73236	15.70819	15.75514	15.12772	24.766	27.22269
gene-AT4G	3.599164	3.22447	2.891225	8.495442	9.572569	9.079354	2.685749	2.478559
gene-AT4G	0	0	0.028539	17.21856	17.44016	16.49031	0	0

gene-AT5G	0.053319	0.05321	0.037702	0.02332	0.081005	0.05036	0.024519	0.189923
gene-AT2G	61.55556	61.00384	62.46866	45.7629	52.75607	49.23917	89.45583	88.11681
gene-AT4G	13.74432	15.6899	13.78854	24.02739	24.57843	26.61539	6.441953	5.392033
gene-AT4G	11.21808	10.94909	11.34446	6.542608	5.956573	5.62799	4.376141	5.087467
gene-AT1G	73.23609	77.43972	75.97981	77.43781	77.63139	80.19841	82.75732	76.46955
gene-AT5G	1.755089	1.902379	2.505771	1.974129	2.180898	2.088767	4.106848	4.230165
gene-AT3G	42.26601	42.46492	38.76751	36.65326	32.88433	32.79044	63.30624	65.10555
gene-AT1G	3.190582	3.620459	3.658277	2.554744	1.724741	2.617452	1.719867	1.821993
gene-AT1G	101.4101	93.6742	96.48693	213.9975	216.8037	222.1868	77.86585	75.89462
gene-AT2G	5.78455	5.020174	5.743806	3.222134	2.725628	2.934021	6.910287	7.391271
gene-AT3G	5.396942	7.020868	5.048701	9.172973	9.356776	8.868119	6.537763	6.616839
gene-AT4G	28.57798	26.40725	28.42095	24.82348	24.2631	25.31444	23.80288	26.67476
gene-AT4G	64.36334	60.55759	63.79038	71.69588	73.22324	73.8639	70.65918	64.65119
gene-AT4G	7.139965	7.558114	5.867949	11.08441	10.53692	10.44187	2.572472	3.483416
gene-AT5G	1.418082	1.189629	1.673171	0.806045	0.919778	1.022326	0.678527	0.286079
gene-AT1G	4.077263	4.171129	3.576647	2.795335	2.821499	2.805123	0.750817	1.116477
gene-AT5G	120.0583	117.9302	118.0686	232.992	238.7991	232.8828	68.07051	69.29697
gene-AT4G	3.302577	2.821043	2.534606	1.765283	1.069314	1.129403	5.691955	5.616085
gene-AT2G	50.48457	51.88247	47.46269	53.54155	56.14586	58.48077	26.22111	28.4186
gene-AT5G	46.12571	37.94838	43.48203	38.8836	36.35915	36.10713	46.79904	47.18227
gene-AT1G	2.903867	3.221111	3.359209	1.617335	2.122348	1.992953	2.116529	2.361536
gene-AT4G	1.513229	2.427715	1.776576	2.731687	1.973299	2.917544	0.884698	1.255526
gene-AT4G	7.172189	7.287719	7.470549	8.229059	7.614854	8.730661	3.518506	4.154168
gene-AT4G	0.551391	0.267486	0.46721	2.180397	1.097802	1.712474	0.993219	0.411725
gene-AT1G	41.50764	38.75668	40.83133	67.28136	65.67874	69.5364	32.13546	30.78498
gene-AT2G	0.827809	1.259737	0.78342	0.389023	0.256539	0.281293	0.089736	0.255875
gene-AT5G	17.77846	18.39422	16.98456	13.2574	14.74316	15.21326	27.16048	28.62235
gene-AT3G	14.54933	14.27986	13.81783	11.98934	10.98362	10.26288	6.242715	5.888838
gene-AT5G	0.712803	1.019829	0.574833	0.049639	0.291158	0.516362	2.048926	2.655421
gene-AT3G	2.874221	1.959765	1.83844	0.85435	0.741332	0.73155	0.537402	0.693518
gene-AT5G	7.143409	5.331078	6.845176	4.383981	7.305668	6.076993	10.94816	8.375435
gene-AT3G	55.53367	54.15114	53.21469	51.10135	48.62597	47.96762	68.36005	63.93776
gene-AT5G	58.42312	58.98343	60.37715	55.82265	54.71555	55.93365	80.4267	82.45626
gene-AT1G	216.1916	218.1057	218.8167	205.1379	208.8228	209.4746	59.15682	64.14761
gene-AT2G	10.62605	10.55172	9.920672	11.42651	10.69992	10.49516	3.669951	4.590505
gene-AT4G	0.685562	0.459099	0.98327	1.550251	1.048375	1.822854	0.200258	0.804166
gene-AT3G	6.318624	6.941096	6.513149	7.515136	7.656923	8.051742	4.731582	5.791452
gene-AT5G	19.56425	19.86677	18.14128	22.96946	23.95978	21.81298	13.8709	15.44072
gene-AT1G	6.343112	5.667026	5.926329	6.04946	6.424997	5.825145	6.831559	7.296458
gene-AT1G	0.616499	0.42412	0.376858	1.107826	1.129404	0.825783	0.321882	0.455502
gene-AT1G	1.473045	1.492841	1.291426	0.422096	0.609549	0.633392	3.534042	3.960397
gene-AT3G	11.77374	11.40155	13.09036	13.36015	13.69006	13.03069	14.52166	15.02945
gene-AT1G	20.9853	20.54449	19.52574	43.64531	42.87598	43.21988	4.527159	5.091571
gene-AT3G	1.932241	2.059009	1.922358	1.73706	1.89493	1.271776	2.590781	3.169793
gene-AT3G	16.82764	16.93581	17.56846	6.248838	7.367507	6.137723	10.49007	9.312155
gene-AT1G	62.33435	58.48906	62.42013	73.85395	77.52292	76.70977	49.13826	53.12339
gene-AT5G	8.346379	9.856231	7.892941	6.613695	6.99157	6.734141	6.638153	6.900957
Arabidopsi	1.059888	1.030161	0.65806	0.369643	0.733348	0.80373	0.33729	0.16261
Arabidopsi	1.320512	0.871842	1.83974	1.013373	1.044102	0.764468	2.074221	1.744974
gene-AT2G	54.01583	53.39512	55.40725	30.03814	32.63456	32.62058	104.2351	99.48281
gene-AT3G	0.37399	0.537662	0.544412	0.617423	0.386672	0.336074	2.0953	1.996346

gene-AT4G	8.350437	7.690486	8.021157	8.784318	9.327612	8.256751	9.881069	9.859562
gene-AT5G	55.30184	55.15029	53.83964	48.668	48.36808	45.96056	87.44575	89.99828
gene-AT5G	3.833259	4.358112	3.460714	7.543061	7.075718	7.396782	5.077756	5.304229
gene-AT1G	59.38486	58.65969	57.78026	25.794	25.28661	25.9014	68.37119	69.30833
gene-AT1G	50.51492	45.44279	49.61997	19.05516	24.72644	22.33728	81.44884	81.63646
gene-AT3G	2.374517	2.288648	2.492891	2.690655	2.655542	3.096766	1.440887	1.330477
gene-AT5G	0.140502	0.221035	0.202299	0.481342	0.412523	0.681084	0.652606	1.183486
gene-AT1G	1.692286	1.947797	2.824508	0.058846	0	0.112908	0	0
gene-AT5G	3.414413	3.801861	3.559266	8.726978	8.591426	7.512264	2.731082	3.659358
gene-AT1G	8.919119	11.22623	11.62788	10.19276	9.925645	8.362116	3.907806	4.140215
gene-AT1G	6.996187	6.30664	5.994617	9.315116	8.240515	8.133249	6.030061	5.819694
gene-AT5G	24.2363	23.94428	25.22546	24.72142	23.72332	23.48921	36.6242	38.29156
gene-AT3G	22.59792	22.67496	21.63524	19.44186	18.46566	17.85136	4.986049	4.954216
gene-AT1G	18.79373	19.36288	19.8306	4.747131	4.420477	4.611084	16.94619	15.21707
gene-AT5G	0.473702	0.22011	0.529596	0.611604	0.426673	0.523328	1.849302	1.533322
gene-AT3G	0.482063	0.40802	0.619347	2.377523	2.818001	2.564066	0.581053	0.487875
gene-AT1G	0.298478	0.2154	0.301896	0.213229	0.226681	0.310038	0.693803	0.931628
gene-AT4G	0.230767	0.27147	0.286783	0.735215	1.067475	1.119722	1.050664	0.777288
gene-AT1G	103.039	99.54367	100.6742	58.63865	56.74497	60.34699	126.3808	124.7871
gene-AT4G	7.581578	6.898089	6.589954	3.255462	3.583166	3.896807	2.306354	2.665958
gene-AT1G	23.57851	21.00248	23.06311	12.71611	12.32382	13.0095	17.97657	17.23981
gene-AT5G	1.78602	0.968557	0.994477	0.507394	0.461934	0.556539	0.860653	0.67948
gene-AT4G	12.18703	11.62909	14.12417	8.049307	8.645566	7.6453	13.69996	13.89571
gene-AT2G	88.65662	109.2687	96.45837	90.34308	93.34432	99.17025	50.13767	36.77589
gene-AT1G	2.065085	1.819959	1.671626	0.824012	0.684289	0.781882	2.507875	2.702407
gene-AT3G	64.58707	64.04209	63.92498	34.5783	35.77251	36.75872	79.81108	79.33729
gene-AT5G	0.722002	0.785514	0.934599	2.095456	1.607525	2.500446	0.121541	0.279975
gene-AT3G	5.788852	5.499445	4.727098	5.195984	5.548988	5.297722	7.564265	7.386184
gene-AT1G	8.642122	8.83149	7.769429	11.72793	11.20533	10.53259	3.278848	4.044226
gene-AT5G	20.83194	21.64909	20.59172	11.19334	13.47959	12.10478	16.01104	15.71037
gene-AT4G	61.62088	54.80255	51.28626	12.74578	16.70316	12.63196	186.4726	198.2693
gene-AT4G	0.655345	1.010834	0.596664	3.848562	3.313224	2.790727	0.201843	0.154403
gene-AT2G	2.367916	1.755282	1.166721	0.205741	0.618918	0.52979	5.186669	7.166242
gene-AT5G	11.19819	11.52319	11.10785	8.287306	9.221205	9.558036	14.99955	15.09981
gene-AT1G	596.9026	605.1322	596.811	333.8889	329.0679	324.0059	1097.886	1050.366
gene-AT4G	1.110026	1.518539	1.44185	1.302166	1.224439	0.896833	0.458529	0.585469
gene-AT1G	0.405997	0.973822	0.407522	0.920964	1.392004	1.498064	2.629932	4.184672
gene-AT5G	1.886858	2.460995	1.501263	2.284633	1.745843	2.371021	0.670412	1.105162
gene-AT3G	25.07024	29.04747	28.71891	19.03083	14.62409	16.46667	43.55541	39.152
gene-AT3G	1.277618	1.616682	1.175021	2.201948	2.1714	2.160364	1.262277	1.435187
gene-AT2G	3.861479	1.481776	2.40184	0.314137	0.867601	0.492681	0.566339	0.124467
gene-AT5G	3.815068	3.502806	3.569662	3.423711	3.113493	2.942401	8.578638	8.240346
gene-AT1G	1.39955	1.095002	1.699935	1.254225	1.183128	1.274132	0.498671	0.526379
gene-AT4G	1.126099	0.506905	1.091411	1.581147	0.599695	0.834747	0.910698	0.964436
gene-AT1G	11.90374	11.84311	11.86312	13.09231	10.68246	13.70116	8.13923	8.309329
gene-AT3G	208.3618	207.6426	207.5905	1110.659	1110.221	1090.501	114.0915	121.2466
gene-AT1G	2.680883	2.598543	2.732934	2.063098	3.079687	3.237042	1.138306	1.143113
gene-AT3G	0.294262	0.428624	0.234319	0.69941	0.318636	0.465546	0.347472	0.424812
gene-AT2G	10.89962	10.48367	10.10915	10.62071	10.61585	9.661635	3.527612	4.452793
gene-AT4G	1.282145	1.84957	1.235714	2.073079	1.906824	1.619352	3.159401	3.444613
gene-AT1G	10.78483	9.038346	10.94813	15.01324	16.20244	14.00202	37.09533	40.60925

gene-AT3G	6.406419	7.735235	7.694625	23.91989	22.69726	21.28099	5.74387	6.994976
gene-AT1G	1.305407	1.781144	1.158281	1.665938	2.163029	2.049953	4.667939	4.909123
gene-AT3G	1.799366	1.802503	2.237624	2.718995	3.34477	2.273646	0.858234	0.920601
gene-AT2G	5.829981	5.591131	4.894482	7.824019	7.438735	5.537009	2.067193	2.424096
gene-AT1G	1.519386	0.78267	1.116455	12.4488	12.96973	13.06096	0.531372	0.378647
gene-AT5G	28.1513	25.07418	25.31142	49.06883	46.63063	45.04916	22.07545	21.09655
gene-AT1G	6.756293	7.053158	8.760388	8.177592	7.748502	7.527807	15.34991	16.80047
gene-AT2G	15.78289	13.99072	13.54821	16.75911	17.75909	18.52279	1.554174	1.275546
gene-AT1G	6.8382	9.17781	7.941092	4.912745	4.055539	4.962587	12.09219	12.75162
gene-AT5G	14.01804	12.79544	14.3965	10.02429	8.507653	9.403749	20.40135	21.15544
gene-AT2G	8.560434	6.494693	7.865432	6.857176	6.872324	7.035651	2.027143	2.172195
gene-AT3G	0.608592	1.77989	1.174928	0.57381	0.639856	0.457634	1.132855	0.735329
gene-AT4G	27.71326	31.65694	27.25802	22.89208	22.32975	22.99654	61.87619	64.75648
gene-AT3G	0.102745	0.016384	0.037568	0	0	0.018293	0.012403	0
gene-AT2G	19.26995	16.27848	17.64932	35.90937	37.73089	35.01939	19.20075	19.51883
gene-AT3G	1.090693	0.809594	1.049806	0.930335	0.857511	0.86282	0.474591	0.595304
gene-AT3G	20.14619	18.61637	18.3986	16.60239	20.18213	18.18336	17.55158	18.41542
gene-AT1G	0.755624	0.641783	0.803585	2.694064	4.023667	2.503117	0.25548	0.359157
gene-AT4G	75.47845	79.14259	77.23003	62.45441	63.17981	63.21207	28.95741	31.74808
gene-AT3G	5.454851	6.202932	5.952011	8.383873	9.380869	9.095446	0.985025	0.982916
gene-AT5G	1.379768	2.077209	1.985328	4.531968	5.19701	4.136665	0.455583	0.64364
gene-AT1G	6.633998	7.18053	7.012459	4.306305	4.469858	4.387823	12.41119	12.91273
gene-AT3G	5.688715	4.761773	4.919133	8.59071	9.364154	9.059702	3.241933	2.620794
gene-AT5G	27.82048	29.76901	28.81067	30.68749	28.68535	29.18404	39.83752	42.27956
gene-AT3G	21.14811	22.11425	22.95809	17.77328	16.20021	16.38644	28.24286	29.0679
gene-AT3G	30.1083	29.47834	30.13974	21.51972	19.94227	18.02644	45.09788	43.33725
gene-AT5G	68.56655	68.61889	74.06845	99.37408	103.8661	102.6708	20.65367	19.82435
gene-AT4G	0.711734	1.395465	0.774232	2.410572	2.551976	2.808809	0.641561	0.593401
gene-AT2G	46.80319	53.44196	45.78379	31.95592	33.69274	30.89183	94.63889	97.03712
gene-AT2G	2.650536	3.106047	1.979181	1.4334	2.058274	1.509794	1.260166	0.747561
gene-AT2G	14.26366	13.87542	14.57136	21.56811	20.27008	18.57716	8.062356	8.394316
gene-AT5G	2.480762	1.478941	1.863637	5.953175	5.40775	5.613973	0.200484	0.111918
gene-AT3G	2.830074	2.480182	3.52443	5.699786	5.126854	5.429764	3.041017	2.825779
gene-AT1G	381.0906	371.5154	386.009	248.154	250.312	245.0122	611.4453	688.5524
gene-AT5G	4.283577	4.721292	4.940982	7.346158	4.685049	5.745799	2.193691	2.665539
gene-AT2G	20.24079	18.87594	18.60816	14.96095	12.91073	13.43547	28.23652	28.11993
gene-AT1G	19.77602	20.49506	16.43267	5.244093	5.780072	5.548307	1.469058	1.426961
gene-AT5G	149.6086	146.7202	145.587	124.5799	121.3101	124.2458	172.8736	183.0954
gene-AT1G	53.9345	48.87503	54.04558	36.44122	33.07284	32.67359	11.68171	13.63697
gene-AT3G	0.385346	0.227611	0.363555	0.606597	0.556871	0.549394	0.194853	0.228968
gene-AT3G	12.16569	11.78792	13.81563	9.495156	10.6654	9.983938	16.84936	17.95486
gene-AT5G	0.962916	1.090318	1.214181	0.286343	0.781929	0.214227	2.723842	3.172559
gene-AT2G	8.064589	7.462563	7.471879	18.18687	18.98434	17.11185	9.442936	7.957205
gene-AT1G	0.664022	1.010388	0.375178	1.663983	1.392357	1.417205	0.284009	0.957357
gene-AT1G	1.169871	1.370638	1.310296	1.369147	1.385415	1.715609	0.846681	0.891975
gene-AT2G	4.668354	4.693282	5.412045	3.588746	3.822991	4.183856	1.147327	0.683261
gene-AT1G	7.410468	7.396896	7.71334	3.721254	3.133025	4.560285	10.57717	11.26379
gene-AT5G	0.280483	0.357731	0.259342	1.581735	1.281083	1.207614	0.301009	0.292248
gene-AT1G	1.055899	1.717798	1.92788	2.848045	2.386469	2.50787	2.052268	2.257
gene-AT4G	9.311805	8.114562	9.049654	5.07291	5.024651	6.125733	4.673956	3.624522
gene-AT2G	4.369624	4.400407	3.803391	73.53896	69.662	68.56002	1.230456	1.169608

gene-AT3G	2.860541	2.937441	3.727502	7.404472	7.846032	6.952876	1.096304	1.140914
gene-AT3G	2.544488	2.425306	2.267202	1.589868	1.438687	1.512934	3.094396	2.758844
gene-AT5G	25.11477	24.33627	23.29113	15.55654	16.99667	15.97453	36.10863	37.1108
gene-AT1G	135.7807	132.133	125.6982	78.80257	76.03391	76.44577	205.4542	211.0344
gene-AT1G	9.826927	9.354746	8.050096	9.481803	9.646276	9.44209	14.9694	14.03966
gene-AT1G	2.354473	2.481408	2.640704	0.452679	0.792899	0.677582	0.636778	0.177136
gene-AT1G	12.35092	13.42731	13.48005	9.905963	11.89506	10.76341	21.26464	20.95751
gene-AT1G	5.669528	5.89545	5.779503	6.516454	8.394099	7.61746	2.057058	2.307128
gene-AT3G	11.25596	11.01531	11.28906	6.271251	4.697919	5.54857	12.19702	12.76998
gene-AT5G	9.155479	6.897393	8.572341	7.298118	5.788854	9.43024	12.98812	12.88575
gene-AT4G	5.470828	4.828885	3.787633	5.371214	7.261968	5.792879	5.299867	4.654138
gene-AT5G	133.3582	126.8411	134.7997	93.4037	92.96014	93.6295	157.0809	171.8992
gene-AT2G	11.608	11.223	10.32749	9.193072	6.868686	8.984168	22.7672	22.6447
gene-AT5G	2.687858	2.428743	2.397159	3.206803	1.78596	1.405808	1.758867	1.474129
gene-AT5G	2.01337	1.613032	1.769968	1.089961	1.087099	1.647641	1.044478	0.498228
gene-AT5G	8.019319	10.58171	7.894876	8.736101	9.87795	11.47919	3.773741	3.903109
gene-AT2G	9.482204	11.59817	9.901091	1.506723	1.767059	1.141509	1.329277	1.588462
gene-AT5G	33.5584	32.01524	35.86174	36.09057	39.33296	38.88273	15.77734	16.58477
gene-AT2G	22.71315	21.46619	23.0874	23.38375	24.21825	24.00908	19.12763	20.26309
gene-AT2G	10.51802	8.918555	9.883615	6.072909	6.474349	5.256877	12.589	12.26832
gene-AT4G	15.2851	16.18626	15.7445	20.34009	19.44678	19.81082	7.113307	7.575938
gene-AT3G	16.70762	18.96171	17.24417	19.4659	21.67338	20.96274	25.95138	25.57643
gene-AT2G	21.22374	20.84548	19.94177	4.356561	5.89081	3.696993	7.173435	6.771453
gene-AT1G	0.496836	0.125997	0.271147	0.581836	1.388938	1.192611	0.141993	0.274585
gene-AT1G	1.79831	1.62086	1.866844	1.309082	0.845551	1.111612	0.208741	0.283152
gene-AT2G	6.38484	5.797029	5.413773	5.535948	5.798356	6.125505	8.054538	10.54405
gene-AT4G	41.37641	44.34379	42.57544	36.58665	34.965	37.36703	71.91627	73.70951
gene-AT1G	5.322651	5.628993	5.893487	11.76448	10.62134	12.9129	3.300913	3.016384
gene-AT3G	410.7648	427.0005	428.2626	933.6307	924.5546	908.1302	171.4598	170.3311
gene-AT1G	79.87553	76.30797	78.89668	89.98515	91.58534	92.98814	57.58024	60.06397
gene-AT4G	31.01344	29.28621	27.86306	10.13427	9.401732	10.19495	53.91333	55.83609
gene-AT5G	1.875935	1.756825	1.013528	1.420239	1.334116	1.583908	1.622317	1.996017
gene-AT2G	52.16046	50.37782	51.59317	33.83877	32.35515	32.16161	71.6978	70.39195
gene-AT2G	1.030736	1.039623	1.133532	2.178087	1.844025	1.701001	0.514728	0.521522
gene-AT5G	6.665188	7.896614	7.745063	6.350874	7.4099	6.49581	6.635672	7.98278
gene-AT4G	7.569067	6.798556	7.259971	2.544301	2.322735	3.029849	2.34963	3.919367
gene-AT2G	25.74985	27.79289	26.4382	15.68792	14.48346	16.85677	37.62732	36.72392
gene-AT4G	1.193562	0.750854	1.634367	2.527179	2.616907	3.102928	0.898759	1.399132
gene-AT4G	1.363779	0.559135	1.423862	1.788068	1.068098	1.790018	0.68356	0.860517
gene-AT5G	10.22152	9.234749	9.857311	8.421202	10.28348	9.942181	15.08007	15.01423
gene-AT3G	15.76664	13.39625	14.09489	14.76757	17.32626	16.42976	5.553575	6.838142
gene-AT5G	50.17503	46.58512	46.13504	32.23372	35.85374	33.17121	70.06474	76.27936
gene-AT5G	27.09059	25.0249	28.95212	3.18468	2.998603	3.073925	2.18185	2.042928
gene-AT4G	0.827674	0.423384	0.639	0.566532	0.720555	0.512358	1.032189	0.981625
gene-AT4G	0.411878	0.897444	0.667663	1.185614	1.666212	1.887723	1.101832	1.007971
gene-AT1G	40.95789	38.82112	40.37698	36.09975	34.06818	34.72329	20.86142	20.72909
gene-AT3G	5.637741	5.458293	5.564072	3.860063	3.058897	3.855581	11.6211	11.36042
gene-AT4G	0.897354	0.911062	0.986274	0.872543	0.781981	1.092493	1.219237	1.583597
gene-AT1G	1.113774	0.609717	0.631038	0.748801	0.93198	0.713892	1.098252	0.967525
gene-AT1G	233.255	241.5202	243.8975	143.4987	147.9239	144.112	599.1779	565.5588
gene-AT3G	26.19955	25.32009	26.63589	43.37196	38.37975	39.00187	11.71198	11.86692

gene-AT1G	0.829648	0.833753	0.218097	2.400964	1.857455	1.450362	0.804439	0.993562
gene-AT3G	6.697126	6.947236	6.750364	16.38896	15.67029	15.57443	3.401227	3.92232
gene-AT3G	2.227244	1.397942	1.618217	0.759951	1.347469	1.251886	0.865545	0.527119
gene-AT4G	1.46695	1.260745	0.79829	2.046471	1.894202	1.854588	0.134929	0.476777
gene-AT3G	36.78415	37.14751	36.33726	23.39108	22.3324	22.63995	56.46348	55.87001
gene-AT4G	5.74565	5.55042	6.180404	10.79382	9.554465	10.17832	3.461808	3.17204
gene-AT4G	29.21934	27.32617	27.63674	18.16021	16.56649	17.2042	10.68613	11.01034
gene-AT4G	1.439458	1.573438	1.457719	5.748868	5.032801	5.202483	0.530486	0.858233
gene-AT3G	9.363618	9.222504	8.790816	11.91688	8.637825	8.768587	13.15157	14.72691
gene-AT4G	47.0037	49.91544	51.07049	109.1667	111.9119	108.5821	1.316011	1.315264
gene-AT3G	0	0	0	0.902847	0.526636	1.187158	0	0
gene-AT3G	2.779615	1.419491	1.620743	3.135245	3.279315	3.962946	3.893594	3.382411
gene-AT4G	83.43389	79.68676	84.44329	75.4531	75.45841	76.08857	45.28389	46.8219
gene-AT5G	7.68247	7.331735	6.946035	7.675377	6.262842	7.401314	0.963189	0.634517
gene-AT5G	7.763586	8.022735	8.179121	8.474839	8.860598	9.711332	12.88249	12.07816
gene-AT1G	12.32562	12.71547	12.70303	12.46699	10.53968	11.66776	4.514207	3.926633
gene-AT4G	11.32408	9.506237	10.78512	12.08467	11.71662	12.18847	20.7032	23.8224
gene-AT3G	27.04743	25.47054	26.68173	26.83977	24.17169	25.11175	43.0619	43.50334
gene-AT1G	28.75161	32.39543	29.91001	22.9856	23.30724	22.70726	24.44467	25.21414
gene-AT1G	106.199	106.9451	108.8191	64.60519	63.30602	64.58609	149.3872	153.9064
gene-AT4G	15.23977	13.97347	14.29939	12.07115	12.36607	10.43921	25.58825	23.00583
gene-AT1G	1574.286	1663.942	1575.639	1001.54	1010.408	1030.54	2395.009	2446.838
gene-AT1G	46.22432	48.66866	48.30343	33.46022	35.07708	34.39763	100.4852	97.5857
gene-AT4G	9.70495	9.508144	8.48215	8.499456	6.860389	8.576902	13.46558	13.36011
gene-AT2G	24.20496	23.39358	23.46699	16.4646	17.15088	17.52247	9.043679	9.279839
gene-AT3G	86.87585	85.02942	84.91542	39.00473	41.54361	38.17347	130.4282	129.1701
gene-AT3G	432.1923	416.7491	440.7573	731.3809	727.8406	724.8016	369.8682	395.4357
gene-AT4G	0.287646	0	0.203148	1.029292	0.748622	1.01129	0.060942	0.195305
gene-AT4G	30.69468	31.52322	34.65791	25.77269	25.74173	24.57475	69.54653	75.88188
gene-AT5G	0.897689	0.913583	0.823051	1.261544	1.193308	1.2223	0.794847	0.545309
gene-AT5G	4.306959	3.923824	4.405196	4.383218	3.638952	4.016768	7.987324	6.710113
gene-AT1G	10.44669	9.597002	8.716453	4.287318	4.870074	3.997648	14.79648	14.8786
gene-AT5G	16.64854	16.1376	16.41932	25.6601	26.70507	27.34383	12.7756	12.14835
gene-AT1G	1.513609	1.780564	1.908765	3.424628	3.111727	3.567909	0.249596	0.430894
gene-AT4G	17.19415	16.88249	17.42164	29.87555	30.98128	32.09816	25.00613	25.41951
gene-AT2G	32.90063	36.19082	36.64888	30.58414	28.25747	27.89789	53.80358	52.01157
gene-AT1G	15.19326	17.14129	16.29733	14.98211	13.67029	13.98045	28.06579	28.99234
gene-AT2G	0.564003	0.377048	0.396975	0.344687	0.768497	0.570068	0.248838	0.285111
gene-AT1G	0.634945	0.673163	0.857771	2.446734	3.012878	3.237659	0.080227	0.19248
gene-AT4G	13.62596	14.54902	14.62168	11.47833	10.2875	11.78822	4.476042	4.186757
gene-AT2G	10.55695	10.89545	12.29208	9.751445	9.695703	10.00001	17.02289	18.39595
gene-AT2G	18.72278	18.43763	18.6503	10.28745	9.845953	9.001573	39.01122	39.61299
gene-AT2G	4.5611	4.234782	5.738232	5.294507	5.719109	4.787923	1.491683	1.243526
gene-AT1G	12.64963	13.16572	11.80169	10.04333	10.15626	10.03677	23.36234	24.24043
gene-AT3G	45.88563	45.95719	47.21098	48.02427	46.48501	48.05376	60.19715	62.80509
gene-AT4G	16.37156	14.60889	16.74839	14.14128	14.9883	14.59796	21.74715	24.39171
gene-AT2G	26.15011	23.83366	26.60787	18.88708	17.91289	16.5114	30.05082	31.10887
gene-AT4G	27.40263	31.72654	28.26101	43.02831	42.51562	43.90035	27.09502	28.88415
gene-AT1G	8.34154	5.736607	5.529808	15.88784	19.65726	20.74032	7.254512	9.595248
gene-AT5G	19.57844	20.83861	21.7374	6.960842	8.554617	7.903272	18.80343	20.77255
gene-AT1G	1.491584	1.095323	1.006554	0.751925	0.802446	0.697142	0.770443	0.803121

gene-AT1G	0	0.349071	0.221445	1.695619	1.779786	1.556787	0.338672	0.21915
gene-AT1G	2.936343	2.306799	3.435012	12.59869	16.22688	13.01533	5.201858	4.498335
gene-AT3G	0.82126	0.742309	1.341682	0.377354	0.678568	0.359389	0.982666	1.189661
gene-AT1G	6.585778	7.020622	5.68139	4.663254	6.119452	4.39541	8.403629	6.241589
gene-AT5G	20.19709	19.52243	21.69352	20.37285	19.91192	20.27706	16.1789	17.61555
gene-AT3G	23.92761	23.23355	25.27991	18.23644	17.29679	17.09715	6.407842	5.16802
gene-AT5G	23.04895	22.66569	23.99637	22.11578	23.11326	20.91836	48.2689	52.02964
gene-AT4G	8.310115	9.624139	7.809451	13.17277	12.61443	12.30608	4.746308	3.914645
gene-AT1G	5.962229	5.406171	6.391753	7.688933	5.791709	6.985834	12.91531	11.43065
gene-AT1G	18.52441	18.53295	18.83957	18.43473	14.98298	15.5453	26.77472	25.86798
gene-AT4G	2.594407	2.473269	2.797236	1.995448	1.251539	1.383993	2.060195	2.408722
gene-AT4G	288.6469	286.5872	299.5624	336.009	312.4386	325.9283	225.4308	231.1834
gene-AT2G	0.342878	0.759645	0.402337	0.573702	0.414984	0.413774	0.042521	0.167492
gene-AT1G	53.55099	57.38368	58.79481	50.77058	51.96124	49.03377	16.45103	17.30281
gene-AT3G	3.618221	3.848505	3.114555	0.616795	0.584181	0.812186	0.295471	0.942133
gene-AT5G	13.67719	11.98399	12.01169	4.661427	5.184934	3.759874	2.831277	2.950398
gene-AT3G	5.772081	5.680602	4.287033	11.48725	11.54277	10.63107	6.780327	6.08525
gene-AT3G	3.845182	4.074141	3.702985	4.32978	5.481492	4.54982	8.327723	10.66747
gene-AT1G	0.889757	1.024822	1.188933	2.891269	2.837401	2.667431	0.180769	0.223988
gene-AT1G	9.563892	8.348323	9.145242	1.395845	1.634309	1.992183	2.328608	2.119272
gene-AT3G	1.040966	1.301554	1.242763	0.736742	0.42559	0.51843	3.773256	4.264272
gene-AT2G	220.1876	207.3732	216.4139	87.56288	88.60577	83.85385	333.6588	340.5932
gene-AT3G	2.715595	2.519457	2.730108	2.507161	2.32127	1.920317	2.457572	2.756889
gene-AT4G	3.079973	4.070466	4.433005	4.345571	3.474053	4.209195	1.401781	1.154535
gene-AT1G	2.309064	1.869135	1.401881	2.360057	2.635139	2.566949	0.343187	0.673733
gene-AT5G	1.031089	0.752712	0.531667	1.475843	2.142348	1.623893	1.360127	1.332007
gene-AT4G	2.772752	2.362718	2.803198	1.392687	1.311397	1.788661	2.565535	2.744882
gene-AT5G	33.40163	32.20657	29.93764	25.92724	25.29271	24.90653	40.71372	45.58242
gene-AT4G	0.074141	0.235822	0.147848	0.235933	0.143838	0.35058	0	0
gene-AT1G	1.08094	1.250289	0.92457	3.772202	4.629195	3.42164	0.72166	1.236516
gene-AT5G	46.76877	40.00931	41.16054	29.20913	27.33616	31.3754	66.51466	62.83793
gene-AT3G	2.49069	2.302696	1.531493	2.053974	1.199688	1.798382	5.505337	4.298483
gene-AT3G	1837.017	1839.057	1896.318	971.1172	954.486	936.9833	2397.781	2504.746
gene-AT2G	0.693259	0.545497	0.924237	1.334251	1.324662	1.006813	0.028432	0.068673
gene-AT5G	12.89515	12.40161	13.25682	16.56907	15.42769	15.35636	8.17183	8.044386
gene-AT3G	3.584611	3.83746	4.408724	3.160127	3.539407	3.119973	4.679148	5.174398
gene-AT4G	980.9431	945.2828	984.46	513.1685	496.4928	485.1042	1470.282	1513.733
gene-AT2G	41.90078	40.15577	42.85817	36.98334	35.85069	38.32406	17.15911	18.54407
gene-AT1G	2.292571	1.935528	2.463985	1.192153	1.28595	2.15434	0.889741	0.978225
gene-AT4G	72.27196	64.71993	62.99837	19.86684	21.31961	19.07556	17.78557	20.58519
gene-AT3G	8.764883	12.27997	8.951984	10.00191	8.243464	11.95052	4.47457	3.77187
gene-AT5G	8.993054	7.884893	8.16049	2.73482	2.698867	2.268748	2.571279	2.625993
gene-AT3G	1.189539	0.669503	0.73335	1.879978	2.076255	1.903869	2.18259	1.619629
gene-AT2G	4.086993	2.938818	3.30755	3.832951	2.821915	3.23648	1.117238	1.794938
gene-AT3G	0.607368	0.584436	0.394816	0.03854	0	0	0.162058	0.220228
gene-AT5G	22.3332	19.43752	20.60531	17.04502	16.17809	16.0307	1.768047	2.050918
gene-AT4G	0.994224	0.892329	1.033538	1.224939	1.544943	1.416861	1.953282	2.248381
gene-AT4G	24.30089	22.04947	24.48945	21.84383	21.62953	19.99031	12.73126	12.34869
gene-AT2G	16.21278	15.50783	15.20358	27.8288	30.31892	26.66519	11.11984	11.60356
gene-AT5G	2.800323	2.065611	2.718271	1.228866	0.968759	1.429658	0.64977	0.906746
gene-AT2G	2.771309	1.919936	2.817902	1.139923	2.17076	1.435204	3.498115	2.843256

gene-AT5G	4.000166	3.93822	3.947038	5.439318	5.181755	5.42777	4.3418	5.664337
gene-AT2G	1124.328	1221.46	1163.022	2118.61	2297.671	2352.898	628.2675	570.5362
gene-AT4G	4.895081	6.117172	4.591682	21.39189	22.95001	23.00085	7.266231	7.326406
gene-AT1G	2.657399	3.360738	2.998587	2.893228	2.896225	2.602834	4.142457	4.400187
gene-AT4G	2.159594	2.182626	2.56406	1.946376	2.18632	1.671292	1.301367	1.013384
gene-AT1G	3.416344	3.075057	2.94181	2.129667	2.318879	2.559432	2.458786	2.407925
gene-AT3G	6.714324	6.481718	7.053911	11.58607	9.586233	9.245878	3.630418	3.315909
gene-AT3G	1.810014	1.686355	1.507836	1.985857	2.703612	2.564061	1.152891	1.330429
gene-AT5G	5.825637	6.424632	6.819496	5.545509	5.780984	5.793659	15.99485	16.66805
gene-AT1G	12.02519	11.46225	11.86229	10.73471	11.0061	11.35895	15.2953	17.39279
gene-AT1G	0.097492	0	0.057553	0.1852	0.223281	0.246771	0.084812	0.159944
gene-AT1G	8.848786	9.078093	8.879932	8.571914	8.362695	9.009358	16.17831	16.09662
gene-AT1G	0.809803	0.785679	0.781789	1.334008	1.147314	1.111604	1.929094	1.967015
gene-AT1G	2.958915	3.313138	2.945282	4.663752	3.795919	4.595731	0.492624	0.241677
gene-AT5G	16.60548	16.45912	16.78634	37.12794	38.08562	39.49855	11.03449	8.242695
gene-AT4G	1.587461	1.082076	1.819832	1.462022	1.183482	1.203251	0.736922	0.819285
gene-AT1G	6.114239	6.797451	7.048489	0.526136	1.090151	0.859999	9.756563	8.874401
gene-AT1G	0.267915	0.691652	0.780833	1.598451	2.04908	1.844496	2.207423	2.211203
gene-AT4G	3.797205	3.880356	4.034802	13.94915	14.24318	14.32766	2.919935	1.25584
gene-AT1G	40.60271	38.51034	36.72062	19.63732	19.2286	20.15924	79.07043	81.10443
gene-AT1G	14.53131	15.15507	15.59411	19.25033	17.45444	16.96163	14.57583	13.30676
gene-AT3G	0	0.153145	0	0.126881	0.300309	0.332319	0	0
gene-AT3G	0.214535	0.435378	0.516532	0.229523	0.459307	0.973681	0.837711	0.180535
gene-AT3G	0.471578	0.587527	0.627271	1.232981	0.969084	0.866419	0.191768	0.108943
gene-AT4G	257.3517	250.6569	255.2951	400.0959	391.6932	391.0227	186.4134	184.8047
gene-AT1G	3.237619	3.62141	3.631466	15.94708	18.30717	18.37104	3.037486	2.288339
gene-AT4G	48.4661	55.98063	54.10564	84.10078	86.76933	87.97195	76.66337	79.83589
gene-AT3G	1.594332	1.35503	1.657008	0.609185	0.472097	0.472581	0.758558	0.73575
gene-AT1G	3.220111	2.832233	3.520576	3.880619	3.957013	3.9343	6.098714	6.343664
gene-AT1G	1.143152	1.432696	1.57612	2.26023	3.956724	2.826151	1.21414	1.167984
gene-AT1G	1.226207	1.271169	0.802054	1.821555	2.969004	1.912098	1.82019	2.799305
gene-AT3G	1.810241	1.568406	1.806516	1.538133	1.895243	2.076098	3.124466	2.912186
gene-AT2G	1.669433	2.249591	2.287688	0.141759	0.170418	0.147308	11.60145	11.82668
gene-AT5G	5.675688	4.91294	4.643304	32.47511	34.57922	32.72514	2.818165	2.598687
gene-AT5G	1.010364	1.683064	0.937183	1.024328	1.246497	1.094802	1.963641	3.477122
gene-AT2G	3.306759	3.085479	2.234795	0.712745	1.207082	0.632705	7.851119	7.761296
gene-AT5G	1.872263	2.060019	1.658017	2.520875	2.821735	2.02491	1.086215	1.106298
gene-AT4G	38.5196	42.45691	42.3492	28.10988	26.60416	25.76739	86.74896	86.10257
gene-AT1G	24.85741	26.32469	25.0128	26.80183	29.82936	28.51394	18.98256	18.38649
gene-AT5G	6.279085	5.267426	5.016059	3.315682	3.185518	3.986867	1.849394	1.712399
gene-AT3G	64.47622	63.64138	63.18975	41.52989	42.03611	39.84516	105.9422	103.9744
gene-AT3G	5.529569	7.72644	7.054955	9.991936	9.835304	9.718431	12.63442	15.16473
gene-AT5G	19.99656	21.58825	17.66531	6.011545	6.499573	5.877896	25.46918	25.20499
gene-AT5G	0.839564	1.349897	0.80044	2.008065	2.875782	2.210365	0.330799	0.364536
gene-AT2G	49.97168	52.02276	52.34406	40.75614	42.71926	44.18288	83.9642	89.43503
gene-AT1G	3.262735	3.688771	3.398703	0.203416	0.32633	0.40336	0.114058	0
gene-AT3G	1.843854	1.583851	2.069502	1.770019	1.937601	1.652137	2.289841	2.505067
gene-AT1G	16.81691	15.8495	17.00563	29.38799	31.0862	25.8042	5.035357	5.034104
gene-AT2G	2.216596	1.585621	2.177155	22.06829	26.39672	27.28427	2.131697	2.331692
gene-AT1G	90.73911	98.72126	94.05033	63.55839	57.86714	61.42548	162.5133	179.3933
gene-AT3G	26.59568	26.60492	26.72004	10.89868	11.19325	11.79047	8.864228	9.46881

gene-AT3G	3.820285	5.183801	3.973419	2.835328	3.645607	3.719323	5.281335	4.732679
gene-AT2G	0.797392	0.820166	0.555169	1.431307	1.149203	1.064557	0.128492	0.244415
gene-AT3G	6.67244	7.667679	6.160633	11.60556	11.97032	11.63416	1.945489	2.350089
gene-AT3G	1.198379	1.538961	1.29136	1.208484	1.393188	0.901051	1.177752	1.841898
gene-AT5G	37.84444	35.03422	37.58675	24.84027	25.30017	26.79113	63.87978	71.75018
gene-AT4G	8.333551	9.461385	8.325555	13.09876	13.02019	12.03881	1.914769	2.104558
gene-AT2G	7.373713	6.203033	6.91817	4.764371	4.393525	2.453395	0	0.586876
Arabidopsi	10.14259	11.4498	10.61681	7.898043	7.543725	7.362461	4.274356	4.445814
gene-AT2G	7.7129	9.633612	8.051412	5.191727	5.37907	5.420934	1.223278	0.916839
gene-AT4G	18.51632	22.17307	16.35778	13.11505	14.47277	13.02824	26.99083	28.93586
gene-AT5G	14.96568	13.75968	14.44863	33.25763	32.48985	32.67479	17.48007	16.93202
gene-AT1G	0.997575	1.091544	1.125243	1.402336	1.344994	1.589802	1.250577	0.870613
gene-AT2G	0.561121	0.129395	0.147735	1.206556	1.780806	1.627808	0.087065	0.2289
gene-AT5G	133.4348	132.2702	138.6463	179.3518	175.6709	174.0821	80.57302	80.40783
gene-AT1G	0.801579	1.25418	0.82831	1.871125	2.020792	2.381154	0.849679	0.856447
gene-AT5G	6.161226	6.230721	6.725851	8.227328	7.409712	6.9394	2.169018	2.429525
gene-AT2G	0.044957	0.243687	0.052085	1.453462	0.696352	1.095892	0.09499	0.277841
gene-AT5G	4.398287	4.03646	4.36376	2.701183	2.367041	2.652033	1.382227	1.729808
gene-AT4G	22.78893	23.29653	24.26586	21.60555	20.29838	21.6761	25.70071	26.50749
gene-AT1G	0.758592	1.638008	1.160619	2.546389	2.48274	2.114462	1.135221	0.698815
gene-AT3G	27.27269	27.20482	27.27656	34.55403	35.30827	33.41985	21.10017	20.18862
gene-AT5G	4.629238	4.536159	4.180758	6.185098	5.864282	5.889686	5.685814	7.137032
gene-AT1G	3.963775	3.84592	2.169265	1.195614	2.482897	1.535637	1.31077	0.596085
gene-AT4G	24.09696	24.485	23.62045	22.24537	19.67831	21.15397	25.91093	27.5455
gene-AT5G	2.596188	2.38441	2.693386	1.07681	0.626846	0.716563	0.453812	0.284463
gene-AT5G	1.147207	0.934163	0.884806	1.108028	1.108896	1.204211	2.43626	2.74725
gene-AT1G	11.56474	11.12493	10.66196	5.091682	5.613607	5.728484	3.943803	4.724367
gene-AT3G	2.602398	2.951412	2.685295	4.403612	4.061842	3.303481	8.861417	10.51411
gene-AT1G	2.290595	2.607855	2.089278	1.965618	2.048048	1.558498	0.760413	0.634779
gene-AT4G	0.620085	0.657364	0.631024	0.761433	0.749451	0.629445	0.160954	0.139914
gene-AT4G	0.542233	0.715333	1.531103	2.158993	2.935415	1.584461	0.968047	0.278402
gene-AT5G	2.115207	1.609668	2.041736	2.660797	2.222382	1.653919	2.071614	1.118847
gene-AT4G	142.487	138.5678	139.839	109.7188	112.114	110.7041	86.84545	85.87362
gene-AT1G	1.497473	1.411591	0.980481	7.08463	7.861872	6.374814	2.529486	2.560647
gene-AT1G	8.048662	9.208434	9.927667	16.78028	14.62198	16.3777	4.571966	4.454079
gene-AT5G	1.006087	1.160798	0.759712	1.46187	1.700635	1.552564	2.200836	1.70053
gene-AT5G	1.028432	0.71884	0.416398	2.813405	2.251867	2.531513	0.168611	0
gene-AT3G	0.044052	0	0	0	0.044173	0.059837	0	0.087683
gene-AT5G	3.56475	2.980441	3.496112	2.658629	2.134444	2.972922	8.670007	7.348969
gene-AT5G	46.57493	41.72571	43.08999	94.80624	96.84007	92.85483	36.7196	36.83993
gene-AT2G	28.29365	27.92246	30.02128	23.60547	23.64831	22.89967	31.57402	30.88345
gene-AT5G	46.69354	46.19828	50.10076	60.28698	62.23767	62.5615	26.64363	29.44832
gene-AT5G	9.549235	11.02936	11.66273	11.98886	10.36582	11.11566	23.41637	24.40868
gene-AT5G	1.653665	2.181457	3.262474	2.21336	1.223426	2.054261	4.263684	4.593292
gene-AT2G	13.98009	14.31462	13.15391	20.55305	17.82074	17.7696	4.770603	6.145845
gene-AT1G	4.381741	3.163738	2.855338	9.454375	9.075354	8.510508	0.636132	0.736872
gene-AT1G	0.245297	0.473056	0.207046	2.053345	2.146736	2.371665	0.421226	0.067135
gene-AT5G	95.87131	89.35082	99.30299	40.68955	42.09724	39.75856	202.3447	206.2775
gene-AT3G	109.8712	106.1354	104.6511	109.0762	100.5718	104.4513	208.1127	208.9966
gene-AT1G	2.117207	1.986404	3.287835	0.811783	1.236156	0.848876	2.380975	2.096526
gene-AT5G	6.87664	7.04763	8.147103	5.024535	7.03453	6.406657	8.873063	7.9072

gene-AT1G	2.022686	2.662887	1.494527	1.923377	2.08759	1.818788	3.317829	2.706984
gene-AT1G	10.40003	8.788158	10.33501	10.34895	10.06065	8.29542	12.16603	13.37355
gene-AT1G	4.776996	4.747343	4.898999	6.356846	5.877671	5.318536	5.141747	5.744532
gene-AT3G	9.456326	10.25672	10.03009	2.287657	2.513862	1.967839	2.008102	2.13774
gene-AT1G	2.44091	2.164446	1.573653	0.622801	0.399741	0.594021	0.806139	0.647042
gene-AT3G	21.29604	22.9874	23.19375	21.7128	19.67203	21.70364	17.21153	16.98456
gene-AT1G	30.5953	28.55477	29.69416	22.99226	21.99584	22.5186	17.52453	19.01094
gene-AT5G	164.0031	161.564	160.6622	118.4607	121.5425	123.1534	79.59632	83.06317
gene-AT3G	0.246248	0.237621	0.105664	1.215789	1.042904	1.086946	0.871587	0.111322
gene-AT1G	0.34697	0.121372	0.409731	0.377954	0.56919	0.104802	0.395253	0.958286
gene-AT1G	0.302491	0.524989	0.251646	1.058179	0.979348	0.896487	0.135332	0.262153
gene-AT5G	0.9059	0.542758	0.377026	0.485473	0.35919	0.758223	0.421155	0.343163
gene-AT3G	3.061699	2.771902	3.157476	3.891922	4.771312	4.748965	3.740046	4.11152
gene-AT1G	23.20997	23.12292	23.57878	31.76813	30.14443	29.89499	6.149627	6.911148
gene-AT5G	5.776053	6.290989	5.712705	2.035028	1.46062	2.253909	1.603187	2.734974
gene-AT2G	7.792184	7.973078	9.090479	10.23015	9.475169	10.112	5.51412	4.232025
gene-AT3G	5.551401	5.0742	6.254935	3.380572	3.348948	3.410803	1.548704	1.742199
gene-AT5G	10.476	10.12876	10.27523	7.497433	7.587708	7.250056	14.57375	14.10129
gene-AT5G	8.199107	7.994794	7.849226	6.490469	6.554461	6.388761	4.705749	6.063375
gene-AT3G	693.824	672.274	691.3985	817.1644	809.7452	802.042	601.6441	628.4165
gene-AT3G	2.502083	2.041135	2.786975	1.844072	1.495645	1.959822	3.27843	2.855103
gene-AT5G	4.390916	4.010363	5.276336	5.439538	5.114858	4.919561	0.816346	1.184538
gene-AT1G	0.994169	2.505854	1.350623	0.526816	0.416591	0.484448	1.619351	2.268362
gene-AT1G	3.293113	2.76349	2.598398	1.071381	0.738201	0.80269	2.012084	2.505534
gene-AT1G	3.28824	3.315799	3.580892	1.086759	1.08444	0.944233	0.979308	1.445472
gene-AT1G	1.791252	1.189782	1.357911	1.345107	1.681942	1.022128	0.416414	0.37256
gene-AT3G	0.334567	0.463636	0.306658	0.81216	0.953536	0.972884	0.095219	0.289083
gene-AT1G	40.47346	37.68958	39.42291	26.00589	28.13893	26.76862	30.17335	31.13721
gene-AT1G	6.249331	5.823747	6.069094	3.040871	3.660385	2.722178	0.40652	0.172414
gene-AT1G	0.762028	0.864344	0.604115	1.818428	1.296386	1.527589	0.190421	0.107973
gene-AT1G	76.48512	79.11717	79.65662	55.19555	58.24581	55.32483	61.43383	60.42047
gene-AT1G	0.890093	0.823735	0.662641	1.395304	1.293514	1.348526	0.594002	0.755852
gene-AT1G	2.051593	1.753535	2.144454	2.802592	3.020355	2.97105	2.883401	3.262
gene-AT3G	2.092729	2.156288	2.249263	3.453183	2.676239	3.144042	1.48726	1.980496
gene-AT2G	2.097076	2.320457	3.053278	2.977266	3.657105	3.314404	2.139765	1.35401
gene-AT5G	3.130301	2.856004	3.343652	1.777668	2.966754	2.657605	4.111304	3.157937
gene-AT5G	3.901615	3.961481	3.587036	3.090424	3.346863	3.016558	4.975349	5.030278
gene-AT5G	3.247827	4.497549	3.951708	7.406085	7.546949	6.490291	2.034189	2.203134
gene-AT5G	19.267	19.68935	20.57339	14.0792	14.66053	14.461	33.90645	34.08685
gene-AT1G	2.230223	1.704185	2.083605	2.949426	2.661825	2.960675	1.105368	1.356408
gene-AT2G	95.21428	102.1229	93.34726	71.48248	70.95705	68.80828	193.7013	176.9071
gene-AT2G	0.841656	1.053392	0.69786	0.920677	1.199538	0.961341	0.479062	0.374914
gene-AT4G	5.152063	4.136375	4.227882	5.972437	6.84197	5.970992	2.436323	2.26969
gene-AT2G	3.943043	5.253574	4.261355	0.870738	1.69857	1.672007	13.1806	10.80922
gene-AT3G	18.36321	19.66941	17.54358	15.16248	15.26014	15.9168	27.13253	32.18924
gene-AT1G	137.9293	130.0644	146.6379	164.8518	157.5595	151.465	108.9527	109.1669
gene-AT1G	31.51429	32.91486	32.31337	20.05331	20.07134	24.92535	45.4501	47.28869
gene-AT3G	51.08991	46.47235	45.66694	160.9943	159.875	153.272	17.99115	16.67214
gene-AT4G	1.400369	2.394114	3.224331	0.63365	0.690468	0.47984	2.290908	1.89992
gene-AT2G	1.980052	1.822036	2.238644	3.198021	2.65262	3.370443	2.100957	2.996121
gene-AT3G	4.464215	2.724658	3.03226	6.313717	5.826266	4.49539	1.538841	1.292747

gene-AT5G	0.857224	0.662788	1.270672	1.035481	0.925185	0.738276	0.727708	0.93045
gene-AT4G	2.558647	3.377418	2.754381	3.83539	4.125388	3.275143	6.044371	5.677518
gene-AT1G	0.676736	0.741443	0.788883	1.972215	1.595661	1.990927	1.053864	1.170364
gene-AT4G	1.639174	1.030427	0.831932	0.917357	0.8813	0.656272	2.082074	1.849648
gene-AT3G	22.38234	18.64814	17.93535	46.16172	45.98013	46.26897	17.82595	16.62568
gene-AT1G	31.05815	33.06661	31.0951	87.15031	86.17004	90.85333	40.49527	38.43126
gene-AT3G	0.52045	0.779327	0.343057	1.858413	1.791655	2.221445	0.95682	1.265312
gene-AT5G	1.100483	1.076875	1.361717	0.64445	0.599928	1.061728	2.048193	3.08887
gene-AT2G	243.4406	241.9073	244.6799	232.4005	230.9733	222.0745	123.8445	128.7215
gene-AT5G	18.62842	18.98982	18.38726	14.35687	15.11212	12.99961	27.90306	26.80827
gene-AT5G	2.088627	2.454568	3.025604	5.218052	3.778846	5.013587	3.692099	3.673205
gene-AT5G	3.209918	2.569532	2.982648	1.710254	1.234535	1.725898	0.945125	0.821415
gene-AT1G	0.208361	0.417778	0.359137	0.776796	0.791125	0.970316	0.286477	0.271451
gene-AT1G	0	0.339054	0	0.603458	1.020674	0.755339	1.032018	1.083925
Arabidopsi	0.618119	0.765879	0.920534	0.235676	0.58705	0.285483	0.937623	1.174722
Arabidopsi	3.863186	2.565056	2.557884	1.459046	1.838408	1.224376	1.394074	0.885201
gene-AT5G	1.041841	1.339057	1.674119	1.287316	1.048734	1.354638	0.42056	0.620308
gene-AT4G	0.17631	0.348749	0.648905	0.411017	0.445242	0.637268	0.203946	0.377178
gene-AT1G	6.13028	3.38696	4.76465	2.82911	2.66899	2.065222	10.03841	12.37876
gene-AT5G	10.34103	13.75687	12.70991	16.23764	17.01066	15.71062	5.525351	4.745456
gene-AT3G	2.491826	3.290091	2.247258	33.22155	33.91488	30.1974	2.231455	1.870206
gene-AT5G	10.62686	8.91466	8.475427	2.764832	3.815476	3.166679	11.06304	9.886593
gene-AT3G	5.977205	5.207052	5.404884	4.920891	5.826297	4.78859	7.71131	5.915103
gene-AT5G	29.70242	27.66264	28.45563	20.29881	19.91148	20.18521	1.614077	2.376079
gene-AT3G	0.972315	0.796375	1.116138	0.945997	1.488261	1.034322	0.201191	0.249465
gene-AT5G	17.49218	19.53965	16.62033	9.802566	11.27068	9.282943	3.841602	4.112511
gene-AT1G	11.70412	11.755	11.61356	9.843477	8.51568	10.41421	23.06709	24.46538
gene-AT3G	1.474292	1.277336	1.366867	1.191666	1.486846	1.841953	0.342897	0.873319
gene-AT1G	6.133294	4.378919	6.21035	5.688471	6.95764	6.371917	11.92102	12.21256
gene-AT3G	14.54083	12.20257	11.85058	14.95931	15.69849	13.90723	8.310627	8.829505
gene-AT4G	8.560255	7.968248	9.684079	2.631057	3.853046	3.752659	7.248131	6.71326
gene-AT3G	521.0189	518.2161	526.3101	179.4763	177.0753	171.6006	681.9174	702.2395
gene-AT4G	6.04388	7.143271	7.257442	10.55223	10.77989	11.78374	11.37527	13.03793
gene-AT3G	29.03829	29.81353	28.856	34.77135	36.63002	33.44449	15.06305	14.45627
gene-AT3G	115.1035	112.1084	109.3634	71.1413	66.21785	68.00613	201.0217	196.5369
gene-AT5G	0.454265	0.618113	0.360369	1.086529	1.450297	1.360372	0.382238	0.129875
gene-AT5G	1237.749	1217.609	1241.041	826.325	822.4302	818.6679	1816.762	1903.607
gene-AT3G	9.378629	8.162766	8.37514	9.770128	9.035013	8.279655	13.55344	13.58299
gene-AT4G	173.3184	177.0627	175.2199	637.2669	658.5274	659.4076	51.80163	54.64513
gene-AT3G	12.21531	12.6145	10.18194	13.31936	15.09553	15.53882	16.30211	15.00763
gene-AT1G	0.721173	1.153153	1.396547	2.521562	2.004995	1.422082	0.598714	1.223986
gene-AT1G	37.68268	41.32642	42.21076	39.78641	37.27137	37.74711	20.50885	22.11267
gene-AT4G	2.06982	1.571064	1.556609	1.607193	1.997146	2.207375	1.069522	0.760539
gene-AT2G	12.30091	11.70443	12.39248	11.83981	13.75459	11.64266	22.24991	24.36919
gene-AT3G	4.344575	4.007878	4.779713	4.859682	5.02188	5.461334	5.257074	6.025767
gene-AT1G	0.421636	0.301836	0.720562	1.173435	1.454907	1.5089	1.015885	0.619874
gene-AT4G	7.352543	8.353644	7.972309	12.01405	11.3772	12.45541	4.03421	4.111948
gene-AT1G	1.312026	1.5645	1.033853	2.835972	1.271719	1.289333	1.869799	1.645921
gene-AT3G	0.198875	0.285146	0.685764	1.248706	0.969249	1.431608	0.449276	0.597495
gene-AT1G	91.52405	89.8068	91.1666	57.26832	58.18918	59.35939	87.40673	88.38605
gene-AT3G	96.81947	94.27696	96.24715	146.6226	141.1244	145.2034	151.9485	159.8069

gene-AT5G	1.734167	1.574091	1.546575	2.208422	2.041796	2.21408	1.553062	1.65947
gene-AT4G	7.673769	6.760446	7.320251	7.292629	8.337397	8.05739	10.26586	8.249938
gene-AT1G	4.103181	4.770422	4.616758	5.090741	4.007199	4.853798	5.644537	7.097602
gene-AT1G	1115.675	1161.606	1115.054	969.2273	961.3716	999.1694	1134.449	1080.646
gene-AT5G	8.022302	6.952044	7.938923	6.322572	6.759897	7.685315	3.290438	2.447447
gene-AT3G	0.698835	0.524513	0.614185	1.191089	1.037819	1.080351	0.286368	0.351586
gene-AT5G	5.913958	5.812519	6.366498	8.291656	8.719708	8.439713	3.396096	3.019225
gene-AT2G	0.938997	0.891174	0.93388	0.944828	0.531102	0.807058	0.315407	0.392563
gene-AT1G	2.152964	3.137592	2.61945	3.132186	3.95784	2.429973	1.610411	0.845651
gene-AT5G	9.139867	9.288039	9.061311	7.106439	8.196998	7.474182	9.003047	9.218809
gene-AT2G	20.33728	16.91085	18.4448	52.35277	56.65503	51.14605	19.33089	19.43528
gene-AT2G	2.671041	2.758237	2.460371	6.024973	5.168213	6.034298	2.270368	2.263451
gene-AT5G	30.33553	27.64103	28.29062	18.66575	19.08836	17.13891	2.729103	2.379169
gene-AT4G	10.929	10.07341	10.05185	14.27533	13.88307	14.27895	13.66479	15.64325
gene-AT2G	20.27594	20.85106	22.46322	18.57684	18.94105	16.90195	3.168976	2.56188
gene-AT1G	56.10383	61.81625	61.30994	76.16426	82.72951	99.2578	3.616424	2.833172
gene-AT1G	0.283105	0.468498	0.29757	0.790741	1.101852	1.299042	0.04115	0.103555
gene-AT2G	2.955013	3.16096	3.709799	8.050505	8.42058	7.941311	7.334805	7.744752
gene-AT5G	20.27204	19.87285	20.00729	11.433	12.34304	10.55836	29.39876	28.36065
gene-AT5G	23.52684	19.84628	22.10841	79.99243	78.32935	74.78868	30.49661	31.21173
gene-AT5G	229.9598	233.1319	232.1944	140.7447	136.7153	140.5996	139.4679	147.802
gene-AT5G	19.58431	20.18839	21.50781	13.06034	13.27954	11.81427	62.25422	61.93629
gene-AT5G	0.405581	0.499254	0.556917	0.83487	0.99816	0.65919	1.202857	0.94217
gene-AT3G	4.342356	4.357418	4.985608	2.305734	1.911437	2.076385	1.796514	1.909923
gene-AT1G	17.1514	14.81597	17.28031	8.968966	9.06013	9.05907	3.510621	1.897078
gene-AT1G	3.210145	2.49376	3.332705	7.858842	8.870286	9.102605	4.236135	4.462945
gene-AT1G	7.531818	7.758603	7.453339	6.806341	7.157477	6.763382	7.032195	8.259661
gene-AT4G	4.815381	5.520474	4.615345	5.548748	5.34679	5.91678	3.81032	3.833328
gene-AT5G	2.884711	3.050554	3.264447	0.874077	1.123054	1.031386	3.034634	4.701766
gene-AT4G	108.4559	100.4363	105.6452	238.8499	224.7566	233.1352	10.88717	13.09665
gene-AT2G	30.43611	28.80962	26.69566	13.71446	11.51709	12.12066	5.567839	6.404369
gene-AT4G	127.2975	126.0677	127.3725	547.0016	533.6712	536.8235	95.04995	93.23953
gene-AT3G	0.329399	0.089301	0.441342	0.043663	0.084697	0.15422	2.627783	4.013739
gene-AT3G	10.144	10.09691	11.41189	40.09082	39.97695	37.89918	10.41051	10.14632
gene-AT4G	7.049797	7.557999	7.135975	4.13502	3.790362	3.539052	11.47319	10.74435
gene-AT4G	0.822947	0.858384	1.095612	1.98813	2.278301	2.378397	0.573056	0.882761
gene-AT4G	30.23989	27.63597	29.28465	28.15346	25.30105	27.01844	23.76165	24.45986
gene-AT5G	5.044354	5.695024	5.066258	3.819108	3.652447	4.716624	8.829813	9.35203
gene-AT1G	1.496853	2.215993	1.823957	0.433629	0.573826	0.511074	0.829862	0.378679
gene-AT2G	12.66039	12.54036	11.19903	6.884985	6.7455	7.346395	7.234264	7.253645
gene-AT2G	12.32255	12.41388	11.4266	15.98599	14.63534	15.5245	16.21128	9.60877
gene-AT3G	1.373921	1.916467	0.914104	1.07096	0.844664	0.694163	2.746452	3.934439
gene-AT5G	0.579643	0.265317	0.453474	0.517012	0.432992	0.51621	0.134524	0.136253
gene-AT4G	9.775249	11.53569	10.17172	20.7049	21.75081	21.199	9.271795	8.544803
gene-AT5G	2.901791	4.644878	3.293365	4.28003	4.485253	3.179492	1.696642	1.536968
gene-AT4G	31.15685	28.99338	33.79508	29.36355	28.12619	27.36224	40.12516	37.14883
gene-AT4G	20.34685	20.36778	22.34273	17.59392	15.75627	15.15387	8.366341	9.353645
gene-AT5G	13.49916	12.20315	13.90284	4.870124	4.705156	3.792049	2.929751	2.54643
gene-AT1G	16.29384	14.66029	16.55511	18.58786	19.26085	19.08166	11.28064	10.21861
gene-AT3G	19.07133	21.81135	20.04439	12.56999	12.50961	13.51452	6.343275	6.557549
gene-AT3G	8.212046	7.937135	7.064272	15.07722	15.73048	13.8427	3.255266	3.630877

gene-AT1G	1.010757	1.267307	0.793263	1.532152	1.34219	1.202059	0.117561	0.147667
gene-AT5G	0.339399	0.437051	0.319194	0.337484	0.201452	0.271679	0.904715	0.748351
gene-AT1G	0.949221	1.119449	0.904999	0.598644	0.75829	1.05254	0.274806	0.321189
gene-AT5G	0.721982	1.103317	0.562812	1.263785	1.085575	1.500274	0.466592	0.937836
gene-AT1G	19.73749	22.09328	20.94767	15.48303	15.00836	14.85101	6.336944	6.22102
gene-AT5G	60.15998	65.9838	61.47634	68.03979	62.76357	66.52285	17.35253	19.01015
gene-AT1G	7.604948	7.415544	7.253892	8.73459	8.571335	7.848391	7.008463	7.508874
gene-AT1G	1.718519	1.657628	1.393814	0.931311	0.836771	0.662811	1.842632	1.841614
gene-AT5G	22.74594	22.73121	25.70573	11.02282	9.43698	10.45201	16.68117	17.34599
gene-AT5G	46.37777	46.39987	31.75458	39.21763	36.76	35.51602	44.09592	74.61376
gene-AT3G	149.8558	144.4976	145.8908	141.4161	140.7881	142.3444	53.68987	54.21942
gene-AT3G	6.102805	4.828959	4.742184	6.366405	5.347004	6.111265	2.183393	1.613609
gene-AT2G	4.305241	4.241756	4.201118	4.455795	4.983708	4.196614	4.415858	3.986141
gene-AT2G	4.670228	5.738393	4.890924	3.44803	3.526362	4.368609	8.512585	7.057281
gene-AT3G	27.52802	26.78825	27.16438	15.96618	15.86932	14.76162	22.23028	24.01696
gene-AT4G	23.8537	25.87902	23.07971	4.553789	5.528848	4.554935	4.591474	5.323962
gene-AT3G	8.702559	8.905832	7.600507	7.657778	8.170114	7.376807	10.25289	10.78464
gene-AT2G	21.42708	22.19816	24.8261	17.95344	18.81794	17.12303	26.68398	27.99012
gene-AT5G	167.0663	165.9264	168.2965	358.0541	362.4655	358.8688	76.39188	78.82142
Arabidopsi	1.254227	0.59543	1.470147	1.004462	0.463912	0.931932	0.121689	0.864992
gene-AT1G	4.200779	3.683241	4.093879	4.145974	3.422362	3.306515	8.430553	8.26598
gene-AT3G	87.77758	94.48407	87.58422	70.6667	75.77119	75.37559	93.27628	87.86697
gene-AT1G	6.263814	5.777029	5.655548	5.89361	5.391288	5.377109	8.383174	8.396376
gene-AT2G	11.53911	11.48727	10.41766	12.09752	13.83553	12.25638	14.55342	16.06401
gene-AT2G	8.51689	8.701289	9.815369	14.136	13.09993	12.6691	3.404582	4.32201
gene-AT5G	6.279561	5.815114	6.012454	2.900999	3.359235	3.198581	0.742294	0.732462
gene-AT5G	5.13877	5.666293	4.906832	7.984276	9.396462	8.180247	0.408897	0.351451
gene-AT2G	265.2072	255.2613	266.1635	195.0837	193.489	200.6032	83.44585	86.86822
gene-AT2G	47.24389	42.15599	45.48965	26.35286	28.35171	30.626	76.14164	79.48706
gene-AT5G	3.636622	2.997276	3.871899	1.88516	2.108127	1.609637	3.290767	3.790808
gene-AT2G	0.510716	1.309406	0.246126	0.473254	0.317755	0.377529	2.080757	2.103462
gene-AT5G	26.94657	27.83758	27.41036	2.788267	3.349473	3.01277	18.44925	17.81514
gene-AT1G	14.22366	15.24104	15.41468	9.289077	10.25663	8.908769	2.836915	3.58345
gene-AT3G	2.143535	1.87987	2.115592	23.3166	23.662	25.71226	0.816819	0.863747
gene-AT5G	2.177313	2.460204	2.364793	3.686386	4.223375	2.910966	4.966998	6.469893
gene-AT2G	2.261482	2.332276	2.712184	1.757284	1.33333	1.251418	3.307239	2.369246
gene-AT4G	55.2913	54.91902	55.16965	71.02297	73.70638	73.99706	119.3879	122.2287
gene-AT5G	39.0568	37.20246	41.14166	23.83703	24.99677	24.77272	43.88868	51.28148
gene-AT1G	17.89098	18.71865	18.52549	17.11444	14.37853	16.6055	33.76817	35.54769
gene-AT1G	3.346832	3.444711	3.556289	2.80345	2.396921	2.081137	4.874345	3.821063
gene-AT5G	39.8526	39.91427	39.4685	29.57436	33.40302	29.35116	79.77628	83.52801
gene-AT3G	0.728856	0.800761	0.858096	0.562042	0.584571	0.441622	3.099676	2.074296
gene-AT4G	2.067469	3.119456	2.750205	4.475162	4.713233	4.7056	0.689081	1.234684
gene-AT1G	126.636	123.7506	129.0731	84.349	80.04406	80.08985	234.8028	254.2773
gene-AT1G	6.197977	5.914098	6.924834	6.786076	7.143176	6.140203	8.769347	8.272003
gene-AT3G	3.075319	2.584942	2.957679	2.108908	0.862468	1.665965	0.425119	0.988898
gene-AT5G	33.95891	34.11555	37.44178	13.46408	11.8433	12.96833	65.19278	66.98038
gene-AT1G	3.872318	2.922247	4.163579	0.320139	0.516416	0.951284	4.345367	6.70154
gene-AT3G	142.5883	135.4814	138.4121	68.9588	69.68983	66.76012	231.2799	239.795
gene-AT4G	16.7218	18.74811	19.55519	7.841954	8.351357	8.281923	6.432068	5.793584
gene-AT1G	2.473572	1.538526	1.992141	2.872778	2.365769	2.523672	1.159247	1.455753

gene-AT1G	8.3937	7.024339	8.119734	6.967874	6.989586	6.981835	3.136274	2.822117
gene-AT3G	8.486611	3.123296	7.030106	7.343687	10.39797	15.06029	6.600762	4.204544
gene-AT4G	1.221466	0.874633	1.040145	1.167721	1.457837	1.585268	0.299954	0.265241
gene-AT5G	0.860502	0.639197	0.786943	1.835141	1.383886	1.45928	0.237193	0.25241
gene-AT3G	1.962796	1.560383	1.484615	4.569942	3.922766	3.849385	1.171588	1.209173
gene-AT1G	3.704933	2.956611	3.581994	2.940004	3.501354	2.773854	2.286348	2.280785
gene-AT1G	10.18384	9.759194	8.157684	3.310535	4.49436	3.535443	5.096198	4.500296
gene-AT3G	61.06908	58.98431	59.41993	34.19853	35.67175	34.57835	88.11942	84.11723
gene-AT2G	21.7143	20.41111	22.03295	14.58306	12.82516	15.32332	33.77292	37.04507
gene-AT4G	8.631239	11.45239	11.65916	14.29415	13.5189	14.19377	5.374014	4.06192
gene-AT1G	11.66757	10.71841	11.66455	8.32884	7.759483	8.445729	14.06727	14.54994
gene-AT3G	157.2527	161.8256	148.889	103.5223	99.00561	98.37245	281.1304	272.4938
gene-AT5G	0.331184	0.472412	0.205394	0.236755	0.36769	0	0.70064	0.766833
gene-AT3G	2.107368	2.940356	2.52033	2.848408	2.439544	2.000558	2.591258	3.796083
gene-AT2G	17.66423	17.75887	18.71319	30.84966	31.66715	29.59447	13.64083	13.69325
gene-AT1G	33.72496	37.69901	28.0677	50.89074	52.02704	56.03045	66.50433	54.62183
gene-AT3G	28.50728	27.36088	31.14036	22.90377	23.22455	24.68752	39.02709	44.50806
gene-AT4G	3.250004	4.156548	4.835157	5.344138	5.286293	5.05281	0.440534	0.217155
gene-AT4G	1.534354	1.731469	1.91758	2.348686	2.26413	2.027496	2.499251	2.562105
gene-AT1G	16.76653	16.05056	18.40118	56.26409	57.27007	54.79099	2.284311	2.170027
gene-AT1G	0.950235	0.785413	0.742952	0.314077	0.583016	0.522531	1.489855	1.637306
gene-AT3G	45.96814	44.7406	44.93291	41.52262	42.20951	42.41493	9.733034	10.79189
gene-AT5G	0.4193	0.428162	0.760024	0.660827	1.026978	1.091623	0.29396	0.514469
gene-AT5G	0.1031	0.13134	0.149347	0.304803	0.509033	0.486193	0.071556	0.016752
gene-AT3G	2.567533	2.652294	2.339879	3.883404	4.124779	3.698982	0.788921	1.224787
gene-AT5G	1.805222	2.300734	1.48775	1.889017	1.028311	1.96652	0.899855	1.680477
gene-AT1G	49.84031	49.30939	48.73161	29.93569	29.48023	27.60735	86.14311	86.03368
gene-AT3G	35.45291	32.57594	32.34405	90.16904	92.26657	86.8371	58.29597	60.05592
gene-AT4G	0.71908	0.594577	0.547844	0.615423	1.596432	1.534054	0.448972	0.135916
gene-AT4G	4.286048	4.90196	4.428185	2.970301	2.646953	3.156901	7.200513	7.550467
gene-AT1G	1.606476	1.996539	1.973573	2.76105	2.351195	2.465638	0.421759	0.382842
gene-AT2G	4.148656	4.953218	4.477548	1.824943	2.026002	2.007583	8.531384	10.62023
gene-AT1G	20.42651	22.05044	21.05098	17.51611	17.35396	17.82351	26.85989	30.80672
gene-AT1G	6.159346	5.935658	6.15171	4.079973	3.886386	3.823381	5.675316	6.509886
gene-AT1G	3.193167	3.680316	3.276413	2.824836	2.558547	2.933132	3.948813	4.375015
gene-AT2G	30.36326	30.57659	31.02148	18.10016	18.56965	17.69519	100.4719	104.6527
gene-AT5G	5.893586	6.010399	5.528387	3.353812	4.134798	3.266576	3.223886	1.99208
gene-AT2G	40.42043	38.46532	36.09498	61.33811	64.90403	60.15752	20.50973	17.83942
gene-AT1G	4.267029	3.577276	4.142337	4.387377	4.693143	4.710662	4.54442	5.249083
gene-AT5G	4.593029	4.738317	3.756614	4.273885	3.565571	4.421607	4.015156	4.089282
gene-AT5G	17.22747	17.67662	19.3675	16.02358	15.85988	14.21592	61.53843	62.66183
gene-AT2G	0	0.431338	0.117816	1.769699	0.874528	1.996534	0.166092	0
gene-AT1G	33.59691	35.68423	31.42113	23.03244	20.83942	23.49162	4.918375	5.167261
gene-AT5G	0.263432	0.223848	0.35259	0.389445	0.399676	0.248159	0.352002	0.438515
gene-AT1G	13.67236	11.71472	12.13971	17.14521	16.47264	17.94953	8.721432	9.21035
gene-AT4G	20.38998	21.69591	19.62147	19.97286	19.86949	19.67882	27.40606	28.64395
gene-AT2G	7.325321	9.84769	7.871688	40.28011	40.0455	39.67598	6.991845	8.660667
gene-AT4G	6.28304	6.592692	8.056943	8.042343	8.727654	9.940784	5.872928	7.842268
gene-AT3G	0.788852	0.616194	0.809307	0.516921	0.627849	0.520126	1.049413	1.083104
gene-AT1G	2.207482	2.6295	3.889395	0	0	0	5.493049	5.556019
gene-AT1G	2.815713	1.811369	1.681862	1.804012	0.844964	1.39017	1.912109	2.091153

gene-AT2G	5.340575	5.51335	5.909423	2.373749	2.278879	3.303586	7.738119	8.809521
gene-AT3G	1.024819	1.275295	1.109915	1.32174	1.487063	1.564314	0.523905	0.085032
gene-AT3G	11.93245	13.40461	11.79743	9.368828	9.418815	8.706738	4.347934	4.665251
gene-AT1G	3.465377	4.149755	4.379022	2.26247	2.086575	2.244779	4.482851	4.696601
gene-AT4G	0.475605	0.272598	0.551407	0.731348	0.762964	0.582197	0.489197	0.46151
gene-AT3G	3.022941	1.10609	1.908013	1.240189	1.528032	1.649356	1.409335	1.212723
gene-AT4G	10.59069	11.03294	13.56262	6.679993	6.610102	6.55551	4.170115	3.839169
gene-AT5G	5.463888	6.017119	4.499675	14.50285	12.62219	14.50622	0.211144	0.377997
Arabidopsi	0.688589	1.243218	1.144264	1.142937	1.530705	1.241278	0.60332	1.208296
gene-AT2G	6.990013	8.398752	7.902941	5.476761	5.673961	5.457487	11.2563	10.92839
gene-AT4G	0.918326	1.988612	2.837099	2.587885	2.598804	3.993735	1.351204	1.737804
gene-AT5G	5.077648	7.836315	6.80356	2.443515	3.424366	2.235696	2.198528	3.629207
gene-AT4G	147.4353	145.9382	148.7476	220.1344	218.5632	213.4412	119.0986	123.8776
gene-AT5G	7.370966	6.893576	7.465982	4.409935	5.910778	6.1518	15.13876	17.89953
gene-AT5G	9.389553	8.531385	7.760294	5.583581	4.749763	6.855117	15.14707	14.02351
gene-AT5G	63.37656	61.51489	62.34031	76.81023	79.53876	69.96116	41.64461	37.71518
gene-AT1G	24.17668	23.14684	23.14804	19.12167	17.175	18.96103	31.86263	35.01616
gene-AT3G	0.499679	0.573014	0.499677	0.316644	0.475549	0.164294	0.091409	0.202829
gene-AT3G	11.7116	10.25578	8.361939	14.48355	14.33982	16.31479	6.303874	5.973429
gene-AT1G	1.863144	2.770077	2.479962	5.430868	3.515017	5.589164	2.377126	0.949005
gene-AT1G	5.75812	4.83788	4.992782	10.22967	11.16164	11.11435	6.735374	5.528434
gene-AT5G	9.253603	10.81027	10.62228	13.51218	17.17505	16.20823	4.183645	5.855392
gene-AT1G	7.718982	8.206581	8.13018	13.26533	15.34699	15.89027	0.671253	0.577552
gene-AT3G	0.372987	0.164442	0.29449	0.189602	0.268911	0.191475	0.813916	0.963267
gene-AT3G	3.114277	3.22884	3.837292	2.492651	2.118445	2.454724	2.495769	4.395045
gene-AT5G	23.92408	24.28461	23.99036	67.64005	68.81159	67.88266	4.069037	4.848781
gene-AT3G	4.188527	2.814891	2.82852	1.894843	1.854154	2.575236	3.719821	4.64855
gene-AT3G	7.620806	9.248658	7.802882	3.032108	2.530828	2.532878	9.085808	9.362811
gene-AT1G	2.500299	3.586463	2.952589	5.723567	6.244728	6.582649	3.972192	3.236093
gene-AT1G	4.380764	3.996894	4.419561	1.655685	1.941865	1.97813	1.474061	0.778879
gene-AT1G	29.79965	27.46145	27.61493	24.82444	25.24391	22.73989	14.10078	15.23558
gene-AT1G	37.61164	39.99518	35.84977	13.18677	13.13906	12.74802	38.87371	37.80961
gene-AT5G	6.393534	7.098868	6.665047	8.462114	7.692895	7.692106	2.431053	3.253256
gene-AT5G	79.0439	77.9247	77.1554	45.51668	44.78458	44.19158	130.2151	137.6101
gene-AT1G	181.6485	175.1236	179.3642	111.7408	113.6126	112.6943	126.2875	128.069
gene-AT2G	6.060015	5.359104	7.220331	36.70815	36.70707	37.61705	0.444156	0.763378
gene-AT3G	0.479139	0.536389	0.401189	0.865657	1.313691	1.351585	0.913378	0.835559
gene-AT1G	1.68837	1.651201	1.276683	1.289624	1.462067	0.966474	5.745531	6.945462
gene-AT1G	1.770363	2.580141	1.952883	0.972853	1.158144	1.124158	3.266101	3.146414
gene-AT3G	18.76882	19.88638	19.33479	35.71849	31.37903	33.13199	20.02775	20.81618
gene-AT3G	1.04301	1.190566	1.724178	2.151572	2.51687	2.258609	0.724594	0.947714
gene-AT1G	46.02062	42.10037	42.98424	22.34268	20.20902	20.53194	66.71417	65.3971
gene-AT2G	6.532498	7.142788	6.409393	3.185462	3.137423	3.517591	6.758577	6.468469
gene-AT4G	4.116194	6.099615	5.911977	6.230866	4.562722	6.178349	2.42494	1.967214
gene-AT5G	2.151392	1.642967	1.738506	2.853825	3.164169	2.165107	1.210193	1.257449
gene-AT5G	9.45799	8.781584	11.30942	6.744337	6.607048	7.936345	10.23462	9.207778
gene-AT2G	27.83007	26.95957	27.88844	8.793107	9.518573	9.053524	13.11124	12.289
gene-AT3G	4.471148	4.846848	5.542125	4.507756	6.709926	4.252117	4.521551	4.432998
gene-AT5G	2.423034	2.184567	2.153758	1.405906	2.296332	0.905135	1.329484	1.89355
gene-AT4G	7.978832	5.177489	6.666574	9.742865	9.532742	8.857892	5.832603	4.85034
gene-AT4G	21.6085	26.28302	25.05402	25.92971	24.21217	27.1639	19.86568	20.46351

gene-AT2G	3.72171	3.456245	3.744654	3.872881	4.351507	3.597128	4.70143	4.778275
gene-AT5G	1.215296	1.297787	0.820935	0.492421	0.34888	0.538301	0.978448	0.83674
gene-AT1G	38.19336	36.90765	35.75585	20.84313	21.67769	21.66068	61.77506	59.93002
gene-AT2G	20.12693	18.0252	18.1823	7.877394	6.672231	7.772996	41.78879	40.93284
gene-AT5G	18.15175	18.46929	19.42647	22.06301	22.07786	23.24716	12.88402	14.18031
gene-AT5G	2.341958	1.939655	1.696625	2.303541	2.158241	2.697045	1.511097	0.939102
gene-AT3G	5.642588	5.847286	5.420635	6.477164	6.731027	6.523892	9.662223	10.87106
Arabidopsi	1.225513	0.92703	1.014032	0.613466	0.423588	0.480695	0.812024	0.96629
gene-AT4G	5.988865	7.463806	6.554464	12.77158	12.25322	12.37814	6.529986	6.265791
gene-AT1G	3.779527	4.826	4.657552	4.443935	3.447738	4.54443	1.718423	2.005945
gene-AT1G	4.509761	3.616496	4.531474	3.377433	4.575198	3.699771	1.354822	1.626706
gene-AT4G	3.641888	6.308968	4.451404	3.75513	4.868803	4.513407	7.840101	6.071113
gene-AT2G	4.667585	6.31626	5.773775	3.735695	3.591032	3.468444	4.806949	4.09728
gene-AT3G	2.13469	2.182853	1.996701	3.408466	3.513073	2.682482	1.784341	1.527097
gene-AT1G	1.087546	1.290875	1.382039	2.145166	2.087597	2.487672	1.232783	1.017612
gene-AT3G	11.32086	12.89789	10.9402	13.78109	15.81037	13.86602	12.30438	12.4262
gene-AT1G	3.11079	2.941681	2.805357	6.528393	6.344953	7.003368	3.689285	4.026546
gene-AT2G	0.272041	0.522575	0.536229	4.554904	2.98753	4.453075	0.22458	0.160308
gene-AT2G	76.95751	75.55985	77.73424	39.35321	41.84729	39.7465	68.33945	72.2245
gene-AT1G	5.224788	5.575742	5.221021	30.53915	28.13688	31.08066	6.873176	8.746796
gene-AT1G	36.71838	36.15662	37.1396	111.3913	106.9164	107.4325	58.3139	62.58229
gene-AT3G	5.960752	8.212785	6.900308	6.41201	5.915479	5.678356	7.835843	8.210484
gene-AT5G	3.46541	3.111321	4.218732	5.113333	4.838929	5.280423	3.931387	3.094182
gene-AT2G	10.14485	10.88158	10.8982	25.32336	25.30592	24.0402	2.49531	3.129817
gene-AT2G	16.45661	16.19262	15.32315	14.80951	13.28231	16.11166	15.37917	17.93103
gene-AT3G	21.20828	19.44593	22.01759	24.57917	25.27862	25.72145	4.092271	5.607571
gene-AT2G	0.866428	1.19754	0.898258	1.191292	1.325083	1.394126	0.450083	0.484516
gene-AT5G	91.79724	85.5034	88.14723	135.5201	139.5065	128.2345	74.94293	80.94576
gene-AT5G	5.329461	4.207658	5.226784	3.912147	3.575435	4.321717	1.052795	1.51449
gene-AT5G	0.652939	0.688708	1.048823	0.406154	0.789556	1.076621	4.279778	4.142459
gene-AT1G	10.63504	10.17964	10.64804	8.380082	8.616437	8.245387	13.11676	11.14484
gene-AT5G	87.46311	84.36774	85.58175	32.00334	33.81746	34.25303	53.66739	53.13714
gene-AT2G	0.85245	0.726248	0.927392	1.28255	1.456927	1.244415	4.248207	3.207029
gene-AT4G	44.46933	41.80339	42.36519	55.01407	57.11597	55.18833	28.39558	29.82169
gene-AT1G	4.990523	3.604203	4.056209	7.150831	5.690084	5.865005	1.465379	1.497645
gene-AT4G	3.376148	3.024924	3.10023	1.064256	1.362008	0.837627	6.644425	5.40466
gene-AT4G	3.44092	3.201876	3.312818	13.78031	13.36328	14.2135	0	0.053785
gene-AT4G	25.91715	24.71106	26.06707	11.49657	10.83581	11.69329	17.51072	19.98634
gene-AT5G	22.43219	23.57146	22.22301	32.12278	33.01258	37.44746	5.767371	5.017094
gene-AT2G	10.65233	11.32009	10.67511	14.83654	14.955	14.99896	9.838359	8.889037
gene-AT3G	11.28219	11.41448	11.16074	8.436425	9.108245	7.953907	26.2694	25.14098
gene-AT3G	1.873901	1.714123	1.167698	2.427953	2.803393	2.62719	1.851136	1.593087
gene-AT2G	19.28113	19.87388	22.46302	26.04839	27.53079	28.77587	14.4993	15.50987
gene-AT2G	4.155271	3.976909	3.933702	4.551887	4.51124	5.325572	1.615528	1.640193
gene-AT1G	1.600466	0.997345	0.943699	0.888825	1.066996	1.228275	2.449047	2.136126
gene-AT5G	1.507109	1.798268	1.819699	0.400946	0.255918	0.295731	1.984354	1.412642
gene-AT3G	6.336797	4.945033	6.314869	4.179768	4.339516	4.644455	6.595142	6.23836
gene-AT3G	2.312646	2.199007	2.767812	0.2994	0.303934	0.384026	0.154168	0.246848
gene-AT3G	3.128198	1.907042	2.534935	1.815647	1.800603	1.702716	4.811996	8.203163
gene-AT5G	3.00648	2.818825	2.535672	0.784004	0.88143	0.966612	5.01205	4.072785
gene-AT5G	11.01351	10.09955	11.46828	15.69592	14.68991	14.93464	10.55615	10.68421

gene-AT3G	10.927	11.54134	10.5871	14.95881	16.34312	13.01036	3.65319	4.86231
gene-AT3G	5.366176	4.587898	4.824815	3.877423	3.845464	4.017369	1.8327	2.379025
gene-AT4G	1.414434	1.664747	1.573498	1.623395	2.155799	2.00476	2.451875	2.347215
gene-AT3G	3.972405	3.739182	3.197861	6.896632	6.499089	7.65587	2.4987	2.310927
gene-AT1G	45.78404	44.26304	46.76675	33.83733	31.73219	33.53037	62.62164	63.55552
gene-AT5G	0.361461	0.220423	0.533473	3.023049	5.520127	2.716885	0.161744	0.051735
gene-AT3G	3.261868	3.498427	3.769435	3.544243	3.852498	4.302096	4.286622	4.441144
gene-AT5G	13.14033	13.21516	14.75839	11.4914	9.608418	8.775856	30.68124	34.11329
gene-AT1G	1.203035	2.145567	1.934737	1.578374	1.030925	1.936641	4.103446	3.842837
gene-AT3G	7.50607	8.645924	8.268588	6.21019	6.892926	6.647982	3.272797	3.783431
gene-AT3G	39.96713	42.03994	40.89593	57.56881	56.58662	58.04302	66.41731	64.67726
gene-AT1G	149.118	147.7422	147.7366	96.24234	95.083	92.10643	248.399	229.6505
gene-AT4G	4.053851	4.57007	3.977335	9.250462	9.513952	9.049925	1.502606	1.438177
gene-AT1G	14.74333	14.85051	15.34823	116.7655	117.2693	112.7156	20.44648	20.95279
gene-AT3G	38.11385	37.27218	32.06139	33.91779	36.21801	34.07048	40.00611	42.00484
gene-AT2G	9.787674	8.157431	8.362339	11.56218	10.24768	11.14443	2.993241	3.060404
gene-AT1G	1.098163	0.823796	0.784267	0.829212	0.856716	0.495102	0.315149	0.524752
gene-AT1G	12.49938	13.18443	12.81815	14.53456	14.21548	13.88777	15.19626	17.18154
gene-AT2G	957.3616	1138.838	893.4849	1257.106	1385.353	1461.578	455.7853	396.221
gene-AT1G	25.25324	26.16821	25.43783	11.38663	11.65958	11.11936	36.76306	39.86604
gene-AT4G	7.442664	6.812631	7.287188	7.538495	8.51435	8.644022	9.57305	9.775391
gene-AT2G	0.912561	1.451186	0.936504	1.717883	2.209113	1.22421	1.048825	0.842607
gene-AT1G	1010.824	1003.407	1016.794	606.5894	605.1251	594.5349	1641.53	1641.722
gene-AT5G	0.300742	0.404351	0.45637	1.184587	1.075871	0.796655	0.721489	0.87628
gene-AT5G	16.59685	17.43272	15.9238	14.04516	13.61329	12.27624	25.54554	27.54925
gene-AT5G	10.22778	10.09956	9.457935	15.49011	13.90057	13.01656	3.641844	4.78545
gene-AT4G	2.354935	2.216267	2.364674	1.777195	1.770416	1.648057	4.543818	4.177146
Arabidopsi	30.54626	31.92032	30.61646	8.459737	9.071735	8.007323	2.039759	2.288173
gene-AT1G	1.812725	1.970147	2.171898	2.387952	2.343486	1.644113	2.641313	2.602776
gene-AT5G	21.73906	19.56865	19.8688	20.3254	21.53251	21.63986	31.7247	30.7885
gene-AT1G	258.6639	268.0626	281.9332	132.995	133.7595	129.2375	494.4907	495.0704
gene-AT1G	19.6727	18.97098	20.67062	13.14231	12.4936	12.117	19.77038	20.25639
gene-AT5G	5.565489	6.45624	6.548004	0.929652	1.242252	1.549698	0.174827	0.191728
gene-AT5G	4.35959	4.737252	4.981997	5.972203	6.51781	5.616989	3.663665	4.53278
gene-AT1G	0.80788	0.548552	0.542483	0.632737	0.799876	0.744166	0.072717	0.099666
gene-AT3G	5.219242	4.278891	3.525193	1.751163	2.398383	2.084328	4.909396	4.971975
gene-AT5G	6.39928	8.932767	6.57268	6.311681	3.446751	3.887018	11.13631	9.003877
gene-AT2G	2.504562	2.453891	1.894513	2.011079	1.672167	1.814553	2.761902	2.560279
gene-AT2G	2.58612	2.062165	2.963172	2.84745	2.550958	2.89582	5.589993	5.889271
gene-AT5G	3.934245	3.657652	4.577781	3.108044	1.846664	2.887155	4.040958	5.140526
gene-AT5G	26.25392	28.19193	21.88887	16.45899	19.6752	16.88075	15.23037	10.49115
gene-AT1G	57.807	58.87224	53.99781	36.01064	38.81218	36.96106	16.11942	19.44581
gene-AT3G	20.53557	18.89438	18.58719	12.81006	13.80947	15.97907	30.93245	30.79746
gene-AT5G	2.186222	3.155526	3.484735	1.781374	1.817831	2.782401	1.837922	1.255892
gene-AT5G	1.993773	1.344445	1.022745	2.246432	2.316719	1.67515	0.265487	0.094901
gene-AT5G	1034.017	1023.846	1069.06	704.6173	698.2076	680.3298	1672.544	1689.974
gene-AT5G	18.49612	17.61331	16.83798	14.18737	15.22301	16.0706	30.03511	28.96275
gene-AT5G	14.33654	14.56548	13.24118	9.582854	10.25737	10.72521	16.17181	17.88608
gene-AT1G	1.041809	1.328421	0.958407	3.197896	3.534707	3.450892	7.183557	8.511923
gene-AT3G	7.575881	7.026899	8.614224	8.380516	7.755202	7.525016	10.49036	13.9414
gene-AT3G	0.206306	0.186154	0.285185	0.76529	0.459809	0.868653	0.282489	0.094198

gene-AT5G	29.29995	29.31549	29.33319	20.89612	22.56603	20.7212	25.35214	25.33887
gene-AT4G	34.45273	34.6353	35.73188	34.95634	35.59202	32.08295	23.25414	22.71944
gene-AT5G	6.515088	5.341906	7.052989	21.33467	23.41613	24.01858	29.56722	34.18018
gene-AT4G	466.4632	484.7524	458.9188	423.0381	441.6439	451.0932	450.9983	425.1391
gene-AT3G	5.497226	7.029223	6.927951	7.828126	7.993119	8.540942	6.905433	8.050249
gene-AT1G	3.930758	4.088018	4.880416	3.537884	4.058651	3.974377	4.609741	4.87135
gene-AT1G	4.878097	4.088605	3.748018	5.344201	7.42946	6.488266	4.068483	4.454149
gene-AT1G	2.327383	2.029646	2.120493	4.650604	6.489565	5.861578	4.985303	4.096321
gene-AT5G	0.263789	0.145391	0.174232	0.571862	0.580549	0.650732	0.786693	0.22544
gene-AT3G	4.29739	5.482544	5.769782	8.343038	8.207814	9.16798	3.115148	3.76344
gene-AT4G	0.475839	0.541096	0.755051	0.235872	0.535692	0.277517	1.552269	1.717774
gene-AT5G	28.00562	26.96197	29.65518	18.29815	16.53391	18.64215	50.14597	49.14478
gene-AT4G	38.94544	41.28262	40.31337	29.73789	29.72241	28.2525	36.51596	40.18126
gene-AT4G	5.341311	4.015095	4.430357	3.155415	3.071062	3.589027	1.436052	1.370041
gene-AT1G	5.011949	4.807283	5.389557	4.034064	3.650634	4.181632	8.192159	8.372715
gene-AT1G	7.649824	7.929929	7.795916	8.864207	8.492339	10.32968	5.723321	4.877362
gene-AT3G	122.543	120.2993	125.1685	80.03363	82.59874	79.25108	214.3838	210.0417
gene-AT3G	1.361268	2.65357	3.27674	4.708267	3.677128	3.74774	4.682197	3.177042
gene-AT2G	2.399952	1.916348	2.185915	1.068084	1.011401	1.214924	0.39794	0.695238
gene-AT1G	14.77842	14.80341	13.47179	16.36548	17.59287	15.07603	3.166067	3.314406
gene-AT1G	9.572396	8.090707	8.883193	4.974699	5.949686	5.310166	8.486052	9.151549
gene-AT3G	0.700625	1.042063	1.265278	0	0	1.174111	0	0
gene-AT3G	2.266532	1.813735	2.842641	1.917523	1.926571	1.916986	3.823689	3.87406
gene-AT5G	10.2102	10.5065	8.294958	5.678052	8.500831	8.215587	10.27771	10.54933
gene-AT5G	369.1093	364.2456	358.2827	166.1598	158.6769	153.9407	954.6477	916.0434
gene-AT3G	6.869257	7.61506	7.771229	11.50645	10.76013	12.09179	4.451903	5.847309
gene-AT5G	15.80855	15.88454	17.5665	289.2231	297.9895	295.0704	16.55501	18.59945
gene-AT3G	69.07443	67.53256	67.31463	32.13094	34.44504	30.86058	33.49767	33.40817
gene-AT4G	4.438556	4.460341	3.789578	0.39715	1.228593	1.532964	3.408939	3.038432
gene-AT4G	2.592829	2.94438	3.338639	0.434652	0.479668	0.896922	0.857749	1.283464
gene-AT3G	7.753152	7.047819	6.557861	6.959248	7.441518	7.651027	1.890608	2.115989
gene-AT3G	5.900116	3.957087	8.933687	5.675148	5.509384	7.148529	10.07962	8.953403
gene-AT1G	15.72688	16.9336	15.74672	8.271763	8.232989	6.85781	15.29923	20.22673
gene-AT5G	0.95768	0.386293	0.594649	0.288596	0.340165	1.012826	0.438288	0.74517
gene-AT4G	2.589958	2.362684	2.089952	1.590469	2.08559	1.772085	2.204135	2.355444
gene-AT5G	6.255308	5.551399	5.000389	5.064054	4.301235	5.066933	7.04066	7.60658
gene-AT1G	2.709318	2.559789	2.632848	2.053855	1.402263	1.785805	6.365502	7.564187
gene-AT2G	114.2193	116.1954	117.8276	155.7365	155.4724	161.6528	125.9897	128.1097
gene-AT3G	6.314884	6.268543	7.377847	14.5913	14.54096	14.51998	6.744335	6.681521
gene-AT1G	11.69824	12.26209	11.53859	4.713886	5.638986	5.887625	4.14959	2.893608
gene-AT3G	31.37577	31.20363	30.60756	22.73607	23.58638	21.49434	46.90035	49.16122
gene-AT2G	61.19331	58.6808	53.92065	84.72675	81.75462	85.22042	78.17207	75.67796
gene-AT1G	7.693146	6.596	8.914829	5.018476	5.961111	4.602754	11.60901	13.9852
gene-AT3G	23.80168	24.78322	24.38945	25.56628	25.45805	26.31359	38.14025	36.67909
gene-AT3G	37.85592	43.99468	31.63303	23.92306	24.1054	23.4713	38.27282	38.03442
gene-AT5G	6.338036	7.933194	9.429013	10.76937	12.35656	11.39919	5.29911	5.361315
gene-AT1G	0.820912	0.757764	1.670107	2.636388	2.417503	2.472195	0.64576	0.611746
gene-AT4G	3.875592	3.57293	4.365607	3.711363	4.525611	4.531774	1.363029	1.302198
gene-AT2G	21.50603	25.12136	26.50598	47.40156	51.32674	50.11631	17.95024	18.02422
gene-AT3G	2.192984	2.266055	2.057338	2.951294	3.900095	3.617337	7.955538	7.005514
gene-AT5G	34.29435	33.79637	34.61547	65.01432	61.94038	69.02839	29.44524	31.21647

gene-AT5G	124.1638	119.3738	120.0888	38.27025	40.58967	38.57551	51.03213	51.26472
gene-AT2G	14.18578	15.89195	11.9156	11.74497	11.21469	13.16866	26.32393	24.32281
gene-AT2G	8.747604	6.973572	8.531846	6.680398	6.551246	6.754166	1.332564	2.051022
gene-AT2G	6.958692	6.67153	6.67628	7.620941	8.454707	8.759179	1.568235	1.763921
gene-AT3G	1.014723	1.737315	0.485125	1.515391	1.376686	0.591238	0.274128	0.214161
gene-AT5G	11.04598	12.61078	11.27275	18.26597	16.6017	17.32679	12.51611	13.84159
gene-AT4G	24.3005	22.92089	25.38444	41.51805	40.07308	39.60098	61.88605	65.2997
gene-AT2G	14.49409	14.50899	15.98287	7.502396	7.475599	6.795677	2.106421	2.125422
gene-AT3G	1.351562	0.988613	1.349234	0.936222	1.567674	1.022075	1.390806	1.758789
gene-AT3G	4.629853	5.676538	5.600514	4.225907	5.109403	4.891322	14.36024	17.75728
gene-AT2G	27.7343	25.64857	25.83913	34.41863	31.02815	30.4514	21.31801	22.00621
gene-AT3G	12.43128	12.95024	14.02519	13.29847	13.16935	15.26214	17.78896	20.0415
gene-AT2G	20.08826	20.09259	20.91872	15.90622	18.01304	16.2878	30.37063	29.46002
gene-AT1G	2.263869	1.9704	2.075663	2.943903	2.711552	2.582452	2.846202	2.111438
gene-AT5G	1.443719	1.480019	1.436712	2.08935	2.352561	1.647752	1.82914	1.054015
gene-AT3G	4.998866	4.430012	5.214869	5.514299	5.09092	5.409905	5.007645	7.204426
gene-AT2G	0.318522	0.292658	0.464503	0.19624	0.185869	0.238129	1.372125	1.187451
gene-AT2G	36.62139	39.60044	37.3281	9.033434	8.461953	9.356607	5.403334	5.435413
gene-AT2G	9.112158	11.15089	11.72221	11.94233	12.68229	14.36554	8.524939	9.471386
gene-AT4G	95.42647	96.61544	100.4008	73.48907	71.34515	69.07491	198.423	196.746
gene-AT1G	11.27579	10.10774	11.97475	12.24573	13.16914	12.50928	9.10133	9.582414
gene-AT1G	0.717067	0.521426	1.056817	0.691331	0.712616	0.694783	0.141189	0.099826
gene-AT2G	3.11849	2.695646	3.396313	4.30541	5.75487	5.682668	2.002978	1.877299
gene-AT1G	1.617257	1.195751	1.29907	0.627814	0.638223	0.703614	2.785739	3.160241
gene-AT1G	3.035065	3.083881	2.892772	1.35386	1.505237	1.800849	2.633972	2.498759
gene-AT5G	121.7144	116.6868	115.2064	75.84552	76.20749	81.63994	203.179	213.4361
gene-AT5G	47.72485	49.32325	48.45927	39.14539	44.3156	41.52119	75.81786	73.33992
gene-AT1G	0	0.153467	0.173434	0.516673	0	0.399469	0.144972	0
gene-AT1G	14.90506	14.74062	15.3686	24.38015	26.56299	26.53473	2.214544	1.33924
gene-AT5G	35.57192	33.53905	33.0197	7.87326	8.324926	9.52901	1.333046	1.032322
gene-AT2G	0.604535	0.607878	0.708337	0.489227	0.355071	0.287865	0.458792	0.830668
gene-AT5G	66.81046	63.4774	67.27225	55.82974	59.50101	57.05397	13.09649	13.94871
gene-AT3G	5.836254	5.964326	6.254142	4.959112	4.577243	5.640569	7.785349	7.034141
gene-AT1G	1.533998	1.550548	1.891518	1.21071	1.269719	1.201166	1.457056	1.356843
gene-AT1G	41.81626	47.04262	41.49718	68.2468	72.1648	63.89315	29.53522	25.62763
gene-AT4G	13.71985	14.74936	14.12095	15.06012	14.31197	16.96752	11.19704	12.87787
gene-AT3G	0.820204	0.75115	0.722479	1.109362	1.041487	1.2207	0.756661	0.33341
gene-AT1G	0.177989	0.109214	0.180484	0.431997	0.559029	0.650675	0.918531	0.61466
gene-AT1G	8.362065	7.988794	8.126742	10.8228	11.25523	11.12561	2.433549	2.313562
gene-AT3G	4.930369	7.294246	5.635258	5.933411	6.890274	4.977343	2.017026	2.194329
gene-AT3G	175.5586	179.6441	182.7771	657.9161	637.0386	631.7917	75.7479	82.94862
gene-AT1G	1.221202	1.272948	1.422621	1.629378	2.168957	1.359209	0.820989	0.810018
gene-AT5G	29.35376	27.42394	27.89569	17.28428	17.1743	16.14134	41.05398	40.85945
gene-AT1G	9.36848	10.11341	9.456599	4.052928	5.208936	5.427131	4.758829	4.618016
gene-AT4G	0.588739	0.304191	0.49305	0.539101	0.79324	0.42595	0.727112	1.066893
gene-AT1G	5.361856	4.768816	3.959152	3.685173	3.488675	4.112143	5.338419	7.483658
gene-AT5G	4.963717	5.50174	4.523236	4.421998	3.84871	4.11597	7.047873	6.35011
gene-AT5G	0.023271	0	0.024059	0	0.072765	0.090381	0	0
gene-AT3G	2.452989	2.24625	2.145346	2.919452	2.574018	1.92699	2.298285	2.814273
gene-AT2G	0.24645	0.245333	0.073683	0.110398	0.266477	0.322872	0.599386	0.713487
gene-AT2G	23.61002	22.72236	23.36106	40.23866	40.5559	38.86393	16.793	16.37773

gene-AT3G	2.20918	2.844225	2.184604	0.695407	0.95103	0.794415	1.014884	1.148073
gene-AT5G	12.44429	12.31509	11.53607	12.94172	11.71597	11.89648	16.8365	17.63747
gene-AT5G	0.788024	0.603675	0.826565	2.107141	1.456998	1.403785	0.955908	0.816067
gene-AT4G	3.898931	4.690892	5.712335	3.543282	3.715366	4.328712	1.956528	3.474055
gene-AT3G	25.82716	27.90627	27.71346	20.00769	20.7926	22.27416	49.77963	47.23457
gene-AT3G	1.675357	1.886824	1.233752	0.933907	0.108384	0.709241	4.031769	5.325978
gene-AT3G	30.62476	28.84829	30.33339	53.67968	53.25367	54.73772	13.64646	13.34665
gene-AT3G	317.0943	315.4189	317.2733	103.2354	100.9333	99.63817	657.5875	739.8533
gene-AT4G	4.532283	3.255773	3.129933	13.95612	14.314	15.38221	7.174583	6.574841
gene-AT3G	8.008854	7.880703	7.601201	14.89618	15.43746	13.29703	5.558894	6.01143
gene-AT5G	12.05798	12.77795	12.58936	7.753089	8.666551	8.702715	16.02605	15.25034
gene-AT4G	11.29141	12.14136	8.989231	10.78842	11.77298	10.74371	17.81628	16.18774
gene-AT3G	4.607958	6.057778	4.611121	6.714681	5.666264	5.291653	9.621553	12.24223
gene-AT2G	222.1549	230.3442	231.905	179.9226	177.474	179.3532	256.1319	285.144
gene-AT5G	23.43279	23.08916	23.60794	45.62678	47.90351	46.64786	17.39419	20.42467
gene-AT2G	11.89167	11.73676	11.53267	28.48051	29.42965	25.81158	10.83069	9.603637
gene-AT1G	10.18618	9.396508	9.766751	11.38098	11.98398	10.12565	8.153537	7.058918
gene-AT4G	9.008278	8.398124	8.775701	6.303787	5.482546	5.599695	9.660112	9.79029
gene-AT1G	13.17402	12.84905	15.36342	28.40706	30.93668	30.61524	10.42014	10.24672
Arabidopsi	4.032961	1.560407	2.440139	1.361657	1.069443	1.599904	2.250797	2.306158
gene-AT5G	1.186187	0.944196	1.455826	1.082817	0.626715	1.138847	1.15701	1.234261
gene-AT5G	7.425958	6.825805	7.586757	5.010749	4.583945	4.376276	1.356559	1.682693
gene-AT5G	240.8521	230.429	231.7721	133.9006	133.8261	127.4626	210.6935	216.8439
gene-AT2G	2.868792	2.323627	2.682547	1.116684	2.179308	1.473421	6.976166	6.162961
gene-AT1G	1.789175	1.288189	1.647236	0.861297	1.139152	1.827291	1.923936	2.38695
gene-AT2G	1.327357	0.742875	0.958385	2.293971	2.979407	2.278353	1.801424	1.735361
gene-AT2G	2.385797	2.115202	0.969389	7.49983	6.705459	6.174296	3.189881	3.074651
gene-AT2G	1.901332	1.821212	1.268516	4.222989	4.372148	4.658411	0.936135	0.780355
gene-AT3G	7.412061	8.172664	8.073482	5.079676	5.400942	4.944708	5.578715	5.206394
gene-AT5G	0.756496	0.914161	1.342997	0	0.121522	0	0	0
gene-AT2G	20.1961	20.29762	19.84746	14.80952	15.77314	13.89761	19.01758	17.96015
gene-AT1G	4.128667	8.350893	5.161907	4.740116	3.508761	3.575935	6.740946	4.973116
gene-AT4G	0.638383	0.707884	0.763559	0.423509	0.264546	0.267772	0.256815	0.300932
gene-AT4G	2.075226	3.530813	2.658884	3.026307	3.684694	3.831328	4.160223	3.637058
gene-AT4G	8.121717	7.852855	8.214005	10.27645	10.08233	11.24292	10.65904	11.89385
gene-AT4G	24.12224	28.01501	26.56057	22.44633	19.58054	20.32414	23.33775	28.03535
gene-AT2G	55.46454	51.78635	58.16121	38.16847	42.18222	41.27982	82.43883	82.46165
gene-AT1G	11.30584	10.92424	10.58272	2.272575	2.852462	3.383195	13.44669	14.04767
gene-AT4G	29.02682	27.36148	28.28332	25.08743	25.96479	27.70527	11.79312	13.69744
gene-AT3G	2.978748	2.628957	3.711445	1.599457	1.343572	1.583716	0.043123	0.036946
gene-AT3G	1.906814	2.236533	2.24895	10.0123	9.17347	9.728012	6.119558	6.165841
gene-AT5G	29.08015	30.89854	30.57802	20.09019	19.13193	20.79978	52.08968	55.39868
gene-AT3G	11.55337	12.42844	9.740583	37.15257	43.80915	39.04578	36.61779	43.21334
gene-AT4G	173.1921	164.0254	161.4098	490.6169	486.6471	499.4884	79.98235	81.247
gene-AT2G	4.055128	3.920265	3.656721	3.946985	4.181717	4.18241	5.807475	5.533056
gene-AT5G	2037.409	2007.389	2005.018	1062.251	1077.311	1073.691	4083.637	4306.942
gene-AT1G	0.169995	0.145114	0.209397	0.337439	0.326818	0.573845	0.2205	0.14849
gene-AT4G	16.14128	15.76903	16.11491	11.93207	11.57742	12.22145	22.74113	24.33479
gene-AT1G	3.12306	2.963424	2.971378	3.407233	2.378514	2.946015	4.250207	4.537865
gene-AT3G	103.2766	103.266	106.1339	82.38202	77.31294	78.1964	182.2086	181.8033
gene-AT5G	147.7194	156.7185	156.6258	130.0348	129.0281	127.229	264.0417	270.623

gene-AT1G	0.579443	0.549735	0.680314	0.372133	0.324542	0.091762	1.332797	1.306531
gene-AT1G	0.882532	0.868186	1.493998	1.207305	1.638977	1.558363	0.224813	0.311561
gene-AT3G	5.514733	4.814347	4.489164	3.630702	4.187634	4.465844	8.210913	6.293596
gene-AT3G	1.54854	1.702069	1.599785	2.887578	2.952066	2.813304	1.109735	1.40911
gene-AT4G	4.699287	4.488306	5.052108	9.342314	8.704939	8.918745	1.890241	2.019209
gene-AT3G	21.24605	20.26767	18.76994	11.67919	12.18448	13.22451	34.21393	34.64534
gene-AT3G	263.3403	258.5083	256.7427	278.7977	272.3948	268.0954	335.3193	348.325
gene-AT2G	20.16054	17.15856	19.49679	14.6465	14.61753	14.43331	32.21951	31.5693
gene-AT3G	0.739085	1.176161	0.636284	1.236465	0.793211	0.873404	0.055516	0.506917
gene-AT1G	0.094327	0.043648	0.217682	0	0.143588	0.073029	0.149991	0.213284
gene-AT1G	6.765248	7.135254	7.968194	6.106139	7.009926	7.516886	3.806527	3.221025
gene-AT3G	6.713464	6.459258	6.12303	7.70859	6.953437	6.636187	12.43769	14.13976
gene-AT3G	1.687325	1.85716	1.438102	2.762285	2.441537	2.798784	0.447616	0.421829
gene-AT1G	1.690829	1.08379	1.510887	1.466727	1.807359	1.386235	1.134151	0.988279
gene-AT1G	1.409747	0.900721	1.07456	2.58128	2.317798	2.733297	4.558893	5.095093
gene-AT5G	2.718589	2.806049	2.886966	1.357802	2.023868	1.683025	5.301259	5.172925
gene-AT1G	17.17501	17.17229	21.53015	21.59149	20.36551	21.78422	45.53107	43.57977
gene-AT3G	17.82542	17.89295	16.05797	160.8142	171.779	153.3264	3.957589	4.216817
gene-AT2G	17.76526	17.97166	17.24296	14.52682	13.30478	14.28602	32.64921	33.01969
gene-AT1G	2.152779	3.095413	2.713229	2.506028	2.812394	2.233881	1.547787	0.976205
gene-AT1G	331.3785	318.7936	329.4629	304.4683	305.6744	302.148	439.227	452.7361
gene-AT1G	0.542259	0.807834	0.538499	0.966394	0.736063	1.135457	0.135254	0.363979
gene-AT3G	8.715835	9.301494	10.32783	10.9046	9.547679	9.279206	7.732911	7.589575
gene-AT1G	29.90732	26.88501	29.70454	24.0988	24.03796	24.90811	31.23836	32.8499
gene-AT1G	4.608014	4.405091	4.664731	3.574815	2.866202	3.024488	5.202754	4.436215
gene-AT3G	78.8306	97.83613	76.63493	33.80343	36.47337	35.275	23.66908	23.77487
gene-AT3G	176.2602	177.198	171.4116	112.0658	119.7583	119.2588	66.78668	49.9767
gene-AT2G	19.62926	21.31141	21.01538	27.32001	26.31149	28.71641	9.443949	9.039329
gene-AT4G	29.27665	26.31117	27.59243	24.80708	17.84757	18.92543	11.21537	12.84727
gene-AT3G	2.892594	3.412378	2.694676	6.04376	5.416766	4.341541	9.611362	9.555182
gene-AT5G	8.062922	8.802671	8.807342	4.752457	4.296343	4.451032	5.250544	5.097114
gene-AT4G	1888.38	1874.911	1901.765	2037.669	2076.517	2001.893	756.7873	796.5967
gene-AT3G	0.132548	0.052583	0.170745	0.206477	0.348948	0.17021	0.115515	0.154368
gene-AT5G	5.418504	6.125816	5.660828	2.741113	2.45911	2.381912	8.913299	8.639098
gene-AT1G	1.335441	0.842066	0.948205	0.304786	0.405586	0.667297	1.221695	1.818529
gene-AT2G	3.030658	2.064646	1.700035	2.872194	3.119849	2.681482	1.101314	0.910421
gene-AT3G	6.964641	7.206473	7.916545	8.651021	9.577621	9.559018	3.041703	3.353419
gene-AT5G	13.78063	14.57704	11.76628	4.404616	4.366908	5.588214	7.742087	9.407147
gene-AT5G	2.178047	2.914052	2.316382	1.752199	1.894982	1.944587	0.248506	0.346417
Arabidopsi	0.346783	0.423755	0.524679	0.971733	0.999254	1.063388	0.182339	0
Arabidopsi	4.701173	4.2732	5.600763	10.55704	8.976579	10.11114	2.272294	2.088264
Arabidopsi	9.700808	11.07338	9.014122	14.11952	12.11832	14.6534	3.268077	2.767593
gene-AT5G	11.84785	13.17349	11.11339	6.740754	4.359266	5.156246	16.66742	15.9651
gene-AT5G	4.683174	4.934917	5.891184	13.17806	12.28879	12.31598	0.878903	1.058211
gene-AT1G	2.185193	2.05047	2.48096	0.949666	1.055075	0.612234	2.513778	2.915462
gene-AT1G	1.432103	1.225977	1.399887	3.861964	4.006015	3.623115	2.065278	1.409799
gene-AT2G	3.175574	2.584463	2.685137	13.42397	12.60305	11.07519	2.64534	2.533171
gene-AT2G	3.515156	3.911819	4.569404	8.163135	7.537569	9.039102	2.983198	3.539796
gene-AT3G	0.406502	0.084504	0.216323	1.335533	0.860938	0.875797	0.420123	0.605955
gene-AT1G	3.652685	3.345707	3.988504	4.376513	3.931042	4.061916	4.98237	4.617952
gene-AT5G	39.54356	38.54977	38.62946	35.48322	33.71221	33.02894	62.04677	55.72312

gene-AT5G	0.644991	0.981973	0.680009	1.575885	1.445788	0.922534	0.851278	0.592133
gene-AT4G	2.836007	3.297352	2.777348	2.805917	3.923013	3.424529	0.43596	0.730855
Arabidopsi	471.4394	504.7089	481.4308	624.7054	659.0394	746.1112	3914.594	1848.185
gene-AT5G	0.204069	0.493385	0.339815	0.232443	0.144518	0.302916	0.860899	0.924327
gene-AT1G	0.81288	1.127161	1.244444	1.05265	1.019088	1.081722	0.442079	0.296056
gene-AT1G	0.422932	1.184456	1.18301	0.175925	0.106444	0.111919	1.466407	1.822621
gene-AT3G	17.9338	16.1281	17.53921	46.92953	42.27117	47.43414	25.98151	21.75244
gene-AT5G	34.63791	35.77048	34.39383	22.49354	19.17158	19.44645	36.99443	39.10437
gene-AT1G	61.13144	62.84815	64.69502	79.72258	86.71839	80.13255	26.06093	30.70796
gene-AT1G	145.8515	138.0645	140.2433	53.04552	56.37159	50.55705	130.3015	126.1356
gene-AT5G	5.959738	7.200805	6.946945	5.189473	5.751533	5.003592	8.827186	6.767227
gene-AT1G	25.40381	24.18295	23.58971	7.368589	7.470853	7.6471	13.89114	14.4106
gene-AT3G	0.944575	0.415957	0.981313	37.28672	34.65949	35.72564	0.261089	0.788389
gene-AT5G	0.635791	0.132384	0.378669	0.40121	0.607437	0.675225	0.180553	0.42452
gene-AT1G	30.40541	29.49667	27.6085	15.25786	16.55428	15.55838	9.439459	8.704823
gene-AT1G	1.149815	0.947784	1.11455	2.14588	1.963144	1.524029	0.498871	0.381307
gene-AT4G	7.677972	8.687645	8.072575	4.399104	4.514579	3.847108	1.707415	2.575117
gene-AT1G	4.343336	4.256922	4.543445	5.644082	4.728163	4.121718	1.94945	2.68334
gene-AT1G	21.20332	20.18844	21.04236	13.25262	12.06264	12.51669	39.57584	42.67543
gene-AT3G	1.273114	1.683418	1.650557	1.832607	1.998319	1.577603	2.193186	2.482238
gene-AT3G	1.555537	1.349349	0.830688	0.982609	1.051784	0.903609	0.150792	0.160653
gene-AT1G	0.393963	0.248502	0.470547	0.715067	1.23554	0.945448	0.199929	0.270434
gene-AT1G	280.2466	276.4177	282.7539	183.1949	183.7273	180.353	403.7638	420.3495
gene-AT1G	13.54893	15.8955	17.8519	0.372172	0.05192	0.434855	0.634579	0.086262
gene-AT1G	187.4671	180.046	189.8647	10.84299	10.1633	11.4744	8.084124	7.480045
gene-AT4G	1.127693	0.879606	1.167025	1.278846	1.331525	0.944367	1.049146	1.462927
gene-AT3G	12.85333	12.04502	12.34981	12.08598	13.17532	12.27196	27.83616	29.60222
gene-AT3G	1.228611	0.609933	1.049777	1.478628	1.196437	1.796958	0.786513	1.332369
gene-AT3G	30.89294	26.28084	25.69999	53.81823	54.36032	56.84993	25.19348	22.96554
gene-AT1G	2.690432	2.642569	2.353318	1.410639	1.979663	2.690145	1.457848	0.752891
gene-AT1G	58.70051	61.04334	62.357	56.70738	56.05977	55.70231	27.59797	28.99041
gene-AT5G	0.76492	0.776662	0.734777	1.012825	0.746217	0.858323	0.52415	0.573943
gene-AT5G	1.244088	1.217777	0.929928	1.301556	1.119892	1.527272	0.540058	0.411268
gene-AT5G	0.616429	0.989283	0.436842	1.866585	1.473797	1.875819	0.606444	0.174307
gene-AT4G	173.4775	163.181	173.0895	76.1013	78.97217	77.5992	83.40786	86.55258
gene-AT3G	26.47546	22.67603	27.09114	23.65717	25.27912	21.57853	34.40791	37.39988
gene-AT3G	2.766566	1.728978	2.055296	1.308864	2.086641	2.115282	0.053859	0.249856
gene-AT4G	13.95885	14.14761	15.40358	6.122254	8.272015	6.97403	33.54567	31.48593
gene-AT1G	6.963988	6.213442	6.477013	6.627533	5.405016	6.542708	12.80847	11.7111
gene-AT5G	2.023307	2.771507	2.675214	0.590692	0.698294	0.45082	0.379885	0.444446
gene-AT5G	18.90934	19.34999	18.68309	26.51112	23.80495	25.49754	5.462867	5.235018
gene-AT4G	1.333614	1.347361	1.243144	3.160481	4.635723	4.044376	0.882504	1.15159
gene-AT3G	1.348158	1.894631	1.32506	0.544074	1.532505	1.012207	2.302448	2.849474
gene-AT4G	47.80415	46.47036	46.62233	131.3801	122.9657	135.613	39.54497	34.21172
gene-AT1G	41.78114	36.98408	39.87045	56.63349	46.53657	84.22258	12.38147	6.557381
gene-AT1G	2084.525	2019.654	2154.777	2918.485	2768.729	2890.708	792.8472	741.7775
gene-AT1G	45.59508	44.18733	46.58195	26.45978	27.34343	26.39025	68.13779	64.1936
gene-AT3G	7.167029	7.811055	7.343094	6.084267	5.813829	6.608032	12.07422	12.8871
gene-AT3G	0.622016	0.49173	0.593815	0.813521	0.729178	0.98589	0.468541	0.354818
gene-AT5G	1.647828	2.26798	2.338761	1.36616	2.272028	2.194338	6.973111	6.012407
gene-AT2G	270.2296	280.6597	274.7946	140.3308	143.909	143.4623	507.3727	495.719

gene-AT2G	20.61444	20.36509	19.43604	19.20134	18.97149	19.07015	8.921631	8.847236
gene-AT3G	48.11901	51.34033	46.2074	41.82502	43.03274	43.08126	53.22234	51.172
gene-AT5G	4.475483	4.424124	5.096461	6.18174	5.432051	5.251416	6.526811	7.006714
gene-AT5G	7.448048	8.197491	7.661842	9.967164	11.543	10.52064	6.809388	7.525213
gene-AT3G	2.892596	2.773865	2.620409	4.303627	5.05808	5.344261	2.713202	2.265143

WT-CK3	WT-N1	WT-N2	WT-N3
1.580094	21.01318	22.22391	24.15933
7.138451	17.45799	15.38321	18.59725
1.188106	0.533359	0.195008	0.341187
9.188429	16.49452	14.81253	16.14708
3.713694	5.965235	6.05216	6.845202
6.866393	0.912138	0.875769	1.168998
9.79356	3.776949	4.198458	4.401814
3.028075	7.035002	6.506035	9.328715
10.95208	18.08807	16.9938	22.0541
23.78978	8.743455	7.250249	6.263759
85.18897	46.53995	46.35345	48.74257
3.848434	12.34894	14.48775	13.48283
2.144415	5.653308	7.562604	6.928164
1204.384	418.0146	408.4854	413.9353
6.965593	4.302822	4.109985	4.019821
10.71598	16.65628	12.9744	16.02059
21.40403	10.68629	9.800953	10.88627
101.1265	148.3527	145.1767	143.0506
44.02588	16.50281	14.58011	14.34727
14.23218	28.17127	26.86106	26.67361
22.77775	3.586889	3.272597	3.28369
29.27067	55.63165	56.49978	58.01837
2.114301	14.812	14.89365	14.40036
34.78509	23.31321	21.43975	21.90876
2.31957	2.578663	2.385226	3.024984
0.210069	0.492183	0.750239	0.720151
0.114892	1.611565	1.067477	1.553882
1.187689	1.908821	1.324457	2.479395
0.260805	0.691082	0.603176	1.046335
20.85308	71.12813	69.60673	75.40103
27.78413	11.34713	11.88989	13.42794
2.179886	2.906686	2.854114	2.941813
14.81458	36.41751	39.3566	40.27819
0.198568	0.591324	0.744207	0.461338
12.44571	5.110334	4.87351	5.715943
28.0926	21.67825	21.29495	19.61794
6.111291	6.87537	5.893235	6.389649
0.541783	1.683693	1.133364	1.340043
28.34844	13.13993	11.47733	13.01684
0.780343	1.999434	2.25986	1.91263
4.29431	1.72425	1.687945	1.561686
6.716621	1.304832	1.706767	1.664884
6.362165	10.84412	9.453987	10.44938
0.565791	1.771741	1.452371	1.581643
0.598026	1.502003	2.171861	1.939186
1.134159	1.153253	0.565115	0.585667
25.54123	6.743416	5.445301	6.503312
0.58449	0.215303	0.254136	0.300556
10.19044	4.583169	2.752827	4.183728

10.76723	4.180399	3.826423	6.098978
12.88666	18.9142	17.23435	19.27437
1.041485	2.518357	2.42463	2.620994
28.51034	10.25304	11.89696	13.07055
9.662941	5.240563	6.627151	5.478192
0.087301	1.280229	0.904833	0.974884
2.886456	6.377372	7.422095	7.412966
44.1858	149.0386	148.5661	153.5457
6.640358	21.16651	20.74544	19.97272
0.651965	1.940118	2.05092	2.545501
0.091765	0.98028	0.738162	1.182544
1.149763	7.74752	7.499932	6.428035
1.59889	0.587025	0.575172	0.516415
64.05344	28.83215	26.83363	29.16873
0.604162	2.105874	2.585839	1.734833
5.222494	2.789096	3.254754	3.046992
29.53052	11.41337	12.05351	12.53361
5.391245	15.82339	15.44978	15.53502
1.193159	3.491227	3.145437	3.514067
0.276241	2.091066	2.300471	1.899427
355.0994	160.7204	156.2244	167.3331
67.1116	76.26461	76.42188	76.44555
21.60412	11.94242	9.766793	12.34411
1.250458	1.463907	1.34261	1.795056
19.1256	7.711922	6.466831	7.436601
3.810968	9.762881	9.941942	9.836206
27.02096	12.86261	11.44278	12.88368
0.909342	1.581043	1.828435	1.9915
1.264419	1.706297	2.68187	2.252401
0.291004	0.803834	0.665401	1.331713
6.22276	15.13723	14.21003	14.84046
34.02467	17.31075	14.96225	18.67957
20.99957	125.9452	114.7849	123.3187
40.02082	15.97005	18.09953	17.6912
2.97658	54.58852	53.48797	58.52647
0.081513	2.169523	1.750949	1.573107
142.1167	54.7523	54.2494	59.46762
12.42269	31.33219	29.31446	30.79909
17.55458	8.913157	9.031217	9.076827
25.97859	12.88577	12.84853	12.48473
11.47412	34.32957	32.07306	34.20316
11.45938	6.083652	6.154509	5.175417
15.48702	17.12174	17.584	18.40386
2.402328	4.208667	3.329178	3.964559
6.979887	15.00793	16.57897	17.15652
3.604844	1.330581	1.20117	1.307789
2.421815	0.932769	1.798661	1.490337
16.70664	55.40331	64.87148	54.68792
11.61096	3.179486	1.882297	4.417089
23.76283	113.8499	115.898	119.1032
12.48277	4.88454	4.421558	4.772571

9.221359	6.164013	5.43928	5.595232
2.967092	1.040483	1.160184	1.412293
3.070726	7.67072	9.819048	9.113151
4.275129	36.58344	34.99669	36.22874
0.09744	0.423061	0.197151	0.281537
1.246368	1.552694	1.984386	2.211369
4.007183	1.047078	0.768947	0.87398
3.84481	1.836107	2.010435	1.894515
14.29853	10.10846	9.984091	10.43733
3.968357	0	0.138582	0.312177
0.242514	1.132534	1.142516	0.984769
5.807021	5.889421	6.32138	5.687865
2.954341	2.33099	2.961561	3.70395
0.567643	5.01276	4.606711	3.164791
17.04869	50.84078	47.36429	49.79179
7.571206	2.612258	3.471024	3.789132
23.81787	11.24334	11.44017	12.31191
0.272696	0.752988	0.713667	0.509227
41.60887	23.61882	22.85725	24.83053
12.78804	117.2429	106.6743	118.2405
8.945771	23.52956	27.59156	26.72683
1.866861	1.469058	1.140745	1.053811
24.18395	6.271314	5.673617	5.284925
4.622398	0.833078	0.794481	0.767275
2.425638	7.072355	8.774651	10.54298
1.451984	1.918374	2.517988	2.101495
11.82798	5.067609	3.705382	3.990388
0.314747	4.318242	3.943736	4.480031
25.46702	63.40265	61.88862	66.01335
3.673479	3.354682	3.539353	3.098741
2.821923	0.808899	1.083766	1.278933
4.901212	0.575292	0.512641	0.447638
11.27661	25.16008	22.6201	23.98008
3.016831	21.51271	21.4476	21.787
15.05849	8.783318	7.253763	6.782374
5.656764	7.402404	6.487913	6.896183
0.341367	0.894834	0.976992	0.733312
4.940057	1.249086	1.609623	2.06694
0.288161	0.811117	0.842685	0.814234
0	0.807166	0.801672	0.56528
22.69004	7.374915	6.74721	7.346374
42.82236	3.457349	1.980394	3.777721
1573.087	369.058	381.32	374.8518
1.675192	2.674592	2.601689	1.839986
0	0.506085	0.502148	0.919792
2.104379	0.832792	0.964003	0.590456
2.146519	7.078493	6.897007	7.158031
20.61047	9.777964	9.814594	9.806727
4.983434	2.333691	2.78306	1.69027
1.993266	3.282558	3.457828	2.481825
52.6471	22.84841	24.03694	21.9381

0.344934	1.754947	2.549826	3.037038
11.10159	4.378285	3.861314	4.984124
3.305435	1.253461	1.498271	1.024828
0.572054	3.168455	3.382996	1.741595
31.29733	0	0	0
173.1965	48.51799	50.61079	50.9982
5.249002	13.12421	13.94671	13.71567
37.16579	83.88563	83.67828	85.85596
41.13449	13.54043	15.2564	14.83615
1.196048	0.30617	0.602984	0.548042
0.719036	2.575686	2.275702	2.979581
3.388133	3.062679	3.172665	2.69052
0.056769	6.54253	5.153548	5.760167
2.208014	6.347654	5.418775	5.813152
8.75068	90.14698	82.9525	88.87946
221.4769	117.5089	111.563	118.8192
9.686534	2.059743	2.574657	2.649677
2.522583	6.737116	5.576266	7.327974
12.15619	8.777036	6.347375	6.580629
27.79371	71.61307	67.85889	75.75047
3.581539	7.71134	9.075535	7.781151
2.142024	0	0	0.077159
26.74441	12.71161	8.161426	12.64197
4.821339	25.10735	22.66738	26.57765
0.048253	1.686982	1.66909	1.11157
10.50107	26.40141	26.56731	27.71234
1.372538	4.530298	3.161305	4.308506
28.915	12.39792	12.45875	13.97524
0.010522	0.346281	0.27909	0.249668
2.729122	8.010023	6.506471	7.475705
5.089363	7.281124	7.519882	8.069311
1.240617	2.55177	2.189486	1.882714
10.90867	3.1494	5.445467	5.257699
0.4178	4.676764	4.333599	5.356828
6.847205	31.22492	28.69227	28.78377
10.52939	4.583001	4.451659	5.444921
2.084201	3.911128	3.760115	3.393002
39.38233	13.59361	14.98508	11.48828
14.21796	5.841184	5.152223	6.379891
4.820343	0.481235	0.481459	0.649065
1.167606	0.78448	0.407995	0.726042
11.63226	4.79147	4.219519	5.773403
120.0809	86.58146	88.86054	88.26088
1.633082	1.005762	0.975093	0.941978
1.347056	6.110743	5.333961	4.794954
0.577272	1.402305	1.393741	2.115503
0.12609	0.699167	0.818761	0.652163
1.845309	4.671575	2.604558	3.872084
1.720656	1.948936	1.76439	1.931395
16.39937	9.204365	9.887789	8.916149
4.345785	10.4768	10.17163	12.42741

86.43811	46.00851	45.98033	48.12122
1.392991	3.89025	3.903044	3.710304
8.896835	21.92998	24.7585	21.02405
1.114903	6.037152	7.864502	6.451856
3.535724	1.153204	1.555583	1.278581
9.350565	4.056006	4.004995	4.771603
4.041667	2.195469	1.672841	1.894042
0.633417	1.918096	2.006472	1.683769
40.65064	5.621246	5.720293	6.270016
1.118417	3.352417	2.776677	2.717042
1.02265	3.13609	2.858649	2.881477
5.153481	14.60433	14.32008	14.73924
0.207747	1.431008	1.174265	1.846152
0	1.28261	1.568912	1.262979
0.245713	3.993795	4.191137	3.959686
4.87332	4.999982	3.249759	3.763027
1.206864	2.65334	3.318442	2.537844
45.30948	20.98757	20.19666	19.93503
0.896681	1.275501	0.957093	0.971719
1.149961	4.515513	4.60376	4.076403
2.904835	0.616929	0.984628	1.787613
36.32531	22.74193	23.69265	24.66781
0.232631	0.507728	0.655829	0.532304
0.094755	0.499054	0.414907	0.521145
6.624695	2.755123	2.954689	3.409674
4.749276	8.243067	8.549498	10.6705
2.284021	6.506429	5.846977	6.662316
0.18169	0.26693	0.053412	0.343202
5.29711	2.735335	2.85852	2.64856
5.10885	4.464015	3.802195	3.26439
74.41367	32.24825	31.14553	32.79433
254.4178	106.6641	102.9559	112.4885
0.451468	1.248347	1.459962	0.815167
67.04855	3.055655	3.07498	2.832334
8.589823	4.65617	3.898461	4.112883
5.315238	12.0877	12.18439	14.02273
52.77268	25.63987	24.28212	28.43256
61.34797	20.88537	22.63398	20.7856
0.998175	0.655219	0.490598	0.210465
512.4297	109.3128	113.029	112.0613
12.2367	32.54369	27.47194	30.59548
24.96444	42.95861	38.87899	46.19296
1.130802	0.313795	0.35504	0.701535
124.5897	55.4176	59.24521	60.21116
13.19734	43.67558	42.19763	45.19762
2.198046	0.961503	1.202527	1.10096
74.63313	18.42243	18.21129	17.69441
2.167437	4.682421	5.215818	4.755806
0	6.981932	5.906566	6.43787
4.989776	1.909562	1.799798	1.398448
17.95627	7.559157	7.750183	9.16837

0.535073	4.125998	3.634881	2.637213
0.301183	1.491897	1.329832	1.034192
2.012933	5.529639	6.621721	5.805214
60.55701	33.88672	29.51556	34.1507
13.048	37.7815	38.39822	47.02096
68.50866	32.27842	32.26101	35.61961
5.741446	16.85614	17.14401	17.33258
248.0189	19.9578	15.85515	18.20974
1.062002	3.81337	2.963421	3.088639
16.67761	63.53644	60.43118	63.15414
11.35772	2.380514	1.531232	2.549772
5.958241	2.980621	2.763823	3.230141
251.489	113.6369	113.0802	121.2003
146.6692	10.12708	10.51597	10.48864
5.243984	2.651558	2.612048	2.354761
32.18946	26.49332	26.76463	21.88217
0.541094	0.082072	0.124619	0.073012
55.05301	18.61204	18.26623	19.53525
5.65162	5.215239	5.170722	4.361308
58.84648	19.47731	15.10763	15.42795
0.305303	1.725907	2.275799	2.652739
10.6681	3.564075	5.053602	4.348782
12.6243	5.812807	8.979147	6.806155
13.93869	4.959443	5.436895	5.048671
167.9998	51.30622	49.75073	53.45601
0.23772	1.122962	1.987896	0.854736
1.979536	0.888916	0.883386	0.933725
2.241459	8.821896	8.368515	8.17844
1.670864	2.101001	1.977029	2.827246
1.188104	1.982556	1.986118	1.190459
2.933854	1.511327	1.18328	1.439858
8.756952	25.49748	21.70047	23.88538
6.510759	22.68829	23.13457	24.40952
3.367141	2.129697	2.332727	1.582333
10.99568	26.37979	25.21165	27.94774
15.85556	7.716428	6.296746	5.958678
1.759986	2.425642	2.082996	2.222705
16.41274	8.199852	9.447588	8.921775
0.350103	1.118341	0.935857	0.937691
1.220542	1.266012	0.736435	0.770511
238.5057	71.63363	71.16251	73.3159
0.30503	0.694967	1.111559	0.843135
8.656039	7.504327	5.710956	7.304633
15.96456	4.640457	5.075553	4.519534
33.56986	11.69953	10.41983	13.00724
200.7815	75.65195	75.07976	78.87508
4.532731	14.3627	16.4413	16.09843
0.687982	4.839293	4.776556	5.795307
1.447515	1.967575	2.196539	2.166394
3.765293	1.803073	2.150859	1.382245
13.81507	4.301538	4.98157	4.569405

6.130992	8.722777	8.371716	9.387063
28.50555	14.50536	13.1704	14.18149
1.440188	1.552413	1.145212	1.791756
4.717755	11.25396	13.21207	14.17536
3.186441	7.991548	8.05368	8.192467
0.532204	2.681621	3.317249	4.015183
479.1055	193.0203	201.3974	209.6415
5.369145	15.59267	14.37923	17.71211
15.57562	72.59328	76.25763	72.33654
0.060907	2.621838	2.49368	2.358998
0.831202	2.452542	2.709632	3.655646
5.885705	20.79877	20.519	22.15193
3.118233	9.459585	7.996352	7.740656
106.7748	29.54779	30.48478	31.19904
3.759263	8.128043	6.720434	7.534355
16.18607	0.460516	0.276696	0.30078
15.52221	35.28954	33.12141	34.48162
0.24995	4.932568	7.442893	6.834869
84.18847	29.69592	30.41027	31.69114
85.64777	34.30019	35.22435	33.90041
8.545641	4.10904	4.3599	4.379506
0.355478	2.851292	2.530766	2.719407
14.35092	48.3912	45.78089	48.25559
145.4149	367.3908	373.325	376.3438
7.178557	3.620381	3.301244	3.193497
0.265943	17.63336	16.48907	18.01872
5.056655	2.882344	1.738665	1.361307
1.247584	2.336244	2.810126	2.481017
382.2629	110.8073	101.8936	112.799
2.666465	1.425683	1.093487	1.212302
32.30822	14.70454	16.00802	15.30453
0.492981	1.011599	1.168709	0.89093
3.990217	4.378767	4.386785	5.122278
135.6396	52.8713	58.15287	55.76864
6.991452	3.03128	3.354977	3.298856
4.520684	14.10343	17.00561	17.26124
17.98651	3.042617	2.452426	2.288515
54.81506	27.62882	27.3439	27.72068
16.01835	7.842248	8.338629	8.075739
10.29043	26.43356	30.13625	29.37017
1.072441	0.308625	1.401244	1.036906
1.817147	0.650363	0.63698	0.769536
3.15688	6.813566	6.421132	6.048584
0.157935	0.577422	1.777776	0.455326
136.6195	26.64654	27.22483	26.48988
0.102697	0.737384	0.540681	0.536216
90.30205	750.8151	744.996	755.9677
8.87147	2.596357	2.693663	2.711562
0	6.649576	6.828903	6.717684
0.170894	1.536634	2.544459	1.917202
10.00716	28.50109	26.66817	31.46993

0.3415	5.753341	4.984692	5.879144
50.42646	0.99552	0.782741	0.758564
0.912965	0	0	0
0.853053	0.087926	0	0.15129
26.53731	9.594739	9.588515	10.4081
0.246261	0.034455	0.097114	0.063481
2.033733	4.372097	5.309405	4.637659
11.27397	2.955807	2.075268	2.546469
16.31045	6.367414	5.5714	7.219671
4.587037	17.04041	18.6537	19.32352
0.408769	1.42938	1.32217	1.749918
48.53611	23.44488	22.47352	21.23498
54.35811	10.13	10.47522	9.572066
15.8854	37.33756	39.66394	39.07269
4.206642	2.143996	2.027141	2.034616
0.496132	1.776326	2.672452	2.05902
30.68636	13.6709	12.27259	13.95674
1.543072	0.61161	0.588767	0.822069
29.29703	6.790389	6.660825	7.111744
0.955941	3.381274	4.084806	4.453528
12.83449	3.676866	4.219088	5.115535
4.547422	9.385428	8.804506	10.27466
55.52357	98.43879	95.78482	107.974
0.321615	5.665912	5.705305	5.529855
144.399	34.12022	30.05532	33.9207
1.5704	0.40798	0.133626	0.12566
85.67446	365.1076	294.7776	347.4117
46.31246	103.3305	100.2242	108.6144
9.47647	25.61223	26.53599	27.07325
0.779286	1.098025	1.907432	1.276435
4.377801	4.58812	4.422835	5.131716
0.472448	1.392647	1.257031	1.798133
8.928776	5.511795	6.732809	6.520379
1.882101	4.996551	3.344596	2.805243
4.951506	9.846325	8.408827	9.446654
2.502924	19.62501	18.72574	18.70132
94.63533	33.06468	30.99603	32.55421
6.57388	18.18895	18.48776	18.07289
0.810623	0.285028	0.652817	0.575318
0.763609	4.32549	3.890039	4.619976
63.2968	123.4269	130.5235	131.0047
1.284241	4.437664	3.694691	4.453636
1.464842	0.931337	1.042654	1.476266
0.351237	2.427913	2.618293	3.646074
0.507958	0.227784	0.333465	0.528502
20.84885	109.9062	106.2129	114.969
14.33041	68.24007	68.6882	69.62018
1.775307	3.894364	5.077695	5.459699
73.49588	49.70191	54.18633	50.20131
0.784104	1.382112	1.165861	1.642802
2.777025	3.179035	3.000692	3.224684

112.0013	15.34198	16.89069	16.1489
0.842808	3.755735	4.325458	4.173595
53.299	51.93338	48.36728	52.95643
0.297262	2.346711	2.023059	1.897571
239.5841	113.9354	1128.988	16.34059
2.316468	0.849855	0.864683	1.00275
15.63855	55.98999	56.8611	58.7194
3.923966	17.54005	18.27739	20.00387
0.231586	0.678386	1.389966	0.818249
57.08983	22.17737	21.80749	23.23264
0.713439	0.258131	0.554852	0.876749
34.09007	13.19755	13.51147	14.26522
5.535371	1.274989	0.994233	0.928777
0.482399	4.41512	4.916445	3.761672
30.72594	13.49192	13.45295	14.61686
2.463116	5.718378	5.221088	5.372788
0.089055	1.139533	1.316389	1.295611
0.413829	0.799177	0.635342	0.415571
1.756555	0.66995	0.301599	0.550094
109.7956	426.7952	418.2242	434.3916
3.659127	7.617711	7.144359	6.943997
26.52432	14.11639	13.55768	13.91013
0.504457	0.40065	0.602158	0.469706
43.1458	19.86672	21.86079	20.93039
0.028578	0.448176	0.457226	0.649701
1.066869	1.408552	1.81331	2.411227
15.05998	4.931042	4.902911	5.85167
31.38832	12.44976	12.17404	11.29764
0.48291	2.25708	1.914377	2.035779
6.726517	11.8881	11.21401	12.05371
1.501756	4.751679	6.96061	4.139233
17.65286	6.579654	5.508954	5.45464
7.443119	17.5336	19.22535	16.84824
31.65409	13.509	14.31143	14.49827
3.449227	1.688709	1.296022	1.594089
32.01053	3.566895	2.595461	3.184832
51.43723	21.87353	19.83179	25.2115
19.70477	4.993022	5.689523	5.535155
8.80531	88.15525	79.48365	86.87454
9.765158	1.794415	2.499506	1.997502
1.03989	0.573132	0.469865	0.739264
2.335432	0.91492	0.536229	0.599822
6.949992	3.457205	3.303609	3.78911
3.079855	12.57974	13.93621	12.67134
30.89121	16.07571	15.47873	13.79405
2.913003	6.318584	6.089821	6.497606
14.64769	36.17893	31.93684	33.69384
146.2442	44.0292	46.74933	44.454
2.113483	4.401447	6.837144	5.763094
28.13852	11.17253	9.22972	12.05182
61.32173	144.6676	141.0043	145.0784

1.497751	14.86591	16.46847	18.25376
11.06599	9.267904	10.92204	9.762119
1.064391	0.475975	0.33123	0.40284
1.57361	7.75841	7.23408	7.896688
12.75221	33.03368	36.52126	36.3643
5.326041	1.587806	1.542111	1.130716
11.34292	4.39221	3.713494	3.756807
7.466419	17.67713	17.38774	18.88766
18.75103	50.1484	50.78324	51.16929
18.11814	53.11287	47.14106	54.37254
63.29637	31.89119	32.85396	34.64539
2.474918	0.685771	0.745545	1.201417
19.72173	10.37154	9.782816	10.72928
13.81513	32.31122	30.64228	34.73168
13.37473	13.72035	16.61192	14.66601
2.583345	9.403868	9.005728	10.00298
0.469314	1.333063	1.415029	1.077831
15.21125	35.53629	38.4505	38.72704
1.757802	0.157025	0.146767	0.23153
1.560301	3.274992	3.032843	4.014044
8.426717	16.87405	16.10802	17.51261
11.63758	4.588514	3.153054	3.795545
42.40207	56.22451	56.32067	64.31896
7.415133	2.274988	2.285639	2.480406
0.677211	3.066072	2.419419	2.839472
1.456329	4.610455	5.061828	6.38072
8.265395	12.88425	11.07699	11.04448
2.553279	125.0458	120.6188	128.1947
37.34675	145.4421	141.2139	143.6884
5.229597	2.057244	2.237959	2.559994
24.59005	4.432757	3.468728	4.47947
29.10164	63.59841	68.33515	72.91656
8.877846	10.17411	10.29477	9.313529
0.488325	0.77046	0.737259	0.718863
2.128646	3.082302	2.303121	2.798955
4.819284	19.2368	19.84501	18.84924
0.279855	0.728577	0.518135	0.913976
10.21683	9.248268	8.570847	10.33536
0.445378	0.924776	0.959749	0.713862
30.87658	9.885262	10.73561	11.26862
1.392131	0.450393	0.959253	0.115389
2.702202	1.379623	1.083509	1.248855
20.4306	4.387246	5.706882	6.402215
3.053725	8.631825	8.691521	8.508975
0	3.623061	3.708567	3.297654
12.38336	23.53508	24.05274	23.79682
32.56934	18.531	18.24683	18.33952
0.461132	1.007431	0.752661	1.322091
3.621364	6.638549	5.337111	5.974247
0.786066	3.008549	2.297517	3.342103
2.919473	4.962456	4.25876	4.924898

19.87381	47.58715	47.11831	49.05135
6.84986	5.903206	5.564155	5.354397
15.50947	34.65878	38.28426	39.12427
4.803571	2.537432	2.874371	2.731711
1.999468	0.230307	0.62136	0.535038
8.24303	31.60357	34.80573	35.92828
1.797367	4.726829	4.589243	4.933285
1.761941	3.379112	3.605956	3.806694
1.977754	1.291925	1.350169	1.75237
36.41337	12.98014	13.40712	13.42883
6.852715	11.75379	10.81393	10.78688
1.433081	1.492247	1.259768	1.238281
4.445235	25.77852	26.79986	26.03977
38.8697	19.72722	20.40163	20.94622
2.123496	0.043278	0.247371	0
3.625553	1.647269	0.860942	1.168236
10.07159	25.61795	27.89117	25.71645
1.866586	0.642416	0.617676	1.236111
2.612773	9.329312	8.2066	9.405402
14.47931	15.59325	14.50197	15.37757
2.700189	1.045521	1.340952	1.45148
223.6527	64.67088	64.28805	65.55246
0.760439	0.436995	0.810707	0.602172
0.629312	0	0.117484	0.305891
548.799	739.4016	690.438	740.2934
1.312635	0.626124	0.427269	0.545142
7.929611	14.32343	13.94043	13.58674
0.77266	2.873164	2.412405	2.451752
12.10397	37.3923	34.10364	39.03374
41.60497	18.8915	19.59977	19.50805
6.070101	14.70974	16.89926	15.24835
19.88899	48.06197	47.8912	49.42893
0.448587	2.098038	1.868451	2.145342
0.91988	0.625547	0.766497	1.313972
4.756174	3.147922	4.203616	4.0739
1.627077	0.605248	0.217234	0.303357
56.30868	17.3325	36.60685	13.92005
8.322111	3.019696	3.427513	2.192437
7.580041	3.670163	3.485371	3.804259
2.735963	1.364503	1.206541	1.910819
2.773312	4.265856	4.248845	3.346515
6.516936	19.85357	16.78149	18.48781
27.15967	33.36526	31.45427	32.37818
13.97472	90.00991	84.85254	88.91046
30.85489	14.41246	12.71877	13.70561
1.527286	4.705035	4.562586	4.862632
12.59413	5.484721	5.057618	5.137708
3.269711	1.932645	1.888016	1.614236
4.065906	1.088912	1.588717	1.625361
11.13396	5.034633	5.380031	4.785226
2.3053	1.357377	0.796309	1.453321

293.8908	84.58807	92.83421	87.65614
82.42885	251.8218	255.11	268.681
9.299936	28.42595	30.82781	30.09671
2.617937	6.158596	5.424043	5.121031
16.87766	24.38669	21.1297	24.3366
410.7177	40.88726	42.54459	40.98052
12.03835	3.987681	4.037105	4.305764
0.726009	12.24694	9.4935	16.52736
74.41931	33.68475	34.78095	38.25012
208.3769	426.3395	412.7054	432.8838
0.578618	0.334327	0.240589	0.30944
14.80467	36.77728	38.08261	37.52542
854.6064	222.2173	240.2252	241.6777
2.633198	6.781574	6.5006	6.67305
12.22415	25.97852	25.90002	25.87414
1.305593	4.005894	3.828678	3.982508
2.627773	1.000686	0.27697	1.085543
2.884624	14.51299	14.09969	14.148
13.23367	19.31815	17.91422	19.97641
3.342506	3.015231	2.494279	2.538048
1705.583	537.5203	534.4902	564.2422
30.64958	7.355093	8.587679	7.626832
10.43217	4.89326	5.027156	6.589753
3.115722	0.9088	1.687281	1.618419
2.349992	5.473187	4.905654	4.97407
10.99353	2.977445	2.875511	2.768819
21.15866	9.331197	8.865732	8.836544
4.501912	1.618409	1.328197	1.73448
2.099286	6.203988	6.684779	6.647067
0.036851	0.613799	0.760738	0.46922
11.33334	47.70502	44.40672	50.75773
8.164232	3.356778	3.312364	2.940621
16.30681	4.718781	5.683703	6.335489
1.25553	11.06613	9.902748	8.93169
10.11859	4.104891	4.27334	5.532432
2.876073	4.726902	4.383557	4.772243
1.317349	4.992683	4.832959	5.069803
0.541977	0.688655	0.869119	1.265641
0.369496	0.143402	0.545215	0.417955
2.242887	4.236178	4.118475	4.918891
6.509194	3.069607	2.818239	3.267949
7.561782	2.930369	2.164148	2.701883
21.20904	53.02502	48.75244	52.5
2.374442	0.745051	0.792239	0.780623
2.495433	5.818012	5.570611	6.19322
7.873381	10.25521	10.41914	12.30267
2.39287	0.664306	0.484666	1.427799
1.934502	0.463409	0.338715	0.41661
50.41566	16.11298	16.90773	15.53529
2.867415	1.700538	1.170405	1.594397
2.236795	9.254241	9.570964	9.953083

15.22983	48.27817	44.54012	48.37573
2.824451	8.725801	7.836433	7.448027
3.444998	16.54128	15.89563	14.62525
57.3706	28.30234	29.05439	30.43562
4.63074	16.08283	14.2068	16.8059
1.173404	4.377769	4.702985	3.671202
24.57911	10.29689	10.36014	11.73723
52.08131	21.00214	21.46614	21.38215
23.43691	8.157531	9.454854	8.882483
51.24942	21.77919	20.07929	21.86271
26.32002	12.46641	10.40952	12.46986
42.08199	13.73151	12.79439	16.9013
4.949636	4.81158	5.233894	4.561686
2.024146	3.230527	3.869577	4.690918
12.96707	7.349532	7.018842	6.700246
4.507543	6.811601	6.222045	7.406743
57.41518	89.11478	89.4315	90.09904
0.467799	0.817613	0.92273	0.977359
1.8696	8.631473	9.279243	8.007162
7.474116	3.593773	3.30599	3.681553
22.53852	72.0276	60.31205	72.32248
0.050895	0.292446	0.236981	0.25004
6.057447	2.321596	1.810967	2.599752
1.101929	1.728389	1.787228	1.557337
1.040139	0.195849	0.123795	0.463541
1.359471	3.924049	3.408632	3.747249
3.795181	3.810092	3.76366	3.572532
2.616689	0.200022	0.202242	0.225306
1.736705	8.729722	6.590449	6.30052
4.798087	1.882868	2.527513	2.274364
9.084661	3.541164	4.156365	3.664614
3.535661	1.063171	0.948318	1.284686
29.04335	14.66011	14.34969	15.35885
3.420348	0.747221	1.083886	1.110087
5.089877	0.257305	0.377491	0.157569
52.92605	16.60207	16.28226	15.36567
56.34449	21.02474	17.46222	18.44147
7.763091	6.985707	6.227705	7.389137
0.438822	1.494791	1.621316	1.973015
29.10457	57.85837	55.71531	54.29986
4.778186	3.925756	3.021586	3.657345
1.125023	6.671811	6.118955	6.307038
0.167855	0.170558	0.36298	0.169877
0.262883	0.677126	0.58342	0.63221
23.05496	8.677645	7.612231	6.179685
1.209981	0.195917	0.152636	0.149973
430.6028	237.6908	236.5806	235.3713
0.29824	0.147128	0.04449	0.177898
11.4473	16.18811	14.73025	18.13145
0.14213	0.449425	0.709771	0.923434
7.417104	2.712854	2.023764	2.920487

31.26562	8.747049	8.876614	8.031325
29.45101	15.69988	14.8921	16.19633
0.952728	2.174963	2.502698	3.253911
2.267743	1.143972	1.106436	1.294084
16.10358	5.23504	5.067142	4.653909
3.742484	29.10708	32.80021	33.45361
44.57195	19.93466	21.94417	22.0891
122.9523	37.90505	34.37457	38.38891
1.456285	4.328796	4.388117	4.408727
0.660324	1.782979	2.312491	1.562144
40.13982	12.93425	12.4761	11.89586
1.802939	1.497293	3.444756	1.067851
1.210761	2.593488	2.653592	2.157166
0.984557	1.018661	1.17023	0.778241
5.181685	2.376922	2.445211	2.625022
3.95989	4.340366	4.127658	4.112983
8.014643	9.263546	6.625352	9.67384
1.511019	1.023309	0.823985	1.215172
3.808059	10.63457	10.47723	9.973802
0.745077	3.75699	3.994862	3.502921
0.327673	0.54293	0.634354	0.478858
5.149669	1.958565	2.783429	2.437515
1.428453	3.299053	4.105644	3.885433
6.580393	14.54698	15.57841	13.0903
0.184209	0.590772	0.805842	0.632035
0.454218	1.984682	2.021146	2.674794
4.793686	16.52692	15.8121	18.79668
3.651431	0.279705	0	0.129052
4.37663	2.324737	2.488336	2.862535
0.171697	1.233108	1.739556	1.618094
0.959326	1.946684	2.411492	2.436392
0.731877	2.101949	1.685484	1.711936
36.78014	16.37515	18.2995	19.02259
69.24897	19.74408	20.03953	19.01731
7.87321	6.283389	3.905923	5.353976
25.78927	10.35177	10.68748	10.91986
32.20939	13.2016	12.40494	13.2052
20.11297	8.325321	7.974913	9.250431
0	2.326438	1.856846	2.489369
0.858047	1.320055	0.958891	0.913389
9.819755	32.88435	31.4546	33.49976
18.46676	35.69669	37.17304	35.43726
1.755358	1.156165	1.077526	1.046645
68.53027	35.7869	37.50359	40.09186
0.292984	1.015124	1.257661	1.401182
19.82408	9.175792	7.993037	7.343938
21.54905	8.456167	7.040491	7.392
29.64112	11.91104	10.8973	11.6116
0.583735	1.153325	1.061226	1.337796
1.367491	3.160384	3.248658	2.699921
11.57132	3.668906	4.611703	4.52251

6.247102	1.088642	1.685343	1.922211
1.114323	2.526203	3.111123	2.980577
1.283955	0.718826	0.337358	0.341648
12.71808	34.26502	36.28306	36.03685
30.45991	14.30622	13.30963	13.81042
3.049355	2.779922	2.608988	2.864191
114.6248	46.5388	45.62983	52.21359
0.449327	0.513247	2.230713	0.147647
0.309583	0.822787	0.842523	1.194289
0.773978	1.565661	1.41373	1.660627
0.187832	0.975481	1.297079	1.307722
0.912984	0.867942	0.876684	0.735862
0.601006	2.288998	2.595023	3.504677
2.610277	12.15865	13.05167	10.02466
20.53539	64.36156	61.63942	67.62902
3.697406	1.047876	1.360257	1.262121
5.305797	13.34545	13.68438	12.49164
8.593758	15.32614	16.18589	16.21216
2.51808	2.043165	2.273071	1.28037
9.162673	3.080983	4.138617	3.996978
3.743119	25.79785	26.72021	26.19369
4.919032	2.189639	2.616097	2.703958
5.463292	0.399805	0.451004	0.830172
0.145146	0.359914	0.260815	0.209522
100.0721	168.9388	161.3545	170.8912
55.61273	15.05704	13.47398	15.92837
1.305689	0.993707	1.105618	1.008838
0.228262	1.015861	1.164397	1.212128
11.61976	84.72418	81.02425	92.00305
9.782728	3.389307	4.160969	5.141798
10.79103	5.382998	4.851773	4.596949
22.81703	11.05397	11.62262	12.74436
8.232704	19.50715	17.16271	17.15629
1.24227	0.64236	1.087066	1.060015
0.140609	0.510031	0.707867	0.98457
4.613025	88.38535	84.14985	91.93943
0.369457	13.28502	14.20834	13.26989
13.7466	9.252584	5.661588	7.476465
6.276013	3.028967	2.674997	3.045886
2.005309	0.772819	0.728183	0.566779
3.396773	24.70612	23.46561	20.76193
199.4654	82.81392	85.21544	88.99649
635.3718	126.6783	120.7346	126.7053
9.124792	1.142685	0.323734	1.124113
0.938779	10.79964	9.584047	11.25779
0	1.619128	1.569936	2.129288
0.552174	0.439314	0.277377	0.173043
3.295369	12.12636	13.56067	14.49071
0.632217	3.757296	2.308346	3.014484
12.57977	6.910013	5.979162	7.492908
1.452783	3.540369	4.53062	3.228958

6.183345	1.783776	1.356583	3.230449
3.515544	9.431049	9.544508	9.949409
1.234298	1.443135	1.285468	2.388762
65.91592	42.13913	41.40743	39.98481
39.74689	99.51534	93.05389	99.01613
1.562711	3.385185	4.146816	4.566302
59.67767	19.86733	17.62471	16.73658
3.159509	9.901914	9.448565	9.414317
33.20373	60.73177	58.77229	61.2934
1.565958	2.497389	3.183585	2.992064
85.53336	21.78613	19.27673	21.80892
33.53026	3.182426	3.284232	4.041367
0.782338	1.915119	2.853233	2.113091
7.759231	2.733968	2.745056	2.745802
0.765954	2.387019	1.754002	2.059974
1.625754	3.09057	4.346597	5.180698
0.287577	2.425434	2.314757	2.46147
3.957686	2.323101	1.981265	2.426432
21.11685	12.75545	10.10537	12.24662
0.971649	21.5007	13.90664	10.89924
33.4526	84.60955	85.31273	81.06933
44.24596	7.696945	7.407378	7.098218
9.665958	2.713116	1.567103	2.067739
2.229441	6.324002	6.844395	9.460469
7.698942	4.524683	4.005662	3.486133
0.062482	1.392056	0.959993	1.395058
3.426059	6.336041	5.283792	7.269665
1.43101	6.301603	4.004059	3.881206
1.635764	0.927355	1.01551	1.045454
3.433146	20.50812	21.20916	22.78065
2.889421	7.448895	6.326164	6.154204
12.46353	46.60312	42.4198	49.61893
0	0.710959	0.258104	0.346572
103.4926	50.91493	51.39639	51.89516
0.768227	5.554872	5.380331	5.562239
1.741939	4.600676	4.360351	4.517758
532.7345	676.7211	3488.774	252.9465
9.129787	4.264076	4.65414	4.964152
4.436909	6.589987	6.097445	5.629826
0.490221	0.404556	0.143438	0.188112
52.85165	104.2018	96.43033	108.2332
26.66861	9.682482	10.79381	8.440721
0.446821	1.918562	1.747206	2.001152
8.598693	26.53525	25.90259	27.46168
2.329709	5.409645	4.999299	6.343604
0.471916	0.351154	0.158501	0.411415
6.398807	22.61512	20.42845	22.40254
2.899247	1.696363	2.597759	2.28661
5.325989	6.764248	6.268874	7.02359
13.32108	13.81496	16.68656	14.78912
0.568339	1.415253	1.700103	1.66828

11.41572	3.267508	3.07765	2.490837
0.596442	1.247407	1.297138	1.325572
0.381764	2.857432	2.912142	2.894776
170.5793	44.94013	39.63629	45.05928
2.179992	0.663306	0.732742	0.34508
4.240739	1.124162	1.04372	1.478037
0.663012	2.164725	1.613827	1.727277
4.458673	8.719069	11.18337	8.884158
10.78657	10.9088	9.897779	9.831927
15.62363	5.982673	6.931429	6.983521
0.483764	1.146263	0.817098	0.842978
7.519105	3.191108	3.468531	3.150712
25.38224	39.7551	39.8509	40.14432
0.280286	0.914631	1.319168	0.682122
60.04539	9.361029	9.071824	10.61848
22.48904	23.76859	25.39442	23.82466
2.752358	9.791181	10.07101	10.3212
9.135048	1.845258	1.979779	2.441943
0.106231	0.642558	0.540462	0.445752
30.7125	28.21275	31.68247	27.5381
20.82191	6.638432	6.59141	6.543054
5.948236	2.032018	2.598176	2.554347
0.458006	1.818739	1.545414	2.212385
0.191335	0.902873	0.302179	0.643601
2.967186	6.599396	5.743391	6.472666
4.200151	1.602947	2.062322	1.526165
1.62276	1.651219	1.962299	2.662977
8.428451	1.897381	1.879759	1.920083
16.31841	5.78944	6.072067	5.34707
32.24344	15.41557	17.123	17.5966
34.15949	72.24307	68.04792	74.27752
4.344998	0.158058	0.223161	0.265802
3.994625	16.54939	16.36341	16.24818
14.63225	31.55966	35.24402	32.14919
0.694145	1.7934	1.828201	1.876543
0.685446	1.218822	1.904249	1.426063
8.408925	3.754961	3.670519	5.046577
4.35368	2.351471	2.897391	1.10914
72.51906	26.99039	25.9018	29.70824
3.243023	1.042844	1.235812	1.09184
15.59644	3.857866	3.215282	4.822063
18.23859	8.617771	9.83333	9.480837
3.333088	1.464626	1.598452	5.389181
1.402902	5.15697	5.283218	4.80141
250.9469	109.9999	109.7413	112.2096
12.9563	7.039283	5.852612	6.709594
50.91161	21.93722	21.58349	23.11482
29.88021	14.50483	15.44471	16.11771
1.382882	3.194046	3.111161	2.49863
1.158606	0	0.159292	0.47931
11.09592	16.98405	17.85792	15.62678

1.951855	8.40821	7.305643	6.760293
9.589801	15.1283	16.27366	16.81298
4.503147	2.846447	1.281907	2.179439
42.48082	3.109811	4.127958	3.704992
3.974745	1.911528	1.911145	2.575936
3.241508	0.49952	0.991348	1.292682
43.79654	12.72268	10.12064	9.41423
8.151782	32.64549	29.96594	34.61144
70.60773	32.45053	31.73388	35.88072
2182.348	517.3663	492.9647	532.6954
1.518045	6.144994	5.370648	6.034262
7.133463	2.867684	1.947802	2.981488
21.31866	50.92444	49.71596	55.31638
0.248656	0.829109	1.00732	0.658947
13.99765	6.448639	7.537504	6.663749
50.61841	7.568208	7.664351	7.737459
11.98492	9.594131	10.14339	9.989897
0.485485	2.155647	2.748471	2.792497
1998.317	301.3535	296.4619	304.3444
2.787399	3.185335	3.638147	5.906831
2.033163	0.853753	0.487034	0.893379
0.39235	2.299072	1.50241	1.862278
0.974655	1.436014	0.874556	1.176545
0.758515	0.459106	0.664831	1.10737
48.85168	12.79873	14.13751	15.26793
1.323654	0.640507	0.532405	0.847442
27.93254	14.09298	13.69944	13.91757
0.287327	1.363504	1.663845	1.690931
4.589464	6.269043	5.504262	5.94576
18.49092	5.762956	6.344011	4.506475
1.624777	7.706635	7.169679	6.213478
11.021	49.31178	44.73795	35.97491
7.830817	19.47883	20.776	20.5755
37.35766	7.804673	7.802514	7.999638
3.195924	1.573929	1.904008	2.402687
240.3526	66.61408	71.75485	72.54333
5.74643	6.373461	5.555155	5.103421
1.926935	1.217914	1.041844	1.09197
3.205491	1.434692	0.990312	1.127792
1.711661	1.18657	1.534032	1.292809
8.629546	10.81028	11.17224	12.25645
4.318899	2.689801	1.038988	2.052304
131.6657	43.93375	43.92418	45.84535
9.850934	10.30266	11.71921	11.67747
13.73218	4.787944	5.926248	4.434032
1521.619	199.6332	190.2341	197.0118
3.786505	7.926053	10.42053	7.940065
83.29812	92.77827	90.044	94.25657
18.52802	4.311295	5.332904	4.019253
7.69162	4.906532	4.738519	5.625443
151.7729	460.5725	468.2414	455.0367

2.895552	5.083154	4.928361	6.312597
78.43611	29.84519	24.90732	28.27114
12.98513	7.012779	5.789674	6.611075
0.199724	107.5948	104.0537	101.872
1.394647	1.927752	1.729095	1.781401
36.26335	17.05244	16.4992	16.88424
14.09203	7.476717	7.665991	7.848354
28.36103	5.592084	6.132286	5.132943
78.93936	133.5297	128.967	141.4628
3.238648	8.950845	9.024194	9.556555
131.1716	57.3485	59.95314	63.18136
3.502269	7.262938	6.260118	6.29254
12.29056	30.68463	31.31785	30.76878
2.780194	5.691972	4.79367	5.064552
39.62208	18.87654	18.149	19.45293
82.9391	22.55315	24.10859	24.82522
2.438243	9.480618	8.500732	8.757595
18.89608	6.868026	6.52585	7.327421
2.181491	16.8176	13.65002	15.73715
0.42524	1.114847	0.964259	1.323774
16.03974	39.8727	41.14884	42.72492
8.062101	17.57027	18.88773	21.35511
0.480105	1.192498	0.94813	0.960465
0.920517	2.664181	2.477105	2.195364
0	0.538278	0.71358	0.918174
37.57079	14.48707	12.2349	15.68445
0.387546	0.654432	0.991292	0.729354
0.708138	2.149101	1.605384	1.531159
2.304691	9.488844	10.41381	9.160621
1.681608	1.288884	0.976789	0.630152
1.227454	3.173481	3.206756	3.315324
3.811327	12.98616	12.44968	12.25049
2.392803	9.009709	9.302988	7.953722
1.280308	3.228144	2.318081	2.914653
3.619314	2.274242	1.630315	1.722526
0.199024	0.872922	1.044976	1.457014
4.05638	1.875627	1.167538	1.504314
4.770455	3.456613	3.009256	4.074147
2.530807	14.76172	13.66558	14.51407
21.00537	9.884576	10.04305	9.988511
7.616551	4.975914	4.821835	5.333847
0.612667	2.030931	2.622888	2.547481
2.646955	6.802884	4.215297	6.536694
0.467172	2.067175	2.777145	2.467722
0.382222	1.274429	0.781117	1.835491
1.703697	3.445013	2.451446	3.2912
0.828924	3.241995	3.190653	3.240858
3.062344	0.225366	0.035981	0.396675
8.919468	4.492126	4.469589	4.193652
0.554067	0.262536	0.275954	0.173521
9.944952	4.027722	5.176174	5.860678

17.96972	2.985725	2.913341	3.223669
14.40131	17.84512	19.2082	18.49894
14.23302	3.105099	2.215316	3.238703
49.46881	11.539	9.997207	9.232896
0.937192	3.603996	2.852371	1.821559
8.784871	5.920711	5.644121	6.241725
0.321353	1.304639	0.835165	1.678507
0.484081	5.24674	5.780078	5.736462
1.69746	1.203536	1.305391	1.357678
10.54424	3.419653	3.841205	3.273471
0.861533	2.492403	3.034361	2.889197
57.53336	28.06201	26.41331	28.2434
5.985734	16.00732	15.8032	17.62587
0.588477	4.295856	5.152966	5.731646
15.97654	8.487599	6.964947	6.985915
0.051887	0.275212	0.165586	0.060217
4.255413	6.093945	5.879056	6.674645
59.4637	155.3646	154.9776	154.7236
1.362608	4.576968	4.613513	6.10199
20.76571	44.83882	43.21991	48.49212
0.306949	1.078518	1.426985	1.105344
1.7604	6.738414	8.458189	8.13314
0.464637	9.171826	9.177889	9.497248
21.46604	25.80178	26.90105	27.25944
17.83491	29.78176	26.98276	34.89453
1.038418	1.864439	2.221186	1.985951
24.74965	2.867793	2.0105	1.81734
4.637288	9.782264	10.00117	9.852429
9.967009	5.119268	4.013474	4.501018
26.48503	56.14785	48.67874	61.37814
5.45362	1.558995	1.882601	2.202186
0.425095	2.088595	1.840079	1.293304
147.5265	34.18228	34.26315	38.79298
3.532888	7.40908	7.1342	6.375983
0.869117	0.200549	0.067975	0.089843
6.089661	8.185446	8.157726	7.519311
3.442101	5.78407	5.53579	6.292335
84.68829	33.21307	32.21144	33.82509
2.31139	6.393002	6.750365	6.306475
1.465023	5.229946	5.674379	7.144081
0.167575	0.990247	0.776258	0.779869
4.845608	20.97567	20.37911	22.2733
2.414126	10.41237	8.945703	10.72376
11.06807	9.842564	8.710093	11.1682
3.896209	0.82771	0.154821	0.854818
40.78411	16.79658	16.56823	17.05994
19.2804	33.70013	36.55955	36.94142
2.443022	32.02373	29.53688	30.76093
2.140801	4.449686	4.501641	4.646369
4.781357	5.253335	4.333576	4.753493
152.9654	79.62212	72.26852	77.10147

5.488275	2.944066	2.317659	2.392524
0.417625	1.064186	1.055127	1.355095
3.378865	0.369366	0.538766	0.258506
2.154126	1.892377	1.76691	1.543643
84.75716	21.41212	16.44986	18.24391
38.70134	22.28266	23.36302	26.17488
88.91173	189.3672	181.0652	193.5143
28.12453	42.46007	39.96248	41.71795
4.996007	4.864879	4.191255	4.128499
753.688	170.4986	185.9009	188.6148
0.615944	1.508536	0.865233	1.146417
4.27663	7.797681	8.418009	8.015805
229.1705	97.06457	95.84879	100.7776
3.407301	6.998532	7.605941	7.033021
7.068062	2.884805	1.869007	3.466839
15.79368	4.245513	4.828413	5.495277
4.612594	2.43032	1.727792	2.157969
326.7283	115.6333	121.5122	120.4074
0.388355	1.567608	1.84493	1.43273
5.254564	11.68636	12.59217	12.90955
12.47792	5.196348	4.878062	5.261605
2.657549	6.085032	5.815516	6.856198
13.29351	53.59965	48.618	52.33326
5.102929	50.2893	50.7712	51.55497
4.612168	1.106975	1.3147	1.269092
3.804741	7.457857	6.89265	6.593319
19.32917	7.553935	9.066195	6.906138
0.684296	0.239187	0.165002	0.334201
1.797403	0.924486	1.088425	1.283014
72.79522	32.20661	31.00027	34.9935
31.47314	12.48452	12.24342	14.39449
5.571004	3.36924	4.152348	2.63459
201.3627	103.9133	101.0402	102.3028
0.948092	2.254392	2.259197	2.600941
0.129787	2.171742	1.955645	2.778563
58.61968	29.63313	25.65694	29.13954
5.329629	5.318078	5.756801	6.452213
8.793765	3.320338	3.205551	3.064219
3.16811	9.396339	8.161904	8.725387
69.23744	16.51498	15.70307	17.04119
1.58053	3.140591	3.104824	3.790551
1.556496	0.633	0.514816	0.626135
0.911632	1.947854	1.41533	1.553054
3.582085	17.08951	19.24265	18.26803
2.173212	3.365476	3.592244	3.317387
12.78432	4.516099	4.727715	5.143527
2.682987	10.55408	12.20038	12.88093
0.464136	3.068433	3.019468	2.97153
40.05571	17.74762	17.20824	18.12298
20.63747	35.76101	35.71462	37.65131
51.99145	120.207	110.9931	116.1864

2.499797	0	0	0
184.9719	105.7536	104.1094	108.0772
1.103344	0.40649	0.074682	0.103649
53.47305	43.31648	42.29094	45.84513
1.464192	1.141529	0.806851	0.448393
56.50584	20.27083	20.94854	20.04207
0.25244	1.716298	2.370423	2.092304
4.269171	9.819082	7.230219	10.51271
0.842556	2.751739	2.75232	3.043242
3.366467	0.281376	0.649355	0.342939
11.47629	4.479836	4.791554	4.598333
13.20791	5.003831	4.771024	4.063441
0.870734	8.313408	6.074325	7.309136
212.5397	70.31677	72.25953	68.61888
9.944308	34.59235	32.29696	37.79991
0.411679	0.66522	0.888164	0.821358
21.01866	83.84485	85.86834	86.14482
14.79219	6.482555	4.67121	7.565681
3.245081	6.957688	6.061451	7.288152
2.147783	7.883041	7.506048	6.355174
684.3124	291.9735	289.9648	296.6735
1.3895	6.933112	6.334895	6.938052
3.340445	1.647722	1.917007	1.370035
0.807546	2.108921	2.11993	1.788949
0.219292	0.409755	0.412305	0.461975
4.967361	12.83982	13.4304	12.04565
8.415899	21.03715	17.99517	16.63058
0.231283	1.515446	0.907937	1.220894
1.913101	2.641787	2.697977	2.25356
14.82477	99.4761	99.95353	95.51472
0.493532	1.750069	2.125122	1.176196
1.958971	4.266464	3.892271	3.924382
4.909387	2.316372	3.017927	3.169601
25.67817	8.517497	8.362158	9.284179
87.90256	227.4302	225.279	230.9297
26.10388	299.5683	308.4613	318.2223
119.8114	58.5741	59.55688	63.84564
5.943805	3.124423	1.958811	2.816251
32.12666	9.281452	6.709191	7.426781
13.06097	2.179181	2.378604	1.43568
8.70456	3.949395	3.314074	2.85773
9.454124	6.295402	5.314369	5.418861
49.18517	15.1736	17.25332	16.4469
27.59444	64.89096	64.25351	65.92682
116.3846	50.20146	50.24947	51.629
3.644889	1.476732	1.579423	1.089674
0.499273	0.271867	0.235203	0.226706
4.384741	2.221528	1.780389	1.866763
225.3833	60.38762	54.18155	47.4804
48.35941	24.39339	23.25207	23.54634
341.3002	74.43957	70.69072	74.68497

81.34705	25.17135	23.39333	25.92402
1.912975	7.2208	7.703249	5.968449
0.331872	1.467944	1.299782	1.391636
112.82	15.04668	13.07742	11.74509
1.644854	0.577688	0.457793	0.726174
0.056712	0.797655	0.839471	0.886673
7.927667	2.494975	2.922782	3.04332
38.35247	19.39759	20.04485	19.59925
1.064367	2.076961	1.41745	1.438383
2.048638	4.585174	4.233764	4.441049
7.521403	31.10212	28.14869	29.07275
1.179038	1.601037	0.658407	0.831159
121.9686	55.29127	54.40336	58.34712
0.220633	0.750615	0.641568	0.678969
2.201238	4.498227	3.748374	4.481937
4.736721	3.574455	4.03632	4.295825
3.190423	14.70089	14.06472	16.34418
8.002615	7.664999	8.589667	9.210726
10.85818	3.617159	3.362697	3.223396
2.131413	1.275456	1.246049	1.039849
4.903834	6.99967	5.932739	6.131883
31.68885	60.84431	58.9484	65.35639
5.721495	7.655785	6.165214	8.009972
321.9311	1048.148	1010.478	1087.037
1.538975	13.38568	13.93698	13.44289
6.045664	0.919937	0.613716	0.978974
0.512743	1.1183	1.724358	1.287461
18.61956	36.48911	29.81903	36.86017
3.010308	9.595576	9.753558	9.819018
23.42516	10.24784	9.896236	10.04796
38.68759	19.31727	20.60033	18.59078
37.97746	13.19212	12.64829	13.73679
18.09838	4.931638	5.769799	6.769827
12.19505	29.57498	30.18478	29.85148
0.472681	1.012185	0.873439	0.580896
1.908524	2.115586	1.516612	1.362882
12.5474	4.801329	4.719092	4.543609
0.30975	3.977379	2.936812	4.039874
14.4268	34.62914	37.40799	37.32581
263.7744	103.1097	98.77956	103.9785
1.963193	6.341329	6.01563	4.861086
0.095122	2.970612	3.577869	3.6318
1.829083	0.154733	0.162166	0.160403
1158.213	394.7078	402.9862	422.2109
8.422481	11.47901	10.69467	13.90156
4.643733	9.183429	9.075886	9.729554
57.59387	17.35588	15.10963	17.8995
24.20321	16.62675	17.31156	17.29315
0.363929	1.902252	2.739661	2.194784
1.919551	3.582356	3.139506	2.582607
14.3227	45.83709	44.53016	47.51261

1.23677	2.040212	2.173857	2.106243
5.601902	3.208354	2.487715	2.570817
28.91235	99.38799	94.83015	104.3169
31.21483	59.23588	62.90564	61.58418
6.556512	4.641089	5.148353	5.363288
0.603299	1.616423	1.3246	1.051817
1.074739	1.2657	0.943684	0.467953
1.594663	3.365067	2.595052	3.221784
44.38182	9.232094	9.424466	9.664784
24.5804	59.52645	56.24896	65.29786
494.7368	195.1929	185.764	199.5094
4.038336	10.76083	10.08806	11.08354
2.376107	4.233584	4.185406	4.170912
1.268697	0.207538	0.150328	0.028426
1.330002	2.414678	2.193355	2.734296
1.365485	1.572533	1.594332	1.400084
15.92328	6.446652	7.513491	6.058055
41.9328	6.936758	7.386179	7.74764
2.015078	0.628994	0.856854	0.811148
0.411695	2.924741	1.721919	0.465525
9.278185	3.986423	3.204543	4.303741
18.40197	44.20436	41.03739	43.34086
9.697095	27.21221	25.12359	28.17585
8.690893	14.2819	10.31383	10.06496
22.33806	46.06785	48.70231	48.53941
10.16997	42.23832	41.25362	41.26067
24.13709	5.341609	5.653853	4.221667
0.418524	2.091551	2.764338	2.779938
6.298289	2.53139	3.828351	2.879868
2.852154	35.39388	30.5794	36.4469
10.51021	20.82477	20.23952	20.58012
66.13135	15.53926	15.02417	16.68955
4.422626	1.341652	0.990573	1.597543
16.99	25.54152	25.30188	25.18668
89.21357	44.36613	43.42746	46.62431
15.74677	2.222225	1.637882	1.529014
15.60922	58.85666	56.75435	59.68251
86.73235	32.61818	34.48712	35.96373
2.912572	1.704251	1.253026	2.143266
1.906349	1.109354	0.539115	0.707747
0.09673	1.293476	1.28539	1.051228
27.74632	54.63951	53.73938	59.26189
1.720937	17.19089	17.05034	16.60411
16.05528	1.65215	0.762832	1.519995
1.823719	1.943943	1.751391	1.641203
2.758143	1.117445	1.001607	0.969183
0.405166	1.756343	1.786882	1.02554
3.258522	2.034728	2.099385	2.125992
1.182005	4.69043	3.656654	4.234378
6.83823	12.51641	10.78302	11.52089
95.58005	7.145854	6.711005	7.188911

16.95246	58.32847	62.36072	62.81306
1.382497	0.328445	0.241636	0.282345
6.382485	14.03003	12.5531	15.59702
3.75813	1.948577	2.088553	0.916491
28.90126	7.900663	7.043558	7.981444
3.637066	1.40556	1.805187	1.509012
6.391018	1.645625	1.316318	1.432654
108.1236	74.228	77.19277	81.10324
55.51933	26.44941	25.83972	26.94558
3.098079	1.274933	1.411331	1.711812
0.900791	1.765122	1.940361	2.337805
1.805655	4.649761	4.288648	3.963944
1.297764	0.568314	0.866386	0.741435
9.675615	22.15632	21.01656	21.35193
29.30138	22.21066	21.64492	19.94297
0.648923	0.693191	0.667283	0.312271
0.350062	0.023929	0.130618	0.134981
6.466317	2.147517	2.423906	2.388588
4.538451	60.9308	56.82114	59.35289
4.944695	6.258923	6.182799	6.104066
1.460666	0.641675	0.3485	0.493128
0.471596	1.133676	1.4634	0.86794
21.36209	59.72499	57.9253	56.55055
15.80883	37.60319	40.4552	38.28546
14.21288	5.138221	5.73576	5.288278
11.95199	28.40224	29.84353	29.5128
0.26604	0.51661	0.86745	0.796914
23.437	8.942977	9.793006	10.65603
0.475028	2.467353	2.775219	2.767147
8.130912	2.61281	3.328688	3.384458
1.956634	7.362849	7.658038	6.57406
8.849677	5.226663	5.57957	5.233008
6.513768	2.5492	2.510711	2.966142
2.885539	7.948091	6.974935	7.26812
2.425419	1.864461	1.425795	1.406571
17.17736	8.745952	8.683775	8.211774
201.0774	384.1608	384.0693	394.3561
1.066188	0.225561	0.144764	0.193844
62.06754	42.57667	43.22532	45.61844
6.271221	10.24281	8.778695	9.484653
3.156612	0.626104	1.23017	1.425842
73.99373	33.7769	30.60207	39.20026
10.43193	8.421599	9.370635	9.875538
24.73822	9.499682	9.245467	9.47002
18.21029	10.34067	8.378501	10.04812
10.17422	3.08263	2.830209	2.459799
6.393818	3.851159	3.38266	3.759327
0.570189	4.640658	4.529261	4.460529
8.528708	19.49765	21.46839	19.43085
32.21191	12.40911	11.93365	12.96192
334.966	133.7482	131.0662	142.5204

509.4875	45.66131	46.35453	49.19455
0	9.319606	11.01894	11.38578
352.2936	156.9604	150.8821	151.5462
13.2495	5.348362	4.673523	5.395054
147.6766	26.07724	25.68156	26.05774
7.223191	14.10701	13.141	14.88627
19.55934	7.452436	6.854317	6.542027
0	1.962646	1.748892	1.65359
14.47693	177.8574	170.3524	180.9466
19.81066	29.23451	33.07415	30.91719
0.534254	0.953456	0.889804	0.683944
0.695281	1.925859	2.138902	2.585676
2.533528	1.244346	0.791694	1.18539
4.849298	3.706494	3.892432	3.40639
0.585281	2.942017	2.64906	3.241282
4.82952	4.073266	3.702511	3.816983
76.49947	1.701578	1.204714	1.425537
0.773402	2.688452	2.275676	2.326632
27.66059	12.97463	12.714	14.07015
63.7312	16.88072	18.73055	18.65067
1.242281	2.322147	2.375201	2.137442
307.3241	117.4433	116.924	122.6257
1.093757	0.89953	0.663525	0.669954
22.1858	6.383922	6.642047	6.170333
18.08654	5.772067	6.446456	5.436986
6.049775	2.69219	2.12781	2.153556
3.913162	2.712243	1.92198	2.442887
128.2683	57.44605	56.16079	62.24415
0.387207	0.309913	0.818245	0.834028
1.774372	1.152019	1.981452	1.760292
13.24084	54.30729	52.51135	55.15724
110.9012	24.70266	25.6122	23.28654
0.569567	0.927181	1.06256	1.551827
12.47415	24.88461	24.71611	26.46489
24.65403	18.03501	14.14252	13.15933
1.701421	3.869354	3.811796	4.561035
6.475008	27.29462	28.83056	28.7731
18.23755	6.851565	5.292369	6.997056
9.400594	3.46457	3.573127	3.895906
17.61056	5.927445	6.280954	6.873049
1.745747	15.44674	15.36832	15.04263
19.31061	23.08783	18.42091	24.8431
0.575804	4.509761	5.736449	5.242294
0.832901	5.141361	2.933041	5.087091
403.4267	190.8712	184.0823	189.7325
19.3007	8.135331	8.320552	8.120187
5.646252	13.91641	12.76148	16.37611
2.389101	1.356097	1.258308	1.027351
0.310395	1.65108	1.62541	1.818089
0.050445	0.831441	1.019856	1.080113
4.030206	24.961	23.27418	25.65452

3.400378	2.304383	1.810782	1.555616
7.822167	2.748551	3.128825	3.651342
0.304752	0.708053	0.633613	0.474981
71.04226	35.22972	36.82567	38.67127
0.114656	0	0.217484	0.377143
9.154376	33.19743	36.20444	36.48225
40.84038	12.45335	11.22731	11.11053
7.712676	5.789379	4.677543	5.112192
4.61852	13.51614	12.37968	12.1245
45.4339	18.46999	20.35589	21.01515
0.328914	5.654256	5.405748	6.009902
8.126526	23.54196	24.27333	23.87354
19.02024	55.13121	55.1107	54.25983
3.152437	3.279387	4.182528	3.248535
0.265056	1.14334	0.785134	1.11338
2.607294	9.25237	8.034269	9.308346
11.94749	158.9113	139.9389	148.3258
4.163548	12.25187	10.13771	11.57238
3.119345	7.507378	7.722825	7.447782
0.345368	12.00171	10.09211	10.80631
206.91	90.52514	93.02225	86.87375
211.3811	53.91579	55.12716	61.22165
2.887018	4.4081	5.180524	4.952575
426.4381	118.2699	115.6826	118.0799
7.955276	4.045336	3.571893	3.853395
2.711101	4.21099	4.551707	4.503732
1.144562	5.676932	4.743203	4.839046
1.887416	6.765439	6.449091	8.234945
18.81939	8.237399	8.790401	8.823393
1.235531	0.937225	1.015699	0.559951
3.965051	25.25249	25.54995	27.73254
12.19331	7.184525	8.51682	5.872799
9.910822	11.68813	10.3293	11.645
0.311132	1.404189	1.798231	1.506469
3.092928	13.31481	13.14064	13.03249
4.805848	4.163529	3.211543	4.14929
2.997009	1.072344	1.399122	0.718857
28.84763	3.557868	3.324115	3.466915
10.78254	22.94608	23.02679	20.71672
21.83715	9.389133	8.428531	8.143716
2.814967	1.349479	0.623523	1.233541
43.28851	32.06766	34.06413	34.52317
0.328214	1.202779	0.872511	1.586127
15.03413	6.556505	5.213233	6.1559
2.64547	1.403338	1.626981	1.638018
5.087352	2.237346	2.086783	2.206393
1.447394	8.316898	8.037974	8.376598
0.27805	0.747814	0.563548	0.677752
26.47582	10.34076	9.834036	11.98363
47.6285	25.63223	25.49307	26.90411
1.122386	1.965135	3.090808	3.369368

32.06121	1.578493	2.204312	1.284165
163.0716	430.7809	425.182	454.5972
15.34485	3.786067	4.730051	4.075879
40.15467	86.86855	81.03986	83.64668
0.227885	0.037646	0.052261	0.132144
2.497473	21.54606	22.10215	23.22261
4.726592	1.750628	3.1271	1.698585
2.111583	13.02594	11.62779	12.68716
0.394717	1.143679	1.197537	1.640449
4.434527	4.986941	4.684418	4.746604
73.02769	26.78365	27.48004	29.94667
5.967701	13.53129	14.0873	14.52474
1.263661	7.202063	7.212849	5.850447
6.407697	15.22746	14.27218	14.21685
18.84068	7.529672	6.824781	8.361923
5.200059	10.7107	10.49328	10.03434
15.17032	303.9427	315.2001	316.9718
1.341323	3.168007	2.95078	2.754802
3.075042	9.390282	6.461958	7.647593
4.023096	11.35705	11.66843	12.47347
0.226541	1.511001	1.444332	1.893084
17.42691	9.094116	7.920999	8.483747
4.641604	14.31246	16.19665	11.02578
10.66718	1.304906	0	0.59574
1068.434	298.8368	332.7068	319.6102
6.53722	8.90331	8.868863	8.914503
1.085406	0.601688	0.754521	0.631599
1.63736	4.869667	4.705107	4.904194
16.10099	49.5838	48.93479	48.08847
12.38472	32.2867	32.71528	34.34185
17.40659	4.744675	3.619789	3.90944
9.863923	4.493614	4.810159	4.929952
0.585852	1.610882	1.806968	1.483968
524.4494	263.2833	275.5085	287.4968
41.41515	20.5508	22.29743	21.18419
3.098618	1.855053	1.208683	1.312146
0.409257	1.188189	0.930486	1.169927
1.26637	2.798029	3.161353	2.849417
4.790048	4.04132	3.016256	2.736703
257.0035	14.02474	15.16753	14.21404
0.936818	1.954476	2.165651	1.82581
4.921034	2.569721	2.998293	2.825156
18.32998	44.78717	45.19636	46.43252
6.155556	17.03818	17.6424	19.13545
3.91898	13.51959	11.30966	14.1968
0	2.143809	3.002339	2.716666
8.10353	40.41834	35.38977	39.03205
20.97895	7.702855	7.936481	7.149021
7.130691	2.945876	2.565622	3.249171
15.15471	4.319649	5.193746	4.368393
29.57348	11.12826	11.3309	12.85572

6.006983	18.90143	20.30278	20.34155
0.151141	0.607119	0.906186	0.527172
8.376877	23.87563	23.14512	24.42842
6.08628	23.61399	23.58002	23.74689
2.010168	8.312028	8.211546	8.894263
0	1.025585	0.686843	1.057461
13.87864	3.052402	2.062829	2.980032
8.560661	1.764358	1.029237	1.496269
12.0691	33.70327	33.42538	33.92128
1.158056	0.739667	0.368181	0.783828
0.232437	3.051295	3.124845	1.162825
43.98199	132.4297	148.8883	151.5606
0.952028	2.302503	1.376196	1.823141
0.59869	1.159182	1.633348	0.664325
18.09568	10.53043	8.625387	10.21286
1.457533	3.06876	3.205071	3.02674
10.88991	2.771047	3.589781	2.711009
0.220508	0.036224	0.031421	0.025494
1.873567	3.122672	3.753247	4.799015
26.97257	9.769537	10.68021	10.97054
12.56756	0.608813	0.108136	0.146806
2.42999	1.364891	1.154966	1.443923
2.887213	3.147657	3.39357	3.269286
3.653667	1.42726	1.481392	1.571602
6.522809	23.78157	24.84494	23.62318
4.109146	12.1622	9.907739	11.33712
46.77266	52.149	50.40649	57.64201
57.7142	26.95044	26.63557	26.10256
0.427048	3.906112	4.033382	3.710283
11.71505	22.12145	21.17512	21.17484
94.42509	43.08774	42.95468	43.31597
16.9679	8.409664	6.124565	6.132257
7.95428	3.349133	2.902079	2.616078
21.85611	34.55634	34.84096	36.08124
2.726321	6.410616	5.858121	6.443262
2.429565	6.005867	5.601602	6.995622
11.47146	1.478318	2.08546	1.815765
329.5123	179.561	165.2559	176.6722
0.590523	2.272047	1.692624	2.582634
45.14722	21.08689	20.78576	22.18555
13.17724	75.9071	72.53359	70.78735
10.1452	38.37571	36.42827	39.4104
1.148485	0.360378	0.676203	0.671777
0.177653	5.492089	5.603359	5.302969
282.4898	89.42355	82.52675	90.0446
1.749822	3.020819	3.516946	4.129472
0.161304	0.581539	0.795931	0.886505
3.629168	3.687312	3.543219	4.52232
0.797142	0.013394	0	0
15.21361	3.452323	4.649018	4.365503
33.21737	7.678428	8.90349	9.113767

11.90325	8.663112	7.656416	8.637539
13.68897	32.27779	32.22038	33.55357
29.5294	9.758124	10.28807	10.75438
1.714379	0.71737	1.302151	0.664726
2.030151	6.273621	6.775393	7.086461
1.051142	0.147254	0	0.063637
2.507612	1.818572	1.676577	2.091259
10.76574	5.369492	4.848818	5.768399
65.18388	155.7366	152.0196	159.5207
3.127644	7.473997	7.694519	8.148767
8.71489	11.18833	10.66428	10.57391
2.289336	1.137479	1.466221	1.929953
7.080798	3.069582	4.197968	3.309752
1.367626	0.459573	0.547011	0.424313
2.957026	1.551853	1.530812	1.622063
6.281298	7.202224	6.365016	7.726864
19.37626	6.510294	7.262231	7.346113
3.355799	1.999598	2.026097	1.29534
181.9163	75.90051	76.06581	78.24752
3.325902	0.931993	0.6631	1.274273
1.883891	18.5252	18.50312	17.2167
10.83123	4.632217	5.302975	5.206623
7.572044	13.7563	14.90108	16.15397
0.676418	0.138083	0.182779	0.156011
13.5469	34.82712	31.28535	34.63318
0.31847	0.458114	0.476417	0.523245
48.03934	11.5095	12.62843	11.50937
5.107501	4.526077	4.225408	4.476308
0.61232	0.675884	1.301513	1.176367
1.460854	0.292636	0.271982	0.323023
3.835059	12.22044	11.3897	11.13298
35.58958	47.70329	44.64105	50.93531
2.45212	8.534163	7.230534	7.842941
307.8375	734.3957	722.8204	747.7983
4.521625	1.398501	0.919339	1.013776
0.90878	1.259863	0.868515	1.699672
4.417179	3.742618	3.526548	3.434711
0.276469	0.618946	0.475336	0.59853
3.356573	7.598326	6.876085	7.391901
1.646104	0.455152	0.482726	0.741258
0.633684	0	0.03542	0
5.165673	2.57139	2.275678	2.785319
30.01779	73.70988	71.57988	76.48507
51.48142	19.92052	20.46264	19.97298
44.70001	18.36268	19.30574	19.62483
78.93202	33.67284	32.67339	33.53027
0.438351	15.82917	13.46453	15.71317
3.386522	0.191773	0.411757	0.299101
6.054305	24.29705	27.32555	27.01304
1.40246	2.399801	2.704961	2.614448
0.892123	11.20984	10.23954	10.95273

7.978552	3.533403	3.628253	3.724694
8.63249	27.08366	25.62465	26.23273
579.6731	297.8929	287.5959	303.8262
14.50763	1.423554	1.605818	1.455242
1.59822	1.078831	1.240382	1.252726
13.65441	3.010578	2.87004	2.759321
3.569495	6.021496	5.607316	5.73079
1.86669	2.330239	2.23972	2.298111
0	1.58105	1.428514	1.947274
8.561257	21.53895	19.56803	20.90958
1.197498	0.570072	0.60869	0.671825
3.35406	15.5852	12.36358	13.93375
28.97424	84.37716	83.82121	85.45194
3.721404	7.794491	8.852393	8.193046
44.23628	3.136469	2.20829	1.736364
4.766898	10.60331	9.061683	12.24634
35.24871	12.00278	11.50074	15.63246
39.21977	71.98399	70.61308	77.04787
10.88362	5.846682	6.293787	5.768567
18.79861	42.51738	45.18229	45.26234
11.26181	0.210346	0	0
19.34047	8.809115	9.040733	10.62318
5.33361	2.024102	2.37158	2.223279
13.44483	4.717542	4.38946	5.29445
246.5501	113.8593	117.3378	115.7309
2.979003	3.706001	4.350432	3.860449
6.353142	11.16619	9.290977	11.93823
3.283283	1.235235	0.894258	1.312849
51.26328	1.621831	1.567557	0.823518
1.53713	4.067987	4.901145	4.110688
1.376025	2.56034	1.982546	2.27544
1.237201	0.776256	0.771939	0.720302
3.956562	4.18773	3.482252	2.951153
125.5893	75.13828	63.63039	69.34768
2.092641	0.640919	1.225909	0.793062
11.48803	23.77338	27.01866	25.48241
30.38614	82.23117	80.36817	84.84032
0.294987	2.733064	3.456858	4.243821
134.8773	368.3246	348.4386	382.438
17.3047	53.9808	56.06609	57.50135
1.029495	0.996845	0.908202	1.466557
9.621986	27.03177	25.78557	25.24869
0.82808	0.485293	1.466818	0.966346
0.106227	2.830166	2.851985	3.092981
0.353778	0.945746	0.725732	0.876167
2.034063	0.905509	0.632399	0.944981
0.088504	0.660567	0.897192	1.002186
1.498467	3.283437	2.892151	2.733925
2.282657	1.017396	1.507002	1.624183
0.322119	12.40997	11.97769	12.79788
7.17905	23.55598	22.3994	22.93418

3.002113	1.763564	2.382899	2.025561
325.3011	79.08449	83.12908	93.39987
0.726163	2.028605	2.599357	1.814448
36.90241	19.01679	20.60918	18.57173
116.6756	37.09065	33.92905	39.87297
7.529438	5.788105	5.910861	4.875108
0	0.279405	0.524897	0.311444
1.762844	8.197098	8.789066	9.13477
0.183794	0.720046	0.637084	0.717861
0.206485	0.284071	0.42184	0.405846
3.48973	29.60387	30.53004	28.01402
6.055633	7.102172	5.541075	5.989693
149.8893	105.7756	102.6723	113.454
171.447	384.4271	371.2318	392.5428
2.612589	4.278254	4.195871	4.278051
1.05106	2.764002	3.172624	2.366265
21.89875	49.39395	47.09427	58.44539
3.487196	35.31022	31.58196	37.11286
1.500735	2.847808	3.368701	3.307963
1543.606	452.4421	441.5118	468.9151
7.771221	2.746576	3.808521	4.108461
0.523885	6.234691	6.137276	5.143187
1.12685	3.162968	2.812957	3.29133
1.502051	0.533992	0.443492	0.744631
2.025071	4.724184	4.474272	4.96041
0.431396	0.783431	0.73414	0.479645
0.900424	1.12637	1.795356	1.440542
4.546642	11.95663	11.06765	12.25114
11.39899	21.05704	15.00886	18.20588
2.943144	1.455248	1.851217	1.328866
20.58248	5.647669	7.304044	6.656561
0.650534	1.334191	0.697331	1.124078
9.788404	33.35273	40.26818	31.03973
40.38606	197.9452	208.3799	211.1635
9.247882	35.3254	35.17247	37.38054
33.06	6.626568	6.842141	6.61437
1.214371	0.672502	0.424336	0.435976
1.087233	2.261841	2.767442	1.867298
59.25699	22.99468	22.01226	23.73659
16.19186	4.230421	3.739627	3.88572
5.03558	13.85626	12.71684	13.12431
1.838591	4.122446	5.120653	6.059005
7.107279	30.39707	26.66913	32.83253
0.647701	2.737492	3.403736	2.757213
3.503277	2.107223	1.519698	2.320115
12.52128	13.54054	10.87498	11.89506
4.26823	1.921932	2.215163	2.387692
7.420881	29.01408	22.79909	25.58608
77.67169	188.6885	181.1445	194.2495
2.362173	6.896014	5.543681	6.510707
7.38944	1.63664	1.742921	1.59344

22.32221	6.439133	6.332845	6.096808
17.93357	15.66482	14.707	15.53511
10.42032	48.05084	49.11336	49.94993
90.70016	9.220693	8.414872	8.622226
0.453526	1.185786	0.77702	0.449259
0.999297	0	0.104429	0
6.297576	1.374505	1.457505	1.056527
26.44272	43.84583	41.59958	49.41192
103.2566	36.26898	35.82042	35.51226
50.37486	5.674792	5.75863	6.23721
46.99002	10.43317	10.17437	10.10598
15.01847	34.83865	32.94576	33.25032
4.466709	2.446419	2.435983	2.287033
2.391554	7.447961	6.165512	7.17954
18.51954	8.854973	8.540111	8.383316
1.594381	2.543003	2.641201	1.773758
23.49096	22.41653	19.56792	23.26481
1.95522	15.50818	12.62044	14.5582
113.6304	29.62572	29.11331	32.57341
2.413517	1.046499	1.000609	1.266362
5.119977	20.30762	20.80721	23.35873
0.542888	2.308243	2.267293	3.243417
36.40598	20.09888	19.70482	21.74172
13.91797	3.782023	3.449336	4.616189
0.269021	5.319975	5.078673	5.406482
15.19369	16.3604	16.86899	19.06557
0.034061	0.572258	0.556511	0.281225
26.90978	46.12382	45.50829	48.51571
26.89254	111.1447	109.921	110.5505
0	2.371902	2.68534	3.078037
4.005276	7.862474	6.293838	7.153709
1.516465	0.56665	0.649207	0.554199
17.24881	18.99503	20.9338	17.74254
2.273167	1.172009	0.568885	1.394806
9.861819	3.224216	3.788067	4.36295
104.2227	27.69659	25.0037	28.56216
5.149916	14.24569	13.86345	15.9032
3.357553	10.13949	9.52764	10.91203
7.115659	2.170206	2.147621	3.029771
0.379777	1.73305	1.0508	1.469734
0.392281	0.770742	1.06048	0.949373
0.589329	1.324724	0.938932	1.387541
0.920405	2.834381	1.977686	1.729021
10.64372	5.034277	5.949839	4.435668
0.115187	0.328922	0.514321	0.363172
106.4523	45.03246	46.13585	49.72063
3.216958	23.25679	21.00607	25.03589
0.034647	0.062848	0.159273	0.32303
4.156197	8.937391	8.365932	9.069108
0	3.423635	3.184254	2.84626
0.695249	2.772542	2.055893	2.129681

0	1.376547	1.040109	1.310602
4.298357	10.36441	11.94454	11.57421
17.41467	3.962723	3.564326	3.97104
0.132985	2.429306	2.888287	2.115257
72.8963	26.51103	27.09954	29.57448
4.85701	15.34472	14.83518	13.8591
10.35244	5.419493	4.993245	5.844268
3.383272	1.262655	1.407524	2.062327
4.52253	1.616936	1.777221	2.524708
2.870727	10.01127	9.991632	9.73151
6.343106	4.82831	4.562386	5.30282
5.605394	18.52242	18.47329	18.40942
50.18637	7.910477	7.163184	8.129592
3.181332	5.548788	6.026774	6.061676
13.37521	5.34172	5.252274	4.652582
8.529448	0.851995	1.48418	1.153641
25.51942	77.66484	77.32119	81.26376
674.8208	85.30373	81.44946	86.24916
67.61018	15.18716	17.8428	17.08721
0.593225	0.500836	0.395666	0.308529
1.770749	5.037815	5.152478	4.74537
4.03462	7.370253	7.997854	8.22297
0.095186	117.6624	116.129	118.2571
27.83836	11.7415	11.86585	11.79654
4.655561	35.35623	31.12551	33.98923
1.91047	5.677522	6.239786	5.648723
1.713333	1.470376	0.589442	0.953851
0.428393	1.915017	1.987442	2.016932
1.282853	0.508361	0.655732	0.735712
7.28028	17.30581	15.32273	15.70291
0.509319	3.565278	2.65227	2.671099
208.9716	68.7881	71.43036	74.01779
1.465799	2.588543	2.409274	3.197173
29.05337	15.31464	14.35438	16.66818
26.65941	10.80087	13.01227	12.24161
247.7823	457.2376	423.1474	441.316
94.02041	57.22144	57.16557	61.09777
0.331258	1.270498	1.414779	1.219055
22.35568	10.63735	8.353164	9.623778
9.87655	4.307166	3.628151	3.538725
154.3541	607.23	598.6576	624.2145
28.5587	6.455221	5.399278	5.72192
4.345916	1.315068	1.637785	2.384391
169.7066	84.97629	85.37347	94.10728
1.914597	7.23185	8.052397	6.394349
0.986619	2.587461	1.547678	2.721411
0.751318	16.89434	16.38473	19.91821
5.671302	29.26329	27.45267	28.34642
0.522057	3.197555	2.531245	2.198539
0	0	0	0.172742
0.104719	1.309004	0.64212	0.929055

12.20727	5.978513	6.844945	7.471205
1.492861	0.262629	0.085024	0.090206
49.64228	24.04437	23.69487	25.46153
13.2359	57.6997	61.55894	57.68971
18.88007	66.7863	67.96747	69.67921
2.252463	0.582845	1.024208	0.728932
15.79425	8.727961	4.5762	4.95817
4.967592	33.96961	33.44615	33.96361
10.38134	7.243966	7.327518	6.315188
97.81894	978.1829	950.4409	998.3265
76.84487	22.81475	20.93091	23.05527
6.832481	0.879097	1.835908	1.316282
0.704569	1.304234	1.149904	1.280491
28.05183	59.66861	58.19704	61.39541
57.6258	8.995162	9.325445	8.802539
7.877886	13.52397	13.26697	12.37881
64.67913	27.40312	25.83044	28.52551
23.47304	8.660833	9.172629	9.954764
0.915326	1.826934	1.671013	2.13082
167.8511	75.94759	69.66772	83.77257
93.20614	36.24437	38.18232	40.44619
8.282012	23.77156	21.64061	23.78591
1.256139	6.393209	4.579165	4.931731
0.355612	0.143803	0.271581	0.068798
0.828043	0.085203	0.200573	0.090897
1.331511	1.315189	0.884624	0.861204
16.73923	1.569505	2.409818	1.869125
29.86937	15.73352	18.93279	18.11873
37.01783	9.619886	9.537137	11.743
657.2322	320.9769	311.9026	320.4931
36.99238	17.93475	20.03506	19.52569
22.72633	6.61034	7.277869	7.521034
8.456752	21.28132	20.29167	21.13489
20.19622	7.005733	6.648786	8.329268
2.313637	3.225632	3.548858	3.657033
29.70133	38.73212	38.0884	38.24115
0.638189	4.255776	4.387062	4.55137
2.875458	8.923254	6.952265	7.262758
23.69681	8.557429	8.575508	10.68342
1.144504	3.88855	4.160979	3.860914
22.04034	8.357809	7.746914	9.057244
1.335112	5.034451	5.289797	6.349348
5.710385	10.67732	12.80007	11.68328
0.775527	0.721266	0.24031	1.131847
4.40277	1.850337	1.982358	1.864155
348.0733	170.8669	154.0603	171.5685
0.613551	1.215991	1.093299	1.126102
0.578397	1.522644	1.248292	1.244281
4.473653	0.129473	0.383283	0.273011
1.384002	3.147032	4.160164	4.466585
17.57252	38.4228	36.6356	40.35141

11.99748	5.848609	6.134291	7.358882
1.916762	0.532104	0.367995	0.830324
6.495885	19.25758	16.26717	18.70296
13.67143	4.393239	4.284	3.577744
3.691643	2.086531	2.013867	2.168609
2.496897	4.329721	5.88031	7.057178
7.251283	1.897758	3.394285	2.897404
3.553349	0.730345	1.791815	1.463286
8.080823	1.947413	1.51927	2.199295
38.8317	20.84889	20.55864	18.26581
121.0989	42.01348	39.17751	42.84563
1.156086	3.654573	3.474627	4.211413
21.1308	68.29284	64.58974	70.08899
3.142099	0.76247	0.951044	0.650964
2.772018	6.348139	5.890443	6.283478
71.54008	150.9676	149.63	160.5693
9.538488	45.82997	45.28949	45.93369
1.885959	1.201102	0.623281	1.270053
0.423874	5.301853	5.796047	5.015355
1.745875	0.715796	0.606131	0.893418
0.293935	1.706813	1.254954	1.50932
3.468326	2.427177	1.578286	1.94352
5.518428	18.82973	19.83459	23.59282
34.89088	35.82585	34.58608	35.51462
0.050207	1.559178	1.232008	0.889228
35.61435	12.39724	13.49195	14.6484
27.49933	8.308639	9.439728	9.98633
2.457091	0.879561	0.950158	0.993815
18.8559	13.4771	13.97485	13.99258
7.757784	2.7396	3.054565	2.876216
5.909558	2.254441	2.373517	2.49784
5.788276	1.95282	1.637236	2.113602
6.901008	3.740926	4.006235	3.089948
5.062727	0.265742	0.654666	0.969845
0.575293	1.027934	1.143228	1.032022
63.79604	31.09057	33.13115	32.44155
0.193454	0.34395	0.326066	0.404229
19.82846	44.86474	44.55886	46.076
1.781237	4.330705	3.512848	3.491099
1.541705	8.122618	6.750433	7.845208
1.984276	1.377458	1.89584	1.606085
2.650118	6.950951	6.944346	7.19282
0.052136	11.31759	9.575999	10.53227
5.715341	8.404255	7.851437	8.32107
2.01969	19.27736	15.32305	17.25412
9.201688	2.146285	1.556525	2.060972
144.0374	50.20404	49.84984	52.45572
1.84695	4.242834	4.854116	5.153685
2.192467	1.117362	1.099327	1.168198
25.68561	71.93298	68.18879	66.6786
1.248843	0.216093	0.336703	0.143147

15.68756	34.25955	34.97633	36.14898
1.936858	17.79491	17.07536	19.51551
27.29859	4.834034	3.568151	3.425107
0.323434	0.766593	1.022277	1.17686
11.1288	1.864322	1.807088	1.49191
49.79313	0.424964	0.427415	0.589549
4.376652	78.13828	69.91958	76.64995
1.865496	0.888175	1.319871	0.547164
0.604897	1.611583	1.788058	2.18298
7.812254	3.460688	1.974586	1.881618
2.484132	1.059436	1.302227	1.373431
54.12344	30.38721	29.16496	30.13678
16.56563	80.01048	80.55486	84.9469
23.6731	11.99658	11.62844	13.51269
22.46532	44.25374	41.99936	47.56245
32.39779	33.98277	33.34928	38.78491
0.854901	0.856476	1.153092	1.168141
8.993104	0.170698	0	0.112784
1.185416	2.623008	2.291751	2.517479
17.0415	27.52883	22.17084	28.92999
67.82225	23.53875	21.85747	23.25646
38.27295	0.706576	0.787272	1.08563
1.091845	2.620441	2.738704	3.356625
63.28509	21.56828	21.31926	22.7905
1.560869	1.271467	1.010054	0.796457
3.740389	6.001461	6.563195	6.345337
244.5941	864.475	865.4366	899.7587
174.8736	73.34827	76.6237	77.33501
1.32761	15.07131	13.8652	14.64722
9.524584	27.35693	28.41592	31.12093
2.359895	9.89622	9.173236	10.36439
1.778078	0.594064	0.7709	1.389638
126.1499	55.10653	51.47038	53.74658
140.0123	33.7547	29.57943	34.53739
0.877529	0.305808	0.322754	0.303132
4.100953	2.710878	0.941766	2.096214
16.37637	37.54095	34.3474	37.22996
0.057352	0	0.060804	0
6.036432	2.974862	2.437001	3.191163
13.60477	33.02474	34.69563	35.85644
2.232906	12.23366	9.994707	11.2029
0.092918	0.519743	0.553068	0.551709
0	0.098243	0	0
32.3299	3.794788	2.900103	4.845628
6.163211	12.63892	12.89347	14.30476
0.27002	1.19385	1.507614	1.349276
10.68431	13.45736	10.81674	11.577
3.094666	6.896147	6.774506	6.745478
0.760999	5.357085	4.684281	4.600691
1.788654	3.445612	3.045359	3.026907
0.323451	0.596305	0.225777	0.770048

0.6446	1.905048	1.188451	1.306808
36.36828	14.89431	14.00152	14.92318
3.71824	10.73776	10.01212	11.09802
2.095455	41.49804	43.86858	47.32835
178.705	64.57551	62.38635	65.41052
8.116193	6.412858	6.437209	5.802431
41.96198	95.09244	92.70649	97.59861
1.058779	0.033506	0.723651	0.35278
91.45948	16.66226	18.13054	18.56327
5.627598	1.547318	1.654017	1.410019
3.144976	5.902233	6.283094	7.046
29.92689	10.92449	10.67461	11.43244
266.6194	80.50586	72.05338	77.23686
8.612712	77.52734	81.32687	79.59988
4.998322	11.24792	10.5143	12.20967
1.363416	0.840201	0.649451	0.752829
10.44176	3.657462	3.449258	4.916811
5.281606	24.70913	26.67685	23.42381
0.602623	0.577225	0.15154	0.431877
0.659283	1.37328	1.604622	1.819237
2.634562	11.68301	10.69398	9.592031
2.591289	5.006251	4.49358	3.673512
14.40709	4.261497	4.075001	3.747213
2.319478	5.886158	4.288985	3.864784
3.265194	4.67769	5.528859	5.519678
1.157839	34.74657	33.33198	38.25584
4.249523	8.563939	10.70395	10.13747
6.307643	13.14853	11.92265	13.21669
1.112264	0.622066	0.302568	0.214682
341.9252	96.67493	100.1565	101.8176
13.93157	37.31541	33.92279	35.34948
28.26964	14.73697	13.78537	14.94413
0	0.019716	0.063801	0
8.392231	5.310003	5.265265	5.111712
2.330751	10.0933	8.961681	8.911663
213.3488	462.1554	445.08	466.1829
0	1.739445	2.290133	1.598471
6.483886	5.859413	6.14448	6.658331
184.0895	67.39542	69.95245	71.13522
141.5006	42.02842	39.8286	41.45969
1.676555	2.991169	3.793094	3.359277
17.84931	34.38048	35.89361	35.28446
10.24636	5.982056	5.649343	6.793808
87.53083	277.6803	287.8642	283.1209
4.622121	11.54063	10.87074	11.52951
6.460721	23.73026	22.66374	24.31492
0.418722	1.424018	1.144946	1.373882
23.07453	5.301523	6.277909	7.043033
2.080453	3.609236	3.305512	5.158066
2.747078	0.876804	0.505608	1.197649
6.30225	22.27868	19.89674	22.90538

16.55702	40.72646	44.42925	43.29306
11.3861	5.358254	5.686592	5.623058
603.6411	874.9288	891.6483	890.7057
1.224647	0.621164	1.245646	0.917515
1.304313	0.742375	1.26963	0.369738
20.74422	9.532708	7.885776	9.011481
2.664149	5.333421	5.500944	4.318552
3.809733	9.736191	10.64998	9.385533
1.540681	5.695271	4.767387	5.336854
163.9211	48.57878	45.79341	47.84573
446.5807	1522.065	1557.941	1579.808
4.555724	1.599916	2.184946	1.687443
1.252494	0.761506	0.881591	1.437554
1.344441	2.30686	2.02967	2.849755
0.094586	1.094117	0.9429	0.781146
2.670489	7.034276	8.602505	7.175504
2.03544	1.220987	1.3271	1.901611
29.21887	93.21309	99.26148	101.7198
8.736552	77.01634	78.10017	80.68325
19.37138	8.4543	8.146256	8.762234
319.7361	141.3087	133.1423	144.2414
0.46764	2.191843	2.199857	2.557572
6.493117	0.208036	0.253498	0.358434
2.091097	0.483187	0.503498	0.772362
1.986197	4.411537	6.197901	6.371791
3.667166	1.313553	2.053673	2.092759
4.048473	8.071682	8.573597	9.262786
17.21904	43.98985	42.3585	42.72863
3.560923	0.404819	0.479783	0.598192
2.465525	4.906	5.307242	5.720555
0.038439	2.310722	1.565328	1.820249
1.016653	3.216199	1.744365	3.148779
31.72845	11.34543	15.53066	12.91616
0.187697	1.424809	1.724734	1.883201
4.644854	1.985573	1.860829	0.956898
3.901965	5.107907	6.322112	7.637149
1.447327	5.572765	5.703093	5.424137
0.241547	0.110462	0.091255	0.145994
14.07994	1.028079	0.597535	1.156279
10.47429	4.094285	3.68322	3.989008
1.696379	1.095467	0.639199	1.084454
21.34938	7.836276	8.559851	8.075712
3.202737	14.18337	13.82773	13.89882
19.41328	62.76567	63.6709	62.35409
17.73682	8.701973	8.822829	9.266446
778.0128	363.5601	353.7912	367.2855
18.70906	7.661597	6.414737	6.575195
9.632833	14.36092	16.00508	18.39718
12.11935	5.666168	5.106711	5.809879
0.436353	3.470557	2.893321	3.867331
27.42178	47.56379	49.797	43.15762

1.189825	1.830905	1.233438	1.355943
0.145546	0.817467	0.625839	0.812345
2.265659	0.755952	1.463569	1.10608
11.68498	25.35503	22.68972	26.41408
21.26672	31.18824	26.40039	28.89606
13.94951	35.91087	32.47757	30.71866
8.171395	14.71114	13.55011	15.0238
0	0	0	0
0.532765	1.820851	1.533829	1.865529
19.12196	22.67051	19.76314	24.48434
2.934596	1.90022	1.580808	0.845822
210.508	74.51325	87.14299	80.85823
27.05244	51.85852	50.24046	48.17497
1.330438	6.47336	6.161107	3.544106
6.792552	3.298699	3.108408	3.257547
3.806785	6.608587	8.977714	8.95779
0.766062	0.742079	0.496287	0.422458
5.558387	1.175533	1.393984	1.199236
18.95327	34.16496	35.27545	34.13302
38.34007	5.554248	5.015687	5.163662
12.3724	8.764182	8.698568	7.975476
123.4618	51.64082	45.43616	47.08168
0.292473	0.332916	0.296735	0.307615
0.468533	0.904398	1.380511	1.147778
0.551112	1.141449	1.010827	1.219348
1.373347	0.47789	0.439361	0.165404
1.552813	10.30028	10.1756	11.30118
0.280134	0.200677	0.202222	0.123455
5.450592	14.6652	13.76593	16.21473
5.791748	1.907313	1.937109	2.346711
1.44174	9.601016	9.794322	11.27848
3.012243	9.969966	9.438548	6.983147
3.665788	2.384337	1.018987	2.177108
0.099585	0.655865	0.495132	0.511003
0.415155	4.109057	3.25107	5.269628
0.978355	5.315879	5.270523	4.858573
0.247072	1.894715	1.696672	1.734806
2.838929	9.744388	7.138459	6.361077
6.043643	3.486182	1.930161	2.690668
6.109917	3.128069	2.526666	2.669601
10.11181	27.42328	27.61399	27.43706
2.172482	0.721117	0.748335	0.345097
0.389806	11.84626	11.00497	13.21979
2.535055	5.283893	4.566782	5.666511
9.733066	21.02949	21.89559	22.17812
184.6559	78.80905	76.47264	79.69182
0.577989	4.074832	3.202954	4.183364
6.82452	15.00422	13.6009	14.97271
5.583044	0.486069	0.291207	0.316957
10.19722	22.94569	21.31684	21.21497
8.424339	4.249778	2.607542	4.019789

13.49526	6.143462	6.111877	6.925503
115.4417	63.38859	62.37306	66.62066
231.2905	49.44141	44.74751	45.54614
0.28218	1.836164	1.617385	1.355676
1.713328	1.091367	1.382343	1.620468
8.647775	2.040798	1.647912	1.444616
2.143274	3.067106	2.746467	3.755882
7.954521	3.982012	3.759163	3.374144
0.242213	0.369891	0.480781	0.401117
246.9542	112.071	112.6805	112.9279
16.13018	5.869268	6.763721	6.463983
829.7936	875.4985	815.4225	886.0421
4.409328	9.714348	8.682838	10.0148
3.588745	0.463926	0.268853	0.784885
4.574049	16.84124	16.98233	18.69523
5.658747	0.999896	0.792142	0.847043
10.58418	5.425667	4.872385	3.070271
581.8544	223.8352	230.2247	237.9308
9.028335	4.764496	3.747025	2.79179
0.791562	1.928422	2.200876	2.130675
2.306916	3.967285	4.80171	4.138408
9.467732	22.54201	21.53868	20.38964
2.89789	5.613208	5.588025	5.987005
0.636233	1.306053	0.980312	0.894108
0.453489	0.883	1.113682	1.227605
22.40179	10.5178	11.00573	11.58031
1.838372	3.331437	2.961878	3.056208
10.18938	26.62283	25.79249	27.37047
130.8458	57.68115	56.59925	63.91733
1.808018	12.38543	12.14622	13.68673
63.83255	58.31194	58.76395	62.03875
19.41527	6.761888	5.409159	6.525705
2.186452	4.897048	4.255694	5.042689
4.473192	2.124249	1.29493	1.406606
1230.966	267.6985	241.5475	257.5261
1.054908	3.934065	3.130615	2.704164
33.71287	3.852776	5.603612	5.564179
3.875397	0.651442	0.842329	0.911535
5.877862	14.69288	14.40892	15.16367
6.546	3.98891	3.671484	3.304982
2.235373	8.111811	3.863321	4.691135
11.95663	19.62092	18.15822	19.41076
4.539655	1.215074	1.692758	1.384096
1.653907	8.140662	6.593576	8.284747
2.386456	2.956207	2.319928	2.882977
59.41291	29.62002	29.08129	29.70564
0.146184	0.648795	0.911913	0.830007
5.628858	3.672092	3.384351	3.608588
0.680175	0.184292	0.557172	0.461523
11.57169	26.90505	24.02274	26.72516
10.77167	30.50723	33.09757	33.06607

29.76847	10.48232	10.52409	10.36123
0.89371	4.048698	4.771702	4.53719
1.48924	5.260316	4.815306	4.635389
9.336295	3.567532	3.288605	3.369944
0	0.258511	0.291281	0.097584
3.064322	0.933579	1.594531	1.266978
3.091863	0.992139	1.470502	1.046328
29.52801	7.062078	7.380292	7.067846
0.887514	2.970471	2.721435	2.851135
13.01459	35.80333	34.75496	33.76263
4.781846	1.40925	0.461951	0.725251
8.471125	15.18093	14.17023	15.4261
2.712919	0.282243	0.03657	0.076175
12.96142	44.81192	46.11104	47.52318
295.4217	118.4146	118.3234	120.7509
39.79267	18.45774	18.65567	19.19019
1.135146	5.593136	6.374487	6.758891
8.222035	82.87301	77.84935	84.42159
7.141425	3.281921	2.758032	3.352602
0.242443	4.704049	6.757501	3.424698
20.77785	10.57491	9.321512	11.41339
1.312639	0.294406	0.201922	0.282332
385.9899	1228.345	1027.17	1000.965
1.565717	3.98077	3.815476	4.051084
22.22881	6.563222	8.85473	6.661292
6.426244	13.7539	14.28253	14.27889
0.317314	2.675258	2.084872	2.979663
0.131343	0.459945	0.384673	0.555586
2.181126	0.544972	0.171742	0.208139
9.125462	0.814441	0.438078	0.483293
9.682488	7.864694	8.225376	7.559016
1.417852	5.43928	4.788455	5.620641
14.3841	4.466053	5.374313	6.264059
12.08508	65.77867	61.94874	63.47797
2.88305	7.661698	7.435065	8.308439
2.622022	10.86814	10.04384	9.717186
1.273659	1.939307	2.232083	2.144682
2.445321	17.28872	16.1114	15.33475
10.42843	32.83496	32.69339	32.4467
2.197372	12.64981	13.27836	12.7349
0.52458	1.829685	1.621451	1.294563
19.31222	9.846301	9.525741	9.029857
3.053433	0.916171	0.738352	1.095557
0.883716	2.837568	4.444805	3.904209
63.66943	31.71229	28.85697	32.13734
2.059896	1.469819	1.006249	0.903005
7.580094	6.045646	5.803156	5.735506
5.176438	4.864078	5.35627	6.545473
1.135621	2.35432	2.337646	2.37809
4.046324	12.36445	11.83926	11.68618
5.62858	8.37919	8.217849	7.832987

2.450886	3.22323	3.164517	3.528939
0.823459	0.066774	0.105357	0.069152
37.24202	61.98605	56.10271	61.17164
4.010231	14.54238	11.96214	14.65833
35.71323	16.14588	16.83819	18.07418
5.091006	2.176618	2.511951	3.282424
1.79412	0.385995	0.83756	0.853494
1.580505	5.305768	7.131387	6.165894
0.370304	1.333765	1.322792	1.196106
197.3994	686.2645	681.9791	710.9351
0.607793	0.705579	0.448042	0.613324
0.942942	2.772631	2.723829	2.765391
0.343323	8.494211	6.697369	6.246892
6.482424	11.08046	12.21062	12.4401
5.844663	1.998311	3.0672	3.52306
0.651662	4.369768	3.575421	2.906125
1.778605	0.994036	0.914595	0.731905
1.352176	4.000327	3.93581	3.856164
25.21588	9.978036	11.43762	12.62593
99.56297	11.22393	10.08299	9.991673
1.036202	3.118533	2.281429	2.633806
9.753516	14.85344	16.78519	16.83997
33.08197	3.149684	3.259098	2.259211
31.40601	14.70928	14.81354	15.33996
117.3447	17.82139	21.52296	19.66653
2.668254	10.95499	10.28627	8.926035
0.229245	0.497374	0.755766	0.776241
6.399963	2.122166	1.326369	2.341345
59.28784	11.68058	9.656381	10.38656
15.36731	7.452923	6.601626	6.466645
1.877845	7.026664	8.237023	7.902562
28.00982	37.15159	36.34551	36.46177
65.50356	20.68826	25.3417	27.28292
213.7695	75.15172	75.53236	77.73866
0.642889	4.143229	4.948612	3.766294
4.630943	0.550725	0	0.322875
8.549273	2.503328	2.162187	2.82563
5.791484	14.96087	13.95046	15.47339
9.377378	35.95145	32.9562	36.31956
3.216845	2.064633	2.397712	2.643888
124.5992	15.1725	15.1587	15.62028
5.775319	24.60247	21.40348	24.50673
9.616984	2.294668	3.293351	4.207852
10.83185	6.13129	5.199169	5.609129
128.9961	28.9583	31.81781	31.43945
43.767	23.58507	22.09809	20.37723
0.311112	0.853663	0.842317	1.07586
8.212954	28.82506	26.9497	26.56944
11.39396	12.69444	11.24316	12.75073
2.781176	0.243935	0.17325	0.098058
94.26868	37.43265	36.41898	39.17481

46.7872	10.22494	9.688643	10.13817
134.3817	44.36327	47.06219	48.70887
0.555136	0.744364	1.089454	1.459016
10.75022	5.024872	4.42847	4.154367
1.289319	0.756698	0.497354	0.897793
28.10821	12.92179	12.94496	12.63461
1452.365	406.6451	422.7795	424.1106
1.684788	6.597511	6.84626	7.137598
0.306261	0.577661	0.773202	0.879274
11.16769	7.015516	6.883324	7.305387
0.705645	3.04837	1.984525	2.792059
0.288826	1.475631	1.918347	1.79549
30.90718	15.83395	14.96789	14.32807
1282.162	173.5687	156.5057	178.2672
0.219882	1.48265	1.492423	2.066925
1.029943	1.054441	0.826146	0.946629
119.8584	173.636	179.8115	184.5965
8.757356	21.14996	17.90755	18.04058
0.366298	1.359799	1.550985	1.585828
33.4689	8.944835	8.216244	8.33246
2.061557	14.27545	12.26254	15.67205
3.070513	2.141343	1.810532	2.359823
6.914477	17.56931	17.06019	18.21978
5.007558	16.56716	12.67693	13.77176
1.760156	7.536136	6.028275	7.39677
0.963951	1.284551	0.991495	2.056388
48.45892	26.49682	28.28848	27.54411
289.5325	74.74555	75.45073	72.98499
15.54331	8.659034	7.574927	7.487334
3.295707	12.55605	11.92391	11.93081
0.687014	1.915522	1.978644	1.918589
1.054573	3.15087	2.373552	3.019417
44.85647	15.66347	14.51448	16.7547
86.44048	46.94023	44.92456	47.81294
3.602551	4.63565	6.039647	5.720205
22.21449	10.80332	11.43484	11.52415
16.77133	4.400673	3.936001	4.362948
98.40843	46.31559	41.99949	49.40227
27.51379	10.12833	9.184241	10.60098
28.2631	61.79627	65.86562	71.84755
111.3619	46.55998	45.76001	49.76417
61.52931	9.23819	10.21089	9.851645
10.32298	30.90879	29.26222	31.17005
0.327181	1.205317	1.511123	0.954992
6.526803	13.28457	12.98706	12.74574
7.647353	18.58691	17.1907	18.47603
80.93246	31.14809	33.38257	33.81474
0.140827	1.531261	1.753047	1.395581
5.66016	1.744231	1.58693	1.709509
8.248566	12.43203	12.90691	13.56564
6.803476	20.61279	19.37255	17.29482

26.20218	12.02621	12.40195	12.95237
20.44626	4.803158	3.963832	4.318411
0.35086	1.328251	3.026731	0.975999
12.39573	3.141274	3.075263	1.91797
23.89287	10.7579	10.90958	12.25629
4.050393	7.765146	7.580326	8.58755
18.17707	5.752747	5.651326	6.017782
0.57622	3.906665	4.66678	3.297252
4.404252	0.132341	0.1325	0.049533
5.47559	0.901277	0.853227	1.926919
13.83101	17.8543	15.51207	15.19541
0.148813	4.801806	3.158138	3.206199
0.447424	1.089512	0.667066	0.793215
0.108283	2.294857	1.681106	2.254061
7.223571	1.932605	2.324849	3.534519
1744.97	418.5338	415.42	429.2775
4.336708	2.278828	2.252535	1.4995
2.643053	3.297028	4.158135	3.127032
2.957323	10.53508	10.83232	12.15778
4.017006	13.06235	14.05768	14.86049
6.290234	14.87698	12.6859	15.18341
6.117746	12.45059	10.62933	10.36134
5.552221	1.724058	2.795456	1.340855
1.113134	0.160935	0.526736	0.410569
1.29552	3.494212	3.941643	3.219585
8.428252	1.219352	0.801079	0.566641
11.84423	5.035116	5.063008	5.454309
22.49274	26.58654	32.35889	32.68166
53.783	11.48097	8.991823	9.065348
19.03828	8.607274	8.726875	11.13783
27.8234	12.64362	14.19576	13.45296
15.0232	5.966846	7.12897	7.564982
26.93873	12.31523	12.92471	13.55883
36.83156	7.687615	7.913734	8.054827
0.526281	0.92761	0.792294	1.04357
8.25487	15.46849	17.3668	18.82218
25.01581	24.00507	28.88254	26.0319
7.323192	18.83624	18.65638	16.99397
0.995396	0.315685	0.239113	0.237206
0.04203	1.056884	1.342357	0.900614
0.709956	0.873931	0.404792	0.809492
6.790598	15.1929	14.93125	14.74147
6.972275	1.254721	2.153371	2.469733
6.540812	19.85956	20.47783	18.93798
4.209358	9.27142	10.71929	10.00486
5.187015	0.439979	0.602085	0.557766
7.252312	24.58497	23.76244	25.00518
0.507365	1.085894	1.207155	0.973269
82.63313	26.82058	29.82269	30.15926
0.89403	1.509258	1.807978	1.557022
26.88672	7.965049	8.69202	10.70235

23.55166	15.45961	16.83768	16.83328
17.36605	35.51516	36.51252	36.98829
0	0.45116	0.336897	0.317731
1.556171	1.921073	1.874231	1.725974
1.410434	1.869011	1.873947	1.82672
0.711433	2.813365	2.360107	2.292537
1.405608	1.598903	1.596125	1.678332
0.224523	1.306164	1.711071	1.482733
17.40203	8.297386	8.014173	9.79431
4.488234	2.287121	1.927231	1.493386
0.043906	0.20173	0.176776	0.520332
0.167703	4.375414	3.501312	3.21648
2.065254	0.783551	1.245883	1.449862
32.39141	4.882351	4.324781	4.908433
12.56641	3.141135	3.965368	3.512174
0.602974	180.6512	182.9439	179.0385
33.60692	6.117932	6.616679	5.651597
0.698301	4.079246	5.704478	5.523881
3.101043	0.924651	0.63302	0.51193
273.1087	64.92894	74.61959	66.55573
36.41908	12.57355	12.32157	11.9729
1.748238	3.382066	3.179198	3.733322
8.267056	47.44112	43.8851	47.5016
1.225929	2.832847	2.404221	2.147575
289.442	89.93224	87.87169	92.55901
10.66864	3.473722	4.515691	3.557308
0.536132	0.084089	0.180554	0.127557
27.88768	10.61868	10.3175	12.62968
0.945143	1.794834	1.157856	1.778987
87.17762	26.7984	28.13826	28.2524
0.591428	0.080934	0.064771	0.232952
21.0831	22.42043	21.26923	21.97085
0.727953	1.162333	1.681335	1.349428
2.851697	4.5335	4.800916	4.235256
17.14828	12.93764	10.81202	10.58586
14.17665	5.624105	4.042326	5.339319
11.1094	31.13739	27.08259	28.90302
583.5586	162.0684	160.9016	168.3392
0.838754	0.211054	0.503849	0.790212
309.7481	115.8508	114.2049	113.8059
4.254897	12.16141	9.96599	12.34748
1.227772	0.349285	0.630602	0.15145
1.406209	0.747548	0.669321	0.977426
15.24305	75.88751	73.05087	81.46531
11.68152	33.12383	30.23086	34.58625
17.66663	14.32629	15.49953	14.80009
0.435675	21.58054	21.53653	22.52046
1.367793	0.590801	0.695326	0.748383
8.263727	3.782801	4.149994	4.006113
1.986121	10.68306	9.665125	9.087141
9.467199	2.317949	2.345206	3.051857

7.017924	5.877003	5.425001	5.127559
0.184926	7.82258	7.884282	8.499664
87.89843	34.90045	40.19694	41.34774
5.151673	1.316021	2.472378	2.675139
0.788756	0.515945	0.308017	0.493396
4.058946	2.204239	1.43561	2.461167
41.319	15.58206	14.92918	14.8534
4.106621	10.07492	10.84781	11.52815
29.98198	50.87875	48.39828	52.28693
0.278398	1.173054	0.83815	1.187189
2.334263	1.446857	1.28517	1.56634
23.2746	53.981	53.87027	57.56623
14.45921	3.582805	4.255874	4.710003
45.77025	240.5875	233.3361	244.2101
16.08221	4.212479	3.866022	3.475766
25.03631	83.65834	80.66863	82.5621
1.090157	3.84575	3.653234	3.98281
50.34064	10.80769	10.94192	11.671
0.955927	2.130857	2.311429	2.523403
2.49659	1.249378	0.88543	0.923937
37.12779	13.61727	14.044	13.1293
7.363604	5.041888	3.934894	4.436336
12.07096	15.59901	14.44423	16.48944
4.247697	15.53211	15.64055	12.534
1.445539	5.658809	4.206677	5.314189
1.827576	0.956964	0.575596	0.750805
5.55606	6.264887	7.159118	5.457363
278.7279	77.99165	77.26241	78.79498
6.715117	2.944712	3.252253	3.667994
2.47819	1.391259	1.683979	1.986349
3.784365	10.40847	10.17229	13.2559
46.64185	47.88027	43.97571	44.5237
0	0.583896	0.449774	0.325844
7.333434	3.185492	3.087024	3.243341
9.752554	3.222078	3.081551	2.948671
6.382893	1.876797	3.357072	3.837035
0.033692	0.179639	0.216016	0.274343
0.198408	3.036913	3.221597	3.240881
0.552646	0.199267	0.299595	0.277734
26.988	10.9165	9.999967	10.3033
4.634703	1.639614	1.39009	1.654866
0	0.234394	0.805009	0.263232
1.001527	0.261696	0.410632	0.813016
56.69667	73.15115	72.42769	75.05553
66.50062	30.40404	24.03569	24.69165
6.78071	1.634617	2.405751	2.339689
4.320736	2.247937	1.448664	1.256247
801.6876	273.6427	267.9495	276.0609
25.86391	44.99476	51.32744	53.41548
34.96744	13.93352	14.2066	15.403
7.844911	0.572334	0.531851	0.92658

79.71618	232.3706	254.9521	247.4783
2.100851	7.31164	8.032834	7.711128
2.84906	3.315298	3.430921	3.303022
1.589879	2.784141	3.417332	3.78029
0.807558	13.40805	10.92681	13.09609
1.066366	1.747846	1.608423	1.720235
13.01442	55.75985	57.24668	54.78802
3.703876	7.30481	6.776888	7.53608
8.343039	37.57713	36.28703	35.74364
5.641659	2.390633	1.498419	2.015747
8.132102	16.6565	17.05797	16.89172
2.393997	2.437516	2.686066	2.425516
25.09191	56.95029	55.25667	59.54911
8.642957	19.99247	19.35609	20.02607
9.126093	3.009704	3.528319	3.303128
0.681246	3.092372	3.165129	4.915468
0.595823	2.106503	2.94626	3.242341
12.31881	5.811119	5.995081	6.554454
26.98142	12.63797	13.38525	13.17298
24.01071	10.93164	8.715145	9.198792
6.270636	2.064002	1.051648	2.302027
210.2849	56.63431	56.07251	52.96502
1.761705	1.711145	1.19861	0.993004
0.714118	2.762854	2.943821	2.151576
1.869168	5.682553	5.482517	4.18895
2.619022	2.085196	2.680378	1.77567
8.682788	20.28559	21.59833	23.12563
41.80071	19.121	16.69434	19.75554
50.61979	23.6688	23.13689	23.96339
19.62302	72.25903	70.80385	71.29049
12.71881	19.93267	19.17623	19.30442
62.69013	23.38591	26.00181	24.66672
2.456252	4.538961	4.729863	4.500867
25.01648	12.16513	13.95595	12.77334
2.214636	1.732835	1.724723	1.974276
1.662727	7.713372	7.000839	7.085501
53.36704	121.7361	117.2883	123.8696
4.197699	3.869647	2.794736	2.732503
7.755222	4.186925	3.043175	3.629371
0.684465	2.075139	1.742733	1.416272
54.6204	20.7461	18.09675	18.88199
1.676046	0.639226	0.67772	0.566915
13.99255	6.754142	6.891142	7.420036
5.816184	1.305064	0.992359	1.187922
49.82211	23.47637	22.13804	23.56056
9.44434	3.269169	3.246831	3.979881
0.520464	18.77498	17.7382	18.01031
0.18598	0.658115	0.630232	0.507618
1.335168	3.334441	3.480739	3.764691
23.80678	36.58504	37.12183	38.79927
4.924812	2.838475	2.616848	2.611416

0.972012	11.99662	8.329817	11.07826
15.12341	4.532893	5.075409	3.860958
788.4718	239.9886	227.5476	240.3817
22.33314	4.400705	4.728449	4.389657
26.46243	11.44619	10.18826	11.8789
3.796432	12.13844	11.5186	12.72737
73.55144	393.9211	412.0583	412.9198
9.264987	10.09334	9.319781	9.282484
0.758428	1.206908	1.305634	0.874425
2.139278	3.342744	3.123506	3.770341
8.571987	18.20234	18.15916	18.83039
0.181432	1.119454	1.231959	0.746888
3.580367	0.467814	0.488278	0.715574
3.90012	4.593046	6.208836	4.504789
3.179904	14.03749	12.58866	15.2746
5.63718	14.99068	17.27003	15.4137
248.4216	77.53534	75.59476	78.12057
0.978947	3.251915	3.394854	3.041844
0.629181	2.197434	2.253259	2.447103
99.56187	14.15684	15.3101	16.43978
0.67285	1.26649	1.452399	1.352369
1.780484	3.745416	4.572126	4.40976
8.609571	12.2229	12.31022	10.48255
283.8803	120.4087	128.3691	132.3507
12.96795	6.198835	5.634512	6.173534
10.52207	20.82914	19.03072	19.61104
0.446189	1.509669	1.747876	1.176614
6.869508	2.976249	4.066443	2.043359
4.959892	2.813971	2.931682	3.276909
40.60127	192.8553	161.3701	178.36
69.80227	100.4513	96.53249	94.45065
0.807841	2.675405	2.388988	2.22729
21.10153	7.500196	6.346993	7.949419
11.50544	3.307443	4.77864	4.092193
42.38664	20.8925	19.40696	20.64917
1.026274	2.211774	5.267853	1.813558
52.92728	8.885819	10.20747	8.134052
0.52228	0.884427	0.765909	0.45831
25.74793	11.24947	10.14439	10.59615
103.1195	40.0644	39.43279	40.70503
0.225736	1.138582	0.888909	0.945986
1.304391	5.252314	5.62471	5.804598
0.479946	0.062434	0.20373	0.129669
101.5642	31.02271	31.25591	30.75277
0.654991	2.696449	2.69562	2.625952
5.231422	2.9858	2.470103	3.003393
188.9991	27.84849	23.47799	30.22612
10.0872	18.70035	17.91005	17.8125
13.93874	44.7306	45.37778	45.33334
1.937178	3.287074	3.626484	3.534061
2.011364	6.318988	4.966197	6.422752

0.583299	2.592716	2.087361	2.872689
6.257079	1.099491	0.773591	1.382039
16.33554	41.15841	39.75454	39.38203
5.861183	2.303336	1.922341	2.270601
1.045266	3.759898	3.887538	3.40192
1.902964	6.565575	8.951073	6.899461
47.91413	25.06021	20.78693	21.84047
7.092371	3.59916	3.813788	3.770786
24.59143	27.37319	25.31919	26.98207
1.663688	3.591697	3.505972	4.120186
1820.251	96.4475	89.30788	92.77987
137.3135	49.20506	52.1856	54.82178
50.7633	32.36652	25.98863	26.70701
3.882654	12.42837	9.661597	10.85214
3.223001	7.514958	9.141812	8.519951
4.581872	33.95108	30.78347	30.99139
45.37288	22.04624	20.17885	20.76622
39.09186	15.13616	13.21127	13.83227
4.098765	2.301112	1.963267	2.495755
19.15031	3.716232	2.372669	3.753808
517.8465	2042.69	1875.615	2006.536
139.8926	35.48822	39.59021	35.52191
4.816859	1.49981	1.480753	1.366181
6.118931	3.202034	3.545727	3.418159
2.910965	1.262549	1.602104	1.504806
15.5752	6.298428	6.60313	7.811429
4.986842	8.065222	10.3957	8.870726
2.523772	5.090245	5.357246	7.980126
1.172216	0.456086	0.397038	0.57038
0.568182	1.393688	1.239294	1.524797
15.35307	9.905579	9.015059	9.244311
15.16713	45.96332	46.65562	46.4009
17.28129	6.055186	5.559109	6.4832
11.02145	47.21132	47.91153	48.16724
196.1172	69.58903	69.5342	71.62434
1.689959	1.687815	1.894321	1.206674
11.72079	3.951469	4.507459	3.932481
0.085891	0.470156	0.596167	0.699673
0.335689	0.87058	1.034389	0.663871
274.2715	127.8866	122.765	126.8524
15.67905	47.34517	49.04673	49.74688
8.480176	2.351384	4.5787	3.722253
0.748274	17.17178	17.37603	17.64012
2.429757	2.924105	4.145586	4.620047
3.210596	0.48102	0.70052	0.643769
1.330689	4.114542	3.448446	5.332155
1.831435	10.15023	8.14908	9.761231
33.8564	15.62797	15.13434	16.3821
450.463	169.6627	171.1997	177.6218
0.946808	7.986625	7.69349	9.423811
7.452267	1.28399	1.130722	1.304453

1.7586	0.435773	0.737958	0.533041
0.347206	0.04548	0.215461	0.150835
14.16407	15.64942	17.08551	17.49393
104.9044	173.5416	161.7218	167.7193
3.154194	8.417908	7.299907	9.624866
99.65934	8.277616	7.608067	6.582171
4.047968	23.48692	20.64779	23.99555
0.869512	7.166272	8.315048	8.895733
17.17546	57.78056	57.71046	57.41308
188.3363	32.32782	35.34769	33.83296
3.211481	5.295537	6.029332	5.549885
0.875366	2.822067	2.881729	3.245459
257.2472	453.4299	447.0445	461.517
8.192071	5.538092	5.748705	5.451992
12.34406	5.192993	5.628394	3.907566
6.794648	25.57872	23.63627	26.16383
0.402531	1.178958	1.447477	1.604926
12.35757	6.642713	6.684701	7.19918
4.292989	11.60572	13.43645	11.30547
0.758447	0.183986	0.343951	0.297589
0.598627	0.660221	0.391842	0.77265
5.764849	14.90684	14.3529	15.47607
1.011086	1.918465	2.395371	2.378606
3.605156	2.544398	1.656897	1.814749
34.76305	12.72475	12.70045	14.47275
21.00181	25.44809	23.57158	20.7586
3.751661	6.685212	7.080478	8.05414
2.241702	0.566487	0.379091	0.933411
46.0618	24.30985	24.23807	27.63995
4.09013	1.664355	1.634578	1.511188
34.29471	17.80002	16.11954	18.08336
1.046419	5.009063	4.694875	5.047009
1.98791	11.41566	11.64852	13.17485
8.341938	3.095614	3.560885	3.707809
2.481958	4.696563	4.747877	4.604165
3.725777	1.546682	1.718465	1.269487
33.9071	120.1521	122.9118	119.1025
13.74028	61.32159	57.66242	62.15204
30.67868	1.489278	1.499877	1.639752
35.49941	10.81816	8.650427	10.065
38.91378	10.76103	9.751403	10.47279
25.23957	10.7544	11.11242	10.84534
0.626147	1.508211	0.83463	1.283066
5.528546	10.19695	10.16701	9.985027
8.244234	16.17226	16.03655	16.95741
176.1499	47.34706	46.50671	50.41654
0.026462	0.04333	0.090682	0.101189
0.382018	1.753564	1.368974	0.987972
1.212559	0.342751	0.048264	0.190108
1.171294	2.774369	3.205783	2.746525
1.948858	1.500065	1.352375	1.230436

28.24032	89.68511	89.51319	91.80938
18.78597	44.12881	41.49728	43.73415
0.312553	0.117893	0.093592	0.238531
35.46796	23.62265	21.71008	21.42354
0.681829	2.317951	2.372488	1.393885
2.11992	6.626588	7.189378	8.053274
8.528955	86.50816	80.825	84.21295
0.306206	0.53057	0.16909	0.060176
1.414934	4.54865	4.420136	4.46683
280.9153	774.6389	666.3598	747.5411
12.53291	7.40302	7.48296	8.435293
46.53014	8.032504	8.205881	8.130941
30.67089	13.98268	13.00514	13.3293
13.96236	5.807256	4.845901	6.132475
2.807672	0.748457	1.150766	0.960098
18.37991	38.26281	35.59523	40.73569
3.517039	3.1855	3.77806	3.542697
0.719264	4.576829	4.763177	4.191912
15.0567	6.771662	5.981102	5.266383
11.29891	3.21856	2.815749	3.540854
19.31174	38.94089	41.46588	38.31048
35.03016	15.07816	15.70144	16.05882
12.55326	20.95317	18.76367	21.28294
24.489	10.4574	10.74308	10.77066
4.567863	9.228725	10.45285	9.399151
0.810538	3.180443	4.866871	4.66093
0	0.419921	0.350976	0.501862
0.915895	87.92607	88.28759	92.98623
0.222632	2.676104	2.339985	1.530044
66.54769	35.45062	29.46471	32.45139
0.095639	2.073595	1.564549	1.300393
8.988838	4.529943	4.273421	4.139309
1.088287	2.7584	2.014344	2.807481
12.33034	3.749071	4.372118	5.767346
18.62359	71.06113	66.2073	70.09179
1.059329	4.018584	4.240201	4.953219
15.1782	46.91052	51.59984	51.69645
3.533467	4.847019	5.539823	4.417261
0.904698	0.269027	0.226074	0.303301
7.790885	15.10596	15.61633	16.13714
206.7579	92.83119	82.22938	87.75108
588.7289	250.0857	241.9457	259.2336
10.52912	1.73236	1.751512	1.796993
32.66278	15.22746	16.7142	15.21558
12.94986	30.26951	30.15885	29.79878
58.60205	9.840338	9.417742	11.10237
70.5574	18.60265	22.30053	20.71853
0.351151	1.273276	0.776816	1.064159
1.944743	6.194089	8.542829	8.138442
1.801021	4.471252	4.536092	3.859022
25.36373	4.632295	5.803951	5.364969

0.411286	0.847986	0.749078	1.579651
127.5387	391.2593	380.3701	409.2015
3.461249	1.919639	1.415958	2.253943
0.721465	0.434478	0.242601	0.905655
0	0	0	0
4.045966	16.17712	17.18445	18.71826
18.19949	8.4189	7.421791	8.409386
4.526905	2.270248	1.863289	2.558396
1.665565	0.593019	0.312051	0.546878
2.145715	0.134207	0.82073	0.419507
1.801347	0.644753	0.580729	0.271942
9.377051	50.82695	50.2098	53.48256
2.61165	3.155819	3.553993	2.931563
2.551885	0.899457	1.079704	1.202766
749.3128	20.51126	20.40527	20.22559
49.31124	23.22715	22.42317	24.46954
115.8084	57.52513	54.30945	59.14178
7.153037	4.062212	5.182955	4.772712
0.778381	15.41348	16.13099	15.11011
3.335969	22.96719	23.56748	23.93097
1.557972	0.87369	0.470758	0.960548
1.504822	5.575098	6.180055	5.929566
0.525469	2.832817	2.367143	2.210587
4.110237	1.859592	2.5296	2.429664
0.382079	1.3476	1.349139	1.597432
0.106367	0.208387	0.261325	0.287363
1.407888	3.11116	2.789722	4.245758
17.80535	17.36928	14.9305	16.98275
0.417872	0.794911	0.924195	1.583499
0	2.707684	2.221051	2.749467
0.876719	1.362812	0.863465	0.926794
20.90486	27.44357	23.68817	26.20079
9.802782	2.795719	1.978562	2.855662
0.733423	2.672728	2.926448	2.572024
2.957963	0.195672	0.937474	0.229646
10.04764	6.100833	6.475269	8.006711
38.79531	120.6346	122.7739	135.6526
6.820478	7.219454	6.433427	7.961753
1.187368	3.232301	2.490471	3.020571
6.756501	19.47457	21.57483	17.71513
48.16437	209.7017	200.1238	207.0011
1.066746	1.86777	1.933797	1.984012
20.8663	23.21784	23.54122	23.66405
7.087382	0.763986	0.84433	0.854064
1.144301	0.536483	0.431619	0.335909
0	5.769317	4.676167	5.770283
1.228181	0.245756	0.224986	0.253097
7.300998	32.41283	28.08113	30.5226
6.167049	3.645036	3.532387	3.082259
0.228713	0.788369	0.504151	0.569433
15.70048	5.090578	4.162956	4.193015

1.650436	6.469893	5.406047	4.517854
26.63786	35.21712	36.18682	39.0706
0.63857	0.478229	0.752185	0.783732
11.36424	3.657985	3.11537	4.432539
4.075745	8.26778	8.302394	9.153956
9.554181	0.214586	0.233236	0.186515
32.62992	28.94486	30.38693	28.22059
24.48753	23.64806	19.83004	26.64794
11.12207	28.8363	26.46325	29.17929
19.6336	50.89406	49.21275	51.55579
0.435656	1.075445	0.879583	1.168423
23.43935	4.763442	4.46204	4.634673
730.3404	333.3915	326.4988	338.4956
42.91422	117.324	109.2595	112.3612
1.197568	11.19174	10.689	10.44688
15.72788	5.312299	3.970391	4.118187
55.87048	23.45355	20.99707	23.29811
0.437978	1.200024	1.234257	1.591715
0.473714	0.875504	0.985122	0.71083
16.00475	6.770137	6.965102	7.213441
0.255167	0.781126	0.713722	0.86006
0.329671	2.567761	3.020011	2.31077
144.9233	62.1732	62.54456	68.36724
8.172684	14.91126	12.28083	12.58864
2.521369	14.68472	12.32172	14.14771
1.816064	1.305032	0.95322	0.724082
1.996902	7.946991	7.128064	5.939431
1.555135	21.29796	16.38526	15.06719
14.27315	0.380659	0.508917	0.241953
0.500431	1.383751	1.165096	0.862439
2.601912	4.794198	4.689597	4.626918
4.261724	10.5944	10.72117	10.50363
3.065006	6.610024	4.848521	6.733886
6.017742	3.272002	2.861127	3.307008
4.930331	1.603198	1.693157	1.700182
0.06787	0.503037	0.135458	0.211399
3.916349	3.045978	1.799584	1.723612
12.61057	27.27433	27.06558	26.0475
58.83104	188.9367	183.4199	202.5021
363.8907	128.3017	132.7442	134.5662
4.098421	3.923121	4.250137	4.972048
2.250671	2.745063	1.768896	3.113269
12.80885	1.426318	1.733098	1.649981
4.175316	2.098284	2.81916	2.382486
2.947209	8.799603	8.325119	9.235606
0.917892	4.478612	3.981034	4.145337
1.425471	12.53416	12.37168	13.50463
2.156269	2.225238	2.706156	2.298837
1.729454	23.19694	24.22401	23.90127
2.544297	7.030405	8.830202	7.864549
22.04573	5.237132	4.667047	3.630455

4.290836	2.64802	3.099736	2.543369
0.392419	2.25244	2.651475	1.932613
4.886688	1.992529	2.247783	1.435819
68.44054	31.22417	31.5716	29.7393
84.44521	24.74371	25.82418	24.38779
21.22874	7.629166	8.896299	7.264215
18.42792	49.09643	52.46651	53.81132
0.696407	5.477526	5.600633	6.257584
4.511804	7.915409	6.175051	4.764751
363.3398	176.0078	167.295	184.0361
0.423406	2.078263	3.683307	4.138991
38.44808	15.21244	14.98256	15.68199
9.072172	4.838836	3.847526	5.151292
4.602645	0.683072	0.326605	0.617365
20.07118	54.60296	46.53697	53.33276
1.690992	2.644237	2.925827	3.221757
0.765308	2.878286	2.127898	3.275628
114.4989	1.953855	2.137221	1.575631
3.469522	0.619383	0.641236	0.417785
37.56237	0.578608	0.656501	1.295623
378.8405	145.298	109.738	121.4633
0.327209	2.36504	2.217557	2.328022
6.056139	1.943161	2.401105	2.527141
0.740967	2.901903	2.377794	2.636761
2.330594	3.206425	3.159143	3.284627
8.908736	24.34861	23.2469	23.54561
9.909955	3.086271	4.605565	4.457611
25.53729	11.21487	11.25096	11.66425
39.76875	26.13855	26.88987	29.76876
0.683603	1.223713	1.767832	1.629511
9.003928	40.31197	37.91993	41.91636
10.33019	5.577827	5.197052	5.05244
1.297347	1.025926	1.347794	0.738022
0.479237	0.81104	1.226944	1.722613
8.439967	23.94941	22.46823	22.08517
1.444807	3.795183	4.077661	4.409829
21.92384	9.996147	10.5201	11.64187
6.946115	3.4237	3.107271	2.97104
8.846568	3.771059	3.864875	3.566178
91.23023	39.36628	40.22697	40.2617
161.0519	58.53532	62.67794	60.85685
14.74417	6.626495	5.333323	6.764244
1.59396	0.778289	0.579162	0.434896
113.9885	30.5184	32.7969	32.62459
3.327589	2.022431	1.443453	1.135384
2.948765	11.05038	10.39458	10.71839
2.038342	0	0.0594	0
0.372926	0.414472	0.338477	0.979355
17.4765	35.90511	39.68943	40.04119
211.6693	26.39904	25.65215	27.90033
13.78614	5.225548	5.420353	4.911483

0.25962	0.782084	0.672305	0.712301
2.505909	0.460722	0.647044	0.385129
674.6118	237.826	235.9442	247.0604
1.70131	0.909458	0.753216	0.859764
18.71615	8.274114	8.15774	9.265473
11.50435	34.09365	30.35544	30.32957
11.17279	2.822926	2.622195	3.526535
2.740606	13.47833	13.66481	13.99628
92.4079	46.32375	45.0161	42.50695
75.18717	36.72451	33.70946	39.23485
0.975251	0.894487	1.363076	1.345582
17.81768	8.243872	8.033865	6.910663
28.89376	15.23007	13.6552	13.44545
24.0329	8.663056	8.995164	9.58474
11.79755	4.432894	4.077518	3.761815
0.646598	0.245772	0.130581	0.146887
61.18644	169.2667	160.3821	172.6517
0.090401	2.996729	2.683902	2.641073
1.230776	8.793332	7.644209	7.053402
1.449234	0.585146	0.789387	1.155207
1.524156	0.902281	0.445217	0.568834
7.656144	2.639059	3.072575	2.783166
57.86934	18.0677	18.45038	18.22193
7.063982	18.35455	16.95083	16.39097
8.482626	4.345842	3.679447	4.087057
9.167468	25.69049	26.09941	25.22648
34.38392	13.21877	13.27618	12.74704
0.860657	0.155179	0.765702	0.760674
120.6626	547.1573	523.0721	558.8496
0.646941	0.875148	0.762775	0.829759
11.79869	32.90502	31.28044	33.11345
19.47526	4.965388	5.180037	4.620234
54.39615	22.49957	22.6538	23.39966
1753.333	524.4544	522.3638	546.7659
15.83458	6.645195	5.624865	6.432621
73.627	207.369	209.5435	219.8018
48.07912	80.92954	81.70589	81.85441
1.710424	0.932598	1.263466	1.11121
0.376451	2.176567	2.07924	2.143554
0.873324	4.62852	4.717202	4.73438
7.214069	3.564366	2.99971	4.07064
5.4708	8.723465	9.730765	10.96209
0	0.231731	0.097232	0.33262
18.58683	169.7446	172.6513	175.4925
8921.445	3361.471	3375.687	3436.349
2.040453	13.17522	15.75775	15.09005
3.211637	7.132645	6.999018	7.673038
1.78282	0.492431	0.290186	0.261087
5.223089	2.069947	2.097968	2.41853
2.76181	20.61095	19.51474	20.15058
5.103253	17.26147	16.48852	17.26741

0.31726	0.6348	1.021422	1.122129
9.435607	11.64755	11.46921	12.33934
112.9961	33.57618	32.40377	33.83278
160.6393	31.05032	29.52869	30.7238
30.35036	12.19106	14.21566	12.95025
28.23419	5.843378	6.170338	7.788462
0.952258	2.341695	1.475143	1.659893
5.102929	2.936585	2.544785	2.335662
5.518879	16.608	15.97863	15.92648
0.092003	1.670062	1.678803	1.772797
0.369915	0.156661	0.154443	0.116758
1.32305	7.636693	7.528414	7.431635
0.029342	1.548986	1.742971	1.725019
8.662472	24.07416	20.01215	24.12416
36.77944	23.30578	21.12017	23.03739
5.14494	36.47981	37.82593	39.11769
0.732846	8.694286	8.705746	9.26786
1.208395	2.188287	3.224609	2.672117
3.26059	21.94382	20.93455	22.14687
3.707873	5.814289	4.899843	5.50617
44.33443	20.70272	23.70184	24.0534
1.529622	0.135369	0.223673	0.146543
43.33731	14.74248	14.00321	17.2677
0.36158	0.605489	0.5193	0.917732
0.11565	0.470554	0.683705	0.248731
0	1.476237	0.788371	1.487993
15.74187	4.965419	4.426291	5.155962
0.05305	0.360226	0.376557	0.472862
59.38325	20.62692	20.42253	20.80681
45.65122	15.94378	17.41337	16.90805
4.602818	22.14606	20.83775	22.88291
0.232859	2.386808	2.098354	1.949448
192.3371	73.85035	68.22768	75.26222
30.08257	17.21002	15.14003	15.25458
2.030452	0.070575	0.163295	0.295014
1.623513	0.285743	0.367288	0.199737
18.49098	0.96412	0.637924	0.984759
2.171545	7.193575	6.774936	7.638287
2.414364	1.888785	1.696808	1.762521
6.216075	3.494782	3.182541	4.608319
4.882787	20.97531	20.25555	19.73913
1.723253	5.648586	6.430254	6.294598
252.159	1148.595	1127.059	1180.99
72.72487	34.9868	32.37507	34.82135
18.16171	8.415756	8.688615	7.909973
6.512994	49.28791	51.21997	51.21096
0.492633	0.267915	0.623246	1.15848
4.767794	17.68919	17.44542	19.91268
0.064617	0.79116	0.756067	0.996444
21.71927	9.226441	11.06234	8.685449
1.794285	3.877919	4.51705	4.798052

0.636067	1.689758	2.224088	2.513865
36.94038	59.64616	56.54969	60.92555
71.75663	13.23874	13.16888	15.46285
20.83604	24.14203	22.23978	22.93074
1.117657	0.663619	1.265594	1.556239
4.999713	2.465191	2.328167	1.784902
5.518907	6.790308	8.613446	7.210897
2.597712	0.985486	0.610176	0.918196
6.165357	2.576516	1.966923	2.589948
2.483263	11.20492	12.28634	12.69682
18.31003	0.8468	1.237573	0.795543
1.698969	4.96949	4.595818	4.53205
0.817103	0.515459	0.259878	0.321771
0.518041	1.546281	1.350006	1.551267
2.313747	10.19258	11.84847	11.51436
2.080477	7.392927	6.340386	7.50273
110.0726	47.53151	51.29122	48.42119
25.96533	2.522582	2.936519	3.470127
7.407187	3.614805	2.560477	3.068268
1.220393	0.7889	0.628406	0.395467
14.53781	34.88095	36.39319	37.34785
27.21815	83.41234	83.13145	84.57668
3.251196	36.57826	34.7638	34.49608
9.568221	9.007126	8.733172	8.515059
0.849005	3.375932	3.474388	3.276743
0.64943	1.352632	1.454408	1.895225
1.285186	0.400506	0.278506	0.510931
8.950906	21.74711	19.87223	20.35019
5.324123	2.824558	2.608509	3.024047
35.52222	33.37848	37.38768	34.07893
54.581	24.42688	24.53656	26.54345
7.385272	2.806764	2.240019	2.15156
1.226891	0.312068	0.478173	0.645485
8.775994	19.11003	17.99582	16.97937
5.284219	0.144638	0.440258	0.199861
13.18121	5.11433	4.832772	4.306012
2.122946	7.699658	7.57079	9.099212
29.56818	10.37348	11.45788	11.79164
0.923948	1.49803	2.179648	2.030243
76.08698	112.734	108.3725	117.1517
3.923036	12.11243	12.56041	11.7984
181.9239	48.74003	45.53548	49.92241
7.04838	6.657563	6.801283	6.171498
2.524891	2.115179	1.952483	2.687558
8.543386	5.05051	4.479837	5.715026
2.076002	1.894759	1.676484	1.771482
2.963093	1.333234	1.231506	1.350712
14.09442	30.68025	29.03034	33.05939
2.926679	8.02384	7.284106	6.349041
53.03785	13.15187	12.30488	15.85391
0.539082	0.899866	1.261307	1.025779

0.230476	1.479473	1.191648	1.186257
0.498976	2.154891	1.817735	2.388909
5.228618	11.00758	11.67856	11.98663
20.82304	5.335898	4.873275	5.826033
265.1518	112.377	98.16956	105.2302
16.21413	7.208242	7.767838	7.113361
0.69571	1.620755	1.386098	2.108253
203.3526	62.75726	59.06636	59.80739
2.266869	4.557829	5.204777	4.756233
2.761168	2.019311	1.983107	2.489642
1.716204	4.430893	4.621664	4.983742
1.068207	0.458868	0.142459	0.235291
0.350632	0.645874	0.835059	0.64417
212.5924	100.0245	93.01955	98.67313
46.31309	22.92981	20.72766	21.33859
7.619181	60.32451	58.68905	62.38671
11.65958	5.665041	5.346023	4.993156
5.268288	15.57769	17.1973	16.26314
2.998413	7.696657	7.092247	6.623423
2.397659	5.221879	3.34092	3.837701
150.0669	28.97303	23.05592	26.7582
6.724388	3.897755	3.424892	3.52665
3.960655	15.25724	14.71062	14.87461
6.874758	24.75462	26.62551	29.43079
1.567311	3.308907	3.614817	3.472698
1.129406	0.920778	0.934114	0.846585
0.413686	1.2553	0.773569	1.031149
5.839288	11.90823	10.48572	10.20243
3.543721	1.502535	1.046037	1.167357
0	1.8824	1.797045	1.067297
2.813544	4.617393	3.913047	4.786176
3.348378	5.621142	4.992842	5.004979
6.771236	1.61788	3.189184	2.616063
0.488651	2.569609	2.997112	3.129435
63.59034	273.2163	261.5251	260.5805
11.24587	9.731356	8.668987	7.494772
10.64199	16.97225	19.2355	18.7024
68.63068	19.80874	18.49923	20.66884
2.018501	0.755351	1.090515	1.000434
1.362809	0.997099	1.501225	1.457397
104.7796	136.9703	131.1098	137.2567
296.2685	101.6586	102.9342	116.6307
24.04714	36.90842	37.64487	37.93555
45.82717	8.772986	10.39222	9.246421
0.684581	8.731846	8.594827	8.144794
6.006113	1.736184	2.540409	2.46186
0.369456	1.498164	1.20575	1.247563
1.457701	17.44391	18.05713	20.72135
6.650744	1.606303	1.692523	1.87399
1.734918	3.973261	4.28412	4.144537
8.448137	1.649219	1.809274	2.10426

42.32468	15.6453	12.49959	13.97785
4.716844	14.66115	13.70587	14.42496
0.718679	3.346752	2.631686	3.305643
4.047809	1.298397	1.244559	1.344695
12.48851	4.216628	4.927842	5.222309
0.818123	1.443224	1.556393	1.838331
1.326819	0.446435	0.562215	1.042537
15.57589	61.45659	59.57258	63.66007
8.411882	6.068718	5.60024	6.181767
7.405352	3.156101	3.635537	3.280831
9.187541	20.63501	19.09784	20.92135
0.052502	0.12354	0.204069	0.32284
0.504572	1.683342	1.380175	1.46237
0.787704	1.798231	0.983353	1.193752
1.653168	1.309748	1.687099	1.514161
0.321343	1.06798	0.896329	0.712492
0.37735	1.484013	1.193067	0.864088
11.18315	4.948119	4.526252	5.585598
2.14638	1.75065	2.01431	2.050614
2.092371	11.89885	12.00632	10.81357
9.324064	30.80263	35.56197	32.54343
0.892032	2.457932	1.903791	2.263795
52.22814	103.1393	98.07274	104.3613
5.256801	14.30068	13.94097	13.41207
15.54761	5.316843	6.082882	6.136959
274.6647	102.7907	105.4068	109.2417
0.230002	0.852434	0.840771	0.524238
0.349867	0.505862	0.126078	0.331444
1.961575	1.347538	1.623992	1.349186
238.9312	150.7718	153.9439	159.7674
5.875378	8.168363	7.791918	8.422151
11.87561	4.380853	3.859778	4.411614
3.436324	0.860094	1.064699	1.585929
1.082736	0.543179	0.550393	0.635376
2.376215	3.8962	5.231493	5.522799
309.8599	936.7665	905.7893	993.3367
2.214599	7.069108	5.662353	6.78301
3.802902	0.136478	0.316051	0.099109
10.94205	4.848501	5.474609	5.137392
66.08837	27.63067	24.75303	26.96019
545.0428	215.155	208.672	215.0631
249.6806	68.68321	73.09217	66.66577
37.12014	12.89317	12.11544	11.97421
44.92452	21.09506	21.45412	21.68563
0.279015	0.540549	0.912046	0.487145
5.158459	1.931808	1.689057	1.515848
10.64461	31.66772	32.00363	30.49823
15.02847	14.09463	13.92011	14.55191
1.514591	7.675394	6.784519	7.490897
0.927808	0.561848	0.725994	0.656227
0.201219	1.508675	1.195337	1.523101

3.417113	1.31852	1.374025	1.153319
3.242646	1.312699	0.885547	1.436625
295.2231	107.8787	99.55318	109.6311
18.20971	69.02208	70.9055	69.88648
4.715455	2.137301	2.309154	2.520173
2.373955	1.28862	0.640569	0.909408
1.954876	4.169174	4.705291	5.527225
0.652816	1.071058	1.400263	1.187057
134.902	45.14192	41.57552	46.01419
6.580513	1.967126	1.11986	1.848254
27.19553	5.744951	5.36786	4.948972
23.91089	62.24065	62.94443	59.78908
9.329429	31.85195	32.04714	34.22902
20.62195	8.2499	8.220971	7.991698
6.934922	7.861369	7.123337	9.400258
0.891311	2.232619	2.363065	1.705421
0.799889	3.365588	1.80056	3.287452
28.52396	31.63537	34.52371	32.51473
1.042124	0.982643	1.623866	1.265185
42.20965	6.801432	6.460059	7.807144
86.74393	209.9413	211.8013	182.9526
12.51376	3.078045	3.292241	2.697311
62.28741	15.91459	14.85836	16.6661
3.467306	6.981426	7.548242	7.651486
6.872774	2.5969	1.941388	2.514066
24.07392	8.89961	6.481662	7.475122
9.266665	21.85466	19.43731	19.0331
6.445498	0.62115	1.017516	0.45781
1.069038	2.330396	2.061346	2.871574
0.893075	3.290744	3.088933	3.899577
7.956752	3.689412	3.786881	3.034564
4.877042	41.71105	37.02552	40.66686
3.17577	11.87275	10.85566	10.32438
18.52543	10.15437	10.37145	11.89461
0.912043	2.454108	2.522711	2.861289
0.198688	0.242407	0.444432	0.544453
68.44092	28.73924	28.57332	29.50431
3.01121	1.386573	1.379155	1.730811
1.438425	5.129463	5.697258	5.544765
0.919613	3.590226	2.45454	2.408247
12.0905	4.415916	5.888572	4.573811
31.29996	38.01846	40.09214	39.80307
3.47442	1.297994	1.183285	1.59886
25.26817	9.79754	10.5639	11.30175
16.20723	9.084388	8.700136	8.984704
2.283844	6.673449	7.635555	8.486739
14.51747	11.02781	12.17333	11.70955
15.15246	4.69166	4.832712	4.871394
37.58372	86.04262	86.25957	94.01609
10.61978	12.80487	12.57162	13.05313
1.592886	0.3573	0.180311	0.178193

3.836406	20.98396	22.51003	19.15237
0.591159	0.303089	0.161779	0.35042
1.079666	3.521674	4.571482	4.462219
29.91316	14.74188	13.36309	15.25035
0.086859	0.247631	0.303532	0.25709
12.76433	5.650065	6.082057	6.030892
168.9556	76.98517	78.03107	81.12868
5.479136	4.120064	4.169024	4.131752
0.821166	2.42281	1.654737	2.032206
0.691931	1.453627	2.08914	1.291699
55.96935	181.7432	191.586	194.5522
5.386564	23.19623	19.97607	22.72407
62.60631	25.09681	25.04115	28.15873
6.680717	4.591065	5.066161	5.558156
50.79048	27.48577	24.42222	26.23527
63.49294	33.80787	37.51269	33.39512
0.085955	1.025401	1.156052	0.825319
0	0.701078	0.728677	0.496128
10.19088	4.532441	4.437064	4.155734
639.9813	304.0553	306.5681	315.3715
6.727476	19.67414	18.18899	19.20038
1.657768	0.659091	0.791913	1.042456
52.307	17.10423	16.30707	17.29355
2.571914	1.906098	1.953809	2.176191
4.242859	1.356574	1.817547	1.39608
6.310667	2.885713	3.371092	3.541008
59.65006	24.93786	24.31761	25.51166
57.48108	463.0793	449.9385	403.9536
10.50649	4.478124	5.024074	4.983673
37.16665	12.74033	10.33838	10.88228
23.00633	10.41803	11.26353	12.39148
119.8002	55.48588	58.96877	59.09369
0.134084	1.004642	0.842912	0.762265
17.90931	5.381619	4.773009	4.445626
0.957719	5.513569	5.009264	4.642138
0.467259	0.011538	0.054433	0
5.161271	11.51248	10.43003	10.90253
17.39656	0.263812	0.490006	0.083045
4.750057	1.249584	0.779998	0.512556
18.53665	42.16728	40.64301	43.22133
2.208411	0.491802	0.761708	1.359648
144.0395	160.0952	157.4424	173.0797
2.298856	5.723892	5.773561	6.602974
1.059281	1.690307	2.200329	2.70887
7.902597	4.684354	5.499921	3.729148
6.278334	2.346394	2.438761	2.66573
0.890602	5.073315	5.383454	6.220064
6.441272	2.585822	1.778325	1.756539
14.85256	15.27526	16.48256	15.71345
42.03284	9.559967	8.593627	8.645355
4.840972	3.529594	3.645002	3.266377

0.855312	0.742235	0.92433	0.776489
5.276042	6.957519	6.157263	6.622018
22.35326	10.80918	9.790054	10.34339
40.89505	100.1986	98.79036	105.3811
179.566	775.704	788.7508	797.9687
4.259469	1.721506	1.690832	1.529718
105.2498	30.44038	32.31669	28.65049
1.962824	3.848567	3.004243	2.801704
11.24353	18.34015	17.09103	18.52699
27.44169	34.45114	29.98001	35.87314
3.437794	1.015837	2.006324	1.504192
125.3927	52.16008	51.54658	53.80433
0.365395	2.565414	1.908487	1.98391
49.87079	51.4761	50.08648	49.055
0.571798	3.730742	4.516033	3.879766
6.779853	3.432894	3.898174	3.690116
111.4226	48.66609	51.99617	49.72295
13.20062	31.98921	34.51586	34.51363
1.688926	0.532044	0.526164	0.550771
0.237077	0.670004	0.679955	0.413419
1.745708	0.460981	0.701396	0.642501
14.75594	64.74733	53.91542	66.44956
0.606439	1.388806	1.756584	2.359944
132.5868	44.9342	47.33492	49.17095
4.313509	10.77817	10.47002	12.4746
7.45879	6.663464	7.475542	6.75648
1.402444	0.51066	0.697919	0.62243
12.47406	48.11746	46.2587	47.23819
1.679027	8.150809	9.393615	7.397324
6.12577	3.504016	4.014156	2.886126
12.75486	5.549094	2.645096	3.446512
3.850675	3.753755	3.108563	3.777817
17.99482	4.535166	3.959241	5.375341
16.16616	7.193702	7.620341	8.541672
6.208156	9.857947	8.55933	8.805844
15.12808	4.426012	4.403305	4.172111
0.233939	1.790872	1.311265	2.171093
2.799354	18.16936	17.60547	20.16068
6.054606	17.07328	17.42328	18.06924
0.785771	2.845493	3.114867	2.525924
4.213565	1.171208	1.247405	2.132862
0.17678	9.743813	10.13372	9.994178
3.583506	1.389552	1.353135	2.025092
7.229906	27.26609	24.36298	28.02539
1.208178	1.972669	2.25684	2.376317
1.451525	3.194789	3.820691	3.209222
1.946653	19.67583	20.93839	23.54994
22.24457	63.2674	61.93684	66.37804
10.38932	5.435826	5.077748	5.288729
22.50683	4.194616	3.213485	3.55526
14.77389	0.660747	0.298692	0.921114

1.852072	15.88932	15.26516	15.94629
0.428357	1.217463	1.010843	1.449354
1.075911	4.134836	3.539914	3.59035
0.477915	1.311944	1.702973	1.282691
3.635229	3.223299	4.251763	3.985322
95.48558	44.93232	45.24431	44.40717
0.818858	1.709794	1.557946	1.973595
30.42397	6.52424	6.58337	6.969548
5.324522	34.6662	38.23817	34.36686
2.932494	4.416023	4.441793	5.886238
48.87226	177.9677	170.5	178.8702
0.259052	2.435155	1.720445	2.896114
0.538443	1.843162	1.65831	1.789319
1.206582	8.664983	7.805506	7.131622
6.596508	8.033904	7.015167	6.954198
2.858366	26.28662	30.35783	28.83402
39.20838	13.8603	13.24857	16.33662
716.8524	236.2962	231.4465	240.7951
7.177596	18.08614	17.57228	20.05917
0.501284	7.653231	7.935048	6.751399
3.811491	8.781348	9.219767	9.209529
1.84866	3.864718	3.690417	3.246856
1.625673	0.86473	0.886001	0.890891
314.9858	115.1854	118.1344	124.9177
2.235947	3.883579	2.580756	4.067392
0.950011	5.213052	6.032579	5.123486
12.07142	5.650608	5.414013	5.474041
1.16435	2.936211	2.957347	3.511104
1.701614	5.703621	5.555822	5.737597
25.8842	10.54136	9.483971	9.971608
1.473322	1.529126	1.519427	1.847555
9.493938	1.824366	0.901833	1.599654
8.02448	13.80664	14.50552	14.49149
97.13007	36.07408	33.22222	35.05594
0	1.627766	2.20518	2.320985
0.125624	0.443425	0.575223	0.451286
3.352663	0.782086	0.595518	0.541949
3.273953	1.021454	1.386033	1.193674
13.95399	4.37624	4.986646	5.037199
2.853956	8.457302	7.612435	7.600072
6.960548	13.35536	16.05302	15.75353
21.94356	12.89476	12.02674	13.54333
5.93762	3.92555	3.2256	3.255456
2.464362	3.935406	3.79479	4.047261
3.539031	4.874384	4.224437	6.172651
10.27235	23.83542	23.17227	23.58707
0.198364	1.138766	0.784691	1.311636
7.283986	3.955297	4.287144	3.867479
2.930705	7.599222	7.616738	7.506677
0.376108	0.962244	1.144515	1.151678
0.113427	0.795071	0.698729	0.837024

47.39193	13.40578	12.92593	14.63776
2.929772	0.778853	0.840806	0.722509
1.073841	60.71515	62.75761	61.70438
3.4692	4.949663	4.325378	4.561046
1.350813	3.312863	4.316633	3.385984
3.430876	1.452449	1.938786	2.624153
10.93158	2.202275	2.343182	1.76031
0.44235	1.405943	1.614897	1.578288
1.835329	0.54936	0.903346	0.873567
0.423447	0.123289	0.192198	0.155812
0.313192	8.254123	7.132895	7.584786
1.646883	2.681988	1.74761	3.111556
6.506299	2.757159	3.529586	2.917192
0.757458	1.535791	1.764451	1.696033
2.702147	1.399089	0.894046	1.225245
5.536085	15.75629	14.84792	15.71374
10.14779	6.707109	7.650558	8.4953
10.97262	25.99631	25.21739	27.03728
3.526009	3.55414	2.877567	3.385315
105.6627	30.82979	32.73059	31.98563
2.090607	5.25291	6.098251	5.869246
1.96323	2.832887	3.148178	2.825473
313.6324	127.1727	120.1692	127.165
21.88321	82.56526	79.23303	82.49046
7.682448	13.21	11.84241	13.1423
3.079283	4.693397	4.39016	5.655618
5.95647	16.09384	16.76952	16.86432
4.647906	4.792372	3.885608	4.499549
0.888509	8.499605	8.939186	8.970938
2.081462	19.18406	17.35783	17.4181
3.644281	7.996515	7.863666	8.658871
48.79289	21.34797	19.60616	22.09471
0.163297	0.490154	0.551743	0.967996
31.34889	11.29687	10.20482	11.61001
147.3006	22.12301	22.75524	22.25194
26.06016	32.03876	33.56237	34.49338
26.97135	11.47064	11.01507	11.6515
6.387627	26.08752	24.29362	26.69591
0.281854	3.569966	3.284101	3.598153
2.345991	1.260495	1.314234	1.130727
0.153302	0.515395	0.659837	0.664678
0	4.819427	3.105479	3.504712
61.65123	218.9009	212.4792	221.9775
7.61298	18.89615	18.08566	19.29813
3.764614	0	0	0
10.52441	233.782	230.4808	240.2453
0.943692	0.237159	0.281564	0.459744
17.48675	86.83497	80.93636	84.26764
14.29583	14.89922	15.4025	15.14503
0	1.249392	0.676448	1.22576
2.4626	0.533608	0.500434	0.430502

129.4836	149.7115	147.1418	155.3806
3.262781	1.224153	1.201168	1.248631
4.227995	11.06046	11.61081	12.19001
4.304154	6.482475	5.918603	4.503917
0.779701	1.845629	2.109272	1.241566
12.37737	13.60262	13.98667	12.5938
8.447392	1.074056	1.168495	1.518408
0.138896	4.151107	4.623383	4.337365
21.12404	33.37733	33.39059	30.5273
0.439052	2.950773	3.778824	3.968279
4.359807	1.127824	1.43988	1.102209
2.575743	20.58169	22.23539	22.77938
4.720201	11.53395	11.36128	12.06732
12.4402	3.874197	4.255471	3.271478
0.645438	3.057039	2.394304	2.734174
9.868254	2.162724	2.294391	2.733534
40.93818	16.95349	12.91401	14.80891
76.44149	39.97651	33.79864	36.74166
59.15011	0.486344	0.430929	0.539564
5.880099	14.80113	13.7127	15.61511
79.60234	189.3818	188.346	194.9451
9.337031	20.76528	19.58145	21.54437
70.48868	25.61684	20.76135	26.26903
14.20454	16.19478	15.66389	14.53826
0.900106	3.176731	3.95788	3.591316
1.204912	4.749425	6.198805	6.614505
71.674	201.7923	201.8654	212.6717
8.115224	52.74623	52.08593	52.276
32.24754	11.49902	10.90304	11.61736
4.89728	58.85793	57.02817	61.24511
77.00065	48.13119	49.27224	49.36716
8.815489	3.6546	2.256092	2.898762
14.46635	29.62816	31.88139	33.30145
4.937855	6.722184	7.890186	7.786616
57.67545	41.54172	40.5803	43.56229
6.374629	18.80028	16.677	18.22055
0.107889	1.241081	1.636338	2.298912
3.751235	0.498844	0.558907	0.3264
28.50417	106.2835	103.0521	106.1907
2.723393	1.133401	1.097062	0.909149
30.54231	5.329717	5.470252	4.396511
147.0473	49.43899	53.38581	51.15273
0.291175	0.771616	0.92843	1.194098
0.179068	0.565201	0.579082	0.464891
0.149552	0.395138	0.244088	0.230214
7.284588	5.52446	4.801547	5.762577
4.719987	18.21728	17.41639	17.74519
5.515755	3.672535	2.171351	3.329218
3.565458	5.292017	4.421416	5.347806
4.742112	7.048344	7.401566	6.908559
0.542279	0.234664	0.424441	0.419305

0.457208	1.341417	1.048962	1.33456
1.94925	3.381169	2.622049	3.201545
41.65885	16.6903	17.96972	17.43191
17.76904	34.01901	32.79876	36.40222
6.777685	3.736004	3.293191	3.13343
3.424062	1.233396	1.723965	0.957505
21.22545	45.58703	44.72414	46.66075
36.88405	103.7179	106.851	111.6644
0.666142	2.488214	3.171772	2.558529
1.362646	3.117952	2.763148	3.663178
11.50853	5.345493	7.627283	8.485599
126.3272	37.29162	35.19428	30.21237
2.061167	1.35744	1.75578	1.432482
11.16444	2.338014	2.861898	2.99382
2.68414	1.159048	1.566559	1.209833
1.646633	8.002636	6.165643	6.078959
0.941201	3.654094	2.360357	4.080932
0.784955	1.883536	1.663227	2.143312
3.254339	5.265058	5.430583	5.106559
21.13875	7.607435	6.261798	7.60824
8.860354	3.164256	3.570285	3.303585
38.48754	13.55353	12.9867	14.07057
2.641295	7.211353	6.817669	8.791194
4.900458	15.22271	13.12304	15.08213
0.401668	3.690219	2.668671	2.668123
0.288031	6.245195	2.831633	4.510886
46.51253	15.12311	22.10409	17.96861
6.234115	13.39504	13.71398	14.91283
5.05947	4.302532	3.208242	4.060141
8.055085	11.33171	10.77578	12.8987
2.150936	6.895472	7.988217	6.998241
14.79899	3.29802	2.612571	3.16592
0.274053	1.71199	1.040259	1.97683
0.816718	2.911757	2.34758	2.705482
15.29335	5.223855	4.188752	5.256581
4.242082	3.691737	2.815136	3.386863
11.79505	18.24712	18.98497	20.43808
2.420741	7.36585	10.02235	9.596698
13.15106	2.542005	2.478987	1.821141
37.65451	242.7457	246.8198	254.9251
2.86563	0.805634	1.208351	1.024766
4.926388	7.436012	7.456687	7.69841
0.173693	7.925873	8.628854	8.013485
2.520047	0.872615	0.857609	1.107697
4.75883	1.594156	2.036639	2.308436
76.9364	69.23298	71.47544	70.51608
22.29427	11.57112	10.54599	10.90992
0.573322	2.44617	2.199294	1.561848
8.460212	3.214805	2.93571	2.470746
2.361275	4.588486	4.909985	4.352138
1.331511	1.467631	2.219402	1.497968

227.4186	76.35814	69.57352	70.37421
16.14627	2.087416	1.541704	1.907205
221.1794	51.23333	48.53464	54.14102
2.584969	6.504754	6.22607	6.620285
73.00292	26.94901	24.96406	27.59766
1.205465	2.320738	3.499185	4.04667
7.273934	3.244175	2.839705	4.242362
0.849209	1.412338	1.428458	1.952704
4.393598	6.828351	5.551855	7.070216
74.68525	45.6129	41.76431	43.4791
444.5244	147.7646	148.0154	149.3554
4.984904	9.164614	7.721348	10.87892
12.29043	6.628233	5.739552	6.834376
5.839057	1.393118	3.608392	2.513443
3.627968	46.10131	46.97468	48.46747
1.22072	2.579682	2.027935	2.668659
132.2064	15.62957	12.70841	12.25233
1.817132	5.118814	6.774172	5.898189
0.782854	3.241905	3.307159	3.026023
0.775614	2.161557	2.382168	2.37547
28.8707	200.7715	200.3358	209.0565
6.030025	0.219284	0.783759	0.341663
6.417287	2.537955	2.746981	2.866135
0.317384	5.240005	3.258785	4.035403
0	0.766323	1.338167	1.410192
138.1617	13.05851	12.91034	14.58472
0.600285	4.878409	3.56428	3.498096
4.069191	15.83501	17.2325	17.21635
3.665897	9.405768	8.197807	10.37064
1.973537	5.683434	4.491327	4.835811
1.795124	5.048733	5.15812	4.959234
14.20145	40.54741	41.34956	43.86739
0.95713	3.891501	2.855117	2.929652
3.000787	32.13318	28.62359	32.14752
18.38843	6.799289	7.356618	7.340041
33.35766	8.753965	9.80981	10.18703
0.13522	0.574971	0.853935	0.774636
23.17469	4.745023	5.286127	5.341063
0	0.072914	0.253867	0.076596
7.07969	3.586578	2.968146	2.849702
1.864672	0.515751	1.085057	0.660545
3.369301	4.709977	3.896215	4.585823
0	0.362608	0.35673	0.384007
1.997502	5.623459	5.73101	5.999165
30.23089	19.90672	21.54216	21.96419
4.310636	10.26144	11.18701	11.13946
2.508203	1.147446	1.026805	0.754153
11.29878	76.55933	79.00321	77.46777
1.475337	0.927185	0.54156	0.853794
0.234288	0.541887	0.946909	0.656946
0.663208	20.38365	19.98084	22.92601

16.95866	203.038	195.2608	207.2903
14.63146	45.16467	44.00683	47.81493
231.4526	111.8138	115.2578	119.7127
11.27575	25.63183	26.22314	29.24153
2.238635	7.748702	8.826233	9.37814
11.71964	31.23233	32.98415	31.08476
5.180526	165.2867	158.4116	160.592
4.454723	4.253765	5.205672	4.971595
4.437497	1.840735	2.299922	2.973283
39.78074	2.536667	3.004648	2.856773
26.36772	11.2533	8.755755	8.71544
53.51136	14.33954	15.15244	17.18174
3.746399	6.916233	6.874241	6.034606
0.441527	1.444696	1.435113	1.838646
265.6635	920.2917	909.185	943.2886
21.2977	3.78428	3.260742	3.670969
49.16952	25.05724	21.91392	24.81572
1.258889	2.612897	3.496461	3.250182
0.584457	0.62859	0.402761	0.68023
5.64297	2.118321	1.59192	1.887991
19.34001	10.51298	8.46032	9.192998
2.559368	22.54614	22.85682	24.45562
2.821201	2.324158	1.86575	2.445914
6.440548	22.00388	20.93812	22.49943
5.312913	2.169507	2.303044	1.75738
53.93969	17.99228	14.8662	15.5343
6.756642	4.073064	2.692865	3.598022
4.54791	14.53592	13.01729	17.32523
2.142275	7.581631	8.109169	9.069584
1.498013	1.388976	2.649204	3.217954
9.998071	56.25211	54.84228	58.33621
5.298946	25.43329	23.69374	24.59922
4.742649	12.17566	11.53967	12.52225
69.63745	99.48695	100.5768	103.8817
20.97472	5.012502	4.444043	4.456186
9.519079	4.14828	4.307198	5.792317
9.331297	15.88595	14.26562	15.80642
1.944803	39.66221	36.34633	37.86258
99.48996	291.6159	280.6993	306.3492
29.12318	4.766922	5.645994	6.035816
7.795591	16.30748	16.64401	18.06455
0.666737	0.99803	1.597002	1.418896
2.760405	0.913337	0.993395	0.938564
2.619878	2.848973	1.821411	2.003482
1.144941	1.229591	0.888785	0.626608
13.33845	17.93339	19.83697	19.90164
1.374089	1.154696	1.640494	2.288096
2.567073	5.121211	5.450099	5.457484
42.83757	11.81764	10.46866	11.79382
2.41451	0.824845	0.974498	0.976049
15.33	28.88592	29.8155	28.17618

3.709295	10.9987	14.47143	12.62857
1.532443	4.041623	3.41437	4.352383
0.336177	7.481728	7.266296	8.556834
11.3434	5.890682	6.129881	5.306252
1.414482	5.122406	4.016752	5.797853
4.654804	1.351897	1.953307	1.815611
1.06406	3.432279	2.123661	2.385242
19.35363	4.037608	4.290885	5.245758
40.90617	35.03624	35.09966	38.18467
3.368401	2.977244	3.306128	3.394027
0.6721	0.157056	0.392106	0.496461
1.585445	2.417648	2.41645	2.232613
45.88252	199.9067	174.8732	203.2743
46.62358	20.63232	18.04839	18.89262
172.9575	39.24416	42.32721	42.55753
6.433319	5.909597	6.765437	8.136528
6.615505	1.188013	1.886263	1.479628
0.930763	1.327778	1.255872	0.721355
4.993545	8.120976	8.184233	10.56366
17.56103	65.8255	60.52974	62.80403
193.4759	54.70187	57.98728	54.25446
0.478948	1.362966	1.146029	1.168326
158.4284	555.5975	497.8747	549.7979
5.22861	3.979701	4.250134	3.248594
3.195358	10.42389	9.290655	9.091188
0.256693	1.548597	1.475533	0.731559
0.393553	0.092753	0.19213	0.254281
0.493042	0.325003	0.177644	0.402499
7.016278	4.925191	5.124605	6.256677
29.49544	16.10018	13.13388	15.9009
2.488438	6.852618	7.977577	7.863277
10.12035	10.17318	8.803704	8.939631
9.379148	5.381601	4.349117	4.676024
9.981738	16.21726	17.57004	19.60873
6.097029	9.015519	11.98808	13.62372
1.433049	2.403253	3.126736	3.208636
1.76085	0.488994	0.425281	0.631977
1.069276	1.928271	2.112957	1.787035
1.584011	5.77016	5.31727	5.686836
3.102453	10.05717	8.849793	8.731139
96.49571	25.59174	28.23257	29.62915
3.70903	18.30891	22.09822	21.36655
2.695629	7.056664	7.125111	7.084583
819.3384	115.8129	114.1112	120.0732
8.529651	4.103123	3.830485	4.223856
27.76119	7.145648	6.926315	5.91035
11.56816	8.380286	9.660819	10.1748
22.14934	9.09637	8.170252	8.523757
11.06793	3.195404	2.625284	2.696199
1.122594	2.929454	2.253037	2.339711
0.676511	0.368378	0.389756	0.454227

65.96082	65.62557	64.25221	59.41756
8.844196	2.635604	3.174237	2.772106
1.438161	0.642291	0.670354	0.68324
84.83842	21.86489	19.51808	21.53769
23.12459	14.11428	14.22458	19.71286
0.823053	1.558897	2.131617	1.923826
8.168802	1.707369	2.186774	3.09962
0.584139	2.46602	1.429151	1.273298
1.274342	0.298263	0.489591	0.239684
1.83015	3.963638	5.567667	3.327581
2.308289	1.524865	2.077911	2.450319
112.4186	26.71758	27.26465	30.82814
9.189037	5.236047	4.43062	5.060287
3.453354	2.167616	2.566703	2.074818
33.22345	16.6551	16.46378	15.83875
0.576358	3.41848	3.486303	3.67156
9.868913	33.64322	34.33567	36.00916
0.851598	1.988771	1.722168	1.801625
5.688197	21.32191	19.73204	23.43605
0.234803	5.220841	4.101271	4.698743
0.503423	0.047627	0.066433	0
0.253594	0.665603	0.577485	0.934328
63.13257	28.37261	30.30918	34.45292
6.008043	13.35246	12.81376	13.36366
18.15735	9.177945	7.122251	8.410625
2.176825	0.569032	0.272941	0.375622
3.565526	13.04414	12.21127	14.53383
3.413237	13.41354	13.68598	15.87858
2.958411	11.79649	11.63123	11.47834
30.00398	64.77682	64.54261	61.72664
10.26314	4.266473	3.790659	3.272978
50.02234	30.108	29.37703	34.39018
3.123789	7.457729	7.94942	7.96705
2.909734	1.006361	0.324616	0.581433
10.42713	4.868265	5.120438	5.512866
324.8752	105.5148	105.1975	112.1747
0.061462	3.389863	2.708861	2.632746
8.429172	0.784694	1.420601	1.457417
0.668991	1.043981	1.450437	1.677669
28.92917	101.1381	97.19361	104.4268
0.136687	0.743256	0.738351	0.169333
6.872761	4.22052	2.665717	3.449045
3.168331	4.448405	4.757286	4.31242
0.318487	0.749614	0.824545	0.635921
11.29818	1.001821	1.019224	0.981928
4.884418	3.731513	3.164801	3.598935
1.47881	7.053939	8.236698	6.926327
1.979486	6.904047	6.568747	6.374888
13.08831	15.32821	14.48683	15.847
4.060084	14.76141	13.49418	13.77295
8.76693	13.89241	13.39761	12.60086

27.52045	9.771805	9.692569	10.29411
5.295727	2.124278	1.663983	1.10383
2.769763	7.389846	5.802481	5.840685
62.04864	23.39268	21.56273	22.38346
0.995433	0.475543	0.381836	0.549508
1220.187	248.9345	250.7386	258.9398
4.087883	8.8813	6.879772	7.32437
21.37681	128.6069	112.655	124.2401
4.145287	10.47224	8.668634	9.168728
97.96352	33.39457	32.9101	34.15958
80.51109	11.87637	10.20416	11.50704
1.7031	9.270922	9.762376	8.192176
0.174812	2.101998	2.420559	2.246128
2.211091	4.565818	3.764787	4.843758
6.021348	12.00485	12.49491	13.77944
0.764958	0.239042	0.268432	0.21769
114.4895	53.24689	52.15982	55.75996
42.41732	14.11807	12.04155	14.75796
1080.405	340.3398	326.4211	356.8212
6.926637	3.535322	3.113626	3.673349
9.837059	11.21017	13.80937	12.02942
14.19115	4.363793	5.361424	4.1436
5.636598	3.918405	4.43276	4.597945
19.1372	3.57135	3.025031	2.755081
44.2492	23.10074	19.90925	24.05394
1.576895	0.667344	0.429525	0.771728
2.079902	1.040042	1.262282	1.475433
22.97368	7.628339	8.690484	7.707592
0.277022	1.77354	2.382983	2.058444
0.033661	1.65369	2.037847	1.906795
2.180657	4.421063	4.002093	4.432563
1.596047	0.396529	0.555339	0.226321
0.264472	0.430466	0.707985	0.786472
1.002687	6.170142	5.627173	6.26343
2.421141	2.472389	2.60741	2.505
4.788408	12.90351	14.50535	14.16096
1.609962	6.879835	5.480436	6.328506
0.525513	0.344041	0.420273	0.354477
0.40955	4.60893	4.266037	4.741193
341.7856	116.0322	120.3657	127.4554
3.72876	9.328045	7.604854	7.334565
0.758252	0.254499	0.31201	0.132444
1.536397	7.99648	7.902876	9.950667
5.115411	1.231084	2.7077	2.306663
0.749408	3.425133	4.861546	3.657829
3.375048	4.87655	5.533159	7.054489
0.313575	0.086284	0.281737	0.175801
5.106055	2.773231	2.124414	2.780973
0	0.666376	0.595955	0.536141
0.56273	1.096467	1.985049	2.514252
3.27309	53.92756	49.00981	54.69756

4.85967	13.01548	14.21181	12.77107
65.12085	30.60063	32.79516	31.58065
1.681877	3.760167	4.116932	3.681252
6.314217	1.865342	1.229017	0.79585
2.430736	1.110267	0.946909	1.150186
14.78983	6.583755	7.080214	6.502223
0.460976	0.695902	0.523371	0.747709
17.53023	37.88657	38.25826	39.88646
0.973156	1.571489	2.576726	2.609004
0.873622	0.374102	0.915253	0.308804
13.24442	4.425709	4.459785	4.962665
22.58389	11.27524	11.96101	12.5492
2.6752	0.71132	1.012359	1.111317
32.8815	136.7026	123.0137	137.4222
9.18612	2.580372	3.220208	3.802747
9.941461	27.01659	28.40542	30.41613
3.646391	8.22008	7.50891	6.427408
0.385483	2.32708	3.050128	2.555964
0.384166	0.646766	1.174452	1.07817
152.497	182.373	177.6166	180.3611
3.828244	3.452259	3.224656	3.232366
0.862005	2.401003	1.899986	2.132361
17.73467	18.25747	18.41898	17.38345
13.82015	32.52939	44.38249	38.67941
4.958392	1.813637	2.740279	2.755336
4.139892	1.453757	0.969253	1.142215
6.821801	7.362579	9.043328	10.34692
3.978983	14.3803	14.36495	13.20197
2.162498	8.969434	10.51499	10.34727
0.933438	2.840993	2.374386	4.143236
4.814888	14.43397	14.09052	16.68608
43.34248	18.43577	18.6892	20.39817
4.590596	21.32943	20.51148	24.00666
8.185176	23.69164	23.52374	25.50486
1.596062	0.518555	0.700712	1.079967
0.834452	0.167311	0.191519	0.171227
21.10035	11.08321	9.838041	11.79016
36.31915	18.73771	19.17656	16.88604
0.966471	6.464575	5.423831	6.13435
2.463471	6.115697	4.833382	4.450101
0.727386	5.550169	5.014257	5.835059
1.67198	3.193483	3.508455	3.628215
3.018098	6.778332	6.896307	6.54611
4.403732	1.848547	2.559648	1.913643
5.545463	4.193382	3.523156	5.261662
9.834016	54.05112	60.94972	56.79148
98.03942	27.04377	24.28856	28.065
0.505519	0.715738	0.808263	0.737194
0.193235	0.976285	1.550112	1.592431
0	0.109102	0.057193	0.071758
176.5809	48.29168	53.10117	46.10419

12.10832	33.12095	30.63413	30.05374
36.2818	13.15025	12.97766	13.80086
3.30126	37.62563	37.73214	38.38224
8.515306	3.874376	3.9359	4.231288
59.78436	24.98284	23.41813	26.74805
16.70864	5.533082	4.995986	5.278
3.946005	39.20514	41.50757	40.52279
3.499699	10.27822	10.2174	9.911214
78.60563	6.945172	7.340403	6.529594
25.38094	93.28236	97.02358	93.84137
1.381461	1.011906	1.20256	1.24294
1.002552	2.785458	1.670492	3.203385
1.983589	4.704625	4.569535	5.22555
6.971465	9.057819	8.525293	9.457133
0.508739	7.831133	5.67038	4.936121
7.079826	14.57018	14.23857	14.32191
8.761589	9.44025	9.643016	9.614193
0.034443	11.6333	14.72815	13.55363
29.14885	14.00844	12.53447	11.25496
1.344482	4.933948	4.147565	4.260428
1.134724	3.881542	3.014297	2.063533
0.913691	0.136018	0.159872	0.340154
3.243836	1.679673	2.062675	1.954852
20.78515	6.584344	8.686122	7.73469
0.959824	1.551941	1.505087	2.530678
7.003679	28.71256	27.29788	30.75733
1.612098	5.698819	4.610746	4.719166
0.229582	2.330481	1.839928	2.403399
43.43822	17.65774	13.34296	17.8288
37.03109	13.18712	13.72177	14.05622
5.674902	15.58055	13.90697	12.60074
12.02661	10.28873	9.433803	9.773323
1.01466	0.528329	1.354206	1.468079
1.741123	11.61933	10.65773	11.40677
0.610284	1.218527	1.132745	1.065845
16.08199	22.60405	24.72715	24.23419
14.65033	5.97729	6.109994	6.647549
15.0465	7.835661	7.605604	9.165428
18.71448	3.826836	3.712838	5.87061
14.4844	51.73545	48.17151	52.38958
2.068174	5.996123	6.121283	5.719938
2.864642	7.319605	7.633169	10.67327
1.257667	3.827153	3.144175	3.520709
0.331965	1.370413	1.713675	1.220386
0.911201	0.397151	0.379893	0.489229
1.565698	1.151679	0.69876	1.543364
23.04936	8.017686	6.905801	6.718419
0.791864	1.619874	1.480178	1.398231
54.92055	25.00024	23.61291	28.35112
23.28511	7.689596	5.925555	6.141644
21.69324	75.52756	74.86752	81.75523

4.764144	2.587438	2.217793	1.952885
10.93707	4.257132	4.744066	3.411758
0.771315	1.175232	0.61957	1.272686
13.89792	4.826728	5.468269	5.116791
0.698208	2.457637	2.186655	2.825715
2.584219	7.34836	5.708977	7.137712
8.645755	6.525659	6.528493	6.332323
10.96522	2.679987	2.257061	2.646628
18.7328	9.373041	6.538913	9.386749
4.022095	4.587115	5.013669	4.182682
7.940416	0.56682	0.546956	0.439711
181.0472	94.68471	96.66335	98.97868
2.091882	1.222111	0.826465	1.209788
1.416289	0.715829	1.113613	1.168975
0.451886	4.922188	3.173	4.006268
5.837336	14.03128	10.96699	11.62881
23.30438	19.80176	18.94457	19.28027
7.841095	3.633136	3.941471	4.406845
1.444461	0.892755	0.714671	0.503257
48.47514	21.4245	20.93436	20.36273
3.283768	1.090523	1.047888	1.669201
2.109219	0.332368	0.849848	0.114935
21.57241	8.485733	8.207083	8.677625
0.557455	1.518803	1.275547	1.188962
6.856124	12.91107	11.62833	13.26597
4.158247	2.883312	3.492825	3.839578
5.757049	14.56108	13.82621	16.05219
117.31	48.1662	48.78624	47.20663
1.973404	5.703465	6.439453	6.756149
7.561517	4.250717	4.563408	3.738973
7.853976	3.992046	4.768643	4.501974
1.563342	4.786727	5.076459	3.946423
24.9908	28.15185	26.94061	28.6001
3.310552	5.325197	4.307735	5.184557
583.5589	223.9153	196.7475	216.6025
9.264835	4.254653	3.982654	4.790746
33.82226	14.96253	13.25587	15.20747
15.19709	0.042288	0.422753	0.314351
47.5886	16.75638	16.05187	16.0675
0.388694	1.448214	1.141129	1.479603
51.99297	112.0578	105.4801	115.0749
95.88079	181.7047	190.8694	193.1065
10.12644	6.970889	6.13257	7.581122
114.0567	39.26122	41.37957	40.44577
295.6941	106.9893	109.4406	108.4392
2.40128	13.00953	12.37802	12.5223
273.0631	92.11208	92.1297	92.71693
1.780878	3.04166	3.227907	2.89337
4.854425	1.946249	2.049773	2.371449
1.510002	7.908363	8.915386	9.998793
4.035422	19.66491	18.08634	18.99366

2.671526	16.36194	17.51377	17.98756
375.3279	195.8951	188.4249	201.78
6.554046	2.012957	2.125954	3.527634
0.393718	1.552708	1.403392	1.091705
7.757299	5.963609	4.995938	6.570932
0.138213	4.07979	3.763078	3.360349
0.964972	4.410366	3.972374	4.079286
6.996531	2.639682	2.889367	3.459682
3.729553	2.884291	2.403897	2.594203
35.92773	11.60231	14.33065	10.76438
11.19256	27.36484	30.27968	28.33104
10.75437	4.653938	5.572924	5.227979
8.454734	3.913065	4.90752	3.976859
15.53964	7.951577	8.781291	8.714651
1.506357	5.787891	4.065287	4.617839
2.528746	59.50414	64.20535	65.07078
1.383665	2.06103	1.723363	1.926452
1.405521	2.266211	2.706489	2.305232
48.65593	47.33741	51.54499	52.06287
2.441785	21.18252	22.14692	25.43668
0.249841	1.011547	1.027777	1.431392
33.70012	23.90447	22.68745	25.63577
10.77089	4.736041	5.212306	5.221683
5.403462	2.014318	3.302781	1.701076
0.321369	0.522726	0.322222	0.366445
1.716261	0.627362	0.638891	1.432647
0.351321	0.217506	0.372235	0.504137
56.63817	39.56336	35.71599	39.58249
52.79696	20.2521	18.16061	18.06676
9.737442	43.31897	42.53817	44.50405
7.984094	31.40962	31.26203	32.02795
0.493355	0.51415	0.283704	0.492421
49.73388	24.56022	25.53802	26.98783
16.19576	31.65619	29.80731	29.99966
2.659493	15.24941	15.25864	15.07967
52.25311	22.85512	21.78938	23.08862
53.86432	146.0186	146.9523	149.6275
96.24537	41.11195	39.98635	42.44062
3.239655	1.455236	1.658378	1.869006
1.475068	1.381638	1.405846	1.346766
0.287741	21.75541	22.1973	17.48724
5.551006	16.47275	15.29528	18.8612
8.307458	22.14006	23.98195	24.2203
24.86075	9.848325	9.740024	11.07319
0.396066	1.29128	1.214777	1.060489
499.8324	1069.412	1074.399	1088.707
1.43774	15.15027	14.23326	15.23587
10.89655	2.238507	2.27415	2.49307
52.2675	25.69344	26.02003	25.28416
0.970613	3.056493	3.929467	3.830675
18.26979	5.929162	5.512455	5.812226

12.08616	27.81501	26.17627	27.45422
6.264623	3.02452	1.98474	2.010612
5.778337	17.11407	15.20891	16.02038
23.64357	5.168664	5.633335	6.398302
4.529387	3.513931	3.223418	3.256809
55.77047	28.29792	28.78877	28.68121
1.947498	15.53583	16.98268	16.68272
7.68243	15.7315	16.00457	16.4842
6.480068	4.262267	2.425676	1.790802
97.70853	13.65467	13.3325	14.05092
11.85418	11.03833	10.70624	13.46013
4.310537	2.390135	2.227514	2.383416
256.0491	89.90107	92.81094	96.82234
14.89734	5.926317	5.011626	4.750309
6.153918	3.562316	4.558434	4.194576
27.53063	14.33792	14.73196	14.92363
23.66636	7.865385	8.844673	7.465332
0.92321	10.47948	9.55474	11.46379
101.2643	39.88149	40.70934	38.35157
0.366891	2.437838	2.153507	2.578261
2.155554	2.675164	1.52757	1.884576
0.75023	12.1791	11.75249	13.64168
5.372056	2.234763	2.540544	2.318505
1.417026	0.300555	0.316099	0.223596
14.40866	3.944897	4.025114	3.732951
136.762	66.94846	69.3747	75.83187
573.6515	95.48549	92.60959	98.37518
4.113881	5.560774	6.093988	5.453179
1.407612	9.521782	10.34886	14.11541
36.70472	94.03698	100.3531	106.6269
3.306348	6.570687	7.187268	8.452337
52.44233	16.22852	14.73187	17.13701
2.417949	9.407375	8.78459	8.961782
5.644587	10.04336	10.4063	12.03813
2.989067	0.81073	1.223686	1.610127
43.42205	89.32485	85.93521	90.90728
0.717721	25.64329	25.39878	28.20055
14.69586	10.82018	12.40171	13.71764
22.19553	45.43049	45.27091	45.36552
131.47	34.71693	34.90543	33.71588
17.33376	52.06036	52.2537	53.93883
3.740816	12.20762	9.518074	12.56169
33.36837	170.0625	161.5865	173.9882
0	0.76963	0.48623	0.994221
2.039438	3.305624	1.863825	2.666305
138.0874	67.91492	70.40563	69.80035
15.24472	6.1466	5.698783	5.094372
3.414357	7.049474	6.836223	8.641054
4.856738	0.629079	0.957782	0.953482
12.22566	7.882356	8.414111	8.944036
5.612789	16.76753	16.33451	15.81234

8.608728	8.256506	6.746634	6.609803
1.695584	6.194437	5.367992	4.51529
10.42318	3.845456	3.55476	3.088526
0.529978	1.407455	1.19089	1.524872
3.9412	2.570346	3.160102	3.763103
1.002076	0.95202	2.361658	3.228253
53.9678	33.88673	33.28499	35.63003
1.307435	0.873606	0.633485	0.461049
7.174046	3.285261	2.912175	3.688118
562.0723	164.2461	159.0871	161.636
3.927194	55.42378	57.12314	54.4639
5.316659	11.73124	12.61515	13.68698
13.60893	1.584889	2.195247	1.763217
2.110842	1.002395	0.87423	0.95392
7.920122	3.60215	3.893822	3.885818
53.02056	22.24981	25.19529	25.93711
2.645967	9.209923	10.49617	8.151383
1.292743	4.144613	4.85796	4.350078
71.00609	37.03574	36.36936	37.73945
13.49438	19.76226	22.5485	21.19855
33.92539	65.25731	63.69144	68.92847
0.393584	2.71259	3.423661	3.377936
26.0804	13.03213	9.986728	13.27659
0.052849	0.191885	0.293825	0.576045
2.337264	1.281646	1.229601	0.942677
1.489575	8.906569	7.923005	8.296313
1.617516	0.34967	0.440613	0.552011
2.260949	0.080721	0.168246	0
172.2994	82.82087	77.57394	83.67691
31.00612	67.82514	64.43472	68.76005
212.1105	100.3323	95.07127	106.4725
57.84342	26.12309	28.05724	25.20039
10.0696	33.41345	34.14046	33.88032
5.981375	15.85358	13.65165	14.46591
2.836632	13.63101	15.11311	13.4141
0.094134	0.476096	0.463819	0.371004
3.829826	9.898834	9.321357	10.3979
4.05474	10.4356	10.59821	12.07074
1.162455	2.394134	2.446994	2.414152
1.812506	2.612682	2.45917	2.299309
0.302622	2.168658	2.215307	2.343251
33.27383	11.38161	10.5306	11.07526
2424.674	741.9664	708.605	771.2434
0.457652	2.173179	2.225824	1.835388
0.553369	1.401715	1.509527	2.025371
2.828819	6.903423	7.325514	7.249478
19.54223	43.9097	46.20153	50.47723
5.792489	5.724061	6.641922	5.849568
5.200447	12.70963	10.09301	11.44415
0.481442	1.070403	0.847297	1.167624
36.5489	15.99749	10.61356	13.31189

2.325672	49.48405	46.14583	53.86309
3.015728	14.05885	13.60784	13.6506
68.39329	23.96923	23.04043	24.42691
10.62072	10.63975	11.43686	11.06355
8.92885	2.931888	3.292173	3.379266
12.27452	12.69277	14.57155	14.36525
19.27214	7.47373	8.284011	8.22074
1.892987	0.431394	0.408488	0.318754
0.896436	8.807967	8.540644	9.905627
24.74835	11.82486	15.73377	12.93486
0.482245	1.517756	2.206291	1.392555
1.675669	15.66578	15.74015	18.03824
0.910467	7.458385	7.879467	7.817338
13.47151	6.218339	5.901314	5.920945
0	0.964146	1.683439	1.142748
3.678797	17.10628	14.53694	19.6143
1.549735	13.86849	14.61469	14.47417
5.32256	65.89421	65.09638	71.56053
1.679901	4.578615	5.358387	4.450618
5.106002	20.41039	21.26496	25.16754
194.1483	82.01235	85.96162	90.54593
3.817612	10.28826	8.90548	11.1887
3.942056	1.162717	1.659284	1.619916
15.05508	9.236285	8.013761	8.669714
803.9895	253.1666	254.6566	265.27
56.87578	27.94524	26.71986	26.14854
2.483274	3.921163	4.471324	4.944018
2.206734	0.566159	0.940608	1.65272
20.6922	19.87777	18.94873	20.76559
121.0591	60.59216	63.25555	61.41787
0.383089	13.77833	13.46639	15.75534
7.397932	12.78172	13.85207	14.35088
1.549205	0.434966	0.585462	0.563038
1.408435	8.934603	9.412992	10.67057
48.45232	179.5798	179.8296	173.1594
2.336249	5.577229	5.729056	6.329389
1.845947	4.43512	4.786323	4.585452
9.717346	4.314538	3.973213	3.638088
7.133012	7.94876	7.624905	7.160582
31.2654	16.79481	16.56093	16.58301
8.014206	16.02001	14.73643	17.63311
0.967646	2.449597	2.248105	2.578469
5.9387	17.86208	16.64364	18.63099
2.124745	1.633652	1.762598	1.939904
82.27973	30.46989	31.19469	31.8024
24.65812	10.49914	11.54174	11.11727
101.4274	65.70699	62.46792	73.63097
37.96927	75.88912	69.02021	70.97086
2.805893	11.40168	12.07265	13.35533
46.60101	19.55565	19.50935	18.07864
27.69862	23.253	23.40851	24.32422

1.791844	3.056053	1.925306	2.807411
3.557225	9.556047	8.984096	10.41661
0.863431	1.521379	1.365809	1.881205
0.999309	4.359725	3.845646	4.142918
0.536843	1.220625	1.064976	0.945539
0.688186	3.334047	3.273486	3.388074
1.721926	2.298446	2.374628	2.499064
6.393471	3.250524	2.857755	3.253005
7.219027	30.8908	32.00642	33.0126
9.501412	5.629553	4.997387	4.251018
6.223032	6.460043	6.302694	7.770421
132.3213	31.71439	31.23731	39.06552
941.7108	123.3199	120.7416	126.2962
22.88672	4.284166	2.946491	3.638382
1.255679	0.57079	0.372286	0.256991
30.2558	16.9118	18.7636	17.63746
19.16297	2.964181	2.726951	2.156235
83.31232	5.455268	5.107226	4.96428
1.714775	5.537937	5.231987	5.311473
212.7778	429.8734	421.014	429.1237
8.932304	17.50149	15.14062	16.89631
28.81312	10.14002	11.18735	9.999938
94.63953	27.15822	25.76576	28.87814
4.577651	0.693216	1.108488	0.647822
6.100417	3.725728	3.094916	3.033643
6.032407	3.502356	2.834862	2.908539
72.5228	46.90036	44.137	45.56774
6.286091	2.849538	2.507444	2.72313
4.758121	2.225984	2.48321	2.641369
0.953841	0.137612	0.379423	0.376718
65.57669	52.39259	47.87314	51.65529
8.702245	3.67529	4.93462	3.928421
1.609534	2.666914	2.909206	2.606769
1.176392	4.831224	4.942908	4.11954
2.62341	3.865151	3.820073	4.311447
0.801824	3.32083	2.332791	3.314306
4.333414	7.174563	7.003941	7.38328
9.41315	15.99728	13.1236	15.57099
26.24041	5.86752	4.46017	6.120336
2.946227	14.98833	13.46307	12.53936
31.11358	0.729163	0.747215	0.974706
0.396818	0.293533	0.291274	0.410952
5.008976	12.73053	11.54735	10.50301
17.88423	58.20505	47.56116	50.96572
0.08135	0.956181	0.991946	1.105127
7.743418	43.0138	43.22027	47.08806
0	0	0	0
5.068453	3.557725	2.837047	3.287756
0.979288	4.645261	5.156014	4.017537
0.633893	0.531735	0.444225	0.68073
36.76519	97.45616	102.8427	103.057

6.954048	18.98764	17.5155	19.86056
1.886834	0.441229	0.478451	0.453396
214.7604	138.1864	126.4835	145.1386
453.5847	760.973	792.0234	811.6923
6.501709	0	0	0
65.91339	23.11604	25.42439	24.11448
1.769033	1.013472	0.963574	0.985734
0.135109	2.128969	2.061749	2.283689
3.216201	10.63923	10.74621	12.08715
25.81847	8.225534	8.35046	7.705577
0.550488	1.25324	1.069488	1.118867
73.51553	31.62509	28.48275	30.01788
850.0037	109.4694	72.67092	124.2222
0.680711	0.386641	0.157411	0.268035
2.968919	8.16695	9.314487	6.557271
1.038662	3.354033	4.413566	2.964607
7.433295	21.93192	19.16386	19.72253
25.57589	14.77424	13.42725	13.33733
0.999916	0.317644	0.449093	0.340217
9.760379	5.193455	5.102655	6.037628
5.836101	3.76039	2.725173	2.95549
17.37822	7.878414	8.164484	8.553732
60.93441	26.71804	25.3446	24.4092
0.647837	0.839292	0.960709	0.832962
3.110449	14.77536	11.68561	13.10857
5.080909	3.241105	2.455577	4.124941
10.95869	6.331144	4.933361	6.233872
1.992585	2.858176	2.058473	2.049383
2.441642	14.92209	14.661	15.02934
21.47852	49.52118	44.96777	50.18985
4.559486	4.306829	3.891969	5.295192
1.899009	2.578805	3.432731	3.786864
19.62201	9.056885	9.158982	10.34293
1.100279	2.169122	3.046816	2.020787
3.215551	8.711869	8.39829	8.611846
23.06229	11.431	9.68161	11.38212
4.44741	6.998239	6.768463	7.357855
14.57762	28.00819	22.6918	26.11595
9.780352	24.0309	22.55969	21.64529
1.018122	4.216341	3.429958	4.474535
0.483798	0.289153	0.160266	0.091848
85.30624	1.940796	1.475408	2.077944
1.476753	3.411659	2.458455	3.682849
2.9887	14.57976	12.01235	15.14219
41.74834	17.94927	16.14969	18.01149
35.74328	4.368825	4.778349	4.597553
50.05808	156.1335	151.5185	160.7916
0	0.888717	0.83318	0.501044
8.488419	16.5372	17.7604	19.94242
6.452951	8.446728	7.112707	8.672698
0.652793	1.552506	1.289159	1.752526

61.33309	32.86441	30.22575	33.15156
4.809469	63.60955	60.66986	65.92436
0.337407	1.240895	1.996496	1.761318
10.97961	7.539648	9.593872	8.604138
29.74341	15.42127	13.91422	14.4132
18.34461	8.030158	7.101958	7.795614
14.30902	36.33274	35.92698	40.28996
1.681459	1.024666	0.882421	1.20623
654.3119	75.41229	74.3179	75.34762
14.17499	21.54376	20.05674	22.23083
4.708193	30.98239	31.10866	31.86673
2.968369	3.689093	4.354896	5.043201
48.47239	209.8725	212.6274	212.918
3.746348	35.11995	41.71856	38.29871
2.307934	14.11384	13.11457	14.84357
22.33725	11.39286	11.46256	11.73869
50.74911	8.066572	7.482541	7.965854
4.552874	2.165736	2.235123	1.988647
4.444444	2.256721	1.998833	2.194599
0.260611	0.895667	1.548907	1.381805
11.1103	5.471991	5.397037	5.272222
46.35203	21.56403	22.0497	24.97032
23.89066	12.74609	11.20451	9.295013
21.6935	8.303851	7.350082	7.7463
0.079192	4.441315	4.696886	6.057129
11.57171	9.169479	9.940243	8.409565
12.12091	3.461116	3.822314	3.540676
4.161861	2.082539	1.951957	2.899053
6.525462	1.570583	1.520178	1.745597
1.647202	1.022991	0.550101	0.720728
8.047111	17.43297	18.38525	17.34711
0.39527	2.035951	2.679235	2.260336
0.84151	3.27546	2.706878	3.080364
7.058183	2.559688	2.958871	2.817914
2.712147	1.093547	0.494558	1.178002
0.008503	0.062132	0	0.20203
6.577935	13.55906	12.06142	13.78937
3.401163	48.05695	49.32085	52.40494
17.50442	3.029724	3.076045	3.34767
374.2349	202.654	182.4326	201.959
17.2762	43.77005	41.28025	42.89644
0.389338	1.233273	0.990108	1.097269
29.50143	2.147104	2.450891	2.50898
15.7746	6.705878	5.141044	4.893168
9.651011	5.60784	8.326436	6.222075
5.500814	43.789	44.95993	44.03321
161.3411	68.85049	77.46073	72.44492
2.519233	0.33996	0.322223	0.346775
3.486501	7.154138	6.454885	7.360736
0.132172	0.99064	0.974002	1.410433
17.91485	4.442965	6.427504	7.276668

1.454912	4.59993	4.661599	3.961731
2.721268	8.650345	5.997664	8.299714
544.4792	235.7776	225.2118	240.0022
39.91005	13.08808	13.85069	12.96204
2.25792	1.928716	1.927517	1.587057
3.516362	1.651484	1.515636	1.371652
108.7366	27.46323	27.21769	27.24591
60.09414	19.28135	16.88855	18.39669
18.06785	57.73908	57.93667	62.07308
0	0.658763	0.574129	0.580192
2.523345	6.820861	5.554775	6.5539
0.436884	2.779652	2.515436	3.044659
70.97031	38.49022	38.50937	39.67768
27.20894	13.07452	13.50755	14.61512
0.508062	3.341925	3.78032	4.475085
2.315134	1.295006	1.193243	1.28848
3.317116	1.329374	1.847197	1.260946
0.12082	0.609561	0.347329	0.52201
499.0515	58.05109	61.76007	57.90878
3.614925	5.000875	4.941044	4.968347
18.77598	3.743638	4.46286	4.222653
1.633733	1.188393	0.632443	0.439167
0.972138	8.913915	7.462169	6.8967
0.129231	5.342842	3.715788	4.257663
0.788069	4.398293	4.271569	4.173334
37.31606	148.481	141.249	150.973
25.35952	65.46329	60.68055	63.10912
3.720984	18.95499	16.0659	17.47955
0.908407	0.589594	0.464295	0.496369
17.71825	49.90787	50.18013	50.7319
8.586409	27.97011	25.78411	26.46534
5.127305	9.361431	9.06483	9.553451
4.912764	7.369471	9.982903	8.371685
64.31099	31.5945	33.97919	30.2056
22.21852	4.119298	4.807232	4.704273
9.967946	25.72576	22.05433	24.4749
24.61178	12.6911	13.23394	11.49772
18.52029	38.82433	39.6257	43.91738
0.231416	0.855072	0.792583	0.931119
9.745341	4.566096	4.415206	5.774102
23.12389	9.769985	10.33983	10.34225
6.872536	11.30195	11.28899	12.5448
0.941004	3.956282	3.265583	3.271148
68.12338	32.71167	30.66006	32.70177
1.249414	13.68853	13.10878	12.09093
3.385694	8.894423	8.595452	9.522358
11.65166	37.27929	36.86936	39.35912
4.795583	9.574448	3.96164	5.956246
251.6196	67.98903	68.3663	72.28307
42.16742	578.3973	552.4818	518.9319
0	0	0.114392	0.143588

0.619306	0.430062	0.764598	0.492396
15.33066	3.521266	3.714427	3.403226
4.08902	1.234339	1.270875	1.223701
0.095674	5.072133	2.652275	3.81935
8.684641	19.01786	18.78488	20.8185
0.370545	1.501706	1.686459	1.808157
17.32621	19.09733	15.94621	16.41533
1.077652	2.890174	2.71614	2.350438
0.554585	2.14466	2.186669	1.631873
0.332903	0.094377	0.219704	0.175869
15.22503	9.113266	9.117565	9.374323
3.47212	8.10764	8.627784	8.682106
15.68312	1.545428	1.319792	1.764068
410.3361	415.8286	402.0161	436.0797
0.903986	1.461689	1.169093	0.919072
3.044693	6.159667	7.917567	7.506951
21.21634	83.8185	81.2611	84.64283
4.136511	1.939368	2.117647	2.906909
20.83742	10.84677	10.71899	10.45643
7.842731	3.731347	4.285477	4.800958
0.290222	0.1094	0.115607	0.207814
1.353625	0.22029	0.435722	0.498648
19.99356	6.795785	6.878003	7.632634
0.490946	0.273546	0.064569	0.11678
6.109107	21.91309	18.76252	19.52035
2.740824	7.663778	7.837693	8.387952
127.294	35.93086	34.92773	37.36017
0.770396	0.105587	0.228382	0.177812
11.45889	28.29401	32.57165	32.42409
13.66475	0.815437	0.952085	0.639623
92.04125	28.42643	25.60529	27.22775
4.39028	2.738484	1.84784	2.858192
19.87114	7.032279	9.312664	8.405348
14.68808	5.728702	6.301445	7.236695
3.614379	11.55248	11.92196	11.34261
0.395063	4.196846	3.351715	3.175361
9.457798	3.947626	3.698151	3.622398
35.75819	13.37954	13.27237	14.27824
3.02316	9.593809	8.415329	9.572667
0.661748	3.512929	4.570096	2.969261
58.10664	27.77022	26.94217	28.91011
25.35631	31.8208	33.07571	34.00477
0.241325	2.339543	1.960953	2.097449
16.33254	5.560106	5.597225	5.899675
7.039175	15.31984	12.15312	13.95224
5.274353	10.23122	8.970929	10.40681
5.584092	6.089511	5.796507	6.186781
0.980645	2.27321	1.914224	2.2965
21.71171	11.8074	12.22534	10.47037
0.939943	1.178257	1.206774	1.480375
14.30804	7.29134	6.427717	7.06111

0.219188	1.434272	2.111612	2.486482
0	4.230701	3.128832	2.911648
0	3.666983	6.410019	4.188507
16.88795	16.60509	16.40187	15.72916
4.505993	7.858421	8.123116	8.648404
19.90847	38.6132	36.20759	41.04707
4.671838	3.821846	2.180355	2.092258
90.05009	40.79002	39.88596	41.22453
34.23147	3.657716	3.762612	4.447027
1.791525	2.269858	2.614849	1.77488
0.539319	135.4255	144.7906	142.1037
1.746872	5.805273	5.463999	5.479995
2.145422	6.887777	8.184004	5.169772
0.290255	10.31706	7.600717	9.801726
20.80412	17.5774	18.71152	20.76678
89.12609	43.46719	44.15984	46.10582
8.407217	21.9538	19.17682	21.2885
101.7174	37.97182	41.23356	37.54729
0.109537	0.815794	0.611246	0.573735
63.24044	37.36405	30.318	31.29378
17.66804	5.854693	5.377026	5.244802
1.814127	0.556369	0.613105	0.916191
17.90618	7.057519	7.207483	7.49456
16.44398	12.35285	11.85014	13.66391
1.492047	3.767729	3.77903	4.686216
270.1918	126.4752	131.5491	133.4562
36.61865	16.72014	17.27761	17.0124
72.60276	180.2698	167.0038	182.6866
22.45618	16.59391	14.49328	13.48197
0.424741	1.573517	1.658707	2.148916
32.12782	18.04043	18.28358	16.97735
4.049178	2.484835	2.22608	2.324499
8.786808	30.14624	28.68483	30.40276
9.72447	19.23054	21.14883	21.90584
2.847917	24.35817	22.94639	21.64228
63.88149	31.99156	29.57617	33.69262
23.08784	4.329748	4.993541	5.063209
15.71045	6.662186	5.720667	6.315989
0.811096	0.565338	0.735669	0.586372
46.90432	28.49815	29.3991	27.61517
15.88062	3.838985	4.759173	4.193638
9.352975	14.05905	16.90186	13.40169
27.26154	26.8535	29.96798	30.10775
1.076289	1.809667	1.81594	2.447028
1853.732	393.7534	385.2398	394.3476
260.8416	190.4954	196.6982	201.804
1.215465	0.220486	0.607276	0.248961
7.680142	2.077943	2.127806	2.587145
6.679271	18.55075	20.40476	20.32142
2.213162	1.493101	1.478078	0.75745
1.095088	4.009696	4.0602	3.962945

0.263313	1.92111	1.652093	1.73336
245.553	127.7663	123.06	129.2581
0.251986	0.419073	0.321823	0.376006
50.90429	20.40794	17.70368	20.29797
34.8751	36.98093	32.1702	37.4507
31.19853	7.317911	7.56134	8.871666
8.522816	37.17864	34.82687	36.3577
0.931229	2.276058	2.60411	2.346922
28.96174	3.363706	3.091507	2.559016
1.793187	2.139209	2.433613	2.534138
53.539	73.94024	74.59749	81.09798
19.62706	93.34739	86.84379	92.67297
61.46546	23.40863	22.13535	22.92598
0.462287	1.665713	0.745891	1.062173
11.27144	19.2888	19.67938	20.1898
0.96691	2.787781	2.060492	2.005127
12.72946	25.64551	23.93549	27.7543
1.993581	4.331945	5.077835	4.77521
0.474675	0.739592	0.895333	0.914242
141.2152	106.1365	99.27558	105.5285
1.31672	3.49973	3.041002	3.874316
0.960248	5.108067	5.183553	4.692386
41.6775	21.46624	19.14614	22.25926
0.788513	5.248226	4.159887	5.019698
2.209052	7.768601	7.427857	7.359404
17.88248	2.43004	1.524682	0.992823
417.5232	171.2893	173.1044	173.8755
7.507797	30.38191	28.30575	27.01358
1.056391	0.691569	0.394389	0.508028
0.244553	2.462769	2.423258	2.81187
3.453132	17.4322	16.18359	15.81657
0.225076	3.052705	2.682201	2.541749
3.515676	8.394472	7.220255	7.215171
5.910742	9.051272	6.531358	6.806502
7.99138	4.232568	4.097574	3.575769
1.373285	0.457321	0.418924	0.658139
27.83455	11.13018	10.09697	11.87912
0.867833	0.211248	0.265981	0.189433
4.011896	3.875826	3.44607	3.05752
2.195765	1.890028	2.120734	2.313032
1.38982	0.525849	0.53349	0.667481
6.039308	2.652985	2.860693	3.042951
15.55418	150.3093	126.9053	110.9656
44.31202	6.377088	6.911427	8.118591
13.49538	4.496007	4.312207	5.16193
0.469535	2.290681	1.695795	2.138946
10.91699	26.74701	25.47723	30.46893
0.061338	0.874002	1.138623	0.721526
3.947497	10.82561	11.07434	11.9219
3.105679	0.981934	0.886613	0.951302
4.481608	9.309511	7.96524	8.322645

1.510374	0.400872	0.987532	0.948049
12.25479	28.80519	26.31844	29.07285
11.21348	6.296699	5.137847	6.541039
102.5334	227.0649	226.7635	235.3977
3.656513	6.68092	10.36134	9.690655
2.176855	5.047144	5.889202	5.251676
5.055104	12.63451	11.48113	11.67959
11.66984	6.661292	7.317246	5.967391
60.05516	154.5757	152.8088	159.529
32.34557	15.96424	15.95369	15.25081
1.114116	7.701145	6.089931	5.237303
33.84481	13.02968	13.48286	13.54356
128.5036	36.31198	34.25132	37.45291
68.13715	32.53991	31.07761	35.76537
17.33595	7.864296	8.833764	9.654309
5.18131	3.022244	3.053619	2.167853
150.6374	29.84929	29.4188	30.05855
38.81978	11.35935	10.9232	10.92152
10.2261	5.444455	6.587324	7.367885
86.6657	4.888404	5.625787	4.434811
3.684046	10.01588	8.555392	8.794563
26.74321	13.53254	10.95102	12.3633
5.830762	0.991413	0.851518	1.066047
49.08496	16.99045	15.9092	17.7665
0.861641	1.875492	2.305474	2.70883
17.96814	35.44051	36.90965	37.60691
0.31411	1.248688	1.009622	1.157333
1.834139	22.79728	21.28602	25.24884
5.606535	3.018144	2.393421	3.50311
6.750742	2.579631	3.105145	3.950136
8.499202	1.937208	2.279944	3.056951
59.92299	29.56909	29.32806	28.42132
1.726332	0.58546	1.063147	0.715267
34.3986	12.77323	11.75398	12.63372
0.858124	0.806948	1.330428	0.870512
1.171664	3.905482	3.375983	3.336572
4.627521	0.699093	0.672059	0.738615
0	1.416664	2.014584	2.180741
54.46194	128.4745	127.5343	130.9229
2.733898	4.756671	6.128857	4.304554
2.223711	1.595428	1.79078	1.393172
6.27943	6.3495	6.117494	7.193822
5.013298	15.35835	16.92264	18.46458
0.430905	1.388715	1.465145	1.445076
22.81106	7.130117	7.076875	8.376559
1.235254	2.115695	3.601261	3.120524
59.36937	19.0323	15.33821	14.1412
1.8463	0.466938	0.623151	0.637673
0.298642	0.912697	1.352206	0.829668
0.433227	0.802691	0.600685	1.259829
1.642182	0.097369	0.312769	0.12855

6.343701	9.618429	8.821526	10.07796
103.5778	21.41051	20.69127	21.90668
1.25223	0	0.17513	0.260412
1.114552	0.200753	0.346383	0.856154
3.767687	13.23153	12.36043	13.68315
0.694949	2.313935	1.751113	2.300632
11.62696	4.352081	4.413077	5.428527
47.67265	22.93432	21.19987	25.22085
47.61614	17.83287	16.22429	19.04642
414.6023	52.44179	51.29203	56.68534
3.867817	8.477996	8.697984	8.56236
2.043324	6.651446	6.297482	8.472828
22.29585	45.83395	41.85241	45.62423
6.654643	0.280519	0.072205	0.169154
0.440282	2.357059	1.848105	3.19617
22.35251	11.87808	10.95439	12.69866
38.38758	92.32775	86.8347	96.78961
0.3924	1.313185	1.347142	1.233719
7.584772	23.44805	21.44088	26.97424
2.743127	6.838327	6.73237	6.941982
1.048406	0.992246	1.073066	1.88954
6.450341	21.44441	23.52722	24.10021
64.81473	45.62128	43.88322	44.70136
0	0.420384	0.447354	0.441033
322.9008	116.2137	112.6002	119.1784
2.669239	0.381754	0.807605	0.981855
1.033392	1.606664	1.897344	2.144067
7.231779	11.63154	13.40499	13.85872
2.124605	6.195866	5.846447	6.15785
2.104216	0	0	0
8.03648	3.040524	3.341457	2.664798
597.3962	263.6707	247.674	272.2138
0.115306	0.320498	0.290259	0.502921
0.290025	0.347526	0.148997	0.339694
3.568925	5.560633	6.070221	5.199471
0.658242	1.645075	1.721237	1.615989
2.328035	6.316189	4.83146	5.168485
1.500626	0.643557	1.326624	0.719725
14.34124	15.30729	13.56928	15.11959
5.222544	3.476648	3.394523	3.888213
2.619033	7.344255	5.274927	4.981921
41.65628	0.418688	0.060323	0.114296
3.774577	3.874901	3.264781	4.203944
3.505407	15.00999	17.58361	14.90814
4789.288	1168.298	1097.149	1151.204
0.545886	1.588582	0.967997	0.816918
28.6097	13.07892	10.71419	11.31166
0.230319	0.833007	0.940264	1.156137
0.281069	2.2075	2.395327	2.237594
2.29548	6.853306	6.138111	6.590271
3.468079	0.455496	0.367794	0.376501

1.087023	5.296858	5.969603	5.251157
32.89318	5.044243	4.628494	4.593484
1.079775	4.722454	3.965469	3.537309
1.359884	3.388331	4.204618	3.206579
3.41635	6.888265	8.16816	7.690822
9.797917	16.05841	15.74106	15.96918
44.48753	6.431163	5.696021	5.569514
20.55934	61.72702	60.72957	65.57918
2.554461	1.42493	1.327667	2.001281
4.591111	18.75472	19.43798	20.66475
0.407883	1.030781	1.030917	0.926102
181.3577	72.16582	67.86864	76.74209
1.318265	4.59578	3.221812	2.880394
1.034258	1.291388	0.585522	1.153951
9.53155	5.066212	4.886766	4.924452
21.28067	55.95483	55.92692	58.02635
37.0012	10.95564	11.51224	10.97088
0.895823	5.075942	3.802811	3.654999
33.50715	81.35011	82.64561	82.79625
3.491101	9.849879	10.28468	11.16717
22.88162	12.91728	12.23531	12.75777
2.564677	4.568998	5.311113	5.074646
40.47606	26.68036	25.76107	27.74069
4.840329	4.142322	5.245545	4.544528
1.433788	0.833126	1.262449	0.948899
25.16915	71.62672	68.11859	72.23163
3.044305	5.349178	5.684263	6.973327
3.254036	9.832766	7.336887	8.686294
0.217467	1.059879	0.535191	0.82769
24.26739	9.862759	9.412277	12.10843
1.159027	3.656919	2.71314	2.797679
7150.164	3367.146	3330.591	3493.176
7.261966	12.78791	13.62076	13.91887
1.102084	0.185994	0.411553	0.196576
7.634802	3.178754	2.553525	3.326149
30.51343	12.10673	11.2034	11.15142
35.0943	13.46732	14.50454	14.36575
12.82804	26.54802	25.32616	24.46322
19.65672	7.312638	4.909673	4.681486
9.241881	6.530618	6.306247	6.038611
3.233459	35.25624	32.62359	35.52786
0.800532	2.172392	2.280935	1.639261
9.354946	9.35897	10.69229	9.673477
0.771413	1.080514	1.340929	1.493183
7.548819	3.79847	3.785913	3.387274
4.523067	2.279302	1.550014	2.42355
37.02083	12.53083	13.61195	12.58905
13.73247	45.9086	47.83777	47.63012
26.78392	14.02203	13.31113	14.00619
2.56925	3.487509	3.055527	3.086026
0	0.193857	0.138982	0.331848

0.226068	1.148373	0.879887	0.783585
98.57435	44.7643	39.45542	41.58359
6.151464	7.860998	8.28476	8.904015
3.649452	1.670107	1.669705	2.176735
76.53645	32.65866	30.6886	33.6918
4.637104	4.830705	4.182357	4.7766
67.04425	27.38094	27.29242	28.44832
2.218247	10.1377	8.985224	9.68024
81.76452	161.8284	162.8468	172.3743
5.287313	2.744603	2.857918	2.920192
6.867866	4.739425	3.515158	4.176208
26.73474	14.33809	12.70737	13.22522
73.30023	36.75792	33.60807	34.6653
2.341266	12.62775	11.99168	11.554
0.585884	1.813085	1.386673	1.636011
0.983803	1.912577	1.952521	1.83019
70.6164	180.6021	176.9901	168.8247
5.92482	1.52161	1.981423	1.492486
25.07381	17.93607	19.31227	18.57831
48.09261	24.38431	23.59131	25.45928
2.663692	11.58493	9.452222	12.99908
0.67643	24.91043	21.39918	27.28109
3.748609	7.81551	8.947451	8.978561
1.351472	1.314207	1.234457	0.97922
34.97237	70.88093	71.15635	73.02667
0.258606	0.229668	0.311966	0.433301
30.36927	7.417751	9.491231	6.912587
6.48941	11.65243	11.89579	11.08472
2.879736	0.700269	0.783197	0.485695
0.784052	1.995468	2.073741	2.963166
10.06434	2.785494	2.241393	3.647015
70.37437	29.71653	30.87101	29.90038
86.79258	28.21669	28.10139	29.30665
59.99457	434.3145	430.1035	449.05
4.415059	9.472886	10.10152	9.973379
0.196032	1.506847	1.544843	1.735113
4.883948	13.27224	12.13973	12.44193
16.85301	41.39209	43.97694	45.64108
6.925288	3.276611	4.50455	3.263728
0.338011	0.463332	0.343106	0.436714
3.991519	1.69773	1.686318	1.808482
12.94407	8.040568	6.775412	6.600971
4.861359	32.35226	31.06276	35.08214
1.970214	7.134386	5.748605	7.026206
8.709671	7.330979	7.91022	8.146862
51.73198	37.14265	33.34559	35.90302
7.111419	3.254283	3.365755	3.654414
0.445953	0.603445	0.808767	0.478646
1.979944	0.660062	1.005367	0.769877
108.036	63.18396	64.14979	69.56024
2.683045	1.335665	2.095901	1.112417

8.870244	4.601151	4.038218	4.837724
90.68101	44.83565	45.63006	46.64806
5.604566	11.80936	11.28649	11.64372
70.72178	20.96548	19.63957	21.59818
84.05898	14.22726	14.526	15.32156
1.232453	3.425467	3.812087	3.24096
1.049522	1.10913	0.558565	1.06552
0.220941	0.29422	0.088684	0.061553
4.216247	4.245017	3.869608	5.319708
3.976174	12.69459	14.37442	13.51773
5.761088	13.6691	14.36096	14.10771
38.44654	18.5502	17.11917	17.27805
5.161437	12.57374	12.6894	12.70419
16.13204	3.600682	4.17884	4.115282
1.154238	0.17854	0.338454	0.31713
0.651192	2.900252	2.018404	2.181813
0.737592	0.231192	0.394624	0.363167
0.727819	6.013747	5.762164	5.775146
124.7024	39.01661	39.34767	42.12122
2.494391	3.464378	3.096371	3.545141
18.36987	5.560017	5.547819	5.802641
0.855223	1.073812	0.985726	0.972113
13.20637	3.708934	4.950475	4.705647
53.51434	135.7064	103.2124	123.5559
2.693776	1.642307	1.55945	1.229222
81.81057	37.44326	39.46463	40.20489
0.350842	2.950346	2.452228	3.299386
6.637948	3.770609	3.879895	2.879537
3.044655	11.73944	12.65843	13.77596
15.91373	23.2151	23.62916	24.8167
186.3724	0.613678	0.745933	0.866888
0.86748	6.251305	4.984023	5.489752
6.579443	0.39018	0.292479	0.738633
16.60237	8.167422	6.517757	8.86235
1133.087	302.7719	284.9964	314.5011
0.651843	0.758954	1.107751	1.135447
2.571773	4.590987	4.77018	4.502975
0.927559	2.354136	1.709716	2.330446
37.29234	14.49993	15.487	15.75477
0.962152	0.390998	0.534224	0.650696
0.652086	0.469608	0.358739	0.135147
8.341858	5.528106	5.222616	5.638302
0.660486	1.16304	1.488631	1.57143
1.004714	2.52802	3.412445	3.557214
6.82099	18.53518	18.48433	17.78719
119.3462	852.5703	853.6055	896.1521
0.785342	4.859483	4.099937	4.151675
0.133269	2.308592	1.676998	2.135628
3.836448	9.800292	9.33156	9.258366
3.892503	2.77585	2.440979	1.760372
39.23326	6.343777	7.03856	8.516309

6.222239	59.2678	60.16307	60.90952
4.683202	3.238544	4.403014	4.462472
1.124784	3.216822	3.73183	3.054729
3.381136	6.497386	5.860491	5.791113
0.425964	4.97193	5.116238	5.5581
21.73484	50.83178	51.23703	51.53314
13.96074	20.04509	19.46873	20.80269
0.713582	1.917204	1.138218	1.298656
12.22691	1.160581	1.197336	1.303752
21.04524	8.294448	8.436412	8.152469
2.296867	6.637563	5.069794	5.646363
0.414145	1.299645	1.555953	1.930796
60.27047	22.24687	23.15663	24.24586
0.064768	0.288702	0.316218	0.319289
17.95286	28.41286	28.62424	31.05434
0.688719	1.592126	1.045017	1.607088
19.34157	5.964811	5.700418	5.777329
0.29535	6.423416	6.387941	7.798417
29.68077	51.10618	58.53924	47.52078
1.021677	6.860065	6.937325	6.828515
0.326739	3.357287	2.984124	2.701401
13.63047	7.146699	7.131479	7.890129
3.440102	7.184874	9.456905	9.438527
42.62372	12.44739	14.19507	12.29068
30.56736	13.98841	13.68666	13.66379
46.50746	19.63724	19.32776	17.63286
18.22779	70.81627	63.65134	74.0645
0.557118	6.297021	5.143527	4.52137
97.92757	24.99248	24.36391	25.44226
1.040013	4.333108	3.605285	2.531435
8.337539	23.87327	23.57005	23.70987
0.041709	2.352521	1.911803	2.662526
2.859882	10.09343	8.383722	7.615776
651.4665	157.3705	160.7853	151.0398
2.767923	6.431365	6.872447	6.029266
29.53853	11.98982	12.36053	12.51813
1.246318	4.002081	5.170908	5.424372
175.3168	64.67484	67.12128	66.96089
14.59703	39.08857	37.91994	40.56011
0.149842	0.233095	0.208621	0.190247
20.99948	6.899039	6.771628	8.051906
3.467746	1.87481	1.289288	2.137278
8.165077	21.44324	20.84587	22.37702
0.473552	1.928837	1.202113	2.263013
0.866144	1.670249	2.330569	1.728649
0.916865	2.447476	2.55325	3.212613
11.56973	1.564759	1.491132	1.683775
0.337619	0.507836	0.188971	0.372833
2.8927	1.333756	0.392357	0.995949
5.197097	2.225293	2.025842	2.776383
1.137497	23.54862	25.42659	24.23394

0.720114	9.100624	8.81874	8.783624
2.876526	1.828066	1.279677	0.944949
37.78561	16.65294	18.41332	17.75638
212.4066	56.7946	57.44233	61.41183
16.06604	4.66765	4.865054	5.996242
0.74401	0.981245	0.167466	0.689748
19.55736	8.64886	8.868221	11.11668
1.815035	6.802863	7.121102	7.414292
12.5736	11.19785	12.61719	11.13802
13.19764	6.093306	6.963649	7.009514
6.871615	2.397004	2.254088	2.26198
177.3628	80.53572	80.01008	88.28986
24.4044	7.16662	5.644533	6.44868
1.077552	3.419344	3.233451	3.282588
0.660866	1.839988	1.324879	1.711372
5.053811	11.77969	13.03399	12.77436
1.419076	1.282975	1.236292	1.334644
17.47367	35.0817	40.37991	40.86192
19.45971	10.85807	11.1764	11.94786
11.21186	1.891411	2.854136	3.53058
7.534267	25.15551	24.66076	26.34506
27.7485	7.77617	9.353928	10.38721
7.498058	10.15364	11.73137	12.26476
0.124605	2.756142	3.147866	2.458452
0.045319	2.253873	2.020713	0.804961
8.038298	3.851443	2.69629	4.041423
73.03639	26.99419	28.63285	30.01289
2.502738	17.69994	15.12759	15.95494
164.4115	742.522	753.9791	766.9443
63.95387	149.9795	140.9445	152.4815
55.42975	1.666572	1.514115	2.332236
1.635413	0.436938	0.441849	1.032735
68.00485	35.43738	30.87808	34.21778
0.67665	1.99617	2.323422	1.42424
8.621105	1.644677	1.731197	1.903994
2.940647	1.523314	1.266372	1.570817
36.3236	7.069866	7.853409	7.678187
0.956282	0.951253	0.328485	0.822148
0.920208	0.449673	0.584125	0.275965
14.62705	4.311399	4.95598	4.004404
6.873771	14.86583	14.63283	16.03376
74.00286	34.04804	32.42742	32.97122
2.174227	5.203052	5.113667	6.323571
1.093177	0.123658	0.401649	0.523334
1.357462	2.417093	2.854534	2.373037
23.30712	51.32138	49.47826	55.8663
11.12572	12.12417	11.4991	11.6306
1.707727	0.091101	0.492934	0.816374
1.387911	3.417026	2.383389	4.333494
608.0772	305.9684	305.4651	316.6302
12.78674	48.31312	46.70348	51.5131

0.657163	3.749985	3.014043	4.412878
2.862573	14.74025	14.09604	15.76384
0.45427	0.332168	0.319399	0.837157
0.418481	1.523954	2.485704	1.987252
57.70287	7.083493	7.112402	7.963511
3.816625	12.17009	11.08887	11.43715
12.05104	8.599437	7.636565	8.659145
0.590645	3.333272	3.241814	3.087449
14.85275	8.040469	6.857178	6.157874
1.187595	28.63709	26.74635	32.85646
0.487618	0	0.012685	0
5.362331	3.046958	3.459543	3.290078
51.37253	134.3703	124.9047	140.6629
0.783471	25.67734	23.27883	26.13902
10.40458	4.382829	5.135609	4.20462
4.531823	17.5776	14.72951	16.57389
20.36762	3.939638	4.353258	3.823709
40.58305	18.19661	19.13906	18.59211
25.22571	8.310179	7.801784	9.340199
150.5685	20.76255	22.2629	22.11039
22.61221	11.21522	11.40644	12.45914
2394.337	899.2347	938.1737	938.0735
102.1478	30.34112	34.1327	38.54877
14.33325	6.178002	7.340209	6.693135
7.724836	39.53553	40.98213	40.2816
136.5261	51.9083	49.68879	56.30638
375.8409	836.8311	820.1319	867.2282
0	1.667215	1.88179	2.309451
68.17742	25.74693	22.82533	23.0011
0.433819	1.424807	1.448732	1.438849
7.433216	3.113543	4.118169	3.715181
15.10241	5.630628	5.644882	5.305111
13.21499	27.88162	28.33259	27.28533
0.256537	2.492094	2.928867	3.148839
26.74582	12.69157	12.06592	11.1778
51.89177	24.52955	27.06789	21.87017
28.27521	3.342507	3.112017	3.680228
0.193141	0.850935	0.954636	1.488819
0.033433	3.361903	3.45844	3.553373
4.586715	23.51551	21.40751	22.65858
18.71926	23.13148	24.96485	22.75394
43.88847	2.32465	2.79092	3.404385
1.283704	4.915173	5.113342	4.635179
21.18087	7.18002	7.993043	8.393215
64.89894	33.81591	30.76572	34.89919
22.10638	11.9631	10.97253	12.64507
31.50333	14.9877	14.30548	16.65829
28.63001	10.48564	9.536196	11.66389
7.257297	33.32312	28.92971	30.80908
19.46555	6.177533	4.848372	6.707741
0.967307	1.685405	1.287447	1.927766

0.178109	2.003827	3.087956	2.356811
5.827088	9.696879	7.897208	9.062207
1.006786	0.407111	0.401491	0.334472
7.341577	2.791137	3.609926	2.555863
16.3381	35.33274	34.52557	38.61789
6.074738	20.56264	19.54389	19.60147
49.31609	15.15473	15.08182	13.78242
3.682255	13.79019	13.43659	14.57338
12.79169	5.472169	5.04166	6.224735
24.44876	32.1842	33.70133	31.36619
2.725086	1.491564	1.194463	1.076727
232.5617	516.1898	538.7041	548.0092
0.130707	0.46648	0.668332	0.757867
17.55472	36.83882	33.01331	33.78233
0.841538	0.424161	0.681498	0.334658
2.380376	1.397025	1.418794	1.431189
7.06604	15.12554	14.51233	15.04904
10.63793	7.680434	6.664049	8.839716
0.163569	1.009321	1.405373	1.662651
1.996808	0.449548	0.814064	0.881457
4.142305	2.643255	2.88497	3.606612
347.6158	11.35581	13.20388	12.23173
1.772011	5.918999	6.415087	5.306465
1.838447	4.637048	4.306336	4.535804
0.360321	1.431288	1.339218	1.656539
1.109887	2.116538	2.465223	2.017891
3.070394	1.507666	1.471337	1.430268
42.25844	22.57966	20.89346	23.21137
0	0.937152	0.778128	1.14455
1.811876	0.62593	0.668123	0.864355
68.13619	35.48719	32.72462	32.0306
5.630995	7.413046	6.751094	7.364961
2611	772.9301	745.8169	808.9578
0.048232	1.084647	1.167421	0.879939
8.057693	24.10053	20.7499	23.36445
4.452714	1.9492	1.785272	1.899771
1527.594	392.0272	372.1752	401.8172
17.44801	31.17613	30.40378	33.07195
1.167312	1.337265	0.897564	0.851328
17.45368	1.672934	1.391653	1.502764
4.048162	7.19019	6.713922	8.886558
2.939736	1.896411	2.311959	2.206597
1.64772	2.121066	2.223465	2.177498
1.271617	4.561491	4.790071	5.701327
0.23076	0.081351	0	0.03707
2.715432	16.43309	19.44074	17.40322
2.384519	2.192533	1.564422	2.532243
13.12482	9.785506	10.48847	9.082448
9.102407	7.502249	8.236528	7.420344
1.02623	0.370973	0.762242	0.780521
3.057651	0.60056	0.584028	0.539997

6.180964	2.431673	1.788541	2.27988
596.147	855.8685	781.6208	812.1393
9.373451	24.75212	27.93004	27.06458
5.296604	2.062465	2.297996	2.177364
0.848824	3.462738	3.398172	2.996845
2.604665	4.902472	4.975338	5.099292
4.040831	33.71612	33.1244	31.8314
1.149669	3.613285	2.736522	3.246886
16.1851	17.14168	16.82195	19.72929
15.36035	8.214021	9.008584	8.33
0.133326	0.710697	0.528316	0.702046
15.12254	7.104512	5.222119	6.384025
1.397727	0.750287	1.094371	0.810033
0.428093	5.237342	5.236187	5.2939
9.361075	39.29738	38.48235	35.47853
0.930235	2.150476	2.848243	3.014171
9.683533	1.025885	1.279463	1.21694
2.523382	2.937037	1.78742	2.066147
2.017266	6.684554	10.99761	7.378089
81.35788	5.133273	4.359755	5.271547
12.58803	45.69651	47.5161	47.29466
0.063582	0.91608	0.971285	0.657603
0.235233	1.150233	1.471666	1.592467
0.042761	1.119531	0.724293	1.098674
184.7576	166.991	162.3948	177.4384
2.824412	19.01431	21.97131	22.58745
83.39862	228.9445	224.0251	254.3871
0.643293	0.300892	0.31363	0.24563
6.559841	1.361925	1.632775	1.395054
1.0224	1.838928	2.246212	1.95682
1.786976	4.731503	5.524244	5.031425
3.355769	1.205475	1.091264	1.294832
9.950233	0.080785	0.198682	0
2.633898	19.12103	21.18964	21.81579
3.24856	0.201375	0.187173	0.251475
7.784159	1.212742	1.697818	1.316339
1.203964	2.844841	3.645436	2.351607
91.62045	13.13133	14.39093	13.12329
19.31497	5.691278	5.451755	5.241924
2.366973	8.548638	7.395165	7.344453
103.585	39.30929	39.7216	38.66525
13.82376	8.019796	7.552466	6.121562
25.86811	5.171873	4.319841	4.605599
0.296853	2.399853	2.144186	2.421762
90.66222	31.9938	30.12102	33.74931
0.038296	0.454972	0.394109	0.197499
2.762994	1.001979	1.295281	0.708114
5.140767	4.725431	3.71425	4.728228
2.409634	17.53057	17.33495	19.22309
165.5163	49.62115	51.25013	50.26051
8.627023	11.11279	10.89593	12.235

5.908813	2.030353	2.085985	2.884108
0.123056	1.235199	1.349685	1.22938
2.400405	5.931365	4.462327	4.757501
1.564546	0.739928	0.40731	0.815909
72.46557	29.58404	30.76379	28.1406
1.773552	5.162831	4.821885	5.11425
0.059687	2.211958	1.794636	2.26271
3.706743	4.957102	5.974347	4.93541
0.957374	14.05825	12.21526	13.7429
28.76338	12.97079	12.07422	12.06628
17.48601	37.10108	34.73473	39.7843
1.27459	3.747424	3.248148	3.070878
0.230351	1.031666	1.09045	1.623742
80.40064	208.7001	215.7271	221.0854
1.061371	1.019641	0.791177	0.888884
2.18537	11.99489	10.38693	11.71433
0.155592	0.964435	2.212625	1.266073
1.281935	2.489345	2.784883	2.535638
27.06451	7.855274	7.018598	8.001018
1.98657	4.036372	5.054347	3.4056
21.58545	47.09211	46.23705	47.42951
6.441634	2.483791	2.149548	2.502378
1.364967	0.736596	1.432263	1.626915
27.47273	14.42278	12.83176	13.83174
0.480955	1.072445	0.653031	0.779047
3.334453	1.401042	1.846038	1.84392
4.099329	8.180084	10.65264	11.15185
6.323283	3.972612	4.217258	4.700863
0.617541	1.161666	1.77172	1.859245
0.162518	0.524651	0.644909	0.562875
0.781927	1.832328	2.534729	1.93363
1.632088	6.9512	6.713971	7.401559
90.68305	217.5651	209.0341	216.9067
1.593836	14.98173	12.3445	12.69399
4.204244	16.73963	15.27081	15.42941
2.062861	1.431897	1.372476	1.914277
0.087273	0.78196	0.722659	1.063667
0.021939	0.699833	0.805289	0.648006
7.980528	1.539925	1.695855	1.417603
36.54664	80.09377	82.15205	81.88304
31.45227	13.35338	14.79292	16.27863
29.95699	66.95482	60.53559	68.51168
25.20972	25.1368	25.35921	23.83424
5.059606	1.759523	1.03248	1.653006
5.838408	17.88952	15.89751	18.34689
0.379705	6.665014	5.373877	6.009062
0.161273	1.290456	2.165801	1.510186
202.9875	41.04168	41.10835	43.4787
200.4178	76.32148	86.10961	83.30186
3.529042	3.451629	3.778131	2.827424
7.67218	43.16424	40.45526	42.60059

3.697698	1.190697	1.447108	2.109108
12.51694	3.056092	3.067144	2.433833
5.866653	2.913984	2.554323	3.373413
2.203603	1.909653	1.802475	1.831328
0.369256	1.032786	0.360582	0.275043
19.05924	1.060604	1.119034	1.071764
19.06555	10.1183	10.55883	11.30289
83.25716	194.3397	197.0479	205.0861
0.819051	1.098637	0.571794	0.36031
0.488272	1.045159	1.151309	1.040541
0.418046	0.912079	0.5512	0.679956
0.328423	1.21352	0.696925	1.199029
4.142889	1.703534	1.416824	1.59697
7.920043	12.97261	12.60226	13.63807
2.607569	1.119203	0.700446	1.374147
3.627803	12.2206	14.71759	13.90434
2.004828	4.051	4.463049	4.178097
16.82554	7.551299	8.17695	7.717432
5.172149	2.444563	2.650133	2.343053
637.849	1584.042	1579.219	1624.514
2.71698	3.473949	3.4212	4.66601
1.464855	4.098486	4.400346	4.049935
2.11097	0.050667	0.240761	0.211181
2.203995	0.547511	0.678532	0.785294
1.111208	0.395376	0.224659	1.209032
0.51228	4.362784	4.430562	4.409586
0.13632	0.833489	0.655872	0.502492
30.89403	11.30399	9.881465	10.89694
0.262806	13.83948	11.36631	12.14264
0.26821	1.883911	1.383921	1.864984
64.31593	25.59769	24.25364	25.27386
0.77503	2.0135	2.263414	2.463153
2.894382	7.96585	7.871347	8.579036
1.702787	6.384808	5.140501	6.04361
1.565817	4.071799	5.119407	4.124758
4.029898	2.115891	1.350496	2.153511
5.757858	1.898677	1.846911	1.621204
1.681104	11.57494	11.67537	10.45274
33.26916	18.06273	18.26382	17.42651
1.598917	3.738244	4.540047	3.74778
190.9616	70.28249	59.22481	62.35466
0.12167	1.054162	1.166025	0.940913
2.491475	3.930798	6.061024	6.077299
12.77689	2.133152	1.605741	1.510556
29.09443	14.02411	13.11317	13.451
110.4563	282.6807	277.7487	291.6471
46.9598	22.44399	20.60196	21.39635
16.27753	901.6607	863.4858	903.4902
2.316341	2.3044	2.583025	4.402233
1.870216	12.92879	14.27902	14.13097
1.51688	8.886854	8.41453	8.506754

0.705835	1.574833	1.849727	2.136491
5.789155	2.43397	2.285135	2.411598
0.998016	1.099804	0.985596	0.948216
2.066887	1.086426	0.810024	0.829064
17.30243	43.26465	43.68837	46.85776
42.98406	55.12743	51.79596	57.6364
1.121313	1.626902	2.110783	2.287445
2.440344	1.129543	0.985089	1.279512
130.0513	315.0552	299.4187	316.0049
28.23899	14.37691	12.31576	12.81338
4.262569	1.789823	2.074113	2.321511
0.937285	0.712988	0.909985	0.752893
0.174912	1.118391	0.79735	1.258117
1.600715	1.494339	2.565177	1.584649
1.463717	0.329554	0.156483	0.191446
1.029078	0.94698	1.554604	1.479162
0.549016	0.835165	0.690575	0.855471
1.01847	1.329105	1.026283	1.221563
11.55143	4.82342	3.719463	3.843704
4.605862	16.94477	13.63745	15.02757
2.207208	17.84024	16.64503	16.38728
10.97992	0.378999	0.568506	0.707662
6.701389	3.773532	3.78394	3.15899
2.287017	16.81611	16.91175	18.35001
0.35574	1.798298	1.930909	1.512937
4.601591	14.022	13.28583	14.68863
20.40786	16.90485	15.37781	16.42425
0.793494	0.477739	0.637475	0.635348
12.29627	13.58044	13.72532	13.19118
9.055198	6.023859	6.048583	6.364547
6.618465	2.023762	2.007489	2.934955
704.9283	37.64494	36.34761	36.81447
15.13255	6.984098	7.436811	8.340471
15.19524	44.93662	38.68123	40.80166
199.2385	62.31321	64.75108	64.15468
0.251116	0.626319	0.617763	0.986071
1869.478	766.8515	763.9294	784.0906
13.2417	5.437157	6.07578	6.025638
53.99735	528.6457	552.0759	557.7591
17.41846	6.721834	5.543825	7.141063
0.517834	1.996197	2.47352	2.587698
22.90085	49.64278	55.15895	53.83755
0.967902	2.015095	2.631066	2.526701
22.44657	10.40443	11.46505	13.18877
5.181698	9.739341	10.5017	10.3119
0.671251	1.013998	1.465825	1.318973
4.59583	5.207252	5.210484	5.096601
2.385705	0.145297	0	0.044091
0.531654	1.561221	1.337835	1.404446
88.23345	30.60894	31.08156	31.54845
160.197	16.38609	17.38622	17.36325

1.644595	1.02525	1.117601	0.829371
9.986887	16.29559	16.31895	17.064
6.8137	2.930167	3.196192	3.32266
1062.206	486.3807	521.1746	526.5828
2.995553	7.454885	8.929101	8.909781
0.222713	1.338342	1.38767	1.361649
3.570029	9.465482	9.621255	10.66805
0.306807	1.487484	0.772703	1.064101
0.981733	5.169548	5.44571	6.141349
9.852321	40.24556	43.43164	41.54632
19.04123	54.92009	52.05003	55.39665
1.547765	8.340468	8.065467	9.191832
2.690043	41.5203	43.12422	45.69761
14.25579	5.502799	5.449905	5.389115
3.442483	10.35586	9.106951	8.569679
2.988578	23.56368	16.62993	19.19741
0	0.843351	0.414663	0.50697
5.791025	29.54391	27.0266	29.36626
29.08646	10.41799	10.3572	10.984
31.90837	62.13799	60.5534	63.20256
145.7125	305.6048	314.3201	317.206
59.71678	29.97366	28.07601	28.49953
0.890428	1.305433	1.295354	1.543937
1.511914	2.954475	2.26628	3.874313
3.330162	10.83028	10.34092	10.86044
4.612181	18.05518	16.44251	18.23356
7.963564	2.744974	2.823378	3.200397
4.391336	13.10896	14.16884	16.8504
4.143008	2.524437	1.495808	2.760278
12.8637	252.1606	240.0478	257.1562
6.210278	18.55792	17.68539	16.8346
104.8852	526.4858	489.5128	538.0028
3.097091	0.077047	0.054073	0.45612
9.873097	50.82148	50.66546	54.34509
12.59109	1.652391	1.21688	1.346151
0.456359	2.653399	2.787253	3.534187
20.96108	50.33544	49.36046	51.12602
8.966637	3.42674	3.89221	3.256323
0.359632	0.66295	1.363882	0.773805
7.614498	14.04314	16.35728	14.52966
15.22549	6.561578	6.556243	8.454082
3.11659	1.402398	0.500999	0.951127
0.18917	1.182697	1.232527	1.584952
10.34551	32.79311	32.5428	34.86568
1.190518	4.173512	3.993269	5.08934
37.04321	19.67428	21.24941	16.30017
9.211426	17.33986	14.89652	18.90883
2.746492	6.638829	6.085211	6.146586
11.44926	44.11994	43.61643	44.56579
7.050799	29.02456	25.99196	28.02284
4.207843	12.24283	14.35689	14.95942

0.173185	1.542607	1.820275	2.33357
0.555249	0.322329	0.138495	0.24244
0.199613	0.497136	0.465136	0.436433
0.653295	1.82011	2.113708	2.479584
6.074735	13.87938	15.29608	13.9527
17.56412	36.6425	35.96982	38.38341
7.871733	20.02823	19.64521	21.35539
1.438674	0.564325	0.445268	1.416576
19.73075	11.96866	10.38849	11.70146
75.6806	40.27248	12.67858	38.06338
55.24193	126.0726	124.1353	145.8401
2.073169	4.079749	4.441409	4.173369
5.474982	2.474887	2.798431	1.987144
5.622899	3.011315	2.511844	2.798587
24.26061	10.41876	9.511708	9.412716
5.326674	3.890817	3.773333	4.272186
11.78297	5.797533	5.577036	5.872848
27.71996	8.223279	7.635976	5.935536
77.90331	254.9737	246.5953	241.8941
0.350944	0.369521	0.196194	0.377202
7.741487	4.615191	4.45331	4.715459
87.56731	38.95152	29.35334	32.58901
7.453367	4.37499	3.763264	4.208403
14.38285	8.226666	6.834861	8.213117
2.888944	7.821766	7.084874	7.9026
0.766539	1.359387	0.864286	1.288924
0.547642	0.255479	0.18492	0.153082
84.21955	142.2561	133.3073	146.2086
82.09114	21.04251	18.97515	21.45667
3.38092	1.796138	1.449127	1.563577
1.627559	10.32388	11.63176	10.10529
18.90773	1.208088	1.678171	1.080224
2.317724	13.07334	10.81987	12.38712
1.061229	50.14333	47.73874	51.77867
5.528848	3.49004	2.718792	4.172589
2.589267	1.04707	1.277332	0.802602
123.2437	102.7678	98.42602	103.3195
44.46539	21.47941	19.98686	19.5728
32.03939	13.1476	10.40842	13.32797
4.901533	1.185181	1.175471	1.183246
78.91644	37.63437	33.88094	38.47091
3.269543	1.306358	1.435617	1.363028
1.031672	3.782173	4.266717	4.459134
242.1707	77.90425	78.12605	85.36326
7.500468	4.085574	3.852508	3.65797
0.714174	0.94052	1.496095	1.397027
67.6255	2.076056	1.892403	1.64307
3.425817	0.095658	0.238593	0.235082
248.3535	20.31607	20.58335	20.20461
5.98058	5.098134	3.792996	5.570961
1.371373	6.703177	8.245844	10.70405

3.005291	7.067779	8.092082	7.839549
6.977914	10.45116	15.18274	17.78515
0.322699	1.120135	0.852652	0.890022
0.340182	1.278714	1.828002	1.308862
1.087145	5.702035	4.912911	5.146122
1.859413	1.132902	1.228255	1.01461
6.104996	2.239571	2.896146	2.373443
87.71985	27.65487	26.57877	27.74555
35.46172	15.3019	16.1674	19.40388
2.962463	8.349929	8.43714	8.50031
14.25578	6.063924	7.140784	8.205774
278.1931	94.88535	99.22273	107.8007
0.984044	0.125447	0.15021	0.140785
3.377045	1.659837	1.896876	1.529197
14.39079	34.42654	32.0492	32.72737
84.16218	29.49241	24.14718	31.75018
45.00592	12.51058	13.64527	13.47838
0.665516	3.57786	4.947551	4.359645
2.299151	1.312598	0.870335	1.061839
3.690151	33.82965	32.44597	33.91509
1.998037	1.535298	1.754658	1.813847
10.57013	23.24	21.44121	23.95458
0.466923	1.233665	0.800447	0.948998
0.017867	4.618497	4.756741	4.915762
1.12485	5.339222	4.243031	4.181723
0.988791	0.827733	0.405656	0.44407
87.10855	29.43405	29.08443	31.88295
56.34885	308.2953	299.7349	310.3287
0.706103	1.054505	1.073578	1.620777
8.15205	7.843903	7.30227	8.982436
0.483597	1.452275	1.041913	1.400576
10.1863	4.934627	5.186799	5.003131
28.38402	13.54411	11.93507	13.93647
5.503978	8.729764	7.798928	6.932909
4.192604	1.445719	1.242603	1.579841
103.9954	37.55289	38.43206	36.90997
2.099805	5.24655	4.602787	5.564054
18.91782	51.23906	53.83754	51.1354
4.548263	1.676843	1.455518	1.851473
4.265699	8.925657	7.84213	8.641233
63.72256	16.28141	15.273	14.19503
0.101388	12.01716	13.84614	14.90954
5.576747	12.28582	13.18409	13.27385
0.291035	0.202195	0.103757	0.160491
8.223016	32.0181	37.37525	36.38909
29.30792	69.31915	70.47788	72.33939
6.854609	49.47471	47.97259	48.16695
5.373673	3.29611	4.041441	3.554221
0.782965	0.258638	0.201711	0.654343
5.003913	0	0.104782	0.169552
1.485507	0.755417	0.567871	0.521269

6.813873	1.847497	1.845424	1.755024
0.670724	1.352123	2.250016	1.407083
4.544798	10.07003	10.00942	8.941008
4.186625	1.955146	2.533674	2.017181
0.472562	1.015431	1.025233	1.296837
1.231601	0.883023	0.286457	0.652724
3.116103	7.343797	7.597702	7.820187
0.393502	11.82061	12.96768	13.59308
0.621606	2.031635	1.986311	2.161003
12.21469	4.485201	4.890753	5.554298
0.991571	5.457543	5.085367	8.322861
3.576054	3.753394	2.90491	3.463131
126.7479	306.3898	301.9388	313.4868
16.49344	4.457316	5.058597	4.075887
14.19069	6.690221	7.838	6.621189
39.78492	173.5555	176.3195	180.077
33.99137	0.243557	0.085906	0.130564
0.157552	0.302212	0.265783	0.548086
6.614719	14.69799	13.7021	15.647
0.700227	10.79332	7.49644	9.479995
6.683267	11.102	10.21224	10.70195
4.712155	12.21626	10.94933	13.16624
0.739175	3.452758	4.037407	4.17992
0.733311	0.913222	0.919471	1.183079
4.19998	1.60878	0.861393	1.608524
4.076733	51.19542	54.23026	54.69539
4.687042	2.113769	1.815507	1.608308
10.29135	1.07377	0.802984	1.120709
2.728902	3.457998	2.347664	2.609449
0.89331	2.00582	1.996968	1.68823
13.05539	38.46395	39.28634	37.28705
35.63868	37.29592	38.6008	42.41409
2.849471	10.61145	9.870324	10.54372
134.3496	96.77672	101.946	102.7767
128.871	62.59304	57.94107	61.2613
0.533042	21.61435	23.36176	24.47373
0.935901	1.151548	1.339848	1.280766
4.844012	3.55066	3.363688	3.95019
3.672918	0.923991	0.698101	0.61191
20.93237	48.34728	46.65176	48.24937
0.964041	2.154641	2.237786	1.836481
72.82183	24.7167	22.94651	20.39862
7.186078	2.631936	1.923729	1.388141
2.380652	3.628176	3.65643	5.037476
1.484137	3.410183	3.422966	5.224582
10.03236	5.112364	3.353919	3.830521
12.61042	9.178759	9.712997	9.3612
6.323487	39.25487	46.94931	47.44951
1.675913	6.208119	3.585594	4.878129
4.871894	14.23616	11.29066	10.76747
17.78847	45.01085	46.30377	46.69682

5.707961	2.684377	2.047419	2.535188
0.607601	0.671624	0.800974	0.539422
65.70767	26.87029	26.15381	30.53624
44.20512	2.64685	3.787072	2.948572
14.1196	35.87846	31.67805	35.8708
1.376671	2.993466	2.407706	3.640633
9.142398	4.531464	4.958402	5.062118
0.783182	0.29542	0.248864	0.321899
7.783247	23.14084	24.1977	25.39821
1.877961	5.802673	4.842938	5.224805
1.748054	5.045931	5.772321	5.983568
7.19869	2.719422	2.851014	3.491906
4.11399	1.044212	1.622504	0.400284
1.696959	4.082606	3.404324	3.813449
1.310557	3.47206	2.738178	3.55323
13.77641	31.83199	27.87123	31.09647
4.824659	20.87292	18.38346	21.22526
0.102674	9.037169	8.606911	9.487394
70.9848	30.3803	27.80298	32.30678
8.078595	135.4859	134.9273	139.0325
58.17064	38.40579	39.15608	39.41585
8.949132	3.313661	3.268752	3.095792
2.799594	9.041263	9.319233	9.872042
3.061507	21.67337	22.51267	23.7564
16.05243	9.943005	8.461172	7.180713
3.84844	22.25466	23.38229	21.74196
0.334318	1.115311	1.283104	1.132864
74.39117	175.0347	172.0738	172.0909
1.310443	2.252723	1.496683	2.105572
6.392844	1.135829	1.481967	1.063558
11.76146	4.510295	4.28454	5.498769
51.94424	28.34411	27.07562	28.55395
2.087147	1.792719	3.045008	2.600148
27.82425	83.02744	80.75708	80.56614
1.653258	2.426443	2.218413	2.270416
7.338392	0.990115	1.402521	1.945274
0	0.977297	0.567539	0.765721
19.82696	11.83373	11.25777	12.4246
6.040131	21.56375	21.78254	21.16923
9.057996	28.14063	25.11555	25.51474
27.15025	6.917036	6.638605	6.367765
1.852477	6.070314	6.062148	5.397071
16.45269	33.08152	32.93936	35.52456
2.170696	4.773096	5.454085	4.605912
2.955565	1.6604	0.70715	0.720076
0.891106	0.666404	0.513752	0.538668
6.687507	2.837779	2.851333	2.760788
0.311164	0.863301	0.421445	0.570439
7.862393	6.525039	4.929594	3.56605
4.646641	1.982204	1.838089	1.96475
10.40205	7.261671	6.319434	6.745472

4.149156	12.67671	14.47667	14.55497
2.420057	3.338063	3.450767	3.93344
2.369866	1.18087	0.363119	0.723599
2.726561	7.42313	7.30933	8.188074
62.83585	31.91556	31.75554	33.43955
0.120553	9.369541	11.37159	10.55306
4.126155	2.526773	2.019204	2.118694
34.68016	5.999892	6.299268	7.149337
4.068391	2.518902	1.759676	2.014783
3.30487	7.815832	6.95596	8.28035
65.58135	143.0421	146.8711	148.8503
252.1371	28.63576	26.50563	31.97424
1.366872	2.573722	2.346725	2.760456
20.13056	105.468	101.0007	102.8388
40.16638	20.87434	21.74386	22.65681
3.673784	9.294677	8.808584	8.758741
0.03969	0.571543	0.857489	0.952613
17.86661	8.62121	8.484896	8.286984
381.7508	1604.071	1499.64	1341.769
39.3499	11.02813	9.913685	12.12746
9.684159	3.992886	4.257061	3.88482
1.824637	3.827604	2.886313	3.366962
1692.285	536.6565	548.1581	565.719
0.457687	0.385438	0.26618	0.46909
25.27446	12.42762	10.66892	13.51846
5.758606	10.29213	9.226129	11.41837
4.754025	3.024255	3.019748	3.05661
2.675782	7.403636	5.700008	7.038775
2.211191	0.965044	0.975794	1.12604
33.20082	87.37015	89.74164	93.74696
465.467	157.9847	153.9143	148.9952
24.81699	8.111033	7.423746	7.555735
0.25528	0.623452	0.561975	0.715099
4.900908	12.07674	8.710662	11.27022
0.139885	0.512144	0.482447	0.487433
3.732444	0.647822	0.977221	1.476169
11.27412	1.454609	1.967591	1.549333
2.192076	0.636184	1.659223	1.118323
4.179978	2.591233	3.019511	3.152421
3.998795	1.270431	1.880587	1.676126
12.06861	6.817011	4.377417	5.041321
19.39918	28.90143	26.69845	28.48673
35.17586	12.95141	11.99112	12.87939
1.246639	0.459089	0.649304	1.354875
0.409431	0.709777	0.591167	0.924053
1768.181	551.8369	508.599	555.9885
28.8008	12.44023	11.23438	11.77066
17.78547	7.358703	8.303422	9.028093
8.24334	3.301386	2.848086	3.262097
10.4873	5.198243	4.761904	5.095259
0	3.422609	3.636012	3.310791

27.80134	63.04538	61.70012	67.1512
22.05593	64.37739	69.05554	65.28059
35.28094	12.89221	10.48197	14.16271
438.7889	153.2966	147.6034	151.4621
6.766705	17.1629	17.63765	18.79333
5.016978	1.911883	1.994529	2.4898
3.792236	10.74763	10.04428	11.2461
3.685381	2.999486	3.592139	3.592618
0.569963	0.625105	1.017107	0.304374
4.075756	8.452395	8.108271	7.821547
1.341581	0.440149	0.563271	0.40066
54.97732	12.2047	14.16009	14.40855
38.64177	7.735991	8.415974	9.039192
1.565785	2.479717	2.525647	2.928796
10.1938	4.221286	3.668553	3.685961
5.751293	14.0961	13.56885	15.12593
215.4008	73.4553	65.15055	72.32601
3.880449	1.727598	1.456142	1.55874
0.536616	0.757361	1.063401	1.101928
2.888823	6.470263	6.642988	6.568736
9.812048	3.978704	4.658308	5.031635
0	0.403389	0	0
3.883357	0.828103	1.100978	1.30696
10.18331	14.2657	15.48877	17.50921
953.7079	237.6249	226.6318	255.3244
5.584091	49.29718	46.75631	49.84192
16.32343	133.9812	139.9175	140.7868
35.27839	75.18623	70.36047	74.24181
3.757781	1.886752	1.659539	2.62587
0.623342	0.38985	0.575423	0.690352
1.708958	4.316302	4.917587	4.899353
7.202597	3.987883	4.240503	4.386835
17.64677	9.233901	12.03234	11.63218
0.580707	3.193109	2.569165	1.835601
2.369453	1.041656	1.25266	1.175745
6.310646	11.09372	11.04254	11.05445
6.52122	3.298509	3.613375	3.773329
132.6061	41.94196	39.41505	44.39201
6.901162	10.38071	10.90014	12.05803
3.388809	9.689961	8.587993	9.713795
50.19738	23.64737	24.73309	27.77796
75.89854	1.744267	2.790683	1.64575
13.50346	1.508919	1.558072	1.925568
39.8468	53.81878	53.14613	53.03518
36.12058	18.61884	19.81305	16.53791
4.183551	15.13013	15.44183	15.87459
0.294747	2.217119	1.450233	1.730093
2.198447	4.165431	4.624887	5.137446
18.72129	40.76643	49.66163	53.25069
8.304012	2.498791	2.669975	3.432518
31.0208	117.1597	111.7281	118.0502

53.74246	22.32267	21.68532	20.99592
30.41212	9.238326	7.448125	8.51428
1.90364	3.022896	3.172238	3.453909
1.332299	7.135302	7.049663	7.319713
0.433826	0.992115	1.265925	1.407965
15.10741	60.9669	61.25163	61.16735
58.47376	31.1028	30.3384	29.98288
2.845252	5.806101	5.880039	5.776263
1.31354	0.558862	0.490459	1.074199
18.46476	3.433574	4.705149	4.803229
18.95513	97.26412	100.8568	95.52425
17.71462	9.369177	10.59559	8.955897
31.19205	13.79738	16.75636	15.58189
2.788049	1.216553	1.135009	1.25097
1.609598	3.958496	4.235879	2.730567
5.539759	2.578954	2.137086	2.66642
1.060876	0.242586	0.096772	0.050495
6.030965	13.94636	11.83362	8.837129
7.99205	27.18178	24.83873	25.24125
197.063	53.05358	50.76596	56.93841
10.80105	22.35755	21.56809	24.92439
0.289871	0.733945	0.72413	0.503195
3.119632	6.19466	5.67418	7.146297
3.327504	0.891195	0.65618	0.484688
2.945765	1.583071	1.138214	1.237896
214.5509	66.34737	64.53129	68.96009
72.16551	30.73136	30.75937	32.59189
0.31128	3.807128	2.11584	3.316511
2.081071	23.59854	24.42823	22.48498
1.713293	4.139364	4.787342	4.719584
0.305906	0.931113	1.010729	0.815634
13.49514	54.90428	57.92603	58.19937
10.35013	3.130744	3.401007	3.378488
1.752937	0.556405	0.698874	0.679186
28.84345	66.34181	69.06116	66.68633
12.23217	32.96581	36.70662	33.64351
0.706495	1.871651	1.752988	1.533234
0.658728	0	0.172799	0.226498
1.357933	7.121518	5.538762	5.962703
1.59617	10.171	10.49061	10.02815
81.77536	98.08213	99.97725	100.4489
1.125291	2.161793	2.195111	1.834907
42.8207	15.36931	14.15057	16.09309
4.41562	12.24582	12.67837	13.80793
0.868045	4.072698	3.813112	3.56425
8.460859	3.538434	3.753942	4.08971
5.431291	2.9308	3.284567	3.62153
0	1.729826	2.274285	2.178633
2.997128	1.502039	1.213841	1.227897
0.802481	0.181452	0.311784	0.26544
17.0364	64.47988	63.4468	66.82974

0.665982	0.694537	1.282551	1.159212
19.0143	7.605374	8.593517	7.463903
0.701398	0.752923	0.501962	0.676411
1.855519	8.458059	8.019937	6.404789
51.87223	19.97888	22.55897	22.40435
4.013629	0.383018	0.74976	0.508945
15.18299	102.8446	98.72078	103.5735
721.476	112.6406	93.33634	98.81789
5.540988	13.34028	13.47006	11.54345
6.446519	17.66885	18.86712	19.07605
18.14376	7.58845	6.458853	7.558278
18.35536	0.992955	0.744642	0.938511
11.17202	4.212919	4.059342	4.899947
268.0087	92.65849	95.83122	101.9781
20.29904	47.75501	45.539	48.16416
10.56923	18.19577	21.30055	20.80126
8.147607	2.947372	3.041387	2.772443
9.486016	3.470054	4.942953	4.867051
10.0941	23.89695	21.4607	25.07411
2.271634	6.918598	7.138327	7.235571
0.741798	0.426057	0.550862	0.475061
1.387343	2.495824	2.846813	2.886132
224.5646	91.54677	85.18853	92.70544
7.315	3.334243	2.615208	3.513892
1.535312	0.849934	0.730974	1.187774
1.259264	3.196065	3.184783	4.043991
2.791668	9.369637	8.799622	10.20563
1.403718	4.477426	4.072803	2.791287
5.637622	13.9412	16.51259	15.49778
0	0	0	0
17.74975	61.22343	64.69025	63.59479
9.05228	2.240482	1.627056	2.629766
0.24069	0.559235	0.291092	0.35999
3.338615	1.662039	1.183089	1.506589
10.23805	5.349995	4.898538	4.372729
26.54143	12.53451	13.36595	14.39885
84.54473	33.49085	32.36146	34.2566
11.74353	1.64094	1.191443	1.945809
12.33697	17.41316	17.4851	19.98876
0.416425	1.411602	0.502966	1.299451
6.083695	43.9024	39.18181	40.83519
57.36699	20.4321	18.63367	22.9072
40.59433	59.83592	63.49397	64.99242
78.47187	929.1101	899.3543	937.1401
5.754036	8.035828	8.378036	8.249116
4120.394	852.2867	934.7281	953.6797
0.126871	0.660949	0.649958	0.741014
24.44144	7.363645	7.57224	8.403454
4.048599	2.209576	1.815704	2.454976
179.9055	67.31238	62.14103	64.2053
275.4377	123.1637	122.1627	126.9191

1.606231	0.283297	0.344318	0.668141
0.123593	0.907524	1.16282	0.836916
8.650421	24.16482	25.3524	22.62977
0.749203	2.747789	3.069037	3.04528
1.775323	9.827584	9.687475	9.948703
35.30605	14.30073	13.7406	12.61725
346.1159	801.7579	798.0733	828.2183
32.8793	14.72872	14.36192	14.60762
0.268537	1.165626	1.100255	1.718757
0.235724	1.256138	1.059548	1.206455
3.804492	10.22761	9.82734	12.88653
12.3223	5.880425	6.72714	7.368186
0.510756	1.702099	1.434746	1.666736
1.052403	4.100403	4.095662	4.332448
3.579252	10.2405	9.459799	11.24645
5.487514	2.12707	2.539448	2.453206
41.59242	27.68057	25.47309	25.63877
3.698079	40.90792	47.19882	47.50133
34.31637	15.73381	16.30235	15.60539
1.124198	3.59816	3.31406	3.436219
466.2129	246.6118	227.3796	254.9236
0.511877	2.32968	1.902608	1.92683
8.933138	17.88128	17.36084	19.40568
34.37472	4.479862	4.04037	4.352212
5.233829	5.789392	5.771972	7.078969
19.95487	0.354782	0.810184	0.507105
65.74035	4.269388	4.52204	5.104782
9.216042	4.980056	4.575838	4.00354
10.21783	3.688629	2.301006	3.880349
8.489029	3.15679	2.311248	3.52176
5.037528	28.70018	28.92927	29.43261
772.6756	1578.606	1592.771	1661.823
0.203426	3.496962	2.859352	3.590141
8.281102	2.218474	2.376189	2.203963
1.262426	0.331219	0.209401	0.27101
0.500766	2.41616	2.36346	2.46753
3.424123	14.10731	14.14839	13.11273
8.075679	4.616428	4.044593	3.291706
0.372925	0.622336	0.625192	0.53885
0	0	0	0
1.611614	9.267379	9.571465	10.38052
2.031197	16.97584	16.29584	16.82452
17.50238	8.791661	6.799988	9.33956
0.497598	1.416682	2.521858	1.939198
2.332647	0.40621	0.545961	0.803413
1.516181	3.192823	2.506377	3.626215
3.417629	20.18609	17.79983	19.58391
2.925316	7.341985	6.555127	8.136511
0.507164	1.121642	0.899587	1.044773
4.523691	2.265584	1.84753	2.38075
64.60926	30.43752	31.62646	34.93762

0.807162	0.508593	0.358118	0.377346
0.630787	2.992704	3.117124	3.292211
1084.317	1331.244	9299.911	556.247
0.831476	0.474215	0.634213	0.624513
0.48127	1.306972	1.071639	1.235591
1.678283	0	0	0.028865
25.33781	30.08364	28.04937	33.07888
38.36722	6.970556	7.627173	7.559897
28.48628	144.3284	148.3104	151.4351
130.4325	76.41322	68.70879	70.75286
8.208584	5.395352	3.216345	3.809419
13.14658	3.083817	3.638903	3.431825
0.791768	23.20001	23.6261	23.23449
0.340659	1.359718	0.984041	1.552447
8.005009	17.83413	19.20046	16.20487
1.348248	1.674958	1.872173	2.348905
2.123187	5.252553	4.597783	4.700758
1.85004	6.709748	6.847662	6.013528
42.48814	10.56258	9.622034	10.39115
2.779703	1.498071	1.360176	0.90387
0.174592	0.756884	0.692588	0.560422
0.136928	0.385082	0.16256	0.485254
418.1728	179.1517	181.0006	190.6269
0.611446	0.732207	0.372657	0.652006
8.56517	20.0788	19.90492	19.56954
1.718798	2.487113	2.732821	2.4634
26.93954	4.556142	4.116638	4.313313
0.814986	3.527176	2.56523	3.866945
22.60365	53.08464	50.2596	52.6723
1.264956	4.993834	4.761905	6.094645
32.80183	24.21748	23.00351	25.4264
0.702777	0.17182	0.293129	0.232714
0.54244	3.634925	3.049896	3.991134
0.818648	3.679673	2.723356	4.301617
90.2803	44.28993	43.54474	45.04972
37.01619	3.211723	4.442566	4.289773
0.362529	1.699244	1.134192	1.810894
32.81967	6.473626	6.929993	5.677166
12.67373	18.41321	16.78844	16.57624
0.593827	0.924502	0.669057	1.002133
5.334406	34.27717	29.86078	33.01953
1.301746	6.556958	7.898451	8.380169
1.842253	0.888002	1.353408	0.794237
39.24217	106.8124	99.22702	109.663
7.893598	38.65219	23.22779	38.47109
767.0073	2006.178	1806.658	1957.753
65.92796	30.58498	27.31025	29.84982
11.34012	5.470867	6.071744	5.307851
0.634019	0.271833	0.310213	0.374263
5.94767	5.471336	5.42298	4.68548
466.3155	102.0499	110.9464	115.8706

8.952772	10.90863	11.74132	9.916089
52.23765	21.29666	20.49799	19.91436
6.28351	2.265661	2.629687	2.92871
7.60841	4.768673	4.298393	5.502215
2.522904	9.722862	7.182031	5.981599
