# Supplementary material for: Ectopic Expression of AeNAC83, a NAC Transcription Factor from Abelmoschus esculentus, Inhibits Growth and Confers Tolerance to Salt Stress in Arabidopsis
Source: Int J Mol Sci. 2022 Sep 5;23(17):10182. doi: 10.3390/ijms231710182 (PMC9456028; doi:10.3390/ijms231710182)
Supplement: Supplementary file 1 [file ijms-23-10182-s001.zip › Table S3.pdf]

Table S3. Primers for quantitative real-time (qRT) PCR.

Gene	Gene accession	Primers sequence (from 5′ to 3′)
AeActin	i2_HQ_OkraNGS_c92341/f5p2/2106	F: TTGTATTGCTCGACGTACCAA
		R: TTATACCCATCAACACGTCCC
AeNAC83	i2_LQ_OkraNGS_c8741/f1p2/2376	F: AAGAACCAAACTCATAGCCAT
		R: CTGCTTTCTTCGGAGTCGTC
AtActin	AT3G18780	F: GTCGTACAACCGGTATTGTGCT
		R: TGTCTCTTACAATTTCCCGCTCT
AUX1	AT5G01240	F: AGTGACCACTGTGAAGGTAAC
		R: CCTGCCTTCTCTATGATTCCCA
ARF	AT2G46530	F: GCCAAACAAGCTTAGAGCCAC
		R: TGTTCCATGTGACCTTGAGGG
SnRK2	AT2G23030	F: CAAACGAGCTTGTGGCTGTC
		R: CGACCCACGCTAGATATCCG
SnRK2	AT4G40010	F: GCCGGTAGATTCAGCGAAGA
		R: TTTCCGGGACAAGACTTCCG
ABF	AT1G49720	F: AAGGGCTCGAAAACAGGCTT
		R: TGACTTCACCTTCTTACCACGG
JAZ	AT1G19180	F: CTCGTGAAGGAGGGCAAACT
		R: TTGGGGTTGGTTGGTTTGGT
JAZ	AT5G13220	F: GGCGCTACCAGAAAAGACGA
		R: TGCTGCTTCATTAGCGACCT
MYC2	AT2G28160	F: GTCAAATCCGGTTCTGCACT
		R: AGCCAATGAACCAAAACGTGA
NPR1	AT4G26120	F: AAACGACCTTACGGGAACGG
		R: TGTTTGAAACGCACTTGGAGC
TGA	AT1G08320	F: ACTACAAATATATACACGCCCACA
		R: TATGGTTCGCCATCTCTACTCA
PR1	AT2G14610	F: ACGGGGAAAACTTAGCCTGG
		R: TTGGCACATCCGAGTCTCAC
PR1	AT4G33720	F: GGAGAGGTTGGGATGTGCAA
		R: GACCAGGACCACACAAACCA
