# Supplementary figures and images for: Ectopic Expression of AeNAC83, a NAC Transcription Factor from Abelmoschus esculentus, Inhibits Growth and Confers Tolerance to Salt Stress in Arabidopsis
Source: Int J Mol Sci. 2022 Sep 5;23(17):10182. doi: 10.3390/ijms231710182 (PMC9456028; doi:10.3390/ijms231710182)

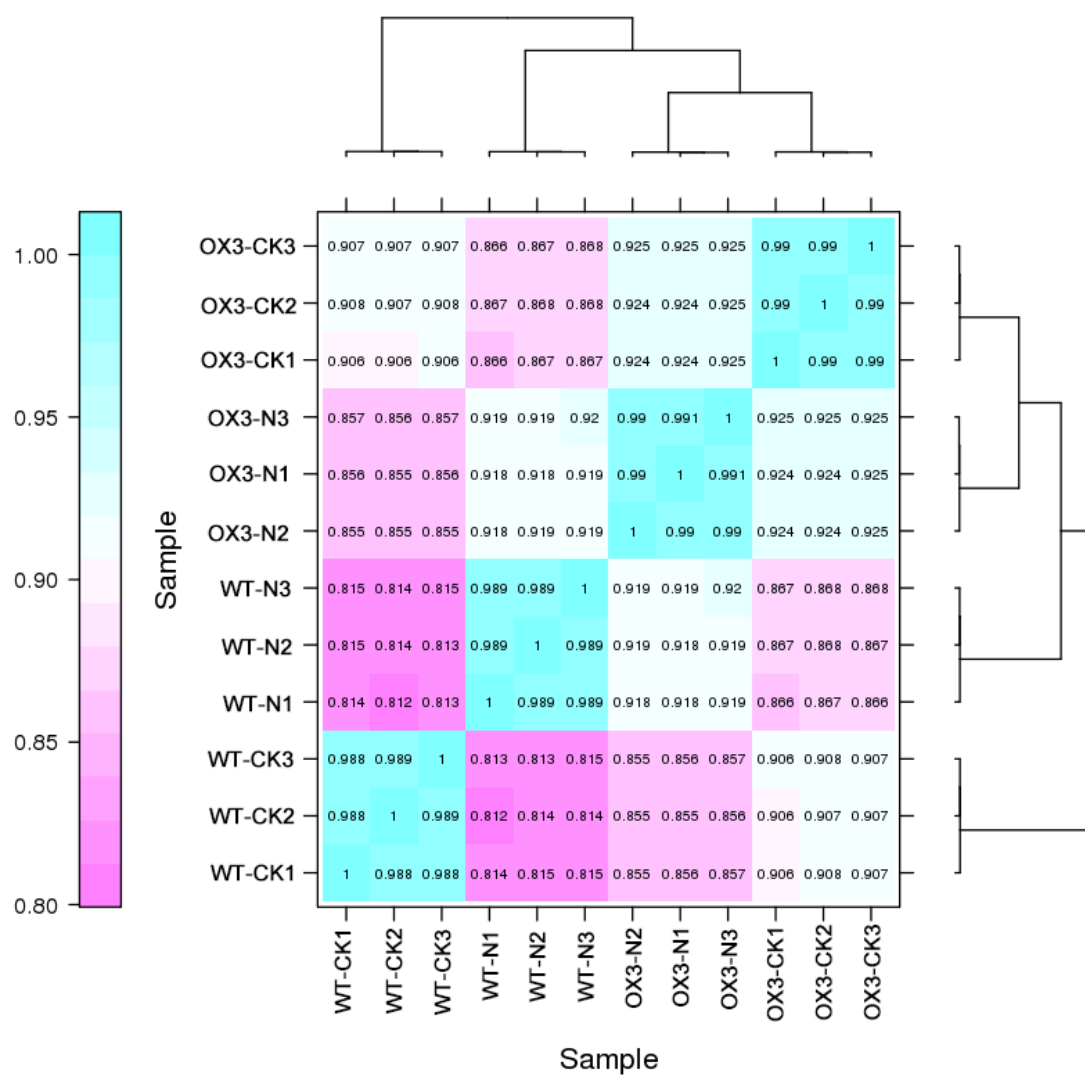

**Figure S1.** Heatmap of Pearson's correlation between samples.

Supplement: Supplementary file 1 [file ijms-23-10182-s001.zip › Figure S1.pdf]

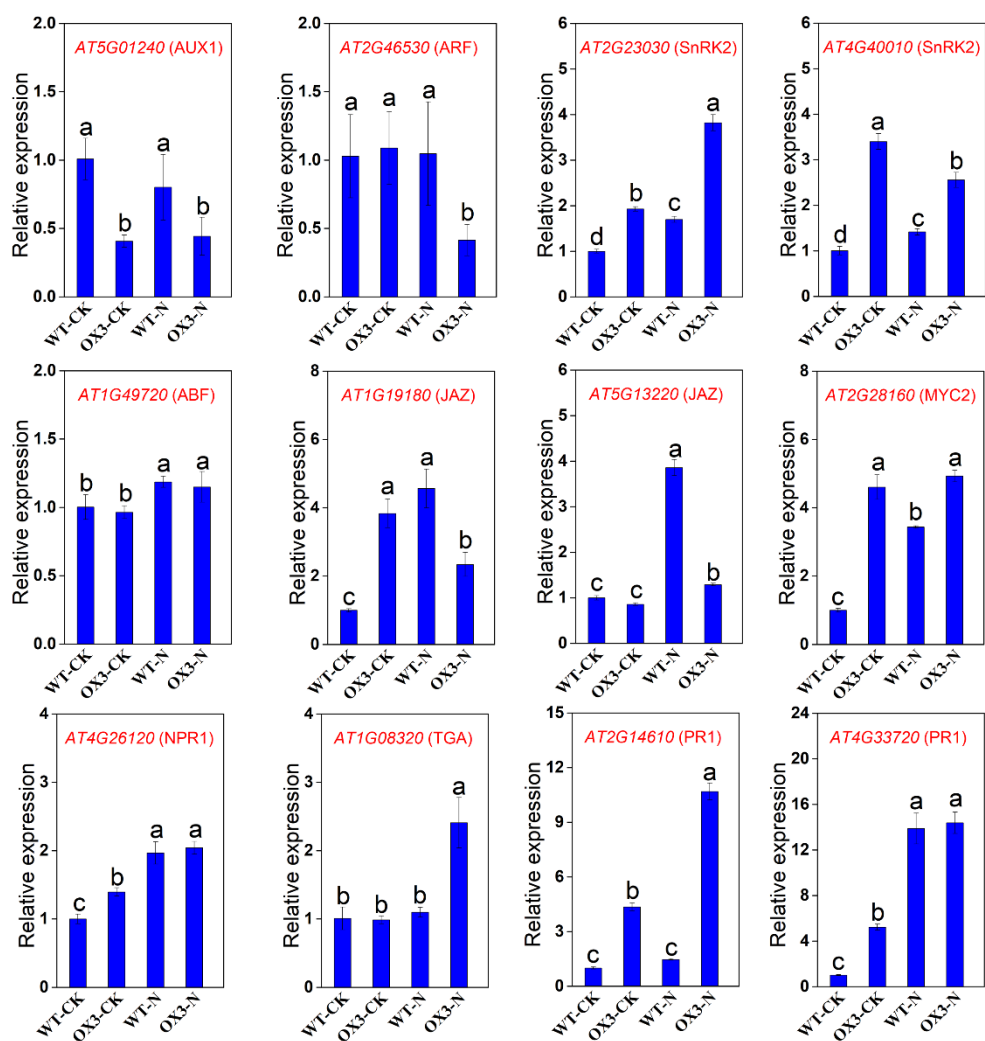

**Figure S3.** Validation of expression pattern of several hormone-related genes by qRT-PCR.

Supplement: Supplementary file 1 [file ijms-23-10182-s001.zip › Figure S3.pdf]
